# A platform to map the mind–mitochondria connection and the hallmarks of psychobiology: the MiSBIE study

**DOI:** 10.1016/j.tem.2024.08.006

**Published:** 2024-10-09

**Authors:** Catherine Kelly, Caroline Trumpff, Carlos Acosta, Stephanie Assuras, Jack Baker, Sophia Basarrate, Alexander Behnke, Ke Bo, Natalia Bobba-Alves, Frances A. Champagne, Quinn Conklin, Marissa Cross, Philip De Jager, Kris Engelstad, Elissa Epel, Soah G. Franklin, Michio Hirano, Qiuhan Huang, Alex Junker, Robert-Paul Juster, Darshana Kapri, Clemens Kirschbaum, Mangesh Kurade, Vincenzo Lauriola, Shufang Li, Cynthia C. Liu, Grace Liu, Bruce McEwen, Marlon A. McGill, Kathleen McIntyre, Anna S. Monzel, Jeremy Michelson, Aric A. Prather, Eli Puterman, Xiomara Q. Rosales, Peter A. Shapiro, David Shire, George M. Slavich, Richard P. Sloan, Janell L.M. Smith, Marisa Spann, Julie Spicer, Gabriel Sturm, Sophia Tepler, Michel Thiebaut de Schotten, Tor D. Wager, Martin Picard, The MiSBIE Study Group

**Affiliations:** 1 Division of Behavioral Medicine, Department of Psychiatry, Columbia University Irving Medical Center, New York, NY, USA; 2 Department of Clinical Neuropsychology, Division of Cognitive Neuroscience, Columbia University Irving Medical Center, New York, NY, USA; 3 Clinical and Biological Psychology, Institute of Psychology and Education, Ulm University, Ulm, Germany; 4 Department of Psychological and Brain Sciences, Dartmouth College, Hanover, NH, USA; 5 Department of Psychology, University of Texas at Austin, Austin, TX, USA; 6 Department of Psychiatry and Behavioral Sciences, University of California, San Francisco, San Francisco, CA, USA; 7 Center for Translational and Computational Neuroimmunology and the Taub Institute for Research on Alzheimer’s Disease and the Aging Brain, Department of Neurology, Columbia University Irving Medical Center, New York, NY, USA; 8 H. Houston Merritt Center for Neuromuscular and Mitochondrial Disorders, Columbia Translational Neuroscience Initiative, Department of Neurology, Columbia University Medical Center, New York, NY, USA; 9 Weill Institute for Neurosciences, Department of Psychiatry and Behavioral Sciences, University of California, San Francisco, San Francisco, CA, USA; 10 Department of Psychiatry and Addiction, University of Montreal, Montreal, Quebec, Canada; 11 Faculty of Psychology, Institute of Biopsychology, Technical University Dresden, Dresden, Germany; 12 Laboratory of Neuroendocrinology, The Rockefeller University, New York, NY, USA; 13 School of Kinesiology, University of British Columbia, Vancouver, British Columbia, Canada; 14 Consultation-Liaison Psychiatry, Department of Psychiatry, Columbia University Irving Medical Center, New York, NY, USA; 15 Department of Psychiatry and Biobehavioral Sciences, University of California, Los Angeles, Los Angeles, CA, USA; 16 Department of Psychiatry, Icahn School of Medicine at Mount Sinai, New York, NY, USA; 17 Brain Connectivity and Behavior Laboratory, Paris, France; Groupe d’Imagerie Neurofonctionnelle, Institut des Maladies Neurodégénératives-UMR 5293, CNRS, CEA University of Bordeaux, Bordeaux, France; 18 Robert N. Butler Columbia Aging Center, Columbia University Mailman School of Public Health, New York, NY, USA; 19 New York State Psychiatric Institute, New York, NY, USA

## Abstract

Health emerges from coordinated psychobiological processes powered by mitochondrial energy transformation. But how do mitochondria regulate the multisystem responses that shape resilience and disease risk across the lifespan? The Mitochondrial Stress, Brain Imaging, and Epigenetics (MiSBIE) study was established to address this question and determine how mitochondria influence the interconnected neuroendocrine, immune, metabolic, cardiovascular, cognitive, and emotional systems among individuals spanning the spectrum of mitochondrial energy transformation capacity, including participants with rare mitochondrial DNA (mtDNA) lesions causing mitochondrial diseases (MitoDs). This interdisciplinary effort is expected to generate new insights into the pathophysiology of MitoDs, provide a foundation to develop novel biomarkers of human health, and integrate our fragmented knowledge of bioenergetic, brain–body, and mind–mitochondria processes relevant to medicine and public health.

## Human psychobiology and energy

The psychobiological processes that sustain health or falter in disease are often referred to as ‘mind–body’ processes. This dualistic nomenclature is overly simplistic but reflects the interplay of evolutionarily rooted processes embedded in modern scientific culture and research approaches. Typically, subjective human experiences are the domain of the ‘mind’ without clear biological mapping, while the objectively quantifiable biological and physiological processes are the domain of biomedicine and of the ‘body’. Both are expressions of the same system [1]. Major gaps in knowledge remain to decipher how mind–body processes interact to allow individuals to enjoy long, meaningful, and healthy lives, or to exhibit increased disease risk.

To sustain health, the subjective mental states must constantly interact with the biological body–**brain** (see Glossary) system [2]. The central nervous system evolved to ensure physiological and energetic readiness to anticipated threats [3], accomplished by transducing subjective mental states into biological processes [4]. For example, psychosocial (dis)stress increases heart rate and blood pressure within seconds, elevates circulating glucocorticoids and blood glucose within minutes, and changes gene expression followed by cellular and organ-level recalibrations over hours to months [5]. All of these processes consume **energy** derived from **mitochondria** [6]. These energy-dependent brain-mediated pathways [7,8] act through neuroimmune and inflammatory processes [9], plus other psychobiological pathways integral to health and **resilience** [10]. Thus, energy-based mind–body processes are core determinants of human health [11,12].

Over time periods spanning months to years, subjective experiences ‘get under the skin’ and shape health outcomes. Positive and negative psychological states, social connections, loneliness, trauma, and chronic stress affect childhood development and growth [13], cardiovascular diseases [14,15], diabetes [16], some aspects of cancer biology [17], psychiatric disorders [18,19], neurodegeneration and dementia [20], wound healing and resistance to viral infections [21,22], biological aging [23,24], and lifespan [25–28]. Therefore, to achieve a satisfactory understanding of the forces that shape human health across the lifespan, we must build a science that integrates the constellation of mechanisms fueling our intertwined psychobiological experience. This requires the incorporation of mechanisms across levels, from social and environmental exposures to molecular mechanisms, within an integrative model that includes the crosstalk among the mind and mitochondrial **bioenergetics** [29] – or the mind–mitochondria connection.

In this article, we outline the rationale for the development of the Mitochondrial Stress, Brain Imaging, and Epigenetics (MiSBIE) study, a quantitative data platform to examine the mind–mitochondria connection in health and mitochondrial diseases (**MitoD**). We first illustrate some hallmarks of psychobiology and highlight general principles to guide studies aiming to link mind–body processes. We then discuss key aspects of the MiSBIE study design and protocol (available in full in the supplemental information online), describe the available outcome measures and biobank, and discuss outstanding questions for relevant fields ranging from psychology and neuroscience to immunometabolism and mitochondrial medicine. Together, this interdisciplinary work will contribute to elucidate the role of mitochondria and energy in human psychobiology, disease risk, health, and well-being.

## The mind–mitochondria connection

As pathways of mind–body interactions are uncovered, initial work has pointed to mitochondrial energy metabolism and signaling as key regulators of stress biology [30]. Mitochondria are energy-transforming organelles that transduce information across levels of complexity, from organelle to organism [31]. Mitochondria provide energy for the brain and all other tissues, including endocrine glands that orchestrate the elaborate interorgan crosstalk [6,32]. In turn, these energized systems sustain allostasis [33] – the anticipatory adaptation that ensures that our physiological states match dynamic mental states [34]. When threatened, stress responses promote recalibrations that drive reactivity and recovery to stressors, which shape endophenotypes determining vulnerability, resistance, and/or resilience over time [35–37]. However, stress responses and allostasis are energetically demanding. As a result, they can divert energy away from longevity-promoting growth, maintenance, and repair (GMR) processes [38]. In response to chronic stress, this diversion of energy from restorative processes towards stress response systems may account for the stress ➔ disease and stress ➔ aging cascades that shape the pace of aging [38]. These energy-restricted pathophysiological processes help us better explain how psychosocial stressors damage the body and mind [23,24,39] and how contemplative practices may promote restoration and healing [40]. Energy flow not only sustains but also instructs adaptive brain–body processes [31,41]. Thus, mitochondrial bioenergetics has emerged as a key substrate regulating how cells and organs are bound and function together as an integrated collective shaping the human experience.

The growing evidence that mind–body processes are regulated by mitochondria suggests that energy is a critical layer of analysis – or a dimension – that our molecular and anatomical reductionist models have overlooked [42,43]. This has led to the mind–mitochondria hypothesis, which proposes that *psychobiological processes link subjective human experiences to molecular and energetic processes within mitochondria* [30]. The connection goes both ways: mental states may influence mitochondrial biology, and mitochondria may influence psychological processes. If supported by empirical evidence, this would mean that a significant portion of the naturally occurring differences in mind–body processes that shape health and disease risk between individuals [5,44] are driven by inherited and acquired variations in mitochondrial biology and functioning.

## The hallmarks of psychobiology

**Figure 1.**
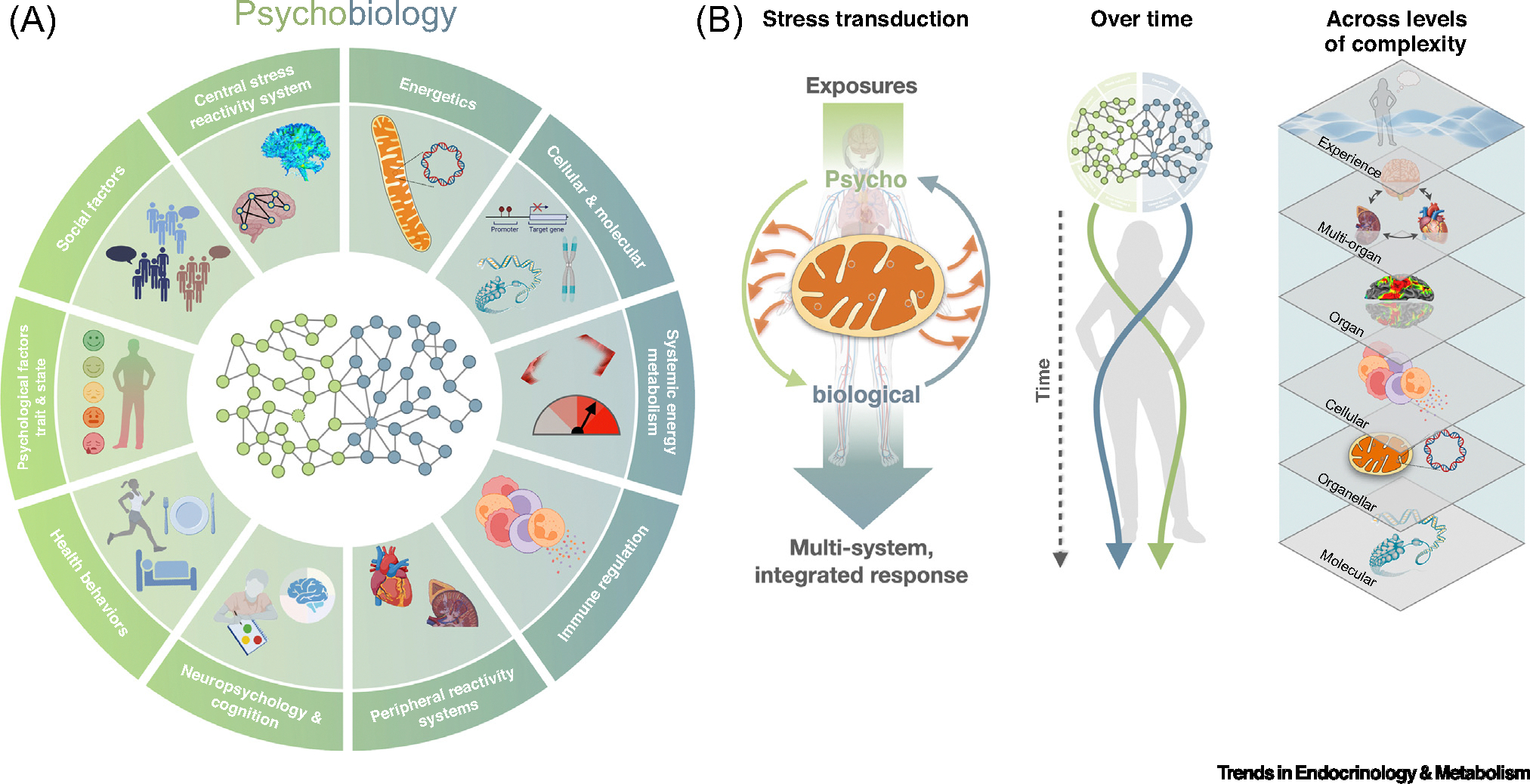
The hallmarks of psychobiology. (A) The hallmarks of psychobiology, separated for illustrative purposes, functionally interact as a psychobiological network of measurable elements (nodes) and functional connections (edges). Psychosocial factors and behaviors include mental states and attributes of the mind (green nodes). Biological processes (blue nodes) overlap and functionally interact with psychosocial factors in more complex ways than the 2D network illustrates. (B) Mitochondria functionally sit at the interface of psychological and biological processes that together transform exposures into integrated stress responses (left), evolve over time and must therefore be captured dynamically via repeated measures and time-series analyses (middle), and operate across levels of complexity (right). Some figure elements created using BioRender.

To bridge the mind–body knowledge gap outlined earlier, we need an integrative data platform that enables investigators to holistically examine interorgan, multilevel processes underlying human psychobiology and the subjective experience of energy across complex systems. We highlight ten hallmarks of mind–body processes for which there is at least some direct evidence that these processes either (i) contribute to the crosstalk between subjective experiences and objective biological processes or (ii) represent a demonstrated window to examine the health-relevant short-term or long-term consequences of mind–body processes (Figure 1).

1. *Energetics* include measures of mitochondrial biology ranging from least informative static molecular markers [e.g., mitochondrial DNA (mtDNA) copy number (mtDNAcn)] to more informative dynamic functional measurements [e.g., ATP production rates, reactive oxygen species (ROS) production] and signaling outcomes [e.g., cell-free mtDNA (cf-mtDNA) release] (for an overview see [30]). Over 30 mitochondrial functions and behaviors can be examined from omics data and direct functional measurements [45], including oxidative phosphorylation (OxPhos), which is a proxy for mitochondrial energy transformation capacity.
2. *Cellular and molecular processes* are the primary changes in the causal chain of processes that determine which genes are turned on or off, regulating their expression in a cell-type-specific manner [46]. Molecular marks including epigenetics (e.g., DNA methylation, histone modifications) tweak the properties of nodes within the organismal network, facilitating or hindering the acquisition of specific physiological and organismal states. Here we also include the microbiome, which refers to the symbiotic populations of microbes that populate the human body, contribute to its molecular makeup, and produce signals that influence brain function and psychological states [47,48].
3. *Systemic energy metabolism* is the dynamic metabolic state of the whole organism measurable from biofluids (glucose, lipids, other metabolites) [49], from exhaled gases (O_2_ consumption and CO_2_ production by indirect calorimetry) [50], or by other methods [51]. This integrated metabolic state emerges from the collective action of all organs and tissues coordinated by the mind.
4. *Immune regulation and inflammation* are changes in the composition or activity of the immune cellular repertoire, plus **cytokines** secreted by both immune and non-immune tissues [52]. Of particular relevance to psychobiology are the circulating immune cells (most directly accessible in blood), which are derived from primary and secondary lymphoid tissues and mobilized into circulation [53], as well as the signaling molecules (e.g., cytokines) released in response to acute and chronic psychosocial (dis)stress [54].
5. *Stress reactivity systems* include the major inducible ‘stress’ axes whose default state is inactive, but which exhibit robust pulsatile activity in response to psychological, social, physical, and other stressors [55]. The best studied systems are the hypothalamic–pituitary–adrenal (HPA) [56] and sympathetic-adrenal–medullary (SAM) [57] axes, which operate together with the sympathetic innervation of virtually every bodily tissue [58]. Stress axes are energy-mobilizing axes and interact with other systemic energy regulation systems [55], including, for example, growth differentiation factor 15 (GDF15) [59,60], extracellular ATP (eATP) [61], and others, which communicate the energetic state of a cell to other cells in paracrine or endocrine manner.
6. *Cognitive processes* include functions through which individuals make sense of and interact with the world, form and retrieve memories of meaningful events, and integrate bodily sensations (interoception) [62]. This also includes allostatic processes that regulate energetic demands and the ability to anticipate and plan for future occurrences [63]. Cognitive processes intersect with self-in-context models that endow events with personal meaning and allow predictive control over behaviors and peripheral physiology, including autonomic, neuroendocrine, and immune functions [64,65]. These give rise to situation-specific affective and emotional responses and are intertwined with other psychological factors.
7. *Health behaviors* include daily choices and habits of activities such as sleep, diet, exercise, and mind–body practices such as yoga and meditation. This also includes all lifestyle behaviors that influence mind and body processes and confer resilience against or risk for mental and physical illnesses [66].
8. *Psychological factors* incorporate trait- and state-level mental processes, or subjective experiences. Psychological traits are stable yet relatively malleable features of each person. They include personality traits like optimism, conscientiousness, and neuroticism; plus evaluative aspects of well-being such as life satisfaction and sense of purpose, and other relatively enduring characteristics that influence emotional and cognitive responses to challenges – linked to biological/physiological signatures and health [67–71]. Psychological states are more labile characteristics that include moods, emotions, and processes such as cognitive appraisals of threat and safety, emotion regulation, and rumination that are co-created by afferent inputs to the brain from the body, together with the social and physical environment that concurrently regulate peripheral physiology [72]. In this domain, we also include pain, which comprises the perception of physical pain and social or empathic pain, also important in the psychobiological foundations of disease [73].
9. *Social and environmental factors* include the social ecosystem that envelops the individual’s inner and outer world, particularly social stressors and support. This includes adverse exposures such as traumatic experiences, particularly during early development, such as abuse [74], stressful daily events (a social conflict), major life events (divorce, loss), and chronic ongoing stressors (discrimination, job strain) [67]. It also includes chronic psychosocial states such as social isolation and loneliness, as well as positive exposures such as actual and perceived social support and social safety [75–77]. Finally, the social exposome includes the broadest range of exposures from socioeconomic resources and status and the structural and physical environment as well (neighborhoods, environmental chemicals, and weather exposures), and social policies that influence health [78], social position, and cultural rituals [79,80].
10. *Brain anatomy and function* refers to the structural and functional properties of the brain as they emerge from the connectivity of anatomical areas that differ in their cell type composition, neurochemistry, activity patterns, and brain-wide connectivity [81,82]. The brain is the integration and predictive inference hub for sensory information [4], whose function is regulated by mitochondrial biology in a brain-region-specific manner [83–85]. The brain is directly or indirectly connected to every part of the organism through the peripheral nervous system.

These hallmarks of mind–body processes are not an exhaustive list of research-worthy topics. Rather, we outline these to paint the rough yet integrative contours of mind–body science and to illustrate the broad spectrum of relevant psychobiological processes. Each hallmark or facet of this model is imperfectly categorized as psychological (left) or biological (right), but together they subsume a set of well-studied variables that can be quantified – sometimes dynamically, over different timeframes. Some hallmarks are potential targets that can be perturbed to derive experimental evidence of their role in stress adaptation and resilience [86] or that can potentially be targeted therapeutically (e.g., neuroendocrine pathways, health behaviors) to enhance resilience and health.

We also highlight some core design principles integral to research aiming to examine, define, and understand dynamic psychobiological interactions (Box 1). These principles were central to the design of the MiSBIE study.

## The psychobiological network

These facets of psychobiology are interconnected through complex patterns of interactions among them. This is illustrated in the network of interconnected nodes and edges (i.e., graph) in the central portion of the diagram in Figure 1A. Each node represents a measurable domain of psychobiological function, and connecting edges represent a documented connection between two nodes.

High connectivity exists among both psychological and biological features. For example, in Figure 1 on the left side, the personality traits extraversion and openness to new experiences influence the tendency to socially affiliate with others and shape psychological states and moods in challenging situations, manifesting as idiosyncratic distributed brain functional connectivity patterns or signatures [87,88]. On the right side, a specific epigenetic imprint altering the expression of the glucocorticoid receptor in hippocampal neurons can blunt sensing of circulating cortisol, impair feedback signaling, and contribute to HPA axis hyperactivity [89], suppressing immune functions [90] and triggering excess energy consumption – or **hypermetabolism** [38,91]. Thus, all living systems exist as distributed networks of information exchange, using energy to bind elements together in an organized coherent whole [92,93]. Establishing the nature of these connections and their inter-regulation is a grand challenge for biomedicine [94,95] and the life sciences in general [10].

**Figure 2.**
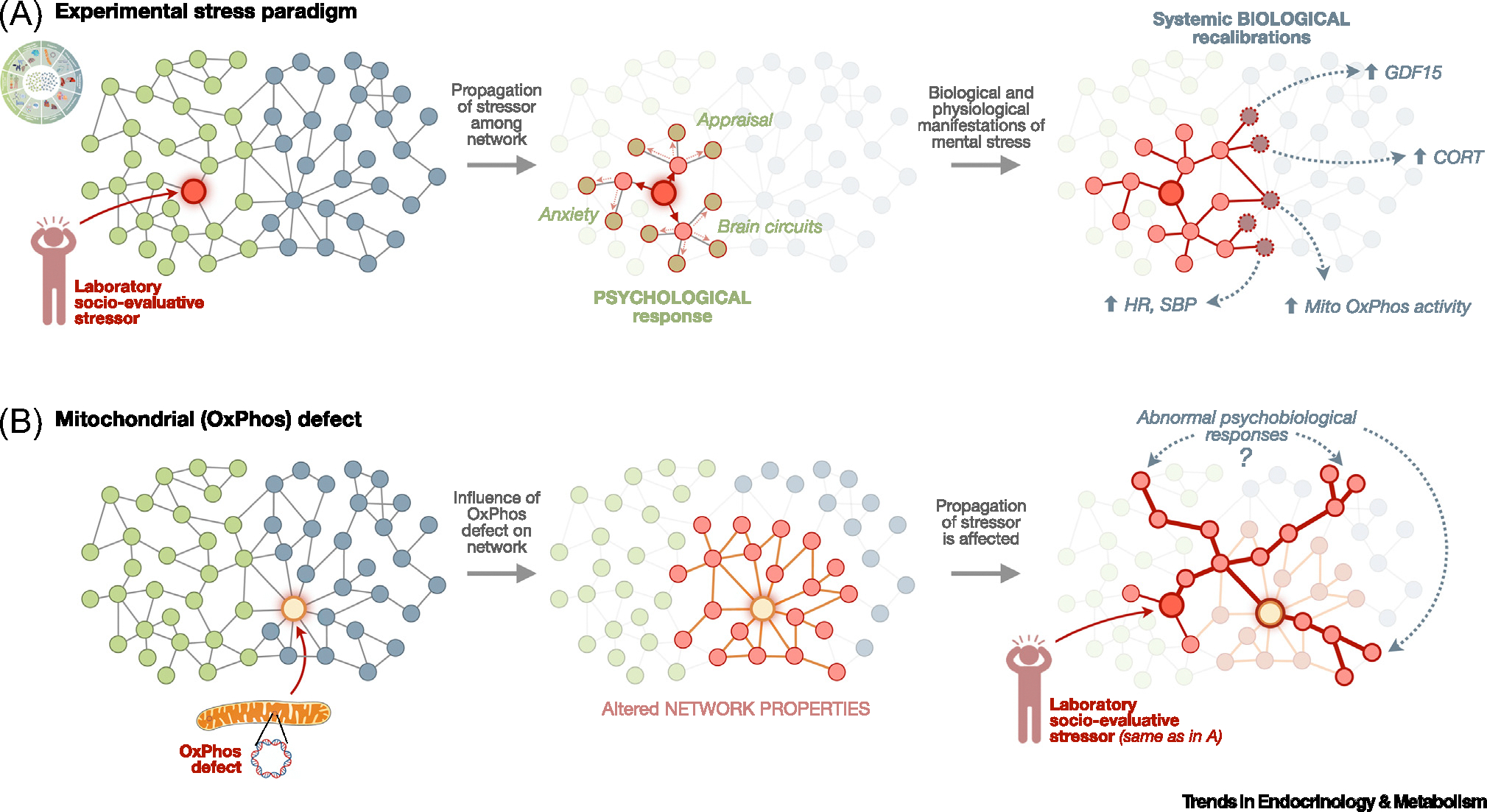
Model of mind–body processes emerging from information flow across the psychobiological network. (A) An experimental paradigm where a standardized laboratory psychosocial stressor [e.g., Trier Social Stress Test (TSST) speech task] triggers proximal psychological processes that ripple out into diverse brain–body systems functionally interconnected and regulated by mitochondrial oxidative phosphorylation (OxPhos). Pre-existing disturbances (e.g., hunger), disorders (e.g., anxiety), and symptoms (e.g., pain) affecting specific nodes of the psychobiological network not illustrated here also could influence both the perception of the stressor and the nature and magnitude of the response elicited by the stressor. (B) Study design where the mitochondrial OxPhos node is perturbed, as in primary mitochondrial diseases. This leads to cellular and physiological recalibrations that influence how the same stressor as in (A) can produce distinct (exaggerated, blunted, or qualitatively distinct) psychobiological responses. The 2D psychobiological network is a static simplification of the complex dynamical system that is the human organism. Abbreviations: CORT, cortisol; GDF15, growth differentiation factor 15; HR, heart rate; SBP, systolic blood pressure.

In terms of regulation, the activity among each hallmark necessarily entails energy consumption. This means that their existence and regulation are not only contingent on the availability of the brain–body hardware (nerves, glands, vasculature, etc.) but also require the constant and proper regulation of energy metabolism [29]. Overactivation of these systems can lead to hypermetabolism [38]. This reinforces the rationale for examining how the central hub of energy metabolism in breathing animals – mitochondria – regulates psychobiological processes. Figure 2 illustrates how a psychosocial stressor propagates or ‘ripples out’ across the psychobiological network and how altered mitochondrial biology may alter the propagation of the stressor.

## Evidence for mitochondrial psychobiology processes

Two main lines of research support the central role of mitochondria in mind–body processes. Here we provide a brief and minimalist overview of this rapidly developing literature.

### Mind ➔ mitochondria

Psychological stress and positive psychological states influence multiple mitochondrial functions measured in brain, immune, and other cell types [96–102]. The underlying mechanisms that transduce mental states into molecular changes in mitochondria remain mostly to be defined but certainly involve hormones acting directly on mitochondria, through the nucleus where they trigger bioenergetic recalibrations, or by diverting energy away from repair processes towards stress mechanisms [23,103,104]. Abnormal states of mind that alter behaviors, such as manic and depressive phases of bipolar disorder [105] and suicidality [106], are also linked to alterations in mitochondrial respiration and circulating metabolite signatures reflecting mitochondrial overload. Interestingly, both severe psychological distress in suicidality [107] and acute psychosocial stress trigger the release of cf-mtDNA [108,109]. Thus, acute and chronic psychosocial stress can alter mitochondrial biology and lead to **mitochondrial allostatic load (MAL)**.

### Mitochondria ➔ mind

Interindividual differences in rodent and human brain mitochondrial biology account for a sizeable portion of interindividual differences in complex social and anxiety-related behaviors and physiological stress responses [84,110,111]. Syngenic mice (i.e., ‘twins’ with the same nuclear genome) with different genetic mitochondrial perturbations respond differently to evoked stress, acutely mobilizing distinct multisystem strategies to the same external stressor [41]. This demonstrates that different aspects of mitochondrial biology are upstream regulators of stress appraisal, physiology, and/or other regulatory nodes. Moreover, experimental manipulation of mitochondria in specific neuronal types or brain areas confirms that mitochondria influence brain function and behaviors, as well as how an animal’s physiology responds to mental stress [83,112,113].

Clinically, primary genetic mitochondrial defects that impair energy transformation by the OxPhos system, such as those studied in MiSBIE, are associated with a greater rate of psychiatric symptoms [114,115]. The effectiveness of nutritional metabolic interventions – namely, nutritional ketosis – as a treatment for serious mental illness [116] offers converging, indirect evidence that mitochondria contribute to mental health in humans [117,118]. Moreover, in older adults, positive and negative psychosocial factors are linked to brain mitochondrial biology; specifically, OxPhos complex I protein abundance and gene expression in glial cells from the prefrontal cortex (DLPFC) [119].

Thus, animal and clinical studies suggest that the functioning of mitochondria is linked to subjective states of mind and to psychobiological stress responses. One robust test of this hypothesis in humans would involve examining interactions among the hallmarks of psychobiology in individuals exhibiting a spectrum of mitochondrial health, including individuals with MitoDs. If the hallmarks of psychobiology are differentially regulated in individuals with and without MitoD, or if their activity and/or interactions can be predicted on the basis of baseline differences in mitochondrial OxPhos capacity or another domain of mitochondrial functioning [45], this would provide support for the mind–mitochondria hypothesis. If specific psychobiological processes are unrelated to measurable mitochondrial properties, mitochondria (energy transformation or signaling functions) may not regulate specific domains of human psychobiology.

## What are mitochondrial diseases?

MitoDs are a heterogeneous group of disorders caused by inherited or sporadic molecular genetic mitochondrial defects generally affecting mitochondrial functions [120]. The majority of patients with MitoDs have maternally inherited or spontaneous mutations in mtDNA, although others have disorders caused by mutations in autosomal genes encoding proteins that reside and operate in mitochondria [121]. Mutations in mtDNA genes affect the synthesis of OxPhos proteins responsible for transforming chemical energy into the electrochemical gradient across the mitochondrial inner membrane, which ultimately powers ATP synthesis and several other functions [45]. Thus, genetic mitochondrial defects also can contribute to maladaptive mitochondrial recalibrations resulting in MAL in affected cells and tissues.

Because there are 100s to 1000s of mtDNA copies per cell, mtDNA mutations exist as a mixture of normal and mutant copies, a state termed heteroplasmy [122]. This generally varies between 0% and ~90%, as 100% pathogenic variants completely abrogates OxPhos activity and is therefore incompatible with life. A higher percentage of mtDNA mutation is generally associated with more severe disease burden [123], but the molecular–clinical correlation is imperfect [124]. Collectively, the heterogeneous clinical disorders and syndromes caused by OxPhos defects are termed mitochondrial disorders or MitoDs. On average, affected individuals have a life expectancy 30–40 years shorter than the average adult [125], notwithstanding pediatric cases where infants or children can fail to properly develop and die prematurely [126]. There are multiple causes of mortality in MitoD, with infectious conditions being pre-dominant [127].

Mitochondrial gene defects affecting OxPhos have profound consequences for the human mind–body system. Since every nucleated cell contains mitochondria, virtually all organ systems can be affected. Therefore, MitoDs are multisystem diseases associated with impaired cognitive function [128,129]. Although the underlying pathogenic mechanisms for OxPhos defects remain unclear, it is apparent that the cause of disease is not limited to ATP depletion, which rarely occurs *in vivo*[91]. Indeed, individuals with OxPhos-deficient mitochondria show exaggerated activation of cellular and tissue-level stress responses, such as the integrated stress response (ISR) [130] and angiogenesis that grows additional blood vessels around affected cells [131], likely as an attempt to restore health. Previous studies using exercise to understand the (patho)physiology of affected patients have identified exaggerated cardiovascular, respiratory, and endocrine responses to mild exercise challenge [132–134]. Thus, mitochondrial OxPhos defects appear to trigger abnormal or exaggerated physiological responses across organ systems to physical challenges, which may contribute to hypermetabolism, or chronically elevated resting energy expenditure among individuals with MitoD [91,125].

Thus, probing the hallmarks of psychobiology in women and men with rare genetic mitochondrial lesions causing MitoD offers a unique opportunity to: (i) develop a causal understanding of the role of mitochondria in the psychobiological processes that underlie human health; and (ii) discover potential modifiers of MitoDs that can help to improve care for affected patients.

## The MiSBIE study

The MiSBIE study was designed to address the role of mitochondria across the hallmarks of psychobiology. Its primary objective is to create a data platform of unprecedented depth in the domains of mitochondrial biology, psychobiology, psychoneuroendocrinology, psychoneuroimmunology, and other fields, which can be made available to the scientific community upon requests (see File S1 in the supplemental information online for an overview of the protocol and Table S1 in the supplemental information online for the dimensionality of the dataset). This National Institutes of Health (NIH)-funded, multiyear interdisciplinary research study (ClinicalTrials.gov #NCT04831424) is the human translation of preclinical studies in mouse models with distinct mitochondrial defects [41]. A key study design element is the inclusion of individuals with genetically defined MitoD (Box 2). The study inclusion and exclusion criteria are listed in File S2 in the supplemental information online. MiSBIE stems from an international collaboration addressing each hallmark of psychobiology and the core principles of psychobiology discussed earlier.

The MiSBIE study was designed to understand each participant as holistically as possible. The extensive 2-day MiSBIE protocol (Figure S1 in the supplemental information online) includes three broad components detailed in Box 3: (i) baseline measurements; (ii) stress reactivity measures that capture the reactivity and recovery of systems over rich time series of electrophysiology, biofluid (blood and saliva), and affect ratings (visual depiction of measurement parameters and intervals in Figure S2 in the supplemental information online); and (iii) home-based assessments that capture diurnal saliva hormone patterns together with self-reported psychosocial factors (Home Logbook in File S3 in the supplemental information online) and actigraphy to objectively assess physical activity and sleep behaviors.

The supplemental material associated with this article provides details around all elements of the study design and database. These include psychosocial questionnaire packages covering broad domains (demographics, health related-behaviors such as sleep and exercise, physical symptoms, aging, social life, personality, stress, affect/mood, and mental health) strategically administered across the 2-day visit (Table S2 in the supplemental information online), biospecimen collection and cryostorage procedures (Figure S3 in the supplemental information online), laboratory methods and assays for processing biofluids and isolating and cryopreserving immune cells, mitochondrial phenotyping on fresh and frozen immune cells, clinical assessments of disease severity and functional capacity, an extensive neuropsychological assessment covering multiple domains (e.g., premorbid functioning, intellectual functioning, visuospatial, language, memory, executive functioning and attention), and a 2-h **neuroimaging** session for structural (T1/T2), functional [(blood-oxygen level-dependent (BOLD) functional magnetic resonance imaging (fMRI)], and diffusion-based imaging of white matter anatomy, among other procedures described in full in File S4 in the supplemental information online.

**Figure 3.**
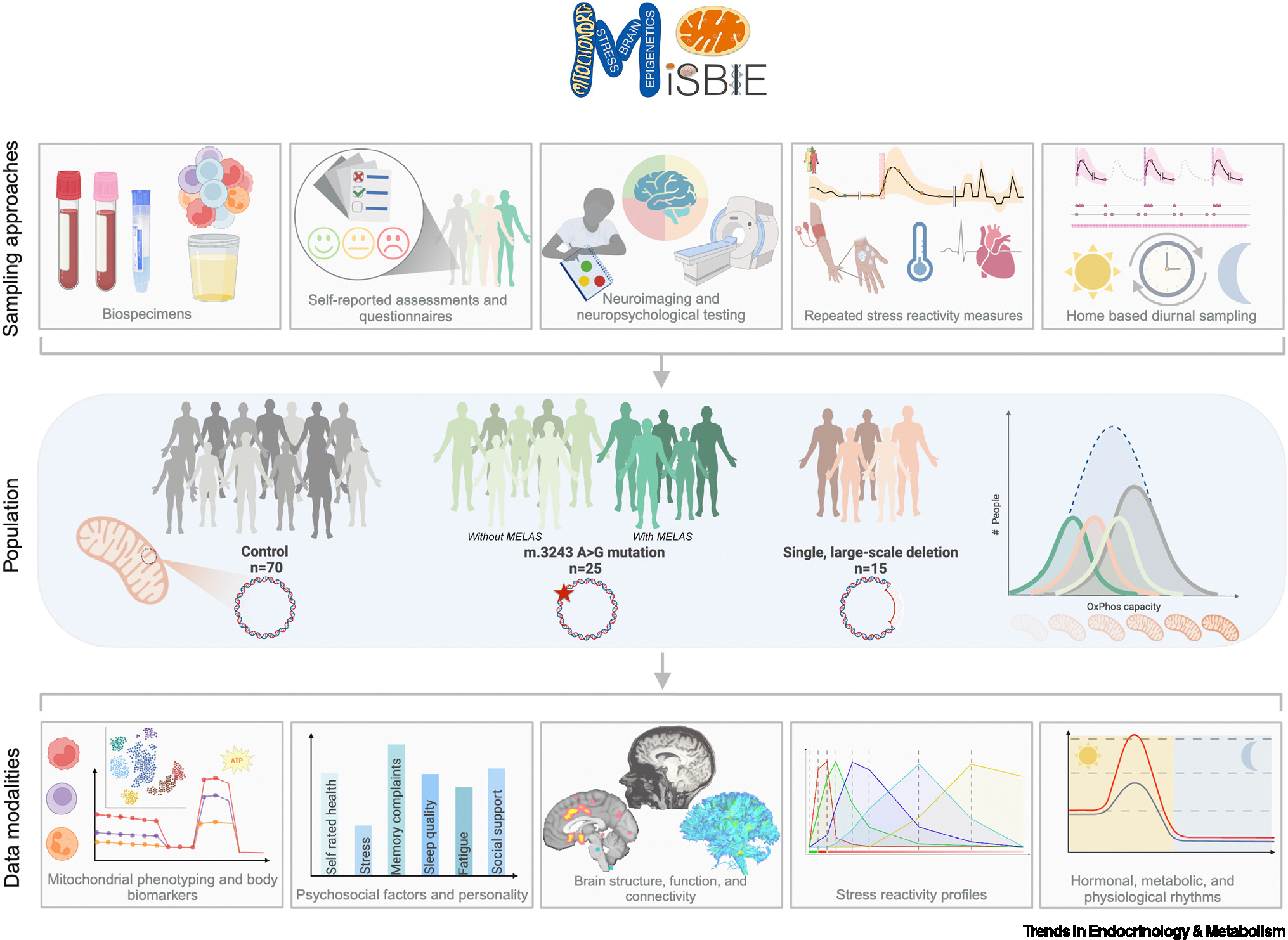
The Mitochondrial Stress, Brain Imaging, and Epigenetics (MiSBIE) study. Top: multimodal approach to sample and data collection addressing the hallmarks of psychobiology via clinician-assessed signs and symptoms, self-reported outcomes, rich time series of psychophysiological signals, and diurnal/behavioral rhythms, in parallel with a rich biobanked tissues for every participant. Middle: the MiSBIE group composition includes individuals with two distinct rare, molecularly defined mitochondrial DNA (mtDNA) lesions and a control group recruited from the community. The Mutation and Deletion groups exhibit distinct molecular and clinical phenotypes. Bottom: example outcome measures range from molecular and cellular bioenergetic profiles, single-cell transcriptomics, and neuroimaging to laboratory and home-based tracking of psychological, physiological, behavior, stress reactivity, and recovery. Abbreviation: OxPhos, oxidative phosphorylation. Figure created using BioRender.

The resulting unique MiSBIE study biobank (File S5 in the supplemental information online) and database (Data Dictionary, File S6 in the supplemental information online) will allow investigators across diverse fields to relate common psychobiological outcomes to bioenergetic parameters and clinical features. Specific questions of high priority that MiSBIE makes possible to address, among many others, are listed in the Outstanding questions. The MiSBIE study population, sample approaches, and data modalities are summarized in Figure 3.

## Study limitations

The MiSBIE study was carefully designed to enhance data quality and reliability through a highly standardized protocol that prioritized consistent meals, rest times, and activity levels, together with several other procedures implemented at each study visit (File S4). Nevertheless, the MiSBIE study has limitations. We highlight six main limitations that should be considered in this and other psychobiological studies.

1. Participants with mtDNA defects often present clinically with comorbid medical conditions that are treated or palliated with medications (i.e., often polypharmacy), which could influence psychophysiological parameters.
2. Recruitment was performed systematically to match control participants to participants with MitoDs based on four parameters: sex, age, physical activity levels, and ethnicity (to control for potential effects of mtDNA haplogroups [44,135]). This has yielded a relatively homogeneous sample driven in part by the more homogeneous ethnic distribution of our clinical populations. Nevertheless, the larger control group sample size allowed recruitment of ethnic minorities across the age range (37% of the control group is non-White).
3. Although the 40 participants with MitoD span a broad range of disease severity and symptoms, the demands of the MiSBIE protocol precluded the enrolling of some of the most severely affected participants with mitochondrial encephalopathy, lactic acidosis, and stroke-like episodes (MELAS) and Kearns–Sayre syndrome (KSS). Therefore, a portion of this sample present with relatively mild symptoms.
4. This first phase of MiSBIE is cross-sectional and does not enable us to draw conclusions about whether stress responses predict disease progression, for example. This limitation may be addressed in subsequent waves of follow-up.
5. Although each outcome measure was collected with the greatest possible rigor, the breadth and number of outcome measures made it impossible to measure each hallmark of psychobiology in as much detail as more focused studies would have enabled.
6. Depending on the study question, the total sample size (*n* = 110) is relatively small. Therefore, novel findings must be replicated in larger cohorts or future studies to establish their external validity and generalizability.

## Concluding remarks and future perspectives

We have described the rationale for examining how mitochondrial biology and bioenergetics in general influence mind–body processes and human health. MiSBIE represents, to our knowledge, the first large-scale transdisciplinary [136] effort to bring an energetic dimension into a psychobiology study. MiSBIE systematically examines this question using a two-pronged approach: (i) by exploring novel molecular- and physiological-level mind–mitochondria associations in healthy individuals with a naturally occurring spectrum of mitochondrial energy production capacity; and (ii) comparing healthy controls with individuals with rare molecularly defined **mtDNA lesions**, representing a unique scientific opportunity to directly evaluate the influence of mitochondrial OxPhos capacity on both well-established and novel mind–body processes.

The MiSBIE study covers several hallmarks of human psychobiology and applies core interdisciplinary principles to quantitatively capture mind–body interactions. Therefore, the study protocol summarized earlier (available in full detail in File S4) represents a portable design and foundation for future cross-sectional and longitudinal studies. The MiSBIE data platform represents a unique opportunity to formulate and test several novel hypotheses linking subjective human experience with molecular, biological, physiological, cognitive, behavioral, and other processes reflecting the (inter)action of multiple organ systems over time (see Outstanding questions). MiSBIE is an initial step in connecting the science and the human experience of energy.

What can we learn from interrogating the mind–mitochondria question in MiSBIE? Immediate potential outcomes of MiSBIE and future mitochondrial psychobiology studies include new insights spanning three main levels.

1. *Personalized medicine*: Realizing the promise of personalized medicine requires understanding the origins of interindividual variation in human health, including how people respond to challenges [95]. MiSBIE will systematically map organelle-to-organism determinants of individual differences in stress reactivity and recovery, thereby informing an energy-focused framework to integrate brain–body processes with biomedicine’s core molecular and cellular focus.
2. *Mitochondrial medicine*: MiSBIE will identify new potential pathophysiological mechanisms for genetic MitoDs and their clinical manifestations. This includes initial evidence to either disprove or support the stress ➔ disease cascade and the discovery of novel disease biomarkers in multiple biofluids, which may eventually improve diagnosis and/or treatment strategies.
3. *Science of health*: By capturing the dynamic, multisystem properties that emerge from the complex interactions across psychobiological domains (brain, immune, energetics, etc.) and levels of analysis (molecule, cell, organ, whole person, interpersonal), MiSBIE establishes a general research framework to develop dynamic biomarkers of human health. Results will need to be projected and validated onto other populations and cohorts to establish their usability and generalizability.

Mapping the mind–mitochondria connection and building a holistic model of human health calls for science at the intersection of disciplines, where so much remains to be discovered. The MiSBIE study is a step in this direction. We call on investigators across fields to include a broad scope of psychological and biological measures, as outlined in Figure 1, and suggest that energetic principles outlined above and elsewhere [38,137–139] will help move us towards an integrative and actionable model of human health.

## Supplementary Material

MMC2 **File S2.** Inclusion and exclusion criteria.

MMC10 **Figure S2.** Stress psychophysiology session.

MMC9 **Figure S1.** Overview of the two-day MiSBIE protocol.

MMC7 **Table S1.** Overview and dimensionality of the MiSBIE study database.

MMC11 **Figure S3.** Biospecimen processing and storage.

MMC3 **File S3.** MiSBIE home logbook.

MMC12 **Appendix 1.** MiSBIE collaborators.

MMC5 **File S5.** Biobank details.

MMC6 **File S6.** Data dictionary v1.0.

MMC1 **File S1.** Summary MiSBIE protocol and outcome measures.

MMC8 **Table S2.** Questionnaire packages v1.0.

MMC4 **File S4.** Detailed protocol, step-by-step experimental procedures, illustrations, materials, and scripts v1.0.

## Box 1. Core principles of psychobiology

The following principles of psychobiology provide a roadmap for research design and implementation within the interdisciplinary science of psychobiology (Figure I).

### Principle 1: include ‘psycho’ and ‘bio’ domains of analyses

Psychobiological studies must embrace mixed methods and include multiple domains of analyses ranging from subjective experiences to the basic molecular operations of cells and organelles. Collecting psychological and biological measures in parallel, or manipulating one domain and monitoring the other, is the quintessential design element of human psychobiological studies required to map potential mechanisms of mind–body processes.

### Principle 2: include multiple levels of analyses

Both biological and psychological processes are nested in a complex hierarchy. Including multiple levels of analyses contributes to testing robustness through converging evidence or lack thereof, and to being more precise about the potential specificity that a specific psychobiological pathway may exhibit.

### Principle 4: to reveal regulation, perturb the system

Physiology is highly adaptable and capable of compensating or ‘covering up’ fairly high levels of dysregulation. This redundancy is likely to be responsible for the broad spectrum of healthy and preclinical phenotypes. Therefore, at rest, some deficiencies and regulatory mechanisms (e.g., how robustly a stress axis can be activated and turned off, how well normoglycemia is maintained) are not visible to standard assessments [140]. Thus, to reveal dysregulation and to recruit health-relevant psychobiological pathways that operate on transient timescales, the system must be perturbed.

### Principle 5: reach across disciplines

Although often challenging for investigators trained within the silo of a given discipline, psychobiological processes are best probed, analyzed, and understood through interdisciplinary lenses. Human mitochondrial psychobiology studies require clinical methods and approaches from cell biology and theory from disciplines ranging from bioenergetics, molecular biology, and psychosocial sciences to neuroscience, among others.

### Principle 6: capture interindividual differences

The mind and its underlying psychobiology exhibit uniquely idiosyncratic features. Historically, most analytical approaches have focused on deploying robust statistics to compare groups or deployed multivariate regression-based methods to capture generalizable differences and associations. Applying and developing new methods to identify with high confidence idiosyncratic and subgroups of ‘response patterns’ will get us closer to understanding the true nature of human psychobiological processes.

## Box 2. MiSBIE study groups

The MiSBIE study sample (*n*=110) includes four groups of people aged 18–60 years (69% females, mean age=38 years). Eligibility for each study group is determined based on inclusion and exclusion criteria listed in Supplemental File 2. Recruitment took place over a 5-year period, from June 2018 to June 2023.

- Group 1: Healthy women and men (Control, *n*=70) covering the normal range of mitochondrial energy production capacity.
- Group 2: Individuals with the most common pathogenic mtDNA point mutation (m.3243A>G, in the transfer RNA leucine 1(UUA/G) [tRNA Leu^(UUR)^], *n*=20) that impairs OxPhos and causes a spectrum of multi-system disease [141]. All participants in this group have the mtDNA mutation, and present moderate-to-mild symptoms of mitochondrial diseases.
- Group 3: These individuals also carry the m.3243A>G mutation but have a history of stroke-like episodes. They therefore have a clinical diagnosis of Mitochondrial Encephalopathy, Lactic Acidosis, and Stroke-like episodes (MELAS, *n*=5) [142]. Compared to Group 2, these individuals generally present more severe signs and symptoms and have a worse prognosis [143,144]. Since participants in Groups 2 and 3 have the same mtDNA mutation, for most analyses, they can be combined as the mutation group (Mutation, *n*=25).
- Group 4: Individuals with a single, large-scale mtDNA deletion, have a missing segment of the mitochondrial genome such that several mtDNA-encoded genes essential to the OxPhos system are deleted [145–147] (Deletion, *n*=15).

Individuals carrying either of the two mtDNA defects (Mutation or Deletion) exhibit variable degrees of OxPhos deficiency and divergent clinical phenotypes, for reasons that remain largely unexplained. Mutation individuals tend to exhibit a predominant neurological phenotype with cognitive impairment, ataxia, diabetes, deafness, and fatigue, MELAS being the most severe form of the disease [128]. On the other hand, Deletion individuals tend to present with myopathy, extraocular muscle paresis, pigmentary retinopathy, cardiac conduction block, and fatigue, giving rise to the clinical syndromes termed chronic progressive ophthalmoplegia (CPEO) or Kearns-Sayre Syndrome (KSS) [146].

Analogous to the early days of neuroscience where precise brain lesion studies allowed investigators to map the involvement of specific brain regions to specific behaviors [148,149], and to preclinical studies [41], the inclusion of two severe and distinct genetic mtDNA lesions in MiSBIE allows to precisely identify the involvement of distinct mitochondrial defects on psychobiological processes with an unprecedented degree of resolution and specificity in humans.

## Box 3. MiSBIE study components

The study is divided into three main components, distributed across a 2-day on-site visit, and an at-home data collection phase (Figure I).

### Component 1 – Baseline resting measures.

This includes baseline anthropometric, DNA, biofluid (plasma, serum, saliva, urine), immune cell mitochondrial phenotyping, clinical symptom assessment, mood, and psychosocial questionnaires (total 39 instruments, see Supplemental Table 2), cardiovascular and body composition, neuropsychological testing, brain imaging, and whole-body resting oxygen consumption (1)Figure I). Together, these measures create an interdisciplinary, multi-system, and multi-level database to understand each person as holistically as possible. The stated goal of the study, as described to participants at the time of recruitment and on Day 1, was “to understand you as a person”. The MiSBIE study visits took place from 9 AM – 5 PM, Tuesday and Wednesday, at Columbia University Irving Medical Center. The full study protocol is illustrated in Supplemental Figure 1.

### Component 2 – Stress reactivity measures.

This includes time-series of blood (plasma and serum), saliva, cardiorespiratory parameters (beat-to-beat heart rate, blood pressure, and respiratory rate), skin conductance, body surface temperature (3 locations), and positive and negative mood. For all parameters, the magnitude, fold-change, or other metrics of stress reactivity and recovery (e.g., slope, area under the curve (AUC)) can be computed (2)Figure I]. The stress psychophysiology session is illustrated in Supplemental Figure 2.

### Component 3 – Home-based assessments of circadian biology.

This includes salivary awakening response of cortisol, GDF15, and other biomarkers, morning-to-evening differences in salivary biomarkers in the ecologically valid home setting, and actigraphy-based estimates of physical activity and sleep health, including timing, quantity, subjective quality, and circadian functioning (e.g., rest-activity rhythms) (3)Figure I]. Daily self-reported mood and sample collection times were recorded in a home logbook deployed on a custom-designed app (Supplemental File 3).

## Outstanding questions

Are normal interindividual differences in the nature and magnitude of psychophysiological responses to socioevaluative stress driven by differences in mitochondrial phenotypes across individuals?

Do mitochondrial OxPhos defects causing MitoD (mutation, deletion) exaggerate or alter neuroendocrine stress reactivity to psychological stress?

Are the classic MitoD biomarkers GDF15, FGF21, lactate, and others sensitive to psychological stress?

Does acute psychological stress trigger the release of cf-mtDNA in blood and saliva, and under what timeframe?

Do mitochondrial OxPhos defects alter time perception and related mental experiences including fatigue, anxiety, and mood?

Is the brain processing of sensory, cognitive, and threat stimuli, measured as brain structural and functional connectivity patterns, altered by mitochondrial energetic defects?

Does MAL cause systemic allostatic load (AL) quantifiable with standard clinical blood biomarkers?

Are individuals with mitochondrial OxPhos defects more vulnerable to the biological embedding of stress and adverse experiences, including accelerated aging phenotypes to adversity?

Can we develop more sensitive and specific MitoD blood or saliva biomarkers using stress-reactive or awakening response dynamics, compared with baseline or convenience sampling?

Do genetic mtDNA defects alter OxPhos capacity in circulating immune cells (monocytes, lymphocytes, neutrophils, platelets)? Do immune cell bioenergetics reflect the (dys)function of other organ systems and correlate with disease severity?

Can we map coherent metabolite (human stress metabolome) and proteome (human stress proteome) signatures of acute psychological stress?

Longitudinally, can we predict disease risk or resilience based on mitochondrial phenotypes and/or psychological stress reactivity profiles? Is there evidence for a stress ➔ metabolism ➔ disease cascade in MitoD?

Do variations in mitochondrial bioenergetics and/or systemic metabolic signals restrain the possible state space of brain activity and of the human mind?

## Highlights

Mitochondria transform the oxygen we breathe and the food we eat to power all brain–body or mind–body processes that produce human experiences.

Experimental studies in animals with mitochondrial defects show that mitochondria influence the perception of stressors and/or the resulting psychobiological responses relevant to resilience and aging.

Different individuals exhibit qualitative and quantitative differences in their affective, neural, and physiological stress responses; these may in part be explained by interindividual differences in mitochondrial biology, a question not previously examined in humans.

The Mitochondrial Stress, Brain Imaging, and Epigenetics (MiSBIE) study includes participants with rare genetic mitochondrial lesions, plus psychobiological stress-response paradigms to examine the mind–mitochondria connection with as much specificity as possible in humans.

The MiSBIE dataset and biobank include deep, multivariate phenotyping covering the hallmarks of psychobiology, detailed in the supplemental information online for this article, and data can be requested by the scientific community from the MiSBIE team.

## Glossary

Allostasis: energy-dependent, anticipatory recalibrations triggered by potential stressors or actual environmental demands, to maintain stability of vital functional parameters. Allostasis is epitomized as ‘stability through change’.
Bioenergetics: coordinated ensemble of biochemical, enzymatic, and mitochondrial processes involved in energy transformation – from chemistry to electricity – fueling all cellular activities that support life and stress responses. The core of mitochondrial bioenergetics is the oxidative phosphorylation (OxPhos) system, which is partially encoded by the mtDNA.
Brain: organ responsible for integrating sensory information, including interoceptive signals, to energetically manage the organism through allostasis. Of all organ systems, the brain has the highest stable energy demand, consuming 20–25% of the total organism’s energy budget.
Cytokines: secreted molecules from non-immune and immune cells used to convey information from one physiological system to another, including GDF15 that signals cellular energetic stress from the body to the brain.
Energy: property of the system reflecting its capacity to perform ‘work’ or to change from one state to another. Energy takes many forms, including but not limited to electrical changes across the mitochondrial or plasma membrane and ATP produced by the OxPhos system in mitochondria.
Hypermetabolism: excess energy consumption relative to the optimal state of the cell or organism.
Mitochondria: family of diverse, multifunctional organelles populating the inside of every cell in the body, involved in energy transformation and signaling within and between cells and between organs systems.
Mitochondrial allostatic load (MAL): the added energetic cost and biological ‘wear and tear’ including the molecular, structural, and functional recalibrations that mitochondria undergo in response to genetic mtDNA lesions (in the context of MiSBIE) or other metabolic, endocrine, biochemical, behavioral, and psychosocial stressors.
Mitochondrial diseases (MitoDs): groups of medical conditions caused by inherited or acquired mitochondrial defects, often of genetic origin affecting either the nuclear (nDNA) or the mitochondrial (mtDNA) genome. MitoDs can affect all organ systems.
Mitochondrial DNA (mtDNA) lesions: analogous to neurological lesions ablating specific brain regions that taught us basic lessons about brain functions; mtDNA lesions include point mutations and deletions of the mitochondrial genome that have the potential to teach us about mitochondrial regulation of human psychobiology.
Neuroimaging: a family of techniques noninvasively measuring activity in the brain. Varieties include MRI based and positron emission tomography (PET), among others. MiSBIE includes three MRI-based types – (i) T1- and T2-weighted images that map gross anatomical structures and gray-matter variations, (ii) diffusion-weighted images that can map white-matter tracts and their variation, and (iii) fMRI-BOLD images that reflect regional brain signals related to blood flow and oxygen consumption on a second-by-second basis.
Resilience: capacity to respond and adapt at a minimal energetic cost and to fully recover function following a stressor.
Skin conductance: electrodermal activity (EDA), also known as galvanic skin response (GSR); measured as fluctuations in conductance (or the inverse of resistance) on the palm of the hand, reflecting sympathetic nervous system activation, measured continuously across the 3-h multi-stress session on MiSBIE Day 1.
Stress response: coordinated activation and deactivation of affective, neural, endocrine, immune, metabolic, cardiovascular, and other psychobiological systems triggered by a perceived or actual threat to one’s physical or psychological integrity. In MiSBIE, a 5-min socioevaluative speech task plus the effort- and pain-inducing stimuli were also used to evoke qualitatively distinct stress responses.
