# Supplementary material for: A platform to map the mind–mitochondria connection and the hallmarks of psychobiology: the MiSBIE study
Source: Trends Endocrinol Metab. Author manuscript; Available in PMC 2024 Nov 12. (PMC11555495; doi:10.1016/j.tem.2024.08.006)
Supplement: MMC2 — File S2. Inclusion and exclusion criteria. [file NIHMS2028739-supplement-MMC2.pdf]

Supplemental File 2

MiSBIE Study – Inclusion and exclusion criteria

Control Group

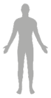

CRITERION	METHOD OF ASCERTAINMENT
**Inclusion**	
1. Individuals between the age of 18- 60.	Patient clinical records<br>Phone screen or clinical evaluation
2. Willing to provide saliva samples and have venous catheter installed for blood collection during the hospital visit	Phone screen or clinical evaluation
3. Willing to provide informed consent and capacity to consent	Self report
4. Use of effective method of birth control for women of childbearing capacity	Phone screen or clinical evaluation
5. English Speaking	Self report, Phone screen or clinical evaluation
**Exclusion**	
1. Individuals with cognitive deficit incapable of providing informed consent will not be included	TICS scores > 30, administered via phone screen or clinical evaluation
2. Symptoms of flu or other seasonal infection four weeks preceding hospital visit, as this would influence immune system parameters	Phone screen or clinical evaluation
3. Raynaud's syndrome (Rayneau phenomenon)	Phone screen or clinical evaluation
4. Involvement in any therapeutic trials listed on clinicaltrials.gov, including exercise	Phone screen or clinical evaluation
5. Metal inside or outside the body or claustrophobia prohibitive to MRI testing	Phone screen or clinical evaluation
6. Diagnosed with mitochondrial disease m.3243A>G, or large scale mtDNA deletion	Phone screen or clinical evaluation

Groups Mutation (m.3243A>G, no MELAS) & Deletion

CRITERION	METHOD OF ASCERTAINMENT
**Inclusion**	
1. Patients between the age of 18-60.	Patient clinical records<br>Phone screen or clinical evaluation
2. Willing to provide saliva samples and have venous catheter installed for blood collection during the hospital visit	Phone screen or clinical evaluation
3. Willing to provide informed consent and capacity to consent	Self report
4. Use of effective method of birth control for women of childbearing capacity	Phone screen or clinical evaluation
5. Harbours a mtDNA mutation. Either the m.3243A>G point mutation, or a single large scale mtDNA deletion.	Patient clinical records, or family history involving them as “obligate carrier” of the mtDNA defect
6. English Speaking	Self report<br>Phone screen or clinical evaluation
7. Confirmatory genetic test or willing to undergo genetic testing if one is not available.	
**Exclusion**	
1. Patients with cognitive deficit incapable of providing informed consent will not be included	TICS scores > 30, administered via phone screen or clinical evaluation
2. Neoplastic disease	Patient clinical records, and self-report
3. Symptoms of flu or other seasonal infection four weeks preceding hospital visit, as this would influence immune system parameters	Phone screen or clinical evaluation
4. Strokes & seizures.	Patient clinical records, and self-report
5. Raynaud’s syndrome (Rayneau phenomenon)	Phone screen or clinical evaluation
6. Involvement in any therapeutic trials listed on clinicaltrials.gov, including exercise	Phone screen or clinical evaluation
7. Clinical use of steroid therapy, which would impact the HPA-axis and other physiological systems (e.g., oral dexamethasone, prednisone, or similar)	Patient clinical records<br>Phone screen or clinical evaluation

Group Mutation (m.3243A>G) with MELAS

CRITERION	METHOD OF ASCERTAINMENT
**Inclusion**	
1. Patients between the age of 18- 60.	Patient clinical records<br>Phone screen or clinical evaluation
2.Willing to provide saliva samples and have venous catheter installed for blood collection during the outpatient visit	Phone screen or clinical evaluation
3. Willing to provide informed consent and capacity to consent	Self-report and clinician assessment
4. Use of effective method of birth control for women of childbearing capacity	Phone screen or clinical evaluation
5. Carries the m.3243A>G point mutation and has had at least one stroke-like episode, seizure, or both.	Patient clinical records, or family history involving them as “obligate carrier” of the mtDNA defect
6. English Speaking	Self-report<br>Phone screen or clinical evaluation
7. Confirmatory genetic test or willing to undergo genetic testing if one is not available.	Medical Records Genetic Test
**Exclusion**	
1. Patients with cognitive deficit incapable of providing informed consent will not be included	TICS scores > 30, administered via phone screen or clinical evaluation
2. Neoplastic disease	Patient clinical records, and self-report
3. Symptoms of flu or other seasonal infection four weeks preceding hospital visit, as this would influence immune system parameters	Phone screen or clinical evaluation
4. Raynaud syndrome (Raynaud phenomenon)	Phone screen or clinical evaluation
5. Involvement in any therapeutic trials listed on clinicaltrials.gov, including exercise	Phone screen or clinical evaluation
6. Clinical use of steroid therapy, which would impact the HPA-axis and other physiological systems (e.g., oral dexamethasone, prednisone, or similar)	Patient clinical records<br>Phone screen or clinical evaluation
