# Supplementary material for: A platform to map the mind–mitochondria connection and the hallmarks of psychobiology: the MiSBIE study
Source: Trends Endocrinol Metab. Author manuscript; Available in PMC 2024 Nov 12. (PMC11555495; doi:10.1016/j.tem.2024.08.006)
Supplement: MMC10 — Figure S2. Stress psychophysiology session. [file NIHMS2028739-supplement-MMC10.pdf]

Supplemental Figure 2

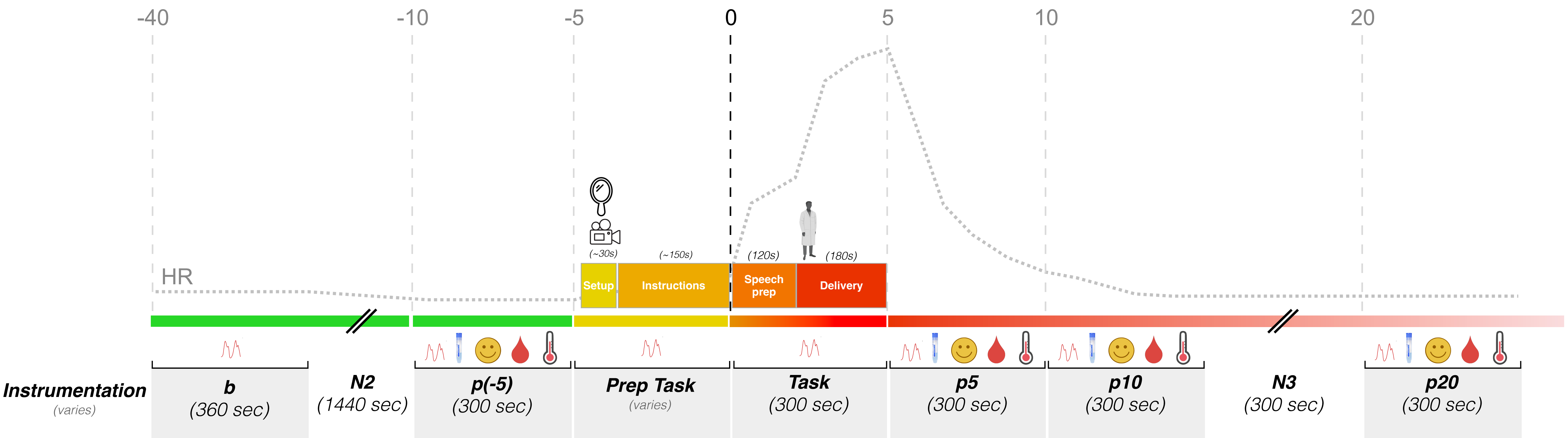

	b	N2	p(-5)	Prep Task	Task	p5	p10	N3	p20
**300s avg**	All variables		All variables	All variables	All variables	All variables	All variables		All variables
**60s avg**	HR, SBP/DBP, HFHRV, resp, EDA		HR, SBP/DBP, HFHRV, resp, EDA	HR, SBP/DBP, HFHRV, resp, EDA	HR, SBP/DBP, HFHRV, resp, EDA	HR, SBP/DBP, HFHRV, resp, EDA	HR, SBP/DBP, HFHRV, resp, EDA		HR, SBP/DBP, HFHRV, resp, EDA
**10s avg**	-		HR, SBP, DBP, EDA	HR, SBP, DBP, EDA	HR, SBP, DBP, EDA	HR, SBP, DBP, EDA	HR, SBP, DBP, EDA		-

p - Post
N - Nada (“nothing” period)
- Blood collection
- Saliva collection
- Affect rating
- Temperature recording
- Heart rate / blood pressure / breathing / skin conductance

Supplemental Figure 2 (continued)

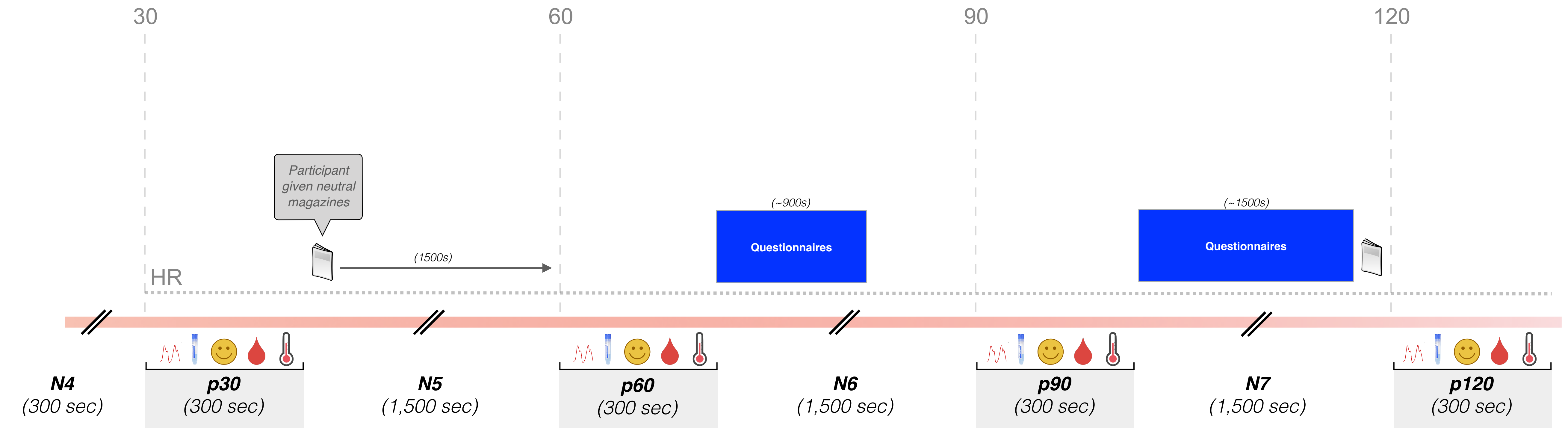

	p30	N5	p60	N6	p90	N7	p120
**300s avg**	All variables		All variables		All variables		All variables
**60s avg**	HR, SBP/DBP, HFHRV, resp, EDA		HR, SBP/DBP, HFHRV, resp, EDA		HR, SBP/DBP, HFHRV, resp, EDA		HR, SBP/DBP, HFHRV, resp, EDA
**10s avg**	-		-		-		-

p - Post
N - Nada ("nothing" period)
- Blood collection
- Saliva collection
- Affect rating
- Temperature recording
- Heart rate / blood pressure / breathing / skin conductance

Supplemental Figure 2 (continued)

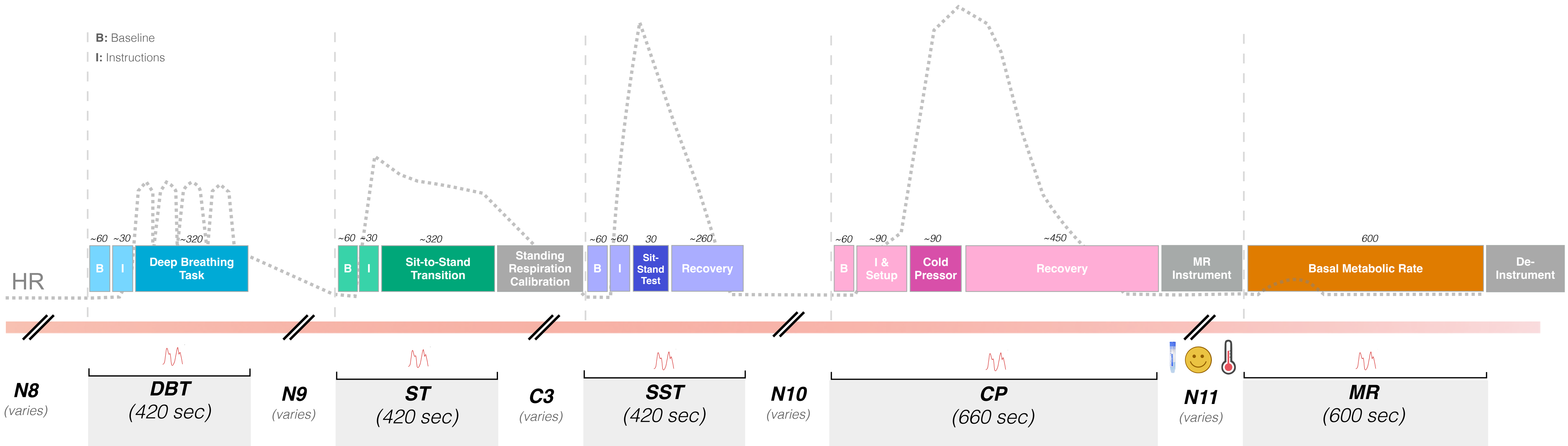

	DBT	ST	SST	CP	MR
**300s avg**	All variables	-	-	All variables	
**60s avg**	HR, SBP/DBP, HFHRV, resp, EDA	HR, SBP/DBP, HFHRV, resp, EDA	HR, SBP/DBP, HFHRV, resp, EDA	HR, SBP/DBP, HFHRV, resp, EDA	
**10s avg**	HR, SBP, DBP, EDA	HR, SBP, DBP, EDA	HR, SBP, DBP, EDA	HR, SBP, DBP, EDA	

p - Post
N - Nada (“nothing” period)
- Blood collection
- Saliva collection
- Affect rating
- Temperature recording
- Heart rate / blood pressure / breathing / skin conductance
