# Supplementary figures and images for: A platform to map the mind–mitochondria connection and the hallmarks of psychobiology: the MiSBIE study
Source: Trends Endocrinol Metab. Author manuscript; Available in PMC 2024 Nov 12. (PMC11555495; doi:10.1016/j.tem.2024.08.006)

## Supplemental Figure 1

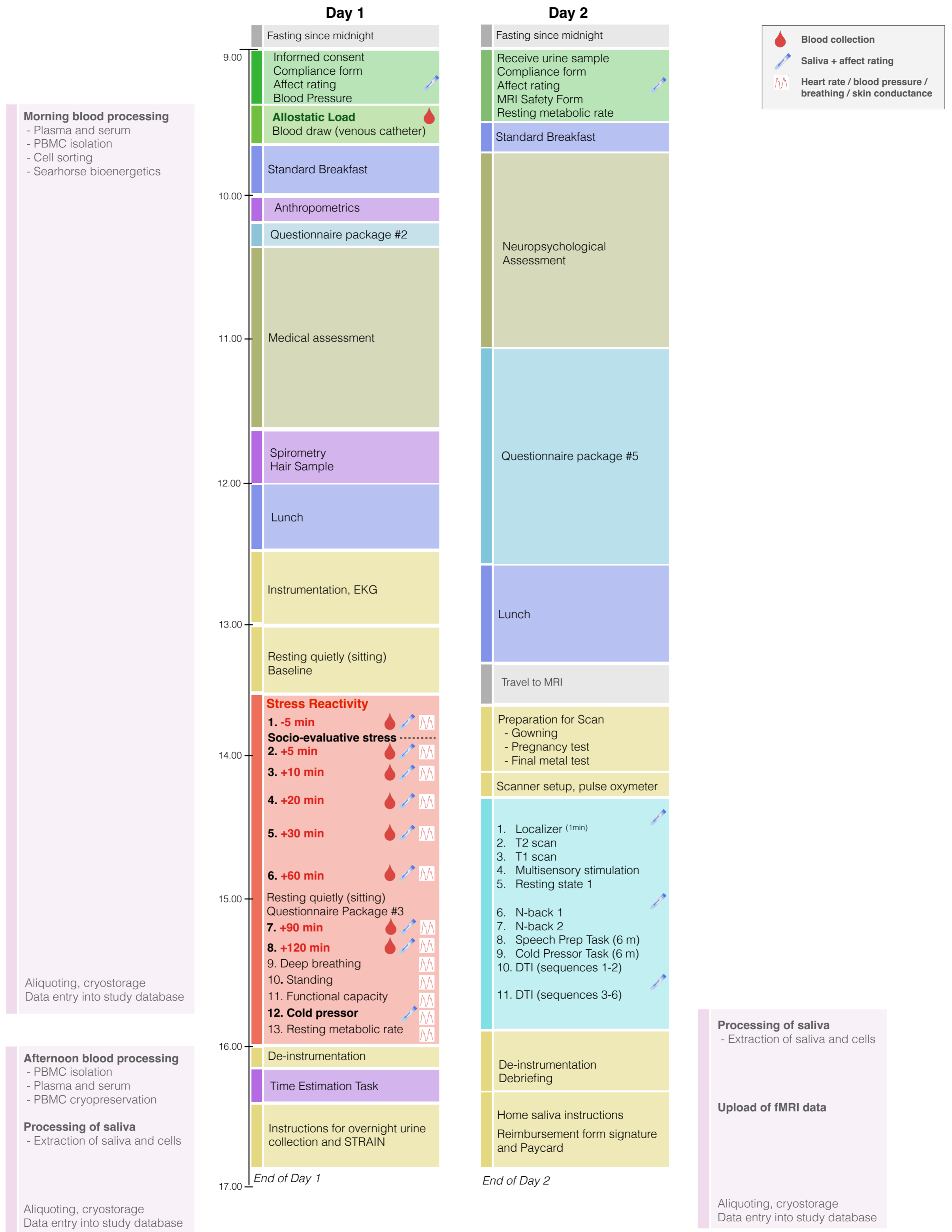

Supplement: MMC9 — Figure S1. Overview of the two-day MiSBIE protocol. [file NIHMS2028739-supplement-MMC9.pdf]

Supplemental Figure 3

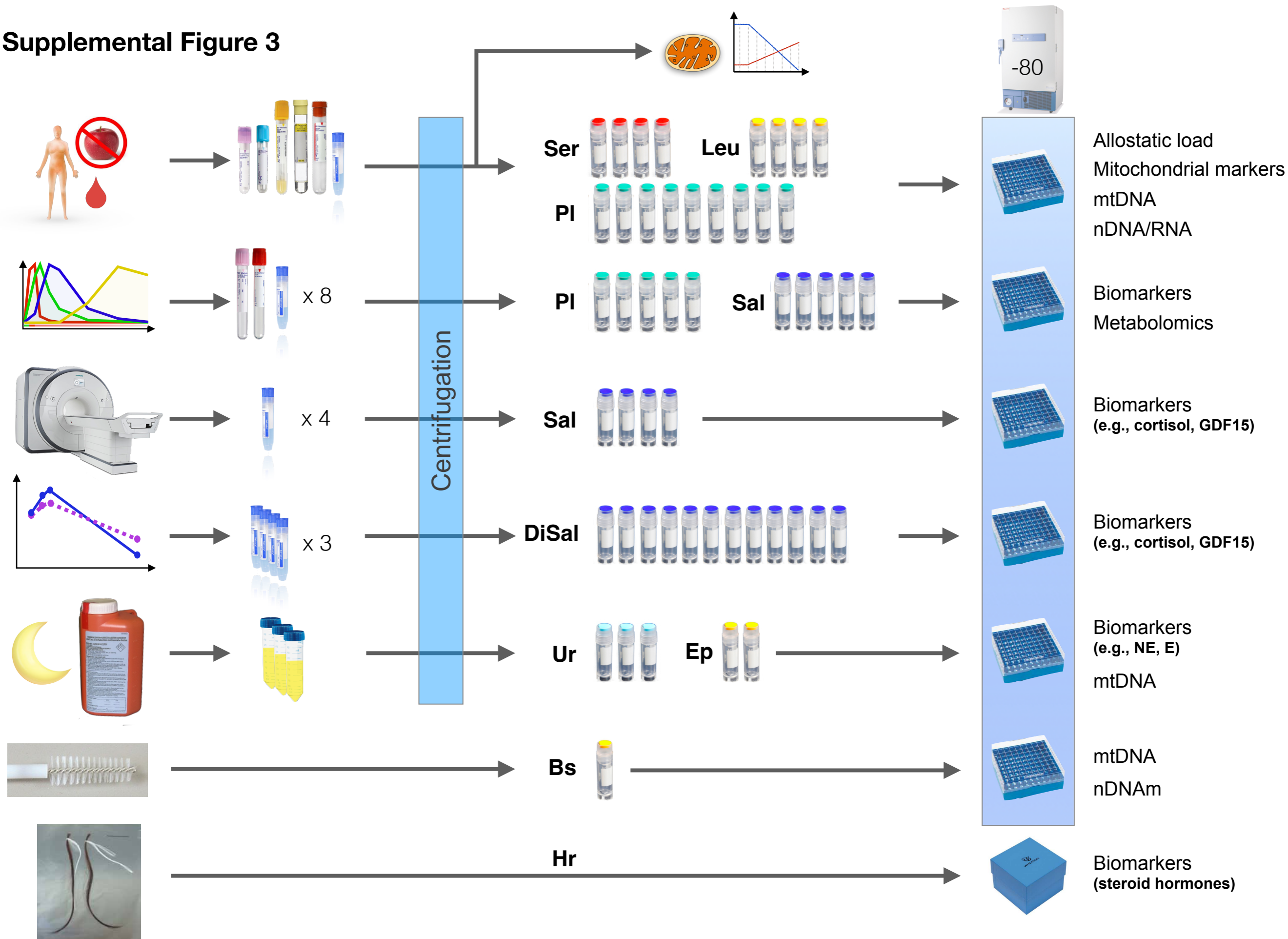

Supplement: MMC11 — Figure S3. Biospecimen processing and storage. [file NIHMS2028739-supplement-MMC11.pdf]
