# Supplementary material for: A platform to map the mind–mitochondria connection and the hallmarks of psychobiology: the MiSBIE study
Source: Trends Endocrinol Metab. Author manuscript; Available in PMC 2024 Nov 12. (PMC11555495; doi:10.1016/j.tem.2024.08.006)
Supplement: MMC7 — Table S1. Overview and dimensionality of the MiSBIE study database. [file NIHMS2028739-supplement-MMC7.pdf]

Supplemental Table 1

Overview and dimensionality of the MiSBIE study database.

Category	Instruments and forms	Scales and subscales	Items
Screening[1]	6	5	70
Medical assessment[2]	5	30	226
Anthropometric and other data[3]	2	3	80
Study-specific tasks[4]	1	4	24
Psychophysiology data[5]	1	82	1,652
Time estimation[6]	1	2	57
Neuropsychological[7]	9	19	123
MRI procedural[8]	1	N/A	59
Neuroimaging[9]	4	16	71
Questionnaires[10]	45	140	2,020
Biospecimen collection [11]	2	3	67
Biospecimen processing[12]	3	14	182
Biospecimen results[13]	7	32	1,386
Home based collection[14]	2	6	288
Procedural/study logistics[15]	2	N/A	66
Other[16]	2	1	17
**Total**	93	357	6,388

[1] Eligibility screening and enrollment forms including phone screening, eligibility checklist, genetic counseling
[2] Medical assessment on Day 1 including clinical scales: NMDAS, Crf Namdc, Clinical Frailty Scale, Karnofsky Scale
[3] Collected throughout Day 1 and 2 including blood pressure, heart rate, percent body fat, resting energy expenditure
[4] Records of timing and tasks administered on Day 1 including cold pressor, sit-stand task, and deep breathing
[5] Pre-processed continuous physiological data from Day 1 (heart rate, blood pressure, skin conductance, ventilation)
[6] Task to quantify time perception, includes interval estimation and production tasks administered on Day 1
[7] Battery of tests administered on Day 2 including DKEFS, WASI-II, TOPF, RBANS, NAB
[8] Documentation of imaging procedures including metal screening, instrumentation, discomfort ratings, scan timing
[9] Numbers represent the raw data files derived from structural and functional MRI; this represents an underestimate of the number of structural and functional neuroimaging variables that will be available after data preprocessing, parcellation, and primary analyses pipelines of structural, fMRI-BOLD, and diffusion-weighted imaging
[10] Questionnaires administered, 11 questionnaire packages are distributed over the study period
[11] Documentation of blood, urine, buccal cell, and saliva collection
[12] Procedural documentation of biological sample processing and quality for urine, saliva, buccal cell, and blood
[13] Biofluid analytes including steroid hormones, mitochondrial parameters, PBMCs levels
[14] Home sample collection including sampling instructions, questionnaires, biospecimen collection
[15] Records of visit timing and detailed procedural checklist
[16] Suicide severity rating scale, Day 1 and Day 2
