# Supplementary material for: A platform to map the mind–mitochondria connection and the hallmarks of psychobiology: the MiSBIE study
Source: Trends Endocrinol Metab. Author manuscript; Available in PMC 2024 Nov 12. (PMC11555495; doi:10.1016/j.tem.2024.08.006)
Supplement: MMC3 — File S3. MiSBIE home logbook. [file NIHMS2028739-supplement-MMC3.pdf]

Supplemental File 3

HOME LOGBOOK

Table of Contents:

Instructions and information .... page 2

Day 1 .... pages 3-6

Day 2 .... pages 7-9

Day 3 .... pages 10-13

Day 4 .... pages 14-16

Day 5 .... pages 17-20

Instructions for Return Shipment .... page 21

If you have experienced symptoms of a cold or flu in the past week, please contact us to schedule another week to collect saliva. **You should not collect samples if you do not feel well.**

Day 1<br>**Monday**	Day 2<br>**Tuesday**	Day 3<br>**Wednesday**	Day 3<br>**Thursday**	Day 5<br>**Friday**
**Awakening Saliva Sampling**<br>**Morning Questionnaire**<br>**+30 min Saliva Sampling**<br>**+45 min Saliva Sampling**<br>**Evening Questionnaire**<br>**Bedtime Saliva Sampling**	**Morning Questionnaire**<br>**Evening Questionnaire**	**Awakening Saliva Sampling**<br>**Morning Questionnaire**<br>**+30 min Saliva Sampling**<br>**+45 min Saliva Sampling**<br>**Evening Questionnaire**<br>**Bedtime Saliva Sampling**	**Morning Questionnaire**<br>**Evening Questionnaire**	**Awakening Saliva Sampling**<br>**Morning Questionnaire**<br>**+30 min Saliva Sampling**<br>**+45 min Saliva Sampling**<br>**Evening Questionnaire**<br>**Bedtime Saliva Sampling**

SCHEDULE FOR QUESTIONNAIRES AND SALIVA SAMPLING

SALIVA SAMPLING INFO: **Please place the saliva sample tubes, pen, Study Logbook, and timer by your bed the night before you start the saliva samples.**

<table>
<tr><th>Sample Number</th><th>Instructions</th></tr>
<tr><td>Sample #1
(immediately when you wake up)</td><td>Start this right when your eyes open or you are ready to get up for the day (and do not go back to sleep).
(Must take by 11:00am at the latest)</td></tr>
<tr><td>Sample #2
(30 minutes after you wake up)</td><td rowspan="2">PLEASE do not have caffeinated beverages (coffee, tea, soda, energy drinks, etc.) before taking these samples.
Do not eat or drink anything 10 minutes before collecting the sample.</td></tr>
<tr><td>Sample #3
(15 minutes after Sample #2)</td></tr>
<tr><td>Sample #4
(bedtime)</td><td>Take this sample right before bed.
Do not eat or drink anything 10 minutes before collecting the sample.</td></tr>
</table>

- Place the cotton your mouth, and carefully **roll it around** in your mouth with your tongue **for 2-3 minutes**. DO NOT bite it.
- Place the cotton back into the tube and securely place the **cap** back on the tube.
- Please place each tube **in the freezer** immediately after collecting it.

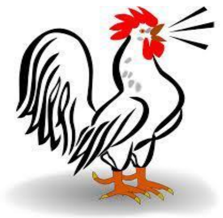

Date: ___________(Monday)

DAY 1 – Saliva Instructions

Upon waking please avoid having beverages (tea, coffee, soda) until you have collected sample #3. All samples must be stored in the freezer after they are filled. Please let us know the true sample time, even though you forget to do it in the order we requested.

Saliva Sample # 1: Immediately when you wake up

1) What time did you collect sample # 1? ___ ___: ___ ___ am/pm (circle one)

Avoid having caffeinated beverages (coffee, tea, soda, energy drinks) before sample #3 (in 45 minutes). It is fine to eat or drink something in the next 20 minutes, but **please** avoid doing so in the 10 minutes right before taking sample #2 or sample #3.

Saliva **Sample # 2:** 30 minutes after sample # 1

2) What time did you collect sample # 2? ___ ___ : ___ ___ am/pm (circle one)

3) Did you eat, drink or brush your teeth before collecting sample # 2? YES NO (circle one)

   a. If so, what: ____________________________

Saliva **Sample # 3**: 15 minutes after sample #2

4) What time did you collect sample # 3? ___ ___: ___ ___ am/pm (circle one)

5) Did you eat, drink or brush your teeth before collecting sample # 2? YES NO (circle one)

   a. If so, what: ____________________________

After collecting sample 3 you are allowed to brush your teeth and have beverages of your choice. You will collect sample #4 in the night before going to bed.

Saliva **Sample # 4:** before going to bed

6) What time did you collect sample # 4? ___ ___: ___ ___ am/pm (circle one)

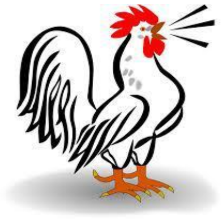

DAY 1 – Awakening

1) What time did you try to go to sleep last night (lights out)? ___ ___ : ___ ___ am / pm
(circle one)

2) What time did you wake up today? ___ ___ : ___ ___ am / pm
(circle one)

3) How would you rate the quality of your sleep last night?
   a) Very bad   b) Fairly bad   c) Fairly good   d) Very good

4) Please indicate by marking "X" on the line below to what extent you are looking forward to versus dreading today's events.

____

Really dreading today's events | *Neutral* | *Really looking forward to today's events*

5) Please indicate by marking "X" on the line below to what extent you are worrying about how things are going to go today.

____

At ease | Neutral | Worrying a lot

6) Please indicate by marking "X" on the line below to what extent you feel the events of your upcoming day are predictable (you know exactly what's going to happen today).

____

Completely Predictable | Completely unpredictable

7) I feel stressed, anxious, overwhelmed
   a) Not at all   b) A little bit   c) Somewhat   d) Moderately   e) Extremely

8) I feel in control, coping well, on top of things
   a) Not at all   b) A little bit   c) Somewhat   d) Moderately   e) Extremely

9) I feel joyful, glad, happy
   a) Not at all   b) A little bit   c) Somewhat   d) Moderately   e) Extremely

10) Did anything disturb your sleep last night? YES NO (circle one)
    Please describe: ____

    ____

11) Did you collect all samples this morning? YES NO (circle one)
    (why)______________________

DAY 1 – Evening

For each of the emotions listed below, please tell us how much you have felt that emotion this evening.

Not at all	A little bit	Moderately	Quite a bit	Extremely
0	1	2	3	4

___ 1. What is the most **amused, fun-loving**, or **silly** you felt?

___ 2. What is the most **angry, irritated**, or **annoyed** you felt?

___ 3. What is the most **ashamed, humiliated**, or **disgraced** you felt?

___ 4. What is the most **awe, wonder,** or a**mazement** you felt?

___ 5. What is the most **bored, disinterested,** or **uninvolved** you felt?

___ 6. What is the most **contemptuous, scornful**, or **disdainful** you felt?

___ 7. What is the most **control, coping well**, or **on top of things** you felt?

___ 8. What is the most **disgust, distaste**, or **revulsion** you felt?

___ 9. What is the most **embarrassed, self-conscious**, or **blushing** you felt?

___ 10. What is the most **excited, eager**, or **enthusiastic** you felt?

___ 11. What is the most **grateful, appreciative,** or **thankful** you felt?

___ 12. What is the most **guilty, repentant**, or **blameworthy** you felt?

___ 13. What is the most **hate, distrust**, or s**uspicion** you felt?

___ 14. What is the most **hopeful, optimistic,** or **encouraged** you felt?

___ 15. What is the most **inspired, uplifted**, or **elevated** you felt?

___ 16. What is the most **interested, alert**, or **curious** you felt?

___ 17. What is the most **joyful, glad,** or **happy** you felt?

___ 18. What is the most **love, closeness**, or **trust** you felt?

___ 19. What is the most **proud, confident**, or **self-assured** you felt?

___ 20. What is the most **rejected, betrayed**, or **left-behind** you felt?

___ 21. What is the most **sad, downhearted**, or **unhappy** you felt?

___ 22. What is the most **satisfied, fulfilled**, or **content** you felt?

___ 23. What is the most **scared, fearful**, or **afraid** you felt?

___ 24. What is the most **stressed, nervous,** or **overwhelmed** you felt?

___ 25. What is the most **tired, sleepy,** or **drowsy** you felt?

___ 26. What is the most **sexual, desiring,** or **flirtatious** you felt?

___ 27. What is the most **sympathy, concern,** or **compassion** you felt?

Date: ___________ (Tuesday)

DAY 2 – Awakening

1) What time did you try to go to sleep last night (lights out)? ___ ___ : ___ ___ am / pm (circle one)

2) What time did you wake up today? ___ ___ : ___ ___ am / pm (circle one)

3) How would you rate the quality of your sleep last night?

   a) Very bad   b) Fairly bad   c) Fairly good   d) Very good

4) Please indicate by marking "X" on the line below to what extent you are looking forward to versus dreading today's events.

____

Really dreading today's events — *Neutral* — *Really looking forward to today's events*

5) Please indicate by marking "X" on the line below to what extent you are worrying about how things are going to go today.

____

At ease — Neutral — Worrying a lot

6) Please indicate by marking "X" on the line below to what extent you feel the events of your upcoming day are predictable (you know exactly what's going to happen today).

____

Completely Predictable — Completely unpredictable

7) I feel stressed, anxious, overwhelmed

   a) Not at all   b) A little bit   c) Somewhat   d) Moderately   e) Extremely

8) I feel in control, coping well, on top of things

   a) Not at all   b) A little bit   c) Somewhat   d) Moderately   e) Extremely

9) I feel joyful, glad, happy

   a) Not at all   b) A little bit   c) Somewhat   d) Moderately   e) Extremely

10) Did anything disturb your sleep last night? YES NO (circle one)

   Please describe: ____

   ____

DAY 2 – Evening

For each of the emotions listed below, please tell us how much you have felt that emotion this evening.

Not at all	A little bit	Moderately	Quite a bit	Extremely
0	1	2	3	4

___ 1. What is the most **amused, fun-loving**, or **silly** you felt?

___ 2. What is the most **angry, irritated**, or **annoyed** you felt?

___ 3. What is the most **ashamed, humiliated**, or **disgraced** you felt?

___ 4. What is the most **awe, wonder,** or a**mazement** you felt?

___ 5. What is the most **bored, disinterested,** or **uninvolved** you felt?

___ 6. What is the most **contemptuous, scornful**, or **disdainful** you felt?

___ 7. What is the most **control, coping well**, or **on top of things** you felt?

___ 8. What is the most **disgust, distaste**, or **revulsion** you felt?

___ 9. What is the most **embarrassed, self-conscious**, or **blushing** you felt?

___ 10. What is the most **excited, eager**, or **enthusiastic** you felt?

___ 11. What is the most **grateful, appreciative,** or **thankful** you felt?

___ 12. What is the most **guilty, repentant**, or **blameworthy** you felt?

___ 13. What is the most **hate, distrust**, or s**uspicion** you felt?

___ 14. What is the most **hopeful, optimistic,** or **encouraged** you felt?

___ 15. What is the most **inspired, uplifted**, or **elevated** you felt?

___ 16. What is the most **interested, alert**, or **curious** you felt?

___ 17. What is the most **joyful, glad,** or **happy** you felt?

___ 18. What is the most **love, closeness**, or **trust** you felt?

___ 19. What is the most **proud, confident**, or **self-assured** you felt?

___ 20. What is the most **rejected, betrayed**, or **left-behind** you felt?

___ 21. What is the most **sad, downhearted**, or **unhappy** you felt?

___ 22. What is the most **satisfied, fulfilled**, or **content** you felt?

___ 23. What is the most **scared, fearful**, or **afraid** you felt?

___ 24. What is the most **stressed, nervous,** or **overwhelmed** you felt?

___ 25. What is the most **tired, sleepy,** or **drowsy** you felt?

___ 26. What is the most **sexual, desiring,** or **flirtatious** you felt?

___ 27. What is the most **sympathy, concern,** or **compassion** you felt?

Date: ___________ (Wednesday)

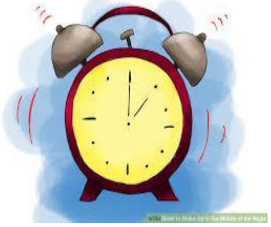

DAY 3 – Saliva Instructions

Upon waking please avoid having beverages (tea, coffee, soda) until you have collected sample #3. All samples must be stored in the freezer after they are filled. Please let us know the true sample time, even though you forget to do it in the order we requested.

Saliva Sample # 1: Immediately when you wake up

1) What time did you collect sample # 1? ___ ___: ___ ___ am/pm (circle one)

Avoid having caffeinated beverages (coffee, tea, soda, energy drinks) before sample #3 (in 45 minutes). It is fine to eat or drink something in the next 20 minutes, but **please** avoid doing so in the 10 minutes right before taking sample #2 or sample #3.

Saliva **Sample # 2:** 30 minutes after sample # 1

2) What time did you collect sample # 2? ___ ___ : ___ ___ am/pm (circle one)

3) Did you eat, drink or brush your teeth before collecting sample # 2? YES NO (circle one)

   a. If so, what: ___________________________

Saliva **Sample # 3**: 15 minutes after sample # 2

4) What time did you collect sample # 3? ___ ___: ___ ___ am/pm (circle one)

5) Did you eat, drink or brush your teeth before collecting sample # 2? YES NO (circle one)

   a. If so, what: ___________________________

After collecting sample 3 you are allowed to brush your teeth and have beverages of your choice. You will collect sample #4 in the night before going to bed.

Saliva **Sample # 4:** before going to bed

6) What time did you collect sample # 4? ___ ___: ___ ___ am/pm (circle one)

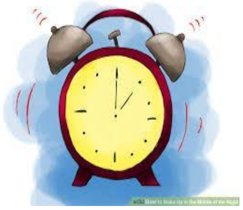

DAY 3 – Awakening

11) What time did you try to go to sleep last night (lights out)? ___ ___ : ___ ___ am / pm
(circle one)

12) What time did you wake up today? ___ ___ : ___ ___ am / pm
(circle one)

13) How would you rate the quality of your sleep last night?

a) Very bad b) Fairly bad c) Fairly good d) Very good

14) Please indicate by marking "X" on the line below to what extent you are looking forward to versus dreading today's events.

____

Really dreading today's events	*Neutral*	*Really looking forward to today's events*

15) Please indicate by marking "X" on the line below to what extent you are worrying about how things are going to go today.

____

At ease	Neutral	Worrying a lot

16) Please indicate by marking "X" on the line below to what extent you feel the events of your upcoming day are predictable (you know exactly what's going to happen today).

____

Completely Predictable	Completely unpredictable

17) I feel stressed, anxious, overwhelmed

a) Not at all b) A little bit c) Somewhat d) Moderately e) Extremely

18) I feel in control, coping well, on top of things

a) Not at all b) A little bit c) Somewhat d) Moderately e) Extremely

19) I feel joyful, glad, happy

a) Not at all b) A little bit c) Somewhat d) Moderately e) Extremely

20) Did anything disturb your sleep last night? YES NO (circle one)

Please describe: ____

____

21) Did you collect all samples this morning? YES NO (circle one)

(why)____________________

DAY 3 – Evening

For each of the emotions listed below, please tell us how much you have felt that emotion this evening.

Not at all	A little bit	Moderately	Quite a bit	Extremely
0	1	2	3	4

___ 1. What is the most **amused, fun-loving**, or **silly** you felt?

___ 2. What is the most **angry, irritated**, or **annoyed** you felt?

___ 3. What is the most **ashamed, humiliated**, or **disgraced** you felt?

___ 4. What is the most **awe, wonder,** or a**mazement** you felt?

___ 5. What is the most **bored, disinterested,** or **uninvolved** you felt?

___ 6. What is the most **contemptuous, scornful**, or **disdainful** you felt?

___ 7. What is the most **control, coping well**, or **on top of things** you felt?

___ 8. What is the most **disgust, distaste**, or **revulsion** you felt?

___ 9. What is the most **embarrassed, self-conscious**, or **blushing** you felt?

___ 10. What is the most **excited, eager**, or **enthusiastic** you felt?

___ 11. What is the most **grateful, appreciative,** or **thankful** you felt?

___ 12. What is the most **guilty, repentant**, or **blameworthy** you felt?

___ 13. What is the most **hate, distrust**, or s**uspicion** you felt?

___ 14. What is the most **hopeful, optimistic,** or **encouraged** you felt?

___ 15. What is the most **inspired, uplifted**, or **elevated** you felt?

___ 16. What is the most **interested, alert**, or **curious** you felt?

___ 17. What is the most **joyful, glad,** or **happy** you felt?

___ 18. What is the most **love, closeness**, or **trust** you felt?

___ 19. What is the most **proud, confident**, or **self-assured** you felt?

___ 20. What is the most **rejected, betrayed**, or **left-behind** you felt?

___ 21. What is the most **sad, downhearted**, or **unhappy** you felt?

___ 22. What is the most **satisfied, fulfilled**, or **content** you felt?

___ 23. What is the most **scared, fearful**, or **afraid** you felt?

___ 24. What is the most **stressed, nervous,** or **overwhelmed** you felt?

___ 25. What is the most **tired, sleepy,** or **drowsy** you felt?

___ 26. What is the most **sexual, desiring,** or **flirtatious** you felt?

___ 27. What is the most **sympathy, concern,** or **compassion** you felt?

Date: ___________ (Thursday)

DAY 4 – Awakening

1) What time did you try to go to sleep last night (lights out)? ___ ___ : ___ ___ am / pm (circle one)

2) What time did you wake up today? ___ ___ : ___ ___ am / pm (circle one)

3) How would you rate the quality of your sleep last night?

   a) Very bad   b) Fairly bad   c) Fairly good   d) Very good

4) Please indicate by marking "X" on the line below to what extent you are looking forward to versus dreading today's events.

____

Really dreading today's events | *Neutral* | *Really looking forward to today's events*

5) Please indicate by marking "X" on the line below to what extent you are worrying about how things are going to go today.

____

At ease | Neutral | Worrying a lot

6) Please indicate by marking "X" on the line below to what extent you feel the events of your upcoming day are predictable (you know exactly what's going to happen today).

____

Completely Predictable | Completely unpredictable

7) I feel stressed, anxious, overwhelmed

   a) Not at all   b) A little bit   c) Somewhat   d) Moderately   e) Extremely

8) I feel in control, coping well, on top of things

   a) Not at all   b) A little bit   c) Somewhat   d) Moderately   e) Extremely

9) I feel joyful, glad, happy

   a) Not at all   b) A little bit   c) Somewhat   d) Moderately   e) Extremely

10) Did anything disturb your sleep last night?   YES   NO (circle one)

   Please describe: ____

   ____

DAY 4 – Evening

For each of the emotions listed below, please tell us how much you have felt that emotion this evening.

Not at all	A little bit	Moderately	Quite a bit	Extremely
0	1	2	3	4

___ 1. What is the most **amused, fun-loving**, or **silly** you felt?

___ 2. What is the most **angry, irritated**, or **annoyed** you felt?

___ 3. What is the most **ashamed, humiliated**, or **disgraced** you felt?

___ 4. What is the most **awe, wonder,** or a**mazement** you felt?

___ 5. What is the most **bored, disinterested,** or **uninvolved** you felt?

___ 6. What is the most **contemptuous, scornful**, or **disdainful** you felt?

___ 7. What is the most **control, coping well**, or **on top of things** you felt?

___ 8. What is the most **disgust, distaste**, or **revulsion** you felt?

___ 9. What is the most **embarrassed, self-conscious**, or **blushing** you felt?

___ 10. What is the most **excited, eager**, or **enthusiastic** you felt?

___ 11. What is the most **grateful, appreciative,** or **thankful** you felt?

___ 12. What is the most **guilty, repentant**, or **blameworthy** you felt?

___ 13. What is the most **hate, distrust**, or s**uspicion** you felt?

___ 14. What is the most **hopeful, optimistic,** or **encouraged** you felt?

___ 15. What is the most **inspired, uplifted**, or **elevated** you felt?

___ 16. What is the most **interested, alert**, or **curious** you felt?

___ 17. What is the most **joyful, glad,** or **happy** you felt?

___ 18. What is the most **love, closeness**, or **trust** you felt?

___ 19. What is the most **proud, confident**, or **self-assured** you felt?

___ 20. What is the most **rejected, betrayed**, or **left-behind** you felt?

___ 21. What is the most **sad, downhearted**, or **unhappy** you felt?

___ 22. What is the most **satisfied, fulfilled**, or **content** you felt?

___ 23. What is the most **scared, fearful**, or **afraid** you felt?

___ 24. What is the most **stressed, nervous,** or **overwhelmed** you felt?

___ 25. What is the most **tired, sleepy,** or **drowsy** you felt?

___ 26. What is the most **sexual, desiring,** or **flirtatious** you felt?

___ 27. What is the most **sympathy, concern,** or **compassion** you felt?

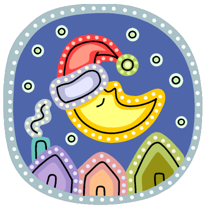

Date: ___________ (Friday)

DAY 5 – Saliva Instructions

Upon waking please avoid having beverages (tea, coffee, soda) until you have collected sample #3. All samples must be stored in the freezer after they are filled. Please let us know the true sample time, even though you forget to do it in the order we requested.

Saliva Sample # 1: Immediately when you wake up

1) What time did you collect sample # 1? ___ ___: ___ ___ am/pm (circle one)

Avoid having caffeinated beverages (coffee, tea, soda, energy drinks) before sample #3 (in 45 minutes). It is fine to eat or drink something in the next 20 minutes, but **please** avoid doing so in the 10 minutes right before taking sample #2 or sample #3.

Saliva **Sample # 2:** 30 minutes after sample # 1

2) What time did you collect sample # 2? ___ ___ : ___ ___ am/pm (circle one)

3) Did you eat, drink or brush your teeth before collecting sample # 2? YES NO (circle one)

   a. If so, what: ___________________________

Saliva **Sample # 3**: 15 minutes after sample # 2

4) What time did you collect sample # 3? ___ ___: ___ ___ am/pm (circle one)

5) Did you eat, drink or brush your teeth before collecting sample # 2? YES NO (circle one)

   a. If so, what: ___________________________

After collecting sample 3 you are allowed to brush your teeth and have beverages of your choice. You will collect sample #4 in the night before going to bed.

Saliva **Sample # 4:** before going to bed

6) What time did you collect sample # 4? ___ ___: ___ ___ am/pm (circle one)

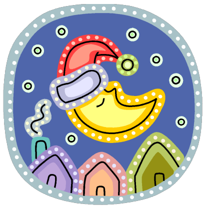

DAY 5– Awakening

1) What time did you try to go to sleep last night (lights out)? ___ ___ : ___ ___ am / pm (circle one)

2) What time did you wake up today? ___ ___ : ___ ___ am / pm (circle one)

3) How would you rate the quality of your sleep last night?

a) Very bad b) Fairly bad c) Fairly good d) Very good

4) Please indicate by marking “X” on the line below to what extent you are looking forward to versus dreading today’s events.

____

Really dreading today’s events	*Neutral*	*Really looking forward to today’s events*

5) Please indicate by marking “X” on the line below to what extent you are worrying about how things are going to go today.

____

At ease	Neutral	Worrying a lot

6) Please indicate by marking “X” on the line below to what extent you feel the events of your upcoming day are predictable (you know exactly what's going to happen today).

____

Completely<br>Completely<br>Predictable	unpredictable

7) I feel stressed, anxious, overwhelmed

a) Not at all b) A little bit c) Somewhat d) Moderately e) Extremely

8) I feel in control, coping well, on top of things

a) Not at all b) A little bit c) Somewhat d) Moderately e) Extremely

9) I feel joyful, glad, happy

a) Not at all b) A little bit c) Somewhat d) Moderately e) Extremely

10) Did anything disturb your sleep last night? YES NO (circle one)

Please describe: ____

____

11) Did you collect all samples this morning? YES NO (circle one)

(why)________________________

12) Did you have any symptoms of a cold in the past week? YES NO (circle one)

DAY 5 – Evening

For each of the emotions listed below, please tell us how much you have felt that emotion this evening.

Not at all	A little bit	Moderately	Quite a bit	Extremely
0	1	2	3	4

___ 1. What is the most **amused, fun-loving**, or **silly** you felt?

___ 2. What is the most **angry, irritated**, or **annoyed** you felt?

___ 3. What is the most **ashamed, humiliated**, or **disgraced** you felt?

___ 4. What is the most **awe, wonder,** or a**mazement** you felt?

___ 5. What is the most **bored, disinterested,** or **uninvolved** you felt?

___ 6. What is the most **contemptuous, scornful**, or **disdainful** you felt?

___ 7. What is the most **control, coping well**, or **on top of things** you felt?

___ 8. What is the most **disgust, distaste**, or **revulsion** you felt?

___ 9. What is the most **embarrassed, self-conscious**, or **blushing** you felt?

___ 10. What is the most **excited, eager**, or **enthusiastic** you felt?

___ 11. What is the most **grateful, appreciative,** or **thankful** you felt?

___ 12. What is the most **guilty, repentant**, or **blameworthy** you felt?

___ 13. What is the most **hate, distrust**, or s**uspicion** you felt?

___ 14. What is the most **hopeful, optimistic,** or **encouraged** you felt?

___ 15. What is the most **inspired, uplifted**, or **elevated** you felt?

___ 16. What is the most **interested, alert**, or **curious** you felt?

___ 17. What is the most **joyful, glad,** or **happy** you felt?

___ 18. What is the most **love, closeness**, or **trust** you felt?

___ 19. What is the most **proud, confident**, or **self-assured** you felt?

___ 20. What is the most **rejected, betrayed**, or **left-behind** you felt?

___ 21. What is the most **sad, downhearted**, or **unhappy** you felt?

___ 22. What is the most **satisfied, fulfilled**, or **content** you felt?

___ 23. What is the most **scared, fearful**, or **afraid** you felt?

___ 24. What is the most **stressed, nervous,** or **overwhelmed** you felt?

___ 25. What is the most **tired, sleepy,** or **drowsy** you felt?

___ 26. What is the most **sexual, desiring,** or **flirtatious** you felt?

___ 27. What is the most **sympathy, concern,** or **compassion** you felt?

INSTRUCTIONS FOR RETURN SHIPMENT

- Please make sure all saliva samples are **frozen** prior to shipping.
- Please ensure that **all tube caps are tightly secured**.
- If you were unable to collect all 12 saliva samples, please only send us those you did collect.
- All saliva samples should be in the **Ziploc bags** provided.
- Securely seal the Ziploc bags and place it inside the **prepaid box** provided.
- Please also include **this completed study logbook, pen and timer** in the same shipment, outside of the Ziploc bags.
- Please do not add anything else to the box, so it will meet the weight requirements for shipping.
- If you have any **receipts** related to your travels for this study, please place them in the "Receipts" envelope provided, and add this to the prepaid box.
- Seal the **Prepaid USPS Priority Mail Express Box** and bring it to the Post Office in your local area for delivery back to us.
- Once we receive your complete package, your compensation will be deposited on to your Paycard. You will be notified when the money is deposited.
- Please remember to sign the MiSBIE Pay Card Receipt form.

If you have ANY questions at all, please call:

646-774-8931 or email ck3107@cumc.columbia.edu

THANK YOU FOR YOUR TIME AND HELP!

– The MiSBIE team
