# Supplementary material for: A platform to map the mind–mitochondria connection and the hallmarks of psychobiology: the MiSBIE study
Source: Trends Endocrinol Metab. Author manuscript; Available in PMC 2024 Nov 12. (PMC11555495; doi:10.1016/j.tem.2024.08.006)
Supplement: MMC12 — Appendix 1. MiSBIE collaborators. [file NIHMS2028739-supplement-MMC12.pdf]

Appendix 1

MiSBIE Study Group Collaborators

John Beard[1,2,3], Daniel W. Belsky[1,2,4], Lauren Chu[5], Alan A. Cohen[1,4,6], Bruce H. Cohen[7,8], Steve Cole[9], Marni Falk[10,11], Lisa Feldman Barrett[12,13], Jack Devine[14], Luigi Ferrucci[15], Linda P Fried[1,4], Masashi Fujita[16], Rachel Haahr[14], Julie B Herbstman[1,6], Amel Karaa[17], Brett Kaufman[18], Christopher P. Kempes[19], Richard Kibbey[20], Iris-Tatjana Kolassa[21], Samantha Leonard[14], Jue Lin[22], Molei Liu[1,23], Nour Makarem[1,2], Daniel Malinsky[1,23], Jennifer J. Manly[24], Anna Marsland[25], Judyann K McNamara[26], Robert K Naviaux[27], Sen Pei[1,6], Amanda Peng[14], Sarah D. Pressman[28], Rebecca Reed[29], Elizabeth Reynolds[30], Carmen Sandi[31], Suzanne Segerstrom[32], Idan Shalev[5], Evan Shaulson[14], Orian Shirihai[33], Jordan Theriault[12,13], Jacob Thomas[14], Johan Van Hove[34], Lukas Van Oudenhove[35], Jennifer Wang[14], Ying Wei[1,23], Phillip Yeske[36], Temmie Yu[14], Ya Zhang[16]

[1] Columbia Science of Health Group, Robert N Butler Columbia Aging Center, Columbia University Mailman School of Public Health, New York, NY, USA

[2] Department of Epidemiology, Columbia University Mailman School of Public Health, New York, NY, USA

[3] Department of Health Policy and Management, Columbia University Mailman School of Public Health, New York, NY, USA

[4] Robert N. Butler Columbia Aging Center, Mailman School of Public Health, Columbia University, New York, NY, USA

[5] Department of Biobehavioral Health, The Pennsylvania State University, University Park, PA, USA

[6] Department of Environmental Health Sciences, Mailman School of Public Health, Columbia University, New York, NY, USA

[7] Department of Pediatrics and the Rebecca D. Considine Research Institute, Akron Children's Hospital, Akron, OH, USA

[8] Department of Pediatrics and Department of Integrative Medical Sciences, Northeast Ohio Medical University, Rootstown, OH, USA

[9] UCLA School of Medicine, Department of Psychiatry & Biobehavioral Sciences, Los Angeles, CA, USA

[10] Mitochondrial Medicine Frontier Program, Division of Human Genetics, Department of Pediatrics, The Children's Hospital of Philadelphia, Philadelphia, PA, USA

[11] Department of Pediatrics, University of Pennsylvania Perelman School of Medicine, Philadelphia, PA, USA

[12] Department of Psychology, Northeastern University, Boston, MA, USA

[13] Department of Psychiatry and the Martinos Center for Biomedical Imaging, Massachusetts General Hospital, Charlestown, MA, USA

[14] Mitochondrial Psychobiology Group, Division of Behavioral Medicine, Department of Psychiatry, Columbia University Irving Medical Center, New York, NY, USA

[15] Translational Gerontology Branch, Intramural Research Program, National Institute on Aging, National Institutes of Health, 251 Bayview Boulevard, 21224, Baltimore, MD, USA

[16] Center for Translational & Computational Neuroimmunology, Department of Neurology, Columbia University Irving Medical Center and the Taub Institute for Research on Alzheimer's Disease and the Aging Brain, New York, NY, USA

[17] Department of Pediatrics, Genetics Unit, Massachusetts General Hospital, Boston, MA, USA

[18] Department of Medicine, Division of Cardiology, Center for Metabolism and Mitochondrial Medicine, University of Pittsburgh School of Medicine, Pittsburgh, PA, USA

[19] Santa Fe Institute, Santa Fe, NM, USA

[20] Department of Internal Medicine, Department of Cellular & Molecular Physiology, Yale University School of Medicine, New Haven, CT, USA

[21] Clinical & Biological Psychology, Institute of Psychology and Education, Ulm University, Ulm, Germany

[22] Department of Biochemistry and Biophysics, University of California San Francisco, San Francisco, CA, USA

[23] Department of Biostatistics, Columbia Mailman School of Public Health, New York, NY, USA

[24] Taub Institute for Research on Alzheimer's Disease and the Aging Brain, Department of Neurology, Columbia University Irving Medical Center, New York, NY, USA

[25] Department of Psychology, University of Pittsburgh, Pittsburgh, PA, USA

[26] Montreal Institute of Classical Homeopathy, Montreal, Canada

[27] The Mitochondrial and Metabolic Disease Center, University of California, San Diego School of Medicine, San Diego, CA, USA

[28] Department of Psychological Science, University of California, Irvine, CA, USA

[29] Department of Psychology, University of Pittsburgh, Pittsburgh, PA, USA

[30] The Champ Foundation, Durham, NC, USA

[31] Laboratory of Behavioral Genetics, Brain Mind Institute, Ecole Polytechnique Fédérale de Lausanne, Lausanne, Switzerland

[32] School of Human Development and Family Sciences, College of Health, Oregon State University, Corvallis, OR, USA

[33] UCLA Metabolism Theme, Department of Medicine, Endocrinology, and Department of Molecular and Medical Pharmacology, David Geffen School of Medicine, University of California, Los Angeles, CA, USA

[34] Department of Pediatrics, Section of Clinical Genetics and Metabolism, University of Colorado, Aurora, CO, USA

[35] Laboratory for Brain-Gut Axis Studies, Translational Research in Gastrointestinal Disorders, Department of Chronic Diseases and Metabolism, KU Leuven, Leuven, Belgium

[36] United Mitochondrial Disease Foundation, Pittsburgh, PA, USA
