# Supplementary material for: A platform to map the mind–mitochondria connection and the hallmarks of psychobiology: the MiSBIE study
Source: Trends Endocrinol Metab. Author manuscript; Available in PMC 2024 Nov 12. (PMC11555495; doi:10.1016/j.tem.2024.08.006)
Supplement: MMC6 — File S6. Data dictionary v1.0. [file NIHMS2028739-supplement-MMC6.pdf]

Supplemental File 6

Data Dictionary

The **Mi**tochondrial **S**tress, **B**rain **I**maging, and **E**pigenetics Study

Version: September 2024

Table of Contents

1. Study Variables (excluding questionnaires) 5
1.1 Day 0 5
1.1.1 Screening 5
1.1.2 Eligibility 14
1.1.3 Genetic Counseling 16
1.1.4 Subject ID 17
1.1.5 Enrollment Form 18
1.1.6 Prescreening Log 18
1.1.7 Genetic Testing 19
1.1.8 Participant Information 21
1.1.9 Day 0 – Participant Checkup 21
1.1.10 Breakfast and Lunch Choices 23
1.2 Day 1 24
1.2.1 Digital Consent 24
1.2.2 Day 1 Data Collection Form 25
1.2.3 Medical Assessment 46
1.2.3.1 General Medical and Neurological Assessment 46
1.2.3.2 Newcastle Mitochondrial Disease Adult Scale 58
1.2.3.3 Newcastle Mitochondrial Disease Adult Scale Scoring 64
1.2.3.4 Case Report Form – North American Mitochondrial Disease Consortium 65
1.2.3.5 Clinical Frailty Scale 120
1.2.3.6 Karnofsky Performance Scale 121
1.2.4 Affect Ratings 122
1.2.5 Day 1 Compliance 123
1.3. Day 2 124
1.3.1 Data Collection Form Day 2 124
1.3.2 Day 2 Compliance 133
1.3.3 Neuropsychological Grading 134
1.3.4 Physiological Session 141
1.4 Biospecimen Analysis 155
1.4.1 Seahorse Data 155
1.4.2 Elisa Run 161
1.5 Biospecimen Results 168
1.5.1 CALM Lab 168
1.5.2 Saliva Steroid Hormone 172
1.5.3 Hair Steroid Hormone 182
1.5.4 Cfdna 183
1.5.5 Catecholamines 238

1.6 Psychophysiological Results 242
1.6.1 Spectral 242
1.6.1 Skin Conductance Response 432

2. Questionnaire Variables 458

2.1 Day 0 458
2.1.1 Day 0 Hotel 458
2.1.1.1 SF-36 SRH Question 458
2.1.1.2 Modified Differential Emotions Scale 458
2.1.1.3 My Daily Stress 458

2.2 Day 1 464
2.2.1 Post Breakfast Questionnaire 464
2.2.1.1 Participant Information 464
2.2.1.2 Physical Activity 464
2.2.1.3 Fat and Sugar 464
2.2.1.4 Food Frequency 464
2.2.1.5 Vitamins and Supplements 464
2.2.2 Primary Appraisal Secondary Appraisal 504
2.2.2.1 PASA 504
2.2.3 Recovery 505
2.2.3.1 Fatigue 505
2.2.3.2 Autonomic Symptoms 505
2.2.4 Day 1 Hotel 537
2.2.4.1 Modified Differential Emotions Scale 537
2.2.4.2 My Daily Stress 537
2.2.4.3 Perceived Age 537
2.2.4.4 Perceived Social Status 537
2.2.4.5 Morningness 537
2.2.4.6 Sleep 537
2.2.4.7 Fatigue 537
2.2.4.8 Social Support 537
2.2.4.9 Lifetime Stressor Exposure 537

2.3 Day 2 573
2.3.1 Part 1 of Questionnaire Package 573
2.3.1.1 Personal Well-Being 574
2.3.1.2 Couples Satisfaction 574
2.3.1.3 Sense of Coherence 574
2.3.1.4 Personality 574
2.3.1.5 Gender Role 574
2.3.1.6 Memory Complaints 574
2.3.1.7 Perceived Social Support 574

2.3.2 Part 2 of Questionnaire Package 607
2.3.2.1 Life Events 608
2.3.2.2 Perceived Stress 608
2.3.2.3 Chronic Stress 608
2.3.2.4 Daily Hassles 608
2.3.2.5 Anxiety 608
2.3.2.6 Loneliness 608
2.3.2.7 Depressive Symptoms 608
2.3.2.8 Burnout 608
2.3.2.9 PTSD 608
2.3.3 Part 3 of Questionnaire Package 641
2.3.3.1 Mood Symptoms 641
2.3.3.2 Childhood Trauma 641

2.4 Questionnaire Scoring 665
2.4.1 Questionnaire Scoring 665

2.5 Home 698
2.5.1 Home Logbook 698
2.4.1.1 Modified Differential Emotions Scale 698
2.4.1.2 My Daily Stress 698

1. Study Variables (excluding questionnaires)

1.1 Day 0

1.1.1 Screening

RedCAP Form Name: Screen

Description: Questions to ascertain inclusion and exclusion criteria.

Variable / Field Name	Section Header	Field Type	Field Label	Choices, Calculations, OR Slider Labels
**screen_id**		text	Screen ID	
**screen_date**	"Thank you for your interest in our study. I can tell you a little bit about the goal of the study, and then I'll ask you some screening questions just to make sure you're completely eligible for the study. Does that sound good?<br>The MiSBIE study is looking at how the mind and body interact with each other. We think that how we feel and what we experience affect our body, and we're interested in finding out why. We're particularly interested in the mitochondria, which is the part of the cell that makes the energy that keeps us alive.<br>(This research study is directed by Drs. Picard and Hirano- who are experts in mitochondrial medicine and neuroscience at	text	Screen Date:	

	Columbia University. This project is also conducted in collaboration with Dr. De Vivo, whom you may know from previous studies.)<br><br>At this point, do you have any questions?"			
**screen_sex**		radio	Sex	1, male | 0, female
**screen_hear_study**		dropdown	1. How did you hear about this study?	1, Clinical patients from Neuromuscular clinic (Dr. Hirano) or private office | 2, Natural History Study (Kris Engelstad) | 3, North American Mitochondrial Disease Consortium (NAMDC) (Dr. Xiomara Rosales) | 4, Dr. Sloan's Registry | 5, Flyer | 6, Other: text box
**screen_hear_study_other**		text	If other, specify:	
**screen_age**		text	2. What is your age?	
**screen_race**		checkbox	3. What is your race/ethnicity? (Choose all that apply)	1, American Indian or Alaska Native | 2, Asian | 3, Black or African American | 4, Hispanic or Latino | 5, Native Hawaiian or Other Pacific Islander | 6, White
**screen_read_level**		radio	4. What level would you consider your reading ability?	1, K-8th Grade | 2, 9th-12th Grade | 3, College Level or Above
**screen_8th_read_level**		yes/no	Do you think you would be able to read and understand a detailed consent form and instructions?	
**screen_mito_disease**		yes/no	5. Have you been diagnosed with mitochondrial disease or could you be a mutation carrier?	
**screen_mito_dis_type**		radio	6. If so, which one?	1, The mitochondrial DNA 3243 A>G mutation or MELAS. This is also known as the m.3243A>G or the A3243G mtDNA mutation. | 2, CPEO (chronic

				progressive external ophthalmalgia) or KSS (Kearns Sayre syndrome) caused by single large scale mtDNA deletion. | 3, Other?
**screen_mito_dis_other**		text	If other, please specify:	
**screen_other_trial**		yes/no	7. Are you currently involved in an experimental drug study, or a trial for exercise?	
**screen_cancer**		yes/no	8. Have you been diagnosed with cancer (Neoplastic disease)?	
**screen_cancer_remission**		yes/no	a. Are you in remission?	
**screen_raynaud**		yes/no	9.Do you have Raynaud's syndrome?	
**screen_steroid**		yes/no	10. Do you take any steroid medication (i.e. oral dexamethasone, prednisone)?	
**screen_medical**		yes/no	11. Have you been diagnosed with any other medical condition?	
**screen_medical_details**		notes	Medical conditions:	
**screen_mri**		yes/no	12. Have you ever had a MRI - magnetic resonance imaging?	
**screen_claustrophobic**		yes/no	13. Do you feel uncomfortable being in closed spaces after a certain period of time?	
**screen_metal**		yes/no	14. Do you have any metal implants in your body, such as a pacemaker, metal clips, or wires?	

**screen_tatoo**		yes/no	15. Do you have any tattoos?	
**screen_tatoo_dtls**		yes/no	If yes: Size, placement, and concentration of ink?	
**screen_braintumor**		yes/no	16. Have you been diagnosed with a brain tumor or other brain lesions?	
**screen_pregnant**		yes/no	17. Are you or could you be pregnant?	
**screen_iud**		yes/no	18. Do you have an intrauterine device (IUD)?	
**screen_iud_yes**		notes	Please provide the kind of IUD you have including the make/model.	
**screen_stroke**		yes/no	19. Have you ever had a stroke or seizure?	
**screen_blood**		yes/no	20. Have you ever fainted or become nauseous as a result of a blood draw?	
**screen_saliva**		yes/no	21. Are you willing to provide saliva samples?	
**screen_smoke**		yes/no	23. Do you smoke cigarettes?	
**screen_activity**		radio	22. During the past month, what kinds of physical activity did you do? Any exercise or strenuous chores? This doesn	1, I did not do much physical activity. I mostly did things like watching television, reading, playing cards, or playing computer games. Only occasionally, no more than once or twice a month, did I do anything more active such as going for a walk or playing tennis. | 2, Once or twice a week, I did light activities such as getting outdoors on the weekends for an easy walk or stroll. Or once or twice a week, I did chores around

				the house such as sweeping floors or vacuuming. | 3, About three times a week, I did moderate activities such as brisk walking, swimming, or riding a bike for about 15-20 minutes each time. Or about once a week, I did moderately difficult chores such as raking or mowing the lawn for about 45-60 minutes. Or about once a week, I played sports such as softball, basketball, or soccer for about 45-60 minutes. | 4, Almost daily, that is five or more times a week, I did moderate activities such as brisk walking, swimming, or riding a bike for 30 minutes or more each time. Or about once a week, I did moderately difficult chores or played sports for 2 hours or more. | 5, About three times a week, I did vigorous activities such as running or riding hard on a bike for 30 minutes or more each time. | 6, Almost daily, that is five or more times a week, I did vigorous activities such as running or riding hard on a bike for 30 minutes or more each time.
**screen_activity_notes**		notes	Notes:	
**screen_memory**		yes/no	23. Do you have problems thinking or with your memory?	
**screen_communicate**		yes/no	Does it impair your ability to communicate?	
**screen_driving**		yes/no	Your activities of daily living like walking, driving, grocery shopping, or making it to appointments?	
**telcog_1**	For this next part, I'm going to ask you some questions as part of a standard memory questionnaire. It should take about 5 minutes. Some of the questions might seem a little weird, but they all are trying to understand different parts	notes	"Please tell me your full name?"	

	of your memory. It's ok if you can't answer everything.<br>Are you in a place where there's not a lot of distractions? If you are, could you move somewhere quieter? If there are calendars or anything that might tell you the date around you, could you put those away?<br>Alright, now I'll ask you the questions.			
**telcog_1_points**		dropdown	Points	0,0 | 1,1 | 2,2
**telcog_2**		notes	"What is today's date?"	
**telcog_2_points**		dropdown	Points	0,0 | 1,1 | 2,2 | 3,3 | 4,4 | 5,5
**telcog_3**		notes	"Where are you right now?"	
**telcog_3_points**		dropdown	Points	0,0 | 1,1 | 2,2 | 3,3 | 4,4 | 5,5
**telcog_4**		notes	"Count backwards from 20 to 1?"	
**telcog_4_points**		dropdown	Points	0,0 | 1,1 | 2,2
**telcog_5**		notes	"I'm going to read you a list of ten words. Please listen carefully and try to remember them. When I am done, tell me as many words as you can, in any order. Ready? The words are: cabin, pipe, elephant, chest, silk, theatre, watch, whip,	

			pillow, giant. Now tell me all the words you can remember."	
**telcog_5_points**		dropdown	Points	0,0 | 1,1 | 2,2 | 3,3 | 4,4 | 5,5 | 6,6 | 7,7 | 8,8 | 9,9 | 10,10
**telcog_6**		notes	"One hundred minus 7 equals what?" "And 7 from that?", etc.	
**telcog_6_points**		dropdown	Points	0,0 | 1,1 | 2,2 | 3,3 | 4,4 | 5,5
**telcog_7**		notes	"What do people usually use to cut paper?"	
**telcog_7_points**		dropdown	Points	0,0 | 1,1
**telcog_8**		notes	"How many things are in a dozen?"	
**telcog_8_points**		dropdown	Points	0,0 | 1,1
**telcog_9**		notes	"What do you call the prickly green plant that live in the desert?"	
**telcog_9_points**		dropdown	Points	0,0 | 1,1
**telcog_10**		notes	"What animal does wool come from?"	
**telcog_10_points**		dropdown	Points	0,0 | 1,1
**telcog_11**		notes	Say this: 'No ifs, ands or buts'<br>Say this: 'Methodist episcopal'.	
**telcog_11_points**		dropdown	Points	0,0 | 1,1 | 2,2

**telcog_12**		notes	"Who is the President of the United States right now?"<br>"Who is the Vice-President?"	
**telcog_12_points**		dropdown	Points	0,0 | 1,1 | 2,2
**telcog_13**		notes	"With your finger, tap 5 times on the path of the phone you speak into."	
**telcog_13_points**		dropdown	Points	0,0 | 1,1 | 2,2
**telcog_14**		notes	"I'm going to give you a word and I want you to give me its opposite. For example, the opposite of hot is cold. What is the opposite of "west?"<br>What is the opposite of generous?	
**telcog_14_points**		dropdown	Points	0, 0 | 1, 1 | 2, 2
**telcog_score**		calc	Score	[telcog_1_points] + [telcog_2_points] + [telcog_3_points] + [telcog_4_points] + [telcog_5_points] + [telcog_6_points] + [telcog_7_points] + [telcog_8_points] + [telcog_9_points] + [telcog_10_points] + [telcog_11_points] + [telcog_12_points] + [telcog_13_points] + [telcog_14_points]
**screen_iv**		yes/no	24. The study involves an intravenous (IV) cathether. This is a little piece of flexible plastic inserted into a vein in your arm, which is used to collect blood at different times instead of inserting a needle every time. Are you OK with this?	
**screen_mri_still**		yes/no	25. Are you ok with staying still for an extended period of time? The	

			MRI involves laying down for an hour and a half, and we do a session on the first day that involves sitting for a few hours while we collect different measurements.	
**screen_travel**		yes/no	Would you be willing to visit Columbia University Medical Center for two consecutive days in the next year to participate in the study? ForIf you come from outside the city, your participation in the MiSBIE Study transportation and lodging will be paid for by our Team.	
**scrn_flu**		yes/no	27. Are you currently experiencing flu symptoms?	
**screen_covid**		yes/no	28. Have you ever gotten tested for COVID-19?	
**screen_covid_pos**		yes/no	a. Was this test positive?	
**screen_covid_symp**		yes/no	29. Did you have symptoms that made it likely you had the coronavirus?	
**screen_cough**		yes/no	30. Do you have a cough, shortness of breath, or a sore throat?	
**screen_travel_outside**		yes/no	31. Have you traveled outside of the tristate area in the past 14 days?	
**screen_covid_contact**		yes/no	32. Have you come into contact with anyone with a known or suspected case of Coronavirus in the past 14 days?	

**screen_flu**		yes/no	28. Are you currently experiencing flu symptoms?	
**screen_upload**		file	Additional Screening Notes	
**screen_review_sig**		file	Study coordinator review of all screening items for completion?	

1.1.2 Eligibility

RedCAP Form Name: Eligibility

Description: Validating inclusion and exclusion criteria for each screening.

Variable / Field Name	Section Header	Field Type	Field Label
**eligible_age**	Inclusion:	yes/no	1. Men and women patients between 18 and 60 years of age
**eligible_saliva**		yes/no	2. Willing to provide saliva samples and have venous catheter installed for blood collection during the outpatient visit
**eligible_consent**		yes/no	3. Willing to provide informed consent and capacity to consent
**eligible_bc**		yes/no	4. Use of effective method of birth control for women of childbearing capacity
**eligible_mtdna**		yes/no	5. Harbours a mtDNA mutation. Either the m.3243A>G point mutation, or a single large scale mtDNA deletion.
**eligible_english**		yes/no	6. English Speaking
**eligble_cog**	Exclusion:	yes/no	1. Patients with cognitive deficit incapable of providing informed consent will not be included
**eligble_neoplastic**		yes/no	2. Neoplastic disease
**eligble_flu**		yes/no	3. Symptoms of flu or other seasonal infection four weeks preceding hospital visit, as this would influence immune system parameters

**eligble_stroke**		yes/no	4. Strokes &seizures.
**eligble_mitoenceph**		yes/no	Patients who have converted to mitochondrial encephalomyopathy with lactic acidosis and stroke-like episodes (MELAS), the most severe form of the m.3243A>G disease.
**eligible_raynaud**		yes/no	5. Raynaud's syndrome (Rayneau phenomenon)
**eligible_othertrail**		yes/no	6. Involvement in any therapeutic trials listed on clinicaltrials.gov, including exercise
**eligible_steroid**		yes/no	7. Clinical use of steroid therapy, which would impact the HPA-axis and other physiological systems (e.g., oral dexamethasone, prednisone, or similar)
**eligible_mri**		yes/no	8. Metal inside or outside the body or claustrophobia prohibitive to MRI testing
**screen_eligible**	Eligibility	yes/no	Is the participant eligible
**not_eligible_text**		descriptive	IF THE INDIVIDUAL DOES NOT FIT THE INCLUSION CRITERIA:<br>"Thank you for answering these questions, and for your interest. Based on some of your answers and the strict inclusion criteria, you are ineligible to participate in this study. However, it is possible that this research will be expanded in the future, in which case you may be eligible.
**other_study**		yes/no	Would you agree to have us contact you in the future for other studies that you may be eligible to participate in?"
**eligible_text**		descriptive	IF THE INDIVIDUAL MATCHES THE INCLUSION CRITERIA:<br>"Thank you for answering these questions. You fit the inclusion criteria for this study and we would like to invite you to participate. In addition to the information I will give you here, you will be provided with what is called an "Informed Consent Form" with detailed information about all the parts of the study. You will be able to carefully read this document and discuss it with anyone, before deciding if you want to participate.
**eligible_yes**		yes/no	Would you like me to tell you about the study? This will take about 5-10 minutes.
**study_mail**		yes/no	If no: Would you like us to mail you a copy of the study brochure with some information in case you may want to participate?]

**eligible_about_study**		descriptive	[If yes, proceed with the text below:]<br>This study takes place over two full days on the Columbia University Medical Center campus. If you are traveling to the city either by driving, flying or taking public transportation, you will arrive to your pre-booked hotel the day before the commencement of the study. Transportation costs are covered by the study. If you have symptoms of flu/cold or a seasonal infection, we should wait four weeks before your visit since this could influence certain measurements.<br>Starting at midnight the night before Day 1 until you come in for your fasting blood draw, you will be asked not to eat or drink. Then on Day 1, you will arrive by taxi at 9am to the New York Presbyterian Hospital for a full day where different measures would be taken including heart rate and blood pressure, blood, saliva, a cheek swab, and small clip of hair. Also, one of the measurements involves putting your hand in a cold-water bath for 90 seconds. This may be uncomfortable, but it will not injure you. You will get breakfast and lunch at no cost to you. Before you leave, we will give you a container with instructions to collect urine overnight to measure certain hormones and metabolites so we can understand more about the link between the mind and the hormones that the body produces.<br>On the next day, you will come back, again before having breakfast, at 9am. The morning will involve answering some questionnaires, and then in the afternoon we
**study_about**		yes/no	I know this is a lot of information. Do you have any questions?
**study_brochure**		yes/no	Would you like us to send you a brochure about the study via email or regular mail?<br>If you have any questions, or if you decide to participate, please call Marissa Cross the study coordinator at 646-774-8930, or send an email to misbie.study@gmail.com

1.1.3 Genetic Counseling

RedCAP Form Name: Genetic Counseling Notes

Description: Validating inclusion and exclusion criteria for each screening.

Variable / Field Name	Field Type	Field Label
**gc_notes**	descriptive	MISBE STUDY (IRB 7424)-Genetic Counseling Note<br>PI: Dr. Martin Picard<br>Study Subject Name{gc_name}<br>[gc_name] is a {gc_age} year old {gc_sex}who presents on {gc_date} to obtain genetic testing to determine if he/she (circle one) carries the m.3243A>G mutation.

		Participation in the MISBE study (IRB 7424) with Dr. Martin Picard requires that the subject carry either the m.3243A>G mutation or a single deletion in the mitochondrial DNA. The following evidence suggests that [gc_name]is a potential carrier of the m.3243A>G mitochondrial mutation.<br>{gc_notes_1}<br>I reviewed mitochondrial genetics including: the nature of the mitochondrial genome, maternal inheritance, heteroplasmy, and possible clinical syndromes associated with the m.3243A>G mutation and the emotional significance of a positive or negative test.<br>Additional discussion included: {gc_notes_2}<br>The patient consented to having genetic testing for the presence of the m.3243A>G mutation performed. No data regarding heteroplasmy level will be provided.<br>The results will be placed in the patient's research record, but will not be placed in the patient's electronic medical record.<br>GINA was discussed. Consent for genetic testing was obtained.<br>Signed,<br>{cg_sig}
**gc_age**	text	age
**gc_sex**	dropdown	sex
**gc_sex_2**	dropdown	sex
**gc_notes_1**	notes	notes
**gc_notes_2**	notes	notes

1.1.4 Subject ID

RedCAP Form Name: Add Subject ID

Description: Subject screening ID was stored to ensure proper screening procedures were followed.

Variable / Field Name	Field Type	Field Label
**subject_id**	text	Subject ID

**screen_id**	text	Screen ID

1.1.5 Enrollment Form

RedCAP Form Name: Enrollment Form

Description: Enrollment form for IRB regulations, also kept in a regulatory binder.

Variable / Field Name	Field Type	Field Label
**record_id**	text	Record ID
**study_title**	text	Study Title
**investigator_name**	text	Investigator Name:
**site_number**	text	Site Number:
**protocol**	text	Protocol:
**date_approached**	text	Date Approached
**name_of_person_approaching**	text	Name of Person Approaching
**recruited_date**	text	Recruited Date
**if_recruited_participant_id**	text	If Recruited, Participant ID
**if_not_recruited_reason**	text	If Not Recruited, Reason

1.1.6 Prescreening Log

Form Name: Prescreening Log

Description: Prescreening Log for IRB regulations.

Variable / Field Name	Field Type	Field Label
**record_id_e5a44a**	text	Record ID
**study_title_e91585**	text	Study Title
**investigator_name_a18262**	text	Investigator Name:
**site_number_94ee54**	text	Site Number:
**protocol_0aec71**	text	Protocol:
**date_approached_23f6df**	text	Date Approached
**name_of_person_approaching_a7f5c8**	text	Name of Person Approaching
**recruited_date_0163e2**	text	Recruited Date
**if_recruited_participant_id_07f56d**	text	If Recruited, Participant ID
**if_not_recruited_reason_2adf4f**	text	If Not Recruited, Reason

1.1.7 Genetic Testing

RedCAP Form Name: Genetic Counseling Notes

Description: This form is used by the genetic counselor, Kris Englestad, and can be found in the screening project. Tracking genetic completed by potential study participants.

Variable / Field Name	Section Header	Field Type	Field Label

**gc_notes**		descriptive	MISBE STUDY (IRB 7424)-Genetic Counseling Note<br>PI: Dr. Martin Piccard<br>Study Subject Name {gc_name}<br>[gc_name] is a {gc_age} year old {gc_sex}who presents on {gc_date} to obtain genetic testing to determine i he/she (circle one) carries the m.3243A>G mutation.<br>Participation in the MISBE study (IRB 7424) with Dr. Martin Piccard requires that the subject carry either the m.3243A>G mutation or a single deletion in the mitochondrial DNA. The following evidence suggests that [gc_name]is a potential carrier of the m.3243A>G mitochondrial mutation.<br>{gc_notes_1}<br>I reviewed mitochondrial genetics including: the nature of the mitochondrial genome, maternal inheritance, heteroplasmy, and possible clinical syndromes associated with the m.3243A>G mutation and the emotional significance of a positive or negative test.<br>Additional discussion included: {gc_notes_2}<br>The patient consented to having genetic testing for the presence of the m.3243A>G mutation performed. No data regarding heteroplasmy level will be provided.<br>The results will be placed in the patient's research record, but will not be placed in the patient's electronic medical record.<br>GINA was discussed. Consent for genetic testing was obtained.<br>Signed,<br>{cg_sig}
**gc_age**		text	age
**gc_date**		text	date
**gc_sex**		dropdown	sex
**gc_sex_2**		dropdown	sex
**gc_notes_1**		notes	notes
**gc_notes_2**		notes	notes

**cg_sig**		file	signature

1.1.8 Participant Information

RedCAP Form Name: Participant Information Form

Description: Basic participant information and contact information.

Variable / Field Name	Field Type	Field Label	Variable / Field Name
**pi_gender**	radio	What is your sex?	1, Male | 2, Female | 3, Other
**pi_geneticdiagnosis**	yes/no	Genetic diagnosis, if any?	
**pi_geneticdiagnosistype**	radio	If yes:	0, Control | 1, 3243 A>G | 2, Single Deletion | 3, MELAS | 4, Other

1.1.9 Day 0 – Participant Checkup

RedCAP Form Name: Checkup Form

Description: Conducted on Day 0 or the morning of Day 1 to check in on participant's alcohol, coffee, and food intake. In addition, we ask questions to access fly or cold like symptoms in the last 4 weeks.

Variable / Field Name	Section Header	Field Type	Field Label
**checkup_script**	script: Hi [pi_name], how are you? This is the study coordinator from the MiSBIE study, and I'm calling to ask you a few questions before you come in to start the study.	yes/no	Do you have a moment?
**checkup_time**		text	What time would you be available to answer some questions? It should take 5 minutes max.
**checkup_1**	Purpose: This form will allow us to determine the participant current status before their arrival for Day 1. The participant must be contacted 5 days and asked these questions prior to their preliminary start date.	yes/no	Are you consuming more alcohol than usual?
**checkup_1_why**		notes	Why:

**checkup_2**		yes/no	Are you consuming more caffeine than usual?
**checkup_2_why**		notes	Many
**checkup_3**		yes/no	Having you been skipping meals for any reason?
**checkup_3_why**		notes	Why:
**checkup_4**		yes/no	Are you experiencing any cold like symptoms
**checkup_4_why**		notes	How long have you been experiencing those symptoms?
**checkup_5**		yes/no	Have you experienced any life changing events?
**checkup_5_why**		notes	What:
**checkup_6**		yes/no	Do you have any dietary restrictions such as gluten intolerance?
**checkup_7**		yes/no	Do you wear glasses or contact lenses?
**checkup_7_why**		descriptive	If yes: explain that they cannot wear glasses in the MRI. However, if they know their prescription, then they can be given MRI safe glasses.
**checkup_notes**		notes	Notes:
**checkup_no_issue**		descriptive	No issues: I would like to thank you for taking the time out to answer the following questions. You will be checking in at the Edge Hotel, where you will receive an envelope from the clerk at the front desk. Please remember to keep all receipts that you may incur getting to the hotel.
**checkup_cold**		descriptive	Cold/Problems: [pi_name], due to you having a cold or experiencing the following issues we would like to move your participation in the study to a later date. Can you please

			provide me with some available dates that may work for you?
**checkup_moreinfo**		descriptive	More Information: First day--the blood draw takes place on the first day, so it might be helpful if you wear something with short sleeves or that you can roll the sleeves up.<br>Please give us your food preference for the first day, and remember that you can check in to the hotel on Monday anytime after 3PM. We're looking forward to having you come in for the study.
**checkup_foodpref**		notes	Food preference:
**covid_temp_day0**		text	Morning Temperature:
**covid_cough_day0**		yes/no	Do you have a cough, shortness of breath, or a sore throat?
**covid_travel_day0**		yes/no	Have you traveled outside of the tristate area in the past 14 days?
**covid_contact_day0**		yes/no	Have you come into contact with anyone with a known or suspected case of Coronavirus in the past 14 days?

1.1.10 Breakfast and Lunch Choices

RedCAP Form Name: Breakfast and Lunch Menu

Description: This form is sent to the participant prior to their visit. Participants choose their preferred breakfast and lunch items.

Variable / Field Name	Section Header	Field Type	Field Label	Variable / Field Name
**brkfst_menu_entree**	Breakfast Menu<br>Please select from the menu below what you would like for breakfast in the morning on the two days you will be participating in this study. Please choose one entree and up to two other items.	radio	Entree	1, Cereal | 2, Bagel

**brkfst_menu_cereal**		radio	Cereal	1, Special K | 2, Granola | 3, Chex | 4, Honey Nut Cheerios
**brkfst_menu_milk**		text	Milk Preference?	
**brkfst_menu_begal**		radio	Bagel	1, Plain | 2, Cinnamon | 3, Cranberry
**brkfst_menu_creamchs**		checkbox		1, Cream Cheese | 2, Butter | 3, Jelly
**brkfst_menu_yogurt**		checkbox	Yogurt	1, Strawberry | 2, Raspberry | 3, Peach | 4, Blueberry
**brkfst_menu_fruit**		checkbox	Fruit	1, Banana | 2, Cheese and Grapes | 3, Pineapple
**lunch_menu_entree**	Lunch Menu<br>Please select from the menu below what you would like for lunch on the two days you will be participating in this study. Choose one entree and up to 3 other items.	radio	Entree	5, Spinach Salad with Egg, Bacon, and Spicy Orange Dressing (contains sesame oil) | 6, Chicken Caesar Salad | 7, Kale Salad with Lemon-Tahini Dressing (contains sesame seeds) Vegan | 8, Turkey and Swiss with Dijon Mustard Sandwich | 9, Chicken Pesto Sandwich | 10, Egg Salad Sandwich Vegetarian
**lunch_menu_yogurt**		checkbox	Yogurt	1, Strawberry | 2, Raspberry | 3, Peach | 4, Blueberry
**lunch_menu_fruit**		checkbox	Fruit	1, Banana | 2, Cheese and Grapes | 3, Pineapple
**lunch_menu_other**		checkbox	Other Snacks	1, Assorted Nuts and Dried Fruit | 2, Hummus with Pretzels | 3, Regular Salted Potato Chips

1.2 Day 1

1.2.1 Digital Consent

RedCAP Form Name: Econsent form

Description: Only used in 2020 and 2021, when in person consent was not permitted. All other consent forms are kept in a locked file cabinet.

Variable / Field Name	Section Header	Field Type	Field Label

**consent_p1**		descriptive	
**consent_p2**		descriptive	
**consent_p3**		descriptive	
**consent_agree**		checkbox	
**consent_ppt_name_3**	Martin Picard, Principal Investigator	text	Print Name:
**consent_ppt_sign_3**		file	Sign:
**consent_ppt_date_3**		text	Date:

1.2.2 Day 1 Data Collection Form

RedCAP Form Name: Data Collection Form Day 1

Description: Form for all Day 1 study procedures. Ensures all procedures are completed and all data and Day 1 details are recorded.

**Variable / Field Name**	**Section Header**	**Field Type**	**Field Label**	
**covid_temp_day1**	COVID SCREENING	text	Morning Temperature:	
**covid_cough_day1**		yes/no	Do you have a cough, shortness of breath, or a sore throat?	
**covid_travel_day1**		yes/no	Have you traveled outside of the tristate area in the past 14 days?	
**covid_contact_day1**		yes/no	Have you come into contact with anyone with a known or suspected case of Coronavirus in the past 14 days?	

**dcf_vaccination_covid**		yes/no	Have you been vaccinated for Covid-19?	
**dcf_vaccination_covid_dose**		radio	How many doses of the vaccine have you received?	1, 1 | 2, 2 | 3, 3 | 4, 4
**dcf_date_day1**	Day 1	text	Date	
**dcf_age**		calc	Age	datediff([dcf_date_day1], [pi_birthdate],'y')
**dcf_starttime_day1**		text	Time	
**dcf_weather_day1**		radio	External Weather:	1, Sunny | 2, Cloudy (but not wet) | 3, Rain/Snow
**dcf_extnl_temp_day1**		text	External Temperature:	
**dcf_ambulatory**		yes/no	Ambulatory, wheelchair	
**dcf_ambulatory_dtl**		text	If Yes, details	
**dcf_alone**		radio	During travel to hotel, participant is:	1, Alone | 2, Accompanied
**dcf_alone_dtls**		text	If accompanied, relation:	
**dcf_alone_1540**		radio	During travel to PH1540, participant is:	1, Alone | 2, Accompanied
**dcf_alone_dtls_2**		text	If accompanied, relation:	
**dcf_housing**		radio	Housing:	1, Drove this morning | 2, Hotel | 3, Other
**dcf_housing_dtls**		text	If Yes, details	1, Alone | 2, Accompanied

**dcf_hippa_1**		checkbox	Sign HIPAA Authorization Form	1, Yes
**dcf_compliance**		checkbox	Compliance Form Day 1	1, Yes
**dcf_mri_result_day1**		yes/no	MRI Results Request	
**dcf_permission**		yes/no	Permission to use samples for future studies:	
**dcf_cssrs_1**		yes/no	1. In the past month, have you wished you were dead or wished you could go to sleep and not wake up?	
**dcf_cssrs_2**		yes/no	2. In the past month, have you actually had any thoughts about killing yourself	
**dcf_cssrs_3**		yes/no	3. Have you been thinking about how you might do this?	
**dcf_cssrs_4**		yes/no	4. Have you had these thoughts and had some intention on acting on them? If yes: HIGH RISK	
**dcf_cssrs_5**		yes/no	5. Have you started to work out or worked out the details of how to kill yourself? Did you intend to carry out this plan? If yes: HIGH RISK	
**dcf_cssrs_6**		yes/no	6. Have you done anything, started to do anything, or prepared to do anything to end your life? If yes, was this	

			in the last month? If yes: HIGH RISK	
**dcf_check_hotel_1**	Anthropometric	checkbox	Check Hotel Questionnaires	1, Yes
**dcf_sys_bp1**		text	Systolic Blood Pressure 1	
**dcf_dia_bp1**		text	Diastolic Blood Pressure 1	
**dcf_hr_1**		text	Heart Rate 1	
**dcf_sys_bp2**		text	Systolic Blood Pressure 2	
**dcf_dia_bp2**		text	Diastolic Blood Pressure 2	
**dcf_hr_2**		text	Heart Rate 2	
**dcf_sys_bp3**		text	Systolic Blood Pressure 3	
**dcf_dia_bp3**		text	Diastolic Blood Pressure 3	
**dcf_hr_3**		text	Heart Rate 3	
**dcf_sys_bp_avg**		calc	Average Systolic Blood Pressure	mean([dcf_sys_bp2], [dcf_sys_bp3])
**dcf_dia_bp_avg**		calc	Average Diastolic Blood Pressure	mean([dcf_dia_bp2], [dcf_dia_bp3])
**dcf_avg_hr**		calc	Average Heart Rate	([dcf_hr_1] + [dcf_hr_2] + [dcf_hr_3])/3
**dcf_bp_hr_notes**		notes	Note	
**dcf_hgt**		text	Height	

**dcf_wgt**		text	Weight	
**dcg_adj_wgt**		calc	Adjusted Weight	if([pi_sex]='2',[dcf_wgt]-1.7,[dcf_wgt]-2.6)
**dcf_bmi**		calc	BMI	([dcg_adj_wgt]/2.2)/(([dcf_hgt]/100)*([dcf_hgt]/100))
**dcf_bodyfat1**		text	Percentage of body fat1	
**dcf_bodyfat2**		text	Percentage of body fat2	
**dcf_bodyfat_avg**		calc	Average percentage of body fat	mean([dcf_bodyfat1], [dcf_bodyfat2])
**dcf_waist**		text	Waist circumference:	
**dcf_hip**		text	Hip circumference:	
**dcf_temp_room**	Fasting blood draw	text	Temperature of room	
**dcf_lastfood**		text	When is the last time participant had food/drink other than water:	
**dcf_medication**		radio	Medication to take?	1, Yes| 2, No | 3, Taken already
**dcf_medication_dtls**		text	Time	
**dcf_affect1**		checkbox	Affect 1	1, Yes
**dcf_saliva1**		checkbox	Saliva 1	1, Yes
**dcf_temp1_dtls**		text	Temp1. H	
**dcf_t1n_dtls**		text	T1. N	

**dcf_t1t_dtls**		text	T1. T	
**dcf_catheter**		radio	Placing catheter:	1, First attempt | 2, More than one attempt
**dcf_catheter_move**		checkbox	Catheter Movement:	1, Movement Before Yellow Tubes | 2, Movement During Yellow Tubes | 3, Movement After Yellow Tubes
**dcf_catheter_dtls**		text	reason:	
**dcf_blood_clct**	Blood tubes (Blood 1)	checkbox	Collected	12, Light Blue 1 | 1, Lavendar 1 | 13, Gold 1 | 14, Gold 2 | 3, Yellow 1 | 4, Yellow 2 | 5, Yellow 3 | 6, Yellow 4 | 7, Yellow 5 | 17, Light Blue 2 | 18, Light Blue 3 | 8, Red 1 | 9, Purple 1 (CBC) | 19, Light Blue 4 | 20, Light Blue 5 | 15, Red 2 | 16, Purple 2 | 10, Green 1 | 11, Green 2
**dcf_blood_cntrfgd**		checkbox	Centrifuged	1, Lavendar 1 | 2, Lavendar 2
**dcf_tube_ice**		checkbox	Tubes on ice	1, Yes
**dcf_affect_blood**		checkbox	Affect Blood	1, Yes
**dcf_blood_draw_painful**		radio	Please rate your discomfort during the blood draw was from 0 (not painful at all) to 10 (very painful):	0, 0 not painful at all | 1, 1 | 2, 2 | 3, 3 | 4, 4 | 5, 5 | 6, 6 | 7, 7 | 8, 8 | 9, 9 | 10, 10 very painful
**dcf_bccl_swab**		checkbox	Buccal swab	1, Yes
**dcf_bccl_cute**		checkbox	Cut swab in tube	1, Yes
**dcf_brkfst_start_day1**	Breakfast	text	Breakfast start:	
**dcf_brkfst_end_day1**		text	Breakfast end:	

**dcf_brkfst_cmpl_day1**		radio	Participant:	1, Ate everything | 2, Not eaten completely
**dcf_brkfst_lo_day1**		file	Picture of leftover breakfast	
**dcf_brkfst_cmpl_dtls_day1**		text	reason:	
**dcf_caffeine**		yes/no	Caffeine Consumed?	
**dcf_demographics**	Hair collection	checkbox	Post-Breakfast Demographics Questionnaire	1, Yes
**dcf_hair**		checkbox	Two clips of hair	1, Yes
**dcf_hair_photo**		file	Take photo of strands	
**dcf_fev1_1**	Spirometry	text	FEV1 1	
**dcf_fev1_pred_perc_1**		text	FEV1 1 Predicted %	
**dcf_fvc_1**		text	FVC 1	
**dcf_fvc_pred_perc_1**		text	FVC 1 Predicted %	
**dcf_fev1fvc_1**		text	FEV1/FVC	
**dcf_fev1fvc_perc_1**		text	FEV1/FVC 1 Predicted %	
**dcf_fev1_2**		text	FEV1 2	
**dcf_fev1_pred_perc_2**		text	FEV1 2 Predicted %	
**dcf_fvc_2**		text	FVC 2	

**dcf_fvc_pred_perc_2**		text	FVC 2 Predicted %	
**dcf_fev1fvc_2**		text	FEV1/FVC	
**dcf_fev1fvc_perc_2**		text	FEV1/FVC 2 Predicted %	
**dcf_fev1_3**		text	FEV1 3	
**dcf_fev1_pred_perc_3**		text	FEV1 3 Predicted %	
**dcf_fvc_3**		text	FVC 3	
**dcf_fvc_pred_perc_3**		text	FVC 3 Predicted %	
**dcf_fev1fvc_3**		text	FEV1/FVC	
**dcf_fev1fvc_perc_3**		text	FEV1/FVC 3 Predicted %	
**dcf_medscreen**	Medical Screening	checkbox	Medical screening, 1.5 hours	1, Yes
**dcf_lunch_day1**		checkbox	Lunch	1, Yes
**dcf_catheter_attempt**	INSTRUMENTATION	radio	Placing catheter:	1, First attempt | 2, More than one attempt
**dcf_catheter_more**		text	Reasons	
**dcf_blood_draw_painful_catheter**		radio	Please rate your discomfort during the blood draw was from 0 (not painful at all) to 10 (very painful):	0, 0 not painful at all | 1, 1 | 2, 2 | 3, 3 | 4, 4 | 5, 5 | 6, 6 | 7, 7 | 8, 8 | 9, 9 | 10, 10 very painful
**dcf_affect_blood2**		checkbox	Affect Blood 2	1, Yes

**dcf_instumt**		checkbox	Instrumentation:	1, 3-lead ECG | 2, Respiration bands | 3, Finger cuff | 4, Skin conductance
**dcf_calibration_sit**		checkbox	Breathing Calibration	1, yes
**dcf_start_rest**		text	Start Time of 30-min resting period	
**dcf_start_end**		text	End Time of 30-min resting period	
**dcf_secpt_des**	Speech	descriptive		
**dcf_affect2**	5 min prior	checkbox	Affect 2	1, Yes
**dcf_blood2**		checkbox	Blood 2	1, Yes
**dcf_saliva2**		checkbox	Saliva 2	1, Yes
**dcf_temp2_dtls**		text	Temp2. H	
**dcf_t2n_dtls**		text	T2. N	
**dcf_t2t_dtls**		text	T2. T	
**dcf_speech_instruc**		checkbox	Instructions for Speech	1, Yes
**dcf_pasa**		checkbox	PASA	1, Yes
**dcf_speech_prep**		checkbox	2 min	1, Yes
**dcf_speech_**		checkbox	3 min	1, Yes
**dcf_affect3**	5 min	checkbox	Affect 3	1, Yes

**dcf_blood3**		checkbox	Blood 3	1, Yes
**dcf_saliva3**		checkbox	Saliva 3	1, Yes
**dcf_temp3_dtls**		text	Temp3. H	
**dcf_t3n_dtls**		text	T3. N	
**dcf_t3t_dtls**		text	T3. T	
**dcf_affect4**	10 min	checkbox	Affect 4	1, Yes
**dcf_blood4**		checkbox	Blood 4	1, Yes
**dcf_saliva4**		checkbox	Saliva 4	1, Yes
**dcf_temp4_dtls**		text	Temp4. H	
**dcf_t4n_dtls**		text	T4. N	
**dcf_t4t_dtls**		text	T4. T	
**dcf_affect5**	20 min	checkbox	Affect 5	1, Yes
**dcf_blood5**		checkbox	Blood 5	1, Yes
**dcf_saliva5**		checkbox	Saliva 5	1, Yes
**dcf_temp5_dtls**		text	Temp5. H	
**dcf_t5n_dtls**		text	T5. N	
**dcf_t5t_dtls**		text	T5. T	

**dcf_affect6**	30 min	checkbox	Affect 6	1, Yes
**dcf_blood6**		checkbox	Blood 6	1, Yes
**dcf_saliva6**		checkbox	Saliva 6	1, Yes
**dcf_temp6_dtls**		text	Temp6. H	
**dcf_t6n_dtls**		text	T6. N	
**dcf_t6t_dtls**		text	T6. T	
**dcf_affect7**	60 min	checkbox	Affect 7	1, Yes
**dcf_blood7**		checkbox	Blood 7	1, Yes
**dcf_saliva7**		checkbox	Saliva 7	1, Yes
**dcf_temp7_dtls**		text	Temp7. H	
**dcf_t7n_dtls**		text	T7. N	
**dcf_t7t_dtls**		text	T7. T	
**dcf_qnaire1_part1**	15 minutes of recovery questionnaires	checkbox	Questionnaire Package 1	1, Yes
**dcf_affect8**	90 min	checkbox	Affect 8	1, Yes
**dcf_blood8**		checkbox	Blood 8	1, Yes
**dcf_saliva8**		checkbox	Saliva 8	1, Yes
**dcf_temp8_dtls**		text	Temp8. H	

**dcf_t8n_dtls**		text	T8. N	
**dcf_t8t_dtls**		text	T8. T	
**dcf_qnaire1_part2**	15 minutes of recovery questionnaires	checkbox	Questionnaire Package 1	1, Yes
**dcf_demographics_90**		checkbox	Post-Breakfast Demographics Questionnaire	1, yes
**dcf_affect9**	120 min	checkbox	Affect 9	1, Yes
**dcf_blood9**		checkbox	Blood 9	1, Yes
**dcf_saliva9**		checkbox	Saliva 9	1, Yes
**dcf_temp9_dtls**		text	Temp9. H	
**dcf_t9n_dtls**		text	T9. N	
**dcf_t9t_dtls**		text	T9. T	
**dcf_cbc**		descriptive	CBC	
**dcf_cbc_60**		checkbox	60	1, Yes
**dcf_cbc_9**		checkbox	90	1, Yes
**dcf_cbc_120**		checkbox	120	1, Yes
**dcf_cbc_nc**		checkbox	Not collected	1, Yes

**dcf_iv_replace**		yes/no	Did the IV need to be replaced?	
**dcf_iv_replace_when**		radio	If yes, when was the IV replaced?	1, 30 minutes before start of baseline | -5, -5 | 2, +5 | 3, +10 | 4, +20 | 5, +30 | 6, +60 | 7, +90 | 8, +120
**dcf_deinstru_yn**		yes/no	Was the participant deinstrumented for a break at any point of the protocol?	
**dcf_deinstru_when**		radio	If yes, when were they deinstrumented?	1, after +10 | 2, after +20 | 3, after +30 | 4, after +60 | 5, after +90 | 6, after +120
**dcf_brth_hrv**	Closure	checkbox	Breathing-induced heart rate variability task	1, Yes
**dcf_sit_stand**		checkbox	Sitting recording/ Standing recording	1, Yes
**dcf_calibration_stand**		checkbox	Breathing Calibration	1, Yes
**dcf_sit_stand_time**		text	30 Sit-stand test Record number of sit-stands	
**dcf_sst_energy**		calc	Energy Expended During Sit-Stand Task	round(([dcf_wgt]*0.8125*(1/2.2046)*([dcf_hgt]/100-0.45)* [dcf_sit_stand_time]*10)/1000,2)
**dcf_5zsit_stand_time**		text	5x Sit Stand Time	
**dcf_temp_h20**	Before Cold-Pressor	text	Temperature of Water	
**dcf_bcp_r**		text	Temp 1. Hleft	
**dcf_bcp_dtls**		text	Temp 1. Right	
**dcf_t10n_dtls**		text	T1. N	

**dcf_t10t_dtls**		text	T1. T	
**dcf_cold_pressor_time**		text	Record seconds hand was submerged	
**dcf_cold_pressor**		checkbox	Cold-Pressor	1, Yes
**dcf_cold_pressor_painful**	After Cold-Pressor	radio	Please rate how painful the task was from 0 (not painful at all) to 10 (extremely painful):	0, 0 not painful at all | 1, 1 | 2, 2 | 3, 3 | 4, 4 | 5, 5 | 6, 6 | 7, 7 | 8, 8 | 9, 9 | 10, 10 extremely painful
**dcf_acp_l**		text	Temp 1. Hleft	
**dcf_acp_r**		text	Temp 1. Right	
**dcf_t11n_dtls**		text	T1. N	
**dcf_t11t_dtls**		text	T1. T	
**dcf_saliva10**	10 min after Cold Pressor onset	checkbox	Saliva 10	1, Yes
**dcf_affect10**		checkbox	Affect 10	1, Yes
**dcf_acp10_l**		text	Temp 1. Hleft	
**dcf_acp10_r**		text	Temp 1. Right	
**dcf_t12n_dtls**		text	T1. N	
**dcf_t12t_dtls**		text	T1. T	
**met_ree_d1**	METABOLIC RATE	text	Resting Energy Expenditure REE (kcal/day)	

**met_vo2_d1**		text	VO2 (mlO2/min)	
**met_feo2_d1**		text	FeO2 (%)	
**met_tidal_d1**		text	Tidal volume (L)	
**met_ve_d1**		text	Minute Ventilation VE (L/min)	
**met_vo2_kg_d1**		calc	VO2 (mLO2/min/kg)	[met_vo2_d1]/([dcf_wgt]/2.205)
**met_rr_d1**		text	Respiratory Rate RR (Breaths/min)	
**dcf_metabolic_move_day1**		yes/no	Did the participant move during this analysis?	
**dcf_metabolic_move_details_day1**		text	if yes, additional box for comments	
**dcf_metabolic_air_day1**		yes/no	Did you notice air escaping from the mouthpiece?	
**dcf_metabolic_air_notes_day1**		notes		
**dcf_deinstrumnt_day1**		checkbox	De-instrumentation	1, Yes
**dcf_cold_physio_painful**		radio	Please rate your discomfort during the physio session from 0 (not uncomfortable at all) to 10 (extremely uncomfortable):	0, 0 not uncomfortable at all | 1, 1 | 2, 2 | 3, 3 | 4, 4 | 5, 5 | 6, 6 | 7, 7 | 8, 8 | 9, 9 | 10, 10 extremely uncomfortable
**dcf_centrifuge_log**		file	Centrifuge Log	

**dcf_time_est_flag**	Verbal Estimation Task (approx. 10min)<br><br>1. The participant sits in a comfortable chair in a quiet room with the experimenter.<br><br>2. The participant is told, "We will run an exercise that looks at how you perceive time. This exercise is meant to help us understand your internal clock. There will be two different exercises, about 10 minutes each.<br><br>3. The participant must remove wristwatch and put away all devices that have a clock on it.<br><br>4. The participant is told, "For the first exercise, I will start a timer of a random period of time. When the timer ends and a sound plays, you will tell me how much time you think has passed. Please refrain from speaking during the task. This will take approximately 10 minutes altogether. Do you have any questions?"<br><br>5. When the participant is ready, the participant is told, "When I say start, tell me how much time has passed after the buzzer sounds... starting now." and initiates a timer with an amount of time indicated in the below table.<br><br>a. Note: Experimenter should not look at participant during the task.<br><br>b. Note: Experimenter should not look at the timer during the task.<br><br>c. Note: Experimenter should ensure that the participant does not see data sheet. Use a clipboard oriented towards experimenter.	yes/no	Flag time estimation data?	

	6. Once the timer is up a sound plays and the experimenter records the amount of time the participant believes has passed.<br>7. This task is repeated to fill the below table, with a 5-second pause between each task where the experimenter sets the next tasks timer and asks, "Are you ready for the next task?".			
**dcf_time_est_flag_notes**		notes	Why was this data flagged?	
**dcf_time_est_start**		text	Protocol Start Time	
**dcf_time_est_t1**	Task 1 Lets Practice (Elapsed seconds 12)	text	Estimate Time (seconds)	
**dcf_time_est_t1_note**		notes	Notes:	
**dcf_time_est_t2**	Task 2 Was that clear? (Elapsed seconds 29)	text	Estimate Time (seconds)	
**dcf_time_est_t2_note**		notes	Notes:	
**dcf_time_est_t3**	Task 3 You are doing great (Elapsed seconds 55)	text	Estimate Time (seconds)	
**dcf_time_est_t3_note**		notes	Notes:	
**dcf_time_est_t4**	Task 4 (Elapsed seconds 25)	text	Estimate Time (seconds)	
**dcf_time_est_t4_note**		notes	Notes:	
**dcf_time_est_t5**	Task 5 (Elapsed seconds 11)	text	Estimate Time (seconds)	

**dcf_time_est_t5_note**		notes	Notes:	
**dcf_time_est_t6**	Task 6 (Elapsed seconds 50)	text	Estimate Time (seconds)	
**dcf_time_est_t6_note**		notes	Notes:	
**dcf_time_est_t7**	Task 7 Half-way through (Elapsed seconds 13)	text	Estimate Time (seconds)	
**dcf_time_est_t7_note**		notes	Notes:	
**dcf_time_est_t8**	Task 7 You are doing great(Elapsed seconds 47)	text	Estimate Time (seconds)	
**dcf_time_est_t8_note**		notes	Notes:	
**dcf_time_est_t9**	Task 9 (Elapsed seconds 32)	text	Estimate Time (seconds)	
**dcf_time_est_t9_note**		notes	Notes:	
**dcf_time_est_t10**	Task 10 Three more left (Elapsed seconds 8)	text	Estimate Time (seconds)	
**dcf_time_est_t10_note**		notes	Notes:	
**dcf_time_est_t11**	Task 11(Elapsed seconds 28)	text	Estimate Time (seconds)	
**dcf_time_est_t11_note**		notes	Notes:	
**dcf_time_est_t12**	Task 12 Last One (Elapsed seconds 51)	text	Estimate Time (seconds)	
**dcf_time_est_t12_note**		notes	Notes:	

**dcf_time_est_end**		text	Protocol end Time	
**dcf_prod_task_start**	Production Task (10min)<br>1. The participant is told, "For the second exercise, I will tell you an amount of time, for example 30 seconds, and you will tell me when 30 seconds has passed. I will tell you when to start counting and simply tell me 'now' when you believe 30 seconds has passed. Please refrain from speaking during the task. These tasks will take approximately 10 minutes all together. Do you have any questions?"<br>2. When the participant is ready, the experimenter says "Tell me when X seconds have passed starting now." and initiates a timer starting at 0<br>3. When the participant believes the allotted time has passed he/she says stops and the experimenter stops the timer and records the elapsed time.<br>a. Note: Experimenter should not look at participant during the task.<br>b. Note: Experimenter should not look at the timer during the task.<br>c. Note: Experimenter should ensure that the participant does not see data sheet. Use a clipboard oriented towards experimenter.<br>d. Note: if there is a delay between the participants saying stop and the experimenter stopping of the timer, mark that down in notes.<br>4. This task is repeated to fill the below table, with a 5-second pause	text	Protocol Start Time	

	between each task, and asks, "Are you ready for the next task?"			
**dcf_prod_task_t1**	Task 1 Lets Practice (Elapsed seconds 10)	text	Estimate Time (seconds)	
**dcf_prod_task_t1_note**		notes	Notes:	
**dcf_prod_task_t2**	Task 2 Was that clear? (Elapsed seconds 10)	text	Estimate Time (seconds)	
**dcf_prod_task_t2_note**		notes	Notes:	
**dcf_prod_task_t3**	Task 3 You are doing great (Elapsed seconds 10)	text	Estimate Time (seconds)	
**dcf_prod_task_t3_note**		notes	Notes:	
**dcf_prod_task_t4**	Task 4 (Elapsed seconds 10)	text	Estimate Time (seconds)	
**dcf_prod_task_t4_note**		notes	Notes:	
**dcf_prod_task_t5**	Task 5 (Elapsed seconds 10)	text	Estimate Time (seconds)	
**dcf_prod_task_t5_note**		notes	Notes:	
**dcf_prod_task_t6**	Task 6 (Elapsed seconds 30)	text	Estimate Time (seconds)	
**dcf_prod_task_t6_note**		notes	Notes:	
**dcf_prod_task_t7**	Task 7 Half-way through (Elapsed seconds 30)	text	Estimate Time (seconds)	

**dcf_prod_task_t7_note**		notes	Notes:	
**dcf_prod_task_t8**	Task 7 You are doing great (Elapsed seconds 30)	text	Estimate Time (seconds)	
**dcf_prod_task_t8_note**		notes	Notes:	
**dcf_prod_task_t9**	Task 9 (Elapsed seconds 30)	text	Estimate Time (seconds)	
**dcf_prod_task_t9_note**		notes	Notes:	
**dcf_prod_task_t10**	Task 10 Three more left (Elapsed seconds 60)	text	Estimate Time (seconds)	
**dcf_prod_task_t10_note**		notes	Notes:	
**dcf_prod_task_t11**	Task 11(Elapsed seconds 60)	text	Estimate Time (seconds)	
**dcf_prod_task_t11_note**		notes	Notes:	
**dcf_prod_task_t12**	Task 12 Last One (Elapsed seconds 60)	text	Estimate Time (seconds)	
**dcf_prod_task_t12_note**		notes	Notes:	
**dcf_prod_task_end**		text	Protocol end Time	
**dcf_method_count**		text	That was the end of the second and final task. What method(s) did you use to count during these tasks?	
**dcf_method_change**		text	Did you change your method of counting during these tasks?	

**dcf_time_est**		checkbox	Time Estimation Task	1, Yes
**dcf_day2overview**		checkbox	Day 2 protocol overview	1, Yes
**dcf_endtime_day1**		text	Time end of day 1	

1.2.3 Medical Assessment

1.2.3.1 General Medical and Neurological Assessment

RedCAP Form Name: General Medical and Neurological Examination

Description: Completed by study physician, includes most items from the medical assessment.

Variable / Field Name	Section Header	Field Type	Field Label	Variable / Field Name
**cns_id**		text	Participant ID	
**cns_visit**		text	Study Visit	
**cns_date**		text	Examination Date	
**weight_copy**		descriptive	weight:[day1_arm_2][dcg_adj_wgt]	
**adj_wgt_perc**		calc	Adjusted Weight Percentile	if(([day1_arm_2][pi_sex]=2) && ([day1_arm_2][dcg_adj_wgt] < 100),1,if(([day1_arm_2][pi_sex]=1) && ([day1_arm_2][dcg_adj_wgt] < 125),1,0))
**height_copy**		descriptive	Height:[day1_arm_2][dcf_hgt]	
**hgt_perc**		calc	Height Percentile	if(([day1_arm_2][pi_sex]=2) && ([day1_arm_2][dcf_hgt] < 149),1,if(([day1_arm_2][pi_sex]=1) && ([day1_arm_2][dcf_hgt] < 161),1,0))
**cns_weight**		text	Weight at visit	
**cns_height**		text	Height at visit	

**cns_hc**		text	Head Circumference	
**cns_instr**	Instructions: Complete all items as accurately as possible. For medical and neurological ratings, check only one response. Comment on all items rated as Abnormal/Present.	descriptive		
**cns_eyes**	General Medical Exam	radio	Eyes	0, Abnormal/Present | 1, Normal/absent
**cns_eyes_dtls**		text	Comments:	
**cns_ears**		radio	Ears	0, Abnormal/Present | 1, Normal/absent
**cns_ears_dtls**		text	Comments:	
**cns_nasopharynx**		radio	Nasopharynx	0, Abnormal/Present | 1, Normal/absent
**cns_nasopharynx_dtls**		text	Comments:	
**cns_lymphnodes**		radio	Lymph nodes	0, Abnormal/Present | 1, Normal/absent
**cns_lymphnodes_dtls**		text	Comments:	
**cns_heart**		radio	Heart	0, Abnormal/Present | 1, Normal/absent
**cns_heart_dtls**		text	Comments:	

**cns_lungs**		radio	Lungs	0, Abnormal/Present | 1, Normal/absent
**cns_lungs_dtls**		text	Comments:	
**cns_abdomen**		radio	Abdomen	0, Abnormal/Present | 1, Normal/absent
**cns_abdomen_dtls**		text	Comments:	
**cns_skeletal**		radio	Skeletal	0, Abnormal/Present | 1, Normal/absent
**cns_skeletal_dtls**		text	Comments:	
**cns_skin**		radio	Skin	0, Abnormal/Present | 1, Normal/absent
**cns_skin_dtls**		text	Comments:	
**cns_vascular**		radio	Vascular	0, Abnormal/Present | 1, Normal/absent
**cns_vascular_dtls**		text	Comments:	
**cns_carotid_artry**		radio	Carotid artery	0, Abnormal/Present | 1, Normal/absent
**cns_carotid_artry_dtls**		text	Comments:	
**cns_temporal_artry**		radio	Temporal artery	0, Abnormal/Present | 1, Normal/absent
**cns_temporal_artry_dtls**		text	Comments:	
**cns_bruits**		radio	Bruits	0, Abnormal/Present | 1, Normal/absent
**cns_bruits_dtls**		text	Comments:	
**cns_ocular_fundi**	Funduscopic Exam	radio	Ocular Fundi	0, Abnormal/Present | 1, Normal/absent

**cns_ocular_fundi_dtls**		text	Comments:	
**cns_retinal_vssls**		radio	Retinal vessels	0, Abnormal/Present | 1, Normal/absent
**cns_retinal_vssls_dtls**		text	Comments:	
**cns_optic_ppll**		radio	Optic papilla	0, Abnormal/Present | 1, Normal/absent
**cns_optic_ppll_dtls**		text	Comments:	
**cns_macula_regn**		radio	Macula region	0, Abnormal/Present | 1, Normal/absent
**cns_macula_regn_dtls**		text	Comments:	
**cns_peripheral_rtn**		radio	Peripheral retina	0, Abnormal/Present | 1, Normal/absent
**cns_peripheral_rtn_dtls**		text	Comments:	
**cns_crnl_nrv_i**	Cranial Nerves	radio	Cranial nerve I	0, Abnormal/Present | 1, Normal/absent
**cns_crnl_nrv_i_dtls**		text	Comments:	
**cns_crnl_nrv_ii**		radio	Cranial nerve II	0, Abnormal/Present | 1, Normal/absent
**cns_crnl_nrv_ii_dtls**		text	Comments:	
**cns_crnl_nrv_iii**		radio	Cranial nerve III	0, Abnormal/Present | 1, Normal/absent
**cns_crnl_nrv_iii_dtls**		text	Comments:	
**cns_crnl_nrv_iv**		radio	Cranial nerve IV	0, Abnormal/Present | 1, Normal/absent
**cns_crnl_nrv_iv_dtls**		text	Comments:	

**cns_crnl_nrv_v**		radio	Cranial nerve V	0, Abnormal/Present | 1, Normal/absent
**cns_crnl_nrv_v_dtls**		text	Comments:	
**cns_crnl_nrv_vi**		radio	Cranial nerve VI	0, Abnormal/Present | 1, Normal/absent
**cns_crnl_nrv_vi_dtls**		text	Comments:	
**cns_crnl_nrv_vii**		radio	Cranial nerve VII	0, Abnormal/Present | 1, Normal/absent
**cns_crnl_nrv_vii_dtls**		text	Comments:	
**cns_crnl_nrv_viii**		radio	Cranial nerve VIII	0, Abnormal/Present | 1, Normal/absent
**cns_crnl_nrv_viii_dtls**		text	Comments:	
**cns_crnl_nrv_ix**		radio	Cranial nerve IX	0, Abnormal/Present | 1, Normal/absent
**cns_crnl_nrv_ix_dtls**		text	Comments:	
**cns_crnl_nrv_x**		radio	Cranial nerve X	0, Abnormal/Present | 1, Normal/absent
**cns_crnl_nrv_x_dtls**		text	Comments:	
**cns_crnl_nrv_xi**		radio	Cranial nerve XI	0, Abnormal/Present | 1, Normal/absent
**cns_crnl_nrv_xi_dtls**		text	Comments:	
**cns_crnl_nrv_xii**		radio	Cranial nerve XII	0, Abnormal/Present | 1, Normal/absent
**cns_crnl_nrv_xii_dtls**		text	Comments:	
**cns_gait_statn**	Stance and Gait	radio	Gait and station	0, Abnormal/Present | 1, Normal/absent

**cns_gait_statn_dtls**		text	Comments:	
**cns_reg_wlk_tst**		radio	Regular walking test	0, Abnormal/Present | 1, Normal/absent
**cns_reg_wlk_tst_dtls**		text	Comments:	
**cns_toe_wlk_tst**		radio	Toe walking test	0, Abnormal/Present | 1, Normal/absent
**cns_toe_wlk_tst_dtls**		text	Comments:	
**cns_heel_wlk_tst**		radio	Heel walking test	0, Abnormal/Present | 1, Normal/absent
**cns_heel_wlk_tst_dtls**		text	Comments:	
**cns_tndm_wlk_tst**		radio	Tandem walking test	0, Abnormal/Present | 1, Normal/absent
**cns_tndm_wlk_tst_dtls**		text	Comments:	
**cns_hp_tst**		radio	Hopping test	0, Abnormal/Present | 1, Normal/absent
**cns_hp_tst_dtls**		text	Comments:	
**cns_romberg_tst**		radio	Romberg test	0, Abnormal/Present | 1, Normal/absent
**cns_romberg_tst_dtls**		text	Comments:	
**cns_chorea**	Involuntary Movement	radio	Chorea	0, Abnormal/Present | 1, Normal/absent
**cns_chorea_dtls**		text	Comments:	
**cns_dystonia**		radio	Dystonia	0, Abnormal/Present | 1, Normal/absent
**cns_dystonia_dtls**		text	Comments:	

**cns_myoclonus**		radio	Myoclonus	0, Abnormal/Present | 1, Normal/absent
**cns_myoclonus_dtls**		text	Comments:	
**cns_choreoathetosis**		radio	Choreoathetosis	0, Abnormal/Present | 1, Normal/absent
**cns_choreoathetosis_dtls**		text	Comments:	
**cns_ballismus**		radio	Ballismus	0, Abnormal/Present | 1, Normal/absent
**cns_ballismus_dtls**		text	Comments:	
**cns_tremor**		radio	Tremor	0, Abnormal/Present | 1, Normal/absent
**cns_tremor_dtls**		text	Comments:	
**cns_im_other**		radio	Other:	0, Abnormal/Present | 1, Normal/absent
**cns_im_other_dtls**		text	Comments:	
**cns_superficial**	Sensation	radio	Superficial	0, Abnormal/Present | 1, Normal/absent
**cns_superficial_dtls**		text	Comments:	
**cns_deep**		radio	Deep	0, Abnormal/Present | 1, Normal/absent
**cns_deep_dtls**		text	Comments:	
**cns_cortical**		radio	Cortical	0, Abnormal/Present | 1, Normal/absent
**cns_cortical_dtls**		text	Comments:	
**cns_chcking_rebnd**	Cerebellar Function	radio	Checking and rebound	0, Abnormal/Present | 1, Normal/absent

**cns_chcking_rebnd_dtls**		text	Comments:	
**cns_speech_coord**		radio	Speech coordination	0, Abnormal/Present | 1, Normal/absent
**cns_speech_coord_dtls**		text	Comments:	
**cns_alt_supin_pron**		radio	Alternate supination-pronation	0, Abnormal/Present | 1, Normal/absent
**cns_alt_supin_pron_dtls**		text	Comments:	
**cns_fng_ns_fng**		radio	Finger-nose-finger	0, Abnormal/Present | 1, Normal/absent
**cns_fng_ns_fng_dtls**		text	Comments:	
**cns_pendular_refl**		radio	Pendular reflexes	0, Abnormal/Present | 1, Normal/absent
**cns_pendular_refl_dtls**		text	Comments:	
**cns_eye_mvt**		radio	Eye movements	0, Abnormal/Present | 1, Normal/absent
**cns_eye_mvt_dtls**		text	Comments:	
**cns_truncal_coord**		radio	Truncal coordination	0, Abnormal/Present | 1, Normal/absent
**cns_truncal_coord_dtls**		text	Comments:	
**cns_head_coord**		radio	Head coordination	0, Abnormal/Present | 1, Normal/absent
**cns_head_coord_dtls**		text	Comments:	
**cns_distalbulk_r**	Muscle Bulk, Tone and Strength	radio	Distal bulk Right	0, Abnormal/Present | 1, Normal/absent

**cns_distalbulk_r_dtls**		text	Comments:	
**cns_distalbulk_l**		radio	Distal bulk Left	0, Abnormal/Present | 1, Normal/absent
**cns_distalbulk_l_dtls**		text	Comments:	
**cns_distaltone_r**		radio	Distal tone Right	0, Abnormal/Present | 1, Normal/absent
**cns_distaltone_r_dtls**		text	Comments:	
**cns_distaltone_l**		radio	Distal tone Left	0, Abnormal/Present | 1, Normal/absent
**cns_distaltone_l_dtls**		text	Comments:	
**cns_distalstrength_r**		radio	Distal strength Right	0, Abnormal/Present | 1, Normal/absent
**cns_distalstrength_r_dtls**		text	Comments:	
**cns_distalstrength_l**		radio	Distal strength Left	0, Abnormal/Present | 1, Normal/absent
**cns_distalstrength_l_dtls**		text	Comments:	
**cns_proximalbulk_r**		radio	Proximal bulk Right	0, Abnormal/Present | 1, Normal/absent
**cns_proximalbulk_r_dtls**		text	Comments:	
**cns_proximalbulk_l**		radio	Proximal bulk Left	0, Abnormal/Present | 1, Normal/absent
**cns_proximalbulk_l_dtls**		text	Comments:	
**cns_proximaltone_r**		radio	Proximal tone Right	0, Abnormal/Present | 1, Normal/absent
**cns_proximaltone_r_dtls**		text	Comments:	

**cns_proximaltone_l**		radio	Proximal tone Left	0, Abnormal/Present | 1, Normal/absent
**cns_proximaltone_l_dtls**		text	Comments:	
**cns_proximalstr_r**		radio	Proximal strength Right	0, Abnormal/Present | 1, Normal/absent
**cns_proximalstr_r_dtls**		text	Comments:	
**cns_proximalstr_l**		radio	Proximal strength Left	0, Abnormal/Present | 1, Normal/absent
**cns_proximalstr_l_dtls**		text	Comments:	
**cns_aj_reflex_l**	Myotatic Reflexes For this section only, code using the standard 0-4 clinical scale.	radio	AJ_reflex: left	0,0 | 1,1 | 2,2 | 1.001,3 |0.001,4
**cns_aj_reflex_r**		radio	AJ_reflex: Right	0,0 | 1,1 | 2,2 | 1.001,3 |0.001,4
**cns_kj_reflex_l**		radio	KJ_reflex: left	0,0 | 1,1 | 2,2 | 1.001,3 |0.001,4
**cns_kj_reflex_r**		radio	KJ_reflex:Right	0,0 | 1,1 | 2,2 | 1.001,3 |0.001,4
**cns_wj_reflex_l**		radio	WJ_reflex left	0,0 | 1,1 | 2,2 | 1.001,3 |0.001,4
**cns_wj_reflex_r**		radio	WJ_reflex: Right	0,0 | 1,1 | 2,2 | 1.001,3 |0.001,4
**cns_bj_reflex_l**		radio	BJ_reflex: left	0,0 | 1,1 | 2,2 | 1.001,3 |0.001,4
**cns_bj_reflex_r**		radio	BJ_reflex: Right	0,0 | 1,1 | 2,2 | 1.001,3 |0.001,4
**cns_tj_reflex_l**		radio	TJ_reflex: Left	0,0 | 1,1 | 2,2 | 1.001,3 |0.001,4

**cns_tj_reflex_r**		radio	TJ_reflex: Right	0,0 | 1,1 | 2,2 | 1.001,3 |0.001,4
**cns_right_toe**	Toe Sign	radio	Right toe	0, Abnormal/Present | 1, Normal/absent
**cns_right_toe_dtls**		text	Comments:	
**cns_left_toe**		radio	Left toe	0, Abnormal/Present | 1, Normal/absent
**cns_left_toe_dtls**		text	Comments:	
**cns_other**	other Findings	radio	other Findings	0, Abnormal/Present | 1, Normal/absent
**cns_other_dtls**		text	Comments:	
**cnsa749a906_addtnl**		notes	Additional Comments	
**cns_score_weight**	CNS Score	dropdown	Weight	1, 1. greater than 3%ile|0, 0 less than 3%ile
**cns_score_height**		dropdown	Height	1, 1. greater than 3%ile|0, 0 less than 3%ile
**cns_score_headcircumference**		dropdown	Head Circumference	1, 1.within 3-97%ile|0, 0 less than 3%ile
**cns_score_generalmedical**		calc	General Medical Exam	[cns_eyes] + [cns_ears] + [cns_nasopharynx] + [cns_lymphnodes] + [cns_heart] + [cns_lungs] + [cns_abdomen] + [cns_skeletal] + [cns_skin] + [cns_vascular] + [cns_carotid_artry] + [cns_temporal_artry] + [cns_bruits]
**cns_score_ocularfundi**		calc	Ocular Fundi	[cns_retinal_vssls] + [cns_optic_ppll] + [cns_macula_regn] + [cns_peripheral_rtn]
**cns_score_cranialnerves**		calc	Cranial Nerves	[cns_crnl_nrv_i] + [cns_crnl_nrv_ii] + [cns_crnl_nrv_iii] + [cns_crnl_nrv_iv] + [cns_crnl_nrv_v] + [cns_crnl_nrv_vi] + [cns_crnl_nrv_vii] + [cns_crnl_nrv_viii] + [cns_crnl_nrv_ix] + [cns_crnl_nrv_x] + [cns_crnl_nrv_xi] + [cns_crnl_nrv_xii]

**cns_score_stancegait**		calc	Stance&Gait	[cns_gait_statn] + [cns_reg_wlk_tst] + [cns_toe_wlk_tst] + [cns_heel_wlk_tst] + [cns_tndm_wlk_tst] + [cns_hp_tst] + [cns_romberg_tst]
**cns_score_involuntarymovements**		calc	Involuntary Movements	[cns_chorea] + [cns_dystonia] + [cns_myoclonus] + [cns_choreoathetosis] + [cns_ballismus] + [cns_tremor] + [cns_im_other]
**cns_score_sensation**		calc	Sensation	[cns_superficial] + [cns_deep] + [cns_cortical]
**cns_score_cerebellarfunction**		calc	Cerebellar Function	[cns_chcking_rebnd] + [cns_speech_coord] + [cns_alt_supin_pron] + [cns_fng_ns_fng] + [cns_pendular_refl] + [cns_eye_mvt] + [cns_truncal_coord] + [cns_head_coord]
**cns_score_musclebulk**		calc	Muscle Bulk,Tone&Strength	([cns_distalbulk_r] + [cns_distalbulk_l] + [cns_distaltone_r] + [cns_distaltone_l] + [cns_distalstrength_r] + [cns_distalstrength_l] + [cns_proximalbulk_r] + [cns_proximalbulk_l] + [cns_proximaltone_r] + [cns_proximaltone_l] + [cns_proximalstr_r] + [cns_proximalstr_l])/2
**cns_score_myotaticreflexes**		calc	Myotatic Reflexes	round(([cns_aj_reflex_l] + [cns_aj_reflex_r] + [cns_kj_reflex_l] + [cns_kj_reflex_r] + [cns_wj_reflex_l] + [cns_wj_reflex_r] + [cns_bj_reflex_l] + [cns_bj_reflex_r] + [cns_tj_reflex_l] + [cns_tj_reflex_r])/2,0)
**cns_score_toesign**		calc	Toe Sign	[cns_right_toe] + [cns_left_toe]
**cns_score_otherfindings**		calc	Other Findings	[cns_other]
**cns_score_total**		calc	Columbia Neurologic Score:	[cns_score_weight] + [cns_score_height] + [cns_score_generalmedical] + [cns_score_ocularfundi] + [cns_score_cranialnerves] + [cns_score_stancegait] + [cns_score_involuntarymovements] + [cns_score_sensation] + [cns_score_cerebellarfunction] + [cns_score_musclebulk] + [cns_score_myotaticreflexes] + [cns_score_toesign] + [cns_score_otherfindings]

1.2.3.2 Newcastle Mitochondrial Disease Adult Scale

RedCAP Form Name: nmdas

Description: Newcastle Mitochondrial Disease Adult Scale. Clinical rating scale designed specifically for the assessment of mitochondrial disease.

Variable / Field Name	Section Header	Field Type	Field Label	Variable / Field Name
**nmdas_id**		text	Participant ID:	
**nmdas_age**		calc	Age of assessment	datediff([nmdas_dob], [nmdas_date],"y")
**nmdas_date**		text	Date of assessment	
**nmdas_cf_1**	Section I - Current Function<br><br>Rate function over the preceding 4-week period, according to patient and/or caregiver interview only. The clinician's subjective judgement of functional ability should not be taken into account.	radio	1. Vision with usual glasses or contact leases	0, Normal | 1, No functional impairment but aware of worsened acuities. | 2, Mild - difficulty with small print or text on television. | 3, Moderate - difficulty outside the home (e.g. bus numbers, road signs or shopping). | 4, Severe - difficulty recognizing faces. | 5, Unable to navigate without help (e.g. carer, dog, cane).
**nmdas_cf_2**		radio	2. Hearing with or without hearing aid	0, Normal | 1, No communication problems but aware of tinnitus or deterioration from prior "normal hearing. | 2, Mid deafness (e.g. missing words in presence of background noise). Fully corrected with hearing aid. | 3, Moderate deafness (e.g. regularly requiring repetition). Not fully corrected with hearing aid. | 4, Severe deafness - poor hearing even with aid (see 3 above). | 5, End stage - virtually no hearing despite aid. Relies heavily on non-verbal communication (e.g. lip reading) or has cochlear implant.
**nmdas_cf_3**		radio	3. Speech	0, Normal | 1, Communication unaffected but patient or others aware of changes in speech patterns or quality. | 2, Mild difficulties - usually understood and rarely asked to repeat things. | 3, Moderate difficulties - poorly understood by strangers and frequently asked to repeat things. | 4, Severe difficulties - poorly understood by family or friends. | 5, Not understood by family or friends. Requires communication aid.

**nmdas_cf_4**		radio	4. Swallowing	0, Normal | 1, Mild-sensation of solids sticking (occasional). | 2, Sensation of Solids sticking (most meals) or need to modify diet (e.g. avoidance of steak/salad) | 3, Difficulty Swallowing solids - affecting meal size or duration. Coughing, choking or nasal regurgitation infrequent (1 to 4 times per month) but more than peers. | 4, Requires adapted diet - regular coughing, choking, or nasal regurgitation (more than once per week) | 5, Requiring enteral feeding (e.g. PEG).
**nmdas_cf_5**		radio	5. Handwriting	0, Normal | 1, Writing speed unaffected but aware of increasing untidiness. | 2, Mild - Has to write slower to maintain tidiness/legibility. | 3, Moderate - Handwriting takes at least twice as long or resorts to printing (must previously have used joined writing). | 4, Severe - Handwriting mostly illegible. Printing very slow and untidy (e.g. "THE BLACKCAT Takes in excess of 30 seconds). | 5, Unable to write. No legible words.
**nmdas_cf_6**		radio	6. Cutting food and handling utensils (irrespective of contributory factors. e.g. weakness, coordination, cognitive function etc. This is also true for questions 7-10)	0, Normal | 1, Slightly slow and/or clumsy but minimal effect on meal duration. | 2, Slow and/or clumsy with extended mea duration, but o help required. | 3, Difficulty cutting lip food and inaccuracy of transfer pronounced. Can manage alone but avoids problem foods (e.g. peas) or carer typically offers minor assistance (e.g. cutting up steak). | 4, Unable to cut up food. Can pass food to mouth with great effort or inaccuracy. Resultant intake minimal. Requires major assistance. | 5, Needs to be fed.
**nmdas_cf_7**		radio	7. Dressing	0, Normal | 1, Occasional difficulties (e.g. shoe laces, buttons etc.) but no real impact on time or effort. Carer typically helps with difficult tasks such as shoe laces or buttons. | 2, Mild - dressing takes longer and requires more effort than expected at the patient's age. No help required. | 3, Moderate - Can dress unaided but takes at least twice as long and is a major effort. Carer typically helps with difficult tasks such as shoe laces or buttons. | 4, Severe - Unable to dress without help but some tasks completed unaided. | 5, Needs to be dressed.
**nmdas_cf_8**		radio	8. Hygiene	0, Normal | 1, Occasional difficulties only but no real impact on time or effort required. | 2, Mild- hygienic care takes longer but quality unaffected. | 3, Moderate - bathes and showers alone with difficulty or needs bath chair/modifications. Dexterous tasks (e.g. brushing teeth, combing hair) performed poorly. | 4, Severe - unable to bathe or shower without help. Major difficulty using toilet alone. Dexterous tasks require help. | 5, Dependent on carers to wash, bathe and toilet
**nmdas_cf_9**		radio	9. Exercise Tolerance.	0, Normal | 1, Unlimited on flat - symptomatic on inclines or stairs. | 2, Able to walk < 1000m on the flat. Restricted on inclines or stairs-rest needed after flight (12

				steps). | 3, Able to walk < 500m on the flat, Rest needed after 8 steps on stairs. | 4, Able to walk < 100m on the flat. Rest needed after 4 steps on stairs. | 5, Able to walk < 25m on the fat, Unable to do stairs alone.
**nmdas_cf_10**		radio	10. Gait Stability	0, Normal | 1, Normal gait - occasional difficulties on turns, uneven ground, or if required to balance on narrow base. | 2, Gait reasonably steady. Aware of impaired balance. Occasionally off balance when walking. | 3, Unsteady gait. Always off balance when walking. Occasional falls. Gait steady with support of stick or person. | 4, Gait grossly unsteady without support. High likelihood of falls. Can only walk short distances (<10m) without support. | 5, Unable to walk without support. Falls on standing
**nmdas_ssi_descrp**		descriptive	Section II - System Specific Involvement<br>Rate function according to patient and/or caregiver interview and consultation with the medical notes. Each inquiry should take into account the situation for the preceding 12-month period only, unless otherwise stated in the question.	
**nmdas_ssi_1**		radio	1. Psychiatric	0, None. | 1, Mild & transient (e.g. reactive depression) - lasting less than 3 months | 2, Mild & persistent (lasting more than 3 months) or recurrent. Patient has consulted GP. | 3, Moderate & warranting specialist treatment (e.g. from a psychiatrist) - e.g. bipolar disorder or depression with vegetative symptoms (insomnia, anorexia, abulia etc.) | 4, Severe (e.g. self-harm - psychosis etc.). | 5, Institutionalized or suicide attempt.
**nmdas_ssi_2**		radio	2. Migraine Headaches during the last 3 months, how many days have headaches prevented the patient from functioning normally at	0, No past history | 1, Asymptomatic but past history of migraines. | 2, One day per month. | 3, Two days per month. | 4, Three days per month. | 5, Four days per month or more.

			school, work, or in the home?	
**nmdas_ssi_3**		radio	3. Seizures	0, No past history | 1, Asymptomatic but past history of epilepsy. | 2, Myoclonic or simple partial seizures only. | 3, Multiple absence, complex partial, or myoclonic seizures affecting function or single generalized Seizure. | 4, Multiple generalized seizures. | 5, Status epilepticus.
**nmdas_ssi_4**		radio	4. Stroke - like episodes (exclude focal deficits felt to be of vascular etiology)	0, None. | 1, Transient focal sensory symptoms only (less than 24 hours). | 2, Transient focal motor symptoms only (less than 24 hours). | 3, Single stroke-like episode affecting one hemisphere (more than 24 hours). | 4, Single stroke-like episode affecting both hemispheres (more than 24 hours). | 5, Multiple stroke-like episodes (more than 24 hours each).
**nmdas_ssi_5**		radio	5. Encephalopathic episodes	0, No past history. | 1, Asymptomatic but past history of encephalopathy. | 2, Mild - single episode of personality or behavioral change but retaining orientation in time/space/person. 3, Moderate - single episode of confusion or disorientation in time, place or person. | 4, Severe - multiple moderate episodes (as above) or emergency hospital admission due to encephalopathy without associated seizures or stroke-like episodes. | 5, Very severe - in association with seizures, strokes or gross lactic acidaemia.
**nmdas_ssi_6**		radio	6. Gastro-intestinal symptoms	0, None | 1, Mild constipation only or past history of bowel resection for dysmotility. | 2, Occasional symptoms of "irritable bowel (pain, bloating or diarrhea) with long spells of normality. | 3, Frequent symptoms (as above) most weeks or severe constipation with bowels open less than once week or need for daily medications | 4, Dysmotility requiring admission or persistent and/or recurrent anorexia/vomiting/weight loss. | 5, Surgical procedures or resections for gastrointestinal dysmotility.
**nmdas_ssi_7**		radio	7. Diabetes mellitus	0, None | 1, Past history of gestational diabetes or transient glucose intolerance related to intercurrent illness. | 2, Impaired glucose tolerance (in absence of intercurrent illness). | 3, NIDDM (Diet). | 4, NIDDM (Tablets). | 5, DM requiring insulin (irrespective of treatment at onset).
**nmdas_ssi_8**		radio	8. Respiratory muscle weakness	0, FVC normal (>=85% predicted). | 1, FVC < 85% predicted. | 2, FVC < 75% predicted. | 3, FVC < 65% predicted | 4, FVC < 55% predicted. | 5, FVC < 45% predicted or ventilatory support for over 6 hours per 24 hr. period (not for OSA alone).

**nmdas_ssi_9**		radio	9. Cardiovascular system.	0, None | 1, Asymptomatic ECG change. | 2, Asymptomatic LVH on echo or non-sustained brady/tachyarrhythmia on ECG. | 3, Sustained or symptomatic arrhythmia, LVH or cardiomyopathy. Dilated chambers Or reduced function on echo. Mobitz II AV black or greater. | 4, Requires pacemaker, defibrillator, arrhythmia ablation, or LVEF <35% on echocardiogram. | 5, Symptoms of left ventricular failure with clinical and/or X-ray evidence of pulmonary edema or LVEF < 30% on echocardiogram.
**nmdas_cca_descp**		descriptive	Section III - Current Clinical Assessment<br>Rate current status according to examination performed at the time of assessment.	
**nmdas_cca_1**		radio	1. Visual acuity with usual glasses, contact lenses or pinhole.	0, CSD ≤ 12 (i.e. normal vision - 676, 676 or better). | 1, CSD ≤ 18 (e.g., 6/9, 6/9). | 2, CSD ≤ 36 (e.g., 6/12, 6/24). | 3, CSD ≤ 60 (e.g., 6/24, 6/36). | 4, CSD ≤ 96 (e.g., 6/60, 6/36). | 5, CSD ≤ 120 (e.g., 6/60, 6760 or worse)
**nmdas_cca_2**		radio	2. Ptosis	0, None | 1, Mild ptosis -- not obscuring either pupil. | 2, Unilateral ptosis obscuring <1/3 of pupil | 3, Bilateral ptosis obscuring < 1/3 or unilateral ptosis obscuring > 1/3 of pupil or prior unilateral surgery. | 4, Bilateral ptosis obscuring > 1/3 of pupils or prior bilateral surgery. | 5, Bilateral ptosis obscuring >2/3 of pupils or > f3 of pupils despite prior bilateral surgery.
**nmdas_cca_3**		radio	3. Chronic Progressive External Ophthalmoplegia	0, None | 1, Some restriction of eye movement (any direction). Abduction complete. | 2, Abduction of worst eye incomplete. | 3, Abduction of worst eye below 60% of normal. | 4, Abduction of worst eye below 30% of normal. | 5, Abduction of worst eye minimal (flicker).
**nmdas_cca_4**		radio	4. Dysphonia/ Dysarthria	0, None. | 1, Minimal - noted on examination only. | 2, Mild - Clear impairment buy easily understood. | 3, Moderate - some words poorly understood and its frequent repetition needed. | 4, Severe - many words poorly understood and frequent repetition needed. | 5, Not understood. Requires communication aid.
**nmdas_cca_5**		radio	5. Myopathy	0, Normal | 1, Minimal reduction on hip flexion and/or shoulder abduction only (e.g., MRC 4+/5) | 2, Mild but clear proximal weakness in hip flexion and shoulder abduction (MRC 4/5). Minimal weakness in elbow flexion and knee extension (MRC 4+/5 - both examined with joint at 90 degrees). | 3, Moderate proximal

				weakness including elbow flexion & knee extension (MRC 4/5/ OR 4-/5) or difficulty rising from a 90-degree squat. | 4, Waddling gait. Unable to rise from a 90 degree squat (=a chair) unaided. | 5, Wheelchair dependent primarily due to proximal weakness.
**nmdas_cca_6**		radio	6. Cerebellar ataxia	0, None. | 1, Normal gait but hesitant heel-toe. | 2, Gait reasonably steady. Unable to maintain heel-toe walking or mild UL dysmetria. | 3, Ataxic gait (but walks unaided) or UL intention tremor & past-pointing. Unable to walk heel-toe - falls immediately. | 4, Severe-gait grossly unsteady without support or UL ataxia sufficient to affect feeding. | 5, Wheelchair dependent primarily due to ataxia or UL ataxia prevents feeding.
**nmdas_cca_7**		radio	7. Neuropathy	0, None | 1, Subtle sensory symptoms or areflexia. | 2, Sensory impairment only (e.g., glove & stocking sensory loss). | 3, Motor impairment (distal weakness) or sensory ataxia. | 4, Sensory ataxia or motor effects severely limit ambulation. | 5, Wheelchair bound primarily due to sensory ataxia or neurogenic weakness.
**nmdas_cca_8**		radio	8. Pyramidal Involvement	0, None. | 1, Focal or generalized increase in tone or reflexes only. | 2, Mild focal weakness, sensory loss or fine motor impairment (e.g., cortical hand). | 3, Moderate hemiplegia allowing unaided ambulation or dense UL monoplegia. | 4, Severe hemiplegia allowing ambulation with aids or moderate tetraplegia (ambulant). | 5, Wheelchair dependent primarily due to hemiplegia of tetraplegia.
**nmdas_cca_9**		radio	9. Extrapyramidal	0, Normal. | 1, Mild and Unilateral. Not disabling (H&Y stage 1). | 2, Mild and bilateral. Minimal disability. Gait affected (H&Y stage 2). | 3, Moderate. Significant slowing of body movements (H&Y stage 3). | 4, Severe. Rigidity and bradykinesia. Unable to live alone. Can walk to limited extent, (H&Y stage 4). | 5, Cannot walk or Stand unaided. Requires constant nursing care (H&Y stage 5).
**nmdas_cca_10**		radio	10. Cognition<br>Patients undergo testing using WTAR, Symbol Search and Speed of comprehension test.	0, Combined centiles 100 or more. | 1, Combined centiles 60 - 99 | 2, Combined centiles 30 - 59 | 3, Combined centiles 15 - 29 | 4, Combined centiles 5 - 14 | 5, Combined centiles 4 or below.
**nmdas_i_ii_iii_score**	Total score for sections I, II and III	calc	Score:	[nmdas_cf_1] + [nmdas_cf_2] + [nmdas_cf_3] + [nmdas_cf_4] + [nmdas_cf_5] + [nmdas_cf_6] + [nmdas_cf_7] + [nmdas_cf_8] + [nmdas_cf_9] + [nmdas_cf_10] + [nmdas_ssi_1] + [nmdas_ssi_2] + [nmdas_ssi_3] + [nmdas_ssi_4] + [nmdas_ssi_5] + [nmdas_ssi_6] + [nmdas_ssi_7] + [nmdas_ssi_8] + [nmdas_ssi_9] + [nmdas_cca_1] + [nmdas_cca_2] + [nmdas_cca_3] +

				[nmdas_cca_4] + [nmdas_cca_5] + [nmdas_cca_6] + [nmdas_cca_7] + [nmdas_cca_8] + [nmdas_cca_9]

1.2.3.3 Newcastle Mitochondrial Disease Adult Scale Scoring

RedCAP Form Name: nmdas scoring

Description: Scores from the nmdas scale, completed during the medical assessment Clinical rating scale designed specifically for the assessment of mitochondrial disease.

Variable / Field Name	Section Header	Field Type	Field Label
**nmdas_chlst**	Checklist - please tick off when completed.	descriptive	
**nmdas_height**		text	Height
**nmdas_fvc1**		text	FVC-1 attempt:
**nmdas_fvc2**		text	FVC-2 attempt:
**nmdas_fvc3**		text	FVC-3 attempt:
**nmdas_fvc_prdct**		text	% Predicated
**nmdas_sf12v**	SF-12v2 self completion questionnaire	text	Raw score
**nmdas_sf12v_scald**		text	Scaled Score
**nmdas_sf12v_centile**		text	Centile
**nmdas_wtar**	WTAR reading test (1 minute)	text	Raw score
**nmdas_wtar_scald**		text	Scaled Score
**nmdas_wtar_centile**		text	Centile

**nmdas_symbolsrch**	Symbol Search (2 minutes)	text	Raw score
**nmdas_symbolsrch_scald**		text	Scaled Score
**nmdas_symbolsrch_centile**		text	Centile
**nmdas_speachcompreh**	Speed of comprehension test (2minutes)	text	Raw score
**nmdas_speachcompreh_scald**		text	Scaled Score
**nmdas_speachcompreh_centile**		text	Centile
**nmdas_diseas_scor**		text	Disease Score (sections I-III)
**nmdas_qol_scor**		text	SF-12v2 Quality of Life Score (section IV)

1.2.3.4 Case Report Form – North American Mitochondrial Disease Consortium

RedCAP Form Name: crf namdc

Description: Form completed during the medical assessment

Variable / Field Name	Section Header	Field Type	Field Label	Variable / Field Name
**crf_dob**	Demographics (complete on enrollment)	text	Date of Birth:	
**crf_sex**		radio	Gender:	1, Male | 2, Female
**crf_race**		radio	Race	1, American Indian/Alaska Native | 2, Asian | 3, Native Hawaiian/Pacific Islander | 4, Black/African American | 5, White | 6, More than one | 7, Other
**crf_race_other**		text	Specify:	

**crf_ethni**		yes/no	Hispanic/Latino?	
**crf_consanguineous**		yes/no	Parents consanguineous?	
**crf_certain**	Diagnoses (complete on enrollment)<br>Clinical Diagnosis	radio	Level of certainty:	1, Definite | 2, Possible | 3, Probable | 4, Asymptomatic
**crf_syndrome**		radio	syndrome	1, Alpers syndrome | 2, Cardiomyopathy | 3, Chronic progressive external ophthalmoplegia(CPEO) | 4, CPEO "Plus" | 5, Diabetes and deafness (DAD) | 6, Kearns-Sayre syndrome (KSS) | 7, Leber hereditary optic neuropathy (LHON) | 8, Leigh syndrome | 9, Maternal-inherited deafness | 10, Mitochondrial encephalomyopathy lactic acidosis stroke-like episodes (MELAS) | 11, Mitochondrial neurogastrointestinal encephalomyopathy (MNGIE) | 12, Multi-systemic syndrome | 13, Myoclonus epilepsy ragged-red fibers (MERRF) | 14, Myopathy with ragged-red fibers | 15, Neuropathy ataxia retinitis pigmentosa (NARP) | 16, Pearson syndrome | 17, Reversible infantile myopathy with cytochrome c oxidase deficiency | 18, Sensory ataxic neuropathy dysarthria phthalmoplegia (SANDO) | 19, Other clinical diagnosis, specify: | 20, No clinical symptoms
**crf_yr_onset**		text	Year of onset	
**complex_i_deficiency**		radio	Complex I Deficiency	1, No | 2, Possible | 3, Probable | 4, Definite | 5, Unknown
**crf_biochdef1_yr**		text	Year of onset	

**crf_biochdef2**		radio	Complex II deficiency	1, No | 2, Possible | 3, Probable | 4, Definite | 5, Unknown
**crf_biochdef2_yr**		text	Year of onset	
**crf_biochdef3**		radio	Complex III deficiency	1, No | 2, Possible | 3, Probable | 4, Definite | 5, Unknown
**crf_biochdef3_yr**		text	Year of onset	
**crf_biochdef4**		radio	Complex IV deficiency	1, No | 2, Possible | 3, Probable | 4, Definite | 5, Unknown
**crf_biochdef4_yr**		text	Year of onset	
**crf_biochdef5**		radio	Complex_V_deficiency	1, No | 2, Possible | 3, Probable | 4, Definite | 5, Unknown
**crf_biochdef5_yr**		text	Year of onset	
**crf_biochdef6**		radio	PDC_deficiency	1, No | 2, Possible | 3, Probable | 4, Definite | 5, Unknown
**crf_biochdef6_yr**		text	Year of onset	
**crf_biochdef7**		radio	Oxidative_phosphorylation_deficiency	1, No | 2, Possible | 3, Probable | 4, Definite | 5, Unknown
**crf_biochdef7_yr**		text	Year of onset	
**crf_biochdef8**		radio	Thymidine_phosphorylase_deficiency	1, No | 2, Possible | 3, Probable | 4, Definite | 5, Unknown
**crf_biochdef8_yr**		text	Year of onset	

**crf_biochdef9**		radio	Loose_coupling_of_oxidation_and_phosphorylation	1, No | 2, Possible | 3, Probable | 4, Definite | 5, Unknown
**crf_biochdef9_yr**		text	Year of onset	
**crf_biochdef10**		radio	Coenzyme_Q10_deficiency	1, No | 2, Possible | 3, Probable | 4, Definite | 5, Unknown
**crf_biochdef10_yr**		text	Year of onset	
**crf_litr**		yes/no	Case reported in the literature?	
**crf_litr_dtls**		text	If Yes, reference:	
**crf_manicardio_all_nounk**	Manifestations (complete on enrollment, update at each visit)<br>Cardiological	radio	Mark all as:	1, No | 2, Unknown
**crf_manicardio1**		radio	Arrhythmia	1, No | 2, Yes | 5, Unknown
**crf_manicardio1_cours**		radio	Course	1, Progressive | 2, Stable | 3, Reversible | 4, Recurrent
**crf_manicardio1_yr**		text	Year of onset	
**crf_manicardio2**		radio	Cardiac conduction block	1, No | 2, Yes | 5, Unknown
**crf_manicardio2_cours**		radio	Course	1, Progressive | 2, Stable | 3, Reversible | 4, Recurrent
**crf_manicardio2_yr**		text	Year of onset	
**crf_manicardio3**		radio	Dilated cardiomyopathy	1, No | 2, Yes | 5, Unknown

**crf_manicardio3_cours**		radio	Course	1, Progressive | 2, Stable | 3, Reversible | 4, Recurrent
**crf_manicardio3_yr**		text	Year of onset	
**crf_manicardio4**		radio	Hypertrophic cardiomyopathy	1, No | 2,Yes | 5, Unknown
**crf_manicardio4_cours**		radio	Course	1, Progressive | 2, Stable | 3, Reversible | 4, Recurrent
**crf_manicardio4_yr**		text	Year of onset	
**crf_manicardio5**		radio	Non? compaction cardiomyopathy	1, No | 2,Yes | 5, Unknown
**crf_manicardio5_cours**		radio	Course	1, Progressive | 2, Stable | 3, Reversible | 4, Recurrent
**crf_manicardio5_yr**		text	Year of onset	
**crf_manicardio6**		radio	Wolff?Parkinson?White (WPW)	1, No | 2,Yes | 5, Unknown
**crf_manicardio6_cours**		radio	Course	1, Progressive | 2, Stable | 3, Reversible | 4, Recurrent
**crf_manicardio6_yr**		text	Year of onset	
**crf_manicardio7**		radio	Other cardiological manifestation	1, No | 2,Yes {crf_manicardio7_other}| 5, Unknown
**crf_manicardio7_cours**		radio	Course	1, Progressive | 2, Stable | 3, Reversible | 4, Recurrent
**crf_manicardio7_yr**		text	Year of onset	
**crf_manicardio7_other**		text	other	

**crf_maniconstit_all_nounk**	Constitutional	radio	Mark all as:	1, No | 2, Unknown
**crf_maniconstit1**		radio	Cachexia	1, No | 2,Yes | 5, Unknown
**crf_maniconstit1_cours**		radio	Course	1, Progressive | 2, Stable | 3, Reversible | 4, Recurrent
**crf_maniconstit1_yr**		text	Year of onset	
**crf_maniconstit2**		radio	Chronic fatigue	1, No | 2,Yes | 5, Unknown
**crf_maniconstit2_cours**		radio	Course	1, Progressive | 2, Stable | 3, Reversible | 4, Recurrent
**crf_maniconstit2_yr**		text	Year of onset	
**crf_maniconstit3**		radio	Chronic shortness of breath or exercise intolerance	1, No | 2, Yes | 5, Unknown
**crf_maniconstit3_cours**		radio	Course	1, Progressive | 2, Stable | 3, Reversible | 4, Recurrent
**crf_maniconstit3_yr**		text	Year of onset	
**crf_maniconstit8**		radio	Exercise intolerance	1, No | 2, Yes | 5, Unknown
**crf_maniconstit8_cours**		radio	Course	1, Progressive | 2, Stable | 3, Reversible | 4, Recurrent
**crf_maniconstit8_yr**		text	Year of onset	
**crf_maniconstit4**		radio	Shortness of stature	1, No | 2,Yes | 5, Unknown
**crf_maniconstit4_cours**		radio	Course	1, Progressive | 2, Stable | 3, Reversible | 4, Recurrent

**crf_maniconstit4_yr**		text	Year of onset	
**crf_maniconstit5**		radio	Thinness	1, No | 2,Yes | 5, Unknown
**crf_maniconstit5_cours**		radio	Course	1, Progressive | 2, Stable | 3, Reversible | 4, Recurrent
**crf_maniconstit5_yr**		text	Year of onset	
**crf_maniconstit6**		radio	Weakness	1, No | 2,Yes | 5, Unknown
**crf_maniconstit6_cours**		radio	Course	1, Progressive | 2, Stable | 3, Reversible | 4, Recurrent
**crf_maniconstit6_yr**		text	Year of onset	
**crf_maniconstit7**		radio	Other constitutional manifestation	1, No | 2,Yes {crf_maniconstit7_other} | 5, Unknown
**crf_maniconstit7_cours**		radio	Course	1, Progressive | 2, Stable | 3, Reversible | 4, Recurrent
**crf_maniconstit7_yr**		text	Year of onset	
**crf_maniconstit7_other**		text	other	
**crf_manidevel_all_nounk**	Developmental	radio	Mark all as:	1, No | 2, Unknown
**crf_manidevel1**		radio	Developmental delay	1, No | 2,Yes | 5, Unknown
**crf_manidevel1_cours**		radio	Course	1, Progressive | 2, Stable | 3, Reversible | 4, Recurrent
**crf_manidevel1_yr**		text	Year of onset	

**crf_manidevel2**		radio	Developmental regression	1, No | 2,Yes | 5, Unknown
**crf_manidevel2_cours**		radio	Course	1, Progressive | 2, Stable | 3, Reversible | 4, Recurrent
**crf_manidevel2_yr**		text	Year of onset	
**crf_manidevel3**		radio	Floppy baby	1, No | 2,Yes | 5, Unknown
**crf_manidevel3_cours**		radio	Course	1, Progressive | 2, Stable | 3, Reversible | 4, Recurrent
**crf_manidevel3_yr**		text	Year of onset	
**crf_manidevel4**		radio	Mental retardation	1, No | 2,Yes | 5, Unknown
**crf_manidevel4_cours**		radio	Course	1, Progressive | 2, Stable | 3, Reversible | 4, Recurrent
**crf_manidevel4_yr**		text	Year of onset	
**crf_manidevel5**		radio	Other developmental manifestation	1, No | 2,Yes {crf_manidevel5_other}| 5, Unknown
**crf_manidevel5_cours**		radio	Course	1, Progressive | 2, Stable | 3, Reversible | 4, Recurrent
**crf_manidevel5_yr**		text	Year of onset	
**crf_manidevel5_other**		text	other	
**crf_maniendo_all_nounk**	Endocrine	radio	Mark all as:	1, No | 2, Unknown
**crf_maniendo1**		radio	Diabetes mellitus	1, No | 2,Yes | 5, Unknown

**crf_maniendo1_cours**		radio	Course	1, Progressive | 2, Stable | 3, Reversible | 4, Recurrent
**crf_maniendo1_yr**		text	Year of onset	
**crf_maniendo2**		radio	Hypogonadotropic hypogonadism	1, No | 2, Yes | 5, Unknown
**crf_maniendo2_cours**		radio	Course	1, Progressive | 2, Stable | 3, Reversible | 4, Recurrent
**crf_maniendo2_yr**		text	Year of onset	
**crf_maniendo3**		radio	hypogonadism	1, No | 2, Yes | 5, Unknown
**crf_maniendo3_cours**		radio	Course	1, Progressive | 2, Stable | 3, Reversible | 4, Recurrent
**crf_maniendo3_yr**		text	Year of onset	
**crf_maniendo4**		radio	Hypoparathyroidism	1, No | 2,Yes | 5, Unknown
**crf_maniendo4_cours**		radio	Course	1, Progressive | 2, Stable | 3, Reversible | 4, Recurrent
**crf_maniendo4_yr**		text	Year of onset	
**crf_maniendo5**		radio	Hypothyroidism	1, No | 2,Yes | 5, Unknown
**crf_maniendo5_cours**		radio	Course	1, Progressive | 2, Stable | 3, Reversible | 4, Recurrent
**crf_maniendo5_yr**		text	Year of onset	
**crf_maniendo6**		radio	Other endocrine manifestation	1, No | 2,Yes {crf_maniendo6_other} | 5, Unknown

**crf_maniendo6_cours**		radio	Course	1, Progressive | 2, Stable | 3, Reversible | 4, Recurrent
**crf_maniendo6_yr**		text	Year of onset	
**crf_maniendo6_other**		text	other	
**crf_manigastro_all_nounk**	Gastrointestinal/Hepatic	radio	Mark all as:	1, No | 2, Unknown
**crf_manigastro_1**		radio	Cirrhosis	1, No | 2,Yes | 5, Unknown
**crf_manigastro_1_cours**		radio	Course	1, Progressive | 2, Stable | 3, Reversible | 4, Recurrent
**crf_manigastro_1_yr**		text	Year of onset	
**crf_manigastro_2**		radio	Dysphagia	1, No | 2,Yes | 5, Unknown
**crf_manigastro_2_cours**		radio	Course	1, Progressive | 2, Stable | 3, Reversible | 4, Recurrent
**crf_manigastro_2_yr**		text	Year of onset	
**crf_manigastro_3**		radio	Exocrine pancreatic dysfunciton	1, No | 2,Yes | 5, Unknown
**crf_manigastro_3_cours**		radio	Course	1, Progressive | 2, Stable | 3, Reversible | 4, Recurrent
**crf_manigastro_3_yr**		text	Year of onset	
**crf_manigastro_4**		radio	Gastrointestinal pseudoobstruction	1, No | 2,Yes | 5, Unknown
**crf_manigastro_4_cours**		radio	Course	1, Progressive | 2, Stable | 3, Reversible | 4, Recurrent

**crf_manigastro_4_yr**		text	Year of onset	
**crf_manigastro_5**		radio	Gastroparesis	1, No | 2,Yes | 5, Unknown
**crf_manigastro_5_cours**		radio	Course	1, Progressive | 2, Stable | 3, Reversible | 4, Recurrent
**crf_manigastro_5_yr**		text	Year of onset	
**crf_manigastro_6**		radio	Hepatic failure	1, No | 2,Yes | 5, Unknown
**crf_manigastro_6_cours**		radio	Course	1, Progressive | 2, Stable | 3, Reversible | 4, Recurrent
**crf_manigastro_6_yr**		text	Year of onset	
**crf_manigastro_7**		radio	Hepatic steatosis	1, No | 2,Yes | 5, Unknown
**crf_manigastro_7_cours**		radio	Course	1, Progressive | 2, Stable | 3, Reversible | 4, Recurrent
**crf_manigastro_7_yr**		text	Year of onset	
**crf_manigastro_8**		radio	Hepatopathy	1, No | 2,Yes | 5, Unknown
**crf_manigastro_8_cours**		radio	Course	1, Progressive | 2, Stable | 3, Reversible | 4, Recurrent
**crf_manigastro_8_yr**		text	Year of onset	
**crf_manigastro_9**		radio	Recurrrent nausea/vommiting	1, No | 2,Yes | 5, Unknown
**crf_manigastro_9_cours**		radio	Course	1, Progressive | 2, Stable | 3, Reversible | 4, Recurrent

**crf_manigastro_9_yr**		text	Year of onset	
**crf_manigastro_10**		radio	Other gastroinstestinal/hepatic manifestation, specify	1, No | 2,Yes {crf_manigastro_10_specify}| 5, Unknown
**crf_manigastro_10_cours**		radio	Course	1, Progressive | 2, Stable | 3, Reversible | 4, Recurrent
**crf_manigastro_10_yr**		text	Year of onset	
**crf_manigastro_10_specify**		text	Other gastroinstestinal/hepatic manifestation, specify	
**crf_manineuro_all_nounk**	Neurological	radio	Mark all as:	1, No | 2, Unknown
**crf_manineuro_1**		radio	Ataxia	1, No | 2,Yes | 5, Unknown
**crf_manineuro_1_cours**		radio	Course	1, Progressive | 2, Stable | 3, Reversible | 4, Recurrent
**crf_manineuro_1_yr**		text	Year of onset	
**crf_manineuro_2**		radio	Chorea	1, No | 2,Yes | 5, Unknown
**crf_manineuro_2_cours**		radio	Course	1, Progressive | 2, Stable | 3, Reversible | 4, Recurrent
**crf_manineuro_2_yr**		text	Year of onset	
**crf_manineuro_3**		radio	Dementia	1, No | 2,Yes | 5, Unknown
**crf_manineuro_3_cours**		radio	Course	1, Progressive | 2, Stable | 3, Reversible | 4, Recurrent
**crf_manineuro_3_yr**		text	Year of onset	

**crf_manineuro_4**		radio	Dysarthria	1, No | 2,Yes | 5, Unknown
**crf_manineuro_4_cours**		radio	Course	1, Progressive | 2, Stable | 3, Reversible | 4, Recurrent
**crf_manineuro_4_yr**		text	Year of onset	
**crf_manineuro_5**		radio	Dystonia	1, No | 2,Yes | 5, Unknown
**crf_manineuro_5_cours**		radio	Course	1, Progressive | 2, Stable | 3, Reversible | 4, Recurrent
**crf_manineuro_5_yr**		text	Year of onset	
**crf_manineuro_6**		radio	Headaches (non?migraine)	1, No | 2,Yes | 5, Unknown
**crf_manineuro_6_cours**		radio	Course	1, Progressive | 2, Stable | 3, Reversible | 4, Recurrent
**crf_manineuro_6_yr**		text	Year of onset	
**crf_manineuro_7**		radio	Hearing loss	1, No | 2,Yes | 5, Unknown
**crf_manineuro_7_cours**		radio	Course	1, Progressive | 2, Stable | 3, Reversible | 4, Recurrent
**crf_manineuro_7_yr**		text	Year of onset	
**crf_manineuro_8**		radio	Hyperactive reflexes	1, No | 2,Yes | 5, Unknown
**crf_manineuro_8_cours**		radio	Course	1, Progressive | 2, Stable | 3, Reversible | 4, Recurrent
**crf_manineuro_8_yr**		text	Year of onset	

**crf_manineuro_9**		radio	Migraine headaches	1, No | 2,Yes | 5, Unknown
**crf_manineuro_9_cours**		radio	Course	1, Progressive | 2, Stable | 3, Reversible | 4, Recurrent
**crf_manineuro_9_yr**		text	Year of onset	
**crf_manineuro_10**		radio	Myoclonus	1, No | 2,Yes | 5, Unknown
**crf_manineuro_10_cours**		radio	Course	1, Progressive | 2, Stable | 3, Reversible | 4, Recurrent
**crf_manineuro_10_yr**		text	Year of onset	
**crf_manineuro_11**		radio	Myoglobinuria	1, No | 2,Yes | 5, Unknown
**crf_manineuro_11_cours**		radio	Course	1, Progressive | 2, Stable | 3, Reversible | 4, Recurrent
**crf_manineuro_11_yr**		text	Year of onset	
**crf_manineuro_12**		radio	Myopathy	1, No | 2,Yes | 5, Unknown
**crf_manineuro_12_cours**		radio	Course	1, Progressive | 2, Stable | 3, Reversible | 4, Recurrent
**crf_manineuro_12_yr**		text	Year of onset	
**crf_manineuro_13**		radio	Neuropathy: axonal	1, No | 2,Yes | 5, Unknown
**crf_manineuro_13_cours**		radio	Course	1, Progressive | 2, Stable | 3, Reversible | 4, Recurrent
**crf_manineuro_13_yr**		text	Year of onset	

**crf_manineuro_14**		radio	Neuropathy: demyelinating	1, No | 2,Yes | 5, Unknown
**crf_manineuro_14_cours**		radio	Course	1, Progressive | 2, Stable | 3, Reversible | 4, Recurrent
**crf_manineuro_14_yr**		text	Year of onset	
**crf_manineuro_15**		radio	Ophthalmoparesis	1, No | 2,Yes | 5, Unknown
**crf_manineuro_15_cours**		radio	Course	1, Progressive | 2, Stable | 3, Reversible | 4, Recurrent
**crf_manineuro_15_yr**		text	Year of onset	
**crf_manineuro_16**		radio	Parkinsonism	1, No | 2,Yes | 5, Unknown
**crf_manineuro_16_cours**		radio	Course	1, Progressive | 2, Stable | 3, Reversible | 4, Recurrent
**crf_manineuro_16_yr**		text	Year of onset	
**crf_manineuro_17**		radio	Ptosis	1, No | 2,Yes | 5, Unknown
**crf_manineuro_17_cours**		radio	Course	1, Progressive | 2, Stable | 3, Reversible | 4, Recurrent
**crf_manineuro_17_yr**		text	Year of onset	
**crf_manineuro_18**		radio	Seizures	1, No | 2,Yes | 5, Unknown
**crf_manineuro_18_cours**		radio	Course	1, Progressive | 2, Stable | 3, Reversible | 4, Recurrent
**crf_manineuro_18_yr**		text	Year of onset	

**crf_manineuro_19**		radio	Spasticity	1, No | 2,Yes | 5, Unknown
**crf_manineuro_19_cours**		radio	Course	1, Progressive | 2, Stable | 3, Reversible | 4, Recurrent
**crf_manineuro_19_yr**		text	Year of onset	
**crf_manineuro_20**		radio	Strokes or stroke like lesions	1, No | 2, Yes | 5, Unknown
**crf_manineuro_20_cours**		radio	Course	1, Progressive | 2, Stable | 3, Reversible | 4, Recurrent
**crf_manineuro_20_yr**		text	Year of onset	
**crf_manineuro_21**		radio	lesions	1, No | 2, Yes | 5, Unknown
**crf_manineuro_21_cours**		radio	Course	1, Progressive | 2, Stable | 3, Reversible | 4, Recurrent
**crf_manineuro_21_yr**		text	Year of onset	
**crf_manineuro_22**		radio	Other encephalopathy	1, No | 2,Yes | 5, Unknown
**crf_manineuro_22_cours**		radio	Course	1, Progressive | 2, Stable | 3, Reversible | 4, Recurrent
**crf_manineuro_22_yr**		text	Year of onset	
**crf_manineuro_23**		radio	Other neurological manifestation	1, No | 2,Yes {crf_manineuro_23_other} | 5, Unknown
**crf_manineuro_23_cours**		radio	Course	1, Progressive | 2, Stable | 3, Reversible | 4, Recurrent
**crf_manineuro_23_yr**		text	Year of onset	

**crf_manineuro_23_other**		text	other	
**crf_maniopthal_all_nounk**	Opthalmological	radio	Mark all as:	1, No | 2, Unknown
**crf_maniopthal1**		radio	Cataracts	1, No | 2,Yes | 5, Unknown
**crf_maniopthal1_cours**		radio	Course	1, Progressive | 2, Stable | 3, Reversible | 4, Recurrent
**crf_maniopthal1_yr**		text	Year of onset	
**crf_maniopthal2**		radio	Optic neuropathy	1, No | 2,Yes | 5, Unknown
**crf_maniopthal2_cours**		radio	Course	1, Progressive | 2, Stable | 3, Reversible | 4, Recurrent
**crf_maniopthal2_yr**		text	Year of onset	
**crf_maniopthal3**		radio	Pigmentary retinopathy	1, No | 2,Yes | 5, Unknown
**crf_maniopthal3_cours**		radio	Course	1, Progressive | 2, Stable | 3, Reversible | 4, Recurrent
**crf_maniopthal3_yr**		text	Year of onset	
**crf_maniopthal4**		radio	Other ophthalmological manifestation	1, No | 2,Yes {crf_maniopthal4_other}| 5, Unknown
**crf_maniopthal4_cours**		radio	Course	1, Progressive | 2, Stable | 3, Reversible | 4, Recurrent
**crf_maniopthal4_yr**		text	Year of onset	
**crf_maniopthal4_other**		text	other	

**crf_maniopsych_all_nounk**	Psychiatric	radio	Mark all as:	1, No | 2, Unknown
**crf_manipsych1**		radio	Anxiety	1, No | 2,Yes | 5, Unknown
**crf_manipsych1_cours**		radio	Course	1, Progressive | 2, Stable | 3, Reversible | 4, Recurrent
**crf_manipsych1_yr**		text	Year of onset	
**crf_manipsych2**		radio	Autism	1, No | 2,Yes | 5, Unknown
**crf_manipsych2_cours**		radio	Course	1, Progressive | 2, Stable | 3, Reversible | 4, Recurrent
**crf_manipsych2_yr**		text	Year of onset	
**crf_manipsych3**		radio	Bipolar disorder	1, No | 2,Yes | 5, Unknown
**crf_manipsych3_cours**		radio	Course	1, Progressive | 2, Stable | 3, Reversible | 4, Recurrent
**crf_manipsych3_yr**		text	Year of onset	
**crf_manipsych4**		radio	Depression	1, No | 2,Yes | 5, Unknown
**crf_manipsych4_cours**		radio	Course	1, Progressive | 2, Stable | 3, Reversible | 4, Recurrent
**crf_manipsych4_yr**		text	Year of onset	
**crf_manipsych5**		radio	Other psychiatric manifestation	1, No | 2,Yes {crf_manipsych5_other} | 5, Unknown
**crf_manipsych5_cours**		radio	Course	1, Progressive | 2, Stable | 3, Reversible | 4, Recurrent

**crf_manipsych5_yr**		text	Year of onset	
**crf_manipsych5_other**		text	other	
**crf_maniorenal_all_nounk**	Renal	radio	Mark all as:	1, No | 2, Unknown
**crf_manirenal1**		radio	Renal tubular acidosis	1, No | 2,Yes | 5, Unknown
**crf_manirenal1_cours**		radio	Course	1, Progressive | 2, Stable | 3, Reversible | 4, Recurrent
**crf_manirenal1_yr**		text	Year of onset	
**crf_manirenal2**		radio	Nephrotic syndrome	1, No | 2,Yes | 5, Unknown
**crf_manirenal2_cours**		radio	Course	1, Progressive | 2, Stable | 3, Reversible | 4, Recurrent
**crf_manirenal2_yr**		text	Year of onset	
**crf_manirenal3**		radio	Other renal manifestation	1, No | 2,Yes | 5, Unknown
**crf_manirenal3_cours**		radio	Course	1, Progressive | 2, Stable | 3, Reversible | 4, Recurrent
**crf_manirenal3_yr**		text	Year of onset	
**crf_manimisc_all_nounk**	Miscellaneous	radio	Mark all as:	1, No | 2, Unknown
**crf_maniother1**		radio	Lipomas	1, No | 2,Yes | 5, Unknown
**crf_maniother1_cours**		radio	Course	1, Progressive | 2, Stable | 3, Reversible | 4, Recurrent

**crf_maniother1_yr**		text	Year of onset	
**crf_maniother2**		radio	Respiratory insufficienc	1, No | 2,Yes | 5, Unknown
**crf_maniother2_cours**		radio	Course	1, Progressive | 2, Stable | 3, Reversible | 4, Recurrent
**crf_maniother2_yr**		text	Year of onset	
**crf_maniother3**		radio	Sideroblastic anemia	1, No | 2,Yes | 5, Unknown
**crf_maniother3_cours**		radio	Course	1, Progressive | 2, Stable | 3, Reversible | 4, Recurrent
**crf_maniother3_yr**		text	Year of onset	
**crf_maniother4**		radio	Other manifestation	1, No | 2,Yes {crf_maniother4_other}| 5, Unknown
**crf_maniother4_cours**		radio	Course	1, Progressive | 2, Stable | 3, Reversible | 4, Recurrent
**crf_maniother4_yr**		text	Year of onset	
**crf_maniother4_other**		text	other	
**crf_rel1**	Complete one page per relative, for as many 1st and 2nd degree relatives as possible	dropdown	Relationship:	1, Mother | 2, Father | 3, Sister | 4, Half-sister (maternal) | 5, Half-sister (paternal) | 6, Brother | 7, Half-brother (maternal) | 8, Half-brother (paternal) | 9, Grandmother (maternal) | 10, Grandmother (paternal) | 11, Grandfather (maternal) | 12, Grandfather (paternal) | 13, Aunt (maternal) | 14, Aunt (maternal - grandmother only) | 15, Aunt (maternal - grandfather only) | 16, Aunt (paternal) | 24, Aunt (paternal - grandmother only) | 17,

				Aunt (paternal - grandfather only) | 18, Uncle (maternal) | 25, Uncle (maternal - grandmother only) | 26, Uncle (maternal - grandfather only) | 19, Uncle (paternal) | 20, Uncle (paternal - grandmother only) | 21, Uncle (paternal - grandfather only) | 22, Daughter | 23, Son
**crf_rel1_1**	Condition<br>Please choose one of the following answers for each question:	radio	Mitochondrial disease	1, No | 2, Yes | 5, Unkown
**crf_rel1_2**		radio	Shortness	1, No | 2, Yes | 5, Unkown
**crf_rel1_3**		radio	Deafness	1, No | 2, Yes | 5, Unkown
**crf_rel1_4**		radio	Blindness	1, No | 2, Yes | 5, Unkown
**crf_rel1_5**		radio	Migraines	1, No | 2, Yes | 5, Unkown
**crf_rel1_6**		radio	Strokes	1, No | 2, Yes | 5, Unkown
**crf_rel1_7**		radio	Seizures	1, No | 2, Yes | 5, Unkown
**crf_rel1_8**		radio	Incoordination	1, No | 2, Yes | 5, Unkown
**crf_rel1_9**		radio	Ptosis	1, No | 2, Yes | 5, Unkown
**crf_rel1_10**		radio	Limb weakness	1, No | 2, Yes | 5, Unkown
**crf_rel1_11**		radio	Peripheral neuropathy	1, No | 2, Yes | 5, Unkown
**crf_rel1_12**		radio	Cardiomyopathy	1, No | 2, Yes | 5, Unkown
**crf_rel1_13**		radio	Gastrointestinal problems	1, No | 2, Yes | 5, Unkown

**crf_rel1_14**		radio	Diabetes mellitus	1, No | 2, Yes | 5, Unkown
**crf_rel1_15**		radio	Liver disease	1, No | 2, Yes | 5, Unkown
**crf_rel1_16**		radio	Kidney disease	1, No | 2, Yes | 5, Unkown
**crf_rel2_add**		yes/no	Do you want to add another relative?	
**crf_rel2**	Complete one page per relative, for as many 1st and 2nd degree relatives as possible	dropdown	Relationship:	1, Mother | 2, Father | 3, Sister | 4, Half-sister (maternal) | 5, Half-sister (paternal) | 6, Brother | 7, Half-brother (maternal) | 8, Half-brother (paternal) | 9, Grandmother (maternal) | 10, Grandmother (paternal) | 11, Grandfather (maternal) | 12, Grandfather (paternal) | 13, Aunt (maternal) | 14, Aunt (maternal - grandmother only) | 15, Aunt (maternal - grandfather only) | 16, Aunt (paternal) | 24, Aunt (paternal - grandmother only) | 17, Aunt (paternal - grandfather only) | 18, Uncle (maternal) | 25, Uncle (maternal - grandmother only) | 26, Uncle (maternal - grandfather only) | 19, Uncle (paternal) | 20, Uncle (paternal - grandmother only) | 21, Uncle (paternal - grandfather only) | 22, Daughter | 23, Son
**crf_rel2_1**	Condition<br>Please choose one of the following answers for each question:	radio	Mitochondrial disease	1, No | 2, Yes | 5, Unkown
**crf_rel2_2**		radio	Shortness	1, No | 2, Yes | 5, Unkown
**crf_rel2_3**		radio	Deafness	1, No | 2, Yes | 5, Unkown
**crf_rel2_4**		radio	Blindness	1, No | 2, Yes | 5, Unkown

**crf_rel2_5**		radio	Migraines	1, No | 2, Yes | 5, Unkown
**crf_rel2_6**		radio	Strokes	1, No | 2, Yes | 5, Unkown
**crf_rel2_7**		radio	Seizures	1, No | 2, Yes | 5, Unkown
**crf_rel2_8**		radio	Incoordination	1, No | 2, Yes | 5, Unkown
**crf_rel2_9**		radio	Ptosis	1, No | 2, Yes | 5, Unkown
**crf_rel2_10**		radio	Limb weakness	1, No | 2, Yes | 5, Unkown
**crf_rel2_11**		radio	Peripheral neuropathy	1, No | 2, Yes | 5, Unkown
**crf_rel2_12**		radio	Cardiomyopathy	1, No | 2, Yes | 5, Unkown
**crf_rel2_13**		radio	Gastrointestinal problems	1, No | 2, Yes | 5, Unkown
**crf_rel2_14**		radio	Diabetes mellitus	1, No | 2, Yes | 5, Unkown
**crf_rel2_15**		radio	Liver disease	1, No | 2, Yes | 5, Unkown
**crf_rel2_16**		radio	Kidney disease	1, No | 2, Yes | 5, Unkown
**crf_rel3_add**		yes/no	Do you want to add another relative?	
**crf_rel3**	Complete one page per relative, for as many 1st and 2nd degree relatives as possible	dropdown	Relationship:	1, Mother | 2, Father | 3, Sister | 4, Half-sister (maternal) | 5, Half-sister (paternal) | 6, Brother | 7, Half-brother (maternal) | 8, Half-brother (paternal) | 9, Grandmother (maternal) | 10, Grandmother (paternal) | 11, Grandfather (maternal) | 12, Grandfather (paternal) | 13, Aunt (maternal) | 14, Aunt (maternal - grandmother only) | 15, Aunt (maternal - grandfather only) | 16, Aunt (paternal) | 24,

				Aunt (paternal - grandmother only) | 17, Aunt (paternal - grandfather only) | 18, Uncle (maternal) | 25, Uncle (maternal - grandmother only) | 26, Uncle (maternal - grandfather only) | 19, Uncle (paternal) | 20, Uncle (paternal - grandmother only) | 21, Uncle (paternal - grandfather only) | 22, Daughter | 23, Son
**crf_rel3_1**	Condition<br>Please choose one of the following answers for each question:	radio	Mitochondrial disease	1, No | 2, Yes | 5, Unkown
**crf_rel3_2**		radio	Shortness	1, No | 2, Yes | 5, Unkown
**crf_rel3_3**		radio	Deafness	1, No | 2, Yes | 5, Unkown
**crf_rel3_4**		radio	Blindness	1, No | 2, Yes | 5, Unkown
**crf_rel3_5**		radio	Migraines	1, No | 2, Yes | 5, Unkown
**crf_rel3_6**		radio	Strokes	1, No | 2, Yes | 5, Unkown
**crf_rel3_7**		radio	Seizures	1, No | 2, Yes | 5, Unkown
**crf_rel3_8**		radio	Incoordination	1, No | 2, Yes | 5, Unkown
**crf_rel3_9**		radio	Ptosis	1, No | 2, Yes | 5, Unkown
**crf_rel3_10**		radio	Limb weakness	1, No | 2, Yes | 5, Unkown
**crf_rel3_11**		radio	Peripheral neuropathy	1, No | 2, Yes | 5, Unkown
**crf_rel3_12**		radio	Cardiomyopathy	1, No | 2, Yes | 5, Unkown

**crf_rel3_13**		radio	Gastrointestinal problems	1, No | 2, Yes | 5, Unkown
**crf_rel3_14**		radio	Diabetes mellitus	1, No | 2, Yes | 5, Unkown
**crf_rel3_15**		radio	Liver disease	1, No | 2, Yes | 5, Unkown
**crf_rel3_16**		radio	Kidney disease	1, No | 2, Yes | 5, Unkown
**crf_rel4_add**		yes/no	Do you want to add another relative?	
**crf_rel4**	Complete one page per relative, for as many 1st and 2nd degree relatives as possible	dropdown	Relationship:	1, Mother | 2, Father | 3, Sister | 4, Half-sister (maternal) | 5, Half-sister (paternal) | 6, Brother | 7, Half-brother (maternal) | 8, Half-brother (paternal) | 9, Grandmother (maternal) | 10, Grandmother (paternal) | 11, Grandfather (maternal) | 12, Grandfather (paternal) | 13, Aunt (maternal) | 14, Aunt (maternal - grandmother only) | 15, Aunt (maternal - grandfather only) | 16, Aunt (paternal) | 24, Aunt (paternal - grandmother only) | 17, Aunt (paternal - grandfather only) | 18, Uncle (maternal) | 25, Uncle (maternal - grandmother only) | 26, Uncle (maternal - grandfather only) | 19, Uncle (paternal) | 20, Uncle (paternal - grandmother only) | 21, Uncle (paternal - grandfather only) | 22, Daughter | 23, Son
**crf_rel4_1**	Condition<br>Please choose one of the following answers for each question:	radio	Mitochondrial disease	1, No | 2, Yes | 5, Unkown
**crf_rel4_2**		radio	Shortness	1, No | 2, Yes | 5, Unkown
**crf_rel4_3**		radio	Deafness	1, No | 2, Yes | 5, Unkown

**crf_rel4_4**		radio	Blindness	1, No | 2, Yes | 5, Unkown
**crf_rel4_5**		radio	Migraines	1, No | 2, Yes | 5, Unkown
**crf_rel4_6**		radio	Strokes	1, No | 2, Yes | 5, Unkown
**crf_rel4_7**		radio	Seizures	1, No | 2, Yes | 5, Unkown
**crf_rel4_8**		radio	Incoordination	1, No | 2, Yes | 5, Unkown
**crf_rel4_9**		radio	Ptosis	1, No | 2, Yes | 5, Unkown
**crf_rel4_10**		radio	Limb weakness	1, No | 2, Yes | 5, Unkown
**crf_rel4_11**		radio	Peripheral neuropathy	1, No | 2, Yes | 5, Unkown
**crf_rel4_12**		radio	Cardiomyopathy	1, No | 2, Yes | 5, Unkown
**crf_rel4_13**		radio	Gastrointestinal problems	1, No | 2, Yes | 5, Unkown
**crf_rel4_14**		radio	Diabetes mellitus	1, No | 2, Yes | 5, Unkown
**crf_rel4_15**		radio	Liver disease	1, No | 2, Yes | 5, Unkown
**crf_rel4_16**		radio	Kidney disease	1, No | 2, Yes | 5, Unkown
**crf_rel5_add**		yes/no	Do you want to add another relative?	
**crf_rel5**	Complete one page per relative, for as many 1st and 2nd degree relatives as possible	dropdown	Relationship:	1, Mother | 2, Father | 3, Sister | 4, Half-sister (maternal) | 5, Half-sister (paternal) | 6, Brother | 7, Half-brother (maternal) | 8, Half-brother (paternal) | 9, Grandmother (maternal) | 10, Grandmother (paternal) | 11, Grandfather (maternal) | 12, Grandfather (paternal) | 13, Aunt

				(maternal) | 14, Aunt (maternal - grandmother only) | 15, Aunt (maternal - grandfather only) | 16, Aunt (paternal) | 24, Aunt (paternal - grandmother only) | 17, Aunt (paternal - grandfather only) | 18, Uncle (maternal) | 25, Uncle (maternal - grandmother only) | 26, Uncle (maternal - grandfather only) | 19, Uncle (paternal) | 20, Uncle (paternal - grandmother only) | 21, Uncle (paternal - grandfather only) | 22, Daughter | 23, Son
**crf_rel5_1**	Condition<br>Please choose one of the following answers for each question:	radio	Mitochondrial disease	1, No | 2, Yes | 5, Unkown
**crf_rel5_2**		radio	Shortness	1, No | 2, Yes | 5, Unkown
**crf_rel5_3**		radio	Deafness	1, No | 2, Yes | 5, Unkown
**crf_rel5_4**		radio	Blindness	1, No | 2, Yes | 5, Unkown
**crf_rel5_5**		radio	Migraines	1, No | 2, Yes | 5, Unkown
**crf_rel5_6**		radio	Strokes	1, No | 2, Yes | 5, Unkown
**crf_rel5_7**		radio	Seizures	1, No | 2, Yes | 5, Unkown
**crf_rel5_8**		radio	Incoordination	1, No | 2, Yes | 5, Unkown
**crf_rel5_9**		radio	Ptosis	1, No | 2, Yes | 5, Unkown
**crf_rel5_10**		radio	Limb weakness	1, No | 2, Yes | 5, Unkown
**crf_rel5_11**		radio	Peripheral neuropathy	1, No | 2, Yes | 5, Unkown

**crf_rel5_12**		radio	Cardiomyopathy	1, No | 2, Yes | 5, Unkown
**crf_rel5_13**		radio	Gastrointestinal problems	1, No | 2, Yes | 5, Unkown
**crf_rel5_14**		radio	Diabetes mellitus	1, No | 2, Yes | 5, Unkown
**crf_rel5_15**		radio	Liver disease	1, No | 2, Yes | 5, Unkown
**crf_rel5_16**		radio	Kidney disease	1, No | 2, Yes | 5, Unkown
**crf_rel6_add**		yes/no	Do you want to add another relative?	
**crf_rel6**	Complete one page per relative, for as many 1st and 2nd degree relatives as possible	dropdown	Relationship:	1, Mother | 2, Father | 3, Sister | 4, Half-sister (maternal) | 5, Half-sister (paternal) | 6, Brother | 7, Half-brother (maternal) | 8, Half-brother (paternal) | 9, Grandmother (maternal) | 10, Grandmother (paternal) | 11, Grandfather (maternal) | 12, Grandfather (paternal) | 13, Aunt (maternal) | 14, Aunt (maternal - grandmother only) | 15, Aunt (maternal - grandfather only) | 16, Aunt (paternal) | 24, Aunt (paternal - grandmother only) | 17, Aunt (paternal - grandfather only) | 18, Uncle (maternal) | 25, Uncle (maternal - grandmother only) | 26, Uncle (maternal - grandfather only) | 19, Uncle (paternal) | 20, Uncle (paternal - grandmother only) | 21, Uncle (paternal - grandfather only) | 22, Daughter | 23, Son
**crf_rel6_1**	Condition<br>Please choose one of the following answers for each question:	radio	Mitochondrial disease	1, No | 2, Yes | 5, Unkown
**crf_rel6_2**		radio	Shortness	1, No | 2, Yes | 5, Unkown

**crf_rel6_3**		radio	Deafness	1, No | 2, Yes | 5, Unkown
**crf_rel6_4**		radio	Blindness	1, No | 2, Yes | 5, Unkown
**crf_rel6_5**		radio	Migraines	1, No | 2, Yes | 5, Unkown
**crf_rel6_6**		radio	Strokes	1, No | 2, Yes | 5, Unkown
**crf_rel6_7**		radio	Seizures	1, No | 2, Yes | 5, Unkown
**crf_rel6_8**		radio	Incoordination	1, No | 2, Yes | 5, Unkown
**crf_rel6_9**		radio	Ptosis	1, No | 2, Yes | 5, Unkown
**crf_rel6_10**		radio	Limb weakness	1, No | 2, Yes | 5, Unkown
**crf_rel6_11**		radio	Peripheral neuropathy	1, No | 2, Yes | 5, Unkown
**crf_rel6_12**		radio	Cardiomyopathy	1, No | 2, Yes | 5, Unkown
**crf_rel6_13**		radio	Gastrointestinal problems	1, No | 2, Yes | 5, Unkown
**crf_rel6_14**		radio	Diabetes mellitus	1, No | 2, Yes | 5, Unkown
**crf_rel6_15**		radio	Liver disease	1, No | 2, Yes | 5, Unkown
**crf_rel6_16**		radio	Kidney disease	1, No | 2, Yes | 5, Unkown
**crf_rel7_add**		yes/no	Do you want to add another relative?	
**crf_rel7**	Complete one page per relative, for as many 1st and 2nd degree relatives as possible	dropdown	Relationship:	1, Mother | 2, Father | 3, Sister | 4, Half-sister (maternal) | 5, Half-sister (paternal) | 6, Brother | 7, Half-brother (maternal) | 8, Half-brother (paternal) | 9, Grandmother (maternal) | 10, Grandmother (paternal) |

				11, Grandfather (maternal) | 12, Grandfather (paternal) | 13, Aunt (maternal) | 14, Aunt (maternal - grandmother only) | 15, Aunt (maternal - grandfather only) | 16, Aunt (paternal) | 24, Aunt (paternal - grandmother only) | 17, Aunt (paternal - grandfather only) | 18, Uncle (maternal) | 25, Uncle (maternal - grandmother only) | 26, Uncle (maternal - grandfather only) | 19, Uncle (paternal) | 20, Uncle (paternal - grandmother only) | 21, Uncle (paternal - grandfather only) | 22, Daughter | 23, Son
**crf_rel7_1**	Condition<br>Please choose one of the following answers for each question:	radio	Mitochondrial disease	1, No | 2, Yes | 5, Unkown
**crf_rel7_2**		radio	Shortness	1, No | 2, Yes | 5, Unkown
**crf_rel7_3**		radio	Deafness	1, No | 2, Yes | 5, Unkown
**crf_rel7_4**		radio	Blindness	1, No | 2, Yes | 5, Unkown
**crf_rel7_5**		radio	Migraines	1, No | 2, Yes | 5, Unkown
**crf_rel7_6**		radio	Strokes	1, No | 2, Yes | 5, Unkown
**crf_rel7_7**		radio	Seizures	1, No | 2, Yes | 5, Unkown
**crf_rel7_8**		radio	Incoordination	1, No | 2, Yes | 5, Unkown
**crf_rel7_9**		radio	Ptosis	1, No | 2, Yes | 5, Unkown
**crf_rel7_10**		radio	Limb weakness	1, No | 2, Yes | 5, Unkown

**crf_rel7_11**		radio	Peripheral neuropathy	1, No | 2, Yes | 5, Unkown
**crf_rel7_12**		radio	Cardiomyopathy	1, No | 2, Yes | 5, Unkown
**crf_rel7_13**		radio	Gastrointestinal problems	1, No | 2, Yes | 5, Unkown
**crf_rel7_14**		radio	Diabetes mellitus	1, No | 2, Yes | 5, Unkown
**crf_rel7_15**		radio	Liver disease	1, No | 2, Yes | 5, Unkown
**crf_rel7_16**		radio	Kidney disease	1, No | 2, Yes | 5, Unkown
**crf_rel8_add**		yes/no	Do you want to add another relative?	
**crf_rel8**	Complete one page per relative, for as many 1st and 2nd degree relatives as possible	dropdown	Relationship:	1, Mother | 2, Father | 3, Sister | 4, Half-sister (maternal) | 5, Half-sister (paternal) | 6, Brother | 7, Half-brother (maternal) | 8, Half-brother (paternal) | 9, Grandmother (maternal) | 10, Grandmother (paternal) | 11, Grandfather (maternal) | 12, Grandfather (paternal) | 13, Aunt (maternal) | 14, Aunt (maternal - grandmother only) | 15, Aunt (maternal - grandfather only) | 16, Aunt (paternal) | 24, Aunt (paternal - grandmother only) | 17, Aunt (paternal - grandfather only) | 18, Uncle (maternal) | 25, Uncle (maternal - grandmother only) | 26, Uncle (maternal - grandfather only) | 19, Uncle (paternal) | 20, Uncle (paternal - grandmother only) | 21, Uncle (paternal - grandfather only) | 22, Daughter | 23, Son
**crf_rel8_1**	Condition<br>Please choose one of the following answers for each question:	radio	Mitochondrial disease	1, No | 2, Yes | 5, Unkown

**crf_rel8_2**		radio	Shortness	1, No | 2, Yes | 5, Unkown
**crf_rel8_3**		radio	Deafness	1, No | 2, Yes | 5, Unkown
**crf_rel8_4**		radio	Blindness	1, No | 2, Yes | 5, Unkown
**crf_rel8_5**		radio	Migraines	1, No | 2, Yes | 5, Unkown
**crf_rel8_6**		radio	Strokes	1, No | 2, Yes | 5, Unkown
**crf_rel8_7**		radio	Seizures	1, No | 2, Yes | 5, Unkown
**crf_rel8_8**		radio	Incoordination	1, No | 2, Yes | 5, Unkown
**crf_rel8_9**		radio	Ptosis	1, No | 2, Yes | 5, Unkown
**crf_rel8_10**		radio	Limb weakness	1, No | 2, Yes | 5, Unkown
**crf_rel8_11**		radio	Peripheral neuropathy	1, No | 2, Yes | 5, Unkown
**crf_rel8_12**		radio	Cardiomyopathy	1, No | 2, Yes | 5, Unkown
**crf_rel8_13**		radio	Gastrointestinal problems	1, No | 2, Yes | 5, Unkown
**crf_rel8_14**		radio	Diabetes mellitus	1, No | 2, Yes | 5, Unkown
**crf_rel8_15**		radio	Liver disease	1, No | 2, Yes | 5, Unkown
**crf_rel8_16**		radio	Kidney disease	1, No | 2, Yes | 5, Unkown
**crf_rel9_add**		yes/no	Do you want to add another relative?	
**crf_rel9**	Complete one page per relative, for as many 1st	dropdown	Relationship:	1, Mother | 2, Father | 3, Sister | 4, Half-sister (maternal) | 5, Half-sister (paternal) |

	and 2nd degree relatives as possible			6, Brother | 7, Half-brother (maternal) | 8, Half-brother (paternal) | 9, Grandmother (maternal) | 10, Grandmother (paternal) | 11, Grandfather (maternal) | 12, Grandfather (paternal) | 13, Aunt (maternal) | 14, Aunt (maternal - grandmother only) | 15, Aunt (maternal - grandfather only) | 16, Aunt (paternal) | 24, Aunt (paternal - grandmother only) | 17, Aunt (paternal - grandfather only) | 18, Uncle (maternal) | 25, Uncle (maternal - grandmother only) | 26, Uncle (maternal - grandfather only) | 19, Uncle (paternal) | 20, Uncle (paternal - grandmother only) | 21, Uncle (paternal - grandfather only) | 22, Daughter | 23, Son
**crf_rel9_1**	Condition<br>Please choose one of the following answers for each question:<br>1, No | 2, Yes | 5, Unkown	radio	Mitochondrial disease	1, No | 2, Yes | 5, Unkown
**crf_rel9_2**		radio	Shortness	1, No | 2, Yes | 5, Unkown
**crf_rel9_3**		radio	Deafness	1, No | 2, Yes | 5, Unkown
**crf_rel9_4**		radio	Blindness	1, No | 2, Yes | 5, Unkown
**crf_rel9_5**		radio	Migraines	1, No | 2, Yes | 5, Unkown
**crf_rel9_6**		radio	Strokes	1, No | 2, Yes | 5, Unkown
**crf_rel9_7**		radio	Seizures	1, No | 2, Yes | 5, Unkown
**crf_rel9_8**		radio	Incoordination	1, No | 2, Yes | 5, Unkown
**crf_rel9_9**		radio	Ptosis	1, No | 2, Yes | 5, Unkown

**crf_rel9_10**		radio	Limb weakness	1, No | 2, Yes | 5, Unkown
**crf_rel9_11**		radio	Peripheral neuropathy	1, No | 2, Yes | 5, Unkown
**crf_rel9_12**		radio	Cardiomyopathy	1, No | 2, Yes | 5, Unkown
**crf_rel9_13**		radio	Gastrointestinal problems	1, No | 2, Yes | 5, Unkown
**crf_rel9_14**		radio	Diabetes mellitus	1, No | 2, Yes | 5, Unkown
**crf_rel9_15**		radio	Liver disease	1, No | 2, Yes | 5, Unkown
**crf_rel9_16**		radio	Kidney disease	1, No | 2, Yes | 5, Unkown
**crf_rel10_add**		yes/no	Do you want to add another relative?	
**crf_rel10**	Complete one page per relative, for as many 1st and 2nd degree relatives as possible	dropdown	Relationship:	1, Mother | 2, Father | 3, Sister | 4, Half-sister (maternal) | 5, Half-sister (paternal) | 6, Brother | 7, Half-brother (maternal) | 8, Half-brother (paternal) | 9, Grandmother (maternal) | 10, Grandmother (paternal) | 11, Grandfather (maternal) | 12, Grandfather (paternal) | 13, Aunt (maternal) | 14, Aunt (maternal - grandmother only) | 15, Aunt (maternal - grandfather only) | 16, Aunt (paternal) | 24, Aunt (paternal - grandmother only) | 17, Aunt (paternal - grandfather only) | 18, Uncle (maternal) | 25, Uncle (maternal - grandmother only) | 26, Uncle (maternal - grandfather only) | 19, Uncle (paternal) | 20, Uncle (paternal - grandmother only) | 21, Uncle (paternal - grandfather only) | 22, Daughter | 23, Son
**crf_rel10_1**	Condition	radio	Mitochondrial disease	1, No | 2, Yes | 5, Unkown

	Please choose one of the following answers for each question:<br><br>1, No | 2, Yes | 5, Unkown			
**crf_rel10_2**		radio	Shortness	1, No | 2, Yes | 5, Unkown
**crf_rel10_3**		radio	Deafness	1, No | 2, Yes | 5, Unkown
**crf_rel10_4**		radio	Blindness	1, No | 2, Yes | 5, Unkown
**crf_rel10_5**		radio	Migraines	1, No | 2, Yes | 5, Unkown
**crf_rel10_6**		radio	Strokes	1, No | 2, Yes | 5, Unkown
**crf_rel10_7**		radio	Seizures	1, No | 2, Yes | 5, Unkown
**crf_rel10_8**		radio	Incoordination	1, No | 2, Yes | 5, Unkown
**crf_rel10_9**		radio	Ptosis	1, No | 2, Yes | 5, Unkown
**crf_rel10_10**		radio	Limb weakness	1, No | 2, Yes | 5, Unkown
**crf_rel10_11**		radio	Peripheral neuropathy	1, No | 2, Yes | 5, Unkown
**crf_rel10_12**		radio	Cardiomyopathy	1, No | 2, Yes | 5, Unkown
**crf_rel10_13**		radio	Gastrointestinal problems	1, No | 2, Yes | 5, Unkown
**crf_rel10_14**		radio	Diabetes mellitus	1, No | 2, Yes | 5, Unkown
**crf_rel10_15**		radio	Liver disease	1, No | 2, Yes | 5, Unkown
**crf_rel10_16**		radio	Kidney disease	1, No | 2, Yes | 5, Unkown

**crf_rel11_add**		yes/no	Do you want to add another relative?	
**crf_rel11**	Complete one page per relative, for as many 1st and 2nd degree relatives as possible	dropdown	Relationship:	1, Mother | 2, Father | 3, Sister | 4, Half-sister (maternal) | 5, Half-sister (paternal) | 6, Brother | 7, Half-brother (maternal) | 8, Half-brother (paternal) | 9, Grandmother (maternal) | 10, Grandmother (paternal) | 11, Grandfather (maternal) | 12, Grandfather (paternal) | 13, Aunt (maternal) | 14, Aunt (maternal - grandmother only) | 15, Aunt (maternal - grandfather only) | 16, Aunt (paternal) | 24, Aunt (paternal - grandmother only) | 17, Aunt (paternal - grandfather only) | 18, Uncle (maternal) | 25, Uncle (maternal - grandmother only) | 26, Uncle (maternal - grandfather only) | 19, Uncle (paternal) | 20, Uncle (paternal - grandmother only) | 21, Uncle (paternal - grandfather only) | 22, Daughter | 23, Son
**crf_rel11_1**	Condition<br>Please choose one of the following answers for each question:<br>1, No | 2, Yes | 5, Unkown	radio	Mitochondrial disease	1, No | 2, Yes | 5, Unkown
**crf_rel11_2**		radio	Shortness	1, No | 2, Yes | 5, Unkown
**crf_rel11_3**		radio	Deafness	1, No | 2, Yes | 5, Unkown
**crf_rel11_4**		radio	Blindness	1, No | 2, Yes | 5, Unkown
**crf_rel11_5**		radio	Migraines	1, No | 2, Yes | 5, Unkown
**crf_rel11_6**		radio	Strokes	1, No | 2, Yes | 5, Unkown
**crf_rel11_7**		radio	Seizures	1, No | 2, Yes | 5, Unkown

**crf_rel11_8**		radio	Incoordination	1, No | 2, Yes | 5, Unkown
**crf_rel11_9**		radio	Ptosis	1, No | 2, Yes | 5, Unkown
**crf_rel11_10**		radio	Limb weakness	1, No | 2, Yes | 5, Unkown
**crf_rel11_11**		radio	Peripheral neuropathy	1, No | 2, Yes | 5, Unkown
**crf_rel11_12**		radio	Cardiomyopathy	1, No | 2, Yes | 5, Unkown
**crf_rel11_13**		radio	Gastrointestinal problems	1, No | 2, Yes | 5, Unkown
**crf_rel11_14**		radio	Diabetes mellitus	1, No | 2, Yes | 5, Unkown
**crf_rel11_15**		radio	Liver disease	1, No | 2, Yes | 5, Unkown
**crf_rel11_16**		radio	Kidney disease	1, No | 2, Yes | 5, Unkown
**crf_rel12_add**		yes/no	Do you want to add another relative?	
**crf_rel12**	Complete one page per relative, for as many 1st and 2nd degree relatives as possible	dropdown	Relationship:	1, Mother | 2, Father | 3, Sister | 4, Half-sister (maternal) | 5, Half-sister (paternal) | 6, Brother | 7, Half-brother (maternal) | 8, Half-brother (paternal) | 9, Grandmother (maternal) | 10, Grandmother (paternal) | 11, Grandfather (maternal) | 12, Grandfather (paternal) | 13, Aunt (maternal) | 14, Aunt (maternal - grandmother only) | 15, Aunt (maternal - grandfather only) | 16, Aunt (paternal) | 24, Aunt (paternal - grandmother only) | 17, Aunt (paternal - grandfather only) | 18, Uncle (maternal) | 25, Uncle (maternal - grandmother only) | 26, Uncle (maternal - grandfather only) | 19, Uncle (paternal) | 20, Uncle (paternal - grandmother only) |

				21, Uncle (paternal - grandfather only) | 22, Daughter | 23, Son
**crf_rel12_1**	Condition<br>Please choose one of the following answers for each question:<br>1, No | 2, Yes | 5, Unkown	radio	Mitochondrial disease	1, No | 2, Yes | 5, Unkown
**crf_rel12_2**		radio	Shortness	1, No | 2, Yes | 5, Unkown
**crf_rel12_3**		radio	Deafness	1, No | 2, Yes | 5, Unkown
**crf_rel12_4**		radio	Blindness	1, No | 2, Yes | 5, Unkown
**crf_rel12_5**		radio	Migraines	1, No | 2, Yes | 5, Unkown
**crf_rel12_6**		radio	Strokes	1, No | 2, Yes | 5, Unkown
**crf_rel12_7**		radio	Seizures	1, No | 2, Yes | 5, Unkown
**crf_rel12_8**		radio	Incoordination	1, No | 2, Yes | 5, Unkown
**crf_rel12_9**		radio	Ptosis	1, No | 2, Yes | 5, Unkown
**crf_rel12_10**		radio	Limb weakness	1, No | 2, Yes | 5, Unkown
**crf_rel12_11**		radio	Peripheral neuropathy	1, No | 2, Yes | 5, Unkown
**crf_rel12_12**		radio	Cardiomyopathy	1, No | 2, Yes | 5, Unkown
**crf_rel12_13**		radio	Gastrointestinal problems	1, No | 2, Yes | 5, Unkown
**crf_rel12_14**		radio	Diabetes mellitus	1, No | 2, Yes | 5, Unkown

**crf_rel12_15**		radio	Liver disease	1, No | 2, Yes | 5, Unkown
**crf_rel12_16**		radio	Kidney disease	1, No | 2, Yes | 5, Unkown
**crf_examdate**	Exams (complete at each visit)	text	Exam date	
**crf_nt1**		yes/no	Audiometry	
**crf_nt_1_yes**		radio	If Yes	1, Normal | 2, Abnormal
**crf_nt_1_yes_dtls**		radio	If Abnormal	1, Sensorineural hearing loss | 2, Conductive hearing loss | 3, High frequency hearing loss | 4, Low frequency hearing loss
**crf_nt2**		yes/no	BAER	
**crf_nt_2_yes**		radio	If Yes	1, Normal | 2, Abnormal
**crf_nt3**		yes/no	CSF drawn	
**crf_nt_3_yes**		text	If Yes, Lactate(mM)	
**crf_nt_3_yes2**		text	Pyruvate (mM)	
**crf_nt_3_yes3**		text	Protein(mg/dl)	
**crf_nt4**		yes/no	Brain CT	
**crf_nt_4_yes**		radio	If Yes	1, Normal | 2, Abnormal
**crf_nt_4_yes_dtls**		radio	If Abnormal	5, Global atrophy | 6, Basal ganglia lesions | 7, Basal ganglia calcifications | 8, Strokes/Stroke-like lesions | 9, White

				matter lesions | 10, Brainstem lesions | 11, Cerebellar atrophy | 12, Other abnormality
**crf_nt_brainctstrk**		text	If Yes,specify	
**crf_nt_brainctwm**		text	If Yes,specify	
**crf_nt_brainctoth**		text	If Yes,specify	
**crf_nt5**		yes/no	EEG	
**crf_nt_5_yes**		radio	If Yes	1, Normal | 2, Abnormal
**crf_nt_5_yes_dtls**		radio	If Abnormal	5, Epileptiform activity | 6, Diffuse slowing | 7, Focal slowing | 8, PLEDS | 9, Other abnormality
**crf_nt_eegoth**		text	If Yes,specify	
**crf_nt6**		yes/no	EMG	
**crf_nt_6_yes**		radio	If Yes	1, Normal | 2, Abnormal
**crf_nt_6_yes_dtls**		radio	If Abnormal	5, Spontaneous activity | 6, Myopathic motor units | 7, Neurogenic motor units | 8, Other abnormality
**crf_nt_emgoth**		text	If Yes,specify	
**crf_nt7**		yes/no	Exercise physiology testing	
**crf_vo2_wt**		text	Weight (kg)	
**crf_vo2**		text	VO2 (l/min)	

**crf_vco2**		text	VCO2 (l/min)	
**crf_ve**		text	VE (l/min)	
**crf_resprt**		text	Respiratory rate (per min)	
**crf_vo2_hr**		text	Heart rate (per minute)	
**crf_vo2_watt**		text	Wattage	
**crf_vo2_mxm**		radio	Is the test	1, Maximal | 2, Sub-maximal
**crf_nt8**		yes/no	Brain MRI:	
**crf_nt_8_yes**		radio	If Yes	1, Normal | 2, Abnormal
**crf_nt_8_yes_dtls**		radio	If Abnormal	5, Global atrophy | 6, Basal ganglia lesions | 7, Basal ganglia calcifications | 8, Strokes/Stroke-like lesions | 9, White matter lesions | 10, Brainstem lesions | 11, Cerebellar atrophy | 12, Other abnormality
**crf_brn_mri_strk**		text	If yes, specify	
**crf_brn_mri_wm**		radio	if yes, specify	1, Single | 2, Multiple | 3, Periventricular | 4, Diffuse | 5, Cystic | 6, Patchy | 7, Unknown
**crf_brn_mri_oth**		radio	Other abnormality	1, No | 2, Yes | 3, Unknown
**crf_brn_mri_oth_dtls**		text	If yes, specify	
**crf_nt9**		yes/no	Brain MRS	
**crf_nt_9_yes**		radio	If Yes	1, Normal | 2, Abnormal

**crf_nt_9_yes_dtls**		radio	If Abnormal	5, Elevated lactate in tissue | 6, Elevated lactate in CSF | 7, Elevated succinate in tissue | 8, Elevated succinate in CSF | 9, Decreased NAA/Cr ratio | 10, Other abnormality
**crf_brn_mrs_oth**		text	If yes, specify	
**crf_nt10**		yes/no	Nerve conduction study	
**crf_nt_10_yes**		radio	If Yes	1, Normal | 2, Abnormal
**crf_nt_10_yes_dtls**		radio	If Abnormal	5, Sensory involvement | 6, Motor involvement | 7, Demyelinating neuropathy | 8, Axonal neuropathy | 9, Other abnormality
**crf_nerv_cond_oth**		text	If yes, specify	
**crf_nt11**		yes/no	PET	
**crf_nt_11_yes**		radio	If Yes	1, Normal | 2, Abnormal
**crf_nt_11_yes_dtls**		radio	If Abnormal	5, Increased glucose metabolism | 6, Decreased glucose metabolism | 7, Other abnormality
**crf_pet_oth**		text	If yes, specify	
**crf_nt12**		yes/no	SPECT	
**crf_nt_12_yes**		radio	If Yes	1, Normal | 2, Abnormal
**crf_nt_12_yes_dtls**		radio	If Abnormal	5, Focal increase in blood flow | 6, Focal decrease in blood flow | 7, Global blood flow | 8, Other abnormality

**crf_spect_gbf**		radio	Global blood flow:	1, Normal | 2, Increased | 3, Decreased | 4, Unknown
**crf_spect_oth**		text	If yes, specify	
**crf_nt13**		yes/no	VER	
**crf_nt_13_yes**		radio	If Yes	1, Normal | 2, Abnormal
**crf_nt_13_yes_dtls**		radio	If Abnormal	5, Conduction delay/demyelination | 6, Low amplitude
**crf_nt14**	Cardiological Testing	yes/no	Echocardiogram	
**crf_nt_14_yes**		radio	If Yes	1, Normal | 2, Abnormal
**crf_nt_14_yes_dtls**		radio	If Abnormal	5, Low EF (<=35%) | 6, Dilated cardiomyopathy | 7, Hypertrophic cardiomyopathy | 8, Non-compaction cardiomyopathy | 9, Restrictive cardiomyopathy | 10, Other abnormality
**crf_ecg_oth**		text	If yes, specify	
**crf_nt15**		yes/no	EKG	
**crf_nt_15_yes**		radio	If Yes	1, Normal | 2, Abnormal
**crf_nt_15_yes_dtls**		radio	If Abnormal	5, Arrhythmia | 6, Pre-excitation syndrome | 7, Cardiac conduction block | 8, Left ventricular hypertrophy | 9, Other abnormality
**crf_ekg_arrhyth**		text	if Yes, specify	
**crf_ekg_other**		text	if Yes, specify	

**crf_nt16**		yes/no	Cardiac MRI	
**crf_nt_16_yes**		radio	If Yes	1, Normal | 2, Abnormal
**crf_nt_16_yes_dtls**		radio	If Abnormal	5, Low EF (<=35%) | 6, Dilated cardiomyopathy | 7, Hypertrophic cardiomyopathy | 8, Non-compaction cardiomyopathy | 9, Restrictive cardiomyopathy | 10, Other abnormality
**crf_geneticdate**	Genetic testing (complete at each visit)<br>Complete one set of pages per source of DNA obtained	text	Exam date	
**crf_gene_source**		radio	Source of DNA	1, Blood | 2, Muscle | 3, Urine | 4, Liver | 5, Fibroblasts | 6, Other, specify
**crf_gene_oth**		text	Specify:	
**crf_gene_sth_blt**		yes/no	Southern blot	
**crf_mtdlt**	Southern blot	radio	MtDNA deletion	1, No | 2, Single | 3, Multiple | 4, Unknown
**crf_mtdlt_perc**		text	% of normal:	
**crf_mtdpl**		radio	MtDNA depletion	1, No | 2, Yes | 3, Unknown
**crf_mtdpl_perc**		text	% of normal:	
**crf_gene_lr_pcr**	Long-range PCR	yes/no	Long-range PCR	
**crf_mtdlt_lr**		radio	MtDNA deletion	1, No | 2, Single | 3, Multiple | 4, Unknown
**crf_mtdlt_perc_lr**		text	% of normal:	

**crf_mtdpl_lr**	Real-time PCR	radio	MtDNA depletion	1, No | 2, Yes | 3, Unknown
**crf_mtdpl_perc_lr**		text	% of normal:	
**crf_gene_rflp**		yes/no	MtDNA tested by RFLP	
**crf_gene_rflp_yes**		radio	If Yes	1, Normal | 2, Abnormal
**crf_gene_rflp_mut**		text	If Abnormal, Mutation	
**crf_gene_rflp_perc**		text	Percentage	
**crf_gene_dtdna**		yes/no	MtDNA tested by RFLP	
**crf_gene_dtdna_yes**		radio	If Yes	1, Normal | 2, Abnormal
**crf_gene_dtdna_mut**		text	If Abnormal, Mutation	
**crf_gene_nuclr**		yes/no	MtDNA tested by RFLP	
**crf_gene_nuclr_yes**		radio	If Yes	1, Normal | 2, Abnormal
**crf_gene_nuclr_mut**		radio	If Abnormal, Mutation	1, Intergenomic communication defect | 2, Complex I subunit mutation | 3, Complex II subunit mutation | 4, Complex III subunit mutation | 5, Complex IV subunit mutation | 6, Complex V subunit mutation | 7, Complex I assembly factor mutation | 8, Complex II assembly factor mutation | 9, Complex III assembly factor mutation | 10, Complex IV assembly factor mutation | 11, Complex V assembly factor mutation | 12, Mitochondrial translation factor mutation | 13, Mitochondrial importation factor mutation | 14, Mitochondrial dynamics mutation | 15, OPA1 mutation | 16,

				Coenzyme Q10 biosynthesis gene mutation
**crf_ba_yes**		yes/no	Blood analysis	
**crf_ba_1**		text	Lactate (mM):	
**crf_ba_2**		text	Pyruvate (mM):	
**crf_ba_3**		text	CK (U/l):	
**crf_ba_4**		text	Thymidine (uM):	
**crf_ba_5**		text	Deoxyuridine (uM):	
**crf_ba_6**		text	Thymidine phosphorylase (nmol/h/mg-protein): ________________	
**crf_ba_7**		text	NH3 (umol/l):	
**crf_ba_plasma_amino**		yes/no	Plasma amino acids tested?	
**crf_ba_plasma_amino_normal_1**		radio	Alanine	1, Normal | 2, Abnormal
**crf_ba_plasma_amino_value_1**		text	value (umol/l)	
**crf_ba_plasma_amino_normal_2**		radio	Glycine	1, Normal | 2, Abnormal
**crf_ba_plasma_amino_value_2**		text	value (umol/l)	
**crf_ba_plasma_amino_normal_3**		radio	Lysine	1, Normal | 2, Abnormal
**crf_ba_plasma_amino_value_3**		text	value (umol/l)	

**crf_ba_plasma_amino_normal_4**		radio	Phenylalanine	1, Normal	2, Abnormal
**crf_ba_plasma_amino_value_4**		text	value (umol/l)	
**crf_ba_plasma_amino_normal_5**		radio	Proline	1, Normal	2, Abnormal
**crf_ba_plasma_amino_value_5**		text	value (umol/l)	
**crf_ba_plasma_amino_normal_6**		radio	Tyrosine	1, Normal	2, Abnormal
**crf_ba_plasma_amino_value_6**		text	value (umol/l)	
**crf_ba_plasma_carnitine_yes/no**		yes/no	Plasma carnitine tested?	
**crf_ba_plasma_carnitine_normal_1**		radio	Free carnitine	1, Normal	2, Abnormal
**crf_ba_plasma_carnitine_value_1**		text	value (umol/l)	
**crf_ba_plasma_carnitine_normal_2**		radio	Total carnitine	1, Normal	2, Abnormal
**crf_ba_plasma_carnitine_value_2**		text	value (umol/l)	
**crf_ba_plasma_carnitine_normal_3**		radio	Carnitine ester	1, Normal	2, Abnormal
**crf_ba_plasma_carnitine_value_3**		text	value (umol/l)	
**crf_ba_plasma_acylcarnitine_yes/no**		yes/no	Plasma acyl-carnitines tested?	
**crf_ba_plasma_acylcarnitine_normal_1**		radio	C3	1, Normal	2, Abnormal

**crf_ba_plasma_arclcarnitine_value_1**		text	value (umol/l)	
**crf_ba_plasma_acylcarnitine_normal_2**		radio	C4-OH	1, Normal | 2, Abnormal
**crf_ba_plasma_arclcarnitine_value_2**		text	value (umol/l)	
**crf_ba_plasma_acylcarnitine_normal_3**		radio	C5:1	1, Normal | 2, Abnormal
**crf_ba_plasma_arclcarnitine_value_3**		text	value (umol/l)	
**crf_ba_plasma_acylcarnitine_normal_4**		radio	C5-OH	1, Normal | 2, Abnormal
**crf_ba_plasma_arclcarnitine_value_4**		text	value (umol/l)	
**crf_ba_plasma_acylcarnitine_normal_5**		radio	C12	1, Normal | 2, Abnormal
**crf_ba_plasma_arclcarnitine_value_5**		text	value (umol/l)	
**crf_ba_plasma_acylcarnitine_normal_6**		radio	C14	1, Normal | 2, Abnormal
**crf_ba_plasma_arclcarnitine_value_6**		text	value (umol/l)	
**crf_ba_plasma_acylcarnitine_normal_7**		radio	C14:1	1, Normal | 2, Abnormal

**crf_ba_plasma_arclcarnitine_value_7**		text	value (umol/l)	
**crf_blu_natgel**		yes/no	Blue native gel	
**crf_blu_natgel_sourc**		radio	If Yes, source	1, Muscle | 2, Cell line | 3, Leukocytes | 4, buffy coat | 5, Platelets | 6, Liver | 7, Other
**crf_blu_natgel_1**		radio	Complex I activity	1, <25% activity | 2, >=25% and <35% activity | 3, >=35% and <45% activity | 4, >=45% and <=60% activity | 5, >60% activity | 6, Not tested/unknown
**crf_blu_natgel_2**		radio	Complex II activity	1, <25% activity | 2, >=25% and <35% activity | 3, >=35% and <45% activity | 4, >=45% and <=60% activity | 5, >60% activity | 6, Not tested/unknown
**crf_blu_natgel_3**		radio	Complex III activity	1, <25% activity | 2, >=25% and <35% activity | 3, >=35% and <45% activity | 4, >=45% and <=60% activity | 5, >60% activity | 6, Not tested/unknown
**crf_blu_natgel_4**		radio	Complex IV activity	1, <25% activity | 2, >=25% and <35% activity | 3, >=35% and <45% activity | 4, >=45% and <=60% activity | 5, >60% activity | 6, Not tested/unknown
**crf_blu_natgel_5**		radio	Complex V activity	1, <25% activity | 2, >=25% and <35% activity | 3, >=35% and <45% activity | 4, >=45% and <=60% activity | 5, >60% activity | 6, Not tested/unknown
**crf_electron**		truefalse	Electron microscopy on muscle tissue	
**crf_electron_normal**		radio	If Yes:	1, Normal | 2, Abnormal

**crf_electron_abn**		radio	If Abnormal:	1, Abnormal cristae | 2, Enlarged mitochondria | 3, Mitochondrial hyperplasia | 4, Paracrystalline inclusions | 5, Rarefaction of mitochondria
**crf_electron_mito**		radio	If Yes:	1, Oncocytic changes | 2, Not oncocytic | 3, Unknown
**crf_q10**		yes/no	HPLC for Coenzyme Q10:	
**crf_q10_source**		radio	If Yes, source:	1, Muscle | 2, Cell line | 3, Leukocytes | 4, buffy coat | 5, Platelets | 6, Liver | 7, Other
**crf_q10_lvl**		radio	level:	1, <25% | 2, >=25% and <35% | 3, >=35% and <45% | 4, >=45% and <=60% | 5, >60%
**crf_muscle**		yes/no	Muscle morphology:	
**crf_muscle_normal**		radio	If Yes:	1, Normal | 2, Abnormal
**crf_muscle_abn**		radio	If Abnormal:	1, Myopathic changes | 2, Neuropathic changes | 3, Ragged red fibers | 4, Ragged blue fibers | 5, COX-deficient fibers | 6, Strongly SDH+ vessels | 7, Excessive glycogen | 8, Excessive lipid | 9, Rarefaction of mitochondria
**crf_muscle_3**		radio	If Yes:	1, <=1% | 2, >1 and <=2% | 3, >2% | 4, Unknown
**crf_muscle_4**		radio	If Yes:	1, <=2% | 2, >2 and <=4% | 3, >4% | 4, Unknown
**crf_muscle_5**		radio	If Yes:	1, <=2% | 2, >2 and <=5% | 3, >5% | 4, Unknown

**crf_polarog**		yes/no	Polarography	
**crf_polarog_sourc**		radio	If Yes, source	1, Muscle | 2, Cell line | 3, Leukocytes | 4, buffy coat | 5, Platelets | 6, Liver | 7, Other
**crf_polarog_1**		radio	Complex I activity	1, <25% activity | 2, >=25% and <35% activity | 3, >=35% and <45% activity | 4, >=45% and <=60% activity | 5, >60% activity | 6, Not tested/unknown
**crf_polarog_2**		radio	Complex II activity	1, <25% activity | 2, >=25% and <35% activity | 3, >=35% and <45% activity | 4, >=45% and <=60% activity | 5, >60% activity | 6, Not tested/unknown
**crf_polarog_3**		radio	Complex IV activity	1, <25% activity | 2, >=25% and <35% activity | 3, >=35% and <45% activity | 4, >=45% and <=60% activity | 5, >60% activity | 6, Not tested/unknown
**crf_polarog_4**		radio	Oxidative phosphorylation	1, Present | 2, Absent | 3, Not tested/unkown
**crf_polarog_5**		radio	Loose coupling of oxidation and phosphorylation	1, Present | 2, Absent | 3, Not tested/unkown
**crf_radioiso**		yes/no	Radioisotope assay for PDC:	
**crf_radioiso_1**		radio	If Yes, activity	1, <25% activity | 2, >=25% and <35% activity | 3, >=35% and <45% activity | 4, >=45% and <=60% activity | 5, >60% activity
**crf_spectroph**		yes/no	Spectrophotometry	
**crf_spectroph_sourc**		radio	If Yes, source	1, Muscle | 2, Cell line | 3, Leukocytes | 4, buffy coat | 5, Platelets | 6, Liver | 7, Other

<table>
<tr><th></th><th></th><th></th><th></th><th></th></tr>
<tr><td>crf_spectroph_1</td><td></td><td>radio</td><td>Complex I activity</td><td>1, <25% activity | 2, >=25% and <35% activity | 3, >=35% and <45% activity | 4, >=45% and <=60% activity | 5, >60% activity | 6, Not tested/unknown</td></tr>
<tr><td>crf_spectroph_2</td><td></td><td>radio</td><td>Complex II activity</td><td>1, <25% activity | 2, >=25% and <35% activity | 3, >=35% and <45% activity | 4, >=45% and <=60% activity | 5, >60% activity | 6, Not tested/unknown</td></tr>
<tr><td>crf_spectroph_3</td><td></td><td>radio</td><td>Complex III activity</td><td>1, <25% activity | 2, >=25% and <35% activity | 3, >=35% and <45% activity | 4, >=45% and <=60% activity | 5, >60% activity | 6, Not tested/unknown</td></tr>
<tr><td>crf_spectroph_4</td><td></td><td>radio</td><td>Complex IV activity</td><td>1, <25% activity | 2, >=25% and <35% activity | 3, >=35% and <45% activity | 4, >=45% and <=60% activity | 5, >60% activity | 6, Not tested/unknown</td></tr>
<tr><td>crf_spectroph_5</td><td></td><td>radio</td><td>Complex V activity</td><td>1, <25% activity | 2, >=25% and <35% activity | 3, >=35% and <45% activity | 4, >=45% and <=60% activity | 5, >60% activity | 6, Not tested/unknown</td></tr>
<tr><td>crf_spectroph_6</td><td></td><td>radio</td><td>Thymidine phosphorylase activity</td><td>1, <25% activity | 2, >=25% and <35% activity | 3, >=35% and <45% activity | 4, >=45% and <=60% activity | 5, >60% activity | 6, Not tested/unknown</td></tr>
<tr><td>crf_urine</td><td></td><td>yes/no</td><td>Urine organic acids tested</td><td></td></tr>
<tr><td>crf_urine_1</td><td></td><td>radio</td><td>3-methyl glutaconic acid</td><td>1, Normal | 2, Abnormal</td></tr>
<tr><td>crf_urine_2</td><td></td><td>radio</td><td>Dicarboxylic acid</td><td>1, Normal | 2, Abnormal</td></tr>
<tr><td>crf_urine_3</td><td></td><td>radio</td><td>Ethylmalonate</td><td>1, Normal | 2, Abnormal</td></tr>
</table>

**crf_urine_4**		radio	Fumarate	1, Normal | 2, Abnormal
**crf_urine_5**		radio	Glutamate	1, Normal | 2, Abnormal
**crf_urine_6**		radio	Lactate	1, Normal | 2, Abnormal
**crf_urine_7**		radio	Malate	1, Normal | 2, Abnormal
**crf_urine_8**		radio	Pyruvate	1, Normal | 2, Abnormal
**crf_urine_9**		radio	Succinate	1, Normal | 2, Abnormal
**crf_urine_10**		radio	Other TCA cycle intermediates	1, Normal | 2, Abnormal
**crf_urine_1_vlu**		text	value (mmol/mol creatine):	
**crf_urine_2_vlu**		text	value (mmol/mol creatine):	
**crf_urine_3_vlu**		text	value (mmol/mol creatine):	
**crf_urine_4_vlu**		text	value (mmol/mol creatine):	
**crf_urine_5_vlu**		text	value (mmol/mol creatine):	
**crf_urine_6_vlu**		text	value (mmol/mol creatine):	
**crf_urine_7_vlu**		text	value (mmol/mol creatine):	
**crf_urine_8_vlu**		text	value (mmol/mol creatine):	
**crf_urine_9_vlu**		text	value (mmol/mol creatine):	
**crf_urine_10_vlu**		text	value (mmol/mol creatine):	

**crf_westernblt**		yes/no	Western blot	
**crf_crf_westernblt_sourc**		radio	If Yes, source	1, Muscle | 2, Cell line | 3, Leukocytes | 4, buffy coat | 5, Platelets | 6, Liver | 7, Other
**crf_crf_westernblt_1**		radio	Complex I activity	1, <25% activity | 2, >=25% and <35% activity | 3, >=35% and <45% activity | 4, >=45% and <=60% activity | 5, >60% activity | 6, Not tested/unknown
**crf_crf_westernblt_2**		radio	Complex II activity	1, <25% activity | 2, >=25% and <35% activity | 3, >=35% and <45% activity | 4, >=45% and <=60% activity | 5, >60% activity | 6, Not tested/unknown
**crf_crf_westernblt_3**		radio	Complex III activity	1, <25% activity | 2, >=25% and <35% activity | 3, >=35% and <45% activity | 4, >=45% and <=60% activity | 5, >60% activity | 6, Not tested/unknown
**crf_crf_westernblt_4**		radio	Complex IV activity	1, <25% activity | 2, >=25% and <35% activity | 3, >=35% and <45% activity | 4, >=45% and <=60% activity | 5, >60% activity | 6, Not tested/unknown
**crf_crf_westernblt_5**		radio	Complex V activity	1, <25% activity | 2, >=25% and <35% activity | 3, >=35% and <45% activity | 4, >=45% and <=60% activity | 5, >60% activity | 6, Not tested/unknown
**crf_31p**		yes/no	31P-NMR:	
**crf_31p_brain**		yes/no	If Yes, done on brain?	
**crf_31p_brn_region**		radio	If Yes, Region:	1, Frontal | 2, Parietal | 3, Occipital | 4, Temporal

**crf_31p_brn_pcr_vlu**		text	Phosphocreatine (PCr) Value	
**crf_31p_brn_pcr**		radio	Result:	1, Normal | 2, High | 3, Low
**crf_31p_brn_pi_vlu**		text	Inorganic phospate (Pi) Value	
**crf_31p_brn_pi**		radio	Result:	1, Normal | 2, High | 3, Low
**crf_31p_brn_pcrpi**		text	PCr/Pi ratio	
**crf_31p_brn_ph_vlu**		text	pH	
**crf_31p_brn_ph**		radio	Result:	1, Normal | 2, High | 3, Low
**crf_31p_brn_atp_vlu**		text	ATP	
**crf_31p_brn_atp**		radio	Result	1, Normal | 2, High | 3, Low
**crf_31p_brn_phos**		radio	Phosphorylation potential	1, Normal | 2, High | 3, Low
**crf_31p_brn_maxatp**		radio	% of maximum ATP biosynthesis	1, Normal | 2, High | 3, Low
**crf_31p_muscle**		yes/no	If Yes, done on muscle?	
**crf_31p_muscl_region**		radio	If Yes, Region:	1,Gastrocnemius | 2, Forearm
**crf_31p_muscl_pcr_vlu**		text	Phosphocreatine (PCr) Value	
**crf_31p_muscl_pcr**		radio	Result:	1, Normal | 2, High | 3, Low
**crf_31p_muscl_pi_vlu**		text	Inorganic phospate (Pi) Value	
**crf_31p_muscl_pi**		radio	Result:	1, Normal | 2, High | 3, Low

**crf_31p_muscl_pcrpi**		text	PCr/Pi ratio	
**crf_31p_muscl_ph_vlu**		text	pH	
**crf_31p_muscl_ph**		radio	Result:	1, Normal | 2, High | 3, Low
**crf_31p_muscl_atp_vlu**		text	ATP	
**crf_31p_muscl_atp**		radio	Result	1, Normal | 2, High | 3, Low
**crf_31p_muscl_phos**		radio	Phosphorylation potential	1, Normal | 2, High | 3, Low
**crf_31p_atp**		radio	% of maximum ATP biosynthesis	1, Normal | 2, High | 3, Low

1.2.3.5 Clinical Frailty Scale

RedCAP Form Name: clinical frailty scale Quantifies individual patient frailty

Variable / Field Name	Section Header	Field Type	Field Label	Variable / Field Name
**cfs**		radio	Clinical Frailty Scale	1,1. Very Fit--People who are robust, active, energetic and motivated. These people commonly exercise regularly. They are among the fittest for their age. | 2, 2. Well--People who have no active disease symptoms but are less fit than category 1. Often, they exercise or are very active occasionally, e.g. seasonally. | 3, 3. Managing Well--People whose medical problems are well controlled, but are not regularly active beyond routine walking. | 4,4. Vulnerable--While not dependent on others for daily help, often symptoms limit activities. A common complaint is being "slowed up", and/or being tired during the day. | 5,5. Mildly Frail--These people often have more evident slowing, and need help in high order IADLs(finances, transportation, heavy housework, medica-tions). Typically, mild frailty progressively impairs shopping and walking outside alone, meal preparation and housework. | 6,6. Moderately Frail--People need help with all outside activities and with keeping house. Inside, they often have problems with stairs and need help with bathing and might need minimal assistance (cuing, standby) with dressing. | 7,7. Severely Frail--Completely dependent for personal care, from whatever cause (physical or cognitive). Even so, they seem stable and not at high risk of dying (within 6 months). | 8,8.Very Severely Frail--Completely dependent, approaching the end of life. Typically, they could not recover even from a minor illness. | 9,9.Terminally Ill - Approaching the end of life. This category applies to people with a life expectancy <6 months, who are not otherwise evidently frail

**cfs_score**		calc	Clinical Frailty Scale Score:	[cfs]

1.2.3.6 Karnofsky Performance Scale

RedCAP Form Name: karnofsky performance status scale definitions rat

Description: Scale completed during the medical assessment. Classifies patients based on functional impairment.

Variable / Field Name	Section Header	Field Type	Field Label	Variable / Field Name
**karno_table**		text	The Karnofsky Performance Scale Index allows patients to be classified as to their functional impairment. This can be used to compare effectiveness of different therapies and to assess the prognosis in individual patients. The lower the Karnofsky score, the worse the survival for most serious illnesses.<br>KARNOFSKY PERFORMANCE STATUS SCALE DEFINITIONS RATING<br>Able to carry on normal activity and to work; no special care needed<br>Unable to work; able to live at home and care for most personal needs; varying amount of assistance needed<br>Unable to care for self; requires equivalent of institutional or hospital care; disease may be progressing rapidly.	
**karnofsky_100**		radio		100, 100 Normal no complaints; no evidence of disease. | 90, 90 Able to carry on normal activity; minor signs or symptoms of disease. | 80, 80 Normal activity with effort; some signs or symptoms of disease. | 70, 70 Cares for self; unable to carry on normal activity or to do active work.
**karnofsky_101**		radio		70, 70 Cares for self; unable to carry on normal activity or to do active work. | 60, 60 Requires occasional assistance, but is able to care for most of his personal needs. | 50, 50 Requires considerable assistance and frequent medical care.
**karnofsky_102**		radio		40, 40 Disabled; requires special care and assistance. | 30, 30 Severely disabled;

				hospital admission is indicated although death not imminent. | 20, 20 Very sick; hospital admission necessary; active supportive treatment necessary. | 10, 10 Moribund; fatal processes progressing rapidly. | 0, 0 Dead

1.2.4 Affect Ratings

RedCAP Form Name: Affect ratings

Description: Affect ratings completed a total of 14 times between day 1 and day 2

Variable / Field Name	Section Header	Field Type	Field Label	Variable / Field Name
**af_time**		text	Time:	
**af_strs**	Please pay attention to how you feel right now, at this moment, and answer the following five questions: Please choose one of the following answers for each question:	radio	How stressed do you feel right now?	1, Not at all 1 | 2, 2 | 3, Somewhat 3 | 4, 4 | 5, Moderately 5 | 6, 6 | 7, Very much 7 | 8, 8 | 9, Extremely 9
**af_angry**		radio	I feel angry	1, Not at all 1 | 2, 2 | 3, Somewhat 3 | 4, 4 | 5, Moderately 5 | 6, 6 | 7, Very much 7 | 8, 8 | 9, Extremely 9
**af_nerv**		radio	I feel nervous	1, Not at all 1 | 2, 2 | 3, Somewhat 3 | 4, 4 | 5, Moderately 5 | 6, 6 | 7, Very much 7 | 8, 8 | 9, Extremely 9
**af_relax**		radio	I feel relaxed	1, Not at all 1 | 2, 2 | 3, Somewhat 3 | 4, 4 | 5, Moderately 5 | 6, 6 | 7, Very much 7 | 8, 8 | 9, Extremely 9
**af_calm**		radio	I feel calm	1, Not at all 1 | 2, 2 | 3, Somewhat 3 | 4, 4 | 5, Moderately 5 | 6, 6 | 7, Very much 7 | 8, 8 | 9, Extremely 9
**af_energetic**		radio	I feel energetic	1, Not at all 1 | 2, 2 | 3, Somewhat 3 | 4, 4 | 5, Moderately 5 | 6, 6 | 7, Very much 7 | 8, 8 | 9, Extremely 9
**af_wornout**		radio	I feel worn out	1, Not at all 1 | 2, 2 | 3, Somewhat 3 | 4, 4 | 5, Moderately 5 | 6, 6 | 7, Very much 7 | 8, 8 | 9, Extremely 9

1.2.5 Day 1 Compliance

RedCAP Form Name: Compliance form day 1

Description: Questions to ensure participant compliance to researcher instructions

Variable / Field Name	Section Header	Field Type	Field Label	Variable / Field Name
**cfq_transportation_1**	Please fill out this form to the best of your ability and do not leave any question unanswered.	radio	How did you get to the study today?	1,Taxi | 2,Walking | 3, Other
**cfq_transportation_other_1**		text	Please explain:	
**cfq_1**		yes/no	Did you experience any symptoms of cold or flu in the past four weeks?	
**cfq_1_dtls**		text	Please explain	
**cfq_2**		yes/no	Did you get a normal night of sleep last night?	
**cfq_2_dtls**		text	Why?	
**cfq_3**		yes/no	Did you go to bed at the same time as you usually do?	
**cfq_3_dtls**		text	Why?	
**cfq_4**		yes/no	Did you consume any alcohol last night?	
**cfq_4_dtls**		text	How many glasses?	
**cfq_5**		yes/no	Did you engage in any strenuous activity in the last two days	

			(e.g., exercise)?	
**cfq_6**		radio	When was the last time you ate or drank something else than water?	1, Before 8pm last night | 2, Before 10pm last night | 3, Before midnight | 4, I had food/drink this morning
**cfq_6_dtls**		text	If so, what:	

1.3. Day 2

1.3.1 Data Collection Form Day 2

RedCAP Form Name: Data Collection Form Day 2

Description: Form for all Day 2 study procedures. Ensures all procedures are completed and all data and Day 2 details are recorded.

Variable / Field Name	Section Header	Field Type	Field Label	Variable / Field Name
**covid_temp_day2**	COVID SCREENING	text	Morning Temperature:	
**covid_cough_day2**		yes/no	Do you have a cough, shortness of breath, or a sore throat?	
**covid_travel_day2**		yes/no	Have you traveled outside of the tristate area in the past 14 days?	
**covid_contact_day2**		yes/no	Have you come into contact with anyone with a known or suspected case of Coronavirus in the past 14 days?	
**dcf_date_day2**	Day 2	text	Date	
**dcf_starttime_day2**		text	Time	
**dcf_weather_day2**		radio	External Weather:	1, Sunny | 2, Cloudy(but not wet) | 3, Rain/Snow

**dcf_extnl_temp_day2**		text	External Temperature:	
**dcf_strain_day2**		checkbox	STRAIN	1, Yes
**dcf_compliance_day2**		checkbox	Compliance Form Day 2	1, Yes
**dcf_urine_recvd**		checkbox	Receive urine container	1, Yes
**dcf_check_hotel_2**		checkbox	Check Hotel Questionnaires	1, Yes
**dcf_affect11**		checkbox	Affect 11	1, Yes
**dcf_saliva11**		checkbox	Saliva 11	1, Yes
**dcf_saliva9_time**		text	Time	
**dcf_mri_screening**		checkbox	MRI Screening Forms	1, Yes
**dcf_pregnancy_test**		radio	Pregnancy test:	1, Positive | 2, Negtive | 3, N/A (e.g., man)
**met_ree_d2**		text	Resting Energy Expenditure REE (kcal/day)	
**met_vo2_d2**		text	VO2 (mlO2/min)	
**respiratory_rate_rr_breaths_min**		text	Respiratory Rate RR (Breaths/min)	
**met_feo2_d2**		text	FeO2 (%)	
**met_tidal_d2**		text	Tidal volume (L)	
**met_ve_d2**		text	Minute Ventilation VE (L/min)	
**met_vo2_kg_d2**		calc	VO2 (mLO2/min/kg)	[met_vo2_d2]/([day1_arm_2][dcf_wgt]/2.205)

**dcf_metabolic_move_day2**		yes/no	Did the participant move during the analysis?	
**dcf_metabolic_move_notes_day1**		notes	If yes, additional box for comments	
**dcf_metabolic_air_day2**		yes/no	Did you notice air escaping from the mouthpiece?	
**dcf_metabolic_air_notes_day2**		notes		
**dcf_brkfst_start_day2**	Breakfast and Instrumentation	text	Breakfast start:	
**dcf_brkfst_end_day2**		text	Breakfast end:	
**dcf_brkfst_cmpl_day2**		radio	Participant:	1, Ate everything | 2, Not eaten completely
**dcf_brkfst_lo_day2**		file	Picture of Breakfast:	
**dcf_brkfst_cmpl_dtls_day2**		text	reason:	
**dcf_caffeine_2**		yes/no	Caffeine Consumed?	
**dcf_nptest**	Neuropsychological assessment	checkbox	Neuropsychological assessment, 75 mins	1, Yes
**dcf_qnaire2**	Questionnaire package	checkbox	Questionnaire package, 2 hours	1, Yes
**dcf_check_suicidality**		checkbox	Check Suicidality Measures (BDI and DSM Level 1)	1, Yes
**dcf_discuss_suicidality**		yes/no	Discussion with Study Coordinator regarding suicidality measures?	

**dcf_notes_suicidality**		notes	Notes regarding discussion	
**dcf_cssrs2_1**		yes/no	1. In the last month, have you wished you were dead or wished you could go to sleep and not wake up?	
**dcf_cssrs2_2**		yes/no	2. In the last month, have you actually had any thoughts about killing yourself?	
**dcf_cssrs2_3**		yes/no	3. Have you been thinking about how you might do this?	
**dcf_cssrs2_4**		yes/no	4. Have you had these thoughts and had some intention of acting on them? If yes: HIGH RISK	
**dcf_cssrs2_5**		yes/no	5. Have you started to work out or worked out the details of how to kill yourself? Did you intend to carry out this plan? If yes: HIGH RISK	
**dcf_cssrs2_6**		yes/no	6. Have you done anything, started to anything, or prepared to do anything to end your life? If yes: HIGH RISK	
**dcf_psy_eval_upld**		file	Psychological Evaluation	
**dcf_lunch_day2**	Lunch	checkbox	Lunch	1, Yes
**dcf_scanner_noise**	MRI Session	checkbox	Play scanner noises	1, Yes
**dcf_arm_warp**		checkbox	Arm wrap shown	1, Yes
**dcf_nback**		checkbox	Introduce n-back task	1, Yes

**dcf_explain_dti**		checkbox	Explain DTI vibrations	1, Yes
**dcf_affect12**		checkbox	Affect 12	1, Yes
**dcf_saliva12**		checkbox	Saliva 12	1, Yes
**dcf_saliva12_time**		text	Time	
**dcf_before_changing**		checkbox	Before changing—remind to remove all metals (bras, hair ties, jewelry)	1, Yes
**dcf_metal_move**		checkbox	Screen for all metals and move in scanner	1, Yes
**dcf_mri_nervousness**		radio	Please rate your nervousness about the scan from 0 (not nervous at all) to 10 (extremely nervous): 0-10	0, 0 not nervous at all | 1, 1 | 2, 2 | 3, 3 | 4, 4 | 5, 5 | 6, 6 | 7, 7 | 8, 8 | 9, 9 | 10, 10 extremely nervous
**dcf_pulse_oximt**		checkbox	Instrumentation with pulse oximeter (heart rate)	1, Yes
**dcf_hr_signal**		yes/no	Signal OK?	
**dcf_tone_volume**		radio	Please rate the volume of these tones	1, 0 not loud | 2, 1 | 3, 2 | 4, 3 | 5, 4 | 6, 5 | 7, 6 | 8, 7 | 9, 8 | 10, 9 | 11, 10 extremely loud
**dcf_mri_starttime**		text	Start time of the MRI scan:	
**dcf_mri_scout**		checkbox	Scout (0:14 mins)	1, Yes
**dcf_mri_scout_nts**		notes	Note	
**dcf_mri_t2scan**		checkbox	T2 scan (8:28 min)	1, Yes
**dcf_mri_t2scan_nts**		notes	Note	

**dcf_mri_t1scan**		checkbox	T1 scan (8:28 min)	1, Yes
**dcf_mri_t1_scan_nts**		notes	Note	
**dcf_mri_discomfort_t1**		radio	Please rate your physical discomfort right now from 0 (completely comfortable) to 10 (extremely uncomfortable): 0-10	0, 0 completely comfortable | 1, 1 | 2, 2 | 3, 3 | 4, 4 | 5, 5 | 6, 6 | 7, 7 | 8, 8 | 9, 9 | 10, 10 extremely uncomfortable
**dcf_mri_multisensory_**		checkbox	Multisensory Stim (5 min)	1, Yes
**dcf_mri_multisensory_nts**		notes	Note	
**dcf_mri_discomfort_multisen**		radio	Please rate your physical discomfort right now from 0 (completely comfortable) to 10 (extremely uncomfortable): 0-10	0, 0 completely comfortable | 1, 1 | 2, 2 | 3, 3 | 4, 4 | 5, 5 | 6, 6 | 7, 7 | 8, 8 | 9, 9 | 10, 10 extremely uncomfortable
**dcf_mri_restingstate**		checkbox	Resting state (10:51 min)	1, Yes
**dcf_mri_restingstate_nts**		notes	Note	
**dcf_mri_discomfort_resting**		radio	Please rate your physical discomfort right now from 0 (completely comfortable) to 10 (extremely uncomfortable): 0-10	0, 0 completely comfortable | 1, 1 | 2, 2 | 3, 3 | 4, 4 | 5, 5 | 6, 6 | 7, 7 | 8, 8 | 9, 9 | 10, 10 extremely uncomfortable
**dcf_affect13**		checkbox	Affect 13	1, Yes
**dcf_saliva13**		checkbox	Saliva 13	1, Yes
**dcf_saliva13_time**		text	Time	
**dcf_mri_nbackrun1**		checkbox	N-back task Run 1 (6 min)	1, Yes
**dcf_mri_nbackrun1_nts**		notes	Note	

**dcf_mri_discomfort_nback1**		radio	Please rate your physical discomfort right now from 0 (completely comfortable) to 10 (extremely uncomfortable): 0-10	0, 0 completely comfortable | 1, 1 | 2, 2 | 3, 3 | 4, 4 | 5, 5 | 6, 6 | 7, 7 | 8, 8 | 9, 9 | 10, 10 extremely uncomfortable
**dcf_mri_nbackrun2**		checkbox	N-back task Run 2 (6 min)	1, Yes
**dcf_mri_nbackrun2_nts**		notes	Note	
**dcf_mri_discomfort_nback2**		radio	Please rate your physical discomfort right now from 0 (completely comfortable) to 10 (extremely uncomfortable): 0-10	0, 0 completely comfortable | 1, 1 | 2, 2 | 3, 3 | 4, 4 | 5, 5 | 6, 6 | 7, 7 | 8, 8 | 9, 9 | 10, 10 extremely uncomfortable
**dcf_mri_speechprep**		checkbox	Speech preparation (6 min)	1, Yes
**dcf_mri_speechprep_nts**		notes	Note	
**dcf_mri_discomfort_speechprep**		radio	Please rate your physical discomfort right now from 0 (completely comfortable) to 10 (extremely uncomfortable): 0-10	0, 0 completely comfortable | 1, 1 | 2, 2 | 3, 3 | 4, 4 | 5, 5 | 6, 6 | 7, 7 | 8, 8 | 9, 9 | 10, 10 extremely uncomfortable
**dcf_mri_coldpress**		checkbox	Cold pressor (6 min)	1, Yes
**dcf_mri_coldpress_nts**		notes	Note	
**dcf_cold_pressor_painful_2**		radio	Please rate how painful the task was from 0 (not painful at all) to 10 (extremely painful):	0, 0 not painful at all | 1, 1 | 2, 2 | 3, 3 | 4, 4 | 5, 5 | 6, 6 | 7, 7 | 8, 8 | 9, 9 | 10, 10 extremely painful
**dcf_mri_discomfort_coldpres**		radio	Please rate your physical discomfort right now from 0 (completely comfortable) to 10 (extremely uncomfortable): 0-10	0, 0 completely comfortable | 1, 1 | 2, 2 | 3, 3 | 4, 4 | 5, 5 | 6, 6 | 7, 7 | 8, 8 | 9, 9 | 10, 10 extremely uncomfortable
**dcf_mri_dti**		checkbox	DTI seg(1-2) (6:27 min)	1, Yes

**dcf_saliva14**		checkbox	Saliva 14	1, Yes
**dcf_affect14**		checkbox	Affect 14	1, Yes
**dcf_saliva14_time**		text	Time	
**dcf_mri_dti2**		checkbox	DTI seg(3-6) (14:37 min)	1, yes
**dcf_mri_dti_nts**		notes	Note	
**dcf_mri_discomfort_dti**		radio	Please rate your physical discomfort right now from 0 (completely comfortable) to 10 (extremely uncomfortable): 0-10	0, 0 completely comfortable | 1, 1 | 2, 2 | 3, 3 | 4, 4 | 5, 5 | 6, 6 | 7, 7 | 8, 8 | 9, 9 | 10, 10 extremely uncomfortable
**dcf_deinstrumnt_day2**		checkbox	De-instrumentation	1, Yes
**dcf_rtn_ph15**		checkbox	Return to PH1540	1, Yes
**dcf_mri_endtime**		text	Time leaving the MRI unit:	
**dcf_debrief_sleep**	Debriefing	yes/no	Did you fall asleep at any point during the MRI?	
**dcf_debrief_sleepwhen**		checkbox	If yes, when?	1, Scout | 2, T2 Scan | 3, T1 Scan | 4, Multisensory | 5, Resting | 6, N-back run 1 | 7, N-back Run 2 | 8, Speech prep | 9, Cold Pressor | 10, DTI 1 | 11, DTI 2
**dcf_debrief**		checkbox	Study debriefing	1, Yes
**dcf_cp_compare**		radio	How did the Day 2 cold pressor compare to day 1:	1, More painful | 2, Less painful | 3, The same
**dcf_speech_compare**		radio	How did the Day 2 speech task compare to day 1's speech task?:	1, More stressful | 2, less stressful | 3, the same

**dcf_speech_nervouscompare**		radio	How did the day 2 speech task compare to day 1's speech task in terms of nervousness?	1, More nervous | 2, less nervous | 3, the same
**dcf_familiar_speech**		yes/no	Were you familiar with the speech task before yesterday?	
**dcf_believe_speech_day1**		radio	To what extent did you believe the speech task on day 1?	1, Believed all parts | 2, Believed some parts | 3, believed no parts
**dcf_believe_speech_day2**		radio	To what extent did you believe the speech task on day 2?	1, Believed all parts | 2, Believed some parts | 3, believed no parts
**dcf_adv_event**		yes/no	Did you experience any adverse events during this study?	
**dcf_adv_event_yes**		text	If yes, please explain	
**dcf_return1540**		checkbox	Return to PH1540	1, yes
**dcf_time_leave_mri**		text	Time leaving the MRI facility:	
**dcf_saliva_instuct**	Instructions for home saliva collection	checkbox	Instructions for saliva	1, Yes
**dcf_saliva_demo**		checkbox	Demonstration	1, Yes
**dcf_fecal_instuct**		checkbox	Instructions for fecal collection	1, Yes
**dcf_fecal_demo**		checkbox	Demonstration	1, Yes
**dcf_log_pckg**		checkbox	Logbook and home iPad	1, Yes
**dcf_reimburse**		checkbox	Reimbursement Form	1, Yes

**dcf_paycard**		checkbox	Paycard Receipt Signed	1, Yes
**dcf_general_notes**		notes	General Notes about Visit	
**dcf_visit_summary**		file	Visit Summary	

1.3.2 Day 2 Compliance

RedCAP Form Name: Compliance form day 2

Description: Questions to ensure participant compliance to research instructions

Variable / Field Name	Section Header	Field Type	Field Label	Variable / Field Name
**cfq_transportation_2**	Please fill out this form to the best of your ability and do not leave any question unanswered.	radio	How did you get to the study today?	1, Taxi | 2, Walking | 3, Other
**cfq_transportation_other_2**		text	Please explain:	
**cfq2_1**		yes/no	Did you go out after participating in the first of the study?	
**cfq2_1_dtls**		text	Length of time:	
**cfq2_2**		yes/no	Did you experience anything unusual after your first day in the study?	
**cfq2_2_dtls**		text	What?	
**cfq2_3**		radio	How many times did you miss the urine collection over the 12-hour period?	1, none | 2, 1-3 times | 3, more than 4 times
**cfq2_4**		yes/no	Did you experience anything unusual this morning?	
**cfq2_4_dtls**		text	What?	

**cfq2_5**		yes/no	Did you have any problems getting out of bed this morning?	
**cfq2_5_dtls**		text	why?	

1.3.3 Neuropsychological Grading

RedCAP Form Name: Neuropsych

Description: Neuropsychological scored results

Variable / Field Name	Section Header	Field Type	Field Label
**neuro_flag**		yes/no	Flag this data?
**neuro_flag_notes**		notes	Why was this data flagged?
**st_free_corsort_raw**	D-KEFS Sorting Test: Summary of Scores<br>Primary Measures	text	Confirmed Correct Sorts Raw Score
**st_free_corsort_scaled**		text	Confirmed Correct Sorts Scaled Score
**st_free_descrp_raw**		text	Free Sorting Description Score Raw Score:
**st_free_descrp_scaled**		text	Free Sorting Description Score Scaled Score:
**st_cor_cardset1_scaled**		text	Confirmed Correct Sorts: Card Set 1 Scaled Score:
**st_cor_cardset2_scaled**		text	Confirmed Correct Sorts: Card Set 2 Scaled Score:
**st_cor_verb_scaled**		text	Confirmed Correct Verbal Sorts Scaled Score:
**st_cor_percep_scaled**		text	Confirmed Correct perceptual Sorts Scaled Score:
**st_rept_scaled**		text	Repeated Sorts Scaled Score:

**st_free_cardset1_scaled**		text	Free Sorting Description Score: Card Set 1 Scaled Score:
**st_free_cardset2_scaled**		text	Free Sorting Description Score: Card Set 2 Scaled Score:
**vf_c1_lf_cor_raw**	D-KEFS Verbal Fluency Test: Summary of Scores<br>Primary Measures	text	Condition 1: Letter Fluency Total Correct Raw Score
**vf_c1_lf_cor_scaled**		text	Condition 1: Letter Fluency Total Correct Scaled Score
**vf_c1_cf_cor_raw**		text	Condition 1: Category Fluency Total Correct Raw Score
**vf_c1_cf_cor_scaled**		text	Condition 1: Category Fluency Total Correct Scaled Score
**vf_c1_cs_cor_raw**		text	Condition 1: Category Switching Total Correct Raw Score
**vf_c1_cs_cor_scaled**		text	Condition 1: Category Switching Total Correct Scaled Score
**vf_c1_cs_tsa_raw**		text	Condition 1: Category Switching Total Switching Accuracy Raw Score
**vf_c1_cs_tsa_scaled**		text	Condition 1: Category Switching Total Switching Accuracy Scaled Score
**vf_lf_cf_contrst_scaled**	Primary Contrast Measures	text	Letter Fluency v.s. Category Fluency Contrast Scaled Score
**vf_cs_cf_contrst_scaled**		text	Category Switching v.s. Category Fluency Contrast Scaled Score
**vf_first_cor_scaled**	Optional Measures: Conditions 1-3 combined	text	First interval Total Correct Scaled Score

**vf_secd_cor_scaled**		text	Second interval Total Correct Scaled Score
**vf_third_cor_scaled**		text	Third interval Total Correct Scaled Score
**vf_fourth_cor_scaled**		text	Fourth interval Total Correct Scaled Score
**vf_set_loss**		text	Set-loss Errors Scaled Score
**vf_repet_error**		text	Repetition Errors Scaled Score
**vf_perc_set_loss**		text	Percent Set-loss Errors Scaled Score
**vf_perc_repet_error**		text	Percent Repetition Errors Scaled Score
**vf_perc_swit_accur**		text	Catogary Switching: Percent Switching Accuracy(Condition 3 only) Scaled Score
**tm_ns_raw**	D-KEFS Trial Making Test: Summary of Scores<br>Primary Measures: Completion Times	text	Condition2: Number Sequencing Raw Score
**tm_ns_scaled**		text	Condition2: Number Sequencing Scaled Score
**tm_ls_raw**		text	Condition3: Letter Sequencing Raw Score
**tm_ls_scaled**		text	Condition3: Letter Sequencing Scaled Score
**tm_nls_raw**		text	Condition4: Number-Letter Switching Raw Score
**tm_nls_scaled**		text	Condition4: Number-Letter Switching Scaled Score
**tm_ns_ls_scaled**	Primary Combined Measure: Completion Times	text	Combined Number Sequencing + Letter Sequencing Composite Scaled Score

**tm_nls_nsls_contras_scaled**	Primary Contrast Measure: Completion Times	text	Number-Letter Switching v.s. Combined Number + Lettere Sequencing Contrast Scaled Score.
**cwi_cn_raw**	D-KEFS Color-Word Interference Test: Summary of Scores<br>Primary Measures: Completion Times	text	Condition1: Color Naming Raw Score
**cwi_cn_scaled**		text	Condition1: Color Naming Scaled Score
**cwi_wr_raw**		text	Condition2: Word Reading Raw Score
**cwi_wr_scaled**		text	Condition2: Word Reading Scaled Score
**cwi_inhib_raw**		text	Condition3: Inhibition Raw Score
**cwi_inhib_scaled**		text	Condition3: Inhibition Scaled Score
**cwi_inhib_swit_raw**		text	Condition4: Inhibition/Switching Raw Score
**cwi_inhib_swit_scaled**		text	Condition4: Inhibition/Switching Scaled Score
**cwi_cn_rd_scaled**	Primary Combined Measure: Completion Times	text	Combined Naming + Reading Composite Scaled Score
**cwi_inh_cn_scaled**	Primary Contrast Measure: Completion Times	text	Inhibition vs. Color Naming Contrast Scaled Score
**wasi_vocab_raw**	WASI-II	text	Vocabulary raw score
**wasi_vocab_tscore**		text	Vocabulary T Score
**wasi_mr_raw**		text	Matrix Reasoning raw score
**wasi_mr_tscore**		text	Metrix Reasoning T Score
**wasi_full_sum_t**		text	Full Scaled-2 Sum of T scores

**wasi_full_sum_c**		text	Full Scaled-2 Composite Score
**topf_raw_score**	TOPF	text	Total Raw Score
**topf_standrd_score**		text	Standard Score
**rbans_ll_total**	rbans	text	list learning total score
**rbans_ll_scaled**		text	list learning scaled score
**rbans_lo_total**		text	line orientation total score
**rbans_lo_scaled**		text	line orientation percentile
**rbans_pn_total**		text	picture naming total score
**rbans_pn_scaled**		text	picture naming percentile
**rbans_cd_total**		text	coding total score
**rbans_cd_scaled**		text	coding naming scaled score
**rbans_lrcl_raw**		text	list recall raw score
**rbans_lrcl_perc**		text	list recall scaled percent
**rbans_lrcg_raw**		text	list recognition raw score
**rbans_lrcg_perc**		text	list recognition percent
**nab_sdgf_raw**	nab	text	Screening Digital Forward Raw Score

**nab_sdgf_t**		text	Screening Digital Forward T Score
**nab_sdgf_perc**		text	Screening Digital Forward Percentile
**nab_sdgf_spn_raw**		text	Screening Digital Forward Longest Span Raw Score
**nab_sdgf_spn_perc**		text	Screening Digital Forward Longest Span Percentile
**nab_sdgb_raw**		text	Screening Digital Backward Raw Score
**nab_sdgb_t**		text	Screening Digital Backward T Score
**nab_sdgb_perc**		text	Screening Digital Backward Percentile
**nab_sdgb_spn_raw**		text	Screening Digital Backward Longest Span Raw Score
**nab_sdgb_spn_perc**		text	Screening Digital Backward Longest Span Percentile
**nab_snl_spd_raw**		text	Screening Numbers & Letters Part A Speed Raw Score
**nab_snl_spd_t**		text	Screening Numbers & Letters Part A Speed T Score
**nab_snl_spd_perc**		text	Screening Numbers & Letters Part A Speed Percentile
**nab_snl_err_raw**		text	Screening Numbers & Letters Part A Error Raw Score
**nab_snl_err_t**		text	Screening Numbers & Letters Part A Error T Score
**nab_snl_err_perc**		text	Screening Numbers & Letters Part A Error Percentile
**nab_snl_eff_raw**		text	Screening Numbers & Letters Part A Efficiency Raw Score
**nab_snl_eff_t**		text	Screening Numbers & Letters Part A Efficiency T Score

**nab_snl_eff_perc**		text	Screening Numbers & Letters Part A Efficiency Percentile
**nab_snl_effb_raw**		text	Screening Numbers & Letters Part B Efficiency Raw Score
**nab_snl_effb_t**		text	Screening Numbers & Letters Part B Efficiency T Score
**nab_snl_effb_perc**		text	Screening Numbers & Letters Part B Efficiency Percentile
**nab_shl_irg_raw**		text	Screening Shape Learning Immediate Recognition Raw Score
**nab_shl_irg_t**		text	Screening Shape Learning Immediate Recognition T Score
**nab_shl_irg_perc**		text	Screening Shape Learning Immediate Recognition Percentile
**nab_shl_drg_raw**		text	Screening Shape Learning Delayed Recognition Raw Score
**nab_shl_drg_perc**		text	Screening Shape Learning Delayed Recognition Percentile
**nab_shl_prt_raw**		text	Screening Shape Learning Percent Retetion Raw Score
**nab_shl_prt_t**		text	Screening Shape Learning Percent Retetion T Score
**nab_shl_prt_perc**		text	Screening Shape Learning Percent Retetion Percentile
**neuro_behav**		radio	Behavioral Observations:
**neuro_notes**		notes	Notes:

1.3.4 Physiological Session

RedCAP Form Name: physio

Description: Physiological session data

Variable / Field Name	Section Header	Field Type	Field Label	Variable / Field Name
**blood_centrifuge_log**		file	Blood Centrifuge Log	
**physio_date**	Session Information	text	Session Date	
**physio_tech**		dropdown	Technician:	33, Vincenzo Lauriola | 34, Jacob Thomas| 10, Other
**physio_tech_other**		text	If other, please specify	
**physio_sestatnotes**		notes	Session Notes:	
**physio_sestat**		dropdown	Session Status	1, Complete | 2, Incomplete PHYS (physical health problem) | 3, Incomplete: TECH (technical reason) | 4, Session not run | 5, Other
**physio_sestat_period**		text	If incomplete, which period was session terminated:	
**physio_sestatrsn**		dropdown	Session Status Reason	1, P-Phys Successful | 2, Hi/Lo BP | 3, Physical Discomfort | 4, Physical Incompetence | 5, Medical Condition | 6,Equipment: ECG/Other | 7, Equipment: Finometer | 8, Equipment: Other | 9, Other
**physio_compltns**		dropdown	PhysData Completeness Codes	1, None | 2, No BP | 3, No rsp | 4, No ECG | 5, All | 6, Other
**physio_scoredate**	Score Log	text	Scoring Date	
**physio_scorer**		dropdown	Scorer:	

**physio_scorer_other**		text	If other, please specify	
**physio_bpwave_base**	Baseline	dropdown	BP Waveform Quality	1, Clean signal | 2, Noise | 3, Missing data | 4,Period not run
**physio_bpreason_base**		dropdown	BP Quality Reason	1, Clean signal | 2, Interference | 3, PP movement | 4, Loss of signal | 5, Missing Data | 6, Period not run
**physio_bpphysio_base**		dropdown	BP Physiology	1, Normal | 2, PVCs | 3, PACs | 4, Other Non-Sinus | 5, Missing Data | 6, Period not run
**physio_bpbi_base**		radio	BP BI	1, Whole | 2, Partial | 3, None| 4, Missing Data | 5, Period not run
**physio_ecgwave_base**		dropdown	ECG Waveform Quality	1, Clean signal | 2, Noise | 3, Missing data | 4,Period not run
**physio_ecgreason_base**		dropdown	ECG Quality Reason	1, Clean signal | 2, Interference | 3, PP movement | 4, Loss of signal | 5, Missing Data | 6, Period not run
**physio_ecgphysio_base**		dropdown	ECG Physiology	1, Normal | 2, PVCs | 3, PACs | 4, Other Non-Sinus | 5, Missing Data | 6, Period not run
**physio_ecgbi_base**		radio	ECG BI	1, Whole | 2, Partial | 3, None| 4, Missing Data | 5, Period not run
**physio_rspwave_base**		dropdown	RSP Waveform Quality	1, Clean signal | 2, Noise | 3, Period not run | 4,Missing data
**physio_rspreason_base**		dropdown	RSP Quality Reason	1, Normal | 2, Interference | 3, PP movement | 4, Loss of signal | 5, Missing Data | 6, Period not run
**physio_notes_base**		notes	Notes:	
**physio_bpwave_pre**	-5 min	dropdown	BP Waveform Quality	1, Clean signal | 2, Noise | 3, Missing data | 4,Period not run
**physio_bpreason_pre**		dropdown	BP Quality Reason	1, Clean signal | 2, Interference | 3, PP movement | 4, Loss of signal | 5, Missing Data | 6, Period not run

**physio_bpphysio_pre**		dropdown	BP Physiology	1, Normal | 2, PVCs | 3, PACs | 4, Other Non-Sinus | 5, Missing Data | 6, Period not run
**physio_bpbi_pre**		radio	BP BI	1, Whole | 2, Partial | 3, None| 4, Missing Data | 5, Period not run
**physio_ecgwave_pre**		dropdown	ECG Waveform Quality	1, Clean signal | 2, Noise | 3, Missing data | 4,Period not run
**physio_ecgreason_pre**		dropdown	ECG Quality Reason	1, Clean signal | 2, Interference | 3, PP movement | 4, Loss of signal | 5, Missing Data | 6, Period not run
**physio_ecgphysio_pre**		dropdown	ECG Physiology	1, Normal | 2, PVCs | 3, PACs | 4, Other Non-Sinus | 5, Missing Data | 6, Period not run
**physio_ecgbi_pre**		radio	ECG BI	1, Whole | 2, Partial | 3, None| 4, Missing Data | 5, Period not run
**physio_rspwave_pre**		dropdown	RSP Waveform Quality	1, Clean signal | 2, Noise | 3, Period not run | 4,Missing data
**physio_rspreason_pre**		dropdown	RSP Quality Reason	1, Normal | 2, Interference | 3, PP movement | 4, Loss of signal | 5, Missing Data | 6, Period not run
**physio_notes_pre**		notes	Notes:	
**physio_bpwave_instruc**	Instruction	dropdown	BP Waveform Quality	1, Clean signal | 2, Noise | 3, Missing data | 4,Period not run
**physio_bpreason_instruc**		dropdown	BP Quality Reason	1, Clean signal | 2, Interference | 3, PP movement | 4, Loss of signal | 5, Missing Data | 6, Period not run
**physio_bpphysio_instruc**		dropdown	BP Physiology	1, Normal | 2, PVCs | 3, PACs | 4, Other Non-Sinus | 5, Missing Data | 6, Period not run
**physio_bpbi_instruc**		radio	BP BI	1, Whole | 2, Partial | 3, None| 4, Missing Data | 5, Period not run
**physio_ecgwave_instruc**		dropdown	ECG Waveform Quality	1, Clean signal | 2, Noise | 3, Missing data | 4,Period not run

**physio_ecgreason_instruc**		dropdown	ECG Quality Reason	1, Clean signal | 2, Interference | 3, PP movement | 4, Loss of signal | 5, Missing Data | 6, Period not run
**physio_ecgphysio_instruc**		dropdown	ECG Physiology	1, Normal | 2, PVCs | 3, PACs | 4, Other Non-Sinus | 5, Missing Data | 6, Period not run
**physio_ecgbi_instruc**		radio	ECG BI	1, Whole | 2, Partial | 3, None| 4, Missing Data | 5, Period not run
**physio_rspwave_instruc**		dropdown	RSP Waveform Quality	1, Clean signal | 2, Noise | 3, Period not run | 4,Missing data
**physio_rspreason_instruc**		dropdown	RSP Quality Reason	1, Normal | 2, Interference | 3, PP movement | 4, Loss of signal | 5, Missing Data | 6, Period not run
**physio_notes_instruc**		notes	Notes:	
**physio_bpwave_preptask**	Speech Preparation	dropdown	BP Waveform Quality	1, Clean signal | 2, Noise | 3, Missing data | 4,Period not run
**physio_bpreason_preptask**		dropdown	BP Quality Reason	1, Clean signal | 2, Interference | 3, PP movement | 4, Loss of signal | 5, Missing Data | 6, Period not run
**physio_bpphysio_preptask**		dropdown	BP Physiology	1, Normal | 2, PVCs | 3, PACs | 4, Other Non-Sinus | 5, Missing Data | 6, Period not run
**physio_bpbi_preptask**		radio	BP BI	1, Whole | 2, Partial | 3, None| 4, Missing Data | 5, Period not run
**physio_ecgwave_preptask**		dropdown	ECG Waveform Quality	1, Clean signal | 2, Noise | 3, Missing data | 4,Period not run
**physio_ecgreason_preptask**		dropdown	ECG Quality Reason	1, Clean signal | 2, Interference | 3, PP movement | 4, Loss of signal | 5, Missing Data | 6, Period not run
**physio_ecgphysio_preptask**		dropdown	ECG Physiology	1, Normal | 2, PVCs | 3, PACs | 4, Other Non-Sinus | 5, Missing Data | 6, Period not run

**physio_ecgbi_preptask**		radio	ECG BI	1, Whole | 2, Partial | 3, None| 4, Missing Data | 5, Period not run
**physio_rspwave_preptask**		dropdown	RSP Waveform Quality	1, Clean signal | 2, Noise | 3, Period not run | 4,Missing data
**physio_rspreason_preptask**		dropdown	RSP Quality Reason	1, Normal | 2, Interference | 3, PP movement | 4, Loss of signal | 5, Missing Data | 6, Period not run
**physio_notes_preptask**		notes	Notes:	
**physio_bpwave_task**	Psychological Stress Onset	dropdown	BP Waveform Quality	1, Clean signal | 2, Noise | 3, Missing data | 4,Period not run
**physio_bpreason_task**		dropdown	BP Quality Reason	1, Clean signal | 2, Interference | 3, PP movement | 4, Loss of signal | 5, Missing Data | 6, Period not run
**physio_bpphysio_task**		dropdown	BP Physiology	1, Normal | 2, PVCs | 3, PACs | 4, Other Non-Sinus | 5, Missing Data | 6, Period not run
**physio_bpbi_task**		radio	BP BI	1, Whole | 2, Partial | 3, None| 4, Missing Data | 5, Period not run
**physio_ecgwave_task**		dropdown	ECG Waveform Quality	1, Clean signal | 2, Noise | 3, Missing data | 4,Period not run
**physio_ecgreason_task**		dropdown	ECG Quality Reason	1, Clean signal | 2, Interference | 3, PP movement | 4, Loss of signal | 5, Missing Data | 6, Period not run
**physio_ecgphysio_task**		dropdown	ECG Physiology	1, Normal | 2, PVCs | 3, PACs | 4, Other Non-Sinus | 5, Missing Data | 6, Period not run
**physio_ecgbi_task**		radio	ECG BI	1, Whole | 2, Partial | 3, None| 4, Missing Data | 5, Period not run
**physio_rspwave_task**		dropdown	RSP Waveform Quality	1, Clean signal | 2, Noise | 3, Period not run | 4,Missing data

**physio_rspreason_task**		dropdown	RSP Quality Reason	1, Normal | 2, Interference | 3, PP movement | 4, Loss of signal | 5, Missing Data | 6, Period not run
**physio_notes_task**		notes	Notes:	
**physio_bpwave_post5**	5 min	dropdown	BP Waveform Quality	1, Clean signal | 2, Noise | 3, Missing data | 4,Period not run
**physio_bpreason_post5**		dropdown	BP Quality Reason	1, Clean signal | 2, Interference | 3, PP movement | 4, Loss of signal | 5, Missing Data | 6, Period not run
**physio_bpphysio_post5**		dropdown	BP Physiology	1, Normal | 2, PVCs | 3, PACs | 4, Other Non-Sinus | 5, Missing Data | 6, Period not run
**physio_bpbi_post5**		radio	BP BI	1, Whole | 2, Partial | 3, None| 4, Missing Data | 5, Period not run
**physio_ecgwave_post5**		dropdown	ECG Waveform Quality	1, Clean signal | 2, Noise | 3, Missing data | 4,Period not run
**physio_ecgreason_post5**		dropdown	ECG Quality Reason	1, Clean signal | 2, Interference | 3, PP movement | 4, Loss of signal | 5, Missing Data | 6, Period not run
**physio_ecgphysio_post5**		dropdown	ECG Physiology	1, Normal | 2, PVCs | 3, PACs | 4, Other Non-Sinus | 5, Missing Data | 6, Period not run
**physio_ecgbi_post5**		radio	ECG BI	1, Whole | 2, Partial | 3, None| 4, Missing Data | 5, Period not run
**physio_rspwave_post5**		dropdown	RSP Waveform Quality	1, Clean signal | 2, Noise | 3, Period not run | 4,Missing data
**physio_rspreason_post5**		dropdown	RSP Quality Reason	1, Normal | 2, Interference | 3, PP movement | 4, Loss of signal | 5, Missing Data | 6, Period not run
**physio_notes_post5**		notes	Notes:	
**physio_bpwave_post10**	10 min	dropdown	BP Waveform Quality	1, Clean signal | 2, Noise | 3, Missing data | 4,Period not run

**physio_bpreason_post10**		dropdown	BP Quality Reason	1, Clean signal | 2, Interference | 3, PP movement | 4, Loss of signal | 5, Missing Data | 6, Period not run
**physio_bpphysio_post10**		dropdown	BP Physiology	1, Normal | 2, PVCs | 3, PACs | 4, Other Non-Sinus | 5, Missing Data | 6, Period not run
**physio_bpbi_post10**		radio	BP BI	1, Whole | 2, Partial | 3, None| 4, Missing Data | 5, Period not run
**physio_ecgwave_post10**		dropdown	ECG Waveform Quality	1, Clean signal | 2, Noise | 3, Missing data | 4,Period not run
**physio_ecgreason_post10**		dropdown	ECG Quality Reason	1, Clean signal | 2, Interference | 3, PP movement | 4, Loss of signal | 5, Missing Data | 6, Period not run
**physio_ecgphysio_post10**		dropdown	ECG Physiology	1, Normal | 2, PVCs | 3, PACs | 4, Other Non-Sinus | 5, Missing Data | 6, Period not run
**physio_ecgbi_post10**		radio	ECG BI	1, Whole | 2, Partial | 3, None| 4, Missing Data | 5, Period not run
**physio_rspwave_post10**		dropdown	RSP Waveform Quality	1, Clean signal | 2, Noise | 3, Period not run | 4,Missing data
**physio_rspreason_post10**		dropdown	RSP Quality Reason	1, Normal | 2, Interference | 3, PP movement | 4, Loss of signal | 5, Missing Data | 6, Period not run
**physio_notes_post10**		notes	Notes:	
**physio_bpwave_post20**	20 min	dropdown	BP Waveform Quality	1, Clean signal | 2, Noise | 3, Missing data | 4,Period not run
**physio_bpreason_post20**		dropdown	BP Quality Reason	1, Clean signal | 2, Interference | 3, PP movement | 4, Loss of signal | 5, Missing Data | 6, Period not run
**physio_bpphysio_post20**		dropdown	BP Physiology	1, Normal | 2, PVCs | 3, PACs | 4, Other Non-Sinus | 5, Missing Data | 6, Period not run

**physio_bpbi_post20**		radio	BP BI	1, Whole | 2, Partial | 3, None| 4, Missing Data | 5, Period not run
**physio_ecgwave_post20**		dropdown	ECG Waveform Quality	1, Clean signal | 2, Noise | 3, Missing data | 4,Period not run
**physio_ecgreason_post20**		dropdown	ECG Quality Reason	1, Clean signal | 2, Interference | 3, PP movement | 4, Loss of signal | 5, Missing Data | 6, Period not run
**physio_ecgphysio_post20**		dropdown	ECG Physiology	1, Normal | 2, PVCs | 3, PACs | 4, Other Non-Sinus | 5, Missing Data | 6, Period not run
**physio_ecgbi_post20**		radio	ECG BI	1, Whole | 2, Partial | 3, None| 4, Missing Data | 5, Period not run
**physio_rspwave_post20**		dropdown	RSP Waveform Quality	1, Clean signal | 2, Noise | 3, Period not run | 4,Missing data
**physio_rspreason_post20**		dropdown	RSP Quality Reason	1, Normal | 2, Interference | 3, PP movement | 4, Loss of signal | 5, Missing Data | 6, Period not run
**physio_notes_post20**		notes	Notes:	
**physio_bpwave_post30**	30 min	dropdown	BP Waveform Quality	1, Clean signal | 2, Noise | 3, Missing data | 4,Period not run
**physio_bpreason_post30**		dropdown	BP Quality Reason	1, Clean signal | 2, Interference | 3, PP movement | 4, Loss of signal | 5, Missing Data | 6, Period not run
**physio_bpphysio_post30**		dropdown	BP Physiology	1, Normal | 2, PVCs | 3, PACs | 4, Other Non-Sinus | 5, Missing Data | 6, Period not run
**physio_bpbi_post30**		radio	BP BI	1, Whole | 2, Partial | 3, None| 4, Missing Data | 5, Period not run
**physio_ecgwave_post30**		dropdown	ECG Waveform Quality	1, Clean signal | 2, Noise | 3, Missing data | 4,Period not run
**physio_ecgreason_post30**		dropdown	ECG Quality Reason	1, Clean signal | 2, Interference | 3, PP movement | 4, Loss of signal | 5, Missing Data | 6, Period not run

**physio_ecgphysio_post30**		dropdown	ECG Physiology	1, Normal | 2, PVCs | 3, PACs | 4, Other Non-Sinus | 5, Missing Data | 6, Period not run
**physio_ecgbi_post30**		radio	ECG BI	1, Whole | 2, Partial | 3, None| 4, Missing Data | 5, Period not run
**physio_rspwave_post30**		dropdown	RSP Waveform Quality	1, Clean signal | 2, Noise | 3, Period not run | 4,Missing data
**physio_rspreason_post30**		dropdown	RSP Quality Reason	1, Normal | 2, Interference | 3, PP movement | 4, Loss of signal | 5, Missing Data | 6, Period not run
**physio_notes_post30**		notes	Notes:	
**physio_bpwave_post60**	60 min	dropdown	BP Waveform Quality	1, Clean signal | 2, Noise | 3, Missing data | 4,Period not run
**physio_bpreason_post60**		dropdown	BP Quality Reason	1, Clean signal | 2, Interference | 3, PP movement | 4, Loss of signal | 5, Missing Data | 6, Period not run
**physio_bpphysio_post60**		dropdown	BP Physiology	1, Normal | 2, PVCs | 3, PACs | 4, Other Non-Sinus | 5, Missing Data | 6, Period not run
**physio_bpbi_post60**		radio	BP BI	1, Whole | 2, Partial | 3, None| 4, Missing Data | 5, Period not run
**physio_ecgwave_post60**		dropdown	ECG Waveform Quality	1, Clean signal | 2, Noise | 3, Missing data | 4,Period not run
**physio_ecgreason_post60**		dropdown	ECG Quality Reason	1, Clean signal | 2, Interference | 3, PP movement | 4, Loss of signal | 5, Missing Data | 6, Period not run
**physio_ecgphysio_post60**		dropdown	ECG Physiology	1, Normal | 2, PVCs | 3, PACs | 4, Other Non-Sinus | 5, Missing Data | 6, Period not run
**physio_ecgbi_post60**		radio	ECG BI	1, Whole | 2, Partial | 3, None| 4, Missing Data | 5, Period not run
**physio_rspwave_post60**		dropdown	RSP Waveform Quality	1, Clean signal | 2, Noise | 3, Period not run | 4,Missing data

**physio_rspreason_post60**		dropdown	RSP Quality Reason	1, Normal | 2, Interference | 3, PP movement | 4, Loss of signal | 5, Missing Data | 6, Period not run
**physio_notes_post60**		notes	Notes:	
**physio_bpwave_post90**	90 min	dropdown	BP Waveform Quality	1, Clean signal | 2, Noise | 3, Missing data | 4,Period not run
**physio_bpreason_post90**		dropdown	BP Quality Reason	1, Clean signal | 2, Interference | 3, PP movement | 4, Loss of signal | 5, Missing Data | 6, Period not run
**physio_bpphysio_post90**		dropdown	BP Physiology	1, Normal | 2, PVCs | 3, PACs | 4, Other Non-Sinus | 5, Missing Data | 6, Period not run
**physio_bpbi_post90**		radio	BP BI	1, Whole | 2, Partial | 3, None| 4, Missing Data | 5, Period not run
**physio_ecgwave_post90**		dropdown	ECG Waveform Quality	1, Clean signal | 2, Noise | 3, Missing data | 4,Period not run
**physio_ecgreason_post90**		dropdown	ECG Quality Reason	1, Clean signal | 2, Interference | 3, PP movement | 4, Loss of signal | 5, Missing Data | 6, Period not run
**physio_ecgphysio_post90**		dropdown	ECG Physiology	1, Normal | 2, PVCs | 3, PACs | 4, Other Non-Sinus | 5, Missing Data | 6, Period not run
**physio_ecgbi_post90**		radio	ECG BI	1, Whole | 2, Partial | 3, None| 4, Missing Data | 5, Period not run
**physio_rspwave_post90**		dropdown	RSP Waveform Quality	1, Clean signal | 2, Noise | 3, Period not run | 4,Missing data
**physio_rspreason_post90**		dropdown	RSP Quality Reason	1, Normal | 2, Interference | 3, PP movement | 4, Loss of signal | 5, Missing Data | 6, Period not run
**physio_notes_post90**		notes	Notes:	
**physio_bpwave_post120**	120 min	dropdown	BP Waveform Quality	1, Clean signal | 2, Noise | 3, Missing data | 4,Period not run

**physio_bpreason_post120**		dropdown	BP Quality Reason	1, Clean signal | 2, Interference | 3, PP movement | 4, Loss of signal | 5, Missing Data | 6, Period not run
**physio_bpphysio_post120**		dropdown	BP Physiology	1, Normal | 2, PVCs | 3, PACs | 4, Other Non-Sinus | 5, Missing Data | 6, Period not run
**physio_bpbi_post120**		radio	BP BI	1, Whole | 2, Partial | 3, None| 4, Missing Data | 5, Period not run
**physio_ecgwave_post120**		dropdown	ECG Waveform Quality	1, Clean signal | 2, Noise | 3, Missing data | 4,Period not run
**physio_ecgreason_post120**		dropdown	ECG Quality Reason	1, Clean signal | 2, Interference | 3, PP movement | 4, Loss of signal | 5, Missing Data | 6, Period not run
**physio_ecgphysio_post120**		dropdown	ECG Physiology	1, Normal | 2, PVCs | 3, PACs | 4, Other Non-Sinus | 5, Missing Data | 6, Period not run
**physio_ecgbi_post120**		radio	ECG BI	1, Whole | 2, Partial | 3, None| 4, Missing Data | 5, Period not run
**physio_rspwave_post120**		dropdown	RSP Waveform Quality	1, Clean signal | 2, Noise | 3, Period not run | 4,Missing data
**physio_rspreason_post120**		dropdown	RSP Quality Reason	1, Normal | 2, Interference | 3, PP movement | 4, Loss of signal | 5, Missing Data | 6, Period not run
**physio_notes_post120**		notes	Notes:	
**physio_bpwave_dbt**	Deep breathing task	dropdown	BP Waveform Quality	1, Clean signal | 2, Noise | 3, Missing data | 4,Period not run
**physio_bpreason_dbt**		dropdown	BP Quality Reason	1, Clean signal | 2, Interference | 3, PP movement | 4, Loss of signal | 5, Missing Data | 6, Period not run
**physio_bpphysio_dbt**		dropdown	BP Physiology	1, Normal | 2, PVCs | 3, PACs | 4, Other Non-Sinus | 5, Missing Data | 6, Period not run

**physio_bpbi_dbt**		radio	BP BI	1, Whole | 2, Partial | 3, None| 4, Missing Data | 5, Period not run
**physio_ecgwave_dbt**		dropdown	ECG Waveform Quality	1, Clean signal | 2, Noise | 3, Missing data | 4,Period not run
**physio_ecgreason_dbt**		dropdown	ECG Quality Reason	1, Clean signal | 2, Interference | 3, PP movement | 4, Loss of signal | 5, Missing Data | 6, Period not run
**physio_ecgphysio_dbt**		dropdown	ECG Physiology	1, Normal | 2, PVCs | 3, PACs | 4, Other Non-Sinus | 5, Missing Data | 6, Period not run
**physio_ecgbi_dbt**		radio	ECG BI	1, Whole | 2, Partial | 3, None| 4, Missing Data | 5, Period not run
**physio_rspwave_dbt**		dropdown	RSP Waveform Quality	1, Clean signal | 2, Noise | 3, Period not run | 4,Missing data
**physio_rspreason_dbt**		dropdown	RSP Quality Reason	1, Normal | 2, Interference | 3, PP movement | 4, Loss of signal | 5, Missing Data | 6, Period not run
**physio_notes_dbt**		notes	Notes:	
**physio_bpwave_st**	Standing transition	dropdown	BP Waveform Quality	1, Clean signal | 2, Noise | 3, Missing data | 4,Period not run
**physio_bpreason_st**		dropdown	BP Quality Reason	1, Clean signal | 2, Interference | 3, PP movement | 4, Loss of signal | 5, Missing Data | 6, Period not run
**physio_bpphysio_st**		dropdown	BP Physiology	1, Normal | 2, PVCs | 3, PACs | 4, Other Non-Sinus | 5, Missing Data | 6, Period not run
**physio_bpbi_st**		radio	BP BI	1, Whole | 2, Partial | 3, None| 4, Missing Data | 5, Period not run
**physio_ecgwave_st**		dropdown	ECG Waveform Quality	1, Clean signal | 2, Noise | 3, Missing data | 4,Period not run
**physio_ecgreason_st**		dropdown	ECG Quality Reason	1, Clean signal | 2, Interference | 3, PP movement | 4, Loss of signal | 5, Missing Data | 6, Period not run

**physio_ecgphysio_st**		dropdown	ECG Physiology	1, Normal | 2, PVCs | 3, PACs | 4, Other Non-Sinus | 5, Missing Data | 6, Period not run
**physio_ecgbi_st**		radio	ECG BI	1, Whole | 2, Partial | 3, None| 4, Missing Data | 5, Period not run
**physio_rspwave_st**		dropdown	RSP Waveform Quality	1, Clean signal | 2, Noise | 3, Period not run | 4,Missing data
**physio_rspreason_st**		dropdown	RSP Quality Reason	1, Normal | 2, Interference | 3, PP movement | 4, Loss of signal | 5, Missing Data | 6, Period not run
**physio_notes_st**		notes	Notes:	
**physio_bpwave_sst**	Sit-stand test	dropdown	BP Waveform Quality	1, Clean signal | 2, Noise | 3, Missing data | 4,Period not run
**physio_bpreason_sst**		dropdown	BP Quality Reason	1, Clean signal | 2, Interference | 3, PP movement | 4, Loss of signal | 5, Missing Data | 6, Period not run
**physio_bpphysio_sst**		dropdown	BP Physiology	1, Normal | 2, PVCs | 3, PACs | 4, Other Non-Sinus | 5, Missing Data | 6, Period not run
**physio_bpbi_sst**		radio	BP BI	1, Whole | 2, Partial | 3, None| 4, Missing Data | 5, Period not run
**physio_ecgwave_sst**		dropdown	ECG Waveform Quality	1, Clean signal | 2, Noise | 3, Missing data | 4,Period not run
**physio_ecgreason_sst**		dropdown	ECG Quality Reason	1, Clean signal | 2, Interference | 3, PP movement | 4, Loss of signal | 5, Missing Data | 6, Period not run
**physio_ecgphysio_sst**		dropdown	ECG Physiology	1, Normal | 2, PVCs | 3, PACs | 4, Other Non-Sinus | 5, Missing Data | 6, Period not run
**physio_ecgbi_sst**		radio	ECG BI	1, Whole | 2, Partial | 3, None| 4, Missing Data | 5, Period not run
**physio_rspwave_sst**		dropdown	RSP Waveform Quality	1, Clean signal | 2, Noise | 3, Period not run | 4,Missing data

**physio_rspreason_sst**		dropdown	RSP Quality Reason	1, Normal | 2, Interference | 3, PP movement | 4, Loss of signal | 5, Missing Data | 6, Period not run
**physio_notes_sst**		notes	Notes:	
**physio_bpwave_cp**	Cold Pressor	dropdown	BP Waveform Quality	1, Clean signal | 2, Noise | 3, Missing data | 4,Period not run
**physio_bpreason_cp**		dropdown	BP Quality Reason	1, Clean signal | 2, Interference | 3, PP movement | 4, Loss of signal | 5, Missing Data | 6, Period not run
**physio_bpphysio_cp**		dropdown	BP Physiology	1, Normal | 2, PVCs | 3, PACs | 4, Other Non-Sinus | 5, Missing Data | 6, Period not run
**physio_bpbi_cp**		radio	BP BI	1, Whole | 2, Partial | 3, None| 4, Missing Data | 5, Period not run
**physio_ecgwave_cp**		dropdown	ECG Waveform Quality	1, Clean signal | 2, Noise | 3, Missing data | 4,Period not run
**physio_ecgreason_cp**		dropdown	ECG Quality Reason	1, Clean signal | 2, Interference | 3, PP movement | 4, Loss of signal | 5, Missing Data | 6, Period not run
**physio_ecgphysio_cp**		dropdown	ECG Physiology	1, Normal | 2, PVCs | 3, PACs | 4, Other Non-Sinus | 5, Missing Data | 6, Period not run
**physio_ecgbi_cp**		radio	ECG BI	1, Whole | 2, Partial | 3, None| 4, Missing Data | 5, Period not run
**physio_rspwave_cp**		dropdown	RSP Waveform Quality	1, Clean signal | 2, Noise | 3, Period not run | 4,Missing data
**physio_rspreason_cp**		dropdown	RSP Quality Reason	1, Normal | 2, Interference | 3, PP movement | 4, Loss of signal | 5, Missing Data | 6, Period not run
**physio_notes_cp**		notes	Notes:	

1.4 Biospecimen Analysis

1.4.1 Seahorse Data

RedCAP Form Name: Seahorse

Description: Seahorse data, images, and notes

Variable / Field Name	Section Header	Field Type	Field Label	Variable / Field Name
**seahorse_expr**	Seahorse	text	<div class="rich-text-field-label"><p>Experimenter name<br></p></div>	
**run_count**		text	Run Count	
**seahorse_date_run**		text	Date of Run	
**seahorse_comments**		dropdown	Seahorse Comments	1, Perfect | 2, Not Perfect
**seahorse_not_perf_rsn**		checkbox	What was not perfect? (multiple choice)	1, Instrument Prep Error | 2, Instrument Runtime Error | 3, Pyruvate Inhibitor Injection Failure | 4, Oligo Injection Failure | 5, FCCP Injection Failure | 6, R+A Injection Failure | 7, Blood Separation Failure | 8, Platelet Stickiness | 9, Not Enough Platelets | 10, Not Enough Neutrophils | 11, Not Enough Lymphocytes | 12, Not Enough Monocytes
**lym_yield**		text	Lymphocytes Yield (Mcells)	

**lym_base_resp**		text	Lymphocytes Baseline Respiration (pmon/min)	
**lym_basal_resp**		text	Lymphocytes Basal Respiration (pmol/min)	
**lym_h_leak**		text	Lymphocytes Proton Leak (pmol/min)	
**lym_atp_resp**		text	Lymphocytes ATP-Linked Respiration (pmol/min)	
**lym_cpl_eff**		text	Lymphocytes Coupling Efficiency (pmol/min)	
**lym_non_resp**		text	Lymphocytes Non-Mitochondrial Respiration (pmol/min)	
**lym_max_resp**		text	Lymphocytes Max Respiration (pmol/min)	
**lym_sparc**		text	Lymphocytes Spare Capacity (pmol/min)	
**lym_base_ecar**		text	Lymphocytes Baseline ECAR (mpH/min)	
**lym_olig_ecar**		text	Lymphocytes Oligo ECAR (mpH/min)	
**lym_fccp_ecar**		text	Lymphocytes FCCP ECAR (mpH/min)	
**lym_ra_ecar**		text	Lymphocytes Rotenone + Antimycin A ECAR (mpH/min)	
**lym_basal_mbs**		text	Lymphocytes Basal Metabolic Switch (%)	
**lym_max_mbs**		text	Lymphocytes Max Metabolic Switch (%)	
**lym_sparc_mbs**		text	Lymphocytes Spare Metabolic Switch (%)	
**lym_base_ecar_mbs**		text	Lymphocytes Baeline ECAR Metabolic Switch (%)	
**lym_fccp_ecar_mbs**		text	Lymphocytes FCCP ECAR Metabolic Switch (%)	

**neut_yield**		text	Neutrophils Yield (Mcells)	
**neut_base_resp**		text	Neutrophils Baseline Respiration (pmon/min)	
**neut_basal_resp**		text	Neutrophils Basal Respiration (pmol/min)	
**neut_h_leak**		text	Neutrophils Proton Leak (pmol/min)	
**neut_atp_resp**		text	Neutrophils ATP-Linked Respiration (pmol/min)	
**neut_cpl_eff**		text	Neutrophils Coupling Efficiency (pmol/min)	
**neut_non_resp**		text	Neutrophils Non-Mitochondrial Respiration (pmol/min)	
**neut_max_resp**		text	Neutrophils Max Respiration (pmol/min)	
**neut_sparc**		text	Neutrophils Spare Capacity (pmol/min)	
**neut_base_ecar**		text	Neutrophils Baseline ECAR (mpH/min)	
**neut_olig_ecar**		text	Neutrophils Oligo ECAR (mpH/min)	
**neut_fccp_ecar**		text	Neutrophils FCCP ECAR (mpH/min)	
**neut_ra_ecar**		text	Neutrophils Rotenone + Antimycin A ECAR (mpH/min)	
**neut_basal_mbs**		text	Neutrophils Basal Metabolic Switch (%)	
**neut_max_mbs**		text	Neutrophils Max Metabolic Switch (%)	
**neut_sparc_mbs**		text	Neutrophils Spare Metabolic Switch (%)	
**neut_base_ecar_mbs**		text	Neutrophils Baeline ECAR Metabolic Switch (%)	

**neut_fccp_ecar_mbs**		text	Neutrophils FCCP ECAR Metabolic Switch (%)	
**mono_yield**		text	Monocytes Yield (Mcells)	
**mono_base_resp**		text	Monocytes Baseline Respiration (pmon/min)	
**mono_basal_resp**		text	Monocytes Basal Respiration (pmol/min)	
**mono_h_leak**		text	Monocytes Proton Leak (pmol/min)	
**mono_atp_resp**		text	Monocytes ATP-Linked Respiration (pmol/min)	
**mono_cpl_eff**		text	Monocytes Coupling Efficiency (pmol/min)	
**mono_non_resp**		text	MonocytesNon-Mitochondrial Respiration (pmol/min)	
**mono_max_resp**		text	Monocytes Max Respiration (pmol/min)	
**mono_sparc**		text	Monocytes Spare Capacity (pmol/min)	
**mono_base_ecar**		text	Monocytes Baseline ECAR (mpH/min)	
**mono_olig_ecar**		text	Monocytes Oligo ECAR (mpH/min)	
**mono_fccp_ecar**		text	Monocytes FCCP ECAR (mpH/min)	
**mono_ra_ecar**		text	MonocytesRotenone + Antimycin A ECAR (mpH/min)	
**mono_basal_mbs**		text	Monocytes Basal Metabolic Switch (%)	
**mono_max_mbs**		text	Monocytes Max Metabolic Switch (%)	
**mono_sparc_mbs**		text	Monocytes Spare Metabolic Switch (%)	

**mono_base_ecar_mbs**		text	Monocytes Baeline ECAR Metabolic Switch (%)	
**mono_fccp_ecar_mbs**		text	Monocytes FCCP ECAR Metabolic Switch (%)	
**plat_yield**		text	Platelets Yield (Mcells)	
**plat_base_resp**		text	Platelets Baseline Respiration (pmon/min)	
**plat_basal_resp**		text	Platelets Basal Respiration (pmol/min)	
**plat_h_leak**		text	Platelets Proton Leak (pmol/min)	
**plat_atp_resp**		text	Platelets ATP-Linked Respiration (pmol/min)	
**plat_cpl_eff**		text	Platelets Coupling Efficiency (pmol/min)	
**plat_non_resp**		text	Platelets Non-Mitochondrial Respiration (pmol/min)	
**plat_max_resp**		text	Platelets Max Respiration (pmol/min)	
**plat_sparc**		text	Platelets Spare Capacity (pmol/min)	
**plat_base_ecar**		text	Platelets Baseline ECAR (mpH/min)	
**plat_olig_ecar**		text	Platelets Oligo ECAR (mpH/min)	
**plat_fccp_ecar**		text	Platelets FCCP ECAR (mpH/min)	
**plat_ra_ecar**		text	Platelets Rotenone + Antimycin A ECAR (mpH/min)	
**plat_basal_mbs**		text	Platelets Basal Metabolic Switch (%)	
**plat_max_mbs**		text	Platelets Max Metabolic Switch (%)	

**plat_sparc_mbs**		text	Platelets Spare Metabolic Switch (%)	
**plat_base_ecar_mbs**		text	Platelets Baeline ECAR Metabolic Switch (%)	
**plat_fccp_ecar_mbs**		text	Platelets FCCP ECAR Metabolic Switch (%)	
**hek_yield**		text	HEK293 Yield (Mcells)	
**hek_base_resp**		text	HEK293 Baseline Respiration (pmon/min)	
**hek_basal_resp**		text	HEK293 Basal Respiration (pmol/min)	
**hek_h_leak**		text	HEK293 Proton Leak (pmol/min)	
**hek_atp_resp**		text	HEK293 ATP-Linked Respiration (pmol/min)	
**hek_cpl_eff**		text	HEK293 Coupling Efficiency (pmol/min)	
**hek_non_resp**		text	HEK293 Non-Mitochondrial Respiration (pmol/min)	
**hek_max_resp**		text	HEK293 Max Respiration (pmol/min)	
**hek_sparc**		text	HEK293 Spare Capacity (pmol/min)	
**hek_base_ecar**		text	HEK293 Baseline ECAR (mpH/min)	
**hek_olig_ecar**		text	HEK293 Oligo ECAR (mpH/min)	
**hek_fccp_ecar**		text	HEK293 FCCP ECAR (mpH/min)	
**hek_ra_ecar**		text	HEK293 Rotenone + Antimycin A ECAR (mpH/min)	
**hek_basal_mbs**		text	HEK293 Basal Metabolic Switch (%)	

**hek_max_mbs**		text	HEK293 Max Metabolic Switch (%)	
**hek_sparc_mbs**		text	HEK293 Spare Metabolic Switch (%)	
**hek_base_ecar_mbs**		text	HEK293 Baeline ECAR Metabolic Switch (%)	
**hek_fccp_ecar_mbs**		text	HEK293 FCCP ECAR Metabolic Switch (%)	
**lym_cell_size**		text	Lymphocytes Cell Size (um)	
**lym_dead**		text	Lymphocytes Dead Cells (%)	
**neut_cell_size**		text	Neutrophiles Cell Size (um)	
**neut_dead**		text	Neutrophils Dead Cells (%)	
**mono_cell_size**		text	Monocytes Cell Size (um)	
**mono_dead**		text	Monocytes Dead Cells (%)	
**lym_well_pic**		file	Lymphocyte Picture	
**neut_well_pic**		file	Neutrophil Picture	
**mono_well_pic**		file	Monocytes Picture	
**plat_well_pic**		file	Platelet Picture	
**hek_well_pic**		file	HEK293 Picture	

1.4.2 Elisa Run

RedCAP Form Name: lps

Description: Old form, no longer in use

Variable / Field Name	Section Header	Field Type	Field Label
**elisa_date_run**		text	Date of Run
**elisa_run**		radio	How did the ELISA run go?
**elisa_run_not_perf**		radio	reason:
**elisa_run_notes**		radio	Explanatory notes
**lps_run**		radio	How did the LPS treatment go?
**lsp_run_not_perf**		radio	reason:
**lps_run_notes**		radio	Explanatory notes
**lps_incub_time**		text	LPS Incubation Time (hrs)
**sam_type**		text	Sample type
**lps_50_il6**		text	LPS 50 IL-6 (pg/ml)
**lps_10_il6**		text	LPS 10 IL-6 (pg/ml)
**lps_2_il6**		text	LPS 2 IL-6 (pg/ml)
**lps_04_il6**		text	LPS 0.4 IL-6 (pg/ml)
**lps_008_il6**		text	LPS 0.08 IL-6 (pg/ml)
**lps_0016_il6**		text	LPS 0.016 IL-6 (pg/ml)
**lps_00032_il6**		text	LPS 0.0032 IL-6 (pg/ml)

**lps_untr_ctrl**		text	Untreated control (pg/ml)
**lps_50_dex_il6**		text	LPS 50 DEX IL-6 (pg/ml)
**lps_10_dex_il6**		text	LPS 10 DEX IL-6 (pg/ml)
**lps_2_dex_il6**		text	LPS 2 DEX IL-6 (pg/ml)
**lps_04_dex_il6**		text	LPS 0.4 DEX IL-6 (pg/ml)
**lps_008_dex_il6**		text	LPS 0.08 DEX IL-6 (pg/ml)
**lps_0016_dex_il6**		text	LPS 0.016 DEX IL-6 (pg/ml)
**lps_00032_dex_il6**		text	LPS 0.0032 DEX IL-6 (pg/ml)
**dex_il6**		text	DEX alone (pg/ml)
**lps_50_rot_il6**		text	LPS 50 Rot IL-6 (pg/ml)
**lps_10_rot_il6**		text	LPS 10 Rot IL-6 (pg/ml)
**lps_2_rot_il6**		text	LPS 2 Rot IL-6 (pg/ml)
**lps_04_rot_il6**		text	LPS 0.4 Rot IL-6 (pg/ml)
**lps_008_rot_il6**		text	LPS 0.08 Rot IL-6 (pg/ml)
**lps_0016_rot_il6**		text	LPS 0.016 Rot IL-6 (pg/ml)
**lps_00032_rot_il6**		text	LPS 0.0032 Rot IL-6 (pg/ml)
**rot_il6**		text	Rot alone (pg/ml)

**lps_50_pa_il6**		text	LPS 50 PA IL-6 (pg/ml)
**lps_10_pa_il6**		text	LPS 10 PA IL-6 (pg/ml)
**lps_2_pa_il6**		text	LPS 2 PA IL-6 (pg/ml)
**lps_04_pa_il6**		text	LPS 0.4 PA IL-6 (pg/ml)
**lps_008_pa_il6**		text	LPS 0.08 PA IL-6 (pg/ml)
**lps_0016_pa_il6**		text	LPS 0.016 PA IL-6 (pg/ml)
**lps_00032_pa_il6**		text	LPS 0.0032 PA IL-6 (pg/ml)
**pa_il6**		text	PA alone (pg/ml)
**lps_50_aa_il6**		text	LPS 50 AA IL-6 (pg/ml)
**lps_10_aa_il6**		text	LPS 10 AA IL-6 (pg/ml)
**lps_2_aa_il6**		text	LPS 2 AA IL-6 (pg/ml)
**lps_04_aa_il6**		text	LPS 0.4 AA IL-6 (pg/ml)
**lps_008_aa_il6**		text	LPS 0.08 AA IL-6 (pg/ml)
**lps_0016_aa_il6**		text	LPS 0.016 AA IL-6 (pg/ml)
**lps_00032_aa_il6**		text	LPS 0.0032 AA IL-6 (pg/ml)
**aa_il6**		text	AA alone (pg/ml)
**lps_50_kcn_il6**		text	LPS 50 KCN IL-6 (pg/ml)

**lps_10_kcn_il6**		text	LPS 10 KCN IL-6 (pg/ml)
**lps_2_kcn_il6**		text	LPS 2 KCN IL-6 (pg/ml)
**lps_04_kcn_il6**		text	LPS 0.4 KCN IL-6 (pg/ml)
**lps_008_kcn_il6**		text	LPS 0.08 KCN IL-6 (pg/ml)
**lps_0016_kcn_il6**		text	LPS 0.016 KCN IL-6 (pg/ml)
**lps_00032_kcn_il6**		text	LPS 0.0032 KCN IL-6 (pg/ml)
**kcn_il6**		text	KCN alone (pg/ml)
**lps_50_oli_il6**		text	LPS 50 Oligo IL-6 (pg/ml)
**lps_10_oli_il6**		text	LPS 10 Oligo IL-6 (pg/ml)
**lps_2_oli_il6**		text	LPS 2 Oligo IL-6 (pg/ml)
**lps_04_oli_il6**		text	LPS 0.4 Oligo IL-6 (pg/ml)
**lps_008_oli_il6**		text	LPS 0.08 Oligo IL-6 (pg/ml)
**lps_0016_oli_il6**		text	LPS 0.016 Oligo IL-6 (pg/ml)
**lps_00032_oli_il6**		text	LPS 0.0032 Oligo IL-6 (pg/ml)
**oli_il6**		text	Oligo alone (pg/ml)
**lps_50_csa_il6**		text	LPS 50 CsA IL-6 (pg/ml)
**lps_10_csa_il6**		text	LPS 10 CsA IL-6 (pg/ml)

**lps_2_csa_il6**		text	LPS 2 CsA IL-6 (pg/ml)
**lps_04_csa_il6**		text	LPS 0.4 CsA IL-6 (pg/ml)
**lps_008_csa_il6**		text	LPS 0.08 CsA IL-6 (pg/ml)
**lps_0016_csa_il6**		text	LPS 0.016 CsA IL-6 (pg/ml)
**lps_00032_csa_il6**		text	LPS 0.0032 CsA IL-6 (pg/ml)
**csa_il6**		text	CsA alone (pg/ml)
**lps_50_fccp_il6**		text	LPS 50 Fccp IL-6 (pg/ml)
**lps_10_fccp_il6**		text	LPS 10 Fccp IL-6 (pg/ml)
**lps_2_fccp_il6**		text	LPS 2 Fccp IL-6 (pg/ml)
**lps_04_fccp_il6**		text	LPS 0.4 Fccp IL-6 (pg/ml)
**lps_008_fccp_il6**		text	LPS 0.08 Fccp IL-6 (pg/ml)
**lps_0016_fccp_il6**		text	LPS 0.016 Fccp IL-6 (pg/ml)
**lps_00032_fccp_il6**		text	LPS 0.0032 Fccp IL-6 (pg/ml)
**fccp_il6**		text	Fccp alone (pg/ml)
**lps_50_mq_il6**		text	LPS 50 MitoQ IL-6 (pg/ml)
**lps_10_mq_il6**		text	LPS 10 MitoQ IL-6 (pg/ml)
**lps_2_mq_il6**		text	LPS 2 MitoQ IL-6 (pg/ml)

**lps_04_mq_il6**		text	LPS 0.4 MitoQ IL-6 (pg/ml)
**lps_008_mq_il6**		text	LPS 0.08 MitoQ IL-6 (pg/ml)
**lps_0016_mq_il6**		text	LPS 0.016 MitoQ IL-6 (pg/ml)
**lps_00032_mq_il6**		text	LPS 0.0032 MitoQ IL-6 (pg/ml)
**mq_il6**		text	MitoQ alone (pg/ml)
**lps_10_dex_rot_il6**		text	LPS 10 DEX Rot IL-6 (pg/ml)
**lps_10_dex_pa_il6**		text	LPS 10 DEX PA IL-6 (pg/ml)
**lps_10_dex_aa_il6**		text	LPS 10 DEX AA IL-6 (pg/ml)
**lps_10_dex_kcn_il6**		text	LPS 10 DEX KCN IL-6 (pg/ml)
**lps_10_dex_oli_il6**		text	LPS 10 DEX Oligo IL-6 (pg/ml)
**lps_10_dex_csa_il6**		text	LPS 10 DEX CsA IL-6 (pg/ml)
**lps_10_dex_fccp_il6**		text	LPS 10 DEX Fccp IL-6 (pg/ml)
**lps_10_dex_mq_il6**		text	LPS 10 DEX MitoQ IL-6 (pg/ml)
**dex_rot_il6**		text	DEX Rot IL-6 (pg/ml)
**dex_pa_il6**		text	DEX PA IL-6 (pg/ml)
**dex_aa_il6**		text	DEX AA IL-6 (pg/ml)
**dex_kcn_il6**		text	DEX KCN IL-6 (pg/ml)

**dex_oli_il6**		text	DEX Oligo IL-6 (pg/ml)
**dex_csa_il6**		text	DEX CsA IL-6 (pg/ml)
**dex_fccp_il6**		text	DEX Fccp IL-6 (pg/ml)
**dex_mq_il6**		text	DEX MitoQ IL-6 (pg/ml)
**dmso_il6**		text	DMSO IL-6 (pg/ml)
**hr_time_b_300s**	300 second epoch-- ECG Base	text	Time

1.5 Biospecimen Results

1.5.1 CALM Lab

RedCAP Form Name: calm_lab

Description: Center for Advanced Laboratory Medicine (CALM) lab results from morning and afternoon blood samples.

Variable / Field Name	Section Header	Field Type	Field Label
**batchdesc_edta1**	EDTA Morning	text	BatchDesc
**sampletype_edta1**		radio	SampleType
**accessionnum_edta1**		radio	AccessionNum
**specimenid_edta1**		radio	SpecimenID
**collectdate_edta1**		radio	CollectDate
**receiveddate_edta1**		radio	ReceivedDate
**hgba1c_edta1**		radio	HgbA1C

**mchc_edta1**		text	MCHC
**mpv_edta1**		text	MPV
**pct_neutrophils_edta1**		text	pct_Neutrophils
**pct_lymphs_edta1**		text	pct_Lymphs
**pct_monos_edta1**		text	pct_Monos
**pct_eos_edta1**		text	pct_Eos
**pct_basos_edta1**		text	pct_Basos
**wbc_edta1**		text	WBC
**rbc_edta1**		text	RBC
**plt_edta1**		text	PLT
**hemoglobin_edta1**		text	Hemoglobin
**hematocrit_edta1**		text	Hematocrit
**mcv_edta1**		text	MCV
**mch_edta1**		text	MCH
**rdw_edta1**		text	RDW
**ig_edta1**		text	IG%
**neutroabsolute_edta1**		text	NeutroAbsolute

**lymphabsolute_edta1**		text	LymphAbsolute
**monoabsolute_edta1**		text	MonoAbsolute
**eosabsolute_edta1**		text	EosAbsolute
**basoabsolute_edta1**		text	BasoAbsolute
**fibrinogen_blue**	blue morning	text	Fibrinogen
**glucose_serum**	Serum morning	text	Glucose
**cholesterol_serum**		text	Cholesterol
**ldl_serum**		text	LDL
**insulin_serum**		text	Insulin
**sodium_serum**		text	Sodium
**potassium_serum**		text	Potassium
**chloride_serum**		text	Chloride
**co2_serum**		text	CO2
**bun_serum**		text	BUN
**creatinine_serum**		text	Creatinine
**albumin_serum**		text	Albumin
**calcium_serum**		text	Calcium

**triglycerides_serum**		text	Triglycerides
**hdl_serum**		text	HDL
**crp_serum**		text	CRP
**cpeptide_serum**		text	C-Peptide
**batchdesc_edta2**	EDTA Afternoon	text	BatchDesc
**sampletype_edta2**		text	SampleType
**accessionnum_edta2**		text	AccessionNum
**specimenid_edta2**		text	SpecimenID
**collectdate_edta2**		text	CollectDate
**receiveddate_edta2**		text	ReceivedDate
**hgba1c_edta2**		text	HgbA1C
**mchc_edta2**		text	MCHC
**mpv_edta2**		text	MPV
**pct_neutrophils_edta2**		text	pct_Neutrophils
**pct_lymphs_edta2**		text	pct_Lymphs
**pct_monos_edta2**		text	pct_Monos
**pct_eos_edta2**		text	pct_Eos

**pct_basos_edta2**		text	pct_Basos
**wbc_edta2**		text	WBC
**rbc_edta2**		text	RBC
**plt_edta2**		text	PLT
**hemoglobin_edta2**		text	Hemoglobin
**hematocrit_edta2**		text	Hematocrit
**mcv_edta2**		text	MCV
**mch_edta2**		text	MCH
**rdw_edta2**		text	RDW
**ig_edta2**		text	IG%
**neutroabsolute_edta2**		text	NeutroAbsolute
**lymphabsolute_edta2**		text	LymphAbsolute
**monoabsolute_edta2**		text	MonoAbsolute
**eosabsolute_edta2**		text	EosAbsolute
**basoabsolute_edta2**		text	BasoAbsolute

1.5.2 Saliva Steroid Hormone

RedCAP Form Name: saliva_hormone

Description: Saliva hormone results.

Variable / Field Name	Field Type	Field Label
**cort_sal_d1_fast**	text	cort_sal_d1_fast
**cort_sal_stress_b**	text	cort_sal_stress_b
**cort_sal_stress_5**	text	cort_sal_stress_5
**cort_sal_stress_10**	text	cort_sal_stress_10
**cort_sal_stress_20**	text	cort_sal_stress_20
**cort_sal_stress_30**	text	cort_sal_stress_30
**cort_sal_stress_60**	text	cort_sal_stress_60
**cort_sal_stress_90**	text	cort_sal_stress_90
**cort_sal_stress_120**	text	cort_sal_stress_120
**cort_sal_stress_cold**	text	cort_sal_stress_Cold
**cort_sal_d2_fast**	text	cort_sal_d2_fast
**cort_sal_brain1**	text	cort_sal_brain1
**cort_sal_brain2**	text	cort_sal_brain2
**cort_sal_brain3**	text	cort_sal_brain3
**cort_h_d1_aw**	text	cort_h_d1_aw
**cort_h_d1_30**	text	cort_h_d1_30

**cort_h_d1_45**	text	cort_h_d1_45
**cort_h_d1_bed**	text	cort_h_d1_bed
**cort_h_d2_aw**	text	cort_h_d2_aw
**cort_h_d2_30**	text	cort_h_d2_30
**cort_h_d2_45**	text	cort_h_d2_45
**cort_h_d2_bed**	text	cort_h_d2_bed
**cort_h_d3_aw**	text	cort_h_d3_aw
**cort_h_d3_30**	text	cort_h_d3_30
**cort_h_d3_45**	text	cort_h_d3_45
**cort_h_d3_bed**	text	cort_h_d3_bed
**cortisone_sal_d1_fast**	text	cortisone_sal_d1_fast
**cortisone_sal_stress_b**	text	cortisone_sal_stress_b
**cortisone_sal_stress_5**	text	cortisone_sal_stress_5
**cortisone_sal_stress_10**	text	cortisone_cort_sal_stress_10
**cortisone_sal_stress_20**	text	cortisone_sal_stress_20
**cortisone_sal_stress_30**	text	cortisone_sal_stress_30
**cortisone_sal_stress_60**	text	cortisone_sal_stress_60

**cortisone_sal_stress_90**	text	cortisone_sal_stress_90
**cortisone_sal_stress_120**	text	cortisone_sal_stress_120
**cortisone_sal_stress_cold**	text	cortisone_sal_stress_cold
**cortisone_sal_d2_fast**	text	cortisone_sal_d2_fast
**cortisone_sal_brain1**	text	cortisone_sal_brain1
**cortisone_sal_brain2**	text	cortisone_sal_brain2
**cortisone_sal_brain3**	text	cortisone_sal_brain3
**cortisone_h_d1_aw**	text	cortisone_h_d1_aw
**cortisone_h_d1_30**	text	cortisone_h_d1_30
**cortisone_h_d1_45**	text	cortisone_h_d1_45
**cortisone_h_d1_bed**	text	cortisone_h_d1_bed
**cortisone_h_d2_aw**	text	cortisone_h_d2_aw
**cortisone_h_d2_30**	text	cortisone_h_d2_30
**cortisone_h_d2_45**	text	cortisone_h_d2_45
**cortisone_h_d2_bed**	text	cortisone_h_d2_bed
**cortisone_h_d3_aw**	text	cortisone_h_d3_aw
**cortisone_h_d3_30**	text	cortisone_h_d3_30

**cortisone_h_d3_45**	text	cortisone_h_d3_45
**cortisone_h_d3_bed**	text	cortisone_h_d3_bed
**testo_sal_d1_fast**	text	testo_sal_d1_fast
**testo_sal_stress_b**	text	testo_sal_stress_b
**testo_sal_stress_5**	text	testo_sal_stress_5
**testo_sal_stress_10**	text	testo_cort_sal_stress_10
**testo_sal_stress_20**	text	testo_sal_stress_20
**testo_sal_stress_30**	text	testo_sal_stress_30
**testo_sal_stress_60**	text	testo_sal_stress_60
**testo_sal_stress_90**	text	testo_sal_stress_90
**testo_sal_stress_120**	text	testo_sal_stress_120
**testo_sal_stress_cold**	text	testo_sal_stress_cold
**testo_sal_d2_fast**	text	testo_sal_d2_fast
**testo_sal_brain1**	text	testo_sal_brain1
**testo_sal_brain2**	text	testo_sal_brain2
**testo_sal_brain3**	text	testo_sal_brain3
**testo_h_d1_aw**	text	testo_h_d1_aw

**testo_h_d1_30**	text	testo_h_d1_30
**testo_h_d1_45**	text	testo_h_d1_45
**testo_h_d1_bed**	text	testo_h_d1_bed
**testo_h_d2_aw**	text	testo_h_d2_aw
**testo_h_d2_30**	text	testo_h_d2_30
**testo_h_d2_45**	text	testo_h_d2_45
**testo_h_d2_bed**	text	testo_h_d2_bed
**testo_h_d3_aw**	text	testo_h_d3_aw
**testo_h_d3_30**	text	testo_h_d3_30
**testo_h_d3_45**	text	testo_h_d3_45
**testo_h_d3_bed**	text	testo_h_d3_bed
**proges_sal_d1_fast**	text	proges_sal_d1_fast
**proges_sal_stress_b**	text	proges_sal_stress_b
**proges_sal_stress_5**	text	proges_sal_stress_5
**proges_sal_stress_10**	text	proges_cort_sal_stress_10
**proges_sal_stress_20**	text	proges_sal_stress_20
**proges_sal_stress_30**	text	proges_sal_stress_30

**proges_sal_stress_60**	text	proges_sal_stress_60
**proges_sal_stress_90**	text	proges_sal_stress_90
**proges_sal_stress_120**	text	proges_sal_stress_120
**proges_sal_stress_cold**	text	proges_sal_stress_cold
**proges_sal_d2_fast**	text	proges_sal_d2_fast
**proges_sal_brain1**	text	proges_sal_brain1
**proges_sal_brain2**	text	proges_sal_brain2
**proges_sal_brain3**	text	proges_sal_brain3
**proges_h_d1_aw**	text	proges_h_d1_aw
**proges_h_d1_30**	text	proges_h_d1_30
**proges_h_d1_45**	text	proges_h_d1_45
**proges_h_d1_bed**	text	proges_h_d1_bed
**proges_h_d2_aw**	text	proges_h_d2_aw
**proges_h_d2_30**	text	proges_h_d2_30
**proges_h_d2_45**	text	proges_h_d2_45
**proges_h_d2_bed**	text	proges_h_d2_bed
**proges_h_d3_aw**	text	proges_h_d3_aw

**proges_h_d3_30**	text	proges_h_d3_30
**proges_h_d3_45**	text	proges_h_d3_45
**proges_h_d3_bed**	text	proges_h_d3_bed
**dheas_sal_d1_fast**	text	dheas_sal_d1_fast
**dheas_sal_stress_b**	text	dheas_sal_stress_b
**dheas_sal_stress_5**	text	dheas_sal_stress_5
**dheas_sal_stress_10**	text	dheas_cort_sal_stress_10
**dheas_sal_stress_20**	text	dheas_sal_stress_20
**dheas_sal_stress_30**	text	dheas_sal_stress_30
**dheas_sal_stress_60**	text	dheas_sal_stress_60
**dheas_sal_stress_90**	text	dheas_sal_stress_90
**dheas_sal_stress_120**	text	dheas_sal_stress_120
**dheas_sal_stress_cold**	text	dheas_sal_stress_cold
**dheas_sal_d2_fast**	text	dheas_sal_d2_fast
**dheas_sal_brain1**	text	dheas_sal_brain1
**dheas_sal_brain2**	text	dheas_sal_brain2
**dheas_sal_brain3**	text	dheas_sal_brain3

**dheas_h_d1_aw**	text	dheas_h_d1_aw
**dheas_h_d1_30**	text	dheas_h_d1_30
**dheas_h_d1_45**	text	dheas_h_d1_45
**dheas_h_d1_bed**	text	dheas_h_d1_bed
**dheas_h_d2_aw**	text	dheas_h_d2_aw
**dheas_h_d2_30**	text	dheas_h_d2_30
**dheas_h_d2_45**	text	dheas_h_d2_45
**dheas_h_d2_bed**	text	dheas_h_d2_bed
**dheas_h_d3_aw**	text	dheas_h_d3_aw
**dheas_h_d3_30**	text	dheas_h_d3_30
**dheas_h_d3_45**	text	dheas_h_d3_45
**dheas_h_d3_bed**	text	dheas_h_d3_bed
**corticosterone_sal_d1_fast**	text	corticosterone_sal_d1_fast
**corticosterone_sal_stress_b**	text	corticosterone_sal_stress_b
**corticosterone_sal_stress_5**	text	corticosterone_sal_stress_5
**corticosterone_sal_stress_10**	text	corticosterone_cort_sal_stress_10
**corticosterone_sal_stress_20**	text	corticosterone_sal_stress_20

**corticosterone_sal_stress_30**	text	corticosterone_sal_stress_30
**corticosterone_sal_stress_60**	text	corticosterone_sal_stress_60
**corticosterone_sal_stress_90**	text	corticosterone_sal_stress_90
**corticosterone_sal_stress_120**	text	corticosterone_sal_stress_120
**corticosterone_sal_stress_cold**	text	corticosterone_sal_stress_cold
**corticosterone_sal_d2_fast**	text	corticosterone_sal_d2_fast
**corticosterone_sal_brain1**	text	corticosterone_sal_brain1
**corticosterone_sal_brain2**	text	corticosterone_sal_brain2
**corticosterone_sal_brain3**	text	corticosterone_sal_brain3
**corticosterone_h_d1_aw**	text	corticosterone_h_d1_aw
**corticosterone_h_d1_30**	text	corticosterone_h_d1_30
**corticosterone_h_d1_45**	text	corticosterone_h_d1_45
**corticosterone_h_d1_bed**	text	corticosterone_h_d1_bed
**corticosterone_h_d2_aw**	text	corticosterone_h_d2_aw
**corticosterone_h_d2_30**	text	corticosterone_h_d2_30
**corticosterone_h_d2_45**	text	corticosterone_h_d2_45
**corticosterone_h_d2_bed**	text	corticosterone_h_d2_bed

**corticosterone_h_d3_aw**	text	corticosterone_h_d3_aw
**corticosterone_h_d3_30**	text	corticosterone_h_d3_30
**corticosterone_h_d3_45**	text	corticosterone_h_d3_45
**corticosterone_h_d3_bed**	text	corticosterone_h_d3_bed

1.5.3 Hair Steroid Hormone

RedCAP Form Name: hair_steroid_hormone

Description: Hair steroid hormone results.

Variable / Field Name	Section Header	Field Type	Field Label
**cort_hair_1**		text	cort_hair_1
**cort_hair_2**		text	cort_hair_2
**cortisone_hair_1**		text	cortisone_hair_1
**cortisone_hair_2**		text	cortisone_hair_2
**testo_hair_1**		text	testo_hair_1
**testo_hair_2**		text	testo_hair_2
**proges_hair_1**		text	proges_hair_1
**proges_hair_2**		text	proges_hair_2
**dheas_hair_1**		text	DHEAs_hair_1

**dheas_hair_2**		text	DHEAs_hair_2
**corticosterone_hair_1**		text	corticosterone_hair_1
**corticosterone_hair_2**		text	corticosterone_hair_2

1.5.4 Cfdna

RedCAP Form Name: calm_lab

Description: Center for Advanced Laboratory Medicine (CALM) lab results from morning and afternoon blood samples.

Variable / Field Name	Section Header	Field Type	Variable / Field Name
**sample_number_day1_awakening**	Experiment: Awakening	text	unique number for each sample, consecutively numbered from 1-4224_day1_Awakening
**plate_number_day1_awakening**		text	Plate number of qpcr_day1_Awakening
**well_day1_awakening**		text	Well in which sample was plated_day1_Awakening
**consolidated_id_day1_awakening**		text	Consolidated ID (participant id + sample id)_day1_Awakening
**biobank_status_day1_awakening**		text	Collection status of sample (MS is missing)_day1_Awakening
**date_day1_awakening**		text	Date of collection_day1_Awakening
**participant_id_day1_awakening**		text	Participant ID aka MI number_day1_Awakening
**sample_id_day1_awakening**		text	ID of unique sample, including sample type, timepoint, and aliquot number._day1_Awakening
**sample_type_day1_awakening**		text	Type of sample (plasma, serum, saliva)_day1_Awakening
**day_day1_awakening**		text	For awakening saliva, day of collection_day1_Awakening

**time_day1_awakening**		text	Timepoint of collection for both awakening and stress samples_day1_Awakening
**experiment_day1_awakening**		text	Category of sample (awakening, misc, stress, fasting)_day1_Awakening
**notes_day1_awakening**		text	Notes about the sample, in particular visual appearance._day1_Awakening
**ln_nd1_copies_ul_day1_awakening**		text	Natural log transformed nd1 copies/ul of sample (mtDNA)_day1_Awakening
**nd1_copies_ul_day1_awakening**		text	b2m copies/ul of sample (mtDNA)_day1_Awakening
**ln_b2m_copies_ul_day1_awakening**		text	Natural log transformed b2m copies/ul of sample (nDNA)_day1_Awakening
**b2m_copies_ul_day1_awakening**		text	b2m copies/ul of sample (nDNA)_day1_Awakening
**nd1_sd_day1_awakening**		text	standard deviation between two measures of nd1_day1_Awakening
**b2m_sd_day1_awakening**		text	standard deviation between two measures of b2m_day1_Awakening
**nd1_cv_day1_awakening**		text	coefficient of variation between two measures of nd1_day1_Awakening
**b2m_cv_day1_awakening**		text	coefficient of variation between two measures of b2m_day1_Awakening
**sample_number_day1_30_min**		text	unique number for each sample, consecutively numbered from 1-4224_day1_+30 min
**plate_number_day1_30_min**		text	Plate number of qpcr_day1_+30 min
**well_day1_30_min**		text	Well in which sample was plated_day1_+30 min
**consolidated_id_day1_30_min**		text	Consolidated ID (participant id + sample id)_day1_+30 min
**biobank_status_day1_30_min**		text	Collection status of sample (MS is missing)_day1_+30 min
**date_day1_30_min**		text	Date of collection_day1_+30 min

**participant_id_day1_30_min**		text	Participant ID aka MI number_day1_+30 min
**sample_id_day1_30_min**		text	ID of unique sample, including sample type, timepoint, and aliquot number._day1_+30 min
**sample_type_day1_30_min**		text	Type of sample (plasma, serum, saliva)_day1_+30 min
**day_day1_30_min**		text	For awakening saliva, day of collection_day1_+30 min
**time_day1_30_min**		text	Timepoint of collection for both awakening and stress samples_day1_+30 min
**experiment_day1_30_min**		text	Category of sample (awakening, misc, stress, fasting)_day1_+30 min
**notes_day1_30_min**		text	Notes about the sample, in particular visual appearance._day1_+30 min
**ln_nd1_copies_ul_day1_30_min**		text	Natural log transformed nd1 copies/ul of sample (mtDNA)_day1_+30 min
**nd1_copies_ul_day1_30_min**		text	b2m copies/ul of sample (mtDNA)_day1_+30 min
**ln_b2m_copies_ul_day1_30_min**		text	Natural log transformed b2m copies/ul of sample (nDNA)_day1_+30 min
**b2m_copies_ul_day1_30_min**		text	b2m copies/ul of sample (nDNA)_day1_+30 min
**nd1_sd_day1_30_min**		text	standard deviation between two measures of nd1_day1_+30 min
**b2m_sd_day1_30_min**		text	standard deviation between two measures of b2m_day1_+30 min
**nd1_cv_day1_30_min**		text	coefficient of variation between two measures of nd1_day1_+30 min
**b2m_cv_day1_30_min**		text	coefficient of variation between two measures of b2m_day1_+30 min
**sample_number_day1_45_min**		text	unique number for each sample, consecutively numbered from 1-4224_day1_+45 min

**plate_number_day1_45_min**		text	Plate number of qpcr_day1_+45 min
**well_day1_45_min**		text	Well in which sample was plated_day1_+45 min
**consolidated_id_day1_45_min**		text	Consolidated ID (participant id + sample id)_day1_+45 min
**biobank_status_day1_45_min**		text	Collection status of sample (MS is missing)_day1_+45 min
**date_day1_45_min**		text	Date of collection_day1_+45 min
**participant_id_day1_45_min**		text	Participant ID aka MI number_day1_+45 min
**sample_id_day1_45_min**		text	ID of unique sample, including sample type, timepoint, and aliquot number._day1_+45 min
**sample_type_day1_45_min**		text	Type of sample (plasma, serum, saliva)_day1_+45 min
**day_day1_45_min**		text	For awakening saliva, day of collection_day1_+45 min
**time_day1_45_min**		text	Timepoint of collection for both awakening and stress samples_day1_+45 min
**experiment_day1_45_min**		text	Category of sample (awakening, misc, stress, fasting)_day1_+45 min
**notes_day1_45_min**		text	Notes about the sample, in particular visual appearance._day1_+45 min
**ln_nd1_copies_ul_day1_45_min**		text	Natural log transformed nd1 copies/ul of sample (mtDNA)_day1_+45 min
**nd1_copies_ul_day1_45_min**		text	b2m copies/ul of sample (mtDNA)_day1_+45 min
**ln_b2m_copies_ul_day1_45_min**		text	Natural log transformed b2m copies/ul of sample (nDNA)_day1_+45 min
**b2m_copies_ul_day1_45_min**		text	b2m copies/ul of sample (nDNA)_day1_+45 min
**nd1_sd_day1_45_min**		text	standard deviation between two measures of nd1_day1_+45 min

**b2m_sd_day1_45_min**		text	standard deviation between two measures of b2m_day1_+45 min
**nd1_cv_day1_45_min**		text	coefficient of variation between two measures of nd1_day1_+45 min
**b2m_cv_day1_45_min**		text	coefficient of variation between two measures of b2m_day1_+45 min
**sample_number_day1_bedtime**		text	unique number for each sample, consecutively numbered from 1-4224_day1_Bedtime
**plate_number_day1_bedtime**		text	Plate number of qpcr_day1_Bedtime
**well_day1_bedtime**		text	Well in which sample was plated_day1_Bedtime
**consolidated_id_day1_bedtime**		text	Consolidated ID (participant id + sample id)_day1_Bedtime
**biobank_status_day1_bedtime**		text	Collection status of sample (MS is missing)_day1_Bedtime
**date_day1_bedtime**		text	Date of collection_day1_Bedtime
**participant_id_day1_bedtime**		text	Participant ID aka MI number_day1_Bedtime
**sample_id_day1_bedtime**		text	ID of unique sample, including sample type, timepoint, and aliquot number._day1_Bedtime
**sample_type_day1_bedtime**		text	Type of sample (plasma, serum, saliva)_day1_Bedtime
**day_day1_bedtime**		text	For awakening saliva, day of collection_day1_Bedtime
**time_day1_bedtime**		text	Timepoint of collection for both awakening and stress samples_day1_Bedtime
**experiment_day1_bedtime**		text	Category of sample (awakening, misc, stress, fasting)_day1_Bedtime
**notes_day1_bedtime**		text	Notes about the sample, in particular visual appearance._day1_Bedtime

**ln_nd1_copies_ul_day1_bedtime**		text	Natural log transformed nd1 copies/ul of sample (mtDNA)_day1_Bedtime
**nd1_copies_ul_day1_bedtime**		text	b2m copies/ul of sample (mtDNA)_day1_Bedtime
**ln_b2m_copies_ul_day1_bedtime**		text	Natural log transformed b2m copies/ul of sample (nDNA)_day1_Bedtime
**b2m_copies_ul_day1_bedtime**		text	b2m copies/ul of sample (nDNA)_day1_Bedtime
**nd1_sd_day1_bedtime**		text	standard deviation between two measures of nd1_day1_Bedtime
**b2m_sd_day1_bedtime**		text	standard deviation between two measures of b2m_day1_Bedtime
**nd1_cv_day1_bedtime**		text	coefficient of variation between two measures of nd1_day1_Bedtime
**b2m_cv_day1_bedtime**		text	coefficient of variation between two measures of b2m_day1_Bedtime
**sample_number_day2_awakening**		text	unique number for each sample, consecutively numbered from 1-4224_day2_Awakening
**plate_number_day2_awakening**		text	Plate number of qpcr_day2_Awakening
**well_day2_awakening**		text	Well in which sample was plated_day2_Awakening
**consolidated_id_day2_awakening**		text	Consolidated ID (participant id + sample id)_day2_Awakening
**biobank_status_day2_awakening**		text	Collection status of sample (MS is missing)_day2_Awakening
**date_day2_awakening**		text	Date of collection_day2_Awakening
**participant_id_day2_awakening**		text	Participant ID aka MI number_day2_Awakening
**sample_id_day2_awakening**		text	ID of unique sample, including sample type, timepoint, and aliquot number._day2_Awakening

**sample_type_day2_awakening**		text	Type of sample (plasma, serum, saliva)_day2_Awakening
**day_day2_awakening**		text	For awakening saliva, day of collection_day2_Awakening
**time_day2_awakening**		text	Timepoint of collection for both awakening and stress samples_day2_Awakening
**experiment_day2_awakening**		text	Category of sample (awakening, misc, stress, fasting)_day2_Awakening
**notes_day2_awakening**		text	Notes about the sample, in particular visual appearance._day2_Awakening
**ln_nd1_copies_ul_day2_awakening**		text	Natural log transformed nd1 copies/ul of sample (mtDNA)_day2_Awakening
**nd1_copies_ul_day2_awakening**		text	b2m copies/ul of sample (mtDNA)_day2_Awakening
**ln_b2m_copies_ul_day2_awakening**		text	Natural log transformed b2m copies/ul of sample (nDNA)_day2_Awakening
**b2m_copies_ul_day2_awakening**		text	b2m copies/ul of sample (nDNA)_day2_Awakening
**nd1_sd_day2_awakening**		text	standard deviation between two measures of nd1_day2_Awakening
**b2m_sd_day2_awakening**		text	standard deviation between two measures of b2m_day2_Awakening
**nd1_cv_day2_awakening**		text	coefficient of variation between two measures of nd1_day2_Awakening
**b2m_cv_day2_awakening**		text	coefficient of variation between two measures of b2m_day2_Awakening
**sample_number_day2_30_min**		text	unique number for each sample, consecutively numbered from 1-4224_day2_+30 min
**plate_number_day2_30_min**		text	Plate number of qpcr_day2_+30 min
**well_day2_30_min**		text	Well in which sample was plated_day2_+30 min
**consolidated_id_day2_30_min**		text	Consolidated ID (participant id + sample id)_day2_+30 min

**biobank_status_day2_30_min**		text	Collection status of sample (MS is missing)_day2_+30 min
**date_day2_30_min**		text	Date of collection_day2_+30 min
**participant_id_day2_30_min**		text	Participant ID aka MI number_day2_+30 min
**sample_id_day2_30_min**		text	ID of unique sample, including sample type, timepoint, and aliquot number._day2_+30 min
**sample_type_day2_30_min**		text	Type of sample (plasma, serum, saliva)_day2_+30 min
**day_day2_30_min**		text	For awakening saliva, day of collection_day2_+30 min
**time_day2_30_min**		text	Timepoint of collection for both awakening and stress samples_day2_+30 min
**experiment_day2_30_min**		text	Category of sample (awakening, misc, stress, fasting)_day2_+30 min
**notes_day2_30_min**		text	Notes about the sample, in particular visual appearance._day2_+30 min
**ln_nd1_copies_ul_day2_30_min**		text	Natural log transformed nd1 copies/ul of sample (mtDNA)_day2_+30 min
**nd1_copies_ul_day2_30_min**		text	b2m copies/ul of sample (mtDNA)_day2_+30 min
**ln_b2m_copies_ul_day2_30_min**		text	Natural log transformed b2m copies/ul of sample (nDNA)_day2_+30 min
**b2m_copies_ul_day2_30_min**		text	b2m copies/ul of sample (nDNA)_day2_+30 min
**nd1_sd_day2_30_min**		text	standard deviation between two measures of nd1_day2_+30 min
**b2m_sd_day2_30_min**		text	standard deviation between two measures of b2m_day2_+30 min
**nd1_cv_day2_30_min**		text	coefficient of variation between two measures of nd1_day2_+30 min
**b2m_cv_day2_30_min**		text	coefficient of variation between two measures of b2m_day2_+30 min

**sample_number_day2_45_min**		text	unique number for each sample, consecutively numbered from 1-4224_day2_+45 min
**plate_number_day2_45_min**		text	Plate number of qpcr_day2_+45 min
**well_day2_45_min**		text	Well in which sample was plated_day2_+45 min
**consolidated_id_day2_45_min**		text	Consolidated ID (participant id + sample id)_day2_+45 min
**biobank_status_day2_45_min**		text	Collection status of sample (MS is missing)_day2_+45 min
**date_day2_45_min**		text	Date of collection_day2_+45 min
**participant_id_day2_45_min**		text	Participant ID aka MI number_day2_+45 min
**sample_id_day2_45_min**		text	ID of unique sample, including sample type, timepoint, and aliquot number._day2_+45 min
**sample_type_day2_45_min**		text	Type of sample (plasma, serum, saliva)_day2_+45 min
**day_day2_45_min**		text	For awakening saliva, day of collection_day2_+45 min
**time_day2_45_min**		text	Timepoint of collection for both awakening and stress samples_day2_+45 min
**experiment_day2_45_min**		text	Category of sample (awakening, misc, stress, fasting)_day2_+45 min
**notes_day2_45_min**		text	Notes about the sample, in particular visual appearance._day2_+45 min
**ln_nd1_copies_ul_day2_45_min**		text	Natural log transformed nd1 copies/ul of sample (mtDNA)_day2_+45 min
**nd1_copies_ul_day2_45_min**		text	b2m copies/ul of sample (mtDNA)_day2_+45 min
**ln_b2m_copies_ul_day2_45_min**		text	Natural log transformed b2m copies/ul of sample (nDNA)_day2_+45 min

**b2m_copies_ul_day2_45_min**		text	b2m copies/ul of sample (nDNA)_day2_+45 min
**nd1_sd_day2_45_min**		text	standard deviation between two measures of nd1_day2_+45 min
**b2m_sd_day2_45_min**		text	standard deviation between two measures of b2m_day2_+45 min
**nd1_cv_day2_45_min**		text	coefficient of variation between two measures of nd1_day2_+45 min
**b2m_cv_day2_45_min**		text	coefficient of variation between two measures of b2m_day2_+45 min
**sample_number_day2_bedtime**		text	unique number for each sample, consecutively numbered from 1-4224_day2_Bedtime
**plate_number_day2_bedtime**		text	Plate number of qpcr_day2_Bedtime
**well_day2_bedtime**		text	Well in which sample was plated_day2_Bedtime
**consolidated_id_day2_bedtime**		text	Consolidated ID (participant id + sample id)_day2_Bedtime
**biobank_status_day2_bedtime**		text	Collection status of sample (MS is missing)_day2_Bedtime
**date_day2_bedtime**		text	Date of collection_day2_Bedtime
**participant_id_day2_bedtime**		text	Participant ID aka MI number_day2_Bedtime
**sample_id_day2_bedtime**		text	ID of unique sample, including sample type, timepoint, and aliquot number._day2_Bedtime
**sample_type_day2_bedtime**		text	Type of sample (plasma, serum, saliva)_day2_Bedtime
**day_day2_bedtime**		text	For awakening saliva, day of collection_day2_Bedtime
**time_day2_bedtime**		text	Timepoint of collection for both awakening and stress samples_day2_Bedtime

**experiment_day2_bedtime**		text	Category of sample (awakening, misc, stress, fasting)_day2_Bedtime
**notes_day2_bedtime**		text	Notes about the sample, in particular visual appearance._day2_Bedtime
**ln_nd1_copies_ul_day2_bedtime**		text	Natural log transformed nd1 copies/ul of sample (mtDNA)_day2_Bedtime
**nd1_copies_ul_day2_bedtime**		text	b2m copies/ul of sample (mtDNA)_day2_Bedtime
**ln_b2m_copies_ul_day2_bedtime**		text	Natural log transformed b2m copies/ul of sample (nDNA)_day2_Bedtime
**b2m_copies_ul_day2_bedtime**		text	b2m copies/ul of sample (nDNA)_day2_Bedtime
**nd1_sd_day2_bedtime**		text	standard deviation between two measures of nd1_day2_Bedtime
**b2m_sd_day2_bedtime**		text	standard deviation between two measures of b2m_day2_Bedtime
**nd1_cv_day2_bedtime**		text	coefficient of variation between two measures of nd1_day2_Bedtime
**b2m_cv_day2_bedtime**		text	coefficient of variation between two measures of b2m_day2_Bedtime
**sample_number_day3_awakening**		text	unique number for each sample, consecutively numbered from 1-4224_day3_Awakening
**plate_number_day3_awakening**		text	Plate number of qpcr_day3_Awakening
**well_day3_awakening**		text	Well in which sample was plated_day3_Awakening
**consolidated_id_day3_awakening**		text	Consolidated ID (participant id + sample id)_day3_Awakening
**biobank_status_day3_awakening**		text	Collection status of sample (MS is missing)_day3_Awakening
**date_day3_awakening**		text	Date of collection_day3_Awakening
**participant_id_day3_awakening**		text	Participant ID aka MI number_day3_Awakening

**sample_id_day3_awakening**		text	ID of unique sample, including sample type, timepoint, and aliquot number._day3_Awakening
**sample_type_day3_awakening**		text	Type of sample (plasma, serum, saliva)_day3_Awakening
**day_day3_awakening**		text	For awakening saliva, day of collection_day3_Awakening
**time_day3_awakening**		text	Timepoint of collection for both awakening and stress samples_day3_Awakening
**experiment_day3_awakening**		text	Category of sample (awakening, misc, stress, fasting)_day3_Awakening
**notes_day3_awakening**		text	Notes about the sample, in particular visual appearance._day3_Awakening
**ln_nd1_copies_ul_day3_awakening**		text	Natural log transformed nd1 copies/ul of sample (mtDNA)_day3_Awakening
**nd1_copies_ul_day3_awakening**		text	b2m copies/ul of sample (mtDNA)_day3_Awakening
**ln_b2m_copies_ul_day3_awakening**		text	Natural log transformed b2m copies/ul of sample (nDNA)_day3_Awakening
**b2m_copies_ul_day3_awakening**		text	b2m copies/ul of sample (nDNA)_day3_Awakening
**nd1_sd_day3_awakening**		text	standard deviation between two measures of nd1_day3_Awakening
**b2m_sd_day3_awakening**		text	standard deviation between two measures of b2m_day3_Awakening
**nd1_cv_day3_awakening**		text	coefficient of variation between two measures of nd1_day3_Awakening
**b2m_cv_day3_awakening**		text	coefficient of variation between two measures of b2m_day3_Awakening
**sample_number_day3_30_min**		text	unique number for each sample, consecutively numbered from 1-4224_day3_+30 min
**plate_number_day3_30_min**		text	Plate number of qpcr_day3_+30 min

**well_day3_30_min**		text	Well in which sample was plated_day3_+30 min
**consolidated_id_day3_30_min**		text	Consolidated ID (participant id + sample id)_day3_+30 min
**biobank_status_day3_30_min**		text	Collection status of sample (MS is missing)_day3_+30 min
**date_day3_30_min**		text	Date of collection_day3_+30 min
**participant_id_day3_30_min**		text	Participant ID aka MI number_day3_+30 min
**sample_id_day3_30_min**		text	ID of unique sample, including sample type, timepoint, and aliquot number._day3_+30 min
**sample_type_day3_30_min**		text	Type of sample (plasma, serum, saliva)_day3_+30 min
**day_day3_30_min**		text	For awakening saliva, day of collection_day3_+30 min
**time_day3_30_min**		text	Timepoint of collection for both awakening and stress samples_day3_+30 min
**experiment_day3_30_min**		text	Category of sample (awakening, misc, stress, fasting)_day3_+30 min
**notes_day3_30_min**		text	Notes about the sample, in particular visual appearance._day3_+30 min
**ln_nd1_copies_ul_day3_30_min**		text	Natural log transformed nd1 copies/ul of sample (mtDNA)_day3_+30 min
**nd1_copies_ul_day3_30_min**		text	b2m copies/ul of sample (mtDNA)_day3_+30 min
**ln_b2m_copies_ul_day3_30_min**		text	Natural log transformed b2m copies/ul of sample (nDNA)_day3_+30 min
**b2m_copies_ul_day3_30_min**		text	b2m copies/ul of sample (nDNA)_day3_+30 min
**nd1_sd_day3_30_min**		text	standard deviation between two measures of nd1_day3_+30 min
**b2m_sd_day3_30_min**		text	standard deviation between two measures of b2m_day3_+30 min

**nd1_cv_day3_30_min**		text	coefficient of variation between two measures of nd1_day3_+30 min
**b2m_cv_day3_30_min**		text	coefficient of variation between two measures of b2m_day3_+30 min
**sample_number_day3_45_min**		text	unique number for each sample, consecutively numbered from 1-4224_day3_+45 min
**plate_number_day3_45_min**		text	Plate number of qpcr_day3_+45 min
**well_day3_45_min**		text	Well in which sample was plated_day3_+45 min
**consolidated_id_day3_45_min**		text	Consolidated ID (participant id + sample id)_day3_+45 min
**biobank_status_day3_45_min**		text	Collection status of sample (MS is missing)_day3_+45 min
**date_day3_45_min**		text	Date of collection_day3_+45 min
**participant_id_day3_45_min**		text	Participant ID aka MI number_day3_+45 min
**sample_id_day3_45_min**		text	ID of unique sample, including sample type, timepoint, and aliquot number._day3_+45 min
**sample_type_day3_45_min**		text	Type of sample (plasma, serum, saliva)_day3_+45 min
**day_day3_45_min**		text	For awakening saliva, day of collection_day3_+45 min
**time_day3_45_min**		text	Timepoint of collection for both awakening and stress samples_day3_+45 min
**experiment_day3_45_min**		text	Category of sample (awakening, misc, stress, fasting)_day3_+45 min
**notes_day3_45_min**		text	Notes about the sample, in particular visual appearance._day3_+45 min
**ln_nd1_copies_ul_day3_45_min**		text	Natural log transformed nd1 copies/ul of sample (mtDNA)_day3_+45 min

**nd1_copies_ul_day3_45_min**		text	b2m copies/ul of sample (mtDNA)_day3_+45 min
**ln_b2m_copies_ul_day3_45_min**		text	Natural log transformed b2m copies/ul of sample (nDNA)_day3_+45 min
**b2m_copies_ul_day3_45_min**		text	b2m copies/ul of sample (nDNA)_day3_+45 min
**nd1_sd_day3_45_min**		text	standard deviation between two measures of nd1_day3_+45 min
**b2m_sd_day3_45_min**		text	standard deviation between two measures of b2m_day3_+45 min
**nd1_cv_day3_45_min**		text	coefficient of variation between two measures of nd1_day3_+45 min
**b2m_cv_day3_45_min**		text	coefficient of variation between two measures of b2m_day3_+45 min
**sample_number_day3_bedtime**		text	unique number for each sample, consecutively numbered from 1-4224_day3_Bedtime
**plate_number_day3_bedtime**		text	Plate number of qpcr_day3_Bedtime
**well_day3_bedtime**		text	Well in which sample was plated_day3_Bedtime
**consolidated_id_day3_bedtime**		text	Consolidated ID (participant id + sample id)_day3_Bedtime
**biobank_status_day3_bedtime**		text	Collection status of sample (MS is missing)_day3_Bedtime
**date_day3_bedtime**		text	Date of collection_day3_Bedtime
**participant_id_day3_bedtime**		text	Participant ID aka MI number_day3_Bedtime
**sample_id_day3_bedtime**		text	ID of unique sample, including sample type, timepoint, and aliquot number._day3_Bedtime
**sample_type_day3_bedtime**		text	Type of sample (plasma, serum, saliva)_day3_Bedtime

**day_day3_bedtime**		text	For awakening saliva, day of collection_day3_Bedtime
**time_day3_bedtime**		text	Timepoint of collection for both awakening and stress samples_day3_Bedtime
**experiment_day3_bedtime**		text	Category of sample (awakening, misc, stress, fasting)_day3_Bedtime
**notes_day3_bedtime**		text	Notes about the sample, in particular visual appearance._day3_Bedtime
**ln_nd1_copies_ul_day3_bedtime**		text	Natural log transformed nd1 copies/ul of sample (mtDNA)_day3_Bedtime
**nd1_copies_ul_day3_bedtime**		text	b2m copies/ul of sample (mtDNA)_day3_Bedtime
**ln_b2m_copies_ul_day3_bedtime**		text	Natural log transformed b2m copies/ul of sample (nDNA)_day3_Bedtime
**b2m_copies_ul_day3_bedtime**		text	b2m copies/ul of sample (nDNA)_day3_Bedtime
**nd1_sd_day3_bedtime**		text	standard deviation between two measures of nd1_day3_Bedtime
**b2m_sd_day3_bedtime**		text	standard deviation between two measures of b2m_day3_Bedtime
**nd1_cv_day3_bedtime**		text	coefficient of variation between two measures of nd1_day3_Bedtime
**b2m_cv_day3_bedtime**		text	coefficient of variation between two measures of b2m_day3_Bedtime
**sample_number_saliva_m5**	Experioment: Stress	text	unique number for each sample, consecutively numbered from 1-4224_saliva_m5
**plate_number_saliva_m5**		text	Plate number of qpcr_saliva_m5
**well_saliva_m5**		text	Well in which sample was plated_saliva_m5
**consolidated_id_saliva_m5**		text	Consolidated ID (participant id + sample id)_saliva_m5
**biobank_status_saliva_m5**		text	Collection status of sample (MS is missing)_saliva_m5

**date_saliva_m5**		text	Date of collection_saliva_m5
**participant_id_saliva_m5**		text	Participant ID aka MI number_saliva_m5
**sample_id_saliva_m5**		text	ID of unique sample, including sample type, timepoint, and aliquot number._saliva_m5
**sample_type_saliva_m5**		text	Type of sample (plasma, serum, saliva)_saliva_m5
**day_saliva_m5**		text	For awakening saliva, day of collection_saliva_m5
**time_saliva_m5**		text	Timepoint of collection for both awakening and stress samples_saliva_m5
**experiment_saliva_m5**		text	Category of sample (awakening, misc, stress, fasting)_saliva_m5
**notes_saliva_m5**		text	Notes about the sample, in particular visual appearance._saliva_m5
**ln_nd1_copies_ul_saliva_m5**		text	Natural log transformed nd1 copies/ul of sample (mtDNA)_saliva_m5
**nd1_copies_ul_saliva_m5**		text	b2m copies/ul of sample (mtDNA)_saliva_m5
**ln_b2m_copies_ul_saliva_m5**		text	Natural log transformed b2m copies/ul of sample (nDNA)_saliva_m5
**b2m_copies_ul_saliva_m5**		text	b2m copies/ul of sample (nDNA)_saliva_m5
**nd1_sd_saliva_m5**		text	standard deviation between two measures of nd1_saliva_m5
**b2m_sd_saliva_m5**		text	standard deviation between two measures of b2m_saliva_m5
**nd1_cv_saliva_m5**		text	coefficient of variation between two measures of nd1_saliva_m5
**b2m_cv_saliva_m5**		text	coefficient of variation between two measures of b2m_saliva_m5
**sample_number_saliva_5**		text	unique number for each sample, consecutively numbered from 1-4224_saliva_5

**plate_number_saliva_5**		text	Plate number of qpcr_saliva_5
**well_saliva_5**		text	Well in which sample was plated_saliva_5
**consolidated_id_saliva_5**		text	Consolidated ID (participant id + sample id)_saliva_5
**biobank_status_saliva_5**		text	Collection status of sample (MS is missing)_saliva_5
**date_saliva_5**		text	Date of collection_saliva_5
**participant_id_saliva_5**		text	Participant ID aka MI number_saliva_5
**sample_id_saliva_5**		text	ID of unique sample, including sample type, timepoint, and aliquot number._saliva_5
**sample_type_saliva_5**		text	Type of sample (plasma, serum, saliva)_saliva_5
**day_saliva_5**		text	For awakening saliva, day of collection_saliva_5
**time_saliva_5**		text	Timepoint of collection for both awakening and stress samples_saliva_5
**experiment_saliva_5**		text	Category of sample (awakening, misc, stress, fasting)_saliva_5
**notes_saliva_5**		text	Notes about the sample, in particular visual appearance._saliva_5
**ln_nd1_copies_ul_saliva_5**		text	Natural log transformed nd1 copies/ul of sample (mtDNA)_saliva_5
**nd1_copies_ul_saliva_5**		text	b2m copies/ul of sample (mtDNA)_saliva_5
**ln_b2m_copies_ul_saliva_5**		text	Natural log transformed b2m copies/ul of sample (nDNA)_saliva_5
**b2m_copies_ul_saliva_5**		text	b2m copies/ul of sample (nDNA)_saliva_5
**nd1_sd_saliva_5**		text	standard deviation between two measures of nd1_saliva_5

**b2m_sd_saliva_5**		text	standard deviation between two measures of b2m_saliva_5
**nd1_cv_saliva_5**		text	coefficient of variation between two measures of nd1_saliva_5
**b2m_cv_saliva_5**		text	coefficient of variation between two measures of b2m_saliva_5
**sample_number_saliva_10**		text	unique number for each sample, consecutively numbered from 1-4224_saliva_10
**plate_number_saliva_10**		text	Plate number of qpcr_saliva_10
**well_saliva_10**		text	Well in which sample was plated_saliva_10
**consolidated_id_saliva_10**		text	Consolidated ID (participant id + sample id)_saliva_10
**biobank_status_saliva_10**		text	Collection status of sample (MS is missing)_saliva_10
**date_saliva_10**		text	Date of collection_saliva_10
**participant_id_saliva_10**		text	Participant ID aka MI number_saliva_10
**sample_id_saliva_10**		text	ID of unique sample, including sample type, timepoint, and aliquot number._saliva_10
**sample_type_saliva_10**		text	Type of sample (plasma, serum, saliva)_saliva_10
**day_saliva_10**		text	For awakening saliva, day of collection_saliva_10
**time_saliva_10**		text	Timepoint of collection for both awakening and stress samples_saliva_10
**experiment_saliva_10**		text	Category of sample (awakening, misc, stress, fasting)_saliva_10
**notes_saliva_10**		text	Notes about the sample, in particular visual appearance._saliva_10
**ln_nd1_copies_ul_saliva_10**		text	Natural log transformed nd1 copies/ul of sample (mtDNA)_saliva_10

**nd1_copies_ul_saliva_10**		text	b2m copies/ul of sample (mtDNA)_saliva_10
**ln_b2m_copies_ul_saliva_10**		text	Natural log transformed b2m copies/ul of sample (nDNA)_saliva_10
**b2m_copies_ul_saliva_10**		text	b2m copies/ul of sample (nDNA)_saliva_10
**nd1_sd_saliva_10**		text	standard deviation between two measures of nd1_saliva_10
**b2m_sd_saliva_10**		text	standard deviation between two measures of b2m_saliva_10
**nd1_cv_saliva_10**		text	coefficient of variation between two measures of nd1_saliva_10
**b2m_cv_saliva_10**		text	coefficient of variation between two measures of b2m_saliva_10
**sample_number_saliva_20**		text	unique number for each sample, consecutively numbered from 1-4224_saliva_20
**plate_number_saliva_20**		text	Plate number of qpcr_saliva_20
**well_saliva_20**		text	Well in which sample was plated_saliva_20
**consolidated_id_saliva_20**		text	Consolidated ID (participant id + sample id)_saliva_20
**biobank_status_saliva_20**		text	Collection status of sample (MS is missing)_saliva_20
**date_saliva_20**		text	Date of collection_saliva_20
**participant_id_saliva_20**		text	Participant ID aka MI number_saliva_20
**sample_id_saliva_20**		text	ID of unique sample, including sample type, timepoint, and aliquot number._saliva_20
**sample_type_saliva_20**		text	Type of sample (plasma, serum, saliva)_saliva_20
**day_saliva_20**		text	For awakening saliva, day of collection_saliva_20

**time_saliva_20**		text	Timepoint of collection for both awakening and stress samples_saliva_20
**experiment_saliva_20**		text	Category of sample (awakening, misc, stress, fasting)_saliva_20
**notes_saliva_20**		text	Notes about the sample, in particular visual appearance._saliva_20
**ln_nd1_copies_ul_saliva_20**		text	Natural log transformed nd1 copies/ul of sample (mtDNA)_saliva_20
**nd1_copies_ul_saliva_20**		text	b2m copies/ul of sample (mtDNA)_saliva_20
**ln_b2m_copies_ul_saliva_20**		text	Natural log transformed b2m copies/ul of sample (nDNA)_saliva_20
**b2m_copies_ul_saliva_20**		text	b2m copies/ul of sample (nDNA)_saliva_20
**nd1_sd_saliva_20**		text	standard deviation between two measures of nd1_saliva_20
**b2m_sd_saliva_20**		text	standard deviation between two measures of b2m_saliva_20
**nd1_cv_saliva_20**		text	coefficient of variation between two measures of nd1_saliva_20
**b2m_cv_saliva_20**		text	coefficient of variation between two measures of b2m_saliva_20
**sample_number_saliva_30**		text	unique number for each sample, consecutively numbered from 1-4224_saliva_30
**plate_number_saliva_30**		text	Plate number of qpcr_saliva_30
**well_saliva_30**		text	Well in which sample was plated_saliva_30
**consolidated_id_saliva_30**		text	Consolidated ID (participant id + sample id)_saliva_30
**biobank_status_saliva_30**		text	Collection status of sample (MS is missing)_saliva_30
**date_saliva_30**		text	Date of collection_saliva_30

**participant_id_saliva_30**		text	Participant ID aka MI number_saliva_30
**sample_id_saliva_30**		text	ID of unique sample, including sample type, timepoint, and aliquot number._saliva_30
**sample_type_saliva_30**		text	Type of sample (plasma, serum, saliva)_saliva_30
**day_saliva_30**		text	For awakening saliva, day of collection_saliva_30
**time_saliva_30**		text	Timepoint of collection for both awakening and stress samples_saliva_30
**experiment_saliva_30**		text	Category of sample (awakening, misc, stress, fasting)_saliva_30
**notes_saliva_30**		text	Notes about the sample, in particular visual appearance._saliva_30
**ln_nd1_copies_ul_saliva_30**		text	Natural log transformed nd1 copies/ul of sample (mtDNA)_saliva_30
**nd1_copies_ul_saliva_30**		text	b2m copies/ul of sample (mtDNA)_saliva_30
**ln_b2m_copies_ul_saliva_30**		text	Natural log transformed b2m copies/ul of sample (nDNA)_saliva_30
**b2m_copies_ul_saliva_30**		text	b2m copies/ul of sample (nDNA)_saliva_30
**nd1_sd_saliva_30**		text	standard deviation between two measures of nd1_saliva_30
**b2m_sd_saliva_30**		text	standard deviation between two measures of b2m_saliva_30
**nd1_cv_saliva_30**		text	coefficient of variation between two measures of nd1_saliva_30
**b2m_cv_saliva_30**		text	coefficient of variation between two measures of b2m_saliva_30
**sample_number_saliva_60**		text	unique number for each sample, consecutively numbered from 1-4224_saliva_60
**plate_number_saliva_60**		text	Plate number of qpcr_saliva_60

**well_saliva_60**		text	Well in which sample was plated_saliva_60
**consolidated_id_saliva_60**		text	Consolidated ID (participant id + sample id)_saliva_60
**biobank_status_saliva_60**		text	Collection status of sample (MS is missing)_saliva_60
**date_saliva_60**		text	Date of collection_saliva_60
**participant_id_saliva_60**		text	Participant ID aka MI number_saliva_60
**sample_id_saliva_60**		text	ID of unique sample, including sample type, timepoint, and aliquot number._saliva_60
**sample_type_saliva_60**		text	Type of sample (plasma, serum, saliva)_saliva_60
**day_saliva_60**		text	For awakening saliva, day of collection_saliva_60
**time_saliva_60**		text	Timepoint of collection for both awakening and stress samples_saliva_60
**experiment_saliva_60**		text	Category of sample (awakening, misc, stress, fasting)_saliva_60
**notes_saliva_60**		text	Notes about the sample, in particular visual appearance._saliva_60
**ln_nd1_copies_ul_saliva_60**		text	Natural log transformed nd1 copies/ul of sample (mtDNA)_saliva_60
**nd1_copies_ul_saliva_60**		text	b2m copies/ul of sample (mtDNA)_saliva_60
**ln_b2m_copies_ul_saliva_60**		text	Natural log transformed b2m copies/ul of sample (nDNA)_saliva_60
**b2m_copies_ul_saliva_60**		text	b2m copies/ul of sample (nDNA)_saliva_60
**nd1_sd_saliva_60**		text	standard deviation between two measures of nd1_saliva_60
**b2m_sd_saliva_60**		text	standard deviation between two measures of b2m_saliva_60

**nd1_cv_saliva_60**		text	coefficient of variation between two measures of nd1_saliva_60
**b2m_cv_saliva_60**		text	coefficient of variation between two measures of b2m_saliva_60
**sample_number_saliva_90**		text	unique number for each sample, consecutively numbered from 1-4224_saliva_90
**plate_number_saliva_90**		text	Plate number of qpcr_saliva_90
**well_saliva_90**		text	Well in which sample was plated_saliva_90
**consolidated_id_saliva_90**		text	Consolidated ID (participant id + sample id)_saliva_90
**biobank_status_saliva_90**		text	Collection status of sample (MS is missing)_saliva_90
**date_saliva_90**		text	Date of collection_saliva_90
**participant_id_saliva_90**		text	Participant ID aka MI number_saliva_90
**sample_id_saliva_90**		text	ID of unique sample, including sample type, timepoint, and aliquot number._saliva_90
**sample_type_saliva_90**		text	Type of sample (plasma, serum, saliva)_saliva_90
**day_saliva_90**		text	For awakening saliva, day of collection_saliva_90
**time_saliva_90**		text	Timepoint of collection for both awakening and stress samples_saliva_90
**experiment_saliva_90**		text	Category of sample (awakening, misc, stress, fasting)_saliva_90
**notes_saliva_90**		text	Notes about the sample, in particular visual appearance._saliva_90
**ln_nd1_copies_ul_saliva_90**		text	Natural log transformed nd1 copies/ul of sample (mtDNA)_saliva_90
**nd1_copies_ul_saliva_90**		text	b2m copies/ul of sample (mtDNA)_saliva_90

**ln_b2m_copies_ul_saliva_90**		text	Natural log transformed b2m copies/ul of sample (nDNA)_saliva_90
**b2m_copies_ul_saliva_90**		text	b2m copies/ul of sample (nDNA)_saliva_90
**nd1_sd_saliva_90**		text	standard deviation between two measures of nd1_saliva_90
**b2m_sd_saliva_90**		text	standard deviation between two measures of b2m_saliva_90
**nd1_cv_saliva_90**		text	coefficient of variation between two measures of nd1_saliva_90
**b2m_cv_saliva_90**		text	coefficient of variation between two measures of b2m_saliva_90
**sample_number_saliva_120**		text	unique number for each sample, consecutively numbered from 1-4224_saliva_120
**plate_number_saliva_120**		text	Plate number of qpcr_saliva_120
**well_saliva_120**		text	Well in which sample was plated_saliva_120
**consolidated_id_saliva_120**		text	Consolidated ID (participant id + sample id)_saliva_120
**biobank_status_saliva_120**		text	Collection status of sample (MS is missing)_saliva_120
**date_saliva_120**		text	Date of collection_saliva_120
**participant_id_saliva_120**		text	Participant ID aka MI number_saliva_120
**sample_id_saliva_120**		text	ID of unique sample, including sample type, timepoint, and aliquot number._saliva_120
**sample_type_saliva_120**		text	Type of sample (plasma, serum, saliva)_saliva_120
**day_saliva_120**		text	For awakening saliva, day of collection_saliva_120
**time_saliva_120**		text	Timepoint of collection for both awakening and stress samples_saliva_120

**experiment_saliva_120**		text	Category of sample (awakening, misc, stress, fasting)_saliva_120
**notes_saliva_120**		text	Notes about the sample, in particular visual appearance._saliva_120
**ln_nd1_copies_ul_saliva_120**		text	Natural log transformed nd1 copies/ul of sample (mtDNA)_saliva_120
**nd1_copies_ul_saliva_120**		text	b2m copies/ul of sample (mtDNA)_saliva_120
**ln_b2m_copies_ul_saliva_120**		text	Natural log transformed b2m copies/ul of sample (nDNA)_saliva_120
**b2m_copies_ul_saliva_120**		text	b2m copies/ul of sample (nDNA)_saliva_120
**nd1_sd_saliva_120**		text	standard deviation between two measures of nd1_saliva_120
**b2m_sd_saliva_120**		text	standard deviation between two measures of b2m_saliva_120
**nd1_cv_saliva_120**		text	coefficient of variation between two measures of nd1_saliva_120
**b2m_cv_saliva_120**		text	coefficient of variation between two measures of b2m_saliva_120
**sample_number_serum_m5**		text	unique number for each sample, consecutively numbered from 1-4224_serum_m5
**plate_number_serum_m5**		text	Plate number of qpcr_serum_m5
**well_serum_m5**		text	Well in which sample was plated_serum_m5
**consolidated_id_serum_m5**		text	Consolidated ID (participant id + sample id)_serum_m5
**biobank_status_serum_m5**		text	Collection status of sample (MS is missing)_serum_m5
**date_serum_m5**		text	Date of collection_serum_m5
**participant_id_serum_m5**		text	Participant ID aka MI number_serum_m5

**sample_id_serum_m5**		text	ID of unique sample, including sample type, timepoint, and aliquot number._serum_m5
**sample_type_serum_m5**		text	Type of sample (plasma, serum, saliva)_serum_m5
**day_serum_m5**		text	For awakening saliva, day of collection_serum_m5
**time_serum_m5**		text	Timepoint of collection for both awakening and stress samples_serum_m5
**experiment_serum_m5**		text	Category of sample (awakening, misc, stress, fasting)_serum_m5
**notes_serum_m5**		text	Notes about the sample, in particular visual appearance._serum_m5
**ln_nd1_copies_ul_serum_m5**		text	Natural log transformed nd1 copies/ul of sample (mtDNA)_serum_m5
**nd1_copies_ul_serum_m5**		text	b2m copies/ul of sample (mtDNA)_serum_m5
**ln_b2m_copies_ul_serum_m5**		text	Natural log transformed b2m copies/ul of sample (nDNA)_serum_m5
**b2m_copies_ul_serum_m5**		text	b2m copies/ul of sample (nDNA)_serum_m5
**nd1_sd_serum_m5**		text	standard deviation between two measures of nd1_serum_m5
**b2m_sd_serum_m5**		text	standard deviation between two measures of b2m_serum_m5
**nd1_cv_serum_m5**		text	coefficient of variation between two measures of nd1_serum_m5
**b2m_cv_serum_m5**		text	coefficient of variation between two measures of b2m_serum_m5
**sample_number_serum_5**		text	unique number for each sample, consecutively numbered from 1-4224_serum_5
**plate_number_serum_5**		text	Plate number of qpcr_serum_5
**well_serum_5**		text	Well in which sample was plated_serum_5

**consolidated_id_serum_5**		text	Consolidated ID (participant id + sample id)_serum_5
**biobank_status_serum_5**		text	Collection status of sample (MS is missing)_serum_5
**date_serum_5**		text	Date of collection_serum_5
**participant_id_serum_5**		text	Participant ID aka MI number_serum_5
**sample_id_serum_5**		text	ID of unique sample, including sample type, timepoint, and aliquot number._serum_5
**sample_type_serum_5**		text	Type of sample (plasma, serum, saliva)_serum_5
**day_serum_5**		text	For awakening saliva, day of collection_serum_5
**time_serum_5**		text	Timepoint of collection for both awakening and stress samples_serum_5
**experiment_serum_5**		text	Category of sample (awakening, misc, stress, fasting)_serum_5
**notes_serum_5**		text	Notes about the sample, in particular visual appearance._serum_5
**ln_nd1_copies_ul_serum_5**		text	Natural log transformed nd1 copies/ul of sample (mtDNA)_serum_5
**nd1_copies_ul_serum_5**		text	b2m copies/ul of sample (mtDNA)_serum_5
**ln_b2m_copies_ul_serum_5**		text	Natural log transformed b2m copies/ul of sample (nDNA)_serum_5
**b2m_copies_ul_serum_5**		text	b2m copies/ul of sample (nDNA)_serum_5
**nd1_sd_serum_5**		text	standard deviation between two measures of nd1_serum_5
**b2m_sd_serum_5**		text	standard deviation between two measures of b2m_serum_5
**nd1_cv_serum_5**		text	coefficient of variation between two measures of nd1_serum_5

**b2m_cv_serum_5**		text	coefficient of variation between two measures of b2m_serum_5
**sample_number_serum_10**		text	unique number for each sample, consecutively numbered from 1-4224_serum_10
**plate_number_serum_10**		text	Plate number of qpcr_serum_10
**well_serum_10**		text	Well in which sample was plated_serum_10
**consolidated_id_serum_10**		text	Consolidated ID (participant id + sample id)_serum_10
**biobank_status_serum_10**		text	Collection status of sample (MS is missing)_serum_10
**date_serum_10**		text	Date of collection_serum_10
**participant_id_serum_10**		text	Participant ID aka MI number_serum_10
**sample_id_serum_10**		text	ID of unique sample, including sample type, timepoint, and aliquot number._serum_10
**sample_type_serum_10**		text	Type of sample (plasma, serum, saliva)_serum_10
**day_serum_10**		text	For awakening saliva, day of collection_serum_10
**time_serum_10**		text	Timepoint of collection for both awakening and stress samples_serum_10
**experiment_serum_10**		text	Category of sample (awakening, misc, stress, fasting)_serum_10
**notes_serum_10**		text	Notes about the sample, in particular visual appearance._serum_10
**ln_nd1_copies_ul_serum_10**		text	Natural log transformed nd1 copies/ul of sample (mtDNA)_serum_10
**nd1_copies_ul_serum_10**		text	b2m copies/ul of sample (mtDNA)_serum_10
**ln_b2m_copies_ul_serum_10**		text	Natural log transformed b2m copies/ul of sample (nDNA)_serum_10

**b2m_copies_ul_serum_10**		text	b2m copies/ul of sample (nDNA)_serum_10
**nd1_sd_serum_10**		text	standard deviation between two measures of nd1_serum_10
**b2m_sd_serum_10**		text	standard deviation between two measures of b2m_serum_10
**nd1_cv_serum_10**		text	coefficient of variation between two measures of nd1_serum_10
**b2m_cv_serum_10**		text	coefficient of variation between two measures of b2m_serum_10
**sample_number_serum_20**		text	unique number for each sample, consecutively numbered from 1-4224_serum_20
**plate_number_serum_20**		text	Plate number of qpcr_serum_20
**well_serum_20**		text	Well in which sample was plated_serum_20
**consolidated_id_serum_20**		text	Consolidated ID (participant id + sample id)_serum_20
**biobank_status_serum_20**		text	Collection status of sample (MS is missing)_serum_20
**date_serum_20**		text	Date of collection_serum_20
**participant_id_serum_20**		text	Participant ID aka MI number_serum_20
**sample_id_serum_20**		text	ID of unique sample, including sample type, timepoint, and aliquot number._serum_20
**sample_type_serum_20**		text	Type of sample (plasma, serum, saliva)_serum_20
**day_serum_20**		text	For awakening saliva, day of collection_serum_20
**time_serum_20**		text	Timepoint of collection for both awakening and stress samples_serum_20
**experiment_serum_20**		text	Category of sample (awakening, misc, stress, fasting)_serum_20

**notes_serum_20**		text	Notes about the sample, in particular visual appearance._serum_20
**ln_nd1_copies_ul_serum_20**		text	Natural log transformed nd1 copies/ul of sample (mtDNA)_serum_20
**nd1_copies_ul_serum_20**		text	b2m copies/ul of sample (mtDNA)_serum_20
**ln_b2m_copies_ul_serum_20**		text	Natural log transformed b2m copies/ul of sample (nDNA)_serum_20
**b2m_copies_ul_serum_20**		text	b2m copies/ul of sample (nDNA)_serum_20
**nd1_sd_serum_20**		text	standard deviation between two measures of nd1_serum_20
**b2m_sd_serum_20**		text	standard deviation between two measures of b2m_serum_20
**nd1_cv_serum_20**		text	coefficient of variation between two measures of nd1_serum_20
**b2m_cv_serum_20**		text	coefficient of variation between two measures of b2m_serum_20
**sample_number_serum_30**		text	unique number for each sample, consecutively numbered from 1-4224_serum_30
**plate_number_serum_30**		text	Plate number of qpcr_serum_30
**well_serum_30**		text	Well in which sample was plated_serum_30
**consolidated_id_serum_30**		text	Consolidated ID (participant id + sample id)_serum_30
**biobank_status_serum_30**		text	Collection status of sample (MS is missing)_serum_30
**date_serum_30**		text	Date of collection_serum_30
**participant_id_serum_30**		text	Participant ID aka MI number_serum_30
**sample_id_serum_30**		text	ID of unique sample, including sample type, timepoint, and aliquot number._serum_30

**sample_type_serum_30**		text	Type of sample (plasma, serum, saliva)_serum_30
**day_serum_30**		text	For awakening saliva, day of collection_serum_30
**time_serum_30**		text	Timepoint of collection for both awakening and stress samples_serum_30
**experiment_serum_30**		text	Category of sample (awakening, misc, stress, fasting)_serum_30
**notes_serum_30**		text	Notes about the sample, in particular visual appearance._serum_30
**ln_nd1_copies_ul_serum_30**		text	Natural log transformed nd1 copies/ul of sample (mtDNA)_serum_30
**nd1_copies_ul_serum_30**		text	b2m copies/ul of sample (mtDNA)_serum_30
**ln_b2m_copies_ul_serum_30**		text	Natural log transformed b2m copies/ul of sample (nDNA)_serum_30
**b2m_copies_ul_serum_30**		text	b2m copies/ul of sample (nDNA)_serum_30
**nd1_sd_serum_30**		text	standard deviation between two measures of nd1_serum_30
**b2m_sd_serum_30**		text	standard deviation between two measures of b2m_serum_30
**nd1_cv_serum_30**		text	coefficient of variation between two measures of nd1_serum_30
**b2m_cv_serum_30**		text	coefficient of variation between two measures of b2m_serum_30
**sample_number_serum_60**		text	unique number for each sample, consecutively numbered from 1-4224_serum_60
**plate_number_serum_60**		text	Plate number of qpcr_serum_60
**well_serum_60**		text	Well in which sample was plated_serum_60
**consolidated_id_serum_60**		text	Consolidated ID (participant id + sample id)_serum_60

**biobank_status_serum_60**		text	Collection status of sample (MS is missing)_serum_60
**date_serum_60**		text	Date of collection_serum_60
**participant_id_serum_60**		text	Participant ID aka MI number_serum_60
**sample_id_serum_60**		text	ID of unique sample, including sample type, timepoint, and aliquot number._serum_60
**sample_type_serum_60**		text	Type of sample (plasma, serum, saliva)_serum_60
**day_serum_60**		text	For awakening saliva, day of collection_serum_60
**time_serum_60**		text	Timepoint of collection for both awakening and stress samples_serum_60
**experiment_serum_60**		text	Category of sample (awakening, misc, stress, fasting)_serum_60
**notes_serum_60**		text	Notes about the sample, in particular visual appearance._serum_60
**ln_nd1_copies_ul_serum_60**		text	Natural log transformed nd1 copies/ul of sample (mtDNA)_serum_60
**nd1_copies_ul_serum_60**		text	b2m copies/ul of sample (mtDNA)_serum_60
**ln_b2m_copies_ul_serum_60**		text	Natural log transformed b2m copies/ul of sample (nDNA)_serum_60
**b2m_copies_ul_serum_60**		text	b2m copies/ul of sample (nDNA)_serum_60
**nd1_sd_serum_60**		text	standard deviation between two measures of nd1_serum_60
**b2m_sd_serum_60**		text	standard deviation between two measures of b2m_serum_60
**nd1_cv_serum_60**		text	coefficient of variation between two measures of nd1_serum_60
**b2m_cv_serum_60**		text	coefficient of variation between two measures of b2m_serum_60

**sample_number_serum_90**		text	unique number for each sample, consecutively numbered from 1-4224_serum_90
**plate_number_serum_90**		text	Plate number of qpcr_serum_90
**well_serum_90**		text	Well in which sample was plated_serum_90
**consolidated_id_serum_90**		text	Consolidated ID (participant id + sample id)_serum_90
**biobank_status_serum_90**		text	Collection status of sample (MS is missing)_serum_90
**date_serum_90**		text	Date of collection_serum_90
**participant_id_serum_90**		text	Participant ID aka MI number_serum_90
**sample_id_serum_90**		text	ID of unique sample, including sample type, timepoint, and aliquot number._serum_90
**sample_type_serum_90**		text	Type of sample (plasma, serum, saliva)_serum_90
**day_serum_90**		text	For awakening saliva, day of collection_serum_90
**time_serum_90**		text	Timepoint of collection for both awakening and stress samples_serum_90
**experiment_serum_90**		text	Category of sample (awakening, misc, stress, fasting)_serum_90
**notes_serum_90**		text	Notes about the sample, in particular visual appearance._serum_90
**ln_nd1_copies_ul_serum_90**		text	Natural log transformed nd1 copies/ul of sample (mtDNA)_serum_90
**nd1_copies_ul_serum_90**		text	b2m copies/ul of sample (mtDNA)_serum_90
**ln_b2m_copies_ul_serum_90**		text	Natural log transformed b2m copies/ul of sample (nDNA)_serum_90
**b2m_copies_ul_serum_90**		text	b2m copies/ul of sample (nDNA)_serum_90

**nd1_sd_serum_90**		text	standard deviation between two measures of nd1_serum_90
**b2m_sd_serum_90**		text	standard deviation between two measures of b2m_serum_90
**nd1_cv_serum_90**		text	coefficient of variation between two measures of nd1_serum_90
**b2m_cv_serum_90**		text	coefficient of variation between two measures of b2m_serum_90
**sample_number_serum_120**		text	unique number for each sample, consecutively numbered from 1-4224_serum_120
**plate_number_serum_120**		text	Plate number of qpcr_serum_120
**well_serum_120**		text	Well in which sample was plated_serum_120
**consolidated_id_serum_120**		text	Consolidated ID (participant id + sample id)_serum_120
**biobank_status_serum_120**		text	Collection status of sample (MS is missing)_serum_120
**date_serum_120**		text	Date of collection_serum_120
**participant_id_serum_120**		text	Participant ID aka MI number_serum_120
**sample_id_serum_120**		text	ID of unique sample, including sample type, timepoint, and aliquot number._serum_120
**sample_type_serum_120**		text	Type of sample (plasma, serum, saliva)_serum_120
**day_serum_120**		text	For awakening saliva, day of collection_serum_120
**time_serum_120**		text	Timepoint of collection for both awakening and stress samples_serum_120
**experiment_serum_120**		text	Category of sample (awakening, misc, stress, fasting)_serum_120
**notes_serum_120**		text	Notes about the sample, in particular visual appearance._serum_120

**ln_nd1_copies_ul_serum_120**		text	Natural log transformed nd1 copies/ul of sample (mtDNA)_serum_120
**nd1_copies_ul_serum_120**		text	b2m copies/ul of sample (mtDNA)_serum_120
**ln_b2m_copies_ul_serum_120**		text	Natural log transformed b2m copies/ul of sample (nDNA)_serum_120
**b2m_copies_ul_serum_120**		text	b2m copies/ul of sample (nDNA)_serum_120
**nd1_sd_serum_120**		text	standard deviation between two measures of nd1_serum_120
**b2m_sd_serum_120**		text	standard deviation between two measures of b2m_serum_120
**nd1_cv_serum_120**		text	coefficient of variation between two measures of nd1_serum_120
**b2m_cv_serum_120**		text	coefficient of variation between two measures of b2m_serum_120
**sample_number_plasma_m5**		text	unique number for each sample, consecutively numbered from 1-4224_plasma_m5
**plate_number_plasma_m5**		text	Plate number of qpcr_plasma_m5
**well_plasma_m5**		text	Well in which sample was plated_plasma_m5
**consolidated_id_plasma_m5**		text	Consolidated ID (participant id + sample id)_plasma_m5
**biobank_status_plasma_m5**		text	Collection status of sample (MS is missing)_plasma_m5
**date_plasma_m5**		text	Date of collection_plasma_m5
**participant_id_plasma_m5**		text	Participant ID aka MI number_plasma_m5
**sample_id_plasma_m5**		text	ID of unique sample, including sample type, timepoint, and aliquot number._plasma_m5
**sample_type_plasma_m5**		text	Type of sample (plasma, serum, saliva)_plasma_m5

**day_plasma_m5**		text	For awakening saliva, day of collection_plasma_m5
**time_plasma_m5**		text	Timepoint of collection for both awakening and stress samples_plasma_m5
**experiment_plasma_m5**		text	Category of sample (awakening, misc, stress, fasting)_plasma_m5
**notes_plasma_m5**		text	Notes about the sample, in particular visual appearance._plasma_m5
**ln_nd1_copies_ul_plasma_m5**		text	Natural log transformed nd1 copies/ul of sample (mtDNA)_plasma_m5
**nd1_copies_ul_plasma_m5**		text	b2m copies/ul of sample (mtDNA)_plasma_m5
**ln_b2m_copies_ul_plasma_m5**		text	Natural log transformed b2m copies/ul of sample (nDNA)_plasma_m5
**b2m_copies_ul_plasma_m5**		text	b2m copies/ul of sample (nDNA)_plasma_m5
**nd1_sd_plasma_m5**		text	standard deviation between two measures of nd1_plasma_m5
**b2m_sd_plasma_m5**		text	standard deviation between two measures of b2m_plasma_m5
**nd1_cv_plasma_m5**		text	coefficient of variation between two measures of nd1_plasma_m5
**b2m_cv_plasma_m5**		text	coefficient of variation between two measures of b2m_plasma_m5
**sample_number_plasma_5**		text	unique number for each sample, consecutively numbered from 1-4224_plasma_5
**plate_number_plasma_5**		text	Plate number of qpcr_plasma_5
**well_plasma_5**		text	Well in which sample was plated_plasma_5
**consolidated_id_plasma_5**		text	Consolidated ID (participant id + sample id)_plasma_5
**biobank_status_plasma_5**		text	Collection status of sample (MS is missing)_plasma_5

**date_plasma_5**		text	Date of collection_plasma_5
**participant_id_plasma_5**		text	Participant ID aka MI number_plasma_5
**sample_id_plasma_5**		text	ID of unique sample, including sample type, timepoint, and aliquot number._plasma_5
**sample_type_plasma_5**		text	Type of sample (plasma, serum, saliva)_plasma_5
**day_plasma_5**		text	For awakening saliva, day of collection_plasma_5
**time_plasma_5**		text	Timepoint of collection for both awakening and stress samples_plasma_5
**experiment_plasma_5**		text	Category of sample (awakening, misc, stress, fasting)_plasma_5
**notes_plasma_5**		text	Notes about the sample, in particular visual appearance._plasma_5
**ln_nd1_copies_ul_plasma_5**		text	Natural log transformed nd1 copies/ul of sample (mtDNA)_plasma_5
**nd1_copies_ul_plasma_5**		text	b2m copies/ul of sample (mtDNA)_plasma_5
**ln_b2m_copies_ul_plasma_5**		text	Natural log transformed b2m copies/ul of sample (nDNA)_plasma_5
**b2m_copies_ul_plasma_5**		text	b2m copies/ul of sample (nDNA)_plasma_5
**nd1_sd_plasma_5**		text	standard deviation between two measures of nd1_plasma_5
**b2m_sd_plasma_5**		text	standard deviation between two measures of b2m_plasma_5
**nd1_cv_plasma_5**		text	coefficient of variation between two measures of nd1_plasma_5
**b2m_cv_plasma_5**		text	coefficient of variation between two measures of b2m_plasma_5
**sample_number_plasma_10**		text	unique number for each sample, consecutively numbered from 1-4224_plasma_10

**plate_number_plasma_10**		text	Plate number of qpcr_plasma_10
**well_plasma_10**		text	Well in which sample was plated_plasma_10
**consolidated_id_plasma_10**		text	Consolidated ID (participant id + sample id)_plasma_10
**biobank_status_plasma_10**		text	Collection status of sample (MS is missing)_plasma_10
**date_plasma_10**		text	Date of collection_plasma_10
**participant_id_plasma_10**		text	Participant ID aka MI number_plasma_10
**sample_id_plasma_10**		text	ID of unique sample, including sample type, timepoint, and aliquot number._plasma_10
**sample_type_plasma_10**		text	Type of sample (plasma, serum, saliva)_plasma_10
**day_plasma_10**		text	For awakening saliva, day of collection_plasma_10
**time_plasma_10**		text	Timepoint of collection for both awakening and stress samples_plasma_10
**experiment_plasma_10**		text	Category of sample (awakening, misc, stress, fasting)_plasma_10
**notes_plasma_10**		text	Notes about the sample, in particular visual appearance._plasma_10
**ln_nd1_copies_ul_plasma_10**		text	Natural log transformed nd1 copies/ul of sample (mtDNA)_plasma_10
**nd1_copies_ul_plasma_10**		text	b2m copies/ul of sample (mtDNA)_plasma_10
**ln_b2m_copies_ul_plasma_10**		text	Natural log transformed b2m copies/ul of sample (nDNA)_plasma_10
**b2m_copies_ul_plasma_10**		text	b2m copies/ul of sample (nDNA)_plasma_10
**nd1_sd_plasma_10**		text	standard deviation between two measures of nd1_plasma_10

**b2m_sd_plasma_10**		text	standard deviation between two measures of b2m_plasma_10
**nd1_cv_plasma_10**		text	coefficient of variation between two measures of nd1_plasma_10
**b2m_cv_plasma_10**		text	coefficient of variation between two measures of b2m_plasma_10
**sample_number_plasma_20**		text	unique number for each sample, consecutively numbered from 1-4224_plasma_20
**plate_number_plasma_20**		text	Plate number of qpcr_plasma_20
**well_plasma_20**		text	Well in which sample was plated_plasma_20
**consolidated_id_plasma_20**		text	Consolidated ID (participant id + sample id)_plasma_20
**biobank_status_plasma_20**		text	Collection status of sample (MS is missing)_plasma_20
**date_plasma_20**		text	Date of collection_plasma_20
**participant_id_plasma_20**		text	Participant ID aka MI number_plasma_20
**sample_id_plasma_20**		text	ID of unique sample, including sample type, timepoint, and aliquot number._plasma_20
**sample_type_plasma_20**		text	Type of sample (plasma, serum, saliva)_plasma_20
**day_plasma_20**		text	For awakening saliva, day of collection_plasma_20
**time_plasma_20**		text	Timepoint of collection for both awakening and stress samples_plasma_20
**experiment_plasma_20**		text	Category of sample (awakening, misc, stress, fasting)_plasma_20
**notes_plasma_20**		text	Notes about the sample, in particular visual appearance._plasma_20
**ln_nd1_copies_ul_plasma_20**		text	Natural log transformed nd1 copies/ul of sample (mtDNA)_plasma_20

**nd1_copies_ul_plasma_20**		text	b2m copies/ul of sample (mtDNA)_plasma_20
**ln_b2m_copies_ul_plasma_20**		text	Natural log transformed b2m copies/ul of sample (nDNA)_plasma_20
**b2m_copies_ul_plasma_20**		text	b2m copies/ul of sample (nDNA)_plasma_20
**nd1_sd_plasma_20**		text	standard deviation between two measures of nd1_plasma_20
**b2m_sd_plasma_20**		text	standard deviation between two measures of b2m_plasma_20
**nd1_cv_plasma_20**		text	coefficient of variation between two measures of nd1_plasma_20
**b2m_cv_plasma_20**		text	coefficient of variation between two measures of b2m_plasma_20
**sample_number_plasma_30**		text	unique number for each sample, consecutively numbered from 1-4224_plasma_30
**plate_number_plasma_30**		text	Plate number of qpcr_plasma_30
**well_plasma_30**		text	Well in which sample was plated_plasma_30
**consolidated_id_plasma_30**		text	Consolidated ID (participant id + sample id)_plasma_30
**biobank_status_plasma_30**		text	Collection status of sample (MS is missing)_plasma_30
**date_plasma_30**		text	Date of collection_plasma_30
**participant_id_plasma_30**		text	Participant ID aka MI number_plasma_30
**sample_id_plasma_30**		text	ID of unique sample, including sample type, timepoint, and aliquot number._plasma_30
**sample_type_plasma_30**		text	Type of sample (plasma, serum, saliva)_plasma_30
**day_plasma_30**		text	For awakening saliva, day of collection_plasma_30

**time_plasma_30**		text	Timepoint of collection for both awakening and stress samples_plasma_30
**experiment_plasma_30**		text	Category of sample (awakening, misc, stress, fasting)_plasma_30
**notes_plasma_30**		text	Notes about the sample, in particular visual appearance._plasma_30
**ln_nd1_copies_ul_plasma_30**		text	Natural log transformed nd1 copies/ul of sample (mtDNA)_plasma_30
**nd1_copies_ul_plasma_30**		text	b2m copies/ul of sample (mtDNA)_plasma_30
**ln_b2m_copies_ul_plasma_30**		text	Natural log transformed b2m copies/ul of sample (nDNA)_plasma_30
**b2m_copies_ul_plasma_30**		text	b2m copies/ul of sample (nDNA)_plasma_30
**nd1_sd_plasma_30**		text	standard deviation between two measures of nd1_plasma_30
**b2m_sd_plasma_30**		text	standard deviation between two measures of b2m_plasma_30
**nd1_cv_plasma_30**		text	coefficient of variation between two measures of nd1_plasma_30
**b2m_cv_plasma_30**		text	coefficient of variation between two measures of b2m_plasma_30
**sample_number_plasma_60**		text	unique number for each sample, consecutively numbered from 1-4224_plasma_60
**plate_number_plasma_60**		text	Plate number of qpcr_plasma_60
**well_plasma_60**		text	Well in which sample was plated_plasma_60
**consolidated_id_plasma_60**		text	Consolidated ID (participant id + sample id)_plasma_60
**biobank_status_plasma_60**		text	Collection status of sample (MS is missing)_plasma_60
**date_plasma_60**		text	Date of collection_plasma_60

**participant_id_plasma_60**		text	Participant ID aka MI number_plasma_60
**sample_id_plasma_60**		text	ID of unique sample, including sample type, timepoint, and aliquot number._plasma_60
**sample_type_plasma_60**		text	Type of sample (plasma, serum, saliva)_plasma_60
**day_plasma_60**		text	For awakening saliva, day of collection_plasma_60
**time_plasma_60**		text	Timepoint of collection for both awakening and stress samples_plasma_60
**experiment_plasma_60**		text	Category of sample (awakening, misc, stress, fasting)_plasma_60
**notes_plasma_60**		text	Notes about the sample, in particular visual appearance._plasma_60
**ln_nd1_copies_ul_plasma_60**		text	Natural log transformed nd1 copies/ul of sample (mtDNA)_plasma_60
**nd1_copies_ul_plasma_60**		text	b2m copies/ul of sample (mtDNA)_plasma_60
**ln_b2m_copies_ul_plasma_60**		text	Natural log transformed b2m copies/ul of sample (nDNA)_plasma_60
**b2m_copies_ul_plasma_60**		text	b2m copies/ul of sample (nDNA)_plasma_60
**nd1_sd_plasma_60**		text	standard deviation between two measures of nd1_plasma_60
**b2m_sd_plasma_60**		text	standard deviation between two measures of b2m_plasma_60
**nd1_cv_plasma_60**		text	coefficient of variation between two measures of nd1_plasma_60
**b2m_cv_plasma_60**		text	coefficient of variation between two measures of b2m_plasma_60
**sample_number_plasma_90**		text	unique number for each sample, consecutively numbered from 1-4224_plasma_90
**plate_number_plasma_90**		text	Plate number of qpcr_plasma_90

**well_plasma_90**		text	Well in which sample was plated_plasma_90
**consolidated_id_plasma_90**		text	Consolidated ID (participant id + sample id)_plasma_90
**biobank_status_plasma_90**		text	Collection status of sample (MS is missing)_plasma_90
**date_plasma_90**		text	Date of collection_plasma_90
**participant_id_plasma_90**		text	Participant ID aka MI number_plasma_90
**sample_id_plasma_90**		text	ID of unique sample, including sample type, timepoint, and aliquot number._plasma_90
**sample_type_plasma_90**		text	Type of sample (plasma, serum, saliva)_plasma_90
**day_plasma_90**		text	For awakening saliva, day of collection_plasma_90
**time_plasma_90**		text	Timepoint of collection for both awakening and stress samples_plasma_90
**experiment_plasma_90**		text	Category of sample (awakening, misc, stress, fasting)_plasma_90
**notes_plasma_90**		text	Notes about the sample, in particular visual appearance._plasma_90
**ln_nd1_copies_ul_plasma_90**		text	Natural log transformed nd1 copies/ul of sample (mtDNA)_plasma_90
**nd1_copies_ul_plasma_90**		text	b2m copies/ul of sample (mtDNA)_plasma_90
**ln_b2m_copies_ul_plasma_90**		text	Natural log transformed b2m copies/ul of sample (nDNA)_plasma_90
**b2m_copies_ul_plasma_90**		text	b2m copies/ul of sample (nDNA)_plasma_90
**nd1_sd_plasma_90**		text	standard deviation between two measures of nd1_plasma_90
**b2m_sd_plasma_90**		text	standard deviation between two measures of b2m_plasma_90

**nd1_cv_plasma_90**		text	coefficient of variation between two measures of nd1_plasma_90
**b2m_cv_plasma_90**		text	coefficient of variation between two measures of b2m_plasma_90
**sample_number_plasma_120**		text	unique number for each sample, consecutively numbered from 1-4224_plasma_120
**plate_number_plasma_120**		text	Plate number of qpcr_plasma_120
**well_plasma_120**		text	Well in which sample was plated_plasma_120
**consolidated_id_plasma_120**		text	Consolidated ID (participant id + sample id)_plasma_120
**biobank_status_plasma_120**		text	Collection status of sample (MS is missing)_plasma_120
**date_plasma_120**		text	Date of collection_plasma_120
**participant_id_plasma_120**		text	Participant ID aka MI number_plasma_120
**sample_id_plasma_120**		text	ID of unique sample, including sample type, timepoint, and aliquot number._plasma_120
**sample_type_plasma_120**		text	Type of sample (plasma, serum, saliva)_plasma_120
**day_plasma_120**		text	For awakening saliva, day of collection_plasma_120
**time_plasma_120**		text	Timepoint of collection for both awakening and stress samples_plasma_120
**experiment_plasma_120**		text	Category of sample (awakening, misc, stress, fasting)_plasma_120
**notes_plasma_120**		text	Notes about the sample, in particular visual appearance._plasma_120
**ln_nd1_copies_ul_plasma_120**		text	Natural log transformed nd1 copies/ul of sample (mtDNA)_plasma_120
**nd1_copies_ul_plasma_120**		text	b2m copies/ul of sample (mtDNA)_plasma_120

**ln_b2m_copies_ul_plasma_120**		text	Natural log transformed b2m copies/ul of sample (nDNA)_plasma_120
**b2m_copies_ul_plasma_120**		text	b2m copies/ul of sample (nDNA)_plasma_120
**nd1_sd_plasma_120**		text	standard deviation between two measures of nd1_plasma_120
**b2m_sd_plasma_120**		text	standard deviation between two measures of b2m_plasma_120
**nd1_cv_plasma_120**		text	coefficient of variation between two measures of nd1_plasma_120
**b2m_cv_plasma_120**		text	coefficient of variation between two measures of b2m_plasma_120
**sample_number_serum_fasting**	Experiment: fasting	text	unique number for each sample, consecutively numbered from 1-4224_serum_fasting
**plate_number_serum_fasting**		text	Plate number of qpcr_serum_fasting
**well_serum_fasting**		text	Well in which sample was plated_serum_fasting
**consolidated_id_serum_fasting**		text	Consolidated ID (participant id + sample id)_serum_fasting
**biobank_status_serum_fasting**		text	Collection status of sample (MS is missing)_serum_fasting
**date_serum_fasting**		text	Date of collection_serum_fasting
**participant_id_serum_fasting**		text	Participant ID aka MI number_serum_fasting
**sample_id_serum_fasting**		text	ID of unique sample, including sample type, timepoint, and aliquot number._serum_fasting
**sample_type_serum_fasting**		text	Type of sample (plasma, serum, saliva)_serum_fasting
**day_serum_fasting**		text	For awakening saliva, day of collection_serum_fasting

**time_serum_fasting**		text	Timepoint of collection for both awakening and stress samples_serum_fasting
**experiment_serum_fasting**		text	Category of sample (awakening, misc, stress, fasting)_serum_fasting
**notes_serum_fasting**		text	Notes about the sample, in particular visual appearance._serum_fasting
**ln_nd1_copies_ul_serum_fasting**		text	Natural log transformed nd1 copies/ul of sample (mtDNA)_serum_fasting
**nd1_copies_ul_serum_fasting**		text	b2m copies/ul of sample (mtDNA)_serum_fasting
**ln_b2m_copies_ul_serum_fasting**		text	Natural log transformed b2m copies/ul of sample (nDNA)_serum_fasting
**b2m_copies_ul_serum_fasting**		text	b2m copies/ul of sample (nDNA)_serum_fasting
**nd1_sd_serum_fasting**		text	standard deviation between two measures of nd1_serum_fasting
**b2m_sd_serum_fasting**		text	standard deviation between two measures of b2m_serum_fasting
**nd1_cv_serum_fasting**		text	coefficient of variation between two measures of nd1_serum_fasting
**b2m_cv_serum_fasting**		text	coefficient of variation between two measures of b2m_serum_fasting
**sample_number_plasma_fasting**		text	unique number for each sample, consecutively numbered from 1-4224_plasma_fasting
**plate_number_plasma_fasting**		text	Plate number of qpcr_plasma_fasting
**well_plasma_fasting**		text	Well in which sample was plated_plasma_fasting
**consolidated_id_plasma_fasting**		text	Consolidated ID (participant id + sample id)_plasma_fasting
**biobank_status_plasma_fasting**		text	Collection status of sample (MS is missing)_plasma_fasting
**date_plasma_fasting**		text	Date of collection_plasma_fasting

**participant_id_plasma_fasting**		text	Participant ID aka MI number_plasma_fasting
**sample_id_plasma_fasting**		text	ID of unique sample, including sample type, timepoint, and aliquot number._plasma_fasting
**sample_type_plasma_fasting**		text	Type of sample (plasma, serum, saliva)_plasma_fasting
**day_plasma_fasting**		text	For awakening saliva, day of collection_plasma_fasting
**time_plasma_fasting**		text	Timepoint of collection for both awakening and stress samples_plasma_fasting
**experiment_plasma_fasting**		text	Category of sample (awakening, misc, stress, fasting)_plasma_fasting
**notes_plasma_fasting**		text	Notes about the sample, in particular visual appearance._plasma_fasting
**ln_nd1_copies_ul_plasma_fasting**		text	Natural log transformed nd1 copies/ul of sample (mtDNA)_plasma_fasting
**nd1_copies_ul_plasma_fasting**		text	b2m copies/ul of sample (mtDNA)_plasma_fasting
**ln_b2m_copies_ul_plasma_fasting**		text	Natural log transformed b2m copies/ul of sample (nDNA)_plasma_fasting
**b2m_copies_ul_plasma_fasting**		text	b2m copies/ul of sample (nDNA)_plasma_fasting
**nd1_sd_plasma_fasting**		text	standard deviation between two measures of nd1_plasma_fasting
**b2m_sd_plasma_fasting**		text	standard deviation between two measures of b2m_plasma_fasting
**nd1_cv_plasma_fasting**		text	coefficient of variation between two measures of nd1_plasma_fasting
**b2m_cv_plasma_fasting**		text	coefficient of variation between two measures of b2m_plasma_fasting
**sample_number_plasma_**		text	unique number for each sample, consecutively numbered from 1-4224_plasma_

**sample_number_saliva_day_1_fasting**	Experiment:nisc	text	unique number for each sample, consecutively numbered from 1-4224_saliva_Day 1 Fasting
**plate_number_saliva_day_1_fasting**		text	Plate number of qpcr_saliva_Day 1 Fasting
**well_saliva_day_1_fasting**		text	Well in which sample was plated_saliva_Day 1 Fasting
**consolidated_id_saliva_day_1_fasting**		text	Consolidated ID (participant id + sample id)_saliva_Day 1 Fasting
**biobank_status_saliva_day_1_fasting**		text	Collection status of sample (MS is missing)_saliva_Day 1 Fasting
**date_saliva_day_1_fasting**		text	Date of collection_saliva_Day 1 Fasting
**participant_id_saliva_day_1_fasting**		text	Participant ID aka MI number_saliva_Day 1 Fasting
**sample_id_saliva_day_1_fasting**		text	ID of unique sample, including sample type, timepoint, and aliquot number._saliva_Day 1 Fasting
**sample_type_saliva_day_1_fasting**		text	Type of sample (plasma, serum, saliva)_saliva_Day 1 Fasting
**day_saliva_day_1_fasting**		text	For awakening saliva, day of collection_saliva_Day 1 Fasting
**time_saliva_day_1_fasting**		text	Timepoint of collection for both awakening and stress samples_saliva_Day 1 Fasting
**experiment_saliva_day_1_fasting**		text	Category of sample (awakening, misc, stress, fasting)_saliva_Day 1 Fasting
**notes_saliva_day_1_fasting**		text	Notes about the sample, in particular visual appearance._saliva_Day 1 Fasting
**ln_nd1_copies_ul_saliva_day_1_fasting**		text	Natural log transformed nd1 copies/ul of sample (mtDNA)_saliva_Day 1 Fasting
**nd1_copies_ul_saliva_day_1_fasting**		text	b2m copies/ul of sample (mtDNA)_saliva_Day 1 Fasting

**ln_b2m_copies_ul_saliva_day_1_fasting**		text	Natural log transformed b2m copies/ul of sample (nDNA)_saliva_Day 1 Fasting
**b2m_copies_ul_saliva_day_1_fasting**		text	b2m copies/ul of sample (nDNA)_saliva_Day 1 Fasting
**nd1_sd_saliva_day_1_fasting**		text	standard deviation between two measures of nd1_saliva_Day 1 Fasting
**b2m_sd_saliva_day_1_fasting**		text	standard deviation between two measures of b2m_saliva_Day 1 Fasting
**nd1_cv_saliva_day_1_fasting**		text	coefficient of variation between two measures of nd1_saliva_Day 1 Fasting
**b2m_cv_saliva_day_1_fasting**		text	coefficient of variation between two measures of b2m_saliva_Day 1 Fasting
**sample_number_saliva_cold_pressor**		text	unique number for each sample, consecutively numbered from 1-4224_saliva_Cold Pressor
**plate_number_saliva_cold_pressor**		text	Plate number of qpcr_saliva_Cold Pressor
**well_saliva_cold_pressor**		text	Well in which sample was plated_saliva_Cold Pressor
**consolidated_id_saliva_cold_pressor**		text	Consolidated ID (participant id + sample id)_saliva_Cold Pressor
**biobank_status_saliva_cold_pressor**		text	Collection status of sample (MS is missing)_saliva_Cold Pressor
**date_saliva_cold_pressor**		text	Date of collection_saliva_Cold Pressor
**participant_id_saliva_cold_pressor**		text	Participant ID aka MI number_saliva_Cold Pressor
**sample_id_saliva_cold_pressor**		text	ID of unique sample, including sample type, timepoint, and aliquot number._saliva_Cold Pressor
**sample_type_saliva_cold_pressor**		text	Type of sample (plasma, serum, saliva)_saliva_Cold Pressor
**day_saliva_cold_pressor**		text	For awakening saliva, day of collection_saliva_Cold Pressor

**time_saliva_cold_pressor**		text	Timepoint of collection for both awakening and stress samples_saliva_Cold Pressor
**experiment_saliva_cold_pressor**		text	Category of sample (awakening, misc, stress, fasting)_saliva_Cold Pressor
**notes_saliva_cold_pressor**		text	Notes about the sample, in particular visual appearance._saliva_Cold Pressor
**ln_nd1_copies_ul_saliva_cold_pressor**		text	Natural log transformed nd1 copies/ul of sample (mtDNA)_saliva_Cold Pressor
**nd1_copies_ul_saliva_cold_pressor**		text	b2m copies/ul of sample (mtDNA)_saliva_Cold Pressor
**ln_b2m_copies_ul_saliva_cold_pressor**		text	Natural log transformed b2m copies/ul of sample (nDNA)_saliva_Cold Pressor
**b2m_copies_ul_saliva_cold_pressor**		text	b2m copies/ul of sample (nDNA)_saliva_Cold Pressor
**nd1_sd_saliva_cold_pressor**		text	standard deviation between two measures of nd1_saliva_Cold Pressor
**b2m_sd_saliva_cold_pressor**		text	standard deviation between two measures of b2m_saliva_Cold Pressor
**nd1_cv_saliva_cold_pressor**		text	coefficient of variation between two measures of nd1_saliva_Cold Pressor
**b2m_cv_saliva_cold_pressor**		text	coefficient of variation between two measures of b2m_saliva_Cold Pressor
**sample_number_saliva_day_2_fasting**		text	unique number for each sample, consecutively numbered from 1-4224_saliva_Day 2 Fasting
**plate_number_saliva_day_2_fasting**		text	Plate number of qpcr_saliva_Day 2 Fasting
**well_saliva_day_2_fasting**		text	Well in which sample was plated_saliva_Day 2 Fasting
**consolidated_id_saliva_day_2_fasting**		text	Consolidated ID (participant id + sample id)_saliva_Day 2 Fasting

**biobank_status_saliva_day_2_fasting**		text	Collection status of sample (MS is missing)_saliva_Day 2 Fasting
**date_saliva_day_2_fasting**		text	Date of collection_saliva_Day 2 Fasting
**participant_id_saliva_day_2_fasting**		text	Participant ID aka MI number_saliva_Day 2 Fasting
**sample_id_saliva_day_2_fasting**		text	ID of unique sample, including sample type, timepoint, and aliquot number._saliva_Day 2 Fasting
**sample_type_saliva_day_2_fasting**		text	Type of sample (plasma, serum, saliva)_saliva_Day 2 Fasting
**day_saliva_day_2_fasting**		text	For awakening saliva, day of collection_saliva_Day 2 Fasting
**time_saliva_day_2_fasting**		text	Timepoint of collection for both awakening and stress samples_saliva_Day 2 Fasting
**experiment_saliva_day_2_fasting**		text	Category of sample (awakening, misc, stress, fasting)_saliva_Day 2 Fasting
**notes_saliva_day_2_fasting**		text	Notes about the sample, in particular visual appearance._saliva_Day 2 Fasting
**ln_nd1_copies_ul_saliva_day_2_fasting**		text	Natural log transformed nd1 copies/ul of sample (mtDNA)_saliva_Day 2 Fasting
**nd1_copies_ul_saliva_day_2_fasting**		text	b2m copies/ul of sample (mtDNA)_saliva_Day 2 Fasting
**ln_b2m_copies_ul_saliva_day_2_fasting**		text	Natural log transformed b2m copies/ul of sample (nDNA)_saliva_Day 2 Fasting
**b2m_copies_ul_saliva_day_2_fasting**		text	b2m copies/ul of sample (nDNA)_saliva_Day 2 Fasting
**nd1_sd_saliva_day_2_fasting**		text	standard deviation between two measures of nd1_saliva_Day 2 Fasting
**b2m_sd_saliva_day_2_fasting**		text	standard deviation between two measures of b2m_saliva_Day 2 Fasting

**nd1_cv_saliva_day_2_fasting**		text	coefficient of variation between two measures of nd1_saliva_Day 2 Fasting
**b2m_cv_saliva_day_2_fasting**		text	coefficient of variation between two measures of b2m_saliva_Day 2 Fasting
**sample_number_saliva_mri_1**		text	unique number for each sample, consecutively numbered from 1-4224_saliva_MRI 1
**plate_number_saliva_mri_1**		text	Plate number of qpcr_saliva_MRI 1
**well_saliva_mri_1**		text	Well in which sample was plated_saliva_MRI 1
**consolidated_id_saliva_mri_1**		text	Consolidated ID (participant id + sample id)_saliva_MRI 1
**biobank_status_saliva_mri_1**		text	Collection status of sample (MS is missing)_saliva_MRI 1
**date_saliva_mri_1**		text	Date of collection_saliva_MRI 1
**participant_id_saliva_mri_1**		text	Participant ID aka MI number_saliva_MRI 1
**sample_id_saliva_mri_1**		text	ID of unique sample, including sample type, timepoint, and aliquot number._saliva_MRI 1
**sample_type_saliva_mri_1**		text	Type of sample (plasma, serum, saliva)_saliva_MRI 1
**day_saliva_mri_1**		text	For awakening saliva, day of collection_saliva_MRI 1
**time_saliva_mri_1**		text	Timepoint of collection for both awakening and stress samples_saliva_MRI 1
**experiment_saliva_mri_1**		text	Category of sample (awakening, misc, stress, fasting)_saliva_MRI 1
**notes_saliva_mri_1**		text	Notes about the sample, in particular visual appearance._saliva_MRI 1
**ln_nd1_copies_ul_saliva_mri_1**		text	Natural log transformed nd1 copies/ul of sample (mtDNA)_saliva_MRI 1
**nd1_copies_ul_saliva_mri_1**		text	b2m copies/ul of sample (mtDNA)_saliva_MRI 1

**ln_b2m_copies_ul_saliva_mri_1**		text	Natural log transformed b2m copies/ul of sample (nDNA)_saliva_MRI 1
**b2m_copies_ul_saliva_mri_1**		text	b2m copies/ul of sample (nDNA)_saliva_MRI 1
**nd1_sd_saliva_mri_1**		text	standard deviation between two measures of nd1_saliva_MRI 1
**b2m_sd_saliva_mri_1**		text	standard deviation between two measures of b2m_saliva_MRI 1
**nd1_cv_saliva_mri_1**		text	coefficient of variation between two measures of nd1_saliva_MRI 1
**b2m_cv_saliva_mri_1**		text	coefficient of variation between two measures of b2m_saliva_MRI 1
**sample_number_saliva_mri_2**		text	unique number for each sample, consecutively numbered from 1-4224_saliva_MRI 2
**plate_number_saliva_mri_2**		text	Plate number of qpcr_saliva_MRI 2
**well_saliva_mri_2**		text	Well in which sample was plated_saliva_MRI 2
**consolidated_id_saliva_mri_2**		text	Consolidated ID (participant id + sample id)_saliva_MRI 2
**biobank_status_saliva_mri_2**		text	Collection status of sample (MS is missing)_saliva_MRI 2
**date_saliva_mri_2**		text	Date of collection_saliva_MRI 2
**participant_id_saliva_mri_2**		text	Participant ID aka MI number_saliva_MRI 2
**sample_id_saliva_mri_2**		text	ID of unique sample, including sample type, timepoint, and aliquot number._saliva_MRI 2
**sample_type_saliva_mri_2**		text	Type of sample (plasma, serum, saliva)_saliva_MRI 2
**day_saliva_mri_2**		text	For awakening saliva, day of collection_saliva_MRI 2
**time_saliva_mri_2**		text	Timepoint of collection for both awakening and stress samples_saliva_MRI 2

**experiment_saliva_mri_2**		text	Category of sample (awakening, misc, stress, fasting)_saliva_MRI 2
**notes_saliva_mri_2**		text	Notes about the sample, in particular visual appearance._saliva_MRI 2
**ln_nd1_copies_ul_saliva_mri_2**		text	Natural log transformed nd1 copies/ul of sample (mtDNA)_saliva_MRI 2
**nd1_copies_ul_saliva_mri_2**		text	b2m copies/ul of sample (mtDNA)_saliva_MRI 2
**ln_b2m_copies_ul_saliva_mri_2**		text	Natural log transformed b2m copies/ul of sample (nDNA)_saliva_MRI 2
**b2m_copies_ul_saliva_mri_2**		text	b2m copies/ul of sample (nDNA)_saliva_MRI 2
**nd1_sd_saliva_mri_2**		text	standard deviation between two measures of nd1_saliva_MRI 2
**b2m_sd_saliva_mri_2**		text	standard deviation between two measures of b2m_saliva_MRI 2
**nd1_cv_saliva_mri_2**		text	coefficient of variation between two measures of nd1_saliva_MRI 2
**b2m_cv_saliva_mri_2**		text	coefficient of variation between two measures of b2m_saliva_MRI 2
**sample_number_saliva_mri_3**		text	unique number for each sample, consecutively numbered from 1-4224_saliva_MRI 3
**plate_number_saliva_mri_3**		text	Plate number of qpcr_saliva_MRI 3
**well_saliva_mri_3**		text	Well in which sample was plated_saliva_MRI 3
**consolidated_id_saliva_mri_3**		text	Consolidated ID (participant id + sample id)_saliva_MRI 3
**biobank_status_saliva_mri_3**		text	Collection status of sample (MS is missing)_saliva_MRI 3
**date_saliva_mri_3**		text	Date of collection_saliva_MRI 3
**participant_id_saliva_mri_3**		text	Participant ID aka MI number_saliva_MRI 3

**sample_id_saliva_mri_3**		text	ID of unique sample, including sample type, timepoint, and aliquot number._saliva_MRI 3
**sample_type_saliva_mri_3**		text	Type of sample (plasma, serum, saliva)_saliva_MRI 3
**day_saliva_mri_3**		text	For awakening saliva, day of collection_saliva_MRI 3
**time_saliva_mri_3**		text	Timepoint of collection for both awakening and stress samples_saliva_MRI 3
**experiment_saliva_mri_3**		text	Category of sample (awakening, misc, stress, fasting)_saliva_MRI 3
**notes_saliva_mri_3**		text	Notes about the sample, in particular visual appearance._saliva_MRI 3
**ln_nd1_copies_ul_saliva_mri_3**		text	Natural log transformed nd1 copies/ul of sample (mtDNA)_saliva_MRI 3
**nd1_copies_ul_saliva_mri_3**		text	b2m copies/ul of sample (mtDNA)_saliva_MRI 3
**ln_b2m_copies_ul_saliva_mri_3**		text	Natural log transformed b2m copies/ul of sample (nDNA)_saliva_MRI 3
**b2m_copies_ul_saliva_mri_3**		text	b2m copies/ul of sample (nDNA)_saliva_MRI 3
**nd1_sd_saliva_mri_3**		text	standard deviation between two measures of nd1_saliva_MRI 3
**b2m_sd_saliva_mri_3**		text	standard deviation between two measures of b2m_saliva_MRI 3
**nd1_cv_saliva_mri_3**		text	coefficient of variation between two measures of nd1_saliva_MRI 3
**b2m_cv_saliva_mri_3**		text	coefficient of variation between two measures of b2m_saliva_MRI 3

1.5.5 Catecholamines

RedCAP Form Name: calm_lab

Description: Center for Advanced Laboratory Medicine (CALM) lab results from morning and afternoon blood samples.

Variable / Field Name	Section Header	Field Type	Field Label
**date_sers11**	Ser-S-1.1	text	date
**sample_type_sers11**		text	sample_type
**norepinephrine_pgml_sers11**		text	norepinephrine_pgml
**epinephrine_pgml_sers11**		text	epinephrine_pgml
**dopamine_pgml_sers11**		text	dopamine_pgml
**serotonin_pgml_sers11**		text	serotonin_pgml
**date_sers21**	Ser-S-2.1	text	date
**sample_type_sers21**		text	sample_type
**norepinephrine_pgml_sers21**		text	norepinephrine_pgml
**epinephrine_pgml_sers21**		text	epinephrine_pgml
**dopamine_pgml_sers21**		text	dopamine_pgml
**serotonin_pgml_sers21**		text	serotonin_pgml
**date_sers31**	Ser-S-3.1	text	date
**sample_type_sers31**		text	sample_type
**norepinephrine_pgml_sers31**		text	norepinephrine_pgml
**epinephrine_pgml_sers31**		text	epinephrine_pgml

**dopamine_pgml_sers31**		text	dopamine_pgml
**serotonin_pgml_sers31**		text	serotonin_pgml
**date_sers41**	Ser-S-4.1	text	date
**sample_type_sers41**		text	sample_type
**norepinephrine_pgml_sers41**		text	norepinephrine_pgml
**epinephrine_pgml_sers41**		text	epinephrine_pgml
**dopamine_pgml_sers41**		text	dopamine_pgml
**serotonin_pgml_sers41**		text	serotonin_pgml
**date_sers51**	Ser-S-5.1	text	date
**sample_type_sers51**		text	sample_type
**norepinephrine_pgml_sers51**		text	norepinephrine_pgml
**epinephrine_pgml_sers51**		text	epinephrine_pgml
**dopamine_pgml_sers51**		text	dopamine_pgml
**serotonin_pgml_sers51**		text	serotonin_pgml
**date_sers61**	Ser-S-6.1	text	date
**sample_type_sers61**		text	sample_type
**norepinephrine_pgml_sers61**		text	norepinephrine_pgml

**epinephrine_pgml_sers61**		text	epinephrine_pgml
**dopamine_pgml_sers61**		text	dopamine_pgml
**serotonin_pgml_sers61**		text	serotonin_pgml
**date_sers71**	Ser-S-7.1	text	date
**sample_type_sers71**		text	sample_type
**norepinephrine_pgml_sers71**		text	norepinephrine_pgml
**epinephrine_pgml_sers71**		text	epinephrine_pgml
**dopamine_pgml_sers71**		text	dopamine_pgml
**serotonin_pgml_sers71**		text	serotonin_pgml
**date_sers81**	Ser-S-8.1	text	date
**sample_type_sers81**		text	sample_type
**norepinephrine_pgml_sers81**		text	norepinephrine_pgml
**epinephrine_pgml_sers81**		text	epinephrine_pgml
**dopamine_pgml_sers81**		text	dopamine_pgml
**serotonin_pgml_sers81**		text	serotonin_pgml
**urine_date**	urine	text	urine_date
**urine_norepinephrine_ngml**		text	urine_norepinephrine_ngml

**urine_epinephrine_ngml**		text	urine_epinephrine_ngml
**urine_dopamine_ngml**		text	urine_dopamine_ngml
**urine_serotonin_ngml**		text	urine_serotonin_ngml

1.6 Psychophysiological Results

1.6.1 Spectral

RedCAP Form Name: spectral

Description: Heart rate, systolic blood pressure, diastolic blood pressure.

Variable / Field Name	Section Header	Field Type	Field Label
**hr_period_b_300s**		text	Period
**hr_epoch_b_300s**		text	Epoch
**hr_duration_b_300s**		text	Duration
**hr_start_b_300s**		text	Start
**hr_end_b_300s**		text	End
**hr_max_time_b_300s**		text	Max_Time
**hr_inttot_b_300s**		text	IntervalsTotal
**hr_intused_b_300s**		text	IntervalsUsed
**hr_avg_b_300s**		text	Average Heart Rate (bpm)
**hr_sd_b_300s**		text	SD

**hr_avd_b_300s**		text	Avd
**hr_rms_b_300s**		text	Sqr(mssd)
**hr_60_medrr_b_300s**		text	60/medrr
**hr_iqr_b_300s**		text	iqrange
**hr_lfv_b_300s**		text	Low Frequency Heart Rate Variability
**hr_hfv_b_300s**		text	High Frequency Heart Rate Variability
**sbp_intav_b_300s**	300 second epoch-- SBP Base	text	IntervalAv
**sbp_avd_b_300s**		text	Avd
**sbp_lfv_b_300s**		text	SpectralBand:0.04:0.15
**sbp_hfv_b_300s**		text	SpectralBand:0.15:0.4
**dbp_intav_b_300s**	300 second epoch-- DBP Base	text	IntervalAv
**dbp_avd_b_300s**		text	Avd
**dbp_lfv_b_300s**		text	SpectralBand:0.04:0.15
**dbp_hfv_b_300s**		text	SpectralBand:0.15:0.4
**hr_time_instruct_300s**	300 second epoch--	text	Time

	ECG Instruct		
**hr_period_instruct_300s**		text	Period
**hr_epoch_instruct_300s**		text	Epoch
**hr_duration_instruct_300s**		text	Duration
**hr_start_instruct_300s**		text	Start
**hr_end_instruct_300s**		text	End
**hr_max_time_instruct_300s**	text	Max_Time	
**hr_inttot_instruct_300s**		text	IntervalsTotal
**hr_intused_instruct_300s**		text	IntervalsUsed
**hr_avg_instruct_300s**		text	Average Heart Rate (bpm)
**hr_sd_instruct_300s**		text	SD
**hr_avd_instruct_300s**		text	Avd
**hr_rms_instruct_300s**		text	Sqr(mssd)
**hr_60_medrr_instruct_300s**	text	60/medrr	
**hr_iqr_instruct_300s**		text	iqrange
**hr_lfv_instruct_300s**		text	Low Frequency Heart Rate Variability
**hr_hfv_instruct_300s**		text	High Frequency Heart Rate Variability

**sbp_intav_instruct_300s**	300 second epoch-- SBP Instruct	text	IntervalAv
**sbp_avd_instruct_300s**		text	Avd
**sbp_lfv_instruct_300s**		text	SpectralBand:0.04:0.15
**sbp_hfv_instruct_300s**		text	SpectralBand:0.15:0.4
**dbp_intav_instruct_300s**	300 second epoch-- DBP Instruct	text	IntervalAv
**dbp_avd_instruct_300s**		text	Avd
**dbp_lfv_instruct_300s**		text	SpectralBand:0.04:0.15
**dbp_hfv_instruct_300s**		text	SpectralBand:0.15:0.4
**hr_time_mr_300s**	300 second epoch-- ECG MR	text	Time
**hr_period_mr_300s**		text	Period
**hr_epoch_mr_300s**		text	Epoch
**hr_duration_mr_300s**		text	Duration
**hr_start_mr_300s**		text	Start
**hr_end_mr_300s**		text	End

**hr_max_time_mr_300s**		text	Max_Time
**hr_inttot_mr_300s**		text	IntervalsTotal
**hr_intused_mr_300s**		text	IntervalsUsed
**hr_avg_mr_300s**		text	Average Heart Rate (bpm)
**hr_sd_mr_300s**		text	SD
**hr_avd_mr_300s**		text	Avd
**hr_rms_mr_300s**		text	Sqr(mssd)
**hr_60_medrr_mr_300s**		text	60/medrr
**hr_iqr_mr_300s**		text	iqrange
**hr_lfv_mr_300s**		text	Low Frequency Heart Rate Variability
**hr_hfv_mr_300s**		text	High Frequency Heart Rate Variability
**sbp_intav_mr_300s**	300 second epoch--SBP MR	text	IntervalAv
**sbp_avd_mr_300s**		text	Avd
**sbp_lfv_mr_300s**		text	SpectralBand:0.04:0.15
**dbp_intav_mr_300s**	300 second epoch--DBP MR	text	IntervalAv

**dbp_avd_mr_300s**		text	Avd
**dbp_lfv_mr_300s**		text	SpectralBand:0.04:0.15
**hr_time_pr_300s**	300 second epoch-- ECG Pre	text	Time
**hr_period_pr_300s**		text	Period
**hr_epoch_pr_300s**		text	Epoch
**hr_duration_pr_300s**		text	Duration
**hr_start_pr_300s**		text	Start
**hr_end_pr_300s**		text	End
**hr_max_time_pr_300s**		text	Max_Time
**hr_inttot_pr_300s**		text	IntervalsTotal
**hr_intused_pr_300s**		text	IntervalsUsed
**hr_avg_pr_300s**		text	Average Heart Rate (bpm)
**hr_sd_pr_300s**		text	SD
**hr_avd_pr_300s**		text	Avd
**hr_rms_pr_300s**		text	Sqr(mssd)
**hr_60_medrr_pr_300s**		text	60/medrr

**hr_iqr_pr_300s**		text	iqrange
**hr_lfv_pr_300s**		text	Low Frequency Heart Rate Variability
**hr_hfv_pr_300s**		text	High Frequency Heart Rate Variability
**sbp_intav_pr_300s**	300 second epoch--SBP Pre	text	IntervalAv
**sbp_avd_pr_300s**		text	Avd
**sbp_lfv_pr_300s**		text	SpectralBand:0.04:0.15
**dbp_intav_pr_300s**	300 second epoch--DBP Pre	text	IntervalAv
**dbp_avd_pr_300s**		text	Avd
**dbp_lfv_pr_300s**		text	SpectralBand:0.04:0.15
**hr_time_prt_300s**	300 second epoch--ECG Pretask	text	Time
**hr_period_prt_300s**		text	Period
**hr_epoch_prt_300s**		text	Epoch
**hr_duration_prt_300s**		text	Duration
**hr_start_prt_300s**		text	Start

**hr_end_prt_300s**		text	End
**hr_max_time_prt_300s**		text	Max_Time
**hr_inttot_prt_300s**		text	IntervalsTotal
**hr_intused_prt_300s**		text	IntervalsUsed
**hr_avg_prt_300s**		text	Average Heart Rate (bpm)
**hr_sd_prt_300s**		text	SD
**hr_avd_prt_300s**		text	Avd
**hr_rms_prt_300s**		text	Sqr(mssd)
**hr_60_medrr_prt_300s**		text	60/medrr
**hr_iqr_prt_300s**		text	iqrange
**hr_lfv_prt_300s**		text	Low Frequency Heart Rate Variability
**hr_hfv_prt_300s**		text	High Frequency Heart Rate Variability
**sbp_intav_prt_300s**	300 second epoch-- SBP Pretask	text	IntervalAv
**sbp_avd_prt_300s**		text	Avd
**sbp_lfv_prt_300s**		text	SpectralBand:0.04:0.15
**dbp_intav_prt_300s**	300 second epoch--	text	IntervalAv

	DBP Pretask		
**dbp_avd_prt_300s**		text	Avd
**dbp_lfv_prt_300s**		text	SpectralBand:0.04:0.15
**hr_time_t_300s**	300 second epoch-- ECG Task	text	Time
**hr_period_t_300s**		text	Period
**hr_epoch_t_300s**		text	Epoch
**hr_duration_t_300s**		text	Duration
**hr_start_t_300s**		text	Start
**hr_end_t_300s**		text	End
**hr_max_time_t_300s**		text	Max_Time
**hr_inttot_t_300s**		text	IntervalsTotal
**hr_intused_t_300s**		text	IntervalsUsed
**hr_avg_t_300s**		text	Average Heart Rate (bpm)
**hr_sd_t_300s**		text	SD
**hr_avd_t_300s**		text	Avd
**hr_rms_t_300s**		text	Sqr(mssd)

**hr_60_medrr_t_300s**		text	60/medrr
**hr_iqr_t_300s**		text	iqrange
**hr_lfv_t_300s**		text	Low Frequency Heart Rate Variability
**hr_hfv_t_300s**		text	High Frequency Heart Rate Variability
**sbp_intav_t_300s**	300 second epoch--SBP Task	text	IntervalAv
**sbp_avd_t_300s**		text	Avd
**sbp_lfv_t_300s**		text	SpectralBand:0.04:0.15
**dbp_intav_t_300s**	300 second epoch--DBP Task	text	IntervalAv
**dbp_avd_t_300s**		text	Avd
**dbp_lfv_t_300s**		text	SpectralBand:0.04:0.15
**hr_time_p5_300s**	300 second epoch--ECG Post5	text	Time
**hr_period_p5_300s**		text	Period
**hr_epoch_p5_300s**		text	Epoch
**hr_duration_p5_300s**		text	Duration
**hr_start_p5_300s**		text	Start

**hr_end_p5_300s**		text	End
**hr_max_time_p5_300s**		text	Max_Time
**hr_inttot_p5_300s**		text	IntervalsTotal
**hr_intused_p5_300s**		text	IntervalsUsed
**hr_avg_p5_300s**		text	Average Heart Rate (bpm)
**hr_sd_p5_300s**		text	SD
**hr_avd_p5_300s**		text	Avd
**hr_rms_p5_300s**		text	Sqr(mssd)
**hr_60_medrr_p5_300s**		text	60/medrr
**hr_iqr_p5_300s**		text	iqrange
**hr_lfv_p5_300s**		text	Low Frequency Heart Rate Variability
**hr_hfv_p5_300s**		text	High Frequency Heart Rate Variability
**sbp_intav_p5_300s**	300 second epoch-- SBP Post5	text	IntervalAv
**sbp_avd_p5_300s**		text	Avd
**sbp_lfv_p5_300s**		text	SpectralBand:0.04:0.15

**dbp_intav_p5_300s**	300 second epoch--DBP Post5	text	IntervalAv
**dbp_avd_p5_300s**		text	Avd
**dbp_lfv_p5_300s**		text	SpectralBand:0.04:0.15
**hr_time_p10_300s**	300 second epoch--ECG Post10	text	Time
**hr_period_p10_300s**		text	Period
**hr_epoch_p10_300s**		text	Epoch
**hr_duration_p10_300s**		text	Duration
**hr_start_p10_300s**		text	Start
**hr_end_p10_300s**		text	End
**hr_max_time_p10_300s**		text	Max_Time
**hr_inttot_p10_300s**		text	IntervalsTotal
**hr_intused_p10_300s**		text	IntervalsUsed
**hr_avg_p10_300s**		text	Average Heart Rate (bpm)
**hr_sd_p10_300s**		text	SD
**hr_avd_p10_300s**		text	Avd

**hr_rms_p10_300s**		text	Sqr(mssd)
**hr_60_medrr_p10_300s**		text	60/medrr
**hr_iqr_p10_300s**		text	iqrange
**hr_lfv_p10_300s**		text	Low Frequency Heart Rate Variability
**hr_hfv_p10_300s**		text	High Frequency Heart Rate Variability
**sbp_intav_p10_300s**	300 second epoch-- SBP Post10	text	IntervalAv
**sbp_avd_p10_300s**		text	Avd
**sbp_lfv_p10_300s**		text	SpectralBand:0.04:0.15
**dbp_intav_p10_300s**	300 second epoch-- DBP Post10	text	IntervalAv
**dbp_avd_p10_300s**		text	Avd
**dbp_lfv_p10_300s**		text	SpectralBand:0.04:0.15
**hr_time_p20_300s**		text	Time
**hr_period_p20_300s**	300 second epoch-- ECG Post20	text	Period
**hr_epoch_p20_300s**		text	Epoch
**hr_duration_p20_300s**		text	Duration

**hr_start_p20_300s**		text	Start
**hr_end_p20_300s**		text	End
**hr_max_time_p20_300s**		text	Max_Time
**hr_inttot_p20_300s**		text	IntervalsTotal
**hr_intused_p20_300s**		text	IntervalsUsed
**hr_avg_p20_300s**		text	Average Heart Rate (bpm)
**hr_sd_p20_300s**		text	SD
**hr_avd_p20_300s**		text	Avd
**hr_rms_p20_300s**		text	Sqr(mssd)
**hr_60_medrr_p20_300s**		text	60/medrr
**hr_iqr_p20_300s**		text	iqrange
**hr_lfv_p20_300s**		text	Low Frequency Heart Rate Variability
**hr_hfv_p20_300s**		text	High Frequency Heart Rate Variability
**sbp_intav_p20_300s**		text	IntervalAv
**sbp_avd_p20_300s**	300 second epoch-- SBP Post20	text	Avd
**sbp_lfv_p20_300s**		text	SpectralBand:0.04:0.15

**dbp_intav_p20_300s**		text	IntervalAv
**dbp_avd_p20_300s**	300 second epoch--DBP Post20	text	Avd
**dbp_lfv_p20_300s**		text	SpectralBand:0.04:0.15
**hr_time_p30_300s**	300 second epoch--ECG Post30	text	Time
**hr_period_p30_300s**		text	Period
**hr_epoch_p30_300s**		text	Epoch
**hr_duration_p30_300s**		text	Duration
**hr_start_p30_300s**		text	Start
**hr_end_p30_300s**		text	End
**hr_max_time_p30_300s**		text	Max_Time
**hr_inttot_p30_300s**		text	IntervalsTotal
**hr_intused_p30_300s**		text	IntervalsUsed
**hr_avg_p30_300s**		text	Average Heart Rate (bpm)
**hr_sd_p30_300s**		text	SD
**hr_avd_p30_300s**		text	Avd

**hr_rms_p30_300s**		text	Sqr(mssd)
**hr_60_medrr_p30_300s**		text	60/medrr
**hr_iqr_p30_300s**		text	iqrange
**hr_lfv_p30_300s**		text	Low Frequency Heart Rate Variability
**hr_hfv_p30_300s**		text	High Frequency Heart Rate Variability
**sbp_intav_p30_300s**	300 second epoch-- SBP Post30	text	IntervalAv
**sbp_avd_p30_300s**		text	Avd
**sbp_lfv_p30_300s**		text	SpectralBand:0.04:0.15
**dbp_intav_p30_300s**	300 second epoch-- DBP Post30	text	IntervalAv
**dbp_avd_p30_300s**		text	Avd
**dbp_lfv_p30_300s**		text	SpectralBand:0.04:0.15
**hr_time_p60_300s**	300 second epoch-- ECG Post60	text	Time
**hr_period_p60_300s**		text	Period
**hr_epoch_p60_300s**		text	Epoch
**hr_duration_p60_300s**		text	Duration

**hr_start_p60_300s**		text	Start
**hr_end_p60_300s**		text	End
**hr_max_time_p60_300s**		text	Max_Time
**hr_inttot_p60_300s**		text	IntervalsTotal
**hr_intused_p60_300s**		text	IntervalsUsed
**hr_avg_p60_300s**		text	Average Heart Rate (bpm)
**hr_sd_p60_300s**		text	SD
**hr_avd_p60_300s**		text	Avd
**hr_rms_p60_300s**		text	Sqr(mssd)
**hr_60_medrr_p60_300s**		text	60/medrr
**hr_iqr_p60_300s**		text	iqrange
**hr_lfv_p60_300s**		text	Low Frequency Heart Rate Variability
**hr_hfv_p60_300s**		text	High Frequency Heart Rate Variability
**sbp_intav_p60_300s**	300 second epoch-- SBP Post60	text	IntervalAv
**sbp_avd_p60_300s**		text	Avd
**sbp_lfv_p60_300s**		text	SpectralBand:0.04:0.15

**dbp_intav_p60_300s**	300 second epoch-- DBP Post60	text	IntervalAv
**dbp_avd_p60_300s**		text	Avd
**dbp_lfv_p60_300s**		text	SpectralBand:0.04:0.15
**hr_time_p90_300s**	300 second epoch-- ECG Post90	text	Time
**hr_period_p90_300s**		text	Period
**hr_epoch_p90_300s**		text	Epoch
**hr_duration_p90_300s**		text	Duration
**hr_start_p90_300s**		text	Start
**hr_end_p90_300s**		text	End
**hr_max_time_p90_300s**		text	Max_Time
**hr_inttot_p90_300s**		text	IntervalsTotal
**hr_intused_p90_300s**		text	IntervalsUsed
**hr_avg_p90_300s**		text	Average Heart Rate (bpm)
**hr_sd_p90_300s**		text	SD
**hr_avd_p90_300s**		text	Avd

**hr_rms_p90_300s**		text	Sqr(mssd)
**hr_60_medrr_p90_300s**		text	60/medrr
**hr_iqr_p90_300s**		text	iqrange
**hr_lfv_p90_300s**		text	Low Frequency Heart Rate Variability
**hr_hfv_p90_300s**		text	High Frequency Heart Rate Variability
**sbp_intav_p90_300s**	300 second epoch--SBP Post90	text	IntervalAv
**sbp_avd_p90_300s**		text	Avd
**sbp_lfv_p90_300s**		text	SpectralBand:0.04:0.15
**dbp_intav_p90_300s**	300 second epoch--DBP Post90	text	IntervalAv
**dbp_avd_p90_300s**		text	Avd
**dbp_lfv_p90_300s**		text	SpectralBand:0.04:0.15
**hr_time_p120_300s**	300 second epoch--ECG Post120	text	Time
**hr_period_p120_300s**		text	Period
**hr_epoch_p120_300s**		text	Epoch

**hr_duration_p120_300s**		text	Duration
**hr_start_p120_300s**		text	Start
**hr_end_p120_300s**		text	End
**hr_max_time_p120_300s**		text	Max_Time
**hr_inttot_p120_300s**		text	IntervalsTotal
**hr_intused_p120_300s**		text	IntervalsUsed
**hr_avg_p120_300s**		text	Average Heart Rate (bpm)
**hr_sd_p120_300s**		text	SD
**hr_avd_p120_300s**		text	Avd
**hr_rms_p120_300s**		text	Sqr(mssd)
**hr_60_medrr_p120_300s**		text	60/medrr
**hr_iqr_p120_300s**		text	iqrange
**hr_lfv_p120_300s**		text	Low Frequency Heart Rate Variability
**hr_hfv_p120_300s**		text	High Frequency Heart Rate Variability
**sbp_intav_p120_300s**	300 second epoch-- SBP Post120	text	IntervalAv
**sbp_avd_p120_300s**		text	Avd

**sbp_lfv_p120_300s**		text	SpectralBand:0.04:0.15
**dbp_intav_p120_300s**	300 second epoch-- DBP Post120	text	IntervalAv
**dbp_avd_p120_300s**		text	Avd
**dbp_lfv_p120_300s**		text	SpectralBand:0.04:0.15
**hr_time_dbt_300s**	300 second epoch-- ECG DBT	text	Time
**hr_period_dbt_300s**		text	Period
**hr_epoch_dbt_300s**		text	Epoch
**hr_duration_dbt_300s**		text	Duration
**hr_start_dbt_300s**		text	Start
**hr_end_dbt_300s**		text	End
**hr_max_time_dbt_300s**		text	Max_Time
**hr_inttot_dbt_300s**		text	IntervalsTotal
**hr_intused_dbt_300s**		text	IntervalsUsed
**hr_avg_dbt_300s**		text	Average Heart Rate (bpm)
**hr_sd_dbt_300s**		text	SD

**hr_avd_dbt_300s**		text	Avd
**hr_rms_dbt_300s**		text	Sqr(mssd)
**hr_60_medrr_dbt_300s**		text	60/medrr
**hr_iqr_dbt_300s**		text	iqrange
**hr_lfv_dbt_300s**		text	Low Frequency Heart Rate Variability
**hr_hfv_dbt_300s**		text	High Frequency Heart Rate Variability
**sbp_intav_dbt_300s**	300 second epoch--SBP DBT	text	IntervalAv
**sbp_avd_dbt_300s**		text	Avd
**sbp_lfv_dbt_300s**		text	SpectralBand:0.04:0.15
**dbp_intav_dbt_300s**	300 second epoch--DBP DBT	text	IntervalAv
**dbp_avd_dbt_300s**		text	Avd
**dbp_lfv_dbt_300s**		text	SpectralBand:0.04:0.15
**hr_time_st_300s**	300 second epoch--ECG ST	text	Time
**hr_period_st_300s**		text	Period
**hr_epoch_st_300s**		text	Epoch

**hr_duration_st_300s**		text	Duration
**hr_start_st_300s**		text	Start
**hr_end_st_300s**		text	End
**hr_max_time_st_300s**		text	Max_Time
**hr_inttot_st_300s**		text	IntervalsTotal
**hr_intused_st_300s**		text	IntervalsUsed
**hr_avg_st_300s**		text	Average Heart Rate (bpm)
**hr_sd_st_300s**		text	SD
**hr_avd_st_300s**		text	Avd
**hr_rms_st_300s**		text	Sqr(mssd)
**hr_60_medrr_st_300s**		text	60/medrr
**hr_iqr_st_300s**		text	iqrange
**hr_lfv_st_300s**		text	Low Frequency Heart Rate Variability
**hr_hfv_st_300s**		text	High Frequency Heart Rate Variability
**sbp_intav_st_300s**	300 second epoch-- SBP ST	text	IntervalAv
**sbp_avd_st_300s**		text	Avd

**sbp_lfv_st_300s**		text	SpectralBand:0.04:0.15
**dbp_intav_st_300s**	300 second epoch-- DBP ST	text	IntervalAv
**dbp_avd_st_300s**		text	Avd
**dbp_lfv_st_300s**		text	SpectralBand:0.04:0.15
**hr_time_sst_300s**	300 second epoch-- ECG SST	text	Time
**hr_period_sst_300s**		text	Period
**hr_epoch_sst_300s**		text	Epoch
**hr_duration_sst_300s**		text	Duration
**hr_start_sst_300s**		text	Start
**hr_end_sst_300s**		text	End
**hr_max_time_sst_300s**		text	Max_Time
**hr_inttot_sst_300s**		text	IntervalsTotal
**hr_intused_sst_300s**		text	IntervalsUsed
**hr_avg_sst_300s**		text	Average Heart Rate (bpm)
**hr_sd_sst_300s**		text	SD

**hr_avd_sst_300s**		text	Avd
**hr_rms_sst_300s**		text	Sqr(mssd)
**hr_60_medrr_sst_300s**		text	60/medrr
**hr_iqr_sst_300s**		text	iqrange
**hr_lfv_sst_300s**		text	Low Frequency Heart Rate Variability
**hr_hfv_sst_300s**		text	High Frequency Heart Rate Variability
**sbp_intav_sst_300s**	300 second epoch-- SBP SST	text	IntervalAv
**sbp_avd_sst_300s**		text	Avd
**sbp_lfv_sst_300s**		text	SpectralBand:0.04:0.15
**dbp_intav_sst_300s**	300 second epoch-- DBP SST	text	IntervalAv
**dbp_avd_sst_300s**		text	Avd
**dbp_lfv_sst_300s**		text	SpectralBand:0.04:0.15
**hr_time_cp_300s**	300 second epoch-- ECG CP	text	Time
**hr_period_cp_300s**		text	Period
**hr_epoch_cp_300s**		text	Epoch

**hr_duration_cp_300s**		text	Duration
**hr_start_cp_300s**		text	Start
**hr_end_cp_300s**		text	End
**hr_max_time_cp_300s**		text	Max_Time
**hr_inttot_cp_300s**		text	IntervalsTotal
**hr_intused_cp_300s**		text	IntervalsUsed
**hr_avg_cp_300s**		text	Average Heart Rate (bpm)
**hr_sd_cp_300s**		text	SD
**hr_avd_cp_300s**		text	Avd
**hr_rms_cp_300s**		text	Sqr(mssd)
**hr_60_medrr_cp_300s**		text	60/medrr
**hr_iqr_cp_300s**		text	iqrange
**hr_lfv_cp_300s**		text	Low Frequency Heart Rate Variability
**hr_hfv_cp_300s**		text	High Frequency Heart Rate Variability
**sbp_intav_cp_300s**	300 second epoch-- SBP CP	text	IntervalAv
**sbp_avd_cp_300s**		text	Avd

**sbp_lfv_cp_300s**		text	SpectralBand:0.04:0.15
**dbp_intav_cp_300s**	300 second epoch-- DBP CP	text	IntervalAv
**dbp_avd_cp_300s**		text	Avd
**dbp_lfv_cp_300s**		text	SpectralBand:0.04:0.15
**hr_avg_b_60s_1**	60 second epoch-- Base	text	Average Heart Rate (bpm)
**hr_avg_b_60s_2**		text	Average Heart Rate (bpm)
**hr_avg_b_60s_3**		text	Average Heart Rate (bpm)
**hr_avg_b_60s_4**		text	Average Heart Rate (bpm)
**hr_avg_b_60s_5**		text	Average Heart Rate (bpm)
**hr_hfv_b_60s_1**		text	HFHRV
**hr_hfv_b_60s_2**		text	HFHRV
**hr_hfv_b_60s_3**		text	HFHRV
**hr_hfv_b_60s_4**		text	HFHRV
**hr_hfv_b_60s_5**		text	HFHRV
**sbp_avd_b_60s_1**		text	Systolic blood pressure value in mmHg

**sbp_avd_b_60s_2**		text	Systolic blood pressure value in mmHg
**sbp_avd_b_60s_3**		text	Systolic blood pressure value in mmHg
**sbp_avd_b_60s_4**		text	Systolic blood pressure value in mmHg
**sbp_avd_b_60s_5**		text	Systolic blood pressure value in mmHg
**dbp_avd_b_60s_1**		text	Diastolic blood pressure valuein mmHg
**dbp_avd_b_60s_2**		text	Diastolic blood pressure valuein mmHg
**dbp_avd_b_60s_3**		text	Diastolic blood pressure valuein mmHg
**dbp_avd_b_60s_4**		text	Diastolic blood pressure valuein mmHg
**dbp_avd_b_60s_5**		text	Diastolic blood pressure valuein mmHg
**respr_count_b_60s_1**		text	resp rate
**respr_count_b_60s_2**		text	resp rate
**respr_count_b_60s_3**		text	resp rate
**respr_count_b_60s_4**		text	resp rate
**respr_count_b_60s_5**		text	resp rate
**hr_avg_instruct_60s_1**	60 second epoch--instruct	text	Average Heart Rate (bpm)
**hr_avg_instruct_60s_2**		text	Average Heart Rate (bpm)

**hr_avg_instruct_60s_3**		text	Average Heart Rate (bpm)
**hr_avg_instruct_60s_4**		text	Average Heart Rate (bpm)
**hr_avg_instruct_60s_5**		text	Average Heart Rate (bpm)
**hr_hfv_instruct_60s_1**		text	HFHRV
**hr_hfv_instruct_60s_2**		text	HFHRV
**hr_hfv_instruct_60s_3**		text	HFHRV
**hr_hfv_instruct_60s_4**		text	HFHRV
**hr_hfv_instruct_60s_5**		text	HFHRV
**sbp_avd_instruct_60s_1**		text	Systolic blood pressure value in mmHg
**sbp_avd_instruct_60s_2**		text	Systolic blood pressure value in mmHg
**sbp_avd_instruct_60s_3**		text	Systolic blood pressure value in mmHg
**sbp_avd_instruct_60s_4**		text	Systolic blood pressure value in mmHg
**sbp_avd_instruct_60s_5**		text	Systolic blood pressure value in mmHg
**dbp_avd_instruct_60s_1**		text	Diastolic blood pressure valuein mmHg
**dbp_avd_instruct_60s_2**		text	Diastolic blood pressure valuein mmHg
**dbp_avd_instruct_60s_3**		text	Diastolic blood pressure valuein mmHg
**dbp_avd_instruct_60s_4**		text	Diastolic blood pressure valuein mmHg

**dbp_avd_instruct_60s_5**		text	Diastolic blood pressure valuein mmHg
**respr_count_instruct_60s_1**	text	resp rate	
**respr_count_instruct_60s_2**	text	resp rate	
**respr_count_instruct_60s_3**	text	resp rate	
**respr_count_instruct_60s_4**	text	resp rate	
**respr_count_instruct_60s_5**	text	resp rate	
**hr_avg_prt_60s_1**	60 second epoch--Pretask	text	Average Heart Rate (bpm)
**hr_avg_prt_60s_2**		text	Average Heart Rate (bpm)
**hr_avg_prt_60s_3**		text	Average Heart Rate (bpm)
**hr_avg_prt_60s_4**		text	Average Heart Rate (bpm)
**hr_avg_prt_60s_5**		text	Average Heart Rate (bpm)
**hr_hfv_prt_60s_1**		text	HFHRV
**hr_hfv_prt_60s_2**		text	HFHRV
**hr_hfv_prt_60s_3**		text	HFHRV
**hr_hfv_prt_60s_4**		text	HFHRV
**hr_hfv_prt_60s_5**		text	HFHRV

**sbp_avd_prt_60s_1**		text	Systolic blood pressure value in mmHg
**sbp_avd_prt_60s_2**		text	Systolic blood pressure value in mmHg
**sbp_avd_prt_60s_3**		text	Systolic blood pressure value in mmHg
**sbp_avd_prt_60s_4**		text	Systolic blood pressure value in mmHg
**sbp_avd_prt_60s_5**		text	Systolic blood pressure value in mmHg
**dbp_avd_prt_60s_1**		text	Diastolic blood pressure valuein mmHg
**dbp_avd_prt_60s_2**		text	Diastolic blood pressure valuein mmHg
**dbp_avd_prt_60s_3**		text	Diastolic blood pressure valuein mmHg
**dbp_avd_prt_60s_4**		text	Diastolic blood pressure valuein mmHg
**dbp_avd_prt_60s_5**		text	Diastolic blood pressure valuein mmHg
**respr_count_prt_60s_1**		text	resp rate
**respr_count_prt_60s_2**		text	resp rate
**respr_count_prt_60s_3**		text	resp rate
**respr_count_prt_60s_4**		text	resp rate
**respr_count_prt_60s_5**		text	resp rate
**hr_avg_pre_60s_1**	60 second epoch--Pre	text	Average Heart Rate (bpm)
**hr_avg_pre_60s_2**		text	Average Heart Rate (bpm)

**hr_avg_pre_60s_3**		text	Average Heart Rate (bpm)
**hr_avg_pre_60s_4**		text	Average Heart Rate (bpm)
**hr_avg_pre_60s_5**		text	Average Heart Rate (bpm)
**hr_hfv_pre_60s_1**		text	HFHRV
**hr_hfv_pre_60s_2**		text	HFHRV
**hr_hfv_pre_60s_3**		text	HFHRV
**hr_hfv_pre_60s_4**		text	HFHRV
**hr_hfv_pre_60s_5**		text	HFHRV
**sbp_avd_pre_60s_1**		text	Systolic blood pressure value in mmHg
**sbp_avd_pre_60s_2**		text	Systolic blood pressure value in mmHg
**sbp_avd_pre_60s_3**		text	Systolic blood pressure value in mmHg
**sbp_avd_pre_60s_4**		text	Systolic blood pressure value in mmHg
**sbp_avd_pre_60s_5**		text	Systolic blood pressure value in mmHg
**dbp_avd_pre_60s_1**		text	Diastolic blood pressure valuein mmHg
**dbp_avd_pre_60s_2**		text	Diastolic blood pressure valuein mmHg
**dbp_avd_pre_60s_3**		text	Diastolic blood pressure valuein mmHg
**dbp_avd_pre_60s_4**		text	Diastolic blood pressure valuein mmHg

**dbp_avd_pre_60s_5**		text	Diastolic blood pressure valuein mmHg
**respr_count_pr_60s_1**		text	resp rate
**respr_count_pr_60s_2**		text	resp rate
**respr_count_pr_60s_3**		text	resp rate
**respr_count_pr_60s_4**		text	resp rate
**respr_count_pr_60s_5**		text	resp rate
**hr_avg_mr_60s_1**	60 second epoch--MR	text	Average Heart Rate (bpm)
**hr_avg_mr_60s_2**		text	Average Heart Rate (bpm)
**hr_avg_mr_60s_3**		text	Average Heart Rate (bpm)
**hr_avg_mr_60s_4**		text	Average Heart Rate (bpm)
**hr_avg_mr_60s_5**		text	Average Heart Rate (bpm)
**hr_hfv_mr_60s_1**		text	HFHRV
**hr_hfv_mr_60s_2**		text	HFHRV
**hr_hfv_mr_60s_3**		text	HFHRV
**hr_hfv_mr_60s_4**		text	HFHRV
**hr_hfv_mr_60s_5**		text	HFHRV
**sbp_avd_mr_60s_1**		text	Systolic blood pressure value in mmHg

**sbp_avd_mr_60s_2**		text	Systolic blood pressure value in mmHg
**sbp_avd_mr_60s_3**		text	Systolic blood pressure value in mmHg
**sbp_avd_mr_60s_4**		text	Systolic blood pressure value in mmHg
**sbp_avd_mr_60s_5**		text	Systolic blood pressure value in mmHg
**dbp_avd_mr_60s_1**		text	Diastolic blood pressure valuein mmHg
**dbp_avd_mr_60s_2**		text	Diastolic blood pressure valuein mmHg
**dbp_avd_mr_60s_3**		text	Diastolic blood pressure valuein mmHg
**dbp_avd_mr_60s_4**		text	Diastolic blood pressure valuein mmHg
**dbp_avd_mr_60s_5**		text	Diastolic blood pressure valuein mmHg
**respr_count_mr_60s_1**		text	resp rate
**respr_count_mr_60s_2**		text	resp rate
**respr_count_mr_60s_3**		text	resp rate
**respr_count_mr_60s_4**		text	resp rate
**respr_count_mr_60s_5**		text	resp rate
**hr_avg_t_60s_1**	60 second epoch--Task	text	Average Heart Rate (bpm)
**hr_avg_t_60s_2**		text	Average Heart Rate (bpm)
**hr_avg_t_60s_3**		text	Average Heart Rate (bpm)

**hr_avg_t_60s_4**		text	Average Heart Rate (bpm)
**hr_avg_t_60s_5**		text	Average Heart Rate (bpm)
**hr_hfv_t_60s_1**		text	HFHRV
**hr_hfv_t_60s_2**		text	HFHRV
**hr_hfv_t_60s_3**		text	HFHRV
**hr_hfv_t_60s_4**		text	HFHRV
**hr_hfv_t_60s_5**		text	HFHRV
**sbp_avd_t_60s_1**		text	Systolic blood pressure value in mmHg
**sbp_avd_t_60s_2**		text	Systolic blood pressure value in mmHg
**sbp_avd_t_60s_3**		text	Systolic blood pressure value in mmHg
**sbp_avd_t_60s_4**		text	Systolic blood pressure value in mmHg
**sbp_avd_t_60s_5**		text	Systolic blood pressure value in mmHg
**dbp_avd_t_60s_1**		text	Diastolic blood pressure valuein mmHg
**dbp_avd_t_60s_2**		text	Diastolic blood pressure valuein mmHg
**dbp_avd_t_60s_3**		text	Diastolic blood pressure valuein mmHg
**dbp_avd_t_60s_4**		text	Diastolic blood pressure valuein mmHg
**dbp_avd_t_60s_5**		text	Diastolic blood pressure valuein mmHg

**respr_count_t_60s_1**		text	resp rate
**respr_count_t_60s_2**		text	resp rate
**respr_count_t_60s_3**		text	resp rate
**respr_count_t_60s_4**		text	resp rate
**respr_count_t_60s_5**		text	resp rate
**hr_avg_p5_60s_1**	60 second epoch--Post5	text	Average Heart Rate (bpm)
**hr_avg_p5_60s_2**		text	Average Heart Rate (bpm)
**hr_avg_p5_60s_3**		text	Average Heart Rate (bpm)
**hr_avg_p5_60s_4**		text	Average Heart Rate (bpm)
**hr_avg_p5_60s_5**		text	Average Heart Rate (bpm)
**hr_hfv_p5_60s_1**		text	HFHRV
**hr_hfv_p5_60s_2**		text	HFHRV
**hr_hfv_p5_60s_3**		text	HFHRV
**hr_hfv_p5_60s_4**		text	HFHRV
**hr_hfv_p5_60s_5**		text	HFHRV
**sbp_avd_p5_60s_1**		text	Systolic blood pressure value in mmHg

**sbp_avd_p5_60s_2**		text	Systolic blood pressure value in mmHg
**sbp_avd_p5_60s_3**		text	Systolic blood pressure value in mmHg
**sbp_avd_p5_60s_4**		text	Systolic blood pressure value in mmHg
**sbp_avd_p5_60s_5**		text	Systolic blood pressure value in mmHg
**dbp_avd_p5_60s_1**		text	Diastolic blood pressure valuein mmHg
**dbp_avd_p5_60s_2**		text	Diastolic blood pressure valuein mmHg
**dbp_avd_p5_60s_3**		text	Diastolic blood pressure valuein mmHg
**dbp_avd_p5_60s_4**		text	Diastolic blood pressure valuein mmHg
**dbp_avd_p5_60s_5**		text	Diastolic blood pressure valuein mmHg
**respr_count_p5_60s_1**		text	resp rate
**respr_count_p5_60s_2**		text	resp rate
**respr_count_p5_60s_3**		text	resp rate
**respr_count_p5_60s_4**		text	resp rate
**respr_count_p5_60s_5**		text	resp rate
**hr_avg_p10_60s_1**	60 second epoch--Post10	text	Average Heart Rate (bpm)
**hr_avg_p10_60s_2**		text	Average Heart Rate (bpm)

**hr_avg_p10_60s_3**		text	Average Heart Rate (bpm)
**hr_avg_p10_60s_4**		text	Average Heart Rate (bpm)
**hr_avg_p10_60s_5**		text	Average Heart Rate (bpm)
**hr_hfv_p10_60s_1**		text	HFHRV
**hr_hfv_p10_60s_2**		text	HFHRV
**hr_hfv_p10_60s_3**		text	HFHRV
**hr_hfv_p10_60s_4**		text	HFHRV
**hr_hfv_p10_60s_5**		text	HFHRV
**sbp_avd_p10_60s_1**		text	Systolic blood pressure value in mmHg
**sbp_avd_p10_60s_2**		text	Systolic blood pressure value in mmHg
**sbp_avd_p10_60s_3**		text	Systolic blood pressure value in mmHg
**sbp_avd_p10_60s_4**		text	Systolic blood pressure value in mmHg
**sbp_avd_p10_60s_5**		text	Systolic blood pressure value in mmHg
**dbp_avd_p10_60s_1**		text	Diastolic blood pressure valuein mmHg
**dbp_avd_p10_60s_2**		text	Diastolic blood pressure valuein mmHg
**dbp_avd_p10_60s_3**		text	Diastolic blood pressure valuein mmHg
**dbp_avd_p10_60s_4**		text	Diastolic blood pressure valuein mmHg

**dbp_avd_p10_60s_5**		text	Diastolic blood pressure valuein mmHg
**respr_count_p10_60s_1**		text	resp rate
**respr_count_p10_60s_2**		text	resp rate
**respr_count_p10_60s_3**		text	resp rate
**respr_count_p10_60s_4**		text	resp rate
**respr_count_p10_60s_5**		text	resp rate
**hr_avg_p20_60s_1**	60 second epoch--Post20	text	Average Heart Rate (bpm)
**hr_avg_p20_60s_2**		text	Average Heart Rate (bpm)
**hr_avg_p20_60s_3**		text	Average Heart Rate (bpm)
**hr_avg_p20_60s_4**		text	Average Heart Rate (bpm)
**hr_avg_p20_60s_5**		text	Average Heart Rate (bpm)
**hr_hfv_p20_60s_1**		text	HFHRV
**hr_hfv_p20_60s_2**		text	HFHRV
**hr_hfv_p20_60s_3**		text	HFHRV
**hr_hfv_p20_60s_4**		text	HFHRV
**hr_hfv_p20_60s_5**		text	HFHRV

**sbp_avd_p20_60s_1**		text	Systolic blood pressure value in mmHg
**sbp_avd_p20_60s_2**		text	Systolic blood pressure value in mmHg
**sbp_avd_p20_60s_3**		text	Systolic blood pressure value in mmHg
**sbp_avd_p20_60s_4**		text	Systolic blood pressure value in mmHg
**sbp_avd_p20_60s_5**		text	Systolic blood pressure value in mmHg
**dbp_avd_p20_60s_1**		text	Diastolic blood pressure valuein mmHg
**dbp_avd_p20_60s_2**		text	Diastolic blood pressure valuein mmHg
**dbp_avd_p20_60s_3**		text	Diastolic blood pressure valuein mmHg
**dbp_avd_p20_60s_4**		text	Diastolic blood pressure valuein mmHg
**dbp_avd_p20_60s_5**		text	Diastolic blood pressure valuein mmHg
**respr_count_p20_60s_1**		text	resp rate
**respr_count_p20_60s_2**		text	resp rate
**respr_count_p20_60s_3**		text	resp rate
**respr_count_p20_60s_4**		text	resp rate
**respr_count_p20_60s_5**		text	resp rate
**hr_avg_p30_60s_1**	60 second epoch--Post30	text	Average Heart Rate (bpm)

**hr_avg_p30_60s_2**		text	Average Heart Rate (bpm)
**hr_avg_p30_60s_3**		text	Average Heart Rate (bpm)
**hr_avg_p30_60s_4**		text	Average Heart Rate (bpm)
**hr_avg_p30_60s_5**		text	Average Heart Rate (bpm)
**hr_hfv_p30_60s_1**		text	HFHRV
**hr_hfv_p30_60s_2**		text	HFHRV
**hr_hfv_p30_60s_3**		text	HFHRV
**hr_hfv_p30_60s_4**		text	HFHRV
**hr_hfv_p30_60s_5**		text	HFHRV
**sbp_avd_p30_60s_1**		text	Systolic blood pressure value in mmHg
**sbp_avd_p30_60s_2**		text	Systolic blood pressure value in mmHg
**sbp_avd_p30_60s_3**		text	Systolic blood pressure value in mmHg
**sbp_avd_p30_60s_4**		text	Systolic blood pressure value in mmHg
**sbp_avd_p30_60s_5**		text	Systolic blood pressure value in mmHg
**dbp_avd_p30_60s_1**		text	Diastolic blood pressure valuein mmHg
**dbp_avd_p30_60s_2**		text	Diastolic blood pressure valuein mmHg
**dbp_avd_p30_60s_3**		text	Diastolic blood pressure valuein mmHg

**dbp_avd_p30_60s_4**		text	Diastolic blood pressure valuein mmHg
**dbp_avd_p30_60s_5**		text	Diastolic blood pressure valuein mmHg
**respr_count_p30_60s_1**		text	resp rate
**respr_count_p30_60s_2**		text	resp rate
**respr_count_p30_60s_3**		text	resp rate
**respr_count_p30_60s_4**		text	resp rate
**respr_count_p30_60s_5**		text	resp rate
**hr_avg_p60_60s_1**	60 second epoch--Post60	text	Average Heart Rate (bpm)
**hr_avg_p60_60s_2**		text	Average Heart Rate (bpm)
**hr_avg_p60_60s_3**		text	Average Heart Rate (bpm)
**hr_avg_p60_60s_4**		text	Average Heart Rate (bpm)
**hr_avg_p60_60s_5**		text	Average Heart Rate (bpm)
**hr_hfv_p60_60s_1**		text	HFHRV
**hr_hfv_p60_60s_2**		text	HFHRV
**hr_hfv_p60_60s_3**		text	HFHRV
**hr_hfv_p60_60s_4**		text	HFHRV

**hr_hfv_p60_60s_5**		text	HFHRV
**sbp_avd_p60_60s_1**		text	Systolic blood pressure value in mmHg
**sbp_avd_p60_60s_2**		text	Systolic blood pressure value in mmHg
**sbp_avd_p60_60s_3**		text	Systolic blood pressure value in mmHg
**sbp_avd_p60_60s_4**		text	Systolic blood pressure value in mmHg
**sbp_avd_p60_60s_5**		text	Systolic blood pressure value in mmHg
**dbp_avd_p60_60s_1**		text	Diastolic blood pressure valuein mmHg
**dbp_avd_p60_60s_2**		text	Diastolic blood pressure valuein mmHg
**dbp_avd_p60_60s_3**		text	Diastolic blood pressure valuein mmHg
**dbp_avd_p60_60s_4**		text	Diastolic blood pressure valuein mmHg
**dbp_avd_p60_60s_5**		text	Diastolic blood pressure valuein mmHg
**respr_count_p60_60s_1**		text	resp rate
**respr_count_p60_60s_2**		text	resp rate
**respr_count_p60_60s_3**		text	resp rate
**respr_count_p60_60s_4**		text	resp rate
**respr_count_p60_60s_5**		text	resp rate

**hr_avg_p90_60s_1**	60 second epoch--Post90	text	Average Heart Rate (bpm)
**hr_avg_p90_60s_2**		text	Average Heart Rate (bpm)
**hr_avg_p90_60s_3**		text	Average Heart Rate (bpm)
**hr_avg_p90_60s_4**		text	Average Heart Rate (bpm)
**hr_avg_p90_60s_5**		text	Average Heart Rate (bpm)
**hr_hfv_p90_60s_1**		text	HFHRV
**hr_hfv_p90_60s_2**		text	HFHRV
**hr_hfv_p90_60s_3**		text	HFHRV
**hr_hfv_p90_60s_4**		text	HFHRV
**hr_hfv_p90_60s_5**		text	HFHRV
**sbp_avd_p90_60s_1**		text	Systolic blood pressure value in mmHg
**sbp_avd_p90_60s_2**		text	Systolic blood pressure value in mmHg
**sbp_avd_p90_60s_3**		text	Systolic blood pressure value in mmHg
**sbp_avd_p90_60s_4**		text	Systolic blood pressure value in mmHg
**sbp_avd_p90_60s_5**		text	Systolic blood pressure value in mmHg
**dbp_avd_p90_60s_1**		text	Diastolic blood pressure valuein mmHg

**dbp_avd_p90_60s_2**		text	Diastolic blood pressure valuein mmHg
**dbp_avd_p90_60s_3**		text	Diastolic blood pressure valuein mmHg
**dbp_avd_p90_60s_4**		text	Diastolic blood pressure valuein mmHg
**dbp_avd_p90_60s_5**		text	Diastolic blood pressure valuein mmHg
**respr_count_p90_60s_1**		text	resp rate
**respr_count_p90_60s_2**		text	resp rate
**respr_count_p90_60s_3**		text	resp rate
**respr_count_p90_60s_4**		text	resp rate
**respr_count_p90_60s_5**		text	resp rate
**hr_avg_p120_60s_1**	60 second epoch--Post120	text	Average Heart Rate (bpm)
**hr_avg_p120_60s_2**		text	Average Heart Rate (bpm)
**hr_avg_p120_60s_3**		text	Average Heart Rate (bpm)
**hr_avg_p120_60s_4**		text	Average Heart Rate (bpm)
**hr_avg_p120_60s_5**		text	Average Heart Rate (bpm)
**hr_hfv_p120_60s_1**		text	HFHRV
**hr_hfv_p120_60s_2**		text	HFHRV

**hr_hfv_p120_60s_3**		text	HFHRV
**hr_hfv_p120_60s_4**		text	HFHRV
**hr_hfv_p120_60s_5**		text	HFHRV
**sbp_avd_p120_60s_1**		text	Systolic blood pressure value in mmHg
**sbp_avd_p120_60s_2**		text	Systolic blood pressure value in mmHg
**sbp_avd_p120_60s_3**		text	Systolic blood pressure value in mmHg
**sbp_avd_p120_60s_4**		text	Systolic blood pressure value in mmHg
**sbp_avd_p120_60s_5**		text	Systolic blood pressure value in mmHg
**dbp_avd_p120_60s_1**		text	Diastolic blood pressure valuein mmHg
**dbp_avd_p120_60s_2**		text	Diastolic blood pressure valuein mmHg
**dbp_avd_p120_60s_3**		text	Diastolic blood pressure valuein mmHg
**dbp_avd_p120_60s_4**		text	Diastolic blood pressure valuein mmHg
**dbp_avd_p120_60s_5**		text	Diastolic blood pressure valuein mmHg
**respr_count_p120_60s_1**		text	resp rate
**respr_count_p120_60s_2**		text	resp rate
**respr_count_p120_60s_3**		text	resp rate
**respr_count_p120_60s_4**		text	resp rate

**respr_count_p120_60s_5**		text	resp rate
**hr_avg_dbt_60s_1**	60 second epoch--DBT	text	Average Heart Rate (bpm)
**hr_avg_dbt_60s_2**		text	Average Heart Rate (bpm)
**hr_avg_dbt_60s_3**		text	Average Heart Rate (bpm)
**hr_avg_dbt_60s_4**		text	Average Heart Rate (bpm)
**hr_avg_dbt_60s_5**		text	Average Heart Rate (bpm)
**hr_hfv_dbt_60s_1**		text	HFHRV
**hr_hfv_dbt_60s_2**		text	HFHRV
**hr_hfv_dbt_60s_3**		text	HFHRV
**hr_hfv_dbt_60s_4**		text	HFHRV
**hr_hfv_dbt_60s_5**		text	HFHRV
**sbp_avd_dbt_60s_1**		text	Systolic blood pressure value in mmHg
**sbp_avd_dbt_60s_2**		text	Systolic blood pressure value in mmHg
**sbp_avd_dbt_60s_3**		text	Systolic blood pressure value in mmHg
**sbp_avd_dbt_60s_4**		text	Systolic blood pressure value in mmHg
**sbp_avd_dbt_60s_5**		text	Systolic blood pressure value in mmHg
**dbp_avd_dbt_60s_1**		text	Diastolic blood pressure valuein mmHg

**dbp_avd_dbt_60s_2**		text	Diastolic blood pressure valuein mmHg
**dbp_avd_dbt_60s_3**		text	Diastolic blood pressure valuein mmHg
**dbp_avd_dbt_60s_4**		text	Diastolic blood pressure valuein mmHg
**dbp_avd_dbt_60s_5**		text	Diastolic blood pressure valuein mmHg
**respr_count_dbt_60s_1**		text	resp rate
**respr_count_dbt_60s_2**		text	resp rate
**respr_count_dbt_60s_3**		text	resp rate
**respr_count_dbt_60s_4**		text	resp rate
**respr_count_dbt_60s_5**		text	resp rate
**hr_avg_st_60s_1**	60 second epoch--ST	text	Average Heart Rate (bpm)
**hr_avg_st_60s_2**		text	Average Heart Rate (bpm)
**hr_avg_st_60s_3**		text	Average Heart Rate (bpm)
**hr_avg_st_60s_4**		text	Average Heart Rate (bpm)
**hr_avg_st_60s_5**		text	Average Heart Rate (bpm)
**hr_hfv_st_60s_1**		text	HFHRV
**hr_hfv_st_60s_2**		text	HFHRV
**hr_hfv_st_60s_3**		text	HFHRV

**hr_hfv_st_60s_4**		text	HFHRV
**hr_hfv_st_60s_5**		text	HFHRV
**sbp_avd_st_60s_1**		text	Systolic blood pressure value in mmHg
**sbp_avd_st_60s_2**		text	Systolic blood pressure value in mmHg
**sbp_avd_st_60s_3**		text	Systolic blood pressure value in mmHg
**sbp_avd_st_60s_4**		text	Systolic blood pressure value in mmHg
**sbp_avd_st_60s_5**		text	Systolic blood pressure value in mmHg
**dbp_avd_st_60s_1**		text	Diastolic blood pressure valuein mmHg
**dbp_avd_st_60s_2**		text	Diastolic blood pressure valuein mmHg
**dbp_avd_st_60s_3**		text	Diastolic blood pressure valuein mmHg
**dbp_avd_st_60s_4**		text	Diastolic blood pressure valuein mmHg
**dbp_avd_st_60s_5**		text	Diastolic blood pressure valuein mmHg
**respr_count_st_60s_1**		text	resp rate
**respr_count_st_60s_2**		text	resp rate
**respr_count_st_60s_3**		text	resp rate
**respr_count_st_60s_4**		text	resp rate
**respr_count_st_60s_5**		text	resp rate

**hr_avg_sst_60s_1**	60 second epoch--SST	text	Average Heart Rate (bpm)
**hr_avg_sst_60s_2**		text	Average Heart Rate (bpm)
**hr_avg_sst_60s_3**		text	Average Heart Rate (bpm)
**hr_avg_sst_60s_4**		text	Average Heart Rate (bpm)
**hr_avg_sst_60s_5**		text	Average Heart Rate (bpm)
**hr_hfv_sst_60s_1**		text	HFHRV
**hr_hfv_sst_60s_2**		text	HFHRV
**hr_hfv_sst_60s_3**		text	HFHRV
**hr_hfv_sst_60s_4**		text	HFHRV
**hr_hfv_sst_60s_5**		text	HFHRV
**sbp_avd_sst_60s_1**		text	Systolic blood pressure value in mmHg
**sbp_avd_sst_60s_2**		text	Systolic blood pressure value in mmHg
**sbp_avd_sst_60s_3**		text	Systolic blood pressure value in mmHg
**sbp_avd_sst_60s_4**		text	Systolic blood pressure value in mmHg
**sbp_avd_sst_60s_5**		text	Systolic blood pressure value in mmHg
**dbp_avd_sst_60s_1**		text	Diastolic blood pressure valuein mmHg
**dbp_avd_sst_60s_2**		text	Diastolic blood pressure valuein mmHg

**dbp_avd_sst_60s_3**		text	Diastolic blood pressure valuein mmHg
**dbp_avd_sst_60s_4**		text	Diastolic blood pressure valuein mmHg
**dbp_avd_sst_60s_5**		text	Diastolic blood pressure valuein mmHg
**respr_count_sst_60s_1**		text	resp rate
**respr_count_sst_60s_2**		text	resp rate
**respr_count_sst_60s_3**		text	resp rate
**respr_count_sst_60s_4**		text	resp rate
**respr_count_sst_60s_5**		text	resp rate
**hr_avg_cp_60s_1**	60 second epoch--CP	text	Average Heart Rate (bpm)
**hr_avg_cp_60s_2**		text	Average Heart Rate (bpm)
**hr_avg_cp_60s_3**		text	Average Heart Rate (bpm)
**hr_avg_cp_60s_4**		text	Average Heart Rate (bpm)
**hr_avg_cp_60s_5**		text	Average Heart Rate (bpm)
**hr_hfv_cp_60s_1**		text	HFHRV
**hr_hfv_cp_60s_2**		text	HFHRV
**hr_hfv_cp_60s_3**		text	HFHRV
**hr_hfv_cp_60s_4**		text	HFHRV

**hr_hfv_cp_60s_5**		text	HFHRV
**sbp_avd_cp_60s_1**		text	Systolic blood pressure value in mmHg
**sbp_avd_cp_60s_2**		text	Systolic blood pressure value in mmHg
**sbp_avd_cp_60s_3**		text	Systolic blood pressure value in mmHg
**sbp_avd_cp_60s_4**		text	Systolic blood pressure value in mmHg
**sbp_avd_cp_60s_5**		text	Systolic blood pressure value in mmHg
**dbp_avd_cp_60s_1**		text	Diastolic blood pressure valuein mmHg
**dbp_avd_cp_60s_2**		text	Diastolic blood pressure valuein mmHg
**dbp_avd_cp_60s_3**		text	Diastolic blood pressure valuein mmHg
**dbp_avd_cp_60s_4**		text	Diastolic blood pressure valuein mmHg
**dbp_avd_cp_60s_5**		text	Diastolic blood pressure valuein mmHg
**respr_count_cp_60s_1**		text	resp rate
**respr_count_cp_60s_2**		text	resp rate
**respr_count_cp_60s_3**		text	resp rate
**respr_count_cp_60s_4**		text	resp rate
**respr_count_cp_60s_5**		text	resp rate

**hr_avg_b_10s_1**	10 second epoch--Base	text	Average Heart Rate (bpm)
**hr_avg_b_10s_2**		text	Average Heart Rate (bpm)
**hr_avg_b_10s_3**		text	Average Heart Rate (bpm)
**hr_avg_b_10s_4**		text	Average Heart Rate (bpm)
**hr_avg_b_10s_5**		text	Average Heart Rate (bpm)
**hr_avg_b_10s_6**		text	Average Heart Rate (bpm)
**hr_avg_b_10s_7**		text	Average Heart Rate (bpm)
**hr_avg_b_10s_8**		text	Average Heart Rate (bpm)
**hr_avg_b_10s_9**		text	Average Heart Rate (bpm)
**hr_avg_b_10s_10**		text	Average Heart Rate (bpm)
**hr_avg_b_10s_11**		text	Average Heart Rate (bpm)
**hr_avg_b_10s_12**		text	Average Heart Rate (bpm)
**hr_avg_b_10s_13**		text	Average Heart Rate (bpm)
**hr_avg_b_10s_14**		text	Average Heart Rate (bpm)
**hr_avg_b_10s_15**		text	Average Heart Rate (bpm)
**hr_avg_b_10s_16**		text	Average Heart Rate (bpm)

**hr_avg_b_10s_17**		text	Average Heart Rate (bpm)
**hr_avg_b_10s_18**		text	Average Heart Rate (bpm)
**hr_avg_b_10s_19**		text	Average Heart Rate (bpm)
**hr_avg_b_10s_20**		text	Average Heart Rate (bpm)
**hr_avg_b_10s_21**		text	Average Heart Rate (bpm)
**hr_avg_b_10s_22**		text	Average Heart Rate (bpm)
**hr_avg_b_10s_23**		text	Average Heart Rate (bpm)
**hr_avg_b_10s_24**		text	Average Heart Rate (bpm)
**hr_avg_b_10s_25**		text	Average Heart Rate (bpm)
**hr_avg_b_10s_26**		text	Average Heart Rate (bpm)
**hr_avg_b_10s_27**		text	Average Heart Rate (bpm)
**hr_avg_b_10s_28**		text	Average Heart Rate (bpm)
**hr_avg_b_10s_29**		text	Average Heart Rate (bpm)
**hr_avg_b_10s_30**		text	Average Heart Rate (bpm)
**sbp_avd_b_10s_1**		text	Systolic blood pressure value in mmHg
**sbp_avd_b_10s_2**		text	Systolic blood pressure value in mmHg
**sbp_avd_b_10s_3**		text	Systolic blood pressure value in mmHg

**sbp_avd_b_10s_4**		text	Systolic blood pressure value in mmHg
**sbp_avd_b_10s_5**		text	Systolic blood pressure value in mmHg
**sbp_avd_b_10s_6**		text	Systolic blood pressure value in mmHg
**sbp_avd_b_10s_7**		text	Systolic blood pressure value in mmHg
**sbp_avd_b_10s_8**		text	Systolic blood pressure value in mmHg
**sbp_avd_b_10s_9**		text	Systolic blood pressure value in mmHg
**sbp_avd_b_10s_10**		text	Systolic blood pressure value in mmHg
**sbp_avd_b_10s_11**		text	Systolic blood pressure value in mmHg
**sbp_avd_b_10s_12**		text	Systolic blood pressure value in mmHg
**sbp_avd_b_10s_13**		text	Systolic blood pressure value in mmHg
**sbp_avd_b_10s_14**		text	Systolic blood pressure value in mmHg
**sbp_avd_b_10s_15**		text	Systolic blood pressure value in mmHg
**sbp_avd_b_10s_16**		text	Systolic blood pressure value in mmHg
**sbp_avd_b_10s_17**		text	Systolic blood pressure value in mmHg
**sbp_avd_b_10s_18**		text	Systolic blood pressure value in mmHg
**sbp_avd_b_10s_19**		text	Systolic blood pressure value in mmHg
**sbp_avd_b_10s_20**		text	Systolic blood pressure value in mmHg

**sbp_avd_b_10s_21**		text	Systolic blood pressure value in mmHg
**sbp_avd_b_10s_22**		text	Systolic blood pressure value in mmHg
**sbp_avd_b_10s_23**		text	Systolic blood pressure value in mmHg
**sbp_avd_b_10s_24**		text	Systolic blood pressure value in mmHg
**sbp_avd_b_10s_25**		text	Systolic blood pressure value in mmHg
**sbp_avd_b_10s_26**		text	Systolic blood pressure value in mmHg
**sbp_avd_b_10s_27**		text	Systolic blood pressure value in mmHg
**sbp_avd_b_10s_28**		text	Systolic blood pressure value in mmHg
**sbp_avd_b_10s_29**		text	Systolic blood pressure value in mmHg
**sbp_avd_b_10s_30**		text	Systolic blood pressure value in mmHg
**dbp_avd_b_10s_1**		text	Diastolic blood pressure valuein mmHg
**dbp_avd_b_10s_2**		text	Diastolic blood pressure valuein mmHg
**dbp_avd_b_10s_3**		text	Diastolic blood pressure valuein mmHg
**dbp_avd_b_10s_4**		text	Diastolic blood pressure valuein mmHg
**dbp_avd_b_10s_5**		text	Diastolic blood pressure valuein mmHg
**dbp_avd_b_10s_6**		text	Diastolic blood pressure valuein mmHg
**dbp_avd_b_10s_7**		text	Diastolic blood pressure valuein mmHg

**dbp_avd_b_10s_8**		text	Diastolic blood pressure valuein mmHg
**dbp_avd_b_10s_9**		text	Diastolic blood pressure valuein mmHg
**dbp_avd_b_10s_10**		text	Diastolic blood pressure valuein mmHg
**dbp_avd_b_10s_11**		text	Diastolic blood pressure valuein mmHg
**dbp_avd_b_10s_12**		text	Diastolic blood pressure valuein mmHg
**dbp_avd_b_10s_13**		text	Diastolic blood pressure valuein mmHg
**dbp_avd_b_10s_14**		text	Diastolic blood pressure valuein mmHg
**dbp_avd_b_10s_15**		text	Diastolic blood pressure valuein mmHg
**dbp_avd_b_10s_16**		text	Diastolic blood pressure valuein mmHg
**dbp_avd_b_10s_17**		text	Diastolic blood pressure valuein mmHg
**dbp_avd_b_10s_18**		text	Diastolic blood pressure valuein mmHg
**dbp_avd_b_10s_19**		text	Diastolic blood pressure valuein mmHg
**dbp_avd_b_10s_20**		text	Diastolic blood pressure valuein mmHg
**dbp_avd_b_10s_21**		text	Diastolic blood pressure valuein mmHg
**dbp_avd_b_10s_22**		text	Diastolic blood pressure valuein mmHg
**dbp_avd_b_10s_23**		text	Diastolic blood pressure valuein mmHg
**dbp_avd_b_10s_24**		text	Diastolic blood pressure valuein mmHg

**dbp_avd_b_10s_25**		text	Diastolic blood pressure valuein mmHg
**dbp_avd_b_10s_26**		text	Diastolic blood pressure valuein mmHg
**dbp_avd_b_10s_27**		text	Diastolic blood pressure valuein mmHg
**dbp_avd_b_10s_28**		text	Diastolic blood pressure valuein mmHg
**dbp_avd_b_10s_29**		text	Diastolic blood pressure valuein mmHg
**dbp_avd_b_10s_30**		text	Diastolic blood pressure valuein mmHg
**hr_avg_pr_10s_1**	10 second epoch--Pre	text	Average Heart Rate (bpm)
**hr_avg_pr_10s_2**		text	Average Heart Rate (bpm)
**hr_avg_pr_10s_3**		text	Average Heart Rate (bpm)
**hr_avg_pr_10s_4**		text	Average Heart Rate (bpm)
**hr_avg_pr_10s_5**		text	Average Heart Rate (bpm)
**hr_avg_pr_10s_6**		text	Average Heart Rate (bpm)
**hr_avg_pr_10s_7**		text	Average Heart Rate (bpm)
**hr_avg_pr_10s_8**		text	Average Heart Rate (bpm)
**hr_avg_pr_10s_9**		text	Average Heart Rate (bpm)
**hr_avg_pr_10s_10**		text	Average Heart Rate (bpm)
**hr_avg_pr_10s_11**		text	Average Heart Rate (bpm)

**hr_avg_pr_10s_12**		text	Average Heart Rate (bpm)
**hr_avg_pr_10s_13**		text	Average Heart Rate (bpm)
**hr_avg_pr_10s_14**		text	Average Heart Rate (bpm)
**hr_avg_pr_10s_15**		text	Average Heart Rate (bpm)
**hr_avg_pr_10s_16**		text	Average Heart Rate (bpm)
**hr_avg_pr_10s_17**		text	Average Heart Rate (bpm)
**hr_avg_pr_10s_18**		text	Average Heart Rate (bpm)
**hr_avg_pr_10s_19**		text	Average Heart Rate (bpm)
**hr_avg_pr_10s_20**		text	Average Heart Rate (bpm)
**hr_avg_pr_10s_21**		text	Average Heart Rate (bpm)
**hr_avg_pr_10s_22**		text	Average Heart Rate (bpm)
**hr_avg_pr_10s_23**		text	Average Heart Rate (bpm)
**hr_avg_pr_10s_24**		text	Average Heart Rate (bpm)
**hr_avg_pr_10s_25**		text	Average Heart Rate (bpm)
**hr_avg_pr_10s_26**		text	Average Heart Rate (bpm)
**hr_avg_pr_10s_27**		text	Average Heart Rate (bpm)
**hr_avg_pr_10s_28**		text	Average Heart Rate (bpm)

**hr_avg_pr_10s_29**		text	Average Heart Rate (bpm)
**hr_avg_pr_10s_30**		text	Average Heart Rate (bpm)
**sbp_avd_pr_10s_1**		text	Systolic blood pressure value in mmHg
**sbp_avd_pr_10s_2**		text	Systolic blood pressure value in mmHg
**sbp_avd_pr_10s_3**		text	Systolic blood pressure value in mmHg
**sbp_avd_pr_10s_4**		text	Systolic blood pressure value in mmHg
**sbp_avd_pr_10s_5**		text	Systolic blood pressure value in mmHg
**sbp_avd_pr_10s_6**		text	Systolic blood pressure value in mmHg
**sbp_avd_pr_10s_7**		text	Systolic blood pressure value in mmHg
**sbp_avd_pr_10s_8**		text	Systolic blood pressure value in mmHg
**sbp_avd_pr_10s_9**		text	Systolic blood pressure value in mmHg
**sbp_avd_pr_10s_10**		text	Systolic blood pressure value in mmHg
**sbp_avd_pr_10s_11**		text	Systolic blood pressure value in mmHg
**sbp_avd_pr_10s_12**		text	Systolic blood pressure value in mmHg
**sbp_avd_pr_10s_13**		text	Systolic blood pressure value in mmHg
**sbp_avd_pr_10s_14**		text	Systolic blood pressure value in mmHg
**sbp_avd_pr_10s_15**		text	Systolic blood pressure value in mmHg

**sbp_avd_pr_10s_16**		text	Systolic blood pressure value in mmHg
**sbp_avd_pr_10s_17**		text	Systolic blood pressure value in mmHg
**sbp_avd_pr_10s_18**		text	Systolic blood pressure value in mmHg
**sbp_avd_pr_10s_19**		text	Systolic blood pressure value in mmHg
**sbp_avd_pr_10s_20**		text	Systolic blood pressure value in mmHg
**sbp_avd_pr_10s_21**		text	Systolic blood pressure value in mmHg
**sbp_avd_pr_10s_22**		text	Systolic blood pressure value in mmHg
**sbp_avd_pr_10s_23**		text	Systolic blood pressure value in mmHg
**sbp_avd_pr_10s_24**		text	Systolic blood pressure value in mmHg
**sbp_avd_pr_10s_25**		text	Systolic blood pressure value in mmHg
**sbp_avd_pr_10s_26**		text	Systolic blood pressure value in mmHg
**sbp_avd_pr_10s_27**		text	Systolic blood pressure value in mmHg
**sbp_avd_pr_10s_28**		text	Systolic blood pressure value in mmHg
**sbp_avd_pr_10s_29**		text	Systolic blood pressure value in mmHg
**sbp_avd_pr_10s_30**		text	Systolic blood pressure value in mmHg
**dbp_avd_pr_10s_1**		text	Diastolic blood pressure valuein mmHg
**dbp_avd_pr_10s_2**		text	Diastolic blood pressure valuein mmHg

**dbp_avd_pr_10s_3**		text	Diastolic blood pressure valuein mmHg
**dbp_avd_pr_10s_4**		text	Diastolic blood pressure valuein mmHg
**dbp_avd_pr_10s_5**		text	Diastolic blood pressure valuein mmHg
**dbp_avd_pr_10s_6**		text	Diastolic blood pressure valuein mmHg
**dbp_avd_pr_10s_7**		text	Diastolic blood pressure valuein mmHg
**dbp_avd_pr_10s_8**		text	Diastolic blood pressure valuein mmHg
**dbp_avd_pr_10s_9**		text	Diastolic blood pressure valuein mmHg
**dbp_avd_pr_10s_10**		text	Diastolic blood pressure valuein mmHg
**dbp_avd_pr_10s_11**		text	Diastolic blood pressure valuein mmHg
**dbp_avd_pr_10s_12**		text	Diastolic blood pressure valuein mmHg
**dbp_avd_pr_10s_13**		text	Diastolic blood pressure valuein mmHg
**dbp_avd_pr_10s_14**		text	Diastolic blood pressure valuein mmHg
**dbp_avd_pr_10s_15**		text	Diastolic blood pressure valuein mmHg
**dbp_avd_pr_10s_16**		text	Diastolic blood pressure valuein mmHg
**dbp_avd_pr_10s_17**		text	Diastolic blood pressure valuein mmHg
**dbp_avd_pr_10s_18**		text	Diastolic blood pressure valuein mmHg
**dbp_avd_pr_10s_19**		text	Diastolic blood pressure valuein mmHg

**dbp_avd_pr_10s_20**		text	Diastolic blood pressure valuein mmHg
**dbp_avd_pr_10s_21**		text	Diastolic blood pressure valuein mmHg
**dbp_avd_pr_10s_22**		text	Diastolic blood pressure valuein mmHg
**dbp_avd_pr_10s_23**		text	Diastolic blood pressure valuein mmHg
**dbp_avd_pr_10s_24**		text	Diastolic blood pressure valuein mmHg
**dbp_avd_pr_10s_25**		text	Diastolic blood pressure valuein mmHg
**dbp_avd_pr_10s_26**		text	Diastolic blood pressure valuein mmHg
**dbp_avd_pr_10s_27**		text	Diastolic blood pressure valuein mmHg
**dbp_avd_pr_10s_28**		text	Diastolic blood pressure valuein mmHg
**dbp_avd_pr_10s_29**		text	Diastolic blood pressure valuein mmHg
**dbp_avd_pr_10s_30**		text	Diastolic blood pressure valuein mmHg
**hr_avg_instruct_10s_1**	10 second epoch--Instruct	text	Average Heart Rate (bpm)
**hr_avg_instruct_10s_2**		text	Average Heart Rate (bpm)
**hr_avg_instruct_10s_3**		text	Average Heart Rate (bpm)
**hr_avg_instruct_10s_4**		text	Average Heart Rate (bpm)
**hr_avg_instruct_10s_5**		text	Average Heart Rate (bpm)

**hr_avg_instruct_10s_6**		text	Average Heart Rate (bpm)
**hr_avg_instruct_10s_7**		text	Average Heart Rate (bpm)
**hr_avg_instruct_10s_8**		text	Average Heart Rate (bpm)
**hr_avg_instruct_10s_9**		text	Average Heart Rate (bpm)
**hr_avg_instruct_10s_10**		text	Average Heart Rate (bpm)
**hr_avg_instruct_10s_11**		text	Average Heart Rate (bpm)
**hr_avg_instruct_10s_12**		text	Average Heart Rate (bpm)
**hr_avg_instruct_10s_13**		text	Average Heart Rate (bpm)
**hr_avg_instruct_10s_14**		text	Average Heart Rate (bpm)
**hr_avg_instruct_10s_15**		text	Average Heart Rate (bpm)
**hr_avg_instruct_10s_16**		text	Average Heart Rate (bpm)
**hr_avg_instruct_10s_17**		text	Average Heart Rate (bpm)
**hr_avg_instruct_10s_18**		text	Average Heart Rate (bpm)
**hr_avg_instruct_10s_19**		text	Average Heart Rate (bpm)
**hr_avg_instruct_10s_20**		text	Average Heart Rate (bpm)
**hr_avg_instruct_10s_21**		text	Average Heart Rate (bpm)
**hr_avg_instruct_10s_22**		text	Average Heart Rate (bpm)

**hr_avg_instruct_10s_23**		text	Average Heart Rate (bpm)
**hr_avg_instruct_10s_24**		text	Average Heart Rate (bpm)
**hr_avg_instruct_10s_25**		text	Average Heart Rate (bpm)
**hr_avg_instruct_10s_26**		text	Average Heart Rate (bpm)
**hr_avg_instruct_10s_27**		text	Average Heart Rate (bpm)
**hr_avg_instruct_10s_28**		text	Average Heart Rate (bpm)
**hr_avg_instruct_10s_29**		text	Average Heart Rate (bpm)
**hr_avg_instruct_10s_30**		text	Average Heart Rate (bpm)
**sbp_avd_instruct_10s_1**		text	Systolic blood pressure value in mmHg
**sbp_avd_instruct_10s_2**		text	Systolic blood pressure value in mmHg
**sbp_avd_instruct_10s_3**		text	Systolic blood pressure value in mmHg
**sbp_avd_instruct_10s_4**		text	Systolic blood pressure value in mmHg
**sbp_avd_instruct_10s_5**		text	Systolic blood pressure value in mmHg
**sbp_avd_instruct_10s_6**		text	Systolic blood pressure value in mmHg
**sbp_avd_instruct_10s_7**		text	Systolic blood pressure value in mmHg
**sbp_avd_instruct_10s_8**		text	Systolic blood pressure value in mmHg
**sbp_avd_instruct_10s_9**		text	Systolic blood pressure value in mmHg

**sbp_avd_instruct_10s_10**		text	Systolic blood pressure value in mmHg
**sbp_avd_instruct_10s_11**		text	Systolic blood pressure value in mmHg
**sbp_avd_instruct_10s_12**		text	Systolic blood pressure value in mmHg
**sbp_avd_instruct_10s_13**		text	Systolic blood pressure value in mmHg
**sbp_avd_instruct_10s_14**		text	Systolic blood pressure value in mmHg
**sbp_avd_instruct_10s_15**		text	Systolic blood pressure value in mmHg
**sbp_avd_instruct_10s_16**		text	Systolic blood pressure value in mmHg
**sbp_avd_instruct_10s_17**		text	Systolic blood pressure value in mmHg
**sbp_avd_instruct_10s_18**		text	Systolic blood pressure value in mmHg
**sbp_avd_instruct_10s_19**		text	Systolic blood pressure value in mmHg
**sbp_avd_instruct_10s_20**		text	Systolic blood pressure value in mmHg
**sbp_avd_instruct_10s_21**		text	Systolic blood pressure value in mmHg
**sbp_avd_instruct_10s_22**		text	Systolic blood pressure value in mmHg
**sbp_avd_instruct_10s_23**		text	Systolic blood pressure value in mmHg
**sbp_avd_instruct_10s_24**		text	Systolic blood pressure value in mmHg
**sbp_avd_instruct_10s_25**		text	Systolic blood pressure value in mmHg
**sbp_avd_instruct_10s_26**		text	Systolic blood pressure value in mmHg

**sbp_avd_instruct_10s_27**		text	Systolic blood pressure value in mmHg
**sbp_avd_instruct_10s_28**		text	Systolic blood pressure value in mmHg
**sbp_avd_instruct_10s_29**		text	Systolic blood pressure value in mmHg
**sbp_avd_instruct_10s_30**		text	Systolic blood pressure value in mmHg
**dbp_avd_instruct_10s_1**		text	Diastolic blood pressure valuein mmHg
**dbp_avd_instruct_10s_2**		text	Diastolic blood pressure valuein mmHg
**dbp_avd_instruct_10s_3**		text	Diastolic blood pressure valuein mmHg
**dbp_avd_instruct_10s_4**		text	Diastolic blood pressure valuein mmHg
**dbp_avd_instruct_10s_5**		text	Diastolic blood pressure valuein mmHg
**dbp_avd_instruct_10s_6**		text	Diastolic blood pressure valuein mmHg
**dbp_avd_instruct_10s_7**		text	Diastolic blood pressure valuein mmHg
**dbp_avd_instruct_10s_8**		text	Diastolic blood pressure valuein mmHg
**dbp_avd_instruct_10s_9**		text	Diastolic blood pressure valuein mmHg
**dbp_avd_instruct_10s_10**		text	Diastolic blood pressure valuein mmHg
**dbp_avd_instruct_10s_11**		text	Diastolic blood pressure valuein mmHg
**dbp_avd_instruct_10s_12**		text	Diastolic blood pressure valuein mmHg
**dbp_avd_instruct_10s_13**		text	Diastolic blood pressure valuein mmHg

**dbp_avd_instruct_10s_14**		text	Diastolic blood pressure valuein mmHg
**dbp_avd_instruct_10s_15**		text	Diastolic blood pressure valuein mmHg
**dbp_avd_instruct_10s_16**		text	Diastolic blood pressure valuein mmHg
**dbp_avd_instruct_10s_17**		text	Diastolic blood pressure valuein mmHg
**dbp_avd_instruct_10s_18**		text	Diastolic blood pressure valuein mmHg
**dbp_avd_instruct_10s_19**		text	Diastolic blood pressure valuein mmHg
**dbp_avd_instruct_10s_20**		text	Diastolic blood pressure valuein mmHg
**dbp_avd_instruct_10s_21**		text	Diastolic blood pressure valuein mmHg
**dbp_avd_instruct_10s_22**		text	Diastolic blood pressure valuein mmHg
**dbp_avd_instruct_10s_23**		text	Diastolic blood pressure valuein mmHg
**dbp_avd_instruct_10s_24**		text	Diastolic blood pressure valuein mmHg
**dbp_avd_instruct_10s_25**		text	Diastolic blood pressure valuein mmHg
**dbp_avd_instruct_10s_26**		text	Diastolic blood pressure valuein mmHg
**dbp_avd_instruct_10s_27**		text	Diastolic blood pressure valuein mmHg
**dbp_avd_instruct_10s_28**		text	Diastolic blood pressure valuein mmHg
**dbp_avd_instruct_10s_29**		text	Diastolic blood pressure valuein mmHg
**dbp_avd_instruct_10s_30**		text	Diastolic blood pressure valuein mmHg

**hr_avg_prt_10s_1**	10 second epoch--Pretask	text	Average Heart Rate (bpm)
**hr_avg_prt_10s_2**		text	Average Heart Rate (bpm)
**hr_avg_prt_10s_3**		text	Average Heart Rate (bpm)
**hr_avg_prt_10s_4**		text	Average Heart Rate (bpm)
**hr_avg_prt_10s_5**		text	Average Heart Rate (bpm)
**hr_avg_prt_10s_6**		text	Average Heart Rate (bpm)
**hr_avg_prt_10s_7**		text	Average Heart Rate (bpm)
**hr_avg_prt_10s_8**		text	Average Heart Rate (bpm)
**hr_avg_prt_10s_9**		text	Average Heart Rate (bpm)
**hr_avg_prt_10s_10**		text	Average Heart Rate (bpm)
**hr_avg_prt_10s_11**		text	Average Heart Rate (bpm)
**hr_avg_prt_10s_12**		text	Average Heart Rate (bpm)
**hr_avg_prt_10s_13**		text	Average Heart Rate (bpm)
**hr_avg_prt_10s_14**		text	Average Heart Rate (bpm)
**hr_avg_prt_10s_15**		text	Average Heart Rate (bpm)
**hr_avg_prt_10s_16**		text	Average Heart Rate (bpm)

**hr_avg_prt_10s_17**		text	Average Heart Rate (bpm)
**hr_avg_prt_10s_18**		text	Average Heart Rate (bpm)
**hr_avg_prt_10s_19**		text	Average Heart Rate (bpm)
**hr_avg_prt_10s_20**		text	Average Heart Rate (bpm)
**hr_avg_prt_10s_21**		text	Average Heart Rate (bpm)
**hr_avg_prt_10s_22**		text	Average Heart Rate (bpm)
**hr_avg_prt_10s_23**		text	Average Heart Rate (bpm)
**hr_avg_prt_10s_24**		text	Average Heart Rate (bpm)
**hr_avg_prt_10s_25**		text	Average Heart Rate (bpm)
**hr_avg_prt_10s_26**		text	Average Heart Rate (bpm)
**hr_avg_prt_10s_27**		text	Average Heart Rate (bpm)
**hr_avg_prt_10s_28**		text	Average Heart Rate (bpm)
**hr_avg_prt_10s_29**		text	Average Heart Rate (bpm)
**hr_avg_prt_10s_30**		text	Average Heart Rate (bpm)
**sbp_avd_prt_10s_1**		text	Systolic blood pressure value in mmHg
**sbp_avd_prt_10s_2**		text	Systolic blood pressure value in mmHg
**sbp_avd_prt_10s_3**		text	Systolic blood pressure value in mmHg

**sbp_avd_prt_10s_4**		text	Systolic blood pressure value in mmHg
**sbp_avd_prt_10s_5**		text	Systolic blood pressure value in mmHg
**sbp_avd_prt_10s_6**		text	Systolic blood pressure value in mmHg
**sbp_avd_prt_10s_7**		text	Systolic blood pressure value in mmHg
**sbp_avd_prt_10s_8**		text	Systolic blood pressure value in mmHg
**sbp_avd_prt_10s_9**		text	Systolic blood pressure value in mmHg
**sbp_avd_prt_10s_10**		text	Systolic blood pressure value in mmHg
**sbp_avd_prt_10s_11**		text	Systolic blood pressure value in mmHg
**sbp_avd_prt_10s_12**		text	Systolic blood pressure value in mmHg
**sbp_avd_prt_10s_13**		text	Systolic blood pressure value in mmHg
**sbp_avd_prt_10s_14**		text	Systolic blood pressure value in mmHg
**sbp_avd_prt_10s_15**		text	Systolic blood pressure value in mmHg
**sbp_avd_prt_10s_16**		text	Systolic blood pressure value in mmHg
**sbp_avd_prt_10s_17**		text	Systolic blood pressure value in mmHg
**sbp_avd_prt_10s_18**		text	Systolic blood pressure value in mmHg
**sbp_avd_prt_10s_19**		text	Systolic blood pressure value in mmHg
**sbp_avd_prt_10s_20**		text	Systolic blood pressure value in mmHg

**sbp_avd_prt_10s_21**		text	Systolic blood pressure value in mmHg
**sbp_avd_prt_10s_22**		text	Systolic blood pressure value in mmHg
**sbp_avd_prt_10s_23**		text	Systolic blood pressure value in mmHg
**sbp_avd_prt_10s_24**		text	Systolic blood pressure value in mmHg
**sbp_avd_prt_10s_25**		text	Systolic blood pressure value in mmHg
**sbp_avd_prt_10s_26**		text	Systolic blood pressure value in mmHg
**sbp_avd_prt_10s_27**		text	Systolic blood pressure value in mmHg
**sbp_avd_prt_10s_28**		text	Systolic blood pressure value in mmHg
**sbp_avd_prt_10s_29**		text	Systolic blood pressure value in mmHg
**sbp_avd_prt_10s_30**		text	Systolic blood pressure value in mmHg
**dbp_avd_prt_10s_1**		text	Diastolic blood pressure valuein mmHg
**dbp_avd_prt_10s_2**		text	Diastolic blood pressure valuein mmHg
**dbp_avd_prt_10s_3**		text	Diastolic blood pressure valuein mmHg
**dbp_avd_prt_10s_4**		text	Diastolic blood pressure valuein mmHg
**dbp_avd_prt_10s_5**		text	Diastolic blood pressure valuein mmHg
**dbp_avd_prt_10s_6**		text	Diastolic blood pressure valuein mmHg
**dbp_avd_prt_10s_7**		text	Diastolic blood pressure valuein mmHg

**dbp_avd_prt_10s_8**		text	Diastolic blood pressure valuein mmHg
**dbp_avd_prt_10s_9**		text	Diastolic blood pressure valuein mmHg
**dbp_avd_prt_10s_10**		text	Diastolic blood pressure valuein mmHg
**dbp_avd_prt_10s_11**		text	Diastolic blood pressure valuein mmHg
**dbp_avd_prt_10s_12**		text	Diastolic blood pressure valuein mmHg
**dbp_avd_prt_10s_13**		text	Diastolic blood pressure valuein mmHg
**dbp_avd_prt_10s_14**		text	Diastolic blood pressure valuein mmHg
**dbp_avd_prt_10s_15**		text	Diastolic blood pressure valuein mmHg
**dbp_avd_prt_10s_16**		text	Diastolic blood pressure valuein mmHg
**dbp_avd_prt_10s_17**		text	Diastolic blood pressure valuein mmHg
**dbp_avd_prt_10s_18**		text	Diastolic blood pressure valuein mmHg
**dbp_avd_prt_10s_19**		text	Diastolic blood pressure valuein mmHg
**dbp_avd_prt_10s_20**		text	Diastolic blood pressure valuein mmHg
**dbp_avd_prt_10s_21**		text	Diastolic blood pressure valuein mmHg
**dbp_avd_prt_10s_22**		text	Diastolic blood pressure valuein mmHg
**dbp_avd_prt_10s_23**		text	Diastolic blood pressure valuein mmHg
**dbp_avd_prt_10s_24**		text	Diastolic blood pressure valuein mmHg

**dbp_avd_prt_10s_25**		text	Diastolic blood pressure valuein mmHg
**dbp_avd_prt_10s_26**		text	Diastolic blood pressure valuein mmHg
**dbp_avd_prt_10s_27**		text	Diastolic blood pressure valuein mmHg
**dbp_avd_prt_10s_28**		text	Diastolic blood pressure valuein mmHg
**dbp_avd_prt_10s_29**		text	Diastolic blood pressure valuein mmHg
**dbp_avd_prt_10s_30**		text	Diastolic blood pressure valuein mmHg
**hr_avg_t_10s_1**	10 second epoch--Task	text	Average Heart Rate (bpm)
**hr_avg_t_10s_2**		text	Average Heart Rate (bpm)
**hr_avg_t_10s_3**		text	Average Heart Rate (bpm)
**hr_avg_t_10s_4**		text	Average Heart Rate (bpm)
**hr_avg_t_10s_5**		text	Average Heart Rate (bpm)
**hr_avg_t_10s_6**		text	Average Heart Rate (bpm)
**hr_avg_t_10s_7**		text	Average Heart Rate (bpm)
**hr_avg_t_10s_8**		text	Average Heart Rate (bpm)
**hr_avg_t_10s_9**		text	Average Heart Rate (bpm)
**hr_avg_t_10s_10**		text	Average Heart Rate (bpm)
**hr_avg_t_10s_11**		text	Average Heart Rate (bpm)

**hr_avg_t_10s_12**		text	Average Heart Rate (bpm)
**hr_avg_t_10s_13**		text	Average Heart Rate (bpm)
**hr_avg_t_10s_14**		text	Average Heart Rate (bpm)
**hr_avg_t_10s_15**		text	Average Heart Rate (bpm)
**hr_avg_t_10s_16**		text	Average Heart Rate (bpm)
**hr_avg_t_10s_17**		text	Average Heart Rate (bpm)
**hr_avg_t_10s_18**		text	Average Heart Rate (bpm)
**hr_avg_t_10s_19**		text	Average Heart Rate (bpm)
**hr_avg_t_10s_20**		text	Average Heart Rate (bpm)
**hr_avg_t_10s_21**		text	Average Heart Rate (bpm)
**hr_avg_t_10s_22**		text	Average Heart Rate (bpm)
**hr_avg_t_10s_23**		text	Average Heart Rate (bpm)
**hr_avg_t_10s_24**		text	Average Heart Rate (bpm)
**hr_avg_t_10s_25**		text	Average Heart Rate (bpm)
**hr_avg_t_10s_26**		text	Average Heart Rate (bpm)
**hr_avg_t_10s_27**		text	Average Heart Rate (bpm)
**hr_avg_t_10s_28**		text	Average Heart Rate (bpm)

**hr_avg_t_10s_29**		text	Average Heart Rate (bpm)
**hr_avg_t_10s_30**		text	Average Heart Rate (bpm)
**sbp_avd_t_10s_1**		text	Systolic blood pressure value in mmHg
**sbp_avd_t_10s_2**		text	Systolic blood pressure value in mmHg
**sbp_avd_t_10s_3**		text	Systolic blood pressure value in mmHg
**sbp_avd_t_10s_4**		text	Systolic blood pressure value in mmHg
**sbp_avd_t_10s_5**		text	Systolic blood pressure value in mmHg
**sbp_avd_t_10s_6**		text	Systolic blood pressure value in mmHg
**sbp_avd_t_10s_7**		text	Systolic blood pressure value in mmHg
**sbp_avd_t_10s_8**		text	Systolic blood pressure value in mmHg
**sbp_avd_t_10s_9**		text	Systolic blood pressure value in mmHg
**sbp_avd_t_10s_10**		text	Systolic blood pressure value in mmHg
**sbp_avd_t_10s_11**		text	Systolic blood pressure value in mmHg
**sbp_avd_t_10s_12**		text	Systolic blood pressure value in mmHg
**sbp_avd_t_10s_13**		text	Systolic blood pressure value in mmHg
**sbp_avd_t_10s_14**		text	Systolic blood pressure value in mmHg
**sbp_avd_t_10s_15**		text	Systolic blood pressure value in mmHg

**sbp_avd_t_10s_16**		text	Systolic blood pressure value in mmHg
**sbp_avd_t_10s_17**		text	Systolic blood pressure value in mmHg
**sbp_avd_t_10s_18**		text	Systolic blood pressure value in mmHg
**sbp_avd_t_10s_19**		text	Systolic blood pressure value in mmHg
**sbp_avd_t_10s_20**		text	Systolic blood pressure value in mmHg
**sbp_avd_t_10s_21**		text	Systolic blood pressure value in mmHg
**sbp_avd_t_10s_22**		text	Systolic blood pressure value in mmHg
**sbp_avd_t_10s_23**		text	Systolic blood pressure value in mmHg
**sbp_avd_t_10s_24**		text	Systolic blood pressure value in mmHg
**sbp_avd_t_10s_25**		text	Systolic blood pressure value in mmHg
**sbp_avd_t_10s_26**		text	Systolic blood pressure value in mmHg
**sbp_avd_t_10s_27**		text	Systolic blood pressure value in mmHg
**sbp_avd_t_10s_28**		text	Systolic blood pressure value in mmHg
**sbp_avd_t_10s_29**		text	Systolic blood pressure value in mmHg
**sbp_avd_t_10s_30**		text	Systolic blood pressure value in mmHg
**dbp_avd_t_10s_1**		text	Diastolic blood pressure valuein mmHg
**dbp_avd_t_10s_2**		text	Diastolic blood pressure valuein mmHg

**dbp_avd_t_10s_3**		text	Diastolic blood pressure valuein mmHg
**dbp_avd_t_10s_4**		text	Diastolic blood pressure valuein mmHg
**dbp_avd_t_10s_5**		text	Diastolic blood pressure valuein mmHg
**dbp_avd_t_10s_6**		text	Diastolic blood pressure valuein mmHg
**dbp_avd_t_10s_7**		text	Diastolic blood pressure valuein mmHg
**dbp_avd_t_10s_8**		text	Diastolic blood pressure valuein mmHg
**dbp_avd_t_10s_9**		text	Diastolic blood pressure valuein mmHg
**dbp_avd_t_10s_10**		text	Diastolic blood pressure valuein mmHg
**dbp_avd_t_10s_11**		text	Diastolic blood pressure valuein mmHg
**dbp_avd_t_10s_12**		text	Diastolic blood pressure valuein mmHg
**dbp_avd_t_10s_13**		text	Diastolic blood pressure valuein mmHg
**dbp_avd_t_10s_14**		text	Diastolic blood pressure valuein mmHg
**dbp_avd_t_10s_15**		text	Diastolic blood pressure valuein mmHg
**dbp_avd_t_10s_16**		text	Diastolic blood pressure valuein mmHg
**dbp_avd_t_10s_17**		text	Diastolic blood pressure valuein mmHg
**dbp_avd_t_10s_18**		text	Diastolic blood pressure valuein mmHg
**dbp_avd_t_10s_19**		text	Diastolic blood pressure valuein mmHg

**dbp_avd_t_10s_20**		text	Diastolic blood pressure valuein mmHg
**dbp_avd_t_10s_21**		text	Diastolic blood pressure valuein mmHg
**dbp_avd_t_10s_22**		text	Diastolic blood pressure valuein mmHg
**dbp_avd_t_10s_23**		text	Diastolic blood pressure valuein mmHg
**dbp_avd_t_10s_24**		text	Diastolic blood pressure valuein mmHg
**dbp_avd_t_10s_25**		text	Diastolic blood pressure valuein mmHg
**dbp_avd_t_10s_26**		text	Diastolic blood pressure valuein mmHg
**dbp_avd_t_10s_27**		text	Diastolic blood pressure valuein mmHg
**dbp_avd_t_10s_28**		text	Diastolic blood pressure valuein mmHg
**dbp_avd_t_10s_29**		text	Diastolic blood pressure valuein mmHg
**dbp_avd_t_10s_30**		text	Diastolic blood pressure valuein mmHg
**hr_avg_p5_10s_1**	10 second epoch--Post5	text	Average Heart Rate (bpm)
**hr_avg_p5_10s_2**		text	Average Heart Rate (bpm)
**hr_avg_p5_10s_3**		text	Average Heart Rate (bpm)
**hr_avg_p5_10s_4**		text	Average Heart Rate (bpm)
**hr_avg_p5_10s_5**		text	Average Heart Rate (bpm)

**hr_avg_p5_10s_6**		text	Average Heart Rate (bpm)
**hr_avg_p5_10s_7**		text	Average Heart Rate (bpm)
**hr_avg_p5_10s_8**		text	Average Heart Rate (bpm)
**hr_avg_p5_10s_9**		text	Average Heart Rate (bpm)
**hr_avg_p5_10s_10**		text	Average Heart Rate (bpm)
**hr_avg_p5_10s_11**		text	Average Heart Rate (bpm)
**hr_avg_p5_10s_12**		text	Average Heart Rate (bpm)
**hr_avg_p5_10s_13**		text	Average Heart Rate (bpm)
**hr_avg_p5_10s_14**		text	Average Heart Rate (bpm)
**hr_avg_p5_10s_15**		text	Average Heart Rate (bpm)
**hr_avg_p5_10s_16**		text	Average Heart Rate (bpm)
**hr_avg_p5_10s_17**		text	Average Heart Rate (bpm)
**hr_avg_p5_10s_18**		text	Average Heart Rate (bpm)
**hr_avg_p5_10s_19**		text	Average Heart Rate (bpm)
**hr_avg_p5_10s_20**		text	Average Heart Rate (bpm)
**hr_avg_p5_10s_21**		text	Average Heart Rate (bpm)
**hr_avg_p5_10s_22**		text	Average Heart Rate (bpm)

**hr_avg_p5_10s_23**		text	Average Heart Rate (bpm)
**hr_avg_p5_10s_24**		text	Average Heart Rate (bpm)
**hr_avg_p5_10s_25**		text	Average Heart Rate (bpm)
**hr_avg_p5_10s_26**		text	Average Heart Rate (bpm)
**hr_avg_p5_10s_27**		text	Average Heart Rate (bpm)
**hr_avg_p5_10s_28**		text	Average Heart Rate (bpm)
**hr_avg_p5_10s_29**		text	Average Heart Rate (bpm)
**hr_avg_p5_10s_30**		text	Average Heart Rate (bpm)
**sbp_avd_p5_10s_1**		text	Systolic blood pressure value in mmHg
**sbp_avd_p5_10s_2**		text	Systolic blood pressure value in mmHg
**sbp_avd_p5_10s_3**		text	Systolic blood pressure value in mmHg
**sbp_avd_p5_10s_4**		text	Systolic blood pressure value in mmHg
**sbp_avd_p5_10s_5**		text	Systolic blood pressure value in mmHg
**sbp_avd_p5_10s_6**		text	Systolic blood pressure value in mmHg
**sbp_avd_p5_10s_7**		text	Systolic blood pressure value in mmHg
**sbp_avd_p5_10s_8**		text	Systolic blood pressure value in mmHg
**sbp_avd_p5_10s_9**		text	Systolic blood pressure value in mmHg

**sbp_avd_p5_10s_10**		text	Systolic blood pressure value in mmHg
**sbp_avd_p5_10s_11**		text	Systolic blood pressure value in mmHg
**sbp_avd_p5_10s_12**		text	Systolic blood pressure value in mmHg
**sbp_avd_p5_10s_13**		text	Systolic blood pressure value in mmHg
**sbp_avd_p5_10s_14**		text	Systolic blood pressure value in mmHg
**sbp_avd_p5_10s_15**		text	Systolic blood pressure value in mmHg
**sbp_avd_p5_10s_16**		text	Systolic blood pressure value in mmHg
**sbp_avd_p5_10s_17**		text	Systolic blood pressure value in mmHg
**sbp_avd_p5_10s_18**		text	Systolic blood pressure value in mmHg
**sbp_avd_p5_10s_19**		text	Systolic blood pressure value in mmHg
**sbp_avd_p5_10s_20**		text	Systolic blood pressure value in mmHg
**sbp_avd_p5_10s_21**		text	Systolic blood pressure value in mmHg
**sbp_avd_p5_10s_22**		text	Systolic blood pressure value in mmHg
**sbp_avd_p5_10s_23**		text	Systolic blood pressure value in mmHg
**sbp_avd_p5_10s_24**		text	Systolic blood pressure value in mmHg
**sbp_avd_p5_10s_25**		text	Systolic blood pressure value in mmHg
**sbp_avd_p5_10s_26**		text	Systolic blood pressure value in mmHg

**sbp_avd_p5_10s_27**		text	Systolic blood pressure value in mmHg
**sbp_avd_p5_10s_28**		text	Systolic blood pressure value in mmHg
**sbp_avd_p5_10s_29**		text	Systolic blood pressure value in mmHg
**sbp_avd_p5_10s_30**		text	Systolic blood pressure value in mmHg
**dbp_avd_p5_10s_1**		text	Diastolic blood pressure valuein mmHg
**dbp_avd_p5_10s_2**		text	Diastolic blood pressure valuein mmHg
**dbp_avd_p5_10s_3**		text	Diastolic blood pressure valuein mmHg
**dbp_avd_p5_10s_4**		text	Diastolic blood pressure valuein mmHg
**dbp_avd_p5_10s_5**		text	Diastolic blood pressure valuein mmHg
**dbp_avd_p5_10s_6**		text	Diastolic blood pressure valuein mmHg
**dbp_avd_p5_10s_7**		text	Diastolic blood pressure valuein mmHg
**dbp_avd_p5_10s_8**		text	Diastolic blood pressure valuein mmHg
**dbp_avd_p5_10s_9**		text	Diastolic blood pressure valuein mmHg
**dbp_avd_p5_10s_10**		text	Diastolic blood pressure valuein mmHg
**dbp_avd_p5_10s_11**		text	Diastolic blood pressure valuein mmHg
**dbp_avd_p5_10s_12**		text	Diastolic blood pressure valuein mmHg
**dbp_avd_p5_10s_13**		text	Diastolic blood pressure valuein mmHg

**dbp_avd_p5_10s_14**		text	Diastolic blood pressure valuein mmHg
**dbp_avd_p5_10s_15**		text	Diastolic blood pressure valuein mmHg
**dbp_avd_p5_10s_16**		text	Diastolic blood pressure valuein mmHg
**dbp_avd_p5_10s_17**		text	Diastolic blood pressure valuein mmHg
**dbp_avd_p5_10s_18**		text	Diastolic blood pressure valuein mmHg
**dbp_avd_p5_10s_19**		text	Diastolic blood pressure valuein mmHg
**dbp_avd_p5_10s_20**		text	Diastolic blood pressure valuein mmHg
**dbp_avd_p5_10s_21**		text	Diastolic blood pressure valuein mmHg
**dbp_avd_p5_10s_22**		text	Diastolic blood pressure valuein mmHg
**dbp_avd_p5_10s_23**		text	Diastolic blood pressure valuein mmHg
**dbp_avd_p5_10s_24**		text	Diastolic blood pressure valuein mmHg
**dbp_avd_p5_10s_25**		text	Diastolic blood pressure valuein mmHg
**dbp_avd_p5_10s_26**		text	Diastolic blood pressure valuein mmHg
**dbp_avd_p5_10s_27**		text	Diastolic blood pressure valuein mmHg
**dbp_avd_p5_10s_28**		text	Diastolic blood pressure valuein mmHg
**dbp_avd_p5_10s_29**		text	Diastolic blood pressure valuein mmHg
**dbp_avd_p5_10s_30**		text	Diastolic blood pressure valuein mmHg

**hr_avg_p10_10s_1**	10 second epoch--Post10	text	Average Heart Rate (bpm)
**hr_avg_p10_10s_2**		text	Average Heart Rate (bpm)
**hr_avg_p10_10s_3**		text	Average Heart Rate (bpm)
**hr_avg_p10_10s_4**		text	Average Heart Rate (bpm)
**hr_avg_p10_10s_5**		text	Average Heart Rate (bpm)
**hr_avg_p10_10s_6**		text	Average Heart Rate (bpm)
**hr_avg_p10_10s_7**		text	Average Heart Rate (bpm)
**hr_avg_p10_10s_8**		text	Average Heart Rate (bpm)
**hr_avg_p10_10s_9**		text	Average Heart Rate (bpm)
**hr_avg_p10_10s_10**		text	Average Heart Rate (bpm)
**hr_avg_p10_10s_11**		text	Average Heart Rate (bpm)
**hr_avg_p10_10s_12**		text	Average Heart Rate (bpm)
**hr_avg_p10_10s_13**		text	Average Heart Rate (bpm)
**hr_avg_p10_10s_14**		text	Average Heart Rate (bpm)
**hr_avg_p10_10s_15**		text	Average Heart Rate (bpm)
**hr_avg_p10_10s_16**		text	Average Heart Rate (bpm)

**hr_avg_p10_10s_17**		text	Average Heart Rate (bpm)
**hr_avg_p10_10s_18**		text	Average Heart Rate (bpm)
**hr_avg_p10_10s_19**		text	Average Heart Rate (bpm)
**hr_avg_p10_10s_20**		text	Average Heart Rate (bpm)
**hr_avg_p10_10s_21**		text	Average Heart Rate (bpm)
**hr_avg_p10_10s_22**		text	Average Heart Rate (bpm)
**hr_avg_p10_10s_23**		text	Average Heart Rate (bpm)
**hr_avg_p10_10s_24**		text	Average Heart Rate (bpm)
**hr_avg_p10_10s_25**		text	Average Heart Rate (bpm)
**hr_avg_p10_10s_26**		text	Average Heart Rate (bpm)
**hr_avg_p10_10s_27**		text	Average Heart Rate (bpm)
**hr_avg_p10_10s_28**		text	Average Heart Rate (bpm)
**hr_avg_p10_10s_29**		text	Average Heart Rate (bpm)
**hr_avg_p10_10s_30**		text	Average Heart Rate (bpm)
**sbp_avd_p10_10s_1**		text	Systolic blood pressure value in mmHg
**sbp_avd_p10_10s_2**		text	Systolic blood pressure value in mmHg
**sbp_avd_p10_10s_3**		text	Systolic blood pressure value in mmHg

**sbp_avd_p10_10s_4**		text	Systolic blood pressure value in mmHg
**sbp_avd_p10_10s_5**		text	Systolic blood pressure value in mmHg
**sbp_avd_p10_10s_6**		text	Systolic blood pressure value in mmHg
**sbp_avd_p10_10s_7**		text	Systolic blood pressure value in mmHg
**sbp_avd_p10_10s_8**		text	Systolic blood pressure value in mmHg
**sbp_avd_p10_10s_9**		text	Systolic blood pressure value in mmHg
**sbp_avd_p10_10s_10**		text	Systolic blood pressure value in mmHg
**sbp_avd_p10_10s_11**		text	Systolic blood pressure value in mmHg
**sbp_avd_p10_10s_12**		text	Systolic blood pressure value in mmHg
**sbp_avd_p10_10s_13**		text	Systolic blood pressure value in mmHg
**sbp_avd_p10_10s_14**		text	Systolic blood pressure value in mmHg
**sbp_avd_p10_10s_15**		text	Systolic blood pressure value in mmHg
**sbp_avd_p10_10s_16**		text	Systolic blood pressure value in mmHg
**sbp_avd_p10_10s_17**		text	Systolic blood pressure value in mmHg
**sbp_avd_p10_10s_18**		text	Systolic blood pressure value in mmHg
**sbp_avd_p10_10s_19**		text	Systolic blood pressure value in mmHg
**sbp_avd_p10_10s_20**		text	Systolic blood pressure value in mmHg

**sbp_avd_p10_10s_21**		text	Systolic blood pressure value in mmHg
**sbp_avd_p10_10s_22**		text	Systolic blood pressure value in mmHg
**sbp_avd_p10_10s_23**		text	Systolic blood pressure value in mmHg
**sbp_avd_p10_10s_24**		text	Systolic blood pressure value in mmHg
**sbp_avd_p10_10s_25**		text	Systolic blood pressure value in mmHg
**sbp_avd_p10_10s_26**		text	Systolic blood pressure value in mmHg
**sbp_avd_p10_10s_27**		text	Systolic blood pressure value in mmHg
**sbp_avd_p10_10s_28**		text	Systolic blood pressure value in mmHg
**sbp_avd_p10_10s_29**		text	Systolic blood pressure value in mmHg
**sbp_avd_p10_10s_30**		text	Systolic blood pressure value in mmHg
**dbp_avd_p10_10s_1**		text	Diastolic blood pressure valuein mmHg
**dbp_avd_p10_10s_2**		text	Diastolic blood pressure valuein mmHg
**dbp_avd_p10_10s_3**		text	Diastolic blood pressure valuein mmHg
**dbp_avd_p10_10s_4**		text	Diastolic blood pressure valuein mmHg
**dbp_avd_p10_10s_5**		text	Diastolic blood pressure valuein mmHg
**dbp_avd_p10_10s_6**		text	Diastolic blood pressure valuein mmHg
**dbp_avd_p10_10s_7**		text	Diastolic blood pressure valuein mmHg

**dbp_avd_p10_10s_8**		text	Diastolic blood pressure valuein mmHg
**dbp_avd_p10_10s_9**		text	Diastolic blood pressure valuein mmHg
**dbp_avd_p10_10s_10**		text	Diastolic blood pressure valuein mmHg
**dbp_avd_p10_10s_11**		text	Diastolic blood pressure valuein mmHg
**dbp_avd_p10_10s_12**		text	Diastolic blood pressure valuein mmHg
**dbp_avd_p10_10s_13**		text	Diastolic blood pressure valuein mmHg
**dbp_avd_p10_10s_14**		text	Diastolic blood pressure valuein mmHg
**dbp_avd_p10_10s_15**		text	Diastolic blood pressure valuein mmHg
**dbp_avd_p10_10s_16**		text	Diastolic blood pressure valuein mmHg
**dbp_avd_p10_10s_17**		text	Diastolic blood pressure valuein mmHg
**dbp_avd_p10_10s_18**		text	Diastolic blood pressure valuein mmHg
**dbp_avd_p10_10s_19**		text	Diastolic blood pressure valuein mmHg
**dbp_avd_p10_10s_20**		text	Diastolic blood pressure valuein mmHg
**dbp_avd_p10_10s_21**		text	Diastolic blood pressure valuein mmHg
**dbp_avd_p10_10s_22**		text	Diastolic blood pressure valuein mmHg
**dbp_avd_p10_10s_23**		text	Diastolic blood pressure valuein mmHg
**dbp_avd_p10_10s_24**		text	Diastolic blood pressure valuein mmHg

**dbp_avd_p10_10s_25**		text	Diastolic blood pressure valuein mmHg
**dbp_avd_p10_10s_26**		text	Diastolic blood pressure valuein mmHg
**dbp_avd_p10_10s_27**		text	Diastolic blood pressure valuein mmHg
**dbp_avd_p10_10s_28**		text	Diastolic blood pressure valuein mmHg
**dbp_avd_p10_10s_29**		text	Diastolic blood pressure valuein mmHg
**dbp_avd_p10_10s_30**		text	Diastolic blood pressure valuein mmHg
**hr_avg_p20_10s_1**	10 second epoch--Post20	text	Average Heart Rate (bpm)
**hr_avg_p20_10s_2**		text	Average Heart Rate (bpm)
**hr_avg_p20_10s_3**		text	Average Heart Rate (bpm)
**hr_avg_p20_10s_4**		text	Average Heart Rate (bpm)
**hr_avg_p20_10s_5**		text	Average Heart Rate (bpm)
**hr_avg_p20_10s_6**		text	Average Heart Rate (bpm)
**hr_avg_p20_10s_7**		text	Average Heart Rate (bpm)
**hr_avg_p20_10s_8**		text	Average Heart Rate (bpm)
**hr_avg_p20_10s_9**		text	Average Heart Rate (bpm)
**hr_avg_p20_10s_10**		text	Average Heart Rate (bpm)

**hr_avg_p20_10s_11**		text	Average Heart Rate (bpm)
**hr_avg_p20_10s_12**		text	Average Heart Rate (bpm)
**hr_avg_p20_10s_13**		text	Average Heart Rate (bpm)
**hr_avg_p20_10s_14**		text	Average Heart Rate (bpm)
**hr_avg_p20_10s_15**		text	Average Heart Rate (bpm)
**hr_avg_p20_10s_16**		text	Average Heart Rate (bpm)
**hr_avg_p20_10s_17**		text	Average Heart Rate (bpm)
**hr_avg_p20_10s_18**		text	Average Heart Rate (bpm)
**hr_avg_p20_10s_19**		text	Average Heart Rate (bpm)
**hr_avg_p20_10s_20**		text	Average Heart Rate (bpm)
**hr_avg_p20_10s_21**		text	Average Heart Rate (bpm)
**hr_avg_p20_10s_22**		text	Average Heart Rate (bpm)
**hr_avg_p20_10s_23**		text	Average Heart Rate (bpm)
**hr_avg_p20_10s_24**		text	Average Heart Rate (bpm)
**hr_avg_p20_10s_25**		text	Average Heart Rate (bpm)
**hr_avg_p20_10s_26**		text	Average Heart Rate (bpm)
**hr_avg_p20_10s_27**		text	Average Heart Rate (bpm)

**hr_avg_p20_10s_28**		text	Average Heart Rate (bpm)
**hr_avg_p20_10s_29**		text	Average Heart Rate (bpm)
**hr_avg_p20_10s_30**		text	Average Heart Rate (bpm)
**sbp_avd_p20_10s_1**		text	Systolic blood pressure value in mmHg
**sbp_avd_p20_10s_2**		text	Systolic blood pressure value in mmHg
**sbp_avd_p20_10s_3**		text	Systolic blood pressure value in mmHg
**sbp_avd_p20_10s_4**		text	Systolic blood pressure value in mmHg
**sbp_avd_p20_10s_5**		text	Systolic blood pressure value in mmHg
**sbp_avd_p20_10s_6**		text	Systolic blood pressure value in mmHg
**sbp_avd_p20_10s_7**		text	Systolic blood pressure value in mmHg
**sbp_avd_p20_10s_8**		text	Systolic blood pressure value in mmHg
**sbp_avd_p20_10s_9**		text	Systolic blood pressure value in mmHg
**sbp_avd_p20_10s_10**		text	Systolic blood pressure value in mmHg
**sbp_avd_p20_10s_11**		text	Systolic blood pressure value in mmHg
**sbp_avd_p20_10s_12**		text	Systolic blood pressure value in mmHg
**sbp_avd_p20_10s_13**		text	Systolic blood pressure value in mmHg
**sbp_avd_p20_10s_14**		text	Systolic blood pressure value in mmHg

**sbp_avd_p20_10s_15**		text	Systolic blood pressure value in mmHg
**sbp_avd_p20_10s_16**		text	Systolic blood pressure value in mmHg
**sbp_avd_p20_10s_17**		text	Systolic blood pressure value in mmHg
**sbp_avd_p20_10s_18**		text	Systolic blood pressure value in mmHg
**sbp_avd_p20_10s_19**		text	Systolic blood pressure value in mmHg
**sbp_avd_p20_10s_20**		text	Systolic blood pressure value in mmHg
**sbp_avd_p20_10s_21**		text	Systolic blood pressure value in mmHg
**sbp_avd_p20_10s_22**		text	Systolic blood pressure value in mmHg
**sbp_avd_p20_10s_23**		text	Systolic blood pressure value in mmHg
**sbp_avd_p20_10s_24**		text	Systolic blood pressure value in mmHg
**sbp_avd_p20_10s_25**		text	Systolic blood pressure value in mmHg
**sbp_avd_p20_10s_26**		text	Systolic blood pressure value in mmHg
**sbp_avd_p20_10s_27**		text	Systolic blood pressure value in mmHg
**sbp_avd_p20_10s_28**		text	Systolic blood pressure value in mmHg
**sbp_avd_p20_10s_29**		text	Systolic blood pressure value in mmHg
**sbp_avd_p20_10s_30**		text	Systolic blood pressure value in mmHg
**dbp_avd_p20_10s_1**		text	Diastolic blood pressure valuein mmHg

**dbp_avd_p20_10s_2**		text	Diastolic blood pressure valuein mmHg
**dbp_avd_p20_10s_3**		text	Diastolic blood pressure valuein mmHg
**dbp_avd_p20_10s_4**		text	Diastolic blood pressure valuein mmHg
**dbp_avd_p20_10s_5**		text	Diastolic blood pressure valuein mmHg
**dbp_avd_p20_10s_6**		text	Diastolic blood pressure valuein mmHg
**dbp_avd_p20_10s_7**		text	Diastolic blood pressure valuein mmHg
**dbp_avd_p20_10s_8**		text	Diastolic blood pressure valuein mmHg
**dbp_avd_p20_10s_9**		text	Diastolic blood pressure valuein mmHg
**dbp_avd_p20_10s_10**		text	Diastolic blood pressure valuein mmHg
**dbp_avd_p20_10s_11**		text	Diastolic blood pressure valuein mmHg
**dbp_avd_p20_10s_12**		text	Diastolic blood pressure valuein mmHg
**dbp_avd_p20_10s_13**		text	Diastolic blood pressure valuein mmHg
**dbp_avd_p20_10s_14**		text	Diastolic blood pressure valuein mmHg
**dbp_avd_p20_10s_15**		text	Diastolic blood pressure valuein mmHg
**dbp_avd_p20_10s_16**		text	Diastolic blood pressure valuein mmHg
**dbp_avd_p20_10s_17**		text	Diastolic blood pressure valuein mmHg
**dbp_avd_p20_10s_18**		text	Diastolic blood pressure valuein mmHg

**dbp_avd_p20_10s_19**		text	Diastolic blood pressure valuein mmHg
**dbp_avd_p20_10s_20**		text	Diastolic blood pressure valuein mmHg
**dbp_avd_p20_10s_21**		text	Diastolic blood pressure valuein mmHg
**dbp_avd_p20_10s_22**		text	Diastolic blood pressure valuein mmHg
**dbp_avd_p20_10s_23**		text	Diastolic blood pressure valuein mmHg
**dbp_avd_p20_10s_24**		text	Diastolic blood pressure valuein mmHg
**dbp_avd_p20_10s_25**		text	Diastolic blood pressure valuein mmHg
**dbp_avd_p20_10s_26**		text	Diastolic blood pressure valuein mmHg
**dbp_avd_p20_10s_27**		text	Diastolic blood pressure valuein mmHg
**dbp_avd_p20_10s_28**		text	Diastolic blood pressure valuein mmHg
**dbp_avd_p20_10s_29**		text	Diastolic blood pressure valuein mmHg
**dbp_avd_p20_10s_30**		text	Diastolic blood pressure valuein mmHg
**hr_avg_p30_10s_1**	10 second epoch--Post30	text	Average Heart Rate (bpm)
**hr_avg_p30_10s_2**		text	Average Heart Rate (bpm)
**hr_avg_p30_10s_3**		text	Average Heart Rate (bpm)
**hr_avg_p30_10s_4**		text	Average Heart Rate (bpm)

**hr_avg_p30_10s_5**		text	Average Heart Rate (bpm)
**hr_avg_p30_10s_6**		text	Average Heart Rate (bpm)
**hr_avg_p30_10s_7**		text	Average Heart Rate (bpm)
**hr_avg_p30_10s_8**		text	Average Heart Rate (bpm)
**hr_avg_p30_10s_9**		text	Average Heart Rate (bpm)
**hr_avg_p30_10s_10**		text	Average Heart Rate (bpm)
**hr_avg_p30_10s_11**		text	Average Heart Rate (bpm)
**hr_avg_p30_10s_12**		text	Average Heart Rate (bpm)
**hr_avg_p30_10s_13**		text	Average Heart Rate (bpm)
**hr_avg_p30_10s_14**		text	Average Heart Rate (bpm)
**hr_avg_p30_10s_15**		text	Average Heart Rate (bpm)
**hr_avg_p30_10s_16**		text	Average Heart Rate (bpm)
**hr_avg_p30_10s_17**		text	Average Heart Rate (bpm)
**hr_avg_p30_10s_18**		text	Average Heart Rate (bpm)
**hr_avg_p30_10s_19**		text	Average Heart Rate (bpm)
**hr_avg_p30_10s_20**		text	Average Heart Rate (bpm)
**hr_avg_p30_10s_21**		text	Average Heart Rate (bpm)

**hr_avg_p30_10s_22**		text	Average Heart Rate (bpm)
**hr_avg_p30_10s_23**		text	Average Heart Rate (bpm)
**hr_avg_p30_10s_24**		text	Average Heart Rate (bpm)
**hr_avg_p30_10s_25**		text	Average Heart Rate (bpm)
**hr_avg_p30_10s_26**		text	Average Heart Rate (bpm)
**hr_avg_p30_10s_27**		text	Average Heart Rate (bpm)
**hr_avg_p30_10s_28**		text	Average Heart Rate (bpm)
**hr_avg_p30_10s_29**		text	Average Heart Rate (bpm)
**hr_avg_p30_10s_30**		text	Average Heart Rate (bpm)
**sbp_avd_p30_10s_1**		text	Systolic blood pressure value in mmHg
**sbp_avd_p30_10s_2**		text	Systolic blood pressure value in mmHg
**sbp_avd_p30_10s_3**		text	Systolic blood pressure value in mmHg
**sbp_avd_p30_10s_4**		text	Systolic blood pressure value in mmHg
**sbp_avd_p30_10s_5**		text	Systolic blood pressure value in mmHg
**sbp_avd_p30_10s_6**		text	Systolic blood pressure value in mmHg
**sbp_avd_p30_10s_7**		text	Systolic blood pressure value in mmHg
**sbp_avd_p30_10s_8**		text	Systolic blood pressure value in mmHg

**sbp_avd_p30_10s_9**		text	Systolic blood pressure value in mmHg
**sbp_avd_p30_10s_10**		text	Systolic blood pressure value in mmHg
**sbp_avd_p30_10s_11**		text	Systolic blood pressure value in mmHg
**sbp_avd_p30_10s_12**		text	Systolic blood pressure value in mmHg
**sbp_avd_p30_10s_13**		text	Systolic blood pressure value in mmHg
**sbp_avd_p30_10s_14**		text	Systolic blood pressure value in mmHg
**sbp_avd_p30_10s_15**		text	Systolic blood pressure value in mmHg
**sbp_avd_p30_10s_16**		text	Systolic blood pressure value in mmHg
**sbp_avd_p30_10s_17**		text	Systolic blood pressure value in mmHg
**sbp_avd_p30_10s_18**		text	Systolic blood pressure value in mmHg
**sbp_avd_p30_10s_19**		text	Systolic blood pressure value in mmHg
**sbp_avd_p30_10s_20**		text	Systolic blood pressure value in mmHg
**sbp_avd_p30_10s_21**		text	Systolic blood pressure value in mmHg
**sbp_avd_p30_10s_22**		text	Systolic blood pressure value in mmHg
**sbp_avd_p30_10s_23**		text	Systolic blood pressure value in mmHg
**sbp_avd_p30_10s_24**		text	Systolic blood pressure value in mmHg
**sbp_avd_p30_10s_25**		text	Systolic blood pressure value in mmHg

**sbp_avd_p30_10s_26**		text	Systolic blood pressure value in mmHg
**sbp_avd_p30_10s_27**		text	Systolic blood pressure value in mmHg
**sbp_avd_p30_10s_28**		text	Systolic blood pressure value in mmHg
**sbp_avd_p30_10s_29**		text	Systolic blood pressure value in mmHg
**sbp_avd_p30_10s_30**		text	Systolic blood pressure value in mmHg
**dbp_avd_p30_10s_1**		text	Diastolic blood pressure valuein mmHg
**dbp_avd_p30_10s_2**		text	Diastolic blood pressure valuein mmHg
**dbp_avd_p30_10s_3**		text	Diastolic blood pressure valuein mmHg
**dbp_avd_p30_10s_4**		text	Diastolic blood pressure valuein mmHg
**dbp_avd_p30_10s_5**		text	Diastolic blood pressure valuein mmHg
**dbp_avd_p30_10s_6**		text	Diastolic blood pressure valuein mmHg
**dbp_avd_p30_10s_7**		text	Diastolic blood pressure valuein mmHg
**dbp_avd_p30_10s_8**		text	Diastolic blood pressure valuein mmHg
**dbp_avd_p30_10s_9**		text	Diastolic blood pressure valuein mmHg
**dbp_avd_p30_10s_10**		text	Diastolic blood pressure valuein mmHg
**dbp_avd_p30_10s_11**		text	Diastolic blood pressure valuein mmHg
**dbp_avd_p30_10s_12**		text	Diastolic blood pressure valuein mmHg

**dbp_avd_p30_10s_13**		text	Diastolic blood pressure valuein mmHg
**dbp_avd_p30_10s_14**		text	Diastolic blood pressure valuein mmHg
**dbp_avd_p30_10s_15**		text	Diastolic blood pressure valuein mmHg
**dbp_avd_p30_10s_16**		text	Diastolic blood pressure valuein mmHg
**dbp_avd_p30_10s_17**		text	Diastolic blood pressure valuein mmHg
**dbp_avd_p30_10s_18**		text	Diastolic blood pressure valuein mmHg
**dbp_avd_p30_10s_19**		text	Diastolic blood pressure valuein mmHg
**dbp_avd_p30_10s_20**		text	Diastolic blood pressure valuein mmHg
**dbp_avd_p30_10s_21**		text	Diastolic blood pressure valuein mmHg
**dbp_avd_p30_10s_22**		text	Diastolic blood pressure valuein mmHg
**dbp_avd_p30_10s_23**		text	Diastolic blood pressure valuein mmHg
**dbp_avd_p30_10s_24**		text	Diastolic blood pressure valuein mmHg
**dbp_avd_p30_10s_25**		text	Diastolic blood pressure valuein mmHg
**dbp_avd_p30_10s_26**		text	Diastolic blood pressure valuein mmHg
**dbp_avd_p30_10s_27**		text	Diastolic blood pressure valuein mmHg
**dbp_avd_p30_10s_28**		text	Diastolic blood pressure valuein mmHg
**dbp_avd_p30_10s_29**		text	Diastolic blood pressure valuein mmHg

**dbp_avd_p30_10s_30**		text	Diastolic blood pressure valuein mmHg
**hr_avg_p60_10s_1**	10 second epoch--Post60	text	Average Heart Rate (bpm)
**hr_avg_p60_10s_2**		text	Average Heart Rate (bpm)
**hr_avg_p60_10s_3**		text	Average Heart Rate (bpm)
**hr_avg_p60_10s_4**		text	Average Heart Rate (bpm)
**hr_avg_p60_10s_5**		text	Average Heart Rate (bpm)
**hr_avg_p60_10s_6**		text	Average Heart Rate (bpm)
**hr_avg_p60_10s_7**		text	Average Heart Rate (bpm)
**hr_avg_p60_10s_8**		text	Average Heart Rate (bpm)
**hr_avg_p60_10s_9**		text	Average Heart Rate (bpm)
**hr_avg_p60_10s_10**		text	Average Heart Rate (bpm)
**hr_avg_p60_10s_11**		text	Average Heart Rate (bpm)
**hr_avg_p60_10s_12**		text	Average Heart Rate (bpm)
**hr_avg_p60_10s_13**		text	Average Heart Rate (bpm)
**hr_avg_p60_10s_14**		text	Average Heart Rate (bpm)
**hr_avg_p60_10s_15**		text	Average Heart Rate (bpm)

**hr_avg_p60_10s_16**		text	Average Heart Rate (bpm)
**hr_avg_p60_10s_17**		text	Average Heart Rate (bpm)
**hr_avg_p60_10s_18**		text	Average Heart Rate (bpm)
**hr_avg_p60_10s_19**		text	Average Heart Rate (bpm)
**hr_avg_p60_10s_20**		text	Average Heart Rate (bpm)
**hr_avg_p60_10s_21**		text	Average Heart Rate (bpm)
**hr_avg_p60_10s_22**		text	Average Heart Rate (bpm)
**hr_avg_p60_10s_23**		text	Average Heart Rate (bpm)
**hr_avg_p60_10s_24**		text	Average Heart Rate (bpm)
**hr_avg_p60_10s_25**		text	Average Heart Rate (bpm)
**hr_avg_p60_10s_26**		text	Average Heart Rate (bpm)
**hr_avg_p60_10s_27**		text	Average Heart Rate (bpm)
**hr_avg_p60_10s_28**		text	Average Heart Rate (bpm)
**hr_avg_p60_10s_29**		text	Average Heart Rate (bpm)
**hr_avg_p60_10s_30**		text	Average Heart Rate (bpm)
**sbp_avd_p60_10s_1**		text	Systolic blood pressure value in mmHg
**sbp_avd_p60_10s_2**		text	Systolic blood pressure value in mmHg

**sbp_avd_p60_10s_3**		text	Systolic blood pressure value in mmHg
**sbp_avd_p60_10s_4**		text	Systolic blood pressure value in mmHg
**sbp_avd_p60_10s_5**		text	Systolic blood pressure value in mmHg
**sbp_avd_p60_10s_6**		text	Systolic blood pressure value in mmHg
**sbp_avd_p60_10s_7**		text	Systolic blood pressure value in mmHg
**sbp_avd_p60_10s_8**		text	Systolic blood pressure value in mmHg
**sbp_avd_p60_10s_9**		text	Systolic blood pressure value in mmHg
**sbp_avd_p60_10s_10**		text	Systolic blood pressure value in mmHg
**sbp_avd_p60_10s_11**		text	Systolic blood pressure value in mmHg
**sbp_avd_p60_10s_12**		text	Systolic blood pressure value in mmHg
**sbp_avd_p60_10s_13**		text	Systolic blood pressure value in mmHg
**sbp_avd_p60_10s_14**		text	Systolic blood pressure value in mmHg
**sbp_avd_p60_10s_15**		text	Systolic blood pressure value in mmHg
**sbp_avd_p60_10s_16**		text	Systolic blood pressure value in mmHg
**sbp_avd_p60_10s_17**		text	Systolic blood pressure value in mmHg
**sbp_avd_p60_10s_18**		text	Systolic blood pressure value in mmHg
**sbp_avd_p60_10s_19**		text	Systolic blood pressure value in mmHg

**sbp_avd_p60_10s_20**		text	Systolic blood pressure value in mmHg
**sbp_avd_p60_10s_21**		text	Systolic blood pressure value in mmHg
**sbp_avd_p60_10s_22**		text	Systolic blood pressure value in mmHg
**sbp_avd_p60_10s_23**		text	Systolic blood pressure value in mmHg
**sbp_avd_p60_10s_24**		text	Systolic blood pressure value in mmHg
**sbp_avd_p60_10s_25**		text	Systolic blood pressure value in mmHg
**sbp_avd_p60_10s_26**		text	Systolic blood pressure value in mmHg
**sbp_avd_p60_10s_27**		text	Systolic blood pressure value in mmHg
**sbp_avd_p60_10s_28**		text	Systolic blood pressure value in mmHg
**sbp_avd_p60_10s_29**		text	Systolic blood pressure value in mmHg
**sbp_avd_p60_10s_30**		text	Systolic blood pressure value in mmHg
**dbp_avd_p60_10s_1**		text	Diastolic blood pressure valuein mmHg
**dbp_avd_p60_10s_2**		text	Diastolic blood pressure valuein mmHg
**dbp_avd_p60_10s_3**		text	Diastolic blood pressure valuein mmHg
**dbp_avd_p60_10s_4**		text	Diastolic blood pressure valuein mmHg
**dbp_avd_p60_10s_5**		text	Diastolic blood pressure valuein mmHg
**dbp_avd_p60_10s_6**		text	Diastolic blood pressure valuein mmHg

**dbp_avd_p60_10s_7**		text	Diastolic blood pressure valuein mmHg
**dbp_avd_p60_10s_8**		text	Diastolic blood pressure valuein mmHg
**dbp_avd_p60_10s_9**		text	Diastolic blood pressure valuein mmHg
**dbp_avd_p60_10s_10**		text	Diastolic blood pressure valuein mmHg
**dbp_avd_p60_10s_11**		text	Diastolic blood pressure valuein mmHg
**dbp_avd_p60_10s_12**		text	Diastolic blood pressure valuein mmHg
**dbp_avd_p60_10s_13**		text	Diastolic blood pressure valuein mmHg
**dbp_avd_p60_10s_14**		text	Diastolic blood pressure valuein mmHg
**dbp_avd_p60_10s_15**		text	Diastolic blood pressure valuein mmHg
**dbp_avd_p60_10s_16**		text	Diastolic blood pressure valuein mmHg
**dbp_avd_p60_10s_17**		text	Diastolic blood pressure valuein mmHg
**dbp_avd_p60_10s_18**		text	Diastolic blood pressure valuein mmHg
**dbp_avd_p60_10s_19**		text	Diastolic blood pressure valuein mmHg
**dbp_avd_p60_10s_20**		text	Diastolic blood pressure valuein mmHg
**dbp_avd_p60_10s_21**		text	Diastolic blood pressure valuein mmHg
**dbp_avd_p60_10s_22**		text	Diastolic blood pressure valuein mmHg
**dbp_avd_p60_10s_23**		text	Diastolic blood pressure valuein mmHg

**dbp_avd_p60_10s_24**		text	Diastolic blood pressure valuein mmHg
**dbp_avd_p60_10s_25**		text	Diastolic blood pressure valuein mmHg
**dbp_avd_p60_10s_26**		text	Diastolic blood pressure valuein mmHg
**dbp_avd_p60_10s_27**		text	Diastolic blood pressure valuein mmHg
**dbp_avd_p60_10s_28**		text	Diastolic blood pressure valuein mmHg
**dbp_avd_p60_10s_29**		text	Diastolic blood pressure valuein mmHg
**dbp_avd_p60_10s_30**		text	Diastolic blood pressure valuein mmHg
**hr_avg_p90_10s_1**	10 second epoch--Post90	text	Average Heart Rate (bpm)
**hr_avg_p90_10s_2**		text	Average Heart Rate (bpm)
**hr_avg_p90_10s_3**		text	Average Heart Rate (bpm)
**hr_avg_p90_10s_4**		text	Average Heart Rate (bpm)
**hr_avg_p90_10s_5**		text	Average Heart Rate (bpm)
**hr_avg_p90_10s_6**		text	Average Heart Rate (bpm)
**hr_avg_p90_10s_7**		text	Average Heart Rate (bpm)
**hr_avg_p90_10s_8**		text	Average Heart Rate (bpm)
**hr_avg_p90_10s_9**		text	Average Heart Rate (bpm)

**hr_avg_p90_10s_10**		text	Average Heart Rate (bpm)
**hr_avg_p90_10s_11**		text	Average Heart Rate (bpm)
**hr_avg_p90_10s_12**		text	Average Heart Rate (bpm)
**hr_avg_p90_10s_13**		text	Average Heart Rate (bpm)
**hr_avg_p90_10s_14**		text	Average Heart Rate (bpm)
**hr_avg_p90_10s_15**		text	Average Heart Rate (bpm)
**hr_avg_p90_10s_16**		text	Average Heart Rate (bpm)
**hr_avg_p90_10s_17**		text	Average Heart Rate (bpm)
**hr_avg_p90_10s_18**		text	Average Heart Rate (bpm)
**hr_avg_p90_10s_19**		text	Average Heart Rate (bpm)
**hr_avg_p90_10s_20**		text	Average Heart Rate (bpm)
**hr_avg_p90_10s_21**		text	Average Heart Rate (bpm)
**hr_avg_p90_10s_22**		text	Average Heart Rate (bpm)
**hr_avg_p90_10s_23**		text	Average Heart Rate (bpm)
**hr_avg_p90_10s_24**		text	Average Heart Rate (bpm)
**hr_avg_p90_10s_25**		text	Average Heart Rate (bpm)
**hr_avg_p90_10s_26**		text	Average Heart Rate (bpm)

**hr_avg_p90_10s_27**		text	Average Heart Rate (bpm)
**hr_avg_p90_10s_28**		text	Average Heart Rate (bpm)
**hr_avg_p90_10s_29**		text	Average Heart Rate (bpm)
**hr_avg_p90_10s_30**		text	Average Heart Rate (bpm)
**sbp_avd_p90_10s_1**		text	Systolic blood pressure value in mmHg
**sbp_avd_p90_10s_2**		text	Systolic blood pressure value in mmHg
**sbp_avd_p90_10s_3**		text	Systolic blood pressure value in mmHg
**sbp_avd_p90_10s_4**		text	Systolic blood pressure value in mmHg
**sbp_avd_p90_10s_5**		text	Systolic blood pressure value in mmHg
**sbp_avd_p90_10s_6**		text	Systolic blood pressure value in mmHg
**sbp_avd_p90_10s_7**		text	Systolic blood pressure value in mmHg
**sbp_avd_p90_10s_8**		text	Systolic blood pressure value in mmHg
**sbp_avd_p90_10s_9**		text	Systolic blood pressure value in mmHg
**sbp_avd_p90_10s_10**		text	Systolic blood pressure value in mmHg
**sbp_avd_p90_10s_11**		text	Systolic blood pressure value in mmHg
**sbp_avd_p90_10s_12**		text	Systolic blood pressure value in mmHg
**sbp_avd_p90_10s_13**		text	Systolic blood pressure value in mmHg

**sbp_avd_p90_10s_14**		text	Systolic blood pressure value in mmHg
**sbp_avd_p90_10s_15**		text	Systolic blood pressure value in mmHg
**sbp_avd_p90_10s_16**		text	Systolic blood pressure value in mmHg
**sbp_avd_p90_10s_17**		text	Systolic blood pressure value in mmHg
**sbp_avd_p90_10s_18**		text	Systolic blood pressure value in mmHg
**sbp_avd_p90_10s_19**		text	Systolic blood pressure value in mmHg
**sbp_avd_p90_10s_20**		text	Systolic blood pressure value in mmHg
**sbp_avd_p90_10s_21**		text	Systolic blood pressure value in mmHg
**sbp_avd_p90_10s_22**		text	Systolic blood pressure value in mmHg
**sbp_avd_p90_10s_23**		text	Systolic blood pressure value in mmHg
**sbp_avd_p90_10s_24**		text	Systolic blood pressure value in mmHg
**sbp_avd_p90_10s_25**		text	Systolic blood pressure value in mmHg
**sbp_avd_p90_10s_26**		text	Systolic blood pressure value in mmHg
**sbp_avd_p90_10s_27**		text	Systolic blood pressure value in mmHg
**sbp_avd_p90_10s_28**		text	Systolic blood pressure value in mmHg
**sbp_avd_p90_10s_29**		text	Systolic blood pressure value in mmHg
**sbp_avd_p90_10s_30**		text	Systolic blood pressure value in mmHg

**dbp_avd_p90_10s_1**		text	Diastolic blood pressure valuein mmHg
**dbp_avd_p90_10s_2**		text	Diastolic blood pressure valuein mmHg
**dbp_avd_p90_10s_3**		text	Diastolic blood pressure valuein mmHg
**dbp_avd_p90_10s_4**		text	Diastolic blood pressure valuein mmHg
**dbp_avd_p90_10s_5**		text	Diastolic blood pressure valuein mmHg
**dbp_avd_p90_10s_6**		text	Diastolic blood pressure valuein mmHg
**dbp_avd_p90_10s_7**		text	Diastolic blood pressure valuein mmHg
**dbp_avd_p90_10s_8**		text	Diastolic blood pressure valuein mmHg
**dbp_avd_p90_10s_9**		text	Diastolic blood pressure valuein mmHg
**dbp_avd_p90_10s_10**		text	Diastolic blood pressure valuein mmHg
**dbp_avd_p90_10s_11**		text	Diastolic blood pressure valuein mmHg
**dbp_avd_p90_10s_12**		text	Diastolic blood pressure valuein mmHg
**dbp_avd_p90_10s_13**		text	Diastolic blood pressure valuein mmHg
**dbp_avd_p90_10s_14**		text	Diastolic blood pressure valuein mmHg
**dbp_avd_p90_10s_15**		text	Diastolic blood pressure valuein mmHg
**dbp_avd_p90_10s_16**		text	Diastolic blood pressure valuein mmHg
**dbp_avd_p90_10s_17**		text	Diastolic blood pressure valuein mmHg

**dbp_avd_p90_10s_18**		text	Diastolic blood pressure valuein mmHg
**dbp_avd_p90_10s_19**		text	Diastolic blood pressure valuein mmHg
**dbp_avd_p90_10s_20**		text	Diastolic blood pressure valuein mmHg
**dbp_avd_p90_10s_21**		text	Diastolic blood pressure valuein mmHg
**dbp_avd_p90_10s_22**		text	Diastolic blood pressure valuein mmHg
**dbp_avd_p90_10s_23**		text	Diastolic blood pressure valuein mmHg
**dbp_avd_p90_10s_24**		text	Diastolic blood pressure valuein mmHg
**dbp_avd_p90_10s_25**		text	Diastolic blood pressure valuein mmHg
**dbp_avd_p90_10s_26**		text	Diastolic blood pressure valuein mmHg
**dbp_avd_p90_10s_27**		text	Diastolic blood pressure valuein mmHg
**dbp_avd_p90_10s_28**		text	Diastolic blood pressure valuein mmHg
**dbp_avd_p90_10s_29**		text	Diastolic blood pressure valuein mmHg
**dbp_avd_p90_10s_30**		text	Diastolic blood pressure valuein mmHg
**hr_avg_p120_10s_1**	10 second epoch--Post120	text	Average Heart Rate (bpm)
**hr_avg_p120_10s_2**		text	Average Heart Rate (bpm)
**hr_avg_p120_10s_3**		text	Average Heart Rate (bpm)

**hr_avg_p120_10s_4**		text	Average Heart Rate (bpm)
**hr_avg_p120_10s_5**		text	Average Heart Rate (bpm)
**hr_avg_p120_10s_6**		text	Average Heart Rate (bpm)
**hr_avg_p120_10s_7**		text	Average Heart Rate (bpm)
**hr_avg_p120_10s_8**		text	Average Heart Rate (bpm)
**hr_avg_p120_10s_9**		text	Average Heart Rate (bpm)
**hr_avg_p120_10s_10**		text	Average Heart Rate (bpm)
**hr_avg_p120_10s_11**		text	Average Heart Rate (bpm)
**hr_avg_p120_10s_12**		text	Average Heart Rate (bpm)
**hr_avg_p120_10s_13**		text	Average Heart Rate (bpm)
**hr_avg_p120_10s_14**		text	Average Heart Rate (bpm)
**hr_avg_p120_10s_15**		text	Average Heart Rate (bpm)
**hr_avg_p120_10s_16**		text	Average Heart Rate (bpm)
**hr_avg_p120_10s_17**		text	Average Heart Rate (bpm)
**hr_avg_p120_10s_18**		text	Average Heart Rate (bpm)
**hr_avg_p120_10s_19**		text	Average Heart Rate (bpm)
**hr_avg_p120_10s_20**		text	Average Heart Rate (bpm)

**hr_avg_p120_10s_21**		text	Average Heart Rate (bpm)
**hr_avg_p120_10s_22**		text	Average Heart Rate (bpm)
**hr_avg_p120_10s_23**		text	Average Heart Rate (bpm)
**hr_avg_p120_10s_24**		text	Average Heart Rate (bpm)
**hr_avg_p120_10s_25**		text	Average Heart Rate (bpm)
**hr_avg_p120_10s_26**		text	Average Heart Rate (bpm)
**hr_avg_p120_10s_27**		text	Average Heart Rate (bpm)
**hr_avg_p120_10s_28**		text	Average Heart Rate (bpm)
**hr_avg_p120_10s_29**		text	Average Heart Rate (bpm)
**hr_avg_p120_10s_30**		text	Average Heart Rate (bpm)
**sbp_avd_p120_10s_1**		text	Systolic blood pressure value in mmHg
**sbp_avd_p120_10s_2**		text	Systolic blood pressure value in mmHg
**sbp_avd_p120_10s_3**		text	Systolic blood pressure value in mmHg
**sbp_avd_p120_10s_4**		text	Systolic blood pressure value in mmHg
**sbp_avd_p120_10s_5**		text	Systolic blood pressure value in mmHg
**sbp_avd_p120_10s_6**		text	Systolic blood pressure value in mmHg
**sbp_avd_p120_10s_7**		text	Systolic blood pressure value in mmHg

**sbp_avd_p120_10s_8**		text	Systolic blood pressure value in mmHg
**sbp_avd_p120_10s_9**		text	Systolic blood pressure value in mmHg
**sbp_avd_p120_10s_10**		text	Systolic blood pressure value in mmHg
**sbp_avd_p120_10s_11**		text	Systolic blood pressure value in mmHg
**sbp_avd_p120_10s_12**		text	Systolic blood pressure value in mmHg
**sbp_avd_p120_10s_13**		text	Systolic blood pressure value in mmHg
**sbp_avd_p120_10s_14**		text	Systolic blood pressure value in mmHg
**sbp_avd_p120_10s_15**		text	Systolic blood pressure value in mmHg
**sbp_avd_p120_10s_16**		text	Systolic blood pressure value in mmHg
**sbp_avd_p120_10s_17**		text	Systolic blood pressure value in mmHg
**sbp_avd_p120_10s_18**		text	Systolic blood pressure value in mmHg
**sbp_avd_p120_10s_19**		text	Systolic blood pressure value in mmHg
**sbp_avd_p120_10s_20**		text	Systolic blood pressure value in mmHg
**sbp_avd_p120_10s_21**		text	Systolic blood pressure value in mmHg
**sbp_avd_p120_10s_22**		text	Systolic blood pressure value in mmHg
**sbp_avd_p120_10s_23**		text	Systolic blood pressure value in mmHg
**sbp_avd_p120_10s_24**		text	Systolic blood pressure value in mmHg

**sbp_avd_p120_10s_25**		text	Systolic blood pressure value in mmHg
**sbp_avd_p120_10s_26**		text	Systolic blood pressure value in mmHg
**sbp_avd_p120_10s_27**		text	Systolic blood pressure value in mmHg
**sbp_avd_p120_10s_28**		text	Systolic blood pressure value in mmHg
**sbp_avd_p120_10s_29**		text	Systolic blood pressure value in mmHg
**sbp_avd_p120_10s_30**		text	Systolic blood pressure value in mmHg
**dbp_avd_p120_10s_1**		text	Diastolic blood pressure valuein mmHg
**dbp_avd_p120_10s_2**		text	Diastolic blood pressure valuein mmHg
**dbp_avd_p120_10s_3**		text	Diastolic blood pressure valuein mmHg
**dbp_avd_p120_10s_4**		text	Diastolic blood pressure valuein mmHg
**dbp_avd_p120_10s_5**		text	Diastolic blood pressure valuein mmHg
**dbp_avd_p120_10s_6**		text	Diastolic blood pressure valuein mmHg
**dbp_avd_p120_10s_7**		text	Diastolic blood pressure valuein mmHg
**dbp_avd_p120_10s_8**		text	Diastolic blood pressure valuein mmHg
**dbp_avd_p120_10s_9**		text	Diastolic blood pressure valuein mmHg
**dbp_avd_p120_10s_10**		text	Diastolic blood pressure valuein mmHg
**dbp_avd_p120_10s_11**		text	Diastolic blood pressure valuein mmHg

**dbp_avd_p120_10s_12**		text	Diastolic blood pressure valuein mmHg
**dbp_avd_p120_10s_13**		text	Diastolic blood pressure valuein mmHg
**dbp_avd_p120_10s_14**		text	Diastolic blood pressure valuein mmHg
**dbp_avd_p120_10s_15**		text	Diastolic blood pressure valuein mmHg
**dbp_avd_p120_10s_16**		text	Diastolic blood pressure valuein mmHg
**dbp_avd_p120_10s_17**		text	Diastolic blood pressure valuein mmHg
**dbp_avd_p120_10s_18**		text	Diastolic blood pressure valuein mmHg
**dbp_avd_p120_10s_19**		text	Diastolic blood pressure valuein mmHg
**dbp_avd_p120_10s_20**		text	Diastolic blood pressure valuein mmHg
**dbp_avd_p120_10s_21**		text	Diastolic blood pressure valuein mmHg
**dbp_avd_p120_10s_22**		text	Diastolic blood pressure valuein mmHg
**dbp_avd_p120_10s_23**		text	Diastolic blood pressure valuein mmHg
**dbp_avd_p120_10s_24**		text	Diastolic blood pressure valuein mmHg
**dbp_avd_p120_10s_25**		text	Diastolic blood pressure valuein mmHg
**dbp_avd_p120_10s_26**		text	Diastolic blood pressure valuein mmHg
**dbp_avd_p120_10s_27**		text	Diastolic blood pressure valuein mmHg
**dbp_avd_p120_10s_28**		text	Diastolic blood pressure valuein mmHg

**dbp_avd_p120_10s_29**		text	Diastolic blood pressure valuein mmHg
**dbp_avd_p120_10s_30**		text	Diastolic blood pressure valuein mmHg
**hr_avg_mr_10s_1**	10 second epoch--MR	text	Average Heart Rate (bpm)
**hr_avg_mr_10s_2**		text	Average Heart Rate (bpm)
**hr_avg_mr_10s_3**		text	Average Heart Rate (bpm)
**hr_avg_mr_10s_4**		text	Average Heart Rate (bpm)
**hr_avg_mr_10s_5**		text	Average Heart Rate (bpm)
**hr_avg_mr_10s_6**		text	Average Heart Rate (bpm)
**hr_avg_mr_10s_7**		text	Average Heart Rate (bpm)
**hr_avg_mr_10s_8**		text	Average Heart Rate (bpm)
**hr_avg_mr_10s_9**		text	Average Heart Rate (bpm)
**hr_avg_mr_10s_10**		text	Average Heart Rate (bpm)
**hr_avg_mr_10s_11**		text	Average Heart Rate (bpm)
**hr_avg_mr_10s_12**		text	Average Heart Rate (bpm)
**hr_avg_mr_10s_13**		text	Average Heart Rate (bpm)
**hr_avg_mr_10s_14**		text	Average Heart Rate (bpm)
**hr_avg_mr_10s_15**		text	Average Heart Rate (bpm)

**hr_avg_mr_10s_16**		text	Average Heart Rate (bpm)
**hr_avg_mr_10s_17**		text	Average Heart Rate (bpm)
**hr_avg_mr_10s_18**		text	Average Heart Rate (bpm)
**hr_avg_mr_10s_19**		text	Average Heart Rate (bpm)
**hr_avg_mr_10s_20**		text	Average Heart Rate (bpm)
**hr_avg_mr_10s_21**		text	Average Heart Rate (bpm)
**hr_avg_mr_10s_22**		text	Average Heart Rate (bpm)
**hr_avg_mr_10s_23**		text	Average Heart Rate (bpm)
**hr_avg_mr_10s_24**		text	Average Heart Rate (bpm)
**hr_avg_mr_10s_25**		text	Average Heart Rate (bpm)
**hr_avg_mr_10s_26**		text	Average Heart Rate (bpm)
**hr_avg_mr_10s_27**		text	Average Heart Rate (bpm)
**hr_avg_mr_10s_28**		text	Average Heart Rate (bpm)
**hr_avg_mr_10s_29**		text	Average Heart Rate (bpm)
**hr_avg_mr_10s_30**		text	Average Heart Rate (bpm)
**sbp_avd_mr_10s_1**		text	Systolic blood pressure value in mmHg
**sbp_avd_mr_10s_2**		text	Systolic blood pressure value in mmHg

**sbp_avd_mr_10s_3**		text	Systolic blood pressure value in mmHg
**sbp_avd_mr_10s_4**		text	Systolic blood pressure value in mmHg
**sbp_avd_mr_10s_5**		text	Systolic blood pressure value in mmHg
**sbp_avd_mr_10s_6**		text	Systolic blood pressure value in mmHg
**sbp_avd_mr_10s_7**		text	Systolic blood pressure value in mmHg
**sbp_avd_mr_10s_8**		text	Systolic blood pressure value in mmHg
**sbp_avd_mr_10s_9**		text	Systolic blood pressure value in mmHg
**sbp_avd_mr_10s_10**		text	Systolic blood pressure value in mmHg
**sbp_avd_mr_10s_11**		text	Systolic blood pressure value in mmHg
**sbp_avd_mr_10s_12**		text	Systolic blood pressure value in mmHg
**sbp_avd_mr_10s_13**		text	Systolic blood pressure value in mmHg
**sbp_avd_mr_10s_14**		text	Systolic blood pressure value in mmHg
**sbp_avd_mr_10s_15**		text	Systolic blood pressure value in mmHg
**sbp_avd_mr_10s_16**		text	Systolic blood pressure value in mmHg
**sbp_avd_mr_10s_17**		text	Systolic blood pressure value in mmHg
**sbp_avd_mr_10s_18**		text	Systolic blood pressure value in mmHg
**sbp_avd_mr_10s_19**		text	Systolic blood pressure value in mmHg

**sbp_avd_mr_10s_20**		text	Systolic blood pressure value in mmHg
**sbp_avd_mr_10s_21**		text	Systolic blood pressure value in mmHg
**sbp_avd_mr_10s_22**		text	Systolic blood pressure value in mmHg
**sbp_avd_mr_10s_23**		text	Systolic blood pressure value in mmHg
**sbp_avd_mr_10s_24**		text	Systolic blood pressure value in mmHg
**sbp_avd_mr_10s_25**		text	Systolic blood pressure value in mmHg
**sbp_avd_mr_10s_26**		text	Systolic blood pressure value in mmHg
**sbp_avd_mr_10s_27**		text	Systolic blood pressure value in mmHg
**sbp_avd_mr_10s_28**		text	Systolic blood pressure value in mmHg
**sbp_avd_mr_10s_29**		text	Systolic blood pressure value in mmHg
**sbp_avd_mr_10s_30**		text	Systolic blood pressure value in mmHg
**dbp_avd_mr_10s_1**		text	Diastolic blood pressure valuein mmHg
**dbp_avd_mr_10s_2**		text	Diastolic blood pressure valuein mmHg
**dbp_avd_mr_10s_3**		text	Diastolic blood pressure valuein mmHg
**dbp_avd_mr_10s_4**		text	Diastolic blood pressure valuein mmHg
**dbp_avd_mr_10s_5**		text	Diastolic blood pressure valuein mmHg
**dbp_avd_mr_10s_6**		text	Diastolic blood pressure valuein mmHg

**dbp_avd_mr_10s_7**		text	Diastolic blood pressure valuein mmHg
**dbp_avd_mr_10s_8**		text	Diastolic blood pressure valuein mmHg
**dbp_avd_mr_10s_9**		text	Diastolic blood pressure valuein mmHg
**dbp_avd_mr_10s_10**		text	Diastolic blood pressure valuein mmHg
**dbp_avd_mr_10s_11**		text	Diastolic blood pressure valuein mmHg
**dbp_avd_mr_10s_12**		text	Diastolic blood pressure valuein mmHg
**dbp_avd_mr_10s_13**		text	Diastolic blood pressure valuein mmHg
**dbp_avd_mr_10s_14**		text	Diastolic blood pressure valuein mmHg
**dbp_avd_mr_10s_15**		text	Diastolic blood pressure valuein mmHg
**dbp_avd_mr_10s_16**		text	Diastolic blood pressure valuein mmHg
**dbp_avd_mr_10s_17**		text	Diastolic blood pressure valuein mmHg
**dbp_avd_mr_10s_18**		text	Diastolic blood pressure valuein mmHg
**dbp_avd_mr_10s_19**		text	Diastolic blood pressure valuein mmHg
**dbp_avd_mr_10s_20**		text	Diastolic blood pressure valuein mmHg
**dbp_avd_mr_10s_21**		text	Diastolic blood pressure valuein mmHg
**dbp_avd_mr_10s_22**		text	Diastolic blood pressure valuein mmHg
**dbp_avd_mr_10s_23**		text	Diastolic blood pressure valuein mmHg

**dbp_avd_mr_10s_24**		text	Diastolic blood pressure valuein mmHg
**dbp_avd_mr_10s_25**		text	Diastolic blood pressure valuein mmHg
**dbp_avd_mr_10s_26**		text	Diastolic blood pressure valuein mmHg
**dbp_avd_mr_10s_27**		text	Diastolic blood pressure valuein mmHg
**dbp_avd_mr_10s_28**		text	Diastolic blood pressure valuein mmHg
**dbp_avd_mr_10s_29**		text	Diastolic blood pressure valuein mmHg
**dbp_avd_mr_10s_30**		text	Diastolic blood pressure valuein mmHg
**hr_avg_dbt_10s_1**		text	Average Heart Rate (bpm)
**hr_avg_dbt_10s_2**		text	Average Heart Rate (bpm)
**hr_avg_dbt_10s_3**		text	Average Heart Rate (bpm)
**hr_avg_dbt_10s_4**		text	Average Heart Rate (bpm)
**hr_avg_dbt_10s_5**		text	Average Heart Rate (bpm)
**hr_avg_dbt_10s_6**		text	Average Heart Rate (bpm)
**hr_avg_dbt_10s_7**		text	Average Heart Rate (bpm)
**hr_avg_dbt_10s_8**		text	Average Heart Rate (bpm)
**hr_avg_dbt_10s_9**		text	Average Heart Rate (bpm)
**hr_avg_dbt_10s_10**		text	Average Heart Rate (bpm)

**hr_avg_dbt_10s_11**		text	Average Heart Rate (bpm)
**hr_avg_dbt_10s_12**		text	Average Heart Rate (bpm)
**hr_avg_dbt_10s_13**		text	Average Heart Rate (bpm)
**hr_avg_dbt_10s_14**		text	Average Heart Rate (bpm)
**hr_avg_dbt_10s_15**		text	Average Heart Rate (bpm)
**hr_avg_dbt_10s_16**		text	Average Heart Rate (bpm)
**hr_avg_dbt_10s_17**		text	Average Heart Rate (bpm)
**hr_avg_dbt_10s_18**		text	Average Heart Rate (bpm)
**hr_avg_dbt_10s_19**		text	Average Heart Rate (bpm)
**hr_avg_dbt_10s_20**		text	Average Heart Rate (bpm)
**hr_avg_dbt_10s_21**		text	Average Heart Rate (bpm)
**hr_avg_dbt_10s_22**		text	Average Heart Rate (bpm)
**hr_avg_dbt_10s_23**		text	Average Heart Rate (bpm)
**hr_avg_dbt_10s_24**		text	Average Heart Rate (bpm)
**hr_avg_dbt_10s_25**		text	Average Heart Rate (bpm)
**hr_avg_dbt_10s_26**		text	Average Heart Rate (bpm)
**hr_avg_dbt_10s_27**		text	Average Heart Rate (bpm)

**hr_avg_dbt_10s_28**		text	Average Heart Rate (bpm)
**hr_avg_dbt_10s_29**		text	Average Heart Rate (bpm)
**hr_avg_dbt_10s_30**		text	Average Heart Rate (bpm)
**sbp_avd_dbt_10s_1**	10 second epoch--DBT	text	Systolic blood pressure value in mmHg
**sbp_avd_dbt_10s_2**		text	Systolic blood pressure value in mmHg
**sbp_avd_dbt_10s_3**		text	Systolic blood pressure value in mmHg
**sbp_avd_dbt_10s_4**		text	Systolic blood pressure value in mmHg
**sbp_avd_dbt_10s_5**		text	Systolic blood pressure value in mmHg
**sbp_avd_dbt_10s_6**		text	Systolic blood pressure value in mmHg
**sbp_avd_dbt_10s_7**		text	Systolic blood pressure value in mmHg
**sbp_avd_dbt_10s_8**		text	Systolic blood pressure value in mmHg
**sbp_avd_dbt_10s_9**		text	Systolic blood pressure value in mmHg
**sbp_avd_dbt_10s_10**		text	Systolic blood pressure value in mmHg
**sbp_avd_dbt_10s_11**		text	Systolic blood pressure value in mmHg
**sbp_avd_dbt_10s_12**		text	Systolic blood pressure value in mmHg
**sbp_avd_dbt_10s_13**		text	Systolic blood pressure value in mmHg
**sbp_avd_dbt_10s_14**		text	Systolic blood pressure value in mmHg

**sbp_avd_dbt_10s_15**		text	Systolic blood pressure value in mmHg
**sbp_avd_dbt_10s_16**		text	Systolic blood pressure value in mmHg
**sbp_avd_dbt_10s_17**		text	Systolic blood pressure value in mmHg
**sbp_avd_dbt_10s_18**		text	Systolic blood pressure value in mmHg
**sbp_avd_dbt_10s_19**		text	Systolic blood pressure value in mmHg
**sbp_avd_dbt_10s_20**		text	Systolic blood pressure value in mmHg
**sbp_avd_dbt_10s_21**		text	Systolic blood pressure value in mmHg
**sbp_avd_dbt_10s_22**		text	Systolic blood pressure value in mmHg
**sbp_avd_dbt_10s_23**		text	Systolic blood pressure value in mmHg
**sbp_avd_dbt_10s_24**		text	Systolic blood pressure value in mmHg
**sbp_avd_dbt_10s_25**		text	Systolic blood pressure value in mmHg
**sbp_avd_dbt_10s_26**		text	Systolic blood pressure value in mmHg
**sbp_avd_dbt_10s_27**		text	Systolic blood pressure value in mmHg
**sbp_avd_dbt_10s_28**		text	Systolic blood pressure value in mmHg
**sbp_avd_dbt_10s_29**		text	Systolic blood pressure value in mmHg
**sbp_avd_dbt_10s_30**		text	Systolic blood pressure value in mmHg
**dbp_avd_dbt_10s_1**		text	Diastolic blood pressure valuein mmHg

**dbp_avd_dbt_10s_2**		text	Diastolic blood pressure valuein mmHg
**dbp_avd_dbt_10s_3**		text	Diastolic blood pressure valuein mmHg
**dbp_avd_dbt_10s_4**		text	Diastolic blood pressure valuein mmHg
**dbp_avd_dbt_10s_5**		text	Diastolic blood pressure valuein mmHg
**dbp_avd_dbt_10s_6**		text	Diastolic blood pressure valuein mmHg
**dbp_avd_dbt_10s_7**		text	Diastolic blood pressure valuein mmHg
**dbp_avd_dbt_10s_8**		text	Diastolic blood pressure valuein mmHg
**dbp_avd_dbt_10s_9**		text	Diastolic blood pressure valuein mmHg
**dbp_avd_dbt_10s_10**		text	Diastolic blood pressure valuein mmHg
**dbp_avd_dbt_10s_11**		text	Diastolic blood pressure valuein mmHg
**dbp_avd_dbt_10s_12**		text	Diastolic blood pressure valuein mmHg
**dbp_avd_dbt_10s_13**		text	Diastolic blood pressure valuein mmHg
**dbp_avd_dbt_10s_14**		text	Diastolic blood pressure valuein mmHg
**dbp_avd_dbt_10s_15**		text	Diastolic blood pressure valuein mmHg
**dbp_avd_dbt_10s_16**		text	Diastolic blood pressure valuein mmHg
**dbp_avd_dbt_10s_17**		text	Diastolic blood pressure valuein mmHg
**dbp_avd_dbt_10s_18**		text	Diastolic blood pressure valuein mmHg

**dbp_avd_dbt_10s_19**		text	Diastolic blood pressure valuein mmHg
**dbp_avd_dbt_10s_20**		text	Diastolic blood pressure valuein mmHg
**dbp_avd_dbt_10s_21**		text	Diastolic blood pressure valuein mmHg
**dbp_avd_dbt_10s_22**		text	Diastolic blood pressure valuein mmHg
**dbp_avd_dbt_10s_23**		text	Diastolic blood pressure valuein mmHg
**dbp_avd_dbt_10s_24**		text	Diastolic blood pressure valuein mmHg
**dbp_avd_dbt_10s_25**		text	Diastolic blood pressure valuein mmHg
**dbp_avd_dbt_10s_26**		text	Diastolic blood pressure valuein mmHg
**dbp_avd_dbt_10s_27**		text	Diastolic blood pressure valuein mmHg
**dbp_avd_dbt_10s_28**		text	Diastolic blood pressure valuein mmHg
**dbp_avd_dbt_10s_29**		text	Diastolic blood pressure valuein mmHg
**dbp_avd_dbt_10s_30**		text	Diastolic blood pressure valuein mmHg
**hr_avg_st_10s_1**	10 second epoch--ST	text	Average Heart Rate (bpm)
**hr_avg_st_10s_2**		text	Average Heart Rate (bpm)
**hr_avg_st_10s_3**		text	Average Heart Rate (bpm)
**hr_avg_st_10s_4**		text	Average Heart Rate (bpm)
**hr_avg_st_10s_5**		text	Average Heart Rate (bpm)

**hr_avg_st_10s_6**		text	Average Heart Rate (bpm)
**hr_avg_st_10s_7**		text	Average Heart Rate (bpm)
**hr_avg_st_10s_8**		text	Average Heart Rate (bpm)
**hr_avg_st_10s_9**		text	Average Heart Rate (bpm)
**hr_avg_st_10s_10**		text	Average Heart Rate (bpm)
**hr_avg_st_10s_11**		text	Average Heart Rate (bpm)
**hr_avg_st_10s_12**		text	Average Heart Rate (bpm)
**hr_avg_st_10s_13**		text	Average Heart Rate (bpm)
**hr_avg_st_10s_14**		text	Average Heart Rate (bpm)
**hr_avg_st_10s_15**		text	Average Heart Rate (bpm)
**hr_avg_st_10s_16**		text	Average Heart Rate (bpm)
**hr_avg_st_10s_17**		text	Average Heart Rate (bpm)
**hr_avg_st_10s_18**		text	Average Heart Rate (bpm)
**hr_avg_st_10s_19**		text	Average Heart Rate (bpm)
**hr_avg_st_10s_20**		text	Average Heart Rate (bpm)
**hr_avg_st_10s_21**		text	Average Heart Rate (bpm)
**hr_avg_st_10s_22**		text	Average Heart Rate (bpm)

**hr_avg_st_10s_23**		text	Average Heart Rate (bpm)
**hr_avg_st_10s_24**		text	Average Heart Rate (bpm)
**hr_avg_st_10s_25**		text	Average Heart Rate (bpm)
**hr_avg_st_10s_26**		text	Average Heart Rate (bpm)
**hr_avg_st_10s_27**		text	Average Heart Rate (bpm)
**hr_avg_st_10s_28**		text	Average Heart Rate (bpm)
**hr_avg_st_10s_29**		text	Average Heart Rate (bpm)
**hr_avg_st_10s_30**		text	Average Heart Rate (bpm)
**sbp_avd_st_10s_1**		text	Systolic blood pressure value in mmHg
**sbp_avd_st_10s_2**		text	Systolic blood pressure value in mmHg
**sbp_avd_st_10s_3**		text	Systolic blood pressure value in mmHg
**sbp_avd_st_10s_4**		text	Systolic blood pressure value in mmHg
**sbp_avd_st_10s_5**		text	Systolic blood pressure value in mmHg
**sbp_avd_st_10s_6**		text	Systolic blood pressure value in mmHg
**sbp_avd_st_10s_7**		text	Systolic blood pressure value in mmHg
**sbp_avd_st_10s_8**		text	Systolic blood pressure value in mmHg
**sbp_avd_st_10s_9**		text	Systolic blood pressure value in mmHg

**sbp_avd_st_10s_10**		text	Systolic blood pressure value in mmHg
**sbp_avd_st_10s_11**		text	Systolic blood pressure value in mmHg
**sbp_avd_st_10s_12**		text	Systolic blood pressure value in mmHg
**sbp_avd_st_10s_13**		text	Systolic blood pressure value in mmHg
**sbp_avd_st_10s_14**		text	Systolic blood pressure value in mmHg
**sbp_avd_st_10s_15**		text	Systolic blood pressure value in mmHg
**sbp_avd_st_10s_16**		text	Systolic blood pressure value in mmHg
**sbp_avd_st_10s_17**		text	Systolic blood pressure value in mmHg
**sbp_avd_st_10s_18**		text	Systolic blood pressure value in mmHg
**sbp_avd_st_10s_19**		text	Systolic blood pressure value in mmHg
**sbp_avd_st_10s_20**		text	Systolic blood pressure value in mmHg
**sbp_avd_st_10s_21**		text	Systolic blood pressure value in mmHg
**sbp_avd_st_10s_22**		text	Systolic blood pressure value in mmHg
**sbp_avd_st_10s_23**		text	Systolic blood pressure value in mmHg
**sbp_avd_st_10s_24**		text	Systolic blood pressure value in mmHg
**sbp_avd_st_10s_25**		text	Systolic blood pressure value in mmHg
**sbp_avd_st_10s_26**		text	Systolic blood pressure value in mmHg

**sbp_avd_st_10s_27**		text	Systolic blood pressure value in mmHg
**sbp_avd_st_10s_28**		text	Systolic blood pressure value in mmHg
**sbp_avd_st_10s_29**		text	Systolic blood pressure value in mmHg
**sbp_avd_st_10s_30**		text	Systolic blood pressure value in mmHg
**dbp_avd_st_10s_1**		text	Diastolic blood pressure valuein mmHg
**dbp_avd_st_10s_2**		text	Diastolic blood pressure valuein mmHg
**dbp_avd_st_10s_3**		text	Diastolic blood pressure valuein mmHg
**dbp_avd_st_10s_4**		text	Diastolic blood pressure valuein mmHg
**dbp_avd_st_10s_5**		text	Diastolic blood pressure valuein mmHg
**dbp_avd_st_10s_6**		text	Diastolic blood pressure valuein mmHg
**dbp_avd_st_10s_7**		text	Diastolic blood pressure valuein mmHg
**dbp_avd_st_10s_8**		text	Diastolic blood pressure valuein mmHg
**dbp_avd_st_10s_9**		text	Diastolic blood pressure valuein mmHg
**dbp_avd_st_10s_10**		text	Diastolic blood pressure valuein mmHg
**dbp_avd_st_10s_11**		text	Diastolic blood pressure valuein mmHg
**dbp_avd_st_10s_12**		text	Diastolic blood pressure valuein mmHg
**dbp_avd_st_10s_13**		text	Diastolic blood pressure valuein mmHg

**dbp_avd_st_10s_14**		text	Diastolic blood pressure valuein mmHg
**dbp_avd_st_10s_15**		text	Diastolic blood pressure valuein mmHg
**dbp_avd_st_10s_16**		text	Diastolic blood pressure valuein mmHg
**dbp_avd_st_10s_17**		text	Diastolic blood pressure valuein mmHg
**dbp_avd_st_10s_18**		text	Diastolic blood pressure valuein mmHg
**dbp_avd_st_10s_19**		text	Diastolic blood pressure valuein mmHg
**dbp_avd_st_10s_20**		text	Diastolic blood pressure valuein mmHg
**dbp_avd_st_10s_21**		text	Diastolic blood pressure valuein mmHg
**dbp_avd_st_10s_22**		text	Diastolic blood pressure valuein mmHg
**dbp_avd_st_10s_23**		text	Diastolic blood pressure valuein mmHg
**dbp_avd_st_10s_24**		text	Diastolic blood pressure valuein mmHg
**dbp_avd_st_10s_25**		text	Diastolic blood pressure valuein mmHg
**dbp_avd_st_10s_26**		text	Diastolic blood pressure valuein mmHg
**dbp_avd_st_10s_27**		text	Diastolic blood pressure valuein mmHg
**dbp_avd_st_10s_28**		text	Diastolic blood pressure valuein mmHg
**dbp_avd_st_10s_29**		text	Diastolic blood pressure valuein mmHg
**dbp_avd_st_10s_30**		text	Diastolic blood pressure valuein mmHg

**hr_avg_sst_10s_1**	10 second epoch--SST	text	Average Heart Rate (bpm)
**hr_avg_sst_10s_2**		text	Average Heart Rate (bpm)
**hr_avg_sst_10s_3**		text	Average Heart Rate (bpm)
**hr_avg_sst_10s_4**		text	Average Heart Rate (bpm)
**hr_avg_sst_10s_5**		text	Average Heart Rate (bpm)
**hr_avg_sst_10s_6**		text	Average Heart Rate (bpm)
**hr_avg_sst_10s_7**		text	Average Heart Rate (bpm)
**hr_avg_sst_10s_8**		text	Average Heart Rate (bpm)
**hr_avg_sst_10s_9**		text	Average Heart Rate (bpm)
**hr_avg_sst_10s_10**		text	Average Heart Rate (bpm)
**hr_avg_sst_10s_11**		text	Average Heart Rate (bpm)
**hr_avg_sst_10s_12**		text	Average Heart Rate (bpm)
**hr_avg_sst_10s_13**		text	Average Heart Rate (bpm)
**hr_avg_sst_10s_14**		text	Average Heart Rate (bpm)
**hr_avg_sst_10s_15**		text	Average Heart Rate (bpm)
**hr_avg_sst_10s_16**		text	Average Heart Rate (bpm)
**hr_avg_sst_10s_17**		text	Average Heart Rate (bpm)

**hr_avg_sst_10s_18**		text	Average Heart Rate (bpm)
**hr_avg_sst_10s_19**		text	Average Heart Rate (bpm)
**hr_avg_sst_10s_20**		text	Average Heart Rate (bpm)
**hr_avg_sst_10s_21**		text	Average Heart Rate (bpm)
**hr_avg_sst_10s_22**		text	Average Heart Rate (bpm)
**hr_avg_sst_10s_23**		text	Average Heart Rate (bpm)
**hr_avg_sst_10s_24**		text	Average Heart Rate (bpm)
**hr_avg_sst_10s_25**		text	Average Heart Rate (bpm)
**hr_avg_sst_10s_26**		text	Average Heart Rate (bpm)
**hr_avg_sst_10s_27**		text	Average Heart Rate (bpm)
**hr_avg_sst_10s_28**		text	Average Heart Rate (bpm)
**hr_avg_sst_10s_29**		text	Average Heart Rate (bpm)
**hr_avg_sst_10s_30**		text	Average Heart Rate (bpm)
**sbp_avd_sst_10s_1**		text	Systolic blood pressure value in mmHg
**sbp_avd_sst_10s_2**		text	Systolic blood pressure value in mmHg
**sbp_avd_sst_10s_3**		text	Systolic blood pressure value in mmHg
**sbp_avd_sst_10s_4**		text	Systolic blood pressure value in mmHg

**sbp_avd_sst_10s_5**		text	Systolic blood pressure value in mmHg
**sbp_avd_sst_10s_6**		text	Systolic blood pressure value in mmHg
**sbp_avd_sst_10s_7**		text	Systolic blood pressure value in mmHg
**sbp_avd_sst_10s_8**		text	Systolic blood pressure value in mmHg
**sbp_avd_sst_10s_9**		text	Systolic blood pressure value in mmHg
**sbp_avd_sst_10s_10**		text	Systolic blood pressure value in mmHg
**sbp_avd_sst_10s_11**		text	Systolic blood pressure value in mmHg
**sbp_avd_sst_10s_12**		text	Systolic blood pressure value in mmHg
**sbp_avd_sst_10s_13**		text	Systolic blood pressure value in mmHg
**sbp_avd_sst_10s_14**		text	Systolic blood pressure value in mmHg
**sbp_avd_sst_10s_15**		text	Systolic blood pressure value in mmHg
**sbp_avd_sst_10s_16**		text	Systolic blood pressure value in mmHg
**sbp_avd_sst_10s_17**		text	Systolic blood pressure value in mmHg
**sbp_avd_sst_10s_18**		text	Systolic blood pressure value in mmHg
**sbp_avd_sst_10s_19**		text	Systolic blood pressure value in mmHg
**sbp_avd_sst_10s_20**		text	Systolic blood pressure value in mmHg
**sbp_avd_sst_10s_21**		text	Systolic blood pressure value in mmHg

**sbp_avd_sst_10s_22**		text	Systolic blood pressure value in mmHg
**sbp_avd_sst_10s_23**		text	Systolic blood pressure value in mmHg
**sbp_avd_sst_10s_24**		text	Systolic blood pressure value in mmHg
**sbp_avd_sst_10s_25**		text	Systolic blood pressure value in mmHg
**sbp_avd_sst_10s_26**		text	Systolic blood pressure value in mmHg
**sbp_avd_sst_10s_27**		text	Systolic blood pressure value in mmHg
**sbp_avd_sst_10s_28**		text	Systolic blood pressure value in mmHg
**sbp_avd_sst_10s_29**		text	Systolic blood pressure value in mmHg
**sbp_avd_sst_10s_30**		text	Systolic blood pressure value in mmHg
**dbp_avd_sst_10s_1**		text	Diastolic blood pressure valuein mmHg
**dbp_avd_sst_10s_2**		text	Diastolic blood pressure valuein mmHg
**dbp_avd_sst_10s_3**		text	Diastolic blood pressure valuein mmHg
**dbp_avd_sst_10s_4**		text	Diastolic blood pressure valuein mmHg
**dbp_avd_sst_10s_5**		text	Diastolic blood pressure valuein mmHg
**dbp_avd_sst_10s_6**		text	Diastolic blood pressure valuein mmHg
**dbp_avd_sst_10s_7**		text	Diastolic blood pressure valuein mmHg
**dbp_avd_sst_10s_8**		text	Diastolic blood pressure valuein mmHg

**dbp_avd_sst_10s_9**		text	Diastolic blood pressure valuein mmHg
**dbp_avd_sst_10s_10**		text	Diastolic blood pressure valuein mmHg
**dbp_avd_sst_10s_11**		text	Diastolic blood pressure valuein mmHg
**dbp_avd_sst_10s_12**		text	Diastolic blood pressure valuein mmHg
**dbp_avd_sst_10s_13**		text	Diastolic blood pressure valuein mmHg
**dbp_avd_sst_10s_14**		text	Diastolic blood pressure valuein mmHg
**dbp_avd_sst_10s_15**		text	Diastolic blood pressure valuein mmHg
**dbp_avd_sst_10s_16**		text	Diastolic blood pressure valuein mmHg
**dbp_avd_sst_10s_17**		text	Diastolic blood pressure valuein mmHg
**dbp_avd_sst_10s_18**		text	Diastolic blood pressure valuein mmHg
**dbp_avd_sst_10s_19**		text	Diastolic blood pressure valuein mmHg
**dbp_avd_sst_10s_20**		text	Diastolic blood pressure valuein mmHg
**dbp_avd_sst_10s_21**		text	Diastolic blood pressure valuein mmHg
**dbp_avd_sst_10s_22**		text	Diastolic blood pressure valuein mmHg
**dbp_avd_sst_10s_23**		text	Diastolic blood pressure valuein mmHg
**dbp_avd_sst_10s_24**		text	Diastolic blood pressure valuein mmHg
**dbp_avd_sst_10s_25**		text	Diastolic blood pressure valuein mmHg

**dbp_avd_sst_10s_26**		text	Diastolic blood pressure valuein mmHg
**dbp_avd_sst_10s_27**		text	Diastolic blood pressure valuein mmHg
**dbp_avd_sst_10s_28**		text	Diastolic blood pressure valuein mmHg
**dbp_avd_sst_10s_29**		text	Diastolic blood pressure valuein mmHg
**dbp_avd_sst_10s_30**		text	Diastolic blood pressure valuein mmHg
**hr_avg_cp_10s_1**	10 second epoch--Cp	text	Average Heart Rate (bpm)
**hr_avg_cp_10s_2**		text	Average Heart Rate (bpm)
**hr_avg_cp_10s_3**		text	Average Heart Rate (bpm)
**hr_avg_cp_10s_4**		text	Average Heart Rate (bpm)
**hr_avg_cp_10s_5**		text	Average Heart Rate (bpm)
**hr_avg_cp_10s_6**		text	Average Heart Rate (bpm)
**hr_avg_cp_10s_7**		text	Average Heart Rate (bpm)
**hr_avg_cp_10s_8**		text	Average Heart Rate (bpm)
**hr_avg_cp_10s_9**		text	Average Heart Rate (bpm)
**hr_avg_cp_10s_10**		text	Average Heart Rate (bpm)
**hr_avg_cp_10s_11**		text	Average Heart Rate (bpm)
**hr_avg_cp_10s_12**		text	Average Heart Rate (bpm)

**hr_avg_cp_10s_13**		text	Average Heart Rate (bpm)
**hr_avg_cp_10s_14**		text	Average Heart Rate (bpm)
**hr_avg_cp_10s_15**		text	Average Heart Rate (bpm)
**hr_avg_cp_10s_16**		text	Average Heart Rate (bpm)
**hr_avg_cp_10s_17**		text	Average Heart Rate (bpm)
**hr_avg_cp_10s_18**		text	Average Heart Rate (bpm)
**hr_avg_cp_10s_19**		text	Average Heart Rate (bpm)
**hr_avg_cp_10s_20**		text	Average Heart Rate (bpm)
**hr_avg_cp_10s_21**		text	Average Heart Rate (bpm)
**hr_avg_cp_10s_22**		text	Average Heart Rate (bpm)
**hr_avg_cp_10s_23**		text	Average Heart Rate (bpm)
**hr_avg_cp_10s_24**		text	Average Heart Rate (bpm)
**hr_avg_cp_10s_25**		text	Average Heart Rate (bpm)
**hr_avg_cp_10s_26**		text	Average Heart Rate (bpm)
**hr_avg_cp_10s_27**		text	Average Heart Rate (bpm)
**hr_avg_cp_10s_28**		text	Average Heart Rate (bpm)
**hr_avg_cp_10s_29**		text	Average Heart Rate (bpm)

**hr_avg_cp_10s_30**		text	Average Heart Rate (bpm)
**sbp_avd_cp_10s_1**		text	Systolic blood pressure value in mmHg
**sbp_avd_cp_10s_2**		text	Systolic blood pressure value in mmHg
**sbp_avd_cp_10s_3**		text	Systolic blood pressure value in mmHg
**sbp_avd_cp_10s_4**		text	Systolic blood pressure value in mmHg
**sbp_avd_cp_10s_5**		text	Systolic blood pressure value in mmHg
**sbp_avd_cp_10s_6**		text	Systolic blood pressure value in mmHg
**sbp_avd_cp_10s_7**		text	Systolic blood pressure value in mmHg
**sbp_avd_cp_10s_8**		text	Systolic blood pressure value in mmHg
**sbp_avd_cp_10s_9**		text	Systolic blood pressure value in mmHg
**sbp_avd_cp_10s_10**		text	Systolic blood pressure value in mmHg
**sbp_avd_cp_10s_11**		text	Systolic blood pressure value in mmHg
**sbp_avd_cp_10s_12**		text	Systolic blood pressure value in mmHg
**sbp_avd_cp_10s_13**		text	Systolic blood pressure value in mmHg
**sbp_avd_cp_10s_14**		text	Systolic blood pressure value in mmHg
**sbp_avd_cp_10s_15**		text	Systolic blood pressure value in mmHg
**sbp_avd_cp_10s_16**		text	Systolic blood pressure value in mmHg

**sbp_avd_cp_10s_17**		text	Systolic blood pressure value in mmHg
**sbp_avd_cp_10s_18**		text	Systolic blood pressure value in mmHg
**sbp_avd_cp_10s_19**		text	Systolic blood pressure value in mmHg
**sbp_avd_cp_10s_20**		text	Systolic blood pressure value in mmHg
**sbp_avd_cp_10s_21**		text	Systolic blood pressure value in mmHg
**sbp_avd_cp_10s_22**		text	Systolic blood pressure value in mmHg
**sbp_avd_cp_10s_23**		text	Systolic blood pressure value in mmHg
**sbp_avd_cp_10s_24**		text	Systolic blood pressure value in mmHg
**sbp_avd_cp_10s_25**		text	Systolic blood pressure value in mmHg
**sbp_avd_cp_10s_26**		text	Systolic blood pressure value in mmHg
**sbp_avd_cp_10s_27**		text	Systolic blood pressure value in mmHg
**sbp_avd_cp_10s_28**		text	Systolic blood pressure value in mmHg
**sbp_avd_cp_10s_29**		text	Systolic blood pressure value in mmHg
**sbp_avd_cp_10s_30**		text	Systolic blood pressure value in mmHg
**dbp_avd_cp_10s_1**		text	Diastolic blood pressure valuein mmHg
**dbp_avd_cp_10s_2**		text	Diastolic blood pressure valuein mmHg
**dbp_avd_cp_10s_3**		text	Diastolic blood pressure valuein mmHg

**dbp_avd_cp_10s_4**		text	Diastolic blood pressure valuein mmHg
**dbp_avd_cp_10s_5**		text	Diastolic blood pressure valuein mmHg
**dbp_avd_cp_10s_6**		text	Diastolic blood pressure valuein mmHg
**dbp_avd_cp_10s_7**		text	Diastolic blood pressure valuein mmHg
**dbp_avd_cp_10s_8**		text	Diastolic blood pressure valuein mmHg
**dbp_avd_cp_10s_9**		text	Diastolic blood pressure valuein mmHg
**dbp_avd_cp_10s_10**		text	Diastolic blood pressure valuein mmHg
**dbp_avd_cp_10s_11**		text	Diastolic blood pressure valuein mmHg
**dbp_avd_cp_10s_12**		text	Diastolic blood pressure valuein mmHg
**dbp_avd_cp_10s_13**		text	Diastolic blood pressure valuein mmHg
**dbp_avd_cp_10s_14**		text	Diastolic blood pressure valuein mmHg
**dbp_avd_cp_10s_15**		text	Diastolic blood pressure valuein mmHg
**dbp_avd_cp_10s_16**		text	Diastolic blood pressure valuein mmHg
**dbp_avd_cp_10s_17**		text	Diastolic blood pressure valuein mmHg
**dbp_avd_cp_10s_18**		text	Diastolic blood pressure valuein mmHg
**dbp_avd_cp_10s_19**		text	Diastolic blood pressure valuein mmHg
**dbp_avd_cp_10s_20**		text	Diastolic blood pressure valuein mmHg

**dbp_avd_cp_10s_21**		text	Diastolic blood pressure valuein mmHg
**dbp_avd_cp_10s_22**		text	Diastolic blood pressure valuein mmHg
**dbp_avd_cp_10s_23**		text	Diastolic blood pressure valuein mmHg
**dbp_avd_cp_10s_24**		text	Diastolic blood pressure valuein mmHg
**dbp_avd_cp_10s_25**		text	Diastolic blood pressure valuein mmHg
**dbp_avd_cp_10s_26**		text	Diastolic blood pressure valuein mmHg
**dbp_avd_cp_10s_27**		text	Diastolic blood pressure valuein mmHg
**dbp_avd_cp_10s_28**		text	Diastolic blood pressure valuein mmHg
**dbp_avd_cp_10s_29**		text	Diastolic blood pressure valuein mmHg
**dbp_avd_cp_10s_30**		text	Diastolic blood pressure valuein mmHg
**respr_base_1**	10 seconds respiration	text	Respiratory Rate for task base Block 1
**respr_base_2**		text	Respiratory Rate for task base Block 2
**respr_base_3**		text	Respiratory Rate for task base Block 3
**respr_base_4**		text	Respiratory Rate for task base Block 4
**respr_base_5**		text	Respiratory Rate for task base Block 5
**respr_base_6**		text	Respiratory Rate for task base Block 6
**respr_base_7**		text	Respiratory Rate for task base Block 7

**respr_base_8**		text	Respiratory Rate for task base Block 8
**respr_base_9**		text	Respiratory Rate for task base Block 9
**respr_base_10**		text	Respiratory Rate for task base Block 10
**respr_base_11**		text	Respiratory Rate for task base Block 11
**respr_base_12**		text	Respiratory Rate for task base Block 12
**respr_base_13**		text	Respiratory Rate for task base Block 13
**respr_base_14**		text	Respiratory Rate for task base Block 14
**respr_base_15**		text	Respiratory Rate for task base Block 15
**respr_base_16**		text	Respiratory Rate for task base Block 16
**respr_base_17**		text	Respiratory Rate for task base Block 17
**respr_base_18**		text	Respiratory Rate for task base Block 18
**respr_base_19**		text	Respiratory Rate for task base Block 19
**respr_base_20**		text	Respiratory Rate for task base Block 20
**respr_base_21**		text	Respiratory Rate for task base Block 21
**respr_base_22**		text	Respiratory Rate for task base Block 22
**respr_base_23**		text	Respiratory Rate for task base Block 23
**respr_base_24**		text	Respiratory Rate for task base Block 24

**respr_base_25**		text	Respiratory Rate for task base Block 25
**respr_base_26**		text	Respiratory Rate for task base Block 26
**respr_base_27**		text	Respiratory Rate for task base Block 27
**respr_base_28**		text	Respiratory Rate for task base Block 28
**respr_base_29**		text	Respiratory Rate for task base Block 29
**respr_base_30**		text	Respiratory Rate for task base Block 30
**respr_base_31**		text	Respiratory Rate for task base Block 31
**respr_base_32**		text	Respiratory Rate for task base Block 32
**respr_base_33**		text	Respiratory Rate for task base Block 33
**respr_base_34**		text	Respiratory Rate for task base Block 34
**respr_base_35**		text	Respiratory Rate for task base Block 35
**respr_base_36**		text	Respiratory Rate for task base Block 36
**respr_base_37**		text	Respiratory Rate for task base Block 37
**respr_base_38**		text	Respiratory Rate for task base Block 38
**respr_base_39**		text	Respiratory Rate for task base Block 39
**respr_base_40**		text	Respiratory Rate for task base Block 40
**respr_base_41**		text	Respiratory Rate for task base Block 41

**respr_base_42**		text	Respiratory Rate for task base Block 42
**respr_base_43**		text	Respiratory Rate for task base Block 43
**respr_base_44**		text	Respiratory Rate for task base Block 44
**respr_base_45**		text	Respiratory Rate for task base Block 45
**respr_base_48**		text	Respiratory Rate for task base Block 48
**respr_base_49**		text	Respiratory Rate for task base Block 49
**respr_base_50**		text	Respiratory Rate for task base Block 50
**respr_cp_1**		text	Respiratory Rate for task cp Block 1
**respr_cp_2**		text	Respiratory Rate for task cp Block 2
**respr_cp_3**		text	Respiratory Rate for task cp Block 3
**respr_cp_4**		text	Respiratory Rate for task cp Block 4
**respr_cp_5**		text	Respiratory Rate for task cp Block 5
**respr_cp_6**		text	Respiratory Rate for task cp Block 6
**respr_cp_7**		text	Respiratory Rate for task cp Block 7
**respr_cp_8**		text	Respiratory Rate for task cp Block 8
**respr_cp_9**		text	Respiratory Rate for task cp Block 9
**respr_cp_10**		text	Respiratory Rate for task cp Block 10

**respr_cp_11**		text	Respiratory Rate for task cp Block 11
**respr_cp_12**		text	Respiratory Rate for task cp Block 12
**respr_cp_13**		text	Respiratory Rate for task cp Block 13
**respr_cp_14**		text	Respiratory Rate for task cp Block 14
**respr_cp_15**		text	Respiratory Rate for task cp Block 15
**respr_cp_16**		text	Respiratory Rate for task cp Block 16
**respr_cp_17**		text	Respiratory Rate for task cp Block 17
**respr_cp_18**		text	Respiratory Rate for task cp Block 18
**respr_cp_19**		text	Respiratory Rate for task cp Block 19
**respr_cp_20**		text	Respiratory Rate for task cp Block 20
**respr_cp_21**		text	Respiratory Rate for task cp Block 21
**respr_cp_22**		text	Respiratory Rate for task cp Block 22
**respr_cp_23**		text	Respiratory Rate for task cp Block 23
**respr_cp_24**		text	Respiratory Rate for task cp Block 24
**respr_cp_25**		text	Respiratory Rate for task cp Block 25
**respr_cp_26**		text	Respiratory Rate for task cp Block 26
**respr_cp_27**		text	Respiratory Rate for task cp Block 27

**respr_cp_28**		text	Respiratory Rate for task cp Block 28
**respr_cp_29**		text	Respiratory Rate for task cp Block 29
**respr_cp_30**		text	Respiratory Rate for task cp Block 30
**respr_cp_31**		text	Respiratory Rate for task cp Block 31
**respr_cp_32**		text	Respiratory Rate for task cp Block 32
**respr_cp_33**		text	Respiratory Rate for task cp Block 33
**respr_cp_34**		text	Respiratory Rate for task cp Block 34
**respr_cp_35**		text	Respiratory Rate for task cp Block 35
**respr_cp_36**		text	Respiratory Rate for task cp Block 36
**respr_cp_37**		text	Respiratory Rate for task cp Block 37
**respr_cp_38**		text	Respiratory Rate for task cp Block 38
**respr_cp_39**		text	Respiratory Rate for task cp Block 39
**respr_cp_40**		text	Respiratory Rate for task cp Block 40
**respr_cp_41**		text	Respiratory Rate for task cp Block 41
**respr_cp_42**		text	Respiratory Rate for task cp Block 42
**respr_cp_43**		text	Respiratory Rate for task cp Block 43
**respr_cp_44**		text	Respiratory Rate for task cp Block 44

**respr_cp_45**		text	Respiratory Rate for task cp Block 45
**respr_cp_46**		text	Respiratory Rate for task cp Block 46
**respr_cp_47**		text	Respiratory Rate for task cp Block 47
**respr_cp_48**		text	Respiratory Rate for task cp Block 48
**respr_cp_49**		text	Respiratory Rate for task cp Block 49
**respr_cp_50**		text	Respiratory Rate for task cp Block 50
**respr_dbt_1**		text	Respiratory Rate for task dbt Block 1
**respr_dbt_2**		text	Respiratory Rate for task dbt Block 2
**respr_dbt_3**		text	Respiratory Rate for task dbt Block 3
**respr_dbt_4**		text	Respiratory Rate for task dbt Block 4
**respr_dbt_5**		text	Respiratory Rate for task dbt Block 5
**respr_dbt_6**		text	Respiratory Rate for task dbt Block 6
**respr_dbt_7**		text	Respiratory Rate for task dbt Block 7
**respr_dbt_8**		text	Respiratory Rate for task dbt Block 8
**respr_dbt_9**		text	Respiratory Rate for task dbt Block 9
**respr_dbt_10**		text	Respiratory Rate for task dbt Block 10
**respr_dbt_11**		text	Respiratory Rate for task dbt Block 11

**respr_dbt_12**		text	Respiratory Rate for task dbt Block 12
**respr_dbt_13**		text	Respiratory Rate for task dbt Block 13
**respr_dbt_14**		text	Respiratory Rate for task dbt Block 14
**respr_dbt_15**		text	Respiratory Rate for task dbt Block 15
**respr_dbt_16**		text	Respiratory Rate for task dbt Block 16
**respr_dbt_17**		text	Respiratory Rate for task dbt Block 17
**respr_dbt_18**		text	Respiratory Rate for task dbt Block 18
**respr_dbt_19**		text	Respiratory Rate for task dbt Block 19
**respr_dbt_20**		text	Respiratory Rate for task dbt Block 20
**respr_dbt_21**		text	Respiratory Rate for task dbt Block 21
**respr_dbt_22**		text	Respiratory Rate for task dbt Block 22
**respr_dbt_23**		text	Respiratory Rate for task dbt Block 23
**respr_dbt_24**		text	Respiratory Rate for task dbt Block 24
**respr_dbt_25**		text	Respiratory Rate for task dbt Block 25
**respr_dbt_26**		text	Respiratory Rate for task dbt Block 26
**respr_dbt_27**		text	Respiratory Rate for task dbt Block 27
**respr_dbt_28**		text	Respiratory Rate for task dbt Block 28

**respr_dbt_29**		text	Respiratory Rate for task dbt Block 29
**respr_dbt_30**		text	Respiratory Rate for task dbt Block 30
**respr_dbt_31**		text	Respiratory Rate for task dbt Block 31
**respr_dbt_32**		text	Respiratory Rate for task dbt Block 32
**respr_dbt_33**		text	Respiratory Rate for task dbt Block 33
**respr_dbt_34**		text	Respiratory Rate for task dbt Block 34
**respr_dbt_35**		text	Respiratory Rate for task dbt Block 35
**respr_dbt_36**		text	Respiratory Rate for task dbt Block 36
**respr_dbt_37**		text	Respiratory Rate for task dbt Block 37
**respr_dbt_38**		text	Respiratory Rate for task dbt Block 38
**respr_dbt_39**		text	Respiratory Rate for task dbt Block 39
**respr_dbt_40**		text	Respiratory Rate for task dbt Block 40
**respr_dbt_41**		text	Respiratory Rate for task dbt Block 41
**respr_dbt_42**		text	Respiratory Rate for task dbt Block 42
**respr_dbt_43**		text	Respiratory Rate for task dbt Block 43
**respr_dbt_44**		text	Respiratory Rate for task dbt Block 44
**respr_dbt_47**		text	Respiratory Rate for task dbt Block 47

**respr_dbt_50**		text	Respiratory Rate for task dbt Block 50
**respr_instruct_1**		text	Respiratory Rate for task instruct Block 1
**respr_instruct_2**		text	Respiratory Rate for task instruct Block 2
**respr_instruct_3**		text	Respiratory Rate for task instruct Block 3
**respr_instruct_4**		text	Respiratory Rate for task instruct Block 4
**respr_instruct_5**		text	Respiratory Rate for task instruct Block 5
**respr_instruct_6**		text	Respiratory Rate for task instruct Block 6
**respr_instruct_7**		text	Respiratory Rate for task instruct Block 7
**respr_instruct_8**		text	Respiratory Rate for task instruct Block 8
**respr_instruct_9**		text	Respiratory Rate for task instruct Block 9
**respr_instruct_10**		text	Respiratory Rate for task instruct Block 10
**respr_instruct_11**		text	Respiratory Rate for task instruct Block 11
**respr_instruct_12**		text	Respiratory Rate for task instruct Block 12
**respr_instruct_13**		text	Respiratory Rate for task instruct Block 13
**respr_instruct_14**		text	Respiratory Rate for task instruct Block 14
**respr_instruct_15**		text	Respiratory Rate for task instruct Block 15
**respr_instruct_16**		text	Respiratory Rate for task instruct Block 16

**respr_instruct_17**		text	Respiratory Rate for task instruct Block 17
**respr_instruct_18**		text	Respiratory Rate for task instruct Block 18
**respr_instruct_19**		text	Respiratory Rate for task instruct Block 19
**respr_instruct_20**		text	Respiratory Rate for task instruct Block 20
**respr_instruct_21**		text	Respiratory Rate for task instruct Block 21
**respr_instruct_22**		text	Respiratory Rate for task instruct Block 22
**respr_instruct_23**		text	Respiratory Rate for task instruct Block 23
**respr_instruct_24**		text	Respiratory Rate for task instruct Block 24
**respr_instruct_25**		text	Respiratory Rate for task instruct Block 25
**respr_instruct_26**		text	Respiratory Rate for task instruct Block 26
**respr_instruct_27**		text	Respiratory Rate for task instruct Block 27
**respr_instruct_28**		text	Respiratory Rate for task instruct Block 28
**respr_instruct_29**		text	Respiratory Rate for task instruct Block 29
**respr_instruct_30**		text	Respiratory Rate for task instruct Block 30
**respr_instruct_31**		text	Respiratory Rate for task instruct Block 31
**respr_instruct_32**		text	Respiratory Rate for task instruct Block 32
**respr_instruct_33**		text	Respiratory Rate for task instruct Block 33

**respr_instruct_34**		text	Respiratory Rate for task instruct Block 34
**respr_instruct_35**		text	Respiratory Rate for task instruct Block 35
**respr_instruct_36**		text	Respiratory Rate for task instruct Block 36
**respr_instruct_37**		text	Respiratory Rate for task instruct Block 37
**respr_instruct_38**		text	Respiratory Rate for task instruct Block 38
**respr_instruct_39**		text	Respiratory Rate for task instruct Block 39
**respr_instruct_40**		text	Respiratory Rate for task instruct Block 40
**respr_instruct_41**		text	Respiratory Rate for task instruct Block 41
**respr_instruct_42**		text	Respiratory Rate for task instruct Block 42
**respr_instruct_43**		text	Respiratory Rate for task instruct Block 43
**respr_instruct_44**		text	Respiratory Rate for task instruct Block 44
**respr_instruct_45**		text	Respiratory Rate for task instruct Block 45
**respr_instruct_46**		text	Respiratory Rate for task instruct Block 46
**respr_instruct_47**		text	Respiratory Rate for task instruct Block 47
**respr_instruct_48**		text	Respiratory Rate for task instruct Block 48
**respr_instruct_49**		text	Respiratory Rate for task instruct Block 49
**respr_instruct_50**		text	Respiratory Rate for task instruct Block 50

**respr_mr_1**		text	Respiratory Rate for task mr Block 1
**respr_mr_2**		text	Respiratory Rate for task mr Block 2
**respr_mr_3**		text	Respiratory Rate for task mr Block 3
**respr_mr_4**		text	Respiratory Rate for task mr Block 4
**respr_mr_5**		text	Respiratory Rate for task mr Block 5
**respr_mr_6**		text	Respiratory Rate for task mr Block 6
**respr_mr_7**		text	Respiratory Rate for task mr Block 7
**respr_mr_8**		text	Respiratory Rate for task mr Block 8
**respr_mr_9**		text	Respiratory Rate for task mr Block 9
**respr_mr_10**		text	Respiratory Rate for task mr Block 10
**respr_mr_11**		text	Respiratory Rate for task mr Block 11
**respr_mr_12**		text	Respiratory Rate for task mr Block 12
**respr_mr_13**		text	Respiratory Rate for task mr Block 13
**respr_mr_14**		text	Respiratory Rate for task mr Block 14
**respr_mr_15**		text	Respiratory Rate for task mr Block 15
**respr_mr_16**		text	Respiratory Rate for task mr Block 16
**respr_mr_17**		text	Respiratory Rate for task mr Block 17

**respr_mr_18**		text	Respiratory Rate for task mr Block 18
**respr_mr_19**		text	Respiratory Rate for task mr Block 19
**respr_mr_20**		text	Respiratory Rate for task mr Block 20
**respr_mr_21**		text	Respiratory Rate for task mr Block 21
**respr_mr_22**		text	Respiratory Rate for task mr Block 22
**respr_mr_23**		text	Respiratory Rate for task mr Block 23
**respr_mr_24**		text	Respiratory Rate for task mr Block 24
**respr_mr_25**		text	Respiratory Rate for task mr Block 25
**respr_mr_26**		text	Respiratory Rate for task mr Block 26
**respr_mr_27**		text	Respiratory Rate for task mr Block 27
**respr_mr_28**		text	Respiratory Rate for task mr Block 28
**respr_mr_29**		text	Respiratory Rate for task mr Block 29
**respr_mr_30**		text	Respiratory Rate for task mr Block 30
**respr_mr_31**		text	Respiratory Rate for task mr Block 31
**respr_mr_32**		text	Respiratory Rate for task mr Block 32
**respr_mr_33**		text	Respiratory Rate for task mr Block 33
**respr_mr_34**		text	Respiratory Rate for task mr Block 34

**respr_mr_35**		text	Respiratory Rate for task mr Block 35
**respr_mr_36**		text	Respiratory Rate for task mr Block 36
**respr_mr_37**		text	Respiratory Rate for task mr Block 37
**respr_mr_38**		text	Respiratory Rate for task mr Block 38
**respr_mr_39**		text	Respiratory Rate for task mr Block 39
**respr_mr_40**		text	Respiratory Rate for task mr Block 40
**respr_mr_41**		text	Respiratory Rate for task mr Block 41
**respr_mr_42**		text	Respiratory Rate for task mr Block 42
**respr_mr_43**		text	Respiratory Rate for task mr Block 43
**respr_mr_44**		text	Respiratory Rate for task mr Block 44
**respr_mr_45**		text	Respiratory Rate for task mr Block 45
**respr_mr_46**		text	Respiratory Rate for task mr Block 46
**respr_mr_47**		text	Respiratory Rate for task mr Block 47
**respr_mr_48**		text	Respiratory Rate for task mr Block 48
**respr_mr_49**		text	Respiratory Rate for task mr Block 49
**respr_mr_50**		text	Respiratory Rate for task mr Block 50
**respr_post10_1**		text	Respiratory Rate for task post10 Block 1

**respr_post10_2**		text	Respiratory Rate for task post10 Block 2
**respr_post10_3**		text	Respiratory Rate for task post10 Block 3
**respr_post10_4**		text	Respiratory Rate for task post10 Block 4
**respr_post10_5**		text	Respiratory Rate for task post10 Block 5
**respr_post10_6**		text	Respiratory Rate for task post10 Block 6
**respr_post10_7**		text	Respiratory Rate for task post10 Block 7
**respr_post10_8**		text	Respiratory Rate for task post10 Block 8
**respr_post10_9**		text	Respiratory Rate for task post10 Block 9
**respr_post10_10**		text	Respiratory Rate for task post10 Block 10
**respr_post10_11**		text	Respiratory Rate for task post10 Block 11
**respr_post10_12**		text	Respiratory Rate for task post10 Block 12
**respr_post10_13**		text	Respiratory Rate for task post10 Block 13
**respr_post10_14**		text	Respiratory Rate for task post10 Block 14
**respr_post10_15**		text	Respiratory Rate for task post10 Block 15
**respr_post10_16**		text	Respiratory Rate for task post10 Block 16
**respr_post10_17**		text	Respiratory Rate for task post10 Block 17
**respr_post10_18**		text	Respiratory Rate for task post10 Block 18

**respr_post10_19**		text	Respiratory Rate for task post10 Block 19
**respr_post10_20**		text	Respiratory Rate for task post10 Block 20
**respr_post10_21**		text	Respiratory Rate for task post10 Block 21
**respr_post10_22**		text	Respiratory Rate for task post10 Block 22
**respr_post10_23**		text	Respiratory Rate for task post10 Block 23
**respr_post10_24**		text	Respiratory Rate for task post10 Block 24
**respr_post10_25**		text	Respiratory Rate for task post10 Block 25
**respr_post10_26**		text	Respiratory Rate for task post10 Block 26
**respr_post10_27**		text	Respiratory Rate for task post10 Block 27
**respr_post10_28**		text	Respiratory Rate for task post10 Block 28
**respr_post10_29**		text	Respiratory Rate for task post10 Block 29
**respr_post10_30**		text	Respiratory Rate for task post10 Block 30
**respr_post10_31**		text	Respiratory Rate for task post10 Block 31
**respr_post10_32**		text	Respiratory Rate for task post10 Block 32
**respr_post10_33**		text	Respiratory Rate for task post10 Block 33
**respr_post10_34**		text	Respiratory Rate for task post10 Block 34
**respr_post10_35**		text	Respiratory Rate for task post10 Block 35

**respr_post10_36**		text	Respiratory Rate for task post10 Block 36
**respr_post10_37**		text	Respiratory Rate for task post10 Block 37
**respr_post10_38**		text	Respiratory Rate for task post10 Block 38
**respr_post10_39**		text	Respiratory Rate for task post10 Block 39
**respr_post10_40**		text	Respiratory Rate for task post10 Block 40
**respr_post10_41**		text	Respiratory Rate for task post10 Block 41
**respr_post10_42**		text	Respiratory Rate for task post10 Block 42
**respr_post10_43**		text	Respiratory Rate for task post10 Block 43
**respr_post10_46**		text	Respiratory Rate for task post10 Block 46
**respr_post10_47**		text	Respiratory Rate for task post10 Block 47
**respr_post10_48**		text	Respiratory Rate for task post10 Block 48
**respr_post10_49**		text	Respiratory Rate for task post10 Block 49
**respr_post10_50**		text	Respiratory Rate for task post10 Block 50
**respr_post120_1**		text	Respiratory Rate for task post120 Block 1
**respr_post120_2**		text	Respiratory Rate for task post120 Block 2
**respr_post120_3**		text	Respiratory Rate for task post120 Block 3
**respr_post120_4**		text	Respiratory Rate for task post120 Block 4

**respr_post120_5**		text	Respiratory Rate for task post120 Block 5
**respr_post120_6**		text	Respiratory Rate for task post120 Block 6
**respr_post120_7**		text	Respiratory Rate for task post120 Block 7
**respr_post120_8**		text	Respiratory Rate for task post120 Block 8
**respr_post120_9**		text	Respiratory Rate for task post120 Block 9
**respr_post120_10**		text	Respiratory Rate for task post120 Block 10
**respr_post120_11**		text	Respiratory Rate for task post120 Block 11
**respr_post120_12**		text	Respiratory Rate for task post120 Block 12
**respr_post120_13**		text	Respiratory Rate for task post120 Block 13
**respr_post120_14**		text	Respiratory Rate for task post120 Block 14
**respr_post120_15**		text	Respiratory Rate for task post120 Block 15
**respr_post120_16**		text	Respiratory Rate for task post120 Block 16
**respr_post120_17**		text	Respiratory Rate for task post120 Block 17
**respr_post120_18**		text	Respiratory Rate for task post120 Block 18
**respr_post120_19**		text	Respiratory Rate for task post120 Block 19
**respr_post120_20**		text	Respiratory Rate for task post120 Block 20
**respr_post120_21**		text	Respiratory Rate for task post120 Block 21

**respr_post120_22**		text	Respiratory Rate for task post120 Block 22
**respr_post120_23**		text	Respiratory Rate for task post120 Block 23
**respr_post120_24**		text	Respiratory Rate for task post120 Block 24
**respr_post120_25**		text	Respiratory Rate for task post120 Block 25
**respr_post120_26**		text	Respiratory Rate for task post120 Block 26
**respr_post120_27**		text	Respiratory Rate for task post120 Block 27
**respr_post120_28**		text	Respiratory Rate for task post120 Block 28
**respr_post120_29**		text	Respiratory Rate for task post120 Block 29
**respr_post120_30**		text	Respiratory Rate for task post120 Block 30
**respr_post120_31**		text	Respiratory Rate for task post120 Block 31
**respr_post120_32**		text	Respiratory Rate for task post120 Block 32
**respr_post120_33**		text	Respiratory Rate for task post120 Block 33
**respr_post120_34**		text	Respiratory Rate for task post120 Block 34
**respr_post120_35**		text	Respiratory Rate for task post120 Block 35
**respr_post120_36**		text	Respiratory Rate for task post120 Block 36
**respr_post120_37**		text	Respiratory Rate for task post120 Block 37
**respr_post120_38**		text	Respiratory Rate for task post120 Block 38

**respr_post120_39**		text	Respiratory Rate for task post120 Block 39
**respr_post120_40**		text	Respiratory Rate for task post120 Block 40
**respr_post120_41**		text	Respiratory Rate for task post120 Block 41
**respr_post120_42**		text	Respiratory Rate for task post120 Block 42
**respr_post120_43**		text	Respiratory Rate for task post120 Block 43
**respr_post120_44**		text	Respiratory Rate for task post120 Block 44
**respr_post120_45**		text	Respiratory Rate for task post120 Block 45
**respr_post120_46**		text	Respiratory Rate for task post120 Block 46
**respr_post120_47**		text	Respiratory Rate for task post120 Block 47
**respr_post120_48**		text	Respiratory Rate for task post120 Block 48
**respr_post120_49**		text	Respiratory Rate for task post120 Block 49
**respr_post120_50**		text	Respiratory Rate for task post120 Block 50
**respr_post20_1**		text	Respiratory Rate for task post20 Block 1
**respr_post20_2**		text	Respiratory Rate for task post20 Block 2
**respr_post20_3**		text	Respiratory Rate for task post20 Block 3
**respr_post20_4**		text	Respiratory Rate for task post20 Block 4
**respr_post20_5**		text	Respiratory Rate for task post20 Block 5

**respr_post20_6**		text	Respiratory Rate for task post20 Block 6
**respr_post20_7**		text	Respiratory Rate for task post20 Block 7
**respr_post20_8**		text	Respiratory Rate for task post20 Block 8
**respr_post20_9**		text	Respiratory Rate for task post20 Block 9
**respr_post20_10**		text	Respiratory Rate for task post20 Block 10
**respr_post20_11**		text	Respiratory Rate for task post20 Block 11
**respr_post20_12**		text	Respiratory Rate for task post20 Block 12
**respr_post20_13**		text	Respiratory Rate for task post20 Block 13
**respr_post20_14**		text	Respiratory Rate for task post20 Block 14
**respr_post20_15**		text	Respiratory Rate for task post20 Block 15
**respr_post20_16**		text	Respiratory Rate for task post20 Block 16
**respr_post20_17**		text	Respiratory Rate for task post20 Block 17
**respr_post20_18**		text	Respiratory Rate for task post20 Block 18
**respr_post20_19**		text	Respiratory Rate for task post20 Block 19
**respr_post20_20**		text	Respiratory Rate for task post20 Block 20
**respr_post20_21**		text	Respiratory Rate for task post20 Block 21
**respr_post20_22**		text	Respiratory Rate for task post20 Block 22

**respr_post20_23**		text	Respiratory Rate for task post20 Block 23
**respr_post20_24**		text	Respiratory Rate for task post20 Block 24
**respr_post20_25**		text	Respiratory Rate for task post20 Block 25
**respr_post20_26**		text	Respiratory Rate for task post20 Block 26
**respr_post20_27**		text	Respiratory Rate for task post20 Block 27
**respr_post20_28**		text	Respiratory Rate for task post20 Block 28
**respr_post20_29**		text	Respiratory Rate for task post20 Block 29
**respr_post20_30**		text	Respiratory Rate for task post20 Block 30
**respr_post20_31**		text	Respiratory Rate for task post20 Block 31
**respr_post20_32**		text	Respiratory Rate for task post20 Block 32
**respr_post20_33**		text	Respiratory Rate for task post20 Block 33
**respr_post20_34**		text	Respiratory Rate for task post20 Block 34
**respr_post20_35**		text	Respiratory Rate for task post20 Block 35
**respr_post20_36**		text	Respiratory Rate for task post20 Block 36
**respr_post20_37**		text	Respiratory Rate for task post20 Block 37
**respr_post20_38**		text	Respiratory Rate for task post20 Block 38
**respr_post20_39**		text	Respiratory Rate for task post20 Block 39

**respr_post20_40**		text	Respiratory Rate for task post20 Block 40
**respr_post20_41**		text	Respiratory Rate for task post20 Block 41
**respr_post20_42**		text	Respiratory Rate for task post20 Block 42
**respr_post20_43**		text	Respiratory Rate for task post20 Block 43
**respr_post20_44**		text	Respiratory Rate for task post20 Block 44
**respr_post20_45**		text	Respiratory Rate for task post20 Block 45
**respr_post20_46**		text	Respiratory Rate for task post20 Block 46
**respr_post20_47**		text	Respiratory Rate for task post20 Block 47
**respr_post20_48**		text	Respiratory Rate for task post20 Block 48
**respr_post20_49**		text	Respiratory Rate for task post20 Block 49
**respr_post20_50**		text	Respiratory Rate for task post20 Block 50
**respr_post30_1**		text	Respiratory Rate for task post30 Block 1
**respr_post30_2**		text	Respiratory Rate for task post30 Block 2
**respr_post30_3**		text	Respiratory Rate for task post30 Block 3
**respr_post30_4**		text	Respiratory Rate for task post30 Block 4
**respr_post30_5**		text	Respiratory Rate for task post30 Block 5
**respr_post30_6**		text	Respiratory Rate for task post30 Block 6

**respr_post30_7**		text	Respiratory Rate for task post30 Block 7
**respr_post30_8**		text	Respiratory Rate for task post30 Block 8
**respr_post30_9**		text	Respiratory Rate for task post30 Block 9
**respr_post30_10**		text	Respiratory Rate for task post30 Block 10
**respr_post30_11**		text	Respiratory Rate for task post30 Block 11
**respr_post30_12**		text	Respiratory Rate for task post30 Block 12
**respr_post30_13**		text	Respiratory Rate for task post30 Block 13
**respr_post30_14**		text	Respiratory Rate for task post30 Block 14
**respr_post30_15**		text	Respiratory Rate for task post30 Block 15
**respr_post30_16**		text	Respiratory Rate for task post30 Block 16
**respr_post30_17**		text	Respiratory Rate for task post30 Block 17
**respr_post30_18**		text	Respiratory Rate for task post30 Block 18
**respr_post30_19**		text	Respiratory Rate for task post30 Block 19
**respr_post30_20**		text	Respiratory Rate for task post30 Block 20
**respr_post30_21**		text	Respiratory Rate for task post30 Block 21
**respr_post30_22**		text	Respiratory Rate for task post30 Block 22
**respr_post30_23**		text	Respiratory Rate for task post30 Block 23

**respr_post30_24**		text	Respiratory Rate for task post30 Block 24
**respr_post30_25**		text	Respiratory Rate for task post30 Block 25
**respr_post30_26**		text	Respiratory Rate for task post30 Block 26
**respr_post30_27**		text	Respiratory Rate for task post30 Block 27
**respr_post30_28**		text	Respiratory Rate for task post30 Block 28
**respr_post30_29**		text	Respiratory Rate for task post30 Block 29
**respr_post30_30**		text	Respiratory Rate for task post30 Block 30
**respr_post30_31**		text	Respiratory Rate for task post30 Block 31
**respr_post30_32**		text	Respiratory Rate for task post30 Block 32
**respr_post30_33**		text	Respiratory Rate for task post30 Block 33
**respr_post30_34**		text	Respiratory Rate for task post30 Block 34
**respr_post30_35**		text	Respiratory Rate for task post30 Block 35
**respr_post30_36**		text	Respiratory Rate for task post30 Block 36
**respr_post30_37**		text	Respiratory Rate for task post30 Block 37
**respr_post30_38**		text	Respiratory Rate for task post30 Block 38
**respr_post30_39**		text	Respiratory Rate for task post30 Block 39
**respr_post30_40**		text	Respiratory Rate for task post30 Block 40

**respr_post30_41**		text	Respiratory Rate for task post30 Block 41
**respr_post30_42**		text	Respiratory Rate for task post30 Block 42
**respr_post30_43**		text	Respiratory Rate for task post30 Block 43
**respr_post30_44**		text	Respiratory Rate for task post30 Block 44
**respr_post30_45**		text	Respiratory Rate for task post30 Block 45
**respr_post30_46**		text	Respiratory Rate for task post30 Block 46
**respr_post30_47**		text	Respiratory Rate for task post30 Block 47
**respr_post30_48**		text	Respiratory Rate for task post30 Block 48
**respr_post30_49**		text	Respiratory Rate for task post30 Block 49
**respr_post30_50**		text	Respiratory Rate for task post30 Block 50
**respr_post5_1**		text	Respiratory Rate for task post5 Block 1
**respr_post5_2**		text	Respiratory Rate for task post5 Block 2
**respr_post5_3**		text	Respiratory Rate for task post5 Block 3
**respr_post5_4**		text	Respiratory Rate for task post5 Block 4
**respr_post5_5**		text	Respiratory Rate for task post5 Block 5
**respr_post5_6**		text	Respiratory Rate for task post5 Block 6
**respr_post5_7**		text	Respiratory Rate for task post5 Block 7

**respr_post5_8**		text	Respiratory Rate for task post5 Block 8
**respr_post5_9**		text	Respiratory Rate for task post5 Block 9
**respr_post5_10**		text	Respiratory Rate for task post5 Block 10
**respr_post5_11**		text	Respiratory Rate for task post5 Block 11
**respr_post5_12**		text	Respiratory Rate for task post5 Block 12
**respr_post5_13**		text	Respiratory Rate for task post5 Block 13
**respr_post5_14**		text	Respiratory Rate for task post5 Block 14
**respr_post5_15**		text	Respiratory Rate for task post5 Block 15
**respr_post5_16**		text	Respiratory Rate for task post5 Block 16
**respr_post5_17**		text	Respiratory Rate for task post5 Block 17
**respr_post5_18**		text	Respiratory Rate for task post5 Block 18
**respr_post5_19**		text	Respiratory Rate for task post5 Block 19
**respr_post5_20**		text	Respiratory Rate for task post5 Block 20
**respr_post5_21**		text	Respiratory Rate for task post5 Block 21
**respr_post5_22**		text	Respiratory Rate for task post5 Block 22
**respr_post5_23**		text	Respiratory Rate for task post5 Block 23
**respr_post5_24**		text	Respiratory Rate for task post5 Block 24

**respr_post5_25**		text	Respiratory Rate for task post5 Block 25
**respr_post5_26**		text	Respiratory Rate for task post5 Block 26
**respr_post5_27**		text	Respiratory Rate for task post5 Block 27
**respr_post5_28**		text	Respiratory Rate for task post5 Block 28
**respr_post5_29**		text	Respiratory Rate for task post5 Block 29
**respr_post5_30**		text	Respiratory Rate for task post5 Block 30
**respr_post5_31**		text	Respiratory Rate for task post5 Block 31
**respr_post5_32**		text	Respiratory Rate for task post5 Block 32
**respr_post5_33**		text	Respiratory Rate for task post5 Block 33
**respr_post5_34**		text	Respiratory Rate for task post5 Block 34
**respr_post5_35**		text	Respiratory Rate for task post5 Block 35
**respr_post5_36**		text	Respiratory Rate for task post5 Block 36
**respr_post5_37**		text	Respiratory Rate for task post5 Block 37
**respr_post5_38**		text	Respiratory Rate for task post5 Block 38
**respr_post5_39**		text	Respiratory Rate for task post5 Block 39
**respr_post5_40**		text	Respiratory Rate for task post5 Block 40
**respr_post5_41**		text	Respiratory Rate for task post5 Block 41

**respr_post5_42**		text	Respiratory Rate for task post5 Block 42
**respr_post5_43**		text	Respiratory Rate for task post5 Block 43
**respr_post5_44**		text	Respiratory Rate for task post5 Block 44
**respr_post5_45**		text	Respiratory Rate for task post5 Block 45
**respr_post5_46**		text	Respiratory Rate for task post5 Block 46
**respr_post5_47**		text	Respiratory Rate for task post5 Block 47
**respr_post5_48**		text	Respiratory Rate for task post5 Block 48
**respr_post5_49**		text	Respiratory Rate for task post5 Block 49
**respr_post5_50**		text	Respiratory Rate for task post5 Block 50
**respr_post60_1**		text	Respiratory Rate for task post60 Block 1
**respr_post60_2**		text	Respiratory Rate for task post60 Block 2
**respr_post60_3**		text	Respiratory Rate for task post60 Block 3
**respr_post60_4**		text	Respiratory Rate for task post60 Block 4
**respr_post60_5**		text	Respiratory Rate for task post60 Block 5
**respr_post60_6**		text	Respiratory Rate for task post60 Block 6
**respr_post60_7**		text	Respiratory Rate for task post60 Block 7
**respr_post60_8**		text	Respiratory Rate for task post60 Block 8

**respr_post60_9**		text	Respiratory Rate for task post60 Block 9
**respr_post60_10**		text	Respiratory Rate for task post60 Block 10
**respr_post60_11**		text	Respiratory Rate for task post60 Block 11
**respr_post60_12**		text	Respiratory Rate for task post60 Block 12
**respr_post60_13**		text	Respiratory Rate for task post60 Block 13
**respr_post60_14**		text	Respiratory Rate for task post60 Block 14
**respr_post60_15**		text	Respiratory Rate for task post60 Block 15
**respr_post60_16**		text	Respiratory Rate for task post60 Block 16
**respr_post60_17**		text	Respiratory Rate for task post60 Block 17
**respr_post60_18**		text	Respiratory Rate for task post60 Block 18
**respr_post60_19**		text	Respiratory Rate for task post60 Block 19
**respr_post60_20**		text	Respiratory Rate for task post60 Block 20
**respr_post60_21**		text	Respiratory Rate for task post60 Block 21
**respr_post60_22**		text	Respiratory Rate for task post60 Block 22
**respr_post60_23**		text	Respiratory Rate for task post60 Block 23
**respr_post60_24**		text	Respiratory Rate for task post60 Block 24
**respr_post60_25**		text	Respiratory Rate for task post60 Block 25

**respr_post60_26**		text	Respiratory Rate for task post60 Block 26
**respr_post60_27**		text	Respiratory Rate for task post60 Block 27
**respr_post60_28**		text	Respiratory Rate for task post60 Block 28
**respr_post60_29**		text	Respiratory Rate for task post60 Block 29
**respr_post60_30**		text	Respiratory Rate for task post60 Block 30
**respr_post60_31**		text	Respiratory Rate for task post60 Block 31
**respr_post60_32**		text	Respiratory Rate for task post60 Block 32
**respr_post60_33**		text	Respiratory Rate for task post60 Block 33
**respr_post60_34**		text	Respiratory Rate for task post60 Block 34
**respr_post60_35**		text	Respiratory Rate for task post60 Block 35
**respr_post60_36**		text	Respiratory Rate for task post60 Block 36
**respr_post60_37**		text	Respiratory Rate for task post60 Block 37
**respr_post60_38**		text	Respiratory Rate for task post60 Block 38
**respr_post60_39**		text	Respiratory Rate for task post60 Block 39
**respr_post60_40**		text	Respiratory Rate for task post60 Block 40
**respr_post60_41**		text	Respiratory Rate for task post60 Block 41
**respr_post60_42**		text	Respiratory Rate for task post60 Block 42

**respr_post60_43**		text	Respiratory Rate for task post60 Block 43
**respr_post60_44**		text	Respiratory Rate for task post60 Block 44
**respr_post60_45**		text	Respiratory Rate for task post60 Block 45
**respr_post60_46**		text	Respiratory Rate for task post60 Block 46
**respr_post60_48**		text	Respiratory Rate for task post60 Block 48
**respr_post60_49**		text	Respiratory Rate for task post60 Block 49
**respr_post90_1**		text	Respiratory Rate for task post90 Block 1
**respr_post90_2**		text	Respiratory Rate for task post90 Block 2
**respr_post90_3**		text	Respiratory Rate for task post90 Block 3
**respr_post90_4**		text	Respiratory Rate for task post90 Block 4
**respr_post90_5**		text	Respiratory Rate for task post90 Block 5
**respr_post90_6**		text	Respiratory Rate for task post90 Block 6
**respr_post90_7**		text	Respiratory Rate for task post90 Block 7
**respr_post90_8**		text	Respiratory Rate for task post90 Block 8
**respr_post90_9**		text	Respiratory Rate for task post90 Block 9
**respr_post90_10**		text	Respiratory Rate for task post90 Block 10
**respr_post90_11**		text	Respiratory Rate for task post90 Block 11

**respr_post90_12**		text	Respiratory Rate for task post90 Block 12
**respr_post90_13**		text	Respiratory Rate for task post90 Block 13
**respr_post90_14**		text	Respiratory Rate for task post90 Block 14
**respr_post90_15**		text	Respiratory Rate for task post90 Block 15
**respr_post90_16**		text	Respiratory Rate for task post90 Block 16
**respr_post90_17**		text	Respiratory Rate for task post90 Block 17
**respr_post90_18**		text	Respiratory Rate for task post90 Block 18
**respr_post90_19**		text	Respiratory Rate for task post90 Block 19
**respr_post90_20**		text	Respiratory Rate for task post90 Block 20
**respr_post90_21**		text	Respiratory Rate for task post90 Block 21
**respr_post90_22**		text	Respiratory Rate for task post90 Block 22
**respr_post90_23**		text	Respiratory Rate for task post90 Block 23
**respr_post90_24**		text	Respiratory Rate for task post90 Block 24
**respr_post90_25**		text	Respiratory Rate for task post90 Block 25
**respr_post90_26**		text	Respiratory Rate for task post90 Block 26
**respr_post90_27**		text	Respiratory Rate for task post90 Block 27
**respr_post90_28**		text	Respiratory Rate for task post90 Block 28

**respr_post90_29**		text	Respiratory Rate for task post90 Block 29
**respr_post90_30**		text	Respiratory Rate for task post90 Block 30
**respr_post90_31**		text	Respiratory Rate for task post90 Block 31
**respr_post90_32**		text	Respiratory Rate for task post90 Block 32
**respr_post90_33**		text	Respiratory Rate for task post90 Block 33
**respr_post90_34**		text	Respiratory Rate for task post90 Block 34
**respr_post90_35**		text	Respiratory Rate for task post90 Block 35
**respr_post90_36**		text	Respiratory Rate for task post90 Block 36
**respr_post90_37**		text	Respiratory Rate for task post90 Block 37
**respr_post90_38**		text	Respiratory Rate for task post90 Block 38
**respr_post90_39**		text	Respiratory Rate for task post90 Block 39
**respr_post90_40**		text	Respiratory Rate for task post90 Block 40
**respr_post90_41**		text	Respiratory Rate for task post90 Block 41
**respr_post90_42**		text	Respiratory Rate for task post90 Block 42
**respr_post90_43**		text	Respiratory Rate for task post90 Block 43
**respr_post90_44**		text	Respiratory Rate for task post90 Block 44
**respr_post90_45**		text	Respiratory Rate for task post90 Block 45

**respr_post90_46**		text	Respiratory Rate for task post90 Block 46
**respr_post90_47**		text	Respiratory Rate for task post90 Block 47
**respr_post90_48**		text	Respiratory Rate for task post90 Block 48
**respr_post90_49**		text	Respiratory Rate for task post90 Block 49
**respr_post90_50**		text	Respiratory Rate for task post90 Block 50
**respr_pre_1**		text	Respiratory Rate for task pre Block 1
**respr_pre_2**		text	Respiratory Rate for task pre Block 2
**respr_pre_3**		text	Respiratory Rate for task pre Block 3
**respr_pre_4**		text	Respiratory Rate for task pre Block 4
**respr_pre_5**		text	Respiratory Rate for task pre Block 5
**respr_pre_6**		text	Respiratory Rate for task pre Block 6
**respr_pre_7**		text	Respiratory Rate for task pre Block 7
**respr_pre_8**		text	Respiratory Rate for task pre Block 8
**respr_pre_9**		text	Respiratory Rate for task pre Block 9
**respr_pre_10**		text	Respiratory Rate for task pre Block 10
**respr_pre_11**		text	Respiratory Rate for task pre Block 11
**respr_pre_12**		text	Respiratory Rate for task pre Block 12

**respr_pre_13**		text	Respiratory Rate for task pre Block 13
**respr_pre_14**		text	Respiratory Rate for task pre Block 14
**respr_pre_15**		text	Respiratory Rate for task pre Block 15
**respr_pre_16**		text	Respiratory Rate for task pre Block 16
**respr_pre_17**		text	Respiratory Rate for task pre Block 17
**respr_pre_18**		text	Respiratory Rate for task pre Block 18
**respr_pre_19**		text	Respiratory Rate for task pre Block 19
**respr_pre_20**		text	Respiratory Rate for task pre Block 20
**respr_pre_21**		text	Respiratory Rate for task pre Block 21
**respr_pre_22**		text	Respiratory Rate for task pre Block 22
**respr_pre_23**		text	Respiratory Rate for task pre Block 23
**respr_pre_24**		text	Respiratory Rate for task pre Block 24
**respr_pre_25**		text	Respiratory Rate for task pre Block 25
**respr_pre_26**		text	Respiratory Rate for task pre Block 26
**respr_pre_27**		text	Respiratory Rate for task pre Block 27
**respr_pre_28**		text	Respiratory Rate for task pre Block 28
**respr_pre_29**		text	Respiratory Rate for task pre Block 29

**respr_pre_30**		text	Respiratory Rate for task pre Block 30
**respr_pre_31**		text	Respiratory Rate for task pre Block 31
**respr_pre_32**		text	Respiratory Rate for task pre Block 32
**respr_pre_33**		text	Respiratory Rate for task pre Block 33
**respr_pre_34**		text	Respiratory Rate for task pre Block 34
**respr_pre_35**		text	Respiratory Rate for task pre Block 35
**respr_pre_36**		text	Respiratory Rate for task pre Block 36
**respr_pre_37**		text	Respiratory Rate for task pre Block 37
**respr_pre_38**		text	Respiratory Rate for task pre Block 38
**respr_pre_39**		text	Respiratory Rate for task pre Block 39
**respr_pre_40**		text	Respiratory Rate for task pre Block 40
**respr_pre_41**		text	Respiratory Rate for task pre Block 41
**respr_pre_42**		text	Respiratory Rate for task pre Block 42
**respr_pre_44**		text	Respiratory Rate for task pre Block 44
**respr_pre_45**		text	Respiratory Rate for task pre Block 45
**respr_pre_46**		text	Respiratory Rate for task pre Block 46
**respr_pre_47**		text	Respiratory Rate for task pre Block 47

**respr_pre_48**		text	Respiratory Rate for task pre Block 48
**respr_pre_49**		text	Respiratory Rate for task pre Block 49
**respr_pre_50**		text	Respiratory Rate for task pre Block 50
**respr_preptask_1**		text	Respiratory Rate for task preptask Block 1
**respr_preptask_2**		text	Respiratory Rate for task preptask Block 2
**respr_preptask_3**		text	Respiratory Rate for task preptask Block 3
**respr_preptask_4**		text	Respiratory Rate for task preptask Block 4
**respr_preptask_5**		text	Respiratory Rate for task preptask Block 5
**respr_preptask_6**		text	Respiratory Rate for task preptask Block 6
**respr_preptask_7**		text	Respiratory Rate for task preptask Block 7
**respr_preptask_8**		text	Respiratory Rate for task preptask Block 8
**respr_preptask_9**		text	Respiratory Rate for task preptask Block 9
**respr_preptask_10**		text	Respiratory Rate for task preptask Block 10
**respr_preptask_11**		text	Respiratory Rate for task preptask Block 11
**respr_preptask_12**		text	Respiratory Rate for task preptask Block 12
**respr_preptask_13**		text	Respiratory Rate for task preptask Block 13
**respr_preptask_14**		text	Respiratory Rate for task preptask Block 14

**respr_preptask_15**		text	Respiratory Rate for task preptask Block 15
**respr_preptask_16**		text	Respiratory Rate for task preptask Block 16
**respr_preptask_17**		text	Respiratory Rate for task preptask Block 17
**respr_preptask_18**		text	Respiratory Rate for task preptask Block 18
**respr_preptask_19**		text	Respiratory Rate for task preptask Block 19
**respr_preptask_20**		text	Respiratory Rate for task preptask Block 20
**respr_preptask_21**		text	Respiratory Rate for task preptask Block 21
**respr_preptask_22**		text	Respiratory Rate for task preptask Block 22
**respr_preptask_23**		text	Respiratory Rate for task preptask Block 23
**respr_preptask_24**		text	Respiratory Rate for task preptask Block 24
**respr_preptask_25**		text	Respiratory Rate for task preptask Block 25
**respr_preptask_26**		text	Respiratory Rate for task preptask Block 26
**respr_preptask_27**		text	Respiratory Rate for task preptask Block 27
**respr_preptask_28**		text	Respiratory Rate for task preptask Block 28
**respr_preptask_29**		text	Respiratory Rate for task preptask Block 29
**respr_preptask_30**		text	Respiratory Rate for task preptask Block 30
**respr_sst_1**		text	Respiratory Rate for task sst Block 1

**respr_sst_2**		text	Respiratory Rate for task sst Block 2
**respr_sst_3**		text	Respiratory Rate for task sst Block 3
**respr_sst_4**		text	Respiratory Rate for task sst Block 4
**respr_sst_5**		text	Respiratory Rate for task sst Block 5
**respr_sst_6**		text	Respiratory Rate for task sst Block 6
**respr_sst_7**		text	Respiratory Rate for task sst Block 7
**respr_sst_8**		text	Respiratory Rate for task sst Block 8
**respr_sst_9**		text	Respiratory Rate for task sst Block 9
**respr_sst_10**		text	Respiratory Rate for task sst Block 10
**respr_sst_11**		text	Respiratory Rate for task sst Block 11
**respr_sst_12**		text	Respiratory Rate for task sst Block 12
**respr_sst_13**		text	Respiratory Rate for task sst Block 13
**respr_sst_14**		text	Respiratory Rate for task sst Block 14
**respr_sst_15**		text	Respiratory Rate for task sst Block 15
**respr_sst_16**		text	Respiratory Rate for task sst Block 16
**respr_sst_17**		text	Respiratory Rate for task sst Block 17
**respr_sst_18**		text	Respiratory Rate for task sst Block 18

**respr_sst_19**		text	Respiratory Rate for task sst Block 19
**respr_sst_20**		text	Respiratory Rate for task sst Block 20
**respr_sst_21**		text	Respiratory Rate for task sst Block 21
**respr_sst_22**		text	Respiratory Rate for task sst Block 22
**respr_sst_23**		text	Respiratory Rate for task sst Block 23
**respr_sst_24**		text	Respiratory Rate for task sst Block 24
**respr_sst_25**		text	Respiratory Rate for task sst Block 25
**respr_sst_26**		text	Respiratory Rate for task sst Block 26
**respr_sst_27**		text	Respiratory Rate for task sst Block 27
**respr_sst_28**		text	Respiratory Rate for task sst Block 28
**respr_sst_29**		text	Respiratory Rate for task sst Block 29
**respr_sst_30**		text	Respiratory Rate for task sst Block 30
**respr_sst_31**		text	Respiratory Rate for task sst Block 31
**respr_sst_32**		text	Respiratory Rate for task sst Block 32
**respr_sst_33**		text	Respiratory Rate for task sst Block 33
**respr_sst_34**		text	Respiratory Rate for task sst Block 34
**respr_sst_35**		text	Respiratory Rate for task sst Block 35

**respr_sst_36**		text	Respiratory Rate for task sst Block 36
**respr_sst_37**		text	Respiratory Rate for task sst Block 37
**respr_sst_38**		text	Respiratory Rate for task sst Block 38
**respr_sst_39**		text	Respiratory Rate for task sst Block 39
**respr_sst_40**		text	Respiratory Rate for task sst Block 40
**respr_sst_41**		text	Respiratory Rate for task sst Block 41
**respr_sst_42**		text	Respiratory Rate for task sst Block 42
**respr_sst_43**		text	Respiratory Rate for task sst Block 43
**respr_sst_44**		text	Respiratory Rate for task sst Block 44
**respr_sst_45**		text	Respiratory Rate for task sst Block 45
**respr_sst_46**		text	Respiratory Rate for task sst Block 46
**respr_sst_47**		text	Respiratory Rate for task sst Block 47
**respr_sst_48**		text	Respiratory Rate for task sst Block 48
**respr_sst_49**		text	Respiratory Rate for task sst Block 49
**respr_sst_50**		text	Respiratory Rate for task sst Block 50
**respr_st_1**		text	Respiratory Rate for task st Block 1
**respr_st_2**		text	Respiratory Rate for task st Block 2

**respr_st_3**		text	Respiratory Rate for task st Block 3
**respr_st_4**		text	Respiratory Rate for task st Block 4
**respr_st_5**		text	Respiratory Rate for task st Block 5
**respr_st_6**		text	Respiratory Rate for task st Block 6
**respr_st_7**		text	Respiratory Rate for task st Block 7
**respr_st_8**		text	Respiratory Rate for task st Block 8
**respr_st_9**		text	Respiratory Rate for task st Block 9
**respr_st_10**		text	Respiratory Rate for task st Block 10
**respr_st_11**		text	Respiratory Rate for task st Block 11
**respr_st_12**		text	Respiratory Rate for task st Block 12
**respr_st_13**		text	Respiratory Rate for task st Block 13
**respr_st_14**		text	Respiratory Rate for task st Block 14
**respr_st_15**		text	Respiratory Rate for task st Block 15
**respr_st_16**		text	Respiratory Rate for task st Block 16
**respr_st_17**		text	Respiratory Rate for task st Block 17
**respr_st_18**		text	Respiratory Rate for task st Block 18
**respr_st_19**		text	Respiratory Rate for task st Block 19

**respr_st_20**		text	Respiratory Rate for task st Block 20
**respr_st_21**		text	Respiratory Rate for task st Block 21
**respr_st_22**		text	Respiratory Rate for task st Block 22
**respr_st_23**		text	Respiratory Rate for task st Block 23
**respr_st_24**		text	Respiratory Rate for task st Block 24
**respr_st_25**		text	Respiratory Rate for task st Block 25
**respr_st_26**		text	Respiratory Rate for task st Block 26
**respr_st_27**		text	Respiratory Rate for task st Block 27
**respr_st_28**		text	Respiratory Rate for task st Block 28
**respr_st_29**		text	Respiratory Rate for task st Block 29
**respr_st_30**		text	Respiratory Rate for task st Block 30
**respr_st_31**		text	Respiratory Rate for task st Block 31
**respr_st_32**		text	Respiratory Rate for task st Block 32
**respr_st_33**		text	Respiratory Rate for task st Block 33
**respr_st_34**		text	Respiratory Rate for task st Block 34
**respr_st_35**		text	Respiratory Rate for task st Block 35
**respr_st_36**		text	Respiratory Rate for task st Block 36

**respr_st_37**		text	Respiratory Rate for task st Block 37
**respr_st_38**		text	Respiratory Rate for task st Block 38
**respr_st_39**		text	Respiratory Rate for task st Block 39
**respr_st_40**		text	Respiratory Rate for task st Block 40
**respr_st_41**		text	Respiratory Rate for task st Block 41
**respr_st_42**		text	Respiratory Rate for task st Block 42
**respr_st_43**		text	Respiratory Rate for task st Block 43
**respr_st_44**		text	Respiratory Rate for task st Block 44
**respr_st_45**		text	Respiratory Rate for task st Block 45
**respr_st_46**		text	Respiratory Rate for task st Block 46
**respr_st_47**		text	Respiratory Rate for task st Block 47
**respr_st_48**		text	Respiratory Rate for task st Block 48
**respr_st_49**		text	Respiratory Rate for task st Block 49
**respr_st_50**		text	Respiratory Rate for task st Block 50
**respr_task_1**		text	Respiratory Rate for task task Block 1
**respr_task_2**		text	Respiratory Rate for task task Block 2
**respr_task_3**		text	Respiratory Rate for task task Block 3

**respr_task_4**		text	Respiratory Rate for task task Block 4
**respr_task_5**		text	Respiratory Rate for task task Block 5
**respr_task_6**		text	Respiratory Rate for task task Block 6
**respr_task_7**		text	Respiratory Rate for task task Block 7
**respr_task_8**		text	Respiratory Rate for task task Block 8
**respr_task_9**		text	Respiratory Rate for task task Block 9
**respr_task_10**		text	Respiratory Rate for task task Block 10
**respr_task_11**		text	Respiratory Rate for task task Block 11
**respr_task_12**		text	Respiratory Rate for task task Block 12
**respr_task_13**		text	Respiratory Rate for task task Block 13
**respr_task_14**		text	Respiratory Rate for task task Block 14
**respr_task_15**		text	Respiratory Rate for task task Block 15
**respr_task_16**		text	Respiratory Rate for task task Block 16
**respr_task_17**		text	Respiratory Rate for task task Block 17
**respr_task_18**		text	Respiratory Rate for task task Block 18
**respr_task_19**		text	Respiratory Rate for task task Block 19
**respr_task_20**		text	Respiratory Rate for task task Block 20

**respr_task_21**		text	Respiratory Rate for task task Block 21
**respr_task_22**		text	Respiratory Rate for task task Block 22
**respr_task_23**		text	Respiratory Rate for task task Block 23
**respr_task_24**		text	Respiratory Rate for task task Block 24
**respr_task_26**		text	Respiratory Rate for task task Block 26
**respr_task_27**		text	Respiratory Rate for task task Block 27
**respr_task_28**		text	Respiratory Rate for task task Block 28
**respr_task_29**		text	Respiratory Rate for task task Block 29
**respr_task_30**		text	Respiratory Rate for task task Block 30
**respr_task_31**		text	Respiratory Rate for task task Block 31
**respr_task_33**		text	Respiratory Rate for task task Block 33
**respr_task_35**		text	Respiratory Rate for task task Block 35
**respr_task_37**		text	Respiratory Rate for task task Block 37
**respr_task_38**		text	Respiratory Rate for task task Block 38
**respr_task_39**		text	Respiratory Rate for task task Block 39
**respr_task_40**		text	Respiratory Rate for task task Block 40
**respr_task_41**		text	Respiratory Rate for task task Block 41

**respr_task_42**		text	Respiratory Rate for task task Block 42
**respr_task_43**		text	Respiratory Rate for task task Block 43
**respr_task_44**		text	Respiratory Rate for task task Block 44
**respr_task_45**		text	Respiratory Rate for task task Block 45

1.6.1 Skin Conductance Response

RedCAP Form Name: skin_conductance_response

Description: Galvanic skin response recorded during psychophysiological session.

Variable / Field Name	Field Type	Field Label
**scr_abs_hr_time_10_min**	text	Skin Conductance Response Absolute Heart Rate Time 10 Minutes
**scr_duration_10_min**	text	Skin Conductance Response Duration 10 Minutes
**scr_end_time_10_min**	text	Skin Conductance Response End Time Time 10 Minutes
**scr_max_sc_10_min**	text	Skin Conductance Response Maximum Sc 10 Minutes
**scr_mean_sc_10_min**	text	Skin Conductance Response Mean Sc 10 Minutes
**scr_min_sc_10_min**	text	Skin Conductance Response Minimum Sc 10 Minutes
**scr_peak_scl_10_min**	text	Skin Conductance Response Peak Skin Conductance Level 10 Minutes
**scr_peak_time_10_min**	text	Skin Conductance Response Peak Time 10 Minutes
**scr_recovery_time_10_min**	text	Skin Conductance Response Recovery Time Time 10 Minutes
**scr_10_min**	text	Skin Conductance Response 10 Minutes

**scr_response_10_min**	text	Skin Conductance Response Response 10 Minutes
**scr_start_time_10_min**	text	Skin Conductance Response Start Time Time 10 Minutes
**scr_tonic_period_10_min**	text	Skin Conductance Response Tonic Period 10 Minutes
**scr_tonic_scl_10_min**	text	Skin Conductance Response Tonic Skin Conductance Level 10 Minutes
**scr_total_scrs_10_min**	text	Skin Conductance Response Total Skin Conductance Responses 10 Minutes
**scr_trough_scr_10_min**	text	Skin Conductance Response Trough Level Skin Conductance Response 10 Minutes
**scr_trough_time_10_min**	text	Skin Conductance Response Trough Level Time 10 Minutes
**scr_abs_hr_time_120_min**	text	Skin Conductance Response Absolute Heart Rate Time 120 Minimum
**scr_duration_120_min**	text	Skin Conductance Response Duration 120 Minimum
**scr_end_time_120_min**	text	Skin Conductance Response End Time Time 120 Minimum
**scr_max_sc_120_min**	text	Skin Conductance Response Maximum Sc 120 Minimum
**scr_mean_sc_120_min**	text	Skin Conductance Response Mean Sc 120 Minimum
**scr_min_sc_120_min**	text	Skin Conductance Response Minimum Sc 120 Minimum
**scr_peak_scl_120_min**	text	Skin Conductance Response Peak Skin Conductance Level 120 Minimum
**scr_peak_time_120_min**	text	Skin Conductance Response Peak Time 120 Minimum
**scr_recovery_time_120_min**	text	Skin Conductance Response Recovery Time Time 120 Minimum
**scr_120_min**	text	Skin Conductance Response 120 Minimum

**scr_response_120_min**	text	Skin Conductance Response Response 120 Minimum
**scr_start_time_120_min**	text	Skin Conductance Response Start Time Time 120 Minimum
**scr_tonic_period_120_min**	text	Skin Conductance Response Tonic Period 120 Minimum
**scr_tonic_scl_120_min**	text	Skin Conductance Response Tonic Skin Conductance Level 120 Minimum
**scr_total_scrs_120_min**	text	Skin Conductance Response Total Skin Conductance Responses 120 Minimum
**scr_trough_scr_120_min**	text	Skin Conductance Response Trough Level Skin Conductance Response 120 Minimum
**scr_trough_time_120_min**	text	Skin Conductance Response Trough Level Time 120 Minimum
**scr_abs_hr_time_20_min**	text	Skin Conductance Response Absolute Heart Rate Time 20 Minimum
**scr_duration_20_min**	text	Skin Conductance Response Duration 20 Minimum
**scr_end_time_20_min**	text	Skin Conductance Response End Time Time 20 Minimum
**scr_max_sc_20_min**	text	Skin Conductance Response Maximum Sc 20 Minimum
**scr_mean_sc_20_min**	text	Skin Conductance Response Mean Sc 20 Minimum
**scr_min_sc_20_min**	text	Skin Conductance Response Minimum Sc 20 Minimum
**scr_peak_scl_20_min**	text	Skin Conductance Response Peak Skin Conductance Level 20 Minimum
**scr_peak_time_20_min**	text	Skin Conductance Response Peak Time 20 Minimum
**scr_recovery_time_20_min**	text	Skin Conductance Response Recovery Time Time 20 Minimum
**scr_20_min**	text	Skin Conductance Response 20 Minimum

**scr_response_20_min**	text	Skin Conductance Response Response 20 Minimum
**scr_start_time_20_min**	text	Skin Conductance Response Start Time Time 20 Minimum
**scr_tonic_period_20_min**	text	Skin Conductance Response Tonic Period 20 Minimum
**scr_tonic_scl_20_min**	text	Skin Conductance Response Tonic Skin Conductance Level 20 Minimum
**scr_total_scrs_20_min**	text	Skin Conductance Response Total Skin Conductance Responses 20 Minimum
**scr_trough_scr_20_min**	text	Skin Conductance Response Trough Level Skin Conductance Response 20 Minimum
**scr_trough_time_20_min**	text	Skin Conductance Response Trough Level Time 20 Minimum
**scr_abs_hr_time_30_min**	text	Skin Conductance Response Absolute Heart Rate Time 30 Minimum
**scr_duration_30_min**	text	Skin Conductance Response Duration 30 Minimum
**scr_end_time_30_min**	text	Skin Conductance Response End Time Time 30 Minimum
**scr_max_sc_30_min**	text	Skin Conductance Response Maximum Sc 30 Minimum
**scr_mean_sc_30_min**	text	Skin Conductance Response Mean Sc 30 Minimum
**scr_min_sc_30_min**	text	Skin Conductance Response Minimum Sc 30 Minimum
**scr_peak_scl_30_min**	text	Skin Conductance Response Peak Skin Conductance Level 30 Minimum
**scr_peak_time_30_min**	text	Skin Conductance Response Peak Time 30 Minimum
**scr_recovery_time_30_min**	text	Skin Conductance Response Recovery Time Time 30 Minimum
**scr_30_min**	text	Skin Conductance Response 30 Minimum

**scr_response_30_min**	text	Skin Conductance Response Response 30 Minimum
**scr_start_time_30_min**	text	Skin Conductance Response Start Time Time 30 Minimum
**scr_tonic_period_30_min**	text	Skin Conductance Response Tonic Period 30 Minimum
**scr_tonic_scl_30_min**	text	Skin Conductance Response Tonic Skin Conductance Level 30 Minimum
**scr_total_scrs_30_min**	text	Skin Conductance Response Total Skin Conductance Responses 30 Minimum
**scr_trough_scr_30_min**	text	Skin Conductance Response Trough Level Skin Conductance Response 30 Minimum
**scr_trough_time_30_min**	text	Skin Conductance Response Trough Level Time 30 Minimum
**scr_abs_hr_time_5_min**	text	Skin Conductance Response Absolute Heart Rate Time 5 Minutes
**scr_duration_5_min**	text	Skin Conductance Response Duration 5 Minutes
**scr_end_time_5_min**	text	Skin Conductance Response End Time Time 5 Minutes
**scr_max_sc_5_min**	text	Skin Conductance Response Maximum Sc 5 Minutes
**scr_mean_sc_5_min**	text	Skin Conductance Response Mean Sc 5 Minutes
**scr_min_sc_5_min**	text	Skin Conductance Response Minimum Sc 5 Minutes
**scr_peak_scl_5_min**	text	Skin Conductance Response Peak Skin Conductance Level 5 Minutes
**scr_peak_time_5_min**	text	Skin Conductance Response Peak Time 5 Minutes
**scr_recovery_time_5_min**	text	Skin Conductance Response Recovery Time Time 5 Minutes
**scr_5_min**	text	Skin Conductance Response 5 Minutes

**scr_response_5_min**	text	Skin Conductance Response Response 5 Minutes
**scr_start_time_5_min**	text	Skin Conductance Response Start Time Time 5 Minutes
**scr_tonic_period_5_min**	text	Skin Conductance Response Tonic Period 5 Minutes
**scr_tonic_scl_5_min**	text	Skin Conductance Response Tonic Skin Conductance Level 5 Minutes
**scr_total_scrs_5_min**	text	Skin Conductance Response Total Skin Conductance Responses 5 Minutes
**scr_trough_scr_5_min**	text	Skin Conductance Response Trough Level Skin Conductance Response 5 Minutes
**scr_trough_time_5_min**	text	Skin Conductance Response Trough Level Time 5 Minutes
**scr_abs_hr_time_60_min**	text	Skin Conductance Response Absolute Heart Rate Time 60 Minimum
**scr_duration_60_min**	text	Skin Conductance Response Duration 60 Minimum
**scr_end_time_60_min**	text	Skin Conductance Response End Time Time 60 Minimum
**scr_max_sc_60_min**	text	Skin Conductance Response Maximum Sc 60 Minimum
**scr_mean_sc_60_min**	text	Skin Conductance Response Mean Sc 60 Minimum
**scr_min_sc_60_min**	text	Skin Conductance Response Minimum Sc 60 Minimum
**scr_peak_scl_60_min**	text	Skin Conductance Response Peak Skin Conductance Level 60 Minimum
**scr_peak_time_60_min**	text	Skin Conductance Response Peak Time 60 Minimum
**scr_recovery_time_60_min**	text	Skin Conductance Response Recovery Time Time 60 Minimum
**scr_60_min**	text	Skin Conductance Response 60 Minimum

**scr_response_60_min**	text	Skin Conductance Response Response 60 Minimum
**scr_start_time_60_min**	text	Skin Conductance Response Start Time Time 60 Minimum
**scr_tonic_period_60_min**	text	Skin Conductance Response Tonic Period 60 Minimum
**scr_tonic_scl_60_min**	text	Skin Conductance Response Tonic Skin Conductance Level 60 Minimum
**scr_total_scrs_60_min**	text	Skin Conductance Response Total Skin Conductance Responses 60 Minimum
**scr_trough_scr_60_min**	text	Skin Conductance Response Trough Level Skin Conductance Response 60 Minimum
**scr_trough_time_60_min**	text	Skin Conductance Response Trough Level Time 60 Minimum
**scr_abs_hr_time_90_min**	text	Skin Conductance Response Absolute Heart Rate Time 90 Minimum
**scr_duration_90_min**	text	Skin Conductance Response Duration 90 Minimum
**scr_end_time_90_min**	text	Skin Conductance Response End Time Time 90 Minimum
**scr_max_sc_90_min**	text	Skin Conductance Response Maximum Sc 90 Minimum
**scr_mean_sc_90_min**	text	Skin Conductance Response Mean Sc 90 Minimum
**scr_min_sc_90_min**	text	Skin Conductance Response Minimum Sc 90 Minimum
**scr_peak_scl_90_min**	text	Skin Conductance Response Peak Skin Conductance Level 90 Minimum
**scr_peak_time_90_min**	text	Skin Conductance Response Peak Time 90 Minimum
**scr_recovery_time_90_min**	text	Skin Conductance Response Recovery Time Time 90 Minimum
**scr_90_min**	text	Skin Conductance Response 90 Minimum

**scr_response_90_min**	text	Skin Conductance Response Response 90 Minimum
**scr_start_time_90_min**	text	Skin Conductance Response Start Time Time 90 Minimum
**scr_tonic_period_90_min**	text	Skin Conductance Response Tonic Period 90 Minimum
**scr_tonic_scl_90_min**	text	Skin Conductance Response Tonic Skin Conductance Level 90 Minimum
**scr_total_scrs_90_min**	text	Skin Conductance Response Total Skin Conductance Responses 90 Minimum
**scr_trough_scr_90_min**	text	Skin Conductance Response Trough Level Skin Conductance Response 90 Minimum
**scr_trough_time_90_min**	text	Skin Conductance Response Trough Level Time 90 Minimum
**scr_abs_hr_time_cp_b**	text	Skin Conductance Response Absolute Heart Rate Time Cp baseline
**scr_duration_cp_b**	text	Skin Conductance Response Duration Cp baseline
**scr_end_time_cp_b**	text	Skin Conductance Response End Time Time Cp baseline
**scr_max_sc_cp_b**	text	Skin Conductance Response Maximum Sc Cp baseline
**scr_mean_sc_cp_b**	text	Skin Conductance Response Mean Sc Cp baseline
**scr_min_sc_cp_b**	text	Skin Conductance Response Minimum Sc Cp baseline
**scr_peak_scl_cp_b**	text	Skin Conductance Response Peak Skin Conductance Level Cp baseline
**scr_peak_time_cp_b**	text	Skin Conductance Response Peak Time Cp baseline
**scr_recovery_time_cp_b**	text	Skin Conductance Response Recovery Time Time Cp baseline
**scr_cp_b**	text	Skin Conductance Response Cp baseline

**scr_response_cp_b**	text	Skin Conductance Response Response Cp baseline
**scr_start_time_cp_b**	text	Skin Conductance Response Start Time Time Cp baseline
**scr_tonic_period_cp_b**	text	Skin Conductance Response Tonic Period Cp baseline
**scr_tonic_scl_cp_b**	text	Skin Conductance Response Tonic Skin Conductance Level Cp baseline
**scr_total_scrs_cp_b**	text	Skin Conductance Response Total Skin Conductance Responses Cp baseline
**scr_trough_scr_cp_b**	text	Skin Conductance Response Trough Level Skin Conductance Response Cp baseline
**scr_trough_time_cp_b**	text	Skin Conductance Response Trough Level Time Cp baseline
**scr_abs_hr_time_cp_r**	text	Skin Conductance Response Absolute Heart Rate Time CP recovery
**scr_duration_cp_r**	text	Skin Conductance Response Duration CP recovery
**scr_end_time_cp_r**	text	Skin Conductance Response End Time Time CP recovery
**scr_max_sc_cp_r**	text	Skin Conductance Response Maximum Sc CP recovery
**scr_mean_sc_cp_r**	text	Skin Conductance Response Mean Sc CP recovery
**scr_min_sc_cp_r**	text	Skin Conductance Response Minimum Sc CP recovery
**scr_peak_scl_cp_r**	text	Skin Conductance Response Peak Skin Conductance Level CP recovery
**scr_peak_time_cp_r**	text	Skin Conductance Response Peak Time CP recovery
**scr_recovery_time_cp_r**	text	Skin Conductance Response Recovery Time Time CP recovery
**scr_cp_r**	text	Skin Conductance Response CP recovery

**scr_response_cp_r**	text	Skin Conductance Response Response CP recovery
**scr_start_time_cp_r**	text	Skin Conductance Response Start Time Time CP recovery
**scr_tonic_period_cp_r**	text	Skin Conductance Response Tonic Period CP recovery
**scr_tonic_scl_cp_r**	text	Skin Conductance Response Tonic Skin Conductance Level CP recovery
**scr_total_scrs_cp_r**	text	Skin Conductance Response Total Skin Conductance Responses CP recovery
**scr_trough_scr_cp_r**	text	Skin Conductance Response Trough Level Skin Conductance Response CP recovery
**scr_trough_time_cp_r**	text	Skin Conductance Response Trough Level Time CP recovery
**scr_abs_hr_time_cp_task**	text	Skin Conductance Response Absolute Heart Rate Time Cp Task
**scr_duration_cp_task**	text	Skin Conductance Response Duration Cp Task
**scr_end_time_cp_task**	text	Skin Conductance Response End Time Time Cp Task
**scr_max_sc_cp_task**	text	Skin Conductance Response Maximum Sc Cp Task
**scr_mean_sc_cp_task**	text	Skin Conductance Response Mean Sc Cp Task
**scr_min_sc_cp_task**	text	Skin Conductance Response Minimum Sc Cp Task
**scr_peak_scl_cp_task**	text	Skin Conductance Response Peak Skin Conductance Level Cp Task
**scr_peak_time_cp_task**	text	Skin Conductance Response Peak Time Cp Task
**scr_recovery_time_cp_task**	text	Skin Conductance Response Recovery Time Time Cp Task
**scr_cp_task**	text	Skin Conductance Response Cp Task

**scr_response_cp_task**	text	Skin Conductance Response Response Cp Task
**scr_start_time_cp_task**	text	Skin Conductance Response Start Time Time Cp Task
**scr_tonic_period_cp_task**	text	Skin Conductance Response Tonic Period Cp Task
**scr_tonic_scl_cp_task**	text	Skin Conductance Response Tonic Skin Conductance Level Cp Task
**scr_total_scrs_cp_task**	text	Skin Conductance Response Total Skin Conductance Responses Cp Task
**scr_trough_scr_cp_task**	text	Skin Conductance Response Trough Level Skin Conductance Response Cp Task
**scr_trough_time_cp_task**	text	Skin Conductance Response Trough Level Time Cp Task
**scr_abs_hr_time_cp_pre**	text	Skin Conductance Response Absolute Heart Rate Time Cp Pre
**scr_duration_cp_pre**	text	Skin Conductance Response Duration Cp Pre
**scr_end_time_cp_pre**	text	Skin Conductance Response End Time Time Cp Pre
**scr_max_sc_cp_pre**	text	Skin Conductance Response Maximum Sc Cp Pre
**scr_mean_sc_cp_pre**	text	Skin Conductance Response Mean Sc Cp Pre
**scr_min_sc_cp_pre**	text	Skin Conductance Response Minimum Sc Cp Pre
**scr_peak_scl_cp_pre**	text	Skin Conductance Response Peak Skin Conductance Level Cp Pre
**scr_peak_time_cp_pre**	text	Skin Conductance Response Peak Time Cp Pre
**scr_recovery_time_cp_pre**	text	Skin Conductance Response Recovery Time Time Cp Pre
**scr_cp_pre**	text	Skin Conductance Response Cp Pre

**scr_response_cp_pre**	text	Skin Conductance Response Response Cp Pre
**scr_start_time_cp_pre**	text	Skin Conductance Response Start Time Time Cp Pre
**scr_tonic_period_cp_pre**	text	Skin Conductance Response Tonic Period Cp Pre
**scr_tonic_scl_cp_pre**	text	Skin Conductance Response Tonic Skin Conductance Level Cp Pre
**scr_total_scrs_cp_pre**	text	Skin Conductance Response Total Skin Conductance Responses Cp Pre
**scr_trough_scr_cp_pre**	text	Skin Conductance Response Trough Level Skin Conductance Response Cp Pre
**scr_trough_time_cp_pre**	text	Skin Conductance Response Trough Level Time Cp Pre
**scr_abs_hr_time_dbt_b**	text	Skin Conductance Response Absolute Heart Rate Time Dbt baseline
**scr_duration_dbt_b**	text	Skin Conductance Response Duration Dbt baseline
**scr_end_time_dbt_b**	text	Skin Conductance Response End Time Time Dbt baseline
**scr_max_sc_dbt_b**	text	Skin Conductance Response Maximum Sc Dbt baseline
**scr_mean_sc_dbt_b**	text	Skin Conductance Response Mean Sc Dbt baseline
**scr_min_sc_dbt_b**	text	Skin Conductance Response Minimum Sc Dbt baseline
**scr_peak_scl_dbt_b**	text	Skin Conductance Response Peak Skin Conductance Level Dbt baseline
**scr_peak_time_dbt_b**	text	Skin Conductance Response Peak Time Dbt baseline
**scr_recovery_time_dbt_b**	text	Skin Conductance Response Recovery Time Time Dbt baseline
**scr_dbt_b**	text	Skin Conductance Response Dbt baseline

**scr_response_dbt_b**	text	Skin Conductance Response Response Dbt baseline
**scr_start_time_dbt_b**	text	Skin Conductance Response Start Time Time Dbt baseline
**scr_tonic_period_dbt_b**	text	Skin Conductance Response Tonic Period Dbt baseline
**scr_tonic_scl_dbt_b**	text	Skin Conductance Response Tonic Skin Conductance Level Dbt baseline
**scr_total_scrs_dbt_b**	text	Skin Conductance Response Total Skin Conductance Responses Dbt baseline
**scr_trough_scr_dbt_b**	text	Skin Conductance Response Trough Level Skin Conductance Response Dbt baseline
**scr_trough_time_dbt_b**	text	Skin Conductance Response Trough Level Time Dbt baseline
**scr_abs_hr_time_dbt_task**	text	Skin Conductance Response Absolute Heart Rate Time Dbt Task
**scr_duration_dbt_task**	text	Skin Conductance Response Duration Dbt Task
**scr_end_time_dbt_task**	text	Skin Conductance Response End Time Time Dbt Task
**scr_max_sc_dbt_task**	text	Skin Conductance Response Maximum Sc Dbt Task
**scr_mean_sc_dbt_task**	text	Skin Conductance Response Mean Sc Dbt Task
**scr_min_sc_dbt_task**	text	Skin Conductance Response Minimum Sc Dbt Task
**scr_peak_scl_dbt_task**	text	Skin Conductance Response Peak Skin Conductance Level Dbt Task
**scr_peak_time_dbt_task**	text	Skin Conductance Response Peak Time Dbt Task
**scr_recovery_time_dbt_task**	text	Skin Conductance Response Recovery Time Time Dbt Task
**scr_dbt_task**	text	Skin Conductance Response Dbt Task

**scr_response_dbt_task**	text	Skin Conductance Response Response Dbt Task
**scr_start_time_dbt_task**	text	Skin Conductance Response Start Time Time Dbt Task
**scr_tonic_period_dbt_task**	text	Skin Conductance Response Tonic Period Dbt Task
**scr_tonic_scl_dbt_task**	text	Skin Conductance Response Tonic Skin Conductance Level Dbt Task
**scr_total_scrs_dbt_task**	text	Skin Conductance Response Total Skin Conductance Responses Dbt Task
**scr_trough_scr_dbt_task**	text	Skin Conductance Response Trough Level Skin Conductance Response Dbt Task
**scr_trough_time_dbt_task**	text	Skin Conductance Response Trough Level Time Dbt Task
**scr_abs_hr_time_dbt_pre**	text	Skin Conductance Response Absolute Heart Rate Time Dbt Pre
**scr_duration_dbt_pre**	text	Skin Conductance Response Duration Dbt Pre
**scr_end_time_dbt_pre**	text	Skin Conductance Response End Time Time Dbt Pre
**scr_max_sc_dbt_pre**	text	Skin Conductance Response Maximum Sc Dbt Pre
**scr_mean_sc_dbt_pre**	text	Skin Conductance Response Mean Sc Dbt Pre
**scr_min_sc_dbt_pre**	text	Skin Conductance Response Minimum Sc Dbt Pre
**scr_peak_scl_dbt_pre**	text	Skin Conductance Response Peak Skin Conductance Level Dbt Pre
**scr_peak_time_dbt_pre**	text	Skin Conductance Response Peak Time Dbt Pre
**scr_recovery_time_dbt_pre**	text	Skin Conductance Response Recovery Time Time Dbt Pre
**scr_dbt_pre**	text	Skin Conductance Response Dbt Pre

**scr_response_dbt_pre**	text	Skin Conductance Response Response Dbt Pre
**scr_start_time_dbt_pre**	text	Skin Conductance Response Start Time Time Dbt Pre
**scr_tonic_period_dbt_pre**	text	Skin Conductance Response Tonic Period Dbt Pre
**scr_tonic_scl_dbt_pre**	text	Skin Conductance Response Tonic Skin Conductance Level Dbt Pre
**scr_total_scrs_dbt_pre**	text	Skin Conductance Response Total Skin Conductance Responses Dbt Pre
**scr_trough_scr_dbt_pre**	text	Skin Conductance Response Trough Level Skin Conductance Response Dbt Pre
**scr_trough_time_dbt_pre**	text	Skin Conductance Response Trough Level Time Dbt Pre
**scr_abs_hr_time_instructions**	text	Skin Conductance Response Absolute Heart Rate Time Instructions
**scr_duration_instructions**	text	Skin Conductance Response Duration Instructions
**scr_end_time_instructions**	text	Skin Conductance Response End Time Time Instructions
**scr_max_sc_instructions**	text	Skin Conductance Response Maximum Sc Instructions
**scr_mean_sc_instructions**	text	Skin Conductance Response Mean Sc Instructions
**scr_min_sc_instructions**	text	Skin Conductance Response Minimum Sc Instructions
**scr_peak_scl_instructions**	text	Skin Conductance Response Peak Skin Conductance Level Instructions
**scr_peak_time_instructions**	text	Skin Conductance Response Peak Time Instructions
**scr_recovery_time_instructions**	text	Skin Conductance Response Recovery Time Time Instructions
**scr_instructions**	text	Skin Conductance Response Instructions

**scr_response_instructions**	text	Skin Conductance Response Response Instructions
**scr_start_time_instructions**	text	Skin Conductance Response Start Time Time Instructions
**scr_tonic_period_instructions**	text	Skin Conductance Response Tonic Period Instructions
**scr_tonic_scl_instructions**	text	Skin Conductance Response Tonic Skin Conductance Level Instructions
**scr_total_scrs_instructions**	text	Skin Conductance Response Total Skin Conductance Responses Instructions
**scr_trough_scr_instructions**	text	Skin Conductance Response Trough Level Skin Conductance Response Instructions
**scr_trough_time_instructions**	text	Skin Conductance Response Trough Level Time Instructions
**scr_abs_hr_time_prep_task**	text	Skin Conductance Response Absolute Heart Rate Time Prep Task
**scr_duration_prep_task**	text	Skin Conductance Response Duration Prep Task
**scr_end_time_prep_task**	text	Skin Conductance Response End Time Time Prep Task
**scr_max_sc_prep_task**	text	Skin Conductance Response Maximum Sc Prep Task
**scr_mean_sc_prep_task**	text	Skin Conductance Response Mean Sc Prep Task
**scr_min_sc_prep_task**	text	Skin Conductance Response Minimum Sc Prep Task
**scr_peak_scl_prep_task**	text	Skin Conductance Response Peak Skin Conductance Level Prep Task
**scr_peak_time_prep_task**	text	Skin Conductance Response Peak Time Prep Task
**scr_recovery_time_prep_task**	text	Skin Conductance Response Recovery Time Time Prep Task
**scr_prep_task**	text	Skin Conductance Response Prep Task

**scr_response_prep_task**	text	Skin Conductance Response Response Prep Task
**scr_start_time_prep_task**	text	Skin Conductance Response Start Time Time Prep Task
**scr_tonic_period_prep_task**	text	Skin Conductance Response Tonic Period Prep Task
**scr_tonic_scl_prep_task**	text	Skin Conductance Response Tonic Skin Conductance Level Prep Task
**scr_total_scrs_prep_task**	text	Skin Conductance Response Total Skin Conductance Responses Prep Task
**scr_trough_scr_prep_task**	text	Skin Conductance Response Trough Level Skin Conductance Response Prep Task
**scr_trough_time_prep_task**	text	Skin Conductance Response Trough Level Time Prep Task
**scr_abs_hr_time_sst_30s_pre**	text	Skin Conductance Response Absolute Heart Rate Time Sst 30s Pre
**scr_duration_sst_30s_pre**	text	Skin Conductance Response Duration Sst 30s Pre
**scr_end_time_sst_30s_pre**	text	Skin Conductance Response End Time Time Sst 30s Pre
**scr_max_sc_sst_30s_pre**	text	Skin Conductance Response Maximum Sc Sst 30s Pre
**scr_mean_sc_sst_30s_pre**	text	Skin Conductance Response Mean Sc Sst 30s Pre
**scr_min_sc_sst_30s_pre**	text	Skin Conductance Response Minimum Sc Sst 30s Pre
**scr_peak_scl_sst_30s_pre**	text	Skin Conductance Response Peak Skin Conductance Level Sst 30s Pre
**scr_peak_time_sst_30s_pre**	text	Skin Conductance Response Peak Time Sst 30s Pre
**scr_recovery_time_sst_30s_pre**	text	Skin Conductance Response Recovery Time Time Sst 30s Pre
**scr_sst_30s_pre**	text	Skin Conductance Response Sst 30s Pre

**scr_response_sst_30s_pre**	text	Skin Conductance Response Response Sst 30s Pre
**scr_start_time_sst_30s_pre**	text	Skin Conductance Response Start Time Time Sst 30s Pre
**scr_tonic_period_sst_30s_pre**	text	Skin Conductance Response Tonic Period Sst 30s Pre
**scr_tonic_scl_sst_30s_pre**	text	Skin Conductance Response Tonic Skin Conductance Level Sst 30s Pre
**scr_total_scrs_sst_30s_pre**	text	Skin Conductance Response Total Skin Conductance Responses Sst 30s Pre
**scr_trough_scr_sst_30s_pre**	text	Skin Conductance Response Trough Level Skin Conductance Response Sst 30s Pre
**scr_trough_time_sst_30s_pre**	text	Skin Conductance Response Trough Level Time Sst 30s Pre
**scr_abs_hr_time_sst_b**	text	Skin Conductance Response Absolute Heart Rate Time Sst baseline
**scr_duration_sst_b**	text	Skin Conductance Response Duration Sst baseline
**scr_end_time_sst_b**	text	Skin Conductance Response End Time Time Sst baseline
**scr_max_sc_sst_b**	text	Skin Conductance Response Maximum Sc Sst baseline
**scr_mean_sc_sst_b**	text	Skin Conductance Response Mean Sc Sst baseline
**scr_min_sc_sst_b**	text	Skin Conductance Response Minimum Sc Sst baseline
**scr_peak_scl_sst_b**	text	Skin Conductance Response Peak Skin Conductance Level Sst baseline
**scr_peak_time_sst_b**	text	Skin Conductance Response Peak Time Sst baseline
**scr_recovery_time_sst_b**	text	Skin Conductance Response Recovery Time Time Sst baseline
**scr_sst_b**	text	Skin Conductance Response Sst baseline

**scr_response_sst_b**	text	Skin Conductance Response Response Sst baseline
**scr_start_time_sst_b**	text	Skin Conductance Response Start Time Time Sst baseline
**scr_tonic_period_sst_b**	text	Skin Conductance Response Tonic Period Sst baseline
**scr_tonic_scl_sst_b**	text	Skin Conductance Response Tonic Skin Conductance Level Sst baseline
**scr_total_scrs_sst_b**	text	Skin Conductance Response Total Skin Conductance Responses Sst baseline
**scr_trough_scr_sst_b**	text	Skin Conductance Response Trough Level Skin Conductance Response Sst baseline
**scr_trough_time_sst_b**	text	Skin Conductance Response Trough Level Time Sst baseline
**scr_abs_hr_time_sst_r**	text	Skin Conductance Response Absolute Heart Rate Time SST recovery
**scr_duration_sst_r**	text	Skin Conductance Response Duration SST recovery
**scr_end_time_sst_r**	text	Skin Conductance Response End Time Time SST recovery
**scr_max_sc_sst_r**	text	Skin Conductance Response Maximum Sc SST recovery
**scr_mean_sc_sst_r**	text	Skin Conductance Response Mean Sc SST recovery
**scr_min_sc_sst_r**	text	Skin Conductance Response Minimum Sc SST recovery
**scr_peak_scl_sst_r**	text	Skin Conductance Response Peak Skin Conductance Level SST recovery
**scr_peak_time_sst_r**	text	Skin Conductance Response Peak Time SST recovery
**scr_recovery_time_sst_r**	text	Skin Conductance Response Recovery Time Time SST recovery
**scr_sst_r**	text	Skin Conductance Response SST recovery

**scr_response_sst_r**	text	Skin Conductance Response Response SST recovery
**scr_start_time_sst_r**	text	Skin Conductance Response Start Time Time SST recovery
**scr_tonic_period_sst_r**	text	Skin Conductance Response Tonic Period SST recovery
**scr_tonic_scl_sst_r**	text	Skin Conductance Response Tonic Skin Conductance Level SST recovery
**scr_total_scrs_sst_r**	text	Skin Conductance Response Total Skin Conductance Responses SST recovery
**scr_trough_scr_sst_r**	text	Skin Conductance Response Trough Level Skin Conductance Response SST recovery
**scr_trough_time_sst_r**	text	Skin Conductance Response Trough Level Time SST recovery
**scr_abs_hr_time_sst_task**	text	Skin Conductance Response Absolute Heart Rate Time Sst Task
**scr_duration_sst_task**	text	Skin Conductance Response Duration Sst Task
**scr_end_time_sst_task**	text	Skin Conductance Response End Time Time Sst Task
**scr_max_sc_sst_task**	text	Skin Conductance Response Maximum Sc Sst Task
**scr_mean_sc_sst_task**	text	Skin Conductance Response Mean Sc Sst Task
**scr_min_sc_sst_task**	text	Skin Conductance Response Minimum Sc Sst Task
**scr_peak_scl_sst_task**	text	Skin Conductance Response Peak Skin Conductance Level Sst Task
**scr_peak_time_sst_task**	text	Skin Conductance Response Peak Time Sst Task
**scr_recovery_time_sst_task**	text	Skin Conductance Response Recovery Time Time Sst Task
**scr_sst_task**	text	Skin Conductance Response Sst Task

**scr_response_sst_task**	text	Skin Conductance Response Response Sst Task
**scr_start_time_sst_task**	text	Skin Conductance Response Start Time Time Sst Task
**scr_tonic_period_sst_task**	text	Skin Conductance Response Tonic Period Sst Task
**scr_tonic_scl_sst_task**	text	Skin Conductance Response Tonic Skin Conductance Level Sst Task
**scr_total_scrs_sst_task**	text	Skin Conductance Response Total Skin Conductance Responses Sst Task
**scr_trough_scr_sst_task**	text	Skin Conductance Response Trough Level Skin Conductance Response Sst Task
**scr_trough_time_sst_task**	text	Skin Conductance Response Trough Level Time Sst Task
**scr_abs_hr_time_st_b**	text	Skin Conductance Response Absolute Heart Rate Time St baseline
**scr_duration_st_b**	text	Skin Conductance Response Duration St baseline
**scr_end_time_st_b**	text	Skin Conductance Response End Time Time St baseline
**scr_max_sc_st_b**	text	Skin Conductance Response Maximum Sc St baseline
**scr_mean_sc_st_b**	text	Skin Conductance Response Mean Sc St baseline
**scr_min_sc_st_b**	text	Skin Conductance Response Minimum Sc St baseline
**scr_peak_scl_st_b**	text	Skin Conductance Response Peak Skin Conductance Level St baseline
**scr_peak_time_st_b**	text	Skin Conductance Response Peak Time St baseline
**scr_recovery_time_st_b**	text	Skin Conductance Response Recovery Time Time St baseline
**scr_st_b**	text	Skin Conductance Response St baseline

**scr_response_st_b**	text	Skin Conductance Response Response St baseline
**scr_start_time_st_b**	text	Skin Conductance Response Start Time Time St baseline
**scr_tonic_period_st_b**	text	Skin Conductance Response Tonic Period St baseline
**scr_tonic_scl_st_b**	text	Skin Conductance Response Tonic Skin Conductance Level St baseline
**scr_total_scrs_st_b**	text	Skin Conductance Response Total Skin Conductance Responses St baseline
**scr_trough_scr_st_b**	text	Skin Conductance Response Trough Level Skin Conductance Response St baseline
**scr_trough_time_st_b**	text	Skin Conductance Response Trough Level Time St baseline
**scr_abs_hr_time_st_pre**	text	Skin Conductance Response Absolute Heart Rate Time St Pre
**scr_duration_st_pre**	text	Skin Conductance Response Duration St Pre
**scr_end_time_st_pre**	text	Skin Conductance Response End Time Time St Pre
**scr_max_sc_st_pre**	text	Skin Conductance Response Maximum Sc St Pre
**scr_mean_sc_st_pre**	text	Skin Conductance Response Mean Sc St Pre
**scr_min_sc_st_pre**	text	Skin Conductance Response Minimum Sc St Pre
**scr_peak_scl_st_pre**	text	Skin Conductance Response Peak Skin Conductance Level St Pre
**scr_peak_time_st_pre**	text	Skin Conductance Response Peak Time St Pre
**scr_recovery_time_st_pre**	text	Skin Conductance Response Recovery Time Time St Pre
**scr_st_pre**	text	Skin Conductance Response St Pre

**scr_response_st_pre**	text	Skin Conductance Response Response St Pre
**scr_start_time_st_pre**	text	Skin Conductance Response Start Time Time St Pre
**scr_tonic_period_st_pre**	text	Skin Conductance Response Tonic Period St Pre
**scr_tonic_scl_st_pre**	text	Skin Conductance Response Tonic Skin Conductance Level St Pre
**scr_total_scrs_st_pre**	text	Skin Conductance Response Total Skin Conductance Responses St Pre
**scr_trough_scr_st_pre**	text	Skin Conductance Response Trough Level Skin Conductance Response St Pre
**scr_trough_time_st_pre**	text	Skin Conductance Response Trough Level Time St Pre
**scr_abs_hr_time_st_task**	text	Skin Conductance Response Absolute Heart Rate Time St Task
**scr_duration_st_task**	text	Skin Conductance Response Duration St Task
**scr_end_time_st_task**	text	Skin Conductance Response End Time Time St Task
**scr_max_sc_st_task**	text	Skin Conductance Response Maximum Sc St Task
**scr_mean_sc_st_task**	text	Skin Conductance Response Mean Sc St Task
**scr_min_sc_st_task**	text	Skin Conductance Response Minimum Sc St Task
**scr_peak_scl_st_task**	text	Skin Conductance Response Peak Skin Conductance Level St Task
**scr_peak_time_st_task**	text	Skin Conductance Response Peak Time St Task
**scr_recovery_time_st_task**	text	Skin Conductance Response Recovery Time Time St Task
**scr_st_task**	text	Skin Conductance Response St Task

**scr_response_st_task**	text	Skin Conductance Response Response St Task
**scr_start_time_st_task**	text	Skin Conductance Response Start Time Time St Task
**scr_tonic_period_st_task**	text	Skin Conductance Response Tonic Period St Task
**scr_tonic_scl_st_task**	text	Skin Conductance Response Tonic Skin Conductance Level St Task
**scr_total_scrs_st_task**	text	Skin Conductance Response Total Skin Conductance Responses St Task
**scr_trough_scr_st_task**	text	Skin Conductance Response Trough Level Skin Conductance Response St Task
**scr_trough_time_st_task**	text	Skin Conductance Response Trough Level Time St Task
**scr_abs_hr_time_task**	text	Skin Conductance Response Absolute Heart Rate Time Task
**scr_duration_task**	text	Skin Conductance Response Duration Task
**scr_end_time_task**	text	Skin Conductance Response End Time Time Task
**scr_max_sc_task**	text	Skin Conductance Response Maximum Sc Task
**scr_mean_sc_task**	text	Skin Conductance Response Mean Sc Task
**scr_min_sc_task**	text	Skin Conductance Response Minimum Sc Task
**scr_peak_scl_task**	text	Skin Conductance Response Peak Skin Conductance Level Task
**scr_peak_time_task**	text	Skin Conductance Response Peak Time Task
**scr_recovery_time_task**	text	Skin Conductance Response Recovery Time Time Task
**scr_task**	text	Skin Conductance Response Task

**scr_response_task**	text	Skin Conductance Response Response Task
**scr_start_time_task**	text	Skin Conductance Response Start Time Time Task
**scr_tonic_period_task**	text	Skin Conductance Response Tonic Period Task
**scr_tonic_scl_task**	text	Skin Conductance Response Tonic Skin Conductance Level Task
**scr_total_scrs_task**	text	Skin Conductance Response Total Skin Conductance Responses Task
**scr_trough_scr_task**	text	Skin Conductance Response Trough Level Skin Conductance Response Task
**scr_trough_time_task**	text	Skin Conductance Response Trough Level Time Task
**scr_abs_hr_time_baseline**	text	Skin Conductance Response Absolute Heart Rate Time baselineaseline
**scr_duration_baseline**	text	Skin Conductance Response Duration baselineaseline
**scr_end_time_baseline**	text	Skin Conductance Response End Time Time baselineaseline
**scr_max_sc_baseline**	text	Skin Conductance Response Maximum Sc baselineaseline
**scr_mean_sc_baseline**	text	Skin Conductance Response Mean Sc baselineaseline
**scr_min_sc_baseline**	text	Skin Conductance Response Minimum Sc baselineaseline
**scr_peak_scl_baseline**	text	Skin Conductance Response Peak Skin Conductance Level baselineaseline
**scr_peak_time_baseline**	text	Skin Conductance Response Peak Time baselineaseline
**scr_recovery_time_baseline**	text	Skin Conductance Response Recovery Time Time baselineaseline
**scr_baseline**	text	Skin Conductance Response baselineaseline

**scr_response_baseline**	text	Skin Conductance Response Response baselineaseline
**scr_start_time_baseline**	text	Skin Conductance Response Start Time Time baselineaseline
**scr_tonic_period_baseline**	text	Skin Conductance Response Tonic Period baselineaseline
**scr_tonic_scl_baseline**	text	Skin Conductance Response Tonic Skin Conductance Level baselineaseline
**scr_total_scrs_baseline**	text	Skin Conductance Response Total Skin Conductance Responses baselineaseline
**scr_trough_scr_baseline**	text	Skin Conductance Response Trough Level Skin Conductance Response baselineaseline
**scr_trough_time_baseline**	text	Skin Conductance Response Trough Level Time baselineaseline
**scr_abs_hr_time_pre_m5_min**	text	Skin Conductance Response Absolute Heart Rate Time Pre Minus 5 Minutes
**scr_duration_pre_m5_min**	text	Skin Conductance Response Duration Pre Minus 5 Minutes
**scr_end_time_pre_m5_min**	text	Skin Conductance Response End Time Time Pre Minus 5 Minutes
**scr_max_sc_pre_m5_min**	text	Skin Conductance Response Maximum Sc Pre Minus 5 Minutes
**scr_mean_sc_pre_m5_min**	text	Skin Conductance Response Mean Sc Pre Minus 5 Minutes
**scr_min_sc_pre_m5_min**	text	Skin Conductance Response Minimum Sc Pre Minus 5 Minutes
**scr_peak_scl_pre_m5_min**	text	Skin Conductance Response Peak Skin Conductance Level Pre Minus 5 Minutes
**scr_peak_time_pre_m5_min**	text	Skin Conductance Response Peak Time Pre Minus 5 Minutes
**scr_recovery_time_pre_m5_min**	text	Skin Conductance Response Recovery Time Time Pre Minus 5 Minutes
**scr_pre_m5_min**	text	Skin Conductance Response Pre Minus 5 Minutes

**scr_response_pre_m5_min**	text	Skin Conductance Response Response Pre Minus 5 Minutes
**scr_start_time_pre_m5_min**	text	Skin Conductance Response Start Time Time Pre Minus 5 Minutes
**scr_tonic_period_pre_m5_min**	text	Skin Conductance Response Tonic Period Pre Minus 5 Minutes
**scr_tonic_scl_pre_m5_min**	text	Skin Conductance Response Tonic Skin Conductance Level Pre Minus 5 Minutes
**scr_total_scrs_pre_m5_min**	text	Skin Conductance Response Total Skin Conductance Responses Pre Minus 5 Minutes
**scr_trough_scr_pre_m5_min**	text	Skin Conductance Response Trough Level Skin Conductance Response Pre Minus 5 Minutes
**scr_trough_time_pre_m5_min**	text	Skin Conductance Response Trough Level Time Pre Minus 5 Minutes

2. Questionnaire Variables

2.1 Day 0

2.1.1 Day 0 Hotel

Redcap Form Name: Day 0 Questionnaire

Questionnaire Name	Title	Variable Name
2.1.1.1 SF-36 SRH Question	SF-36 SRH Question (SRH)	srh
2.1.1.2 Modified Differential Emotions Scale	Modified Differential Emotions Scale (mDES)	mdes
2.1.1.3 My Daily Stress	My Daily Stress (DISE)	dise

Variable / Field Name	Form Name	Section Header	Field Type	Field Label	Choices, Calculations, OR Slider Labels
**srh**	day_0_questionnaire	General Health<br>The following questions will ask you about your general health.	radio	In general, would you say that your health is:	1, Excellent | 2, Very Good | 3, Good | 4, Fair | 5, Poor

**mdes_amused_d0**	day_0_questionnaire	Emotions<br>For each of the emotions listed below, please tell us how much you have felt that emotion this evening.<br>Please choose one of the following answers for each question:	radio	1. I felt amused, fun-loving, or silly.	0, Not at all | 1, A little bit | 2, Moderately | 3, Quite a bit | 4, Extremely
**mdes_angry_d0**	day_0_questionnaire		radio	2. I felt angry, irritated, or annoyed.	0, Not at all | 1, A little bit | 2, Moderately | 3, Quite a bit | 4, Extremely
**mdes_ashamed_d0**	day_0_questionnaire		radio	3. I felt ashamed, humiliated, or disgraced.	0, Not at all | 1, A little bit | 2, Moderately | 3, Quite a bit | 4, Extremely
**mdes_awe_d0**	day_0_questionnaire		radio	4. I felt awe, wonder, or amazement.	0, Not at all | 1, A little bit | 2, Moderately | 3, Quite a bit | 4, Extremely
**mdes_bored_d0**	day_0_questionnaire		radio	5. I felt bored, disinterested, uninvolved.	0, Not at all | 1, A little bit | 2, Moderately | 3, Quite a bit | 4, Extremely
**mdes_contemptuous_d0**	day_0_questionnaire		radio	6. I felt contemptuous, scornful, or disdainful.	0, Not at all | 1, A little bit | 2, Moderately | 3, Quite a bit | 4, Extremely
**mdes_control_d0**	day_0_questionnaire		radio	7. I felt control, coping well, on top of things.	0, Not at all | 1, A little bit | 2, Moderately | 3, Quite a bit | 4, Extremely
**mdes_disgust_d0**	day_0_questionnaire		radio	8. I felt disgust, distaste, or revulsion.	0, Not at all | 1, A little bit | 2, Moderately | 3, Quite a bit | 4, Extremely
**mdes_embarrassed_d0**	day_0_questionnaire		radio	9. I felt embarrassed, self-conscious, or blushing.	0, Not at all | 1, A little bit | 2, Moderately | 3, Quite a bit | 4, Extremely

**mdes_excited_d0**	day_0_questionnaire		radio	10. I felt excited, eager, enthusiastic.	0, Not at all | 1, A little bit | 2, Moderately | 3, Quite a bit | 4, Extremely
**mdes_grateful_d0**	day_0_questionnaire		radio	11. I felt grateful, appreciative, or thankful.	0, Not at all | 1, A little bit | 2, Moderately | 3, Quite a bit | 4, Extremely
**mdes_guilty_d0**	day_0_questionnaire		radio	12. I felt guilty, repentant, or blameworthy.	0, Not at all | 1, A little bit | 2, Moderately | 3, Quite a bit | 4, Extremely
**mdes_hate_d0**	day_0_questionnaire		radio	13. I felt hatred, distrust, or suspicion.	0, Not at all | 1, A little bit | 2, Moderately | 3, Quite a bit | 4, Extremely
**mdes_hopeful_d0**	day_0_questionnaire		radio	14. I felt hopeful, optimistic, or encouraged.	0, Not at all | 1, A little bit | 2, Moderately | 3, Quite a bit | 4, Extremely
**mdes_inspired_d0**	day_0_questionnaire		radio	15. I felt inspired, uplifted, or elevated.	0, Not at all | 1, A little bit | 2, Moderately | 3, Quite a bit | 4, Extremely
**mdes_interested_d0**	day_0_questionnaire		radio	16. I felt interested, alert, or curious.	0, Not at all | 1, A little bit | 2, Moderately | 3, Quite a bit | 4, Extremely
**mdes_joyful_d0**	day_0_questionnaire		radio	17. I felt joyful, glad, or happy.	0, Not at all | 1, A little bit | 2, Moderately | 3, Quite a bit | 4, Extremely
**mdes_love_d0**	day_0_questionnaire		radio	18. I felt love, closeness, or trust.	0, Not at all | 1, A little bit | 2, Moderately | 3, Quite a bit | 4, Extremely

**mdes_proud_d0**	day_0_questionnaire		radio	19. I felt proud, confident, or self-assured.	0, Not at all | 1, A little bit | 2, Moderately | 3, Quite a bit | 4, Extremely
**mdes_rejected_d0**	day_0_questionnaire		radio	20. I felt rejected, betrayed, left-behind.	0, Not at all | 1, A little bit | 2, Moderately | 3, Quite a bit | 4, Extremely
**mdes_sad_d0**	day_0_questionnaire		radio	21. I felt sad, downhearted, or unhappy.	0, Not at all | 1, A little bit | 2, Moderately | 3, Quite a bit | 4, Extremely
**mdes_satisfied_d0**	day_0_questionnaire		radio	22. I felt satisfied, fulfilled, content.	0, Not at all | 1, A little bit | 2, Moderately | 3, Quite a bit | 4, Extremely
**mdes_scared_d0**	day_0_questionnaire		radio	23. I felt scared, fearful, or afraid.	0, Not at all | 1, A little bit | 2, Moderately | 3, Quite a bit | 4, Extremely
**mdes_stressed_d0**	day_0_questionnaire		radio	24. I felt stressed, nervous, or overwhelmed.	0, Not at all | 1, A little bit | 2, Moderately | 3, Quite a bit | 4, Extremely
**mdes_tired_d0**	day_0_questionnaire		radio	25. I felt tired, sleepy, drowsy.	0, Not at all | 1, A little bit | 2, Moderately | 3, Quite a bit | 4, Extremely
**mdes_sextual_d0**	day_0_questionnaire		radio	26. I felt sexual, desiring, flirtatious.	0, Not at all | 1, A little bit | 2, Moderately | 3, Quite a bit | 4, Extremely
**mdes_sympathy_d0**	day_0_questionnaire		radio	27. I felt sympathy, concern, compassion.	0, Not at all | 1, A little bit | 2, Moderately | 3, Quite a bit | 4, Extremely

**dise_stress_time_d0**	day_0_questionnaire	My daily stress<br>We all have events that happen on a daily basis that are considered stressful. We are interested in one actual event that caused you the MOST stress today. This one event could be something major or something minor. It may involve your spouse, your child/children, something at work, finances, or something else.<br>Using the questions below, please describe, with as many details as possible, the event in your life that caused you the most stress today. We are interested in what actually happened, in other words how the event unfolded.	dropdown	1. At approximately what time of the day did this occur?	0, 12 AM | 1, 1 M | 2, 2 AM | 3, 3AM | 4, 4AM | 5, 5AM | 6, 6AM | 7, 7AM | 8, 8AM | 9, 9AM | 10, 10AM | 11, 11AM | 12, 12PM | 13, 1PM | 14, 2PM | 15, 3PM | 16, 4PM | 17, 5PM | 18, 6PM | 19, 7PM | 20, 8PM | 21, 9PM | 22, 10PM | 23, 11PM
**dise_stresslength_d0**	day_0_questionnaire		radio	2. How long did this event last?	1, For several minutes | 2, For up to an hour | 3, For several hours | 4, For the rest of the day
**dise_howstressful_d0**	day_0_questionnaire		radio	3. How stressful do you think the average person would find this event?	0, Not at all stressful | 1, A little stressful | 2, Somewhat stressful | 3, Moderately stressful | 4, Very stressful
**dise_stresstype_d0**	day_0_questionnaire		checkbox	4. Please review the following list and then select the category or categories from the following list that best describe the stressor described above.	1, Housing | 2, Money/Possessions | 3, Crime / Legal | 4, Your own health | 5, The health/death of a loved one | 6, Caregiving for child/children | 7, Marital/Partner relationship | 8, Other family / Household member relationships | 9, Education | 10, Work | 11, Lack of sleep | 12, Other

**dise_instructions_d0**	day_0_questionnaire		text	Please describe (in a max of 5 words):	
**dise_stressful_d0**	day_0_questionnaire		slider	5. How stressful was this situation for you, today, at its peak?	Not at all | Somewhat | Extremely
**dise_angry_d0**	day_0_questionnaire		slider	6. How angry did you feel at the peak of this stressor?	Not at all | Somewhat | Extremely
**dise_anxious_d0**	day_0_questionnaire		slider	7. How anxious did you feel at the peak of this stressor?	Not at all | Somewhat | Extremely
**dise_sad_d0**	day_0_questionnaire		slider	8. How sad did you feel, at the peak of this stressor?	Not at all | Somewhat | Extremely
**dise_shame_d0**	day_0_questionnaire		slider	9. How much shame did you feel, at the peak of this stressor?	Not at all | Somewhat | Extremely
**dise_length_d0**	day_0_questionnaire		radio	10. For how long afterward did your negative emotions last?	1, Not at all | 2, For several minutes afterwards | 3, For up to an hour afterwards | 4, For more than an hour | 5, For the rest of the day
**dise_stressprevalence_d0**	day_0_questionnaire		radio	11. To what extent did you find yourself thinking about this stressful situation in the rest of your day afterward?	0, Not at all | 1, A little | 2, Moderately | 3, Quite a bit | 4, A lot
**dise_control_d0**	day_0_questionnaire		radio	12. Do you feel that you have control over the stressful situation (not	0, Not at all | 1, A little | 2, Moderately | 3, Quite a bit | 4, A lot

				your reaction to it, but the actual situation)?	
**dise_resources_d0**	day_0_questionnaire		radio	13. To what extent do you have the resources (emotional, interpersonal, or cognitive) to deal with this situation?	0, Not at all | 1, A little | 2, Moderately | 3, Quite a bit | 4, A lot
**dise_demanding_d0**	day_0_questionnaire		radio	14. How demanding is it to deal with this situation?	0, Not at all | 1, A little | 2, Moderately | 3, Quite a bit | 4, A lot
**dise_handlestress_d0**	day_0_questionnaire		radio	15. To what extent do you feel you are able to effectively handle this situation?	0, Not at all | 1, A little | 2, Moderately | 3, Quite a bit | 4, A lot

2.2 Day 1

2.2.1 Post Breakfast Questionnaire

Redcap Form Name: Day 1 Post Breakfast Questionnaire

**Questionnaire Name**	**Title**	**Variable Name**
2.2.1.1 Participant Information	Participant Information (PI)	pi
2.2.1.2 Physical Activity	International Physical Activity Questionnaire (IPAQ)	ipaq
2.2.1.3 Fat and Sugar	Short Questionnaire for Fat and Free Sugars (SQFFS)	sqffs
2.2.1.4 Food Frequency	Food Frequency Inventory (FFI)	ffi
2.2.1.5 Vitamins and Supplements	MESA Vitamin Use Subscale (MESA)	vit

**Variable / Field Name**	**Form Name**	**Section Header**	**Field Type**	**Field Label**	**Choices, Calculations, OR Slider Labels**

**day1_post_breakfastcv**	day_1_postbreakfast_questionnaire		descriptive	Thank you for your participation in the MiSBIE study!<br>The following questions are a validated set of questionnaires that will help us understand you better as a person. It is very important that you answer each question as well as you can.<br>This package should take about 30 min to complete.<br>We want to take this opportunity to remind you that all answers<br>will remain strictly confidential.	
**pi_genderid**	day_1_postbreakfast_questionnaire		radio	What best describes your current gender identity?	1, Male | 2, Female | 3, I prefer to use another term (e.g., gender fluid, nonbinary)
**pi_genderid_nb**	day_1_postbreakfast_questionnaire		text	Please specify:	
**pi_sex**	day_1_postbreakfast_questionnaire		radio	What sex were you assigned at birth, meaning on your original birth certificate?	1, Male | 2, Female
**pi_race**	day_1_postbreakfast_questionnaire		checkbox	What is your race/ethnicity (choose all that apply)?	1, American Indian or Alaska Native | 2, Asian | 3, Black or African American | 4, Hispanic or Latino | 5, Native Hawaiian or Other Pacific Islander | 6, White
**pi_handedness**	day_1_postbreakfast_questionnaire		radio	Which is your dominant hand?	1, Left| 2, Right| 3, Ambidextrous
**pi_married**	day_1_postbreakfast_questionnaire	Social Web	yes/no	Are you married or living with someone?	

**pi_kids**	day_1_postbreakfast_questionnaire		yes/no	Do you have children?	
**pi_numberkids**	day_1_postbreakfast_questionnaire		dropdown	How many children do you have?	1, 1 | 2, 2 | 3, 3 | 4, 4 | 5, 5 | 6, 6 | 7, 7 | 8, 8 | 9, 9 | 10, 10 | 11, More than 10
**pi_siblings**	day_1_postbreakfast_questionnaire		yes/no	Do you have brothers or sisters?	
**pi_numbersiblings**	day_1_postbreakfast_questionnaire		dropdown	How many brothers or sisters do you have?	1, 1 | 2, 2 | 3, 3 | 4, 4 | 5, 5 | 6, 6 | 7, 7 | 8, 8 | 9, 9 | 10, 10 | 11, More than 10
**pi_religious**	day_1_postbreakfast_questionnaire		yes/no	Do you actively practice a religion?	
**pi_religiontype**	day_1_postbreakfast_questionnaire		dropdown	Which religion do you practice?	1, Christian | 2, Jewish | 3, Muslim | 4, Jehovah's Witness | 5, Mormon | 6, Other
**pi_religionother**	day_1_postbreakfast_questionnaire		text	Please specify:	
**pi_religionfrequency**	day_1_postbreakfast_questionnaire		radio	How often do you attend religious meetings?	1, More than once a week | 2, Once a week | 3, A few times a month | 4, Once a month | 5, A few times a year | 6, Never
**pi_smoke**	day_1_postbreakfast_questionnaire	Life Habits	yes/no	Do you smoke?	
**pi_smoketype**	day_1_postbreakfast_questionnaire		checkbox	What do you smoke? (Choose all that apply)	1, Cigarettes | 2, Cigars | 3, Pipes | 4, E-cigs | 5, Other

**pi_smokeother**	day_1_postbreakfast_questionnaire		text	Please specify:	
**pi_smokingfrequency**	day_1_postbreakfast_questionnaire		dropdown	How many of these do you smoke per day?	1, 1 | 2, 2 | 3, 3 | 4, 4 | 5, 5 | 6, 6 | 7, 7 | 8, 8 | 9, 9 | 10, 10 | 11, 11 | 12, 12 | 13, 13 | 14, 14 | 15, 15 | 16, 16 | 17, 17 | 18, 18 | 19, 19 | 20, 20 | 21, 21 | 22, 22 | 23, 23 | 24, 24 | 25, 25 | 26, 26 | 27, 27 | 28, 28 | 29, 29 | 30, 30 | 31, 31 | 32, 32 | 33, 33 | 34, 34 | 35, 35 | 36, 36 | 37, 37 | 38, 38 | 39, 39 | 40, 40 | 41, 41 | 42, 42 | 43, 43 | 44, 44 | 45, 45 | 46, 46 | 47, 47 | 48, 48 | 49, 49 | 50, 50
**pi_nicotine_replc**	day_1_postbreakfast_questionnaire		yes/no	Do you use nicotine replacement therapy?	
**pi_nicotine_replc_type**	day_1_postbreakfast_questionnaire		checkbox	What nicotine replacement therapy do you use? (Choose all that apply)	1, Gum | 2, Inhalers | 3, Lozenges | 4, Nasal spray | 5, Skin patch | 6, Other (please describe):
**pi_nicotine_replc_other**	day_1_postbreakfast_questionnaire		text	please describe:	
**pi_nicotine_replc_freq**	day_1_postbreakfast_questionnaire		dropdown	How many of these do you use per day?	1, 1 | 2, 2 | 3, 3 | 4, 4 | 5, 5 | 6, 6 | 7, 7 | 8, 8 | 9, 9 | 10, 10 | 11, 11 | 12, 12 | 13, 13 | 14, 14 | 15, 15 | 16, 16 | 17, 17 | 18, 18 | 19, 19 | 20, 20 | 21, 21 | 22, 22 | 23, 23 | 24, 24 | 25, 25 | 26, 26 | 27, 27 | 28, 28 | 29, 29 | 30, 30 | 31, 31 | 32, 32 | 33, 33 | 34, 34 | 35, 35 | 36, 36 | 37, 37 | 38, 38 | 39, 39 | 40, 40 | 41, 41 | 42, 42 | 43, 43 | 44, 44 | 45,

					45 | 46, 46 | 47, 47 | 48, 48 | 49, 49 | 50, 50 and more
**pi_alcohol12m**	day_1_postbreakfast_questionnaire		radio	How often have you had an alcoholic drink of any kind during the last 12 months?	1, Almost every day | 2, Five or six days a week | 3, Three or four days a week | 4, Once or twice a week | 5, Once or twice a month | 6, Once every couple of months | 7, Once or twice a year | 8, Not at all in the last 12 months
**pi_alcoholperday**	day_1_postbreakfast_questionnaire		dropdown	In general, how many glasses of alcohol do you drink?	1, 1 or 2 glasses per week | 2, 3 or 4 glasses per week | 3, 5 or 6 glasses per week | 4, 7 or 9 glasses per week | 5, 10 glasses or more per week
**pi_alcoholtype**	day_1_postbreakfast_questionnaire		checkbox	In general, what is the type of alcohol you drink? (Choose all that apply)	6, Normal strength beer/lager/stout/cider/shandy | 7, Strong beer/lager/stout/cider | 8, Spirits or liqueurs | 9, Sherry or martini | 10, Wine | 11, Alcopops/pre-mixed alcoholic drinks | 12, Other alcoholic drinks | 13, Low alcohol drinks only
**pi_highschool**	day_1_postbreakfast_questionnaire	Work	yes/no	Did you finish high school?	
**pi_postsecondary**	day_1_postbreakfast_questionnaire		dropdown	If so, how many years of school did you do after high school?	1, 1 | 2, 2 | 3, 3 | 4, 4 | 5, 5 | 6, 6 | 7, 7 | 8, 8 | 9, 9 | 10, 10 | 11, More than 10
**pi_work**	day_1_postbreakfast_questionnaire		radio	How would you define your current work status?	1, Unemployed | 2, Working for pay or profit | 3, Pupil, student, further training, or

					unpaid work experience | 4, In retirement, early retirement, or has given up business | 5, Permanently disabled | 6, In compulsory military or community service | 7, Fulfilling domestic tasks | 8, Other
**pi_workother**	day_1_postbreakfast_ questionnaire		text	Please specify:	
**pi_professional**	day_1_postbreakfast_ questionnaire		dropdown	How would you define your current professional category? (Choose the category that most closely applies)	1, Managers | 2, Professionals | 3, Technicians and associate professionals | 4, Clerical support workers | 5, Service and sales workers | 6, Skilled agricultural, forestry and fishery workers | 7, Craft and related trades workers | 8, Plant and machine operators and assemblers | 9, Elementary occupations | 10, Armed forces occupations
**pi_job**	day_1_postbreakfast_ questionnaire		text	What is your profession?	
**pi_income**	day_1_postbreakfast_ questionnaire		radio	What is the yearly income of your household?	1, Less than $20,000 | 2, Between $20,000 and $59,999 | 3, Between $60,000 and $99,999 | 4, Between $100,000and $149,999 | 5, Between $150,000 and $199,999 | 6, Between $200,000 and $249,999 | 7, Over $250,000

**pi_healtheffect**	day_1_postbreakfast_questionnaire	Health	radio	To what extent does your health condition keep you from doing things you would like?	1, Not at all | 2, A little | 3, Very much
**pi_doctorvisits**	day_1_postbreakfast_questionnaire		radio	On average, how many times per year do you see a doctor?	1, Once a week | 2, Once every 2 weeks | 3, Once a month | 4, Between 2 and 12 times a year | 5, Once a year | 6, Less than once a year
**pi_doctortype**	day_1_postbreakfast_questionnaire		checkbox	What kind of doctor(s) do you generally see (neurologist, gynecologist, etc.)? (Choose all that apply)	1, Allergist or Immunologist | 2, Anesthesiologist | 3, Cardiologist | 4, Dermatologist | 5, Gastroenterologist | 6, Nephrologist | 7, Neurologist | 8, Neurosurgeon | 9, Obstetrician | 10, Gynecologist | 11, Occupational Medicine Physician | 12, Ophthalmologist | 13, Pathologist | 14, Plastic Surgeon | 15, Podiatrist | 16, Psychiatrist | 17, Pulmonary Medicine Physician | 18, Rheumatologist | 19, Urologist | 20, Other
**pi_inhaler**	day_1_postbreakfast_questionnaire		yes/no	Do you use a prescribed inhaler for a respiratory disorder?	
**pi_inhalerpurpose**	day_1_postbreakfast_questionnaire		text	If so, for what purpose?	
**pi_respdiagnosis**	day_1_postbreakfast_questionnaire		yes/no	Have you been diagnosed with a respiratory illness?	

**pi_respdisease**	day_1_postbreakfast_questionnaire		radio	If yes, which one?	1, Chronic Obstructive Pulmonary Disease (COPD) | 2, Asthma | 3, Other
**pi_respdiseaseother**	day_1_postbreakfast_questionnaire		text	Please specify:	
**pi_hospital12m**	day_1_postbreakfast_questionnaire		yes/no	In the last year, have you been hospitalized?	
**pi_hospitalfrequency**	day_1_postbreakfast_questionnaire		dropdown	How many times have you been hospitalized in the last year?	1, 1 | 2, 2 | 3, 3 | 4, 4 | 5, 5 | 6, 6 | 7, 7 | 8, 8 | 9, 9 | 10, 10 | 11, 11 | 12, 12 | 13, 13 | 14, 14 | 15, 15 | 16, 16 | 17, 17 | 18, 18 | 19, 19 | 20, 20 | 21, 21 | 22, 22 | 23, 23 | 24, 24 | 25, 25 | 26, 26 | 27, 27 | 28, 28 | 29, 29 | 30, 30 | 31, 31 | 32, 32 | 33, 33 | 34, 34 | 35, 35 | 36, 36 | 37, 37 | 38, 38 | 39, 39 | 40, 40 | 41, 41 | 42, 42 | 43, 43 | 44, 44 | 45, 45 | 46, 46 | 47, 47 | 48, 48 | 49, 49 | 50, 50
**pi_glassescontacts**	day_1_postbreakfast_questionnaire		yes/no	Do you wear contact lenses or glasses?	
**pi_hairproduct**	day_1_postbreakfast_questionnaire		yes/no	Do you use any hair dying products?	
**pi_hairproductdate**	day_1_postbreakfast_questionnaire		text	When was the last time you used any hair dying or hair coloring products?	
**pi_menstruation**	day_1_postbreakfast_questionnaire		yes/no	Do you have menstrual periods?	

**pi_menstruationdate**	day_1_postbreakfast_questionnaire		text	If so, when was the last day of your last menstrual period?	
**pi_menstruationlength**	day_1_postbreakfast_questionnaire		dropdown	How long are your periods?	1, <2 days | 2, 2 days | 3, 3 days | 4, 4 days | 5, 5 days | 6, 6 days | 7, 7 days | 8, 8 days | 9, 9 days | 10, 10 days | 11, > 10 days
**pi_contraceptive**	day_1_postbreakfast_questionnaire		yes/no	Do you use any contraceptive method?	
**pi_contraceptivetype**	day_1_postbreakfast_questionnaire		checkbox	What kind of contraceptive method do you use? (Choose all that apply)	1, The Pill (i.e., hormonal contraception) | 2, Condoms | 3, Contraceptive patch (e.g., Xulane) | 4, Vaginal contraceptive ring (e.g., NuvaRing) | 5, IUD (e.g., Mirena) | 6, Implants (e.g., Nexplanon) | 7, Injection (e.g., Depo-Provera) | 8, Sterilization (e.g., tubal ligation) | 9, Diaphragm | 10, Spermicides | 11, Other
**pi_contraceptiveother**	day_1_postbreakfast_questionnaire		text	Please specify:	
**qnair_physicalact**	day_1_postbreakfast_questionnaire	Physical Activity	radio	During the past month, which statement best describes the kinds of physical activity you usually did? Do not include the time you spent working at a job. Please read all six statements before selecting one.	1, I did not do much physical activity. I mostly did things like watching television, reading, playing cards, or playing computer games. Only occasionally, no more than once or twice a month, did I do anything more active such as going for a walk or playing tennis. | 2, Once or twice a week, I did light activities such as getting outdoors on the

					weekends for an easy walk or stroll. Or once or twice a week, I did chores around the house such as sweeping floors or vacuuming. | 3, About three times a week, I did moderate activities such as brisk walking, swimming, or riding a bike for about 15-20 minutes each time. Or about once a week, I did moderately difficult chores such as raking or mowing the lawn for about 45-60 minutes. Or about once a week, I played sports such as softball, basketball, or soccer for about 45-60 minutes. | 4, Almost daily, that is five or more times a week, I did moderate activities such as brisk walking, swimming, or riding a bike for 30 minutes or more each time. Or about once a week, I did moderately difficult chores or played sports for 2 hours or more. | 5, About three times a week, I did vigorous activities such as running or riding hard on a bike for 30 minutes or more each time. | 6, Almost daily, that is five or more times a week, I did vigorous activities such as running or riding hard on a bike for 30 minutes or more each time.

**lcat_1**	day_1_postbreakfast_questionnaire	INTERNATIONAL PHYSICAL ACTIVITY QUESTIONNAIRE<br><br>We are interested in finding out about the kinds of physical activities that people do as part of their everyday lives. The questions will ask you about the time you spent being physically active in the last 7 days. Please answer each question even if you do not consider yourself to be an active person. Please think about the activities you do at work, as part of your house and yard work, to get from place to place, and in your spare time for recreation, exercise or sport.<br><br>Think about all the vigorous and moderate activities that you did in the last 7 days. Vigorous physical activities refer to activities that take hard physical effort and make you breathe much harder than normal. Moderate activities refer to activities that take moderate physical effort and make you breathe somewhat harder than normal.<br><br>PART 1: JOB-RELATED PHYSICAL ACTIVITY<br>The first section is about your work. This includes paid jobs, farming, volunteer work, course work, and any other unpaid work that you did outside your home. Do not include unpaid	yes/no	Do you currently have a job or do any unpaid work outside your home?	

		work you might do around your home like housework, yard work, general maintenance, and caring for your family (these are asked about in Part 3).			
**lcat_2**	day_1_postbreakfast_questionnaire		dropdown	During the last 7 days, on how many days did you do vigorous physical activities like heavy lifting, digging, heavy construction, or climbing up stairs as part of your work? Think about only those physical activities that you did for at least 10 minutes at a time.	1, 1 Day | 2, 2 Days | 3, 3 Days | 4, 4 Days | 5, 5 Days | 6, 6 Days | 7, 7 Days
**lcat_2_no**	day_1_postbreakfast_questionnaire		checkbox		1, No vigorous job-related physical activity
**lcat_3_h**	day_1_postbreakfast_questionnaire		dropdown	How much time did you usually spend on one of those days doing vigorous physical activities as part of your work?	0, 0 | 1, 1 | 2, 2 | 3, 3 | 4, 4 | 5, 5 | 6, 6 | 7, 7 | 8, 8 | 9, 9 | 10, 10
**lcat_3_m**	day_1_postbreakfast_questionnaire		dropdown		0, 0 | 1, 1 | 2, 2 | 3, 3 | 4, 4 | 5, 5 | 6, 6 | 7, 7 | 8, 8 | 9, 9 | 10, 10 | 11, 11 | 12, 12 | 13, 13 | 14, 14 | 15, 15 | 16, 16 | 17, 17 | 18, 18 | 19, 19 | 20, 20 | 21, 21 | 22, 22 | 23, 23 | 24, 24 | 25, 25 | 26, 26 | 27, 27 | 28, 28 | 29, 29 | 30, 30 | 31, 31 | 32, 32 | 33, 33 | 34, 34 | 35, 35 | 36, 36 | 37, 37 | 38, 38 | 39, 39 | 40, 40 | 41, 41 | 42, 42 | 43, 43 | 44, 44 | 45, 45 | 46, 46 | 47, 47 | 48, 48 | 49, 49 | 50, 50 | 51, 51 | 52, 52 | 53, 53 | 54, 54 | 55, 55 | 56, 56 | 57, 57 | 58, 58 | 59, 59 | 60, 60

**lcat_4**	day_1_postbreakfast_questionnaire		dropdown	Again, think about only those physical activities that you did for at least 10 minutes at a time. During the last 7 days, on how many days did you do moderate physical activities like carrying light loads as part of your work? Please do not include walking.	1, 1 Day | 2, 2 Days | 3, 3 Days | 4, 4 Days | 5, 5 Days | 6, 6 Days | 7, 7 Days
**lcat_4_no**	day_1_postbreakfast_questionnaire		checkbox		1, No moderate job-related physical activity
**lcat_5_h**	day_1_postbreakfast_questionnaire		dropdown	How much time did you usually spend on one of those days doing moderate physical activities as part of your work?	0, 0 | 1, 1 | 2, 2 | 3, 3 | 4, 4 | 5, 5 | 6, 6 | 7, 7 | 8, 8 | 9, 9 | 10, 10
**lcat_5_m**	day_1_postbreakfast_questionnaire		dropdown		0,0 | 1, 1 | 2, 2 | 3, 3 | 4, 4 | 5, 5 | 6, 6 | 7, 7 | 8, 8 | 9, 9 | 10, 10 | 11, 11 | 12, 12 | 13, 13 | 14, 14 | 15, 15 | 16, 16 | 17, 17 | 18, 18 | 19, 19 | 20, 20 | 21, 21 | 22, 22 | 23, 23 | 24, 24 | 25, 25 | 26, 26 | 27, 27 | 28, 28 | 29, 29 | 30, 30 | 31, 31 | 32, 32 | 33, 33 | 34, 34 | 35, 35 | 36, 36 | 37, 37 | 38, 38 | 39, 39 | 40, 40 | 41, 41 | 42, 42 | 43, 43 | 44, 44 | 45, 45 | 46, 46 | 47, 47 | 48, 48 | 49, 49 | 50, 50 | 51, 51 | 52, 52 | 53, 53 | 54, 54 | 55, 55 | 56, 56 | 57, 57 | 58, 58 | 59, 59 | 60, 60
**lcat_6**	day_1_postbreakfast_questionnaire		dropdown	During the last 7 days, on how many days did you walk for at least 10 minutes at a time as part of your work? Please do not count any walking you did to travel to or from work.	1, 1 Day | 2, 2 Days | 3, 3 Days | 4, 4 Days | 5, 5 Days | 6, 6 Days | 7, 7 Days

**Icat_6_no**	day_1_postbreakfast_questionnaire		checkbox		1,No job-related walking
**Icat_7_h**	day_1_postbreakfast_questionnaire		dropdown	How much time did you usually spend on one of those days walkings as part of your work?	0, 0 | 1, 1 | 2, 2 | 3, 3 | 4, 4 | 5, 5 | 6, 6 | 7, 7 | 8, 8 | 9, 9 | 10, 10
**Icat_7_m**	day_1_postbreakfast_questionnaire		dropdown		0, 0 | 1, 1 | 2, 2 | 3, 3 | 4, 4 | 5, 5 | 6, 6 | 7, 7 | 8, 8 | 9, 9 | 10, 10 | 11, 11 | 12, 12 | 13, 13 | 14, 14 | 15, 15 | 16, 16 | 17, 17 | 18, 18 | 19, 19 | 20, 20 | 21, 21 | 22, 22 | 23, 23 | 24, 24 | 25, 25 | 26, 26 | 27, 27 | 28, 28 | 29, 29 | 30, 30 | 31, 31 | 32, 32 | 33, 33 | 34, 34 | 35, 35 | 36, 36 | 37, 37 | 38, 38 | 39, 39 | 40, 40 | 41, 41 | 42, 42 | 43, 43 | 44, 44 | 45, 45 | 46, 46 | 47, 47 | 48, 48 | 49, 49 | 50, 50 | 51, 51 | 52, 52 | 53, 53 | 54, 54 | 55, 55 | 56, 56 | 57, 57 | 58, 58 | 59, 59 | 60, 60
**a**	day_1_postbreakfast_questionnaire	PART 2: TRANSPORTATION PHYSICAL ACTIVITY<br>These questions are about how you traveled from place to place, including to places like work, stores, movies, and so on.	dropdown	During the last 7 days, on how many days did you travel in a motor vehicle like a train, bus, car, or tram?	1, 1 Day | 2, 2 Days | 3, 3 Days | 4, 4 Days | 5, 5 Days | 6, 6 Days | 7, 7 Days
**Icat_8_no**	day_1_postbreakfast_questionnaire		checkbox		1, No traveling in a motor vehicle
**Icat_9**	day_1_postbreakfast_questionnaire		dropdown	How much time did you usually spend on one of those days traveling in a train, bus, car, tram, or other kind of motor vehicle?	0, 0 | 1, 1 | 2, 2 | 3, 3 | 4, 4 | 5, 5 | 6, 6 | 7, 7 | 8, 8 | 9, 9 | 10, 10

**lcat_9_m**	day_1_postbreakfast_questionnaire		dropdown		0, 0 | 1, 1 | 2, 2 | 3, 3 | 4, 4 | 5, 5 | 6, 6 | 7, 7 | 8, 8 | 9, 9 | 10, 10 | 11, 11 | 12, 12 | 13, 13 | 14, 14 | 15, 15 | 16, 16 | 17, 17 | 18, 18 | 19, 19 | 20, 20 | 21, 21 | 22, 22 | 23, 23 | 24, 24 | 25, 25 | 26, 26 | 27, 27 | 28, 28 | 29, 29 | 30, 30 | 31, 31 | 32, 32 | 33, 33 | 34, 34 | 35, 35 | 36, 36 | 37, 37 | 38, 38 | 39, 39 | 40, 40 | 41, 41 | 42, 42 | 43, 43 | 44, 44 | 45, 45 | 46, 46 | 47, 47 | 48, 48 | 49, 49 | 50, 50 | 51, 51 | 52, 52 | 53, 53 | 54, 54 | 55, 55 | 56, 56 | 57, 57 | 58, 58 | 59, 59 | 60, 60
**lcat_10**	day_1_postbreakfast_questionnaire	Now think only about the bicycling and walking you might have done to travel to and from work, to do errands, or to go from place to place.	dropdown	During the last 7 days, on how many days did you bicycle for at least 10 minutes at a time to go from place to place?	1, 1 Day | 2, 2 Days | 3, 3 Days | 4, 4 Days | 5, 5 Days | 6, 6 Days | 7, 7 Days
**lcat_10_no**	day_1_postbreakfast_questionnaire		checkbox		1, No bicycling from place to place
**lcat_11**	day_1_postbreakfast_questionnaire		dropdown	How much time did you usually spend on one of those days to bicycle from place to place?	0, 0 | 1, 1 | 2, 2 | 3, 3 | 4, 4 | 5, 5 | 6, 6 | 7, 7 | 8, 8 | 9, 9 | 10, 10
**lcat_11_m**	day_1_postbreakfast_questionnaire		dropdown		0, 0 | 1, 1 | 2, 2 | 3, 3 | 4, 4 | 5, 5 | 6, 6 | 7, 7 | 8, 8 | 9, 9 | 10, 10 | 11, 11 | 12, 12 | 13, 13 | 14, 14 | 15, 15 | 16, 16 | 17, 17 | 18, 18 | 19, 19 | 20, 20 | 21, 21 | 22, 22 | 23, 23 | 24, 24 | 25, 25 | 26, 26 | 27, 27 | 28, 28 | 29, 29 | 30, 30 | 31, 31 | 32, 32 | 33, 33 | 34,

					34 | 35, 35 | 36, 36 | 37, 37 | 38, 38 | 39, 39 | 40, 40 | 41, 41 | 42, 42 | 43, 43 | 44, 44 | 45, 45 | 46, 46 | 47, 47 | 48, 48 | 49, 49 | 50, 50 | 51, 51 | 52, 52 | 53, 53 | 54, 54 | 55, 55 | 56, 56 | 57, 57 | 58, 58 | 59, 59 | 60, 60
**lcat_12**	day_1_postbreakfast_questionnaire		dropdown	During the last 7 days, on how many days did you walk for at least 10 minutes at a time to go from place to place?	1, 1 Day | 2, 2 Days | 3, 3 Days | 4, 4 Days | 5, 5 Days | 6, 6 Days | 7, 7 Days
**lcat_12_no**	day_1_postbreakfast_questionnaire		checkbox		1, No walking from place to place
**lcat_13**	day_1_postbreakfast_questionnaire		dropdown	How much time did you usually spend on one of those days walking from place to place?	0, 0 | 1, 1 | 2, 2 | 3, 3 | 4, 4 | 5, 5 | 6, 6 | 7, 7 | 8, 8 | 9, 9 | 10, 10
**lcat_13_m**	day_1_postbreakfast_questionnaire		dropdown		0, 0 | 1, 1 | 2, 2 | 3, 3 | 4, 4 | 5, 5 | 6, 6 | 7, 7 | 8, 8 | 9, 9 | 10, 10 | 11, 11 | 12, 12 | 13, 13 | 14, 14 | 15, 15 | 16, 16 | 17, 17 | 18, 18 | 19, 19 | 20, 20 | 21, 21 | 22, 22 | 23, 23 | 24, 24 | 25, 25 | 26, 26 | 27, 27 | 28, 28 | 29, 29 | 30, 30 | 31, 31 | 32, 32 | 33, 33 | 34, 34 | 35, 35 | 36, 36 | 37, 37 | 38, 38 | 39, 39 | 40, 40 | 41, 41 | 42, 42 | 43, 43 | 44, 44 | 45, 45 | 46, 46 | 47, 47 | 48, 48 | 49, 49 | 50, 50 | 51, 51 | 52, 52 | 53, 53 | 54, 54 | 55, 55 | 56, 56 | 57, 57 | 58, 58 | 59, 59 | 60, 60

**lcat_14**	day_1_postbreakfast_questionnaire	PART 3: HOUSEWORK, HOUSE MAINTENANCE, AND CARING FOR FAMILY<br>This section is about some of the physical activities you might have done in the last 7 days in and around your home, such as housework, gardening, yard work, general maintenance work, and caring for your family.	dropdown	Think about only those physical activities that you did for at least 10 minutes at a time. During the last 7 days, on how many days did you do vigorous physical activities like heavy lifting, chopping wood, shoveling snow, or digging in the garden or yard?	1, 1 Day | 2, 2 Days | 3, 3 Days | 4, 4 Days | 5, 5 Days | 6, 6 Days | 7, 7 Days
**lcat_14_no**	day_1_postbreakfast_questionnaire		checkbox		1, No vigorous activity in garden or yard
**lcat_15**	day_1_postbreakfast_questionnaire		dropdown	How much time did you usually spend on one of those days doing vigorous physical activities in the garden or yard?	0, 0 | 1, 1 | 2, 2 | 3, 3 | 4, 4 | 5, 5 | 6, 6 | 7, 7 | 8, 8 | 9, 9 | 10, 10
**lcat_15_m**	day_1_postbreakfast_questionnaire		dropdown		0, 0 | 1, 1 | 2, 2 | 3, 3 | 4, 4 | 5, 5 | 6, 6 | 7, 7 | 8, 8 | 9, 9 | 10, 10 | 11, 11 | 12, 12 | 13, 13 | 14, 14 | 15, 15 | 16, 16 | 17, 17 | 18, 18 | 19, 19 | 20, 20 | 21, 21 | 22, 22 | 23, 23 | 24, 24 | 25, 25 | 26, 26 | 27, 27 | 28, 28 | 29, 29 | 30, 30 | 31, 31 | 32, 32 | 33, 33 | 34, 34 | 35, 35 | 36, 36 | 37, 37 | 38, 38 | 39, 39 | 40, 40 | 41, 41 | 42, 42 | 43, 43 | 44, 44 | 45, 45 | 46, 46 | 47, 47 | 48, 48 | 49, 49 | 50, 50 | 51, 51 | 52, 52 | 53, 53 | 54, 54 | 55, 55 | 56, 56 | 57, 57 | 58, 58 | 59, 59 | 60, 60
**lcat_16**	day_1_postbreakfast_questionnaire		dropdown	Again, think about only those physical activities that you did for at least 10 minutes at a time. During the last 7 days, on how many days	1, 1 Day | 2, 2 Days | 3, 3 Days | 4, 4 Days | 5, 5 Days | 6, 6 Days | 7, 7 Days

				did you do moderate activities like carrying light loads, sweeping, washing windows, and raking in the garden or yard?	
**lcat_16_no**	day_1_postbreakfast_questionnaire		checkbox		1, No moderate activity in garden or yard
**lcat_17**	day_1_postbreakfast_questionnaire		dropdown	How much time did you usually spend on one of those days doing moderate physical activities in the garden or yard?	0, 0 | 1, 1 | 2, 2 | 3, 3 | 4, 4 | 5, 5 | 6, 6 | 7, 7 | 8, 8 | 9, 9 | 10, 10
**lcat_17_m**	day_1_postbreakfast_questionnaire		dropdown		0, 0 | 1, 1 | 2, 2 | 3, 3 | 4, 4 | 5, 5 | 6, 6 | 7, 7 | 8, 8 | 9, 9 | 10, 10 | 11, 11 | 12, 12 | 13, 13 | 14, 14 | 15, 15 | 16, 16 | 17, 17 | 18, 18 | 19, 19 | 20, 20 | 21, 21 | 22, 22 | 23, 23 | 24, 24 | 25, 25 | 26, 26 | 27, 27 | 28, 28 | 29, 29 | 30, 30 | 31, 31 | 32, 32 | 33, 33 | 34, 34 | 35, 35 | 36, 36 | 37, 37 | 38, 38 | 39, 39 | 40, 40 | 41, 41 | 42, 42 | 43, 43 | 44, 44 | 45, 45 | 46, 46 | 47, 47 | 48, 48 | 49, 49 | 50, 50 | 51, 51 | 52, 52 | 53, 53 | 54, 54 | 55, 55 | 56, 56 | 57, 57 | 58, 58 | 59, 59 | 60, 60
**lcat_18**	day_1_postbreakfast_questionnaire		dropdown	Once again, think about only those physical activities that you did for at least 10 minutes at a time. During the last 7 days, on how many days did you do moderate activities like carrying light loads, washing windows, scrubbing floors, and sweeping inside your home?	1, 1 Day | 2, 2 Days | 3, 3 Days | 4, 4 Days | 5, 5 Days | 6, 6 Days | 7, 7 Days

**lcat_18_no**	day_1_postbreakfast_questionnaire		checkbox		1, No moderate activity inside home
**lcat_19**	day_1_postbreakfast_questionnaire		dropdown	How much time did you usually spend on one of those days doing moderate physical activities inside your home?	0, 0 | 1, 1 | 2, 2 | 3, 3 | 4, 4 | 5, 5 | 6, 6 | 7, 7 | 8, 8 | 9, 9 | 10, 10
**lcat_19_m**	day_1_postbreakfast_questionnaire		dropdown		0, 0 | 1, 1 | 2, 2 | 3, 3 | 4, 4 | 5, 5 | 6, 6 | 7, 7 | 8, 8 | 9, 9 | 10, 10 | 11, 11 | 12, 12 | 13, 13 | 14, 14 | 15, 15 | 16, 16 | 17, 17 | 18, 18 | 19, 19 | 20, 20 | 21, 21 | 22, 22 | 23, 23 | 24, 24 | 25, 25 | 26, 26 | 27, 27 | 28, 28 | 29, 29 | 30, 30 | 31, 31 | 32, 32 | 33, 33 | 34, 34 | 35, 35 | 36, 36 | 37, 37 | 38, 38 | 39, 39 | 40, 40 | 41, 41 | 42, 42 | 43, 43 | 44, 44 | 45, 45 | 46, 46 | 47, 47 | 48, 48 | 49, 49 | 50, 50 | 51, 51 | 52, 52 | 53, 53 | 54, 54 | 55, 55 | 56, 56 | 57, 57 | 58, 58 | 59, 59 | 60, 60
**lcat_20**	day_1_postbreakfast_questionnaire	PART 4: RECREATION, SPORT, AND LEISURE-TIME PHYSICAL ACTIVITY<br>This section is about all the physical activities that you did in the last 7 days solely for recreation, sport, exercise, or leisure. Please do not include any activities you have already mentioned.	dropdown	Not counting any walking you have already mentioned, during the last 7 days, on how many days did you walk for at least 10 minutes at a time in your leisure time?	1, 1 Day | 2, 2 Days | 3, 3 Days | 4, 4 Days | 5, 5 Days | 6, 6 Days | 7, 7 Days
**lcat_20_no**	day_1_postbreakfast_questionnaire		checkbox		1, No walking in leisure time

**lcat_21**	day_1_postbreakfast_questionnaire		dropdown	How much time did you usually spend on one of those days walking in your leisure time?	0, 0 | 1, 1 | 2, 2 | 3, 3 | 4, 4 | 5, 5 | 6, 6 | 7, 7 | 8, 8 | 9, 9 | 10, 10
**lcat_21_m**	day_1_postbreakfast_questionnaire		dropdown		0, 0 | 1, 1 | 2, 2 | 3, 3 | 4, 4 | 5, 5 | 6, 6 | 7, 7 | 8, 8 | 9, 9 | 10, 10 | 11, 11 | 12, 12 | 13, 13 | 14, 14 | 15, 15 | 16, 16 | 17, 17 | 18, 18 | 19, 19 | 20, 20 | 21, 21 | 22, 22 | 23, 23 | 24, 24 | 25, 25 | 26, 26 | 27, 27 | 28, 28 | 29, 29 | 30, 30 | 31, 31 | 32, 32 | 33, 33 | 34, 34 | 35, 35 | 36, 36 | 37, 37 | 38, 38 | 39, 39 | 40, 40 | 41, 41 | 42, 42 | 43, 43 | 44, 44 | 45, 45 | 46, 46 | 47, 47 | 48, 48 | 49, 49 | 50, 50 | 51, 51 | 52, 52 | 53, 53 | 54, 54 | 55, 55 | 56, 56 | 57, 57 | 58, 58 | 59, 59 | 60, 60
**lcat_22**	day_1_postbreakfast_questionnaire		dropdown	Think about only those physical activities that you did for at least 10 minutes at a time. During the last 7 days, on how many days did you do vigorous physical activities like aerobics, running, fast bicycling, or fast swimming in your leisure time?	1, 1 Day | 2, 2 Days | 3, 3 Days | 4, 4 Days | 5, 5 Days | 6, 6 Days | 7, 7 Days
**lcat_22_no**	day_1_postbreakfast_questionnaire		checkbox		1, No vigorous activity in leisure time
**lcat_23**	day_1_postbreakfast_questionnaire		dropdown	How much time did you usually spend on one of those days doing vigorous physical activities in your leisure time?	0, 0 | 1, 1 | 2, 2 | 3, 3 | 4, 4 | 5, 5 | 6, 6 | 7, 7 | 8, 8 | 9, 9 | 10, 10
**lcat_23_m**	day_1_postbreakfast_questionnaire		dropdown		0, 0 | 1, 1 | 2, 2 | 3, 3 | 4, 4 | 5, 5 | 6, 6 | 7, 7 | 8, 8 | 9, 9 | 10, 10 | 11, 11 | 12, 12 | 13,

					13 | 14, 14 | 15, 15 | 16, 16 | 17, 17 | 18, 18 | 19, 19 | 20, 20 | 21, 21 | 22, 22 | 23, 23 | 24, 24 | 25, 25 | 26, 26 | 27, 27 | 28, 28 | 29, 29 | 30, 30 | 31, 31 | 32, 32 | 33, 33 | 34, 34 | 35, 35 | 36, 36 | 37, 37 | 38, 38 | 39, 39 | 40, 40 | 41, 41 | 42, 42 | 43, 43 | 44, 44 | 45, 45 | 46, 46 | 47, 47 | 48, 48 | 49, 49 | 50, 50 | 51, 51 | 52, 52 | 53, 53 | 54, 54 | 55, 55 | 56, 56 | 57, 57 | 58, 58 | 59, 59 | 60, 60
**lcat_24**	day_1_postbreakfast_questionnaire		dropdown	Again, think about only those physical activities that you did for at least 10 minutes at a time. During the last 7 days, on how many days did you do moderate physical activities like bicycling at a regular pace, swimming at a regular pace, and doubles tennis in your leisure time?	1, 1 Day | 2, 2 Days | 3, 3 Days | 4, 4 Days | 5, 5 Days | 6, 6 Days | 7, 7 Days
**lcat_24_no**	day_1_postbreakfast_questionnaire		checkbox		1, No moderate activity in leisure time
**lcat_25**	day_1_postbreakfast_questionnaire		dropdown	How much time did you usually spend on one of those days doing moderate physical activities in your leisure time?	0, 0 | 1, 1 | 2, 2 | 3, 3 | 4, 4 | 5, 5 | 6, 6 | 7, 7 | 8, 8 | 9, 9 | 10, 10
**lcat_25_m**	day_1_postbreakfast_questionnaire		dropdown		0, 0 | 1, 1 | 2, 2 | 3, 3 | 4, 4 | 5, 5 | 6, 6 | 7, 7 | 8, 8 | 9, 9 | 10, 10 | 11, 11 | 12, 12 | 13, 13 | 14, 14 | 15, 15 | 16, 16 | 17, 17 | 18, 18 | 19, 19 | 20, 20 | 21, 21 | 22, 22 | 23, 23 | 24, 24 | 25, 25 | 26, 26 | 27, 27 | 28, 28 | 29, 29 | 30, 30 | 31, 31 | 32, 32 | 33, 33 | 34,

					34 | 35, 35 | 36, 36 | 37, 37 | 38, 38 | 39, 39 | 40, 40 | 41, 41 | 42, 42 | 43, 43 | 44, 44 | 45, 45 | 46, 46 | 47, 47 | 48, 48 | 49, 49 | 50, 50 | 51, 51 | 52, 52 | 53, 53 | 54, 54 | 55, 55 | 56, 56 | 57, 57 | 58, 58 | 59, 59 | 60, 60
**lcat_26**	day_1_postbreakfast_questionnaire	PART 5: TIME SPENT SITTING<br>The last questions are about the time you spend sitting while at work, at home, while doing course work, and during leisure time. This may include time spent sitting at a desk, visiting friends, reading, or sitting or lying down to watch television. Do not include any time spent sitting in a motor vehicle that you have already told me about.	dropdown	During the last 7 days, how much time did you usually spend sitting on a weekday?	0, 0 | 1, 1 | 2, 2 | 3, 3 | 4, 4 | 5, 5 | 6, 6 | 7, 7 | 8, 8 | 9, 9 | 10, 10
**lcat_26_m**	day_1_postbreakfast_questionnaire		dropdown		0, 0 | 1, 1 | 2, 2 | 3, 3 | 4, 4 | 5, 5 | 6, 6 | 7, 7 | 8, 8 | 9, 9 | 10, 10 | 11, 11 | 12, 12 | 13, 13 | 14, 14 | 15, 15 | 16, 16 | 17, 17 | 18, 18 | 19, 19 | 20, 20 | 21, 21 | 22, 22 | 23, 23 | 24, 24 | 25, 25 | 26, 26 | 27, 27 | 28, 28 | 29, 29 | 30, 30 | 31, 31 | 32, 32 | 33, 33 | 34, 34 | 35, 35 | 36, 36 | 37, 37 | 38, 38 | 39, 39 | 40, 40 | 41, 41 | 42, 42 | 43, 43 | 44, 44 | 45, 45 | 46, 46 | 47, 47 | 48, 48 | 49, 49 | 50, 50 | 51, 51 | 52, 52 | 53, 53 | 54, 54 | 55, 55 | 56, 56 | 57, 57 | 58, 58 | 59, 59 | 60, 60

**lcat_27**	day_1_postbreakfast_questionnaire		dropdown	During the last 7 days, how much time did you usually spend sitting on a weekend day?	0, 0 | 1, 1 | 2, 2 | 3, 3 | 4, 4 | 5, 5 | 6, 6 | 7, 7 | 8, 8 | 9, 9 | 10, 10
**lcat_27_m**	day_1_postbreakfast_questionnaire		dropdown		0, 0 | 1, 1 | 2, 2 | 3, 3 | 4, 4 | 5, 5 | 6, 6 | 7, 7 | 8, 8 | 9, 9 | 10, 10| 11, 11 | 12, 12 | 13, 13 | 14, 14 | 15, 15 | 16, 16 | 17, 17 | 18, 18 | 19, 19 | 20, 20| 21, 21 | 22, 22 | 23, 23 | 24, 24 | 25, 25 | 26, 26 | 27, 27 | 28, 28 | 29, 29 | 30, 30| 31, 31 | 32, 32 | 33, 33 | 34, 34 | 35, 35 | 36, 36 | 37, 37 | 38, 38 | 39, 39 | 40, 40| 41, 41 | 42, 42 | 43, 43 | 44, 44 | 45, 45 | 46, 46 | 47, 47 | 48, 48 | 49, 49 | 50, 50| 51, 51 | 52, 52 | 53, 53 | 54, 54 | 55, 55 | 56, 56 | 57, 57 | 58, 58 | 59, 59 | 60, 60
**sqffs_mince_d1**	day_1_postbreakfast_questionnaire	Food<br>Instructions: Think about the food you've eaten over the past year. Remember breakfast, lunch, dinner, and eating out. Please select the option that best describes how often you have consumed each of the following food or drink items.	radio	1. Mince, beef, or lamb, such as in hamburgers, nachos, or bolognaise	0, Never | 1, Less than 1 per month | 2, 2-3 per month | 3, 1-2 per week | 4, 3-4 per week | 5, 5+ per week
**sqffs_beef_d1**	day_1_postbreakfast_questionnaire		radio	2. Beef or pork, such as steak, ribs, roasts, or in sandwiches	0, Never | 1, Less than 1 per month | 2, 2-3 per month | 3, 1-2 per week | 4, 3-4 per week | 5, 5+ per week
**sqffs_friedchicken_d1**	day_1_postbreakfast_questionnaire		radio	3. Fried chicken or chicken burgers	0, Never | 1, Less than 1 per month | 2, 2-3 per month | 3, 1-2 per week | 4,

					3-4 per week | 5, 5+ per week
**sqffs_sausages_d1**	day_1_postbreakfast_questionnaire		radio	4. Sausages, frankfurts, or salami	0, Never | 1, Less than 1 per month | 2, 2-3 per month | 3, 1-2 per week | 4, 3-4 per week | 5, 5+ per week
**sqffs_bacon_d1**	day_1_postbreakfast_questionnaire		radio	5. Bacon	0, Never | 1, Less than 1 per month | 2, 2-3 per month | 3, 1-2 per week | 4, 3-4 per week | 5, 5+ per week
**sqffs_saladdressing_d1**	day_1_postbreakfast_questionnaire		radio	6. Salad dressings (not low fat)	0, Never | 1, Less than 1 per month | 2, 2-3 per month | 3, 1-2 per week | 4, 3-4 per week | 5, 5+ per week
**sqffs_margarine_d1**	day_1_postbreakfast_questionnaire		radio	7. Margarine, butter, or oil in cooking	0, Never | 1, Less than 1 per month | 2, 2-3 per month | 3, 1-2 per week | 4, 3-4 per week | 5, 5+ per week
**sqffs_eggs_d1**	day_1_postbreakfast_questionnaire		radio	8. Eggs (not egg whites alone)	0, Never | 1, Less than 1 per month | 2, 2-3 per month | 3, 1-2 per week | 4, 3-4 per week | 5, 5+ per week
**sqffs_pizza_d1**	day_1_postbreakfast_questionnaire		radio	9. Pizza	0, Never | 1, Less than 1 per month | 2, 2-3 per month | 3, 1-2 per week | 4, 3-4 per week | 5, 5+ per week

**sqffsz_cheese_d1**	day_1_postbreakfast_questionnaire		radio	10. Cheese or cheese spread (not low fat)	0, Never | 1, Less than 1 per month | 2, 2-3 per month | 3, 1-2 per week | 4, 3-4 per week | 5, 5+ per week
**sqffs_frenchfries_d1**	day_1_postbreakfast_questionnaire		radio	11. French fries or fried potatoes	0, Never | 1, Less than 1 per month | 2, 2-3 per month | 3, 1-2 per week | 4, 3-4 per week | 5, 5+ per week
**sqffs_cornchips_d1**	day_1_postbreakfast_questionnaire		radio	12. Corn chips, potato chips, or popcorn with butter	0, Never | 1, Less than 1 per month | 2, 2-3 per month | 3, 1-2 per week | 4, 3-4 per week | 5, 5+ per week
**sqffs_doughnuts_d1**	day_1_postbreakfast_questionnaire		radio	13. Doughnuts, pastries, or croissants	0, Never | 1, Less than 1 per month | 2, 2-3 per month | 3, 1-2 per week | 4, 3-4 per week | 5, 5+ per week
**sqffs_cakes_d1**	day_1_postbreakfast_questionnaire		radio	14. Cakes or cookies	0, Never | 1, Less than 1 per month | 2, 2-3 per month | 3, 1-2 per week | 4, 3-4 per week | 5, 5+ per week
**sqffs_icecream_d1**	day_1_postbreakfast_questionnaire		radio	15. Ice cream (not sorbet or low fat)	0, Never | 1, Less than 1 per month | 2, 2-3 per month | 3, 1-2 per week | 4, 3-4 per week | 5, 5+ per week
**sqffs_chocolate_d1**	day_1_postbreakfast_questionnaire		radio	16. Chocolate	0, Never | 1, Less than 1 per month | 2, 2-3 per month | 3, 1-2 per week | 4,

					3-4 per week | 5, 5+ per week
**sqffs_lollies_d1**	day_1_postbreakfast_questionnaire		radio	17. Lollies	0, Never | 1, Less than 1 per month | 2, 2-3 per month | 3, 1-2 per week | 4, 3-4 per week | 5, 5+ per week
**sqffs_spreads_d1**	day_1_postbreakfast_questionnaire		radio	18. Spreads, including peanut butter, jam, or honey	0, Never | 1, Less than 1 per month | 2, 2-3 per month | 3, 1-2 per week | 4, 3-4 per week | 5, 5+ per week
**sqffs_pancakes_d1**	day_1_postbreakfast_questionnaire		radio	19. Pancakes or French toast	0, Never | 1, Less than 1 per month | 2, 2-3 per month | 3, 1-2 per week | 4, 3-4 per week | 5, 5+ per week
**sqffs_sportsdrinks_d1**	day_1_postbreakfast_questionnaire		radio	20. Sports drinks, such as Gatorade, or energy drinks, such as Red Bull	0, Never | 1, Less than 1 per month | 2, 2-3 per month | 3, 1-2 per week | 4, 3-4 per week | 5, 5+ per week
**sqffs_softdrink_d1**	day_1_postbreakfast_questionnaire		radio	21. Soft drink (not including diet soda)	0, Never | 1, Less than 1 per month | 2, 2-3 per month | 3, 1-2 per week | 4, 3-4 per week | 5, 5+ per week
**sqffs_milk_d1**	day_1_postbreakfast_questionnaire		radio	22. Milk (full fat only). Include milk drunk by itself or in cappuccinos, milkshakes, hot chocolates, etc.	0, Never | 1, Less than 1 per month | 2, 2-3 per month | 3, 1-2 per week | 4, 3-4 per week | 5, 5+ per week

**sqffs_sweeteneddrink_d1**	day_1_postbreakfast_questionnaire		radio	23. Other sweetened beverages, such as juice with added sugar, cordial, or sweetened teas	0, Never | 1, Less than 1 per month | 2, 2-3 per month | 3, 1-2 per week | 4, 3-4 per week | 5, 5+ per week
**sqffs_whitebread_d1**	day_1_postbreakfast_questionnaire		radio	24. White bread (white bread only)	0, Never | 1, Less than 1 per month | 2, 2-3 per month | 3, 1-2 per week | 4, 3-4 per week | 5, 5+ per week
**sqffs_fastfood_d1**	day_1_postbreakfast_questionnaire		radio	25. In the past year, how many times have you eaten food from a takeaway or fast food restaurant, such as McDonald's, KFC, Mexican, Chinese, Thai, or Italian (pizza or pasta)?	0, Never | 1, Less than 1 per month | 2, 2-3 per month | 3, 1-2 per week | 4, 3-4 per week | 5, 5+ per week
**sqffs_addedsugar_d1**	day_1_postbreakfast_questionnaire		radio	26. In the past week, how many teaspoons of sugar have you added to your beverages, cereal, or food?	0, None | 1, 1-2 | 2, 3-4 | 3, 5-6 | 4, 7+
**ffi_fishprotein_d1**	day_1_postbreakfast_questionnaire	What I Eat<br>Over the past 12 months, how often did you eat each of the following foods?<br><br>Protein Sources<br>Please choose one of the following answers for each question:	radio	Fish (char, herring, salmon, tuna)	0, Never | 1, Daily | 2, Weekly | 3, Monthly
**ffi_shellfishprotein_d1**	day_1_postbreakfast_questionnaire		radio	Shellfish (clams, oysters, shrimp, lobster)	0, Never | 1, Daily | 2, Weekly | 3, Monthly
**ffi_poultryprotein_d1**	day_1_postbreakfast_questionnaire		radio	Poultry (chicken)	0, Never | 1, Daily | 2, Weekly | 3, Monthly

**ffi_leanmeatprotein_d1**	day_1_postbreakfast_questionnaire		radio	Lean meat (ham, beef, veal)	0, Never | 1, Daily | 2, Weekly | 3, Monthly
**ffi_tofuprotein_d1**	day_1_postbreakfast_questionnaire		radio	Tofu, soy burgers, or soy-meat substitute (e.g., veggie burgers)	0, Never | 1, Daily | 2, Weekly | 3, Monthly
**ffi_legumeprotein_d1**	day_1_postbreakfast_questionnaire		radio	Cooked legumes (pinto, kidney, black-eyed peas, lentils, baked beans)	0, Never | 1, Daily | 2, Weekly | 3, Monthly
**ffi_eggprotein_d1**	day_1_postbreakfast_questionnaire		radio	Eggs	0, Never | 1, Daily | 2, Weekly | 3, Monthly
**ffi_peanutbutterprotein_d1**	day_1_postbreakfast_questionnaire		radio	Peanut butter, other nut butter	0, Never | 1, Daily | 2, Weekly | 3, Monthly
**ffi_nutprotein_d1**	day_1_postbreakfast_questionnaire		radio	Nuts (peanuts, walnuts, seeds, cashews, almonds)	0, Never | 1, Daily | 2, Weekly | 3, Monthly
**ffi_wheatbreadstarch_d1**	day_1_postbreakfast_questionnaire	Starches<br>Please choose one of the following answers for each question:	radio	White bread	0, Never | 1, Daily | 2, Weekly | 3, Monthly
**ffi_pastastarch_d1**	day_1_postbreakfast_questionnaire		radio	Whole wheat bread or rye	0, Never | 1, Daily | 2, Weekly | 3, Monthly
**ffi_ricestarch_d1**	day_1_postbreakfast_questionnaire		radio	Pasta (e.g. spaghetti) or couscous	0, Never | 1, Daily | 2, Weekly | 3, Monthly
**ffi_bagelstarch_d1**	day_1_postbreakfast_questionnaire		radio	Rice or other cooked grains (bulgar quinoa)	0, Never | 1, Daily | 2, Weekly | 3, Monthly
**ffi_cerealstarch_d1**	day_1_postbreakfast_questionnaire		radio	Bagels	0, Never | 1, Daily | 2, Weekly | 3, Monthly

**ffi_flatbreadstarch_d1**	day_1_postbreakfast_questionnaire		radio	Cereal (oatmeal, cold cereal)	0, Never | 1, Daily | 2, Weekly | 3, Monthly
**ffi_whitebreadstarch_d1**	day_1_postbreakfast_questionnaire		radio	Flat breads (pitas, tortillas)	0, Never | 1, Daily | 2, Weekly | 3, Monthly
**ffi_cheesedairy_d1**	day_1_postbreakfast_questionnaire	Dairy Products<br>Please choose one of the following answers for each question:	radio	Cheese	0, Never | 1, Daily | 2, Weekly | 3, Monthly
**ffi_yogurtdairy_d1**	day_1_postbreakfast_questionnaire		radio	Yogurt (not frozen yogurt) or Kefir	0, Never | 1, Daily | 2, Weekly | 3, Monthly
**ffi_fruit_d1**	day_1_postbreakfast_questionnaire	Fruits and Vegetables<br>Please choose one of the following answers for each question:	radio	Fresh/Raw fruit (e.g. oranges, strawberries, bananas)	0, Never | 1, Daily | 2, Weekly | 3, Monthly
**ffi_cannedfruit_d1**	day_1_postbreakfast_questionnaire		radio	Canned fruit	0, Never | 1, Daily | 2, Weekly | 3, Monthly
**ffi_frozenfruit_d1**	day_1_postbreakfast_questionnaire		radio	Frozen fruit	0, Never | 1, Daily | 2, Weekly | 3, Monthly
**ffi_leafyveg_d1**	day_1_postbreakfast_questionnaire		radio	Leafy vegetables/Green salad (e.g. romaine lettuce, spinach, kale)	0, Never | 1, Daily | 2, Weekly | 3, Monthly
**ffi_freshveg_d1**	day_1_postbreakfast_questionnaire		radio	Fresh/raw Vegetables (e.g. broccoli, carrots, squash)	0, Never | 1, Daily | 2, Weekly | 3, Monthly
**ffi_cannedveg_d1**	day_1_postbreakfast_questionnaire		radio	Canned vegetables	0, Never | 1, Daily | 2, Weekly | 3, Monthly
**ffi_frozenveg_d1**	day_1_postbreakfast_questionnaire		radio	Frozen vegetables	0, Never | 1, Daily | 2, Weekly | 3, Monthly

**ffi_pizzacombo_d1**	day_1_postbreakfast_questionnaire	Combination Foods<br>Please choose one of the following answers for each question:	radio	Pizza	0, Never | 1, Daily | 2, Weekly | 3, Monthly
**ffi_frenchfriescombo_d1**	day_1_postbreakfast_questionnaire		radio	French fries, hash browns, home fries, or poutine	0, Never | 1, Daily | 2, Weekly | 3, Monthly
**ffi_frozenfoodcombo_d1**	day_1_postbreakfast_questionnaire		radio	Frozen food meal	0, Never | 1, Daily | 2, Weekly | 3, Monthly
**ffi_soupcombo_d1**	day_1_postbreakfast_questionnaire		radio	Soup (broth based)	0, Never | 1, Daily | 2, Weekly | 3, Monthly
**ffi_potatochipcombo_d1**	day_1_postbreakfast_questionnaire	Fats, Oils, and Sweets<br>Please choose one of the following answers for each question:	radio	Potato chips, tortilla chips, or corn chips	0, Never | 1, Daily | 2, Weekly | 3, Monthly
**ffi_cookiesfats_d1**	day_1_postbreakfast_questionnaire		radio	Cookies or brownies	0, Never | 1, Daily | 2, Weekly | 3, Monthly
**ffi_mayonnaisefats_d1**	day_1_postbreakfast_questionnaire		radio	Mayonnaise (regular)	0, Never | 1, Daily | 2, Weekly | 3, Monthly
**ffi_buttersidefats_d1**	day_1_postbreakfast_questionnaire		radio	Butter or margarine (on bread or as condiment)	0, Never | 1, Daily | 2, Weekly | 3, Monthly
**ffi_buttercookingfats_d1**	day_1_postbreakfast_questionnaire		radio	Butter, margarine, or oil (in cooking)	0, Never | 1, Daily | 2, Weekly | 3, Monthly
**ffi_darkchocolatefats_d1**	day_1_postbreakfast_questionnaire		radio	Dark chocolate	0, Never | 1, Daily | 2, Weekly | 3, Monthly
**ffi_croissantfats_d1**	day_1_postbreakfast_questionnaire		radio	Croissant, brioche, donuts, cakes, or pastries	0, Never | 1, Daily | 2, Weekly | 3, Monthly

**ffi_fruitjuicebev_d1**	day_1_postbreakfast_questionnaire	Beverages<br>Please choose one of the following answers for each question:	radio	100% fruit juice (orange juice, grapefruit juice, tomato juice)	0, Never | 1, Daily | 2, Weekly | 3, Monthly
**ffi_fruitdrink_bev_d1**	day_1_postbreakfast_questionnaire		radio	Fruit drinks (Hi-C, Kool-Aid, cranberry cocktail)	0, Never | 1, Daily | 2, Weekly | 3, Monthly
**ffi_milkbev_d1**	day_1_postbreakfast_questionnaire		radio	Glass of milk (skim, 1%, 2%, 3.25%) (not in cereal, not in coffee)	0, Never | 1, Daily | 2, Weekly | 3, Monthly
**ffi_cannedmilkbev_d1**	day_1_postbreakfast_questionnaire		radio	Canned milk (evaporated)	0, Never | 1, Daily | 2, Weekly | 3, Monthly
**ffi_soybev_d1**	day_1_postbreakfast_questionnaire		radio	Fortified soy beverage	0, Never | 1, Daily | 2, Weekly | 3, Monthly
**ffi_sodabev_d1**	day_1_postbreakfast_questionnaire		radio	Soft drinks, soda, pop: Regular (not diet)	0, Never | 1, Daily | 2, Weekly | 3, Monthly
**ffi_dietsodabev_d1**	day_1_postbreakfast_questionnaire		radio	Soft drinks, soda, pop: Diet	0, Never | 1, Daily | 2, Weekly | 3, Monthly
**ffi_beerbev_d1**	day_1_postbreakfast_questionnaire		radio	Beer	0, Never | 1, Daily | 2, Weekly | 3, Monthly
**ffi_winebev_d1**	day_1_postbreakfast_questionnaire		radio	Wine, wine coolers	0, Never | 1, Daily | 2, Weekly | 3, Monthly
**ffi_liquorbev_d1**	day_1_postbreakfast_questionnaire		radio	Liquor, mixed drinks	0, Never | 1, Daily | 2, Weekly | 3, Monthly
**ffi_coffeebev_d1**	day_1_postbreakfast_questionnaire		radio	Coffee	0, Never | 1, Daily | 2, Weekly | 3, Monthly

**ffi_unsweetteabev_d1**	day_1_postbreakfast_questionnaire		radio	Unsweetened tea (green, black)	0, Never | 1, Daily | 2, Weekly | 3, Monthly
**vit_instructions**	day_1_postbreakfast_questionnaire	Vitamins and Supplements	yes/no	Do you take any vitamins such as:<br>Vitamin A<br>Beta-carotene<br>B1 (Thiamin)<br>B2 (Riboflavin)<br>B6<br>B12<br>Vitamin C<br>Vitamin E<br>Folate<br>Lutein<br>Niacin<br>Multivitamin<br>Etc.	
**vit_type**	day_1_postbreakfast_questionnaire		checkbox	Which vitamins do you take (choose all that apply) :	1, Vitamin A (not beta-carotene) | 2, Beta-carotene | 3, B1 (Thiamin) | 4, B2 (Riboflavin) | 5, B6 | 6, B12 | 7, Vitamin C | 8, Vitamin E | 9, Folate | 10, Lutein | 11, Niacin | 12, Multivitamin | 13, Other
**vit_vita_days**	day_1_postbreakfast_questionnaire		dropdown	How many days out of the week do you take vitamin A (not beta-carotene)?	1, 1 | 2, 2 | 3, 3 | 4, 4 | 5, 5 | 6, 6 | 7, 7
**vit_vita_amt**	day_1_postbreakfast_questionnaire		dropdown	On each day you take vitamin A (not beta-carotene), how many times do you take it?	1, 1 | 2, 2 | 3, 3 | 4, 4 | 5, 5 or more
**vit_bcar_days**	day_1_postbreakfast_questionnaire		dropdown	How many days out of the week do you take beta-carotene?	1, 1 | 2, 2 | 3, 3 | 4, 4 | 5, 5 | 6, 6 | 7, 7

**vit_bcar_amt**	day_1_postbreakfast_questionnaire		dropdown	On each day you take beta-carotene, how many times do you take it?	1, 1 | 2, 2 | 3, 3 | 4, 4 | 5, 5 or more
**vit_vb1_days**	day_1_postbreakfast_questionnaire		dropdown	How many days out of the week do you take B1 (thiamin)?	1, 1 | 2, 2 | 3, 3 | 4, 4 | 5, 5 | 6, 6 | 7, 7
**vit_vb1_amt**	day_1_postbreakfast_questionnaire		dropdown	On each day you take B1 (thiamin), how many times do you take it?	1, 1 | 2, 2 | 3, 3 | 4, 4 | 5, 5 or more
**vit_vb2_days**	day_1_postbreakfast_questionnaire		dropdown	How many days out of the week do you take B2 (riboflavin)?	1, 1 | 2, 2 | 3, 3 | 4, 4 | 5, 5 | 6, 6 | 7, 7
**vit_vb2_amt**	day_1_postbreakfast_questionnaire		dropdown	On each day you take B2 (riboflavin), how many times do you take it?	1, 1 | 2, 2 | 3, 3 | 4, 4 | 5, 5 or more
**vit_vb6_days**	day_1_postbreakfast_questionnaire		dropdown	How many days out of the week do you take B6?	1 | 2 | 3 | 4 | 5 | 6 | 7
**vit_vb6_amt**	day_1_postbreakfast_questionnaire		dropdown	On each day you take B6, how many times do you take it?	1, 1 | 2, 2 | 3, 3 | 4, 4 | 5, 5 or more
**vit_vb12_days**	day_1_postbreakfast_questionnaire		dropdown	How many days out of the week do you take B12?	1 | 2 | 3 | 4 | 5 | 6 | 7
**vit_vb12_amt**	day_1_postbreakfast_questionnaire		dropdown	On each day you take B12, how many times do you take it?	1, 1 | 2, 2 | 3, 3 | 4, 4 | 5, 5 or more
**vit_vc_days**	day_1_postbreakfast_questionnaire		dropdown	How many days out of the week do you take vitamin C?	1, 1 | 2, 2 | 3, 3 | 4, 4 | 5, 5 | 6, 6 | 7, 7
**vit_vc_amt**	day_1_postbreakfast_questionnaire		dropdown	On each day you take vitamin C, how many times do you take it?	1, 1 | 2, 2 | 3, 3 | 4, 4 | 5, 5 or more
**vit_ve_days**	day_1_postbreakfast_questionnaire		dropdown	How many days out of the week do you take vitamin E?	1, 1 | 2, 2 | 3, 3 | 4, 4 | 5, 5 | 6, 6 | 7, 7

**vit_ve_amt**	day_1_postbreakfast_questionnaire		dropdown	On each day you take vitamin E, how many times do you take it?	1, 1 | 2, 2 | 3, 3 | 4, 4 | 5, 5 or more
**vit_fola_days**	day_1_postbreakfast_questionnaire		dropdown	How many days out of the week do you take folate?	1, 1 | 2, 2 | 3, 3 | 4, 4 | 5, 5 | 6, 6 | 7, 7
**vit_fola_amt**	day_1_postbreakfast_questionnaire		dropdown	On each day you take folate, how many times do you take it?	1, 1 | 2, 2 | 3, 3 | 4, 4 | 5, 5 or more
**vit_lutein_days**	day_1_postbreakfast_questionnaire		dropdown	How many days out of the week do you take lutein?	1, 1 | 2, 2 | 3, 3 | 4, 4 | 5, 5 | 6, 6 | 7, 7
**vit_lute_amt**	day_1_postbreakfast_questionnaire		dropdown	On each day you take lutein, how many times do you take it?	1, 1 | 2, 2 | 3, 3 | 4, 4 | 5, 5 or more
**vit_niac_days**	day_1_postbreakfast_questionnaire		dropdown	How many days out of the week do you take niacin?	1, 1 | 2, 2 | 3, 3 | 4, 4 | 5, 5 | 6, 6 | 7, 7
**vit_niac_amt**	day_1_postbreakfast_questionnaire		dropdown	On each day you take niacin, how many times do you take it?	1, 1 | 2, 2 | 3, 3 | 4, 4 | 5, 5 or more
**vit_multi_days**	day_1_postbreakfast_questionnaire		dropdown	How many days out of the week do you take a multivitamin?	1, 1 | 2, 2 | 3, 3 | 4, 4 | 5, 5 | 6, 6 | 7, 7
**vit_multi_amt**	day_1_postbreakfast_questionnaire		dropdown	On each day you take a multivitamin, how many times do you take it?	1, 1 | 2, 2 | 3, 3 | 4, 4 | 5, 5 or more
**vit_other_name**	day_1_postbreakfast_questionnaire		text	What is the name of this other vitamin?	
**vit_other_days**	day_1_postbreakfast_questionnaire		dropdown	How many days out of the week do you take this other vitamin?	1, 1 | 2, 2 | 3, 3 | 4, 4 | 5, 5 | 6, 6 | 7, 7

**min_instructions**	day_1_postbreakfast_questionnaire		yes/no	Do you take any minerals such as:<br>Calcium<br>Dolomite<br>Tums<br>Chromium<br>Iron<br>Magnesium<br>Potassium<br>Selenium<br>Zinc<br>Etc.	
**min_type**	day_1_postbreakfast_questionnaire		checkbox	Which minerals do you take?	1, Calcium or dolomite, Tums | 2, Chromium | 3, Iron | 4, Magnesium | 5, Potassium | 6, Selenium | 7, Zinc | 8, Other
**min_calc_days**	day_1_postbreakfast_questionnaire		dropdown	How many days out of the week do you take calcium, dolomite, or Tums?	1, 1 | 2, 2 | 3, 3 | 4, 4 | 5, 5 | 6, 6 | 7, 7
**min_calc_amt**	day_1_postbreakfast_questionnaire		dropdown	On each day you take calcium, dolomite, or Tums, how many times do you take it?	1, 1 | 2, 2 | 3, 3 | 4, 4 | 5, 5 or more
**min_chrom_days**	day_1_postbreakfast_questionnaire		dropdown	How many days out of the week do you take chromium?	1, 1 | 2, 2 | 3, 3 | 4, 4 | 5, 5 | 6, 6 | 7, 7
**min_chrom_amt**	day_1_postbreakfast_questionnaire		dropdown	On each day you take chromium, how many times do you take it?	1, 1 | 2, 2 | 3, 3 | 4, 4 | 5, 5 or more
**min_iron_days**	day_1_postbreakfast_questionnaire		dropdown	How many days out of the week do you take iron?	1, 1 | 2, 2 | 3, 3 | 4, 4 | 5, 5 | 6, 6 | 7, 7
**min_iron_amt**	day_1_postbreakfast_questionnaire		dropdown	On each day you take iron, how many times do you take it?	1, 1 | 2, 2 | 3, 3 | 4, 4 | 5, 5 or more

**min_magn_days**	day_1_postbreakfast_questionnaire		dropdown	How many days out of the week do you take magnesium?	1, 1 | 2, 2 | 3, 3 | 4, 4 | 5, 5 | 6, 6 | 7, 7
**min_magn_amt**	day_1_postbreakfast_questionnaire		dropdown	On each day you take magnesium, how many times do you take it?	1, 1 | 2, 2 | 3, 3 | 4, 4 | 5, 5 or more
**min_pota_days**	day_1_postbreakfast_questionnaire		dropdown	How many days out of the week do you take potassium?	1, 1 | 2, 2 | 3, 3 | 4, 4 | 5, 5 | 6, 6 | 7, 7
**min_pota_amt**	day_1_postbreakfast_questionnaire		dropdown	On each day you take potassium, how many times do you take it?	1, 1 | 2, 2 | 3, 3 | 4, 4 | 5, 5 or more
**min_sele_days**	day_1_postbreakfast_questionnaire		dropdown	How many days out of the week do you take selenium?	1, 1 | 2, 2 | 3, 3 | 4, 4 | 5, 5 | 6, 6 | 7, 7
**min_sele_amt**	day_1_postbreakfast_questionnaire		dropdown	On each day you take selenium, how many times do you take it?	1, 1 | 2, 2 | 3, 3 | 4, 4 | 5, 5 or more
**min_zinc_days**	day_1_postbreakfast_questionnaire		dropdown	How many days out of the week do you take zinc?	1, 1 | 2, 2 | 3, 3 | 4, 4 | 5, 5 | 6, 6 | 7, 7
**min_zinc_amt**	day_1_postbreakfast_questionnaire		dropdown	On each day you take zinc, how many times do you take it?	1, 1 | 2, 2 | 3, 3 | 4, 4 | 5, 5 or more
**min_other_name**	day_1_postbreakfast_questionnaire		text	What is the name of this other mineral?	
**min_other_days**	day_1_postbreakfast_questionnaire		dropdown	How many days out of the week do you take this other mineral?	1, 1 | 2, 2 | 3, 3 | 4, 4 | 5, 5 | 6, 6 | 7, 7
**min_otheramt**	day_1_postbreakfast_questionnaire		dropdown	On each day you take this other mineral, how many times do you take it?	1, 1 | 2, 2 | 3, 3 | 4, 4 | 5, 5 or more

**suppl_instructions**	day_1_postbreakfast_questionnaire		yes/no	Do you take any supplements such as:<br>Brewer's Yeast<br>Creatine<br>Coenzyme Q<br>DHEA<br>Echinacea<br>Ginseng or ginseng tea<br>Ginkgo<br>Glucosamine/Chondroitin<br>Kelp<br>Melatonin<br>Metamucil<br>Other fiber supplements (Citracil)<br>Primrose oil<br>Saw Palmetto<br>St. John's Wort<br>Cod liver oil, other fish oils, or omega-3 fatty acids<br>Etc.	
**suppl_type**	day_1_postbreakfast_questionnaire		checkbox	What supplements do you take?	1, Brewer's Yeast | 2, Creatine | 3, Coenzyme Q | 4, DHEA | 5, Echinacea | 6, Ginseng or ginseng tea | 7, Ginkgo | 8, Glucosamine/Chondroitin | 9, Kelp | 10, Melatonin | 11, Metamucil | 12, Other fiber supplements (Citracil) | 13, Primrose oil | 14, Saw Palmetto | 15, St. John's Wort | 16, Cod liver oil, other fish oils, or omega-3 fatty acids | 17, Other
**suppl_brewersyeast_days**	day_1_postbreakfast_questionnaire		dropdown	How many days out of the week do you take brewer's yeast?	1, 1 | 2, 2 | 3, 3 | 4, 4 | 5, 5 | 6, 6 | 7, 7

**suppl_brewersyeast_amt**	day_1_postbreakfast_questionnaire		dropdown	On each day you take brewer's yeast, how many times do you take it?	1, 1 | 2, 2 | 3, 3 | 4, 4 | 5, 5 or more
**suppl_creatine_days**	day_1_postbreakfast_questionnaire		dropdown	How many days out of the week do you take creatine?	1, 1 | 2, 2 | 3, 3 | 4, 4 | 5, 5 | 6, 6 | 7, 7
**suppl_creatine_amt**	day_1_postbreakfast_questionnaire		dropdown	On each day you take creatine, how many times do you take it?	1, 1 | 2, 2 | 3, 3 | 4, 4 | 5, 5 or more
**suppl_coenzymeq_days**	day_1_postbreakfast_questionnaire		dropdown	How many days out of the week do you take coenzyme Q?	1, 1 | 2, 2 | 3, 3 | 4, 4 | 5, 5 | 6, 6 | 7, 7
**suppl_coenzymeq_amt**	day_1_postbreakfast_questionnaire		dropdown	On each day you take coenzyme Q, how many times do you take it?	1, 1 | 2, 2 | 3, 3 | 4, 4 | 5, 5 or more
**suppl_dhea_days**	day_1_postbreakfast_questionnaire		dropdown	How many days out of the week do you take DHEA?	1 | 2 | 3 | 4 | 5 | 6 | 7
**suppl_dhea_amt**	day_1_postbreakfast_questionnaire		dropdown	On each day you take DHEA, how many times do you take it?	1, 1 | 2, 2 | 3, 3 | 4, 4 | 5, 5 or more
**suppl_echinacea_days**	day_1_postbreakfast_questionnaire		dropdown	How many days out of the week do you take echinacea?	1, 1 | 2, 2 | 3, 3 | 4, 4 | 5, 5 | 6, 6 | 7, 7
**suppl_echinacea_amt**	day_1_postbreakfast_questionnaire		dropdown	On each day you take echinacea, how many times do you take it?	1, 1 | 2, 2 | 3, 3 | 4, 4 | 5, 5 or more
**suppl_ginseng_days**	day_1_postbreakfast_questionnaire		dropdown	How many days out of the week do you take ginseng or ginseng tea?	1, 1 | 2, 2 | 3, 3 | 4, 4 | 5, 5 | 6, 6 | 7, 7
**suppl_ginseng_amt**	day_1_postbreakfast_questionnaire		dropdown	On each day you take ginseng or ginseng tea, how many times do you take it?	1, 1 | 2, 2 | 3, 3 | 4, 4 | 5, 5 or more
**suppl_ginkgo_days**	day_1_postbreakfast_questionnaire		dropdown	How many days out of the week do you take ginkgo?	1, 1 | 2, 2 | 3, 3 | 4, 4 | 5, 5 | 6, 6 | 7, 7

**suppl_ginkgo_amt**	day_1_postbreakfast_questionnaire		dropdown	On each day you take ginkgo, how many times do you take it?	1, 1 | 2, 2 | 3, 3 | 4, 4 | 5, 5 or more
**suppl_glucosamine_days**	day_1_postbreakfast_questionnaire		dropdown	How many days out of the week do you take glucosamine/chondroitin?	1, 1 | 2, 2 | 3, 3 | 4, 4 | 5, 5 | 6, 6 | 7, 7
**suppl_glucosamine_amt**	day_1_postbreakfast_questionnaire		dropdown	On each day you take glucosamine/chondroitin, how many times do you take it?	1, 1 | 2, 2 | 3, 3 | 4, 4 | 5, 5 or more
**suppl_kelp_days**	day_1_postbreakfast_questionnaire		dropdown	How many days out of the week do you take kelp?	1, 1 | 2, 2 | 3, 3 | 4, 4 | 5, 5 | 6, 6 | 7, 7
**suppl_kelp_amt**	day_1_postbreakfast_questionnaire		dropdown	On each day you take kelp, how many times do you take it?	1, 1 | 2, 2 | 3, 3 | 4, 4 | 5, 5 or more
**suppl_melatonin_days**	day_1_postbreakfast_questionnaire		dropdown	How many days out of the week do you take melatonin?	1, 1 | 2, 2 | 3, 3 | 4, 4 | 5, 5 | 6, 6 | 7, 7
**suppl_melatonin_amt**	day_1_postbreakfast_questionnaire		dropdown	On each day you take melatonin, how many times do you take it?	1, 1 | 2, 2 | 3, 3 | 4, 4 | 5, 5 or more
**suppl_metamucil_days**	day_1_postbreakfast_questionnaire		dropdown	How many days out of the week do you take metamucil?	1, 1 | 2, 2 | 3, 3 | 4, 4 | 5, 5 | 6, 6 | 7, 7
**suppl_metamucil_amt**	day_1_postbreakfast_questionnaire		dropdown	On each day you take metamucil, how many times do you take it?	1, 1 | 2, 2 | 3, 3 | 4, 4 | 5, 5 or more
**suppl_fiber_days**	day_1_postbreakfast_questionnaire		dropdown	How many days out of the week do you take other fiber supplements (citracil)?	1, 1 | 2, 2 | 3, 3 | 4, 4 | 5, 5 | 6, 6 | 7, 7
**suppl_fiber_amt**	day_1_postbreakfast_questionnaire		dropdown	On each day you take other fiber supplements (citracil), how many times do you take it?	1, 1 | 2, 2 | 3, 3 | 4, 4 | 5, 5 or more

**suppl_primrose_days**	day_1_postbreakfast_questionnaire		dropdown	How many days out of the week do you take primrose oil?	1, 1 | 2, 2 | 3, 3 | 4, 4 | 5, 5 | 6, 6 | 7, 7
**suppl_primrose_amt**	day_1_postbreakfast_questionnaire		dropdown	On each day you take primrose oil, how many times do you take it?	1, 1 | 2, 2 | 3, 3 | 4, 4 | 5, 5 or more
**suppl_palmetto_days**	day_1_postbreakfast_questionnaire		dropdown	How many days out of the week do you take saw palmetto?	1, 1 | 2, 2 | 3, 3 | 4, 4 | 5, 5 | 6, 6 | 7, 7
**suppl_palmetto_amt**	day_1_postbreakfast_questionnaire		dropdown	On each day you take saw palmetto, how many times do you take it?	1, 1 | 2, 2 | 3, 3 | 4, 4 | 5, 5 or more
**suppl_stjohnswort_days**	day_1_postbreakfast_questionnaire		dropdown	How many days out of the week do you take St. John's Wort?	1 | 2 | 3 | 4 | 5 | 6 | 7
**suppl_stjohnswort_amt**	day_1_postbreakfast_questionnaire		dropdown	On each day you take St. John's Wort, how many times do you take it?	1, 1 | 2, 2 | 3, 3 | 4, 4 | 5, 5 or more
**suppl_codliveroil_days**	day_1_postbreakfast_questionnaire		dropdown	How many days out of the week do you take cod liver oil, other fish oils, or omega-3 fatty acids?	1, 1 | 2, 2 | 3, 3 | 4, 4 | 5, 5 | 6, 6 | 7, 7
**suppl_codliveroil_amt**	day_1_postbreakfast_questionnaire		dropdown	On each day you take cod liver oil, other fish oils, or omega-3 fatty acids, how many times do you take it?	1, 1 | 2, 2 | 3, 3 | 4, 4 | 5, 5 or more
**suppl_fattyacids_dose**	day_1_postbreakfast_questionnaire		text	What dose of omega-3 fatty acids do you take (per day)?	
**suppl_other_name**	day_1_postbreakfast_questionnaire		text	What is the name of this other supplement?	
**suppl_other_days**	day_1_postbreakfast_questionnaire		dropdown	How many days out of the week do you take this other supplement?	1, 1 | 2, 2 | 3, 3 | 4, 4 | 5, 5 | 6, 6 | 7, 7

**suppl_other_amt**	day_1_postbreakfast_questionnaire		dropdown	On each day you take this other supplement, how many times do you take it?	1, 1 | 2, 2 | 3, 3 | 4, 4 | 5, 5 or more
**hair_wash_freq**	day_1_postbreakfast_questionnaire	The following questions will help us better understand how certain hormones are expressed in your hair.	dropdown	How frequently do you wash your hair?	1, 1 | 2, 2 | 3, 3 | 4, 4 | 5, 5 | 6, 6 | 7, 7 | 8,8 | 9,9 | 10,10
**hair_conditioner**	day_1_postbreakfast_questionnaire		yes/no	Do you use a conditioner?	
**hair_bleach**	day_1_postbreakfast_questionnaire		yes/no	Do you color or bleach your hair?	
**hair_perm**	day_1_postbreakfast_questionnaire		yes/no	Have you recently had a perm?	
**hair_stranghten**	day_1_postbreakfast_questionnaire		yes/no	Do you use chemical hair straighteners?	
**hair_med**	day_1_postbreakfast_questionnaire		yes/no	Do you use over the counter or prescription medications for any scalp conditions?	
**hair_steroid**	day_1_postbreakfast_questionnaire		yes/no	Have you taken any steroids?	

2.2.2 Primary Appraisal Secondary Appraisal

Redcap Form Name: Pasa

Questionnaire Name	Title	Variable Name
2.2.2.1 PASA	Primary Appraisal Secondary Appraisal (PASA)	pasa

Variable / Field Name	Form Name	Section Header	Field Type	Field Label	Choices, Calculations, OR Slider Labels

**pasa_1**	pasa	The following sentences refer to the oncoming situation. Please indicate what goes through your mind regarding all these sentences by ticking the respective circle. For each sentences, you can thereby indicate how much you agree or disagree with the it. Please answer all sentences.<br>Please choose one of the following answers for each question:<br>1, Totally disagree | 2, Rather disagree | 3, Disagree to some extent | 4, Agree to some extent | 5, Rather agree | 6, Totally agree	radio	1. I do not feel threatened by the situation	1, Totally disagree | 2, Rather disagree | 3, Disagree to some extent | 4, Agree to some extent | 5, Rather agree | 6, Totally agree
**pasa_5**	pasa		radio	5. I find this situation very unpleasant.	1, Totally disagree | 2, Rather disagree | 3, Disagree to some extent | 4, Agree to some extent | 5, Rather agree | 6, Totally agree
**pasa_9**	pasa		radio	9. I do not feel worried because the situation does not represent any threat for me.	1, Totally disagree | 2, Rather disagree | 3, Disagree to some extent | 4, Agree to some extent | 5, Rather agree | 6, Totally agree
**pasa_13**	pasa		radio	13. This situation scares me.	1, Totally disagree | 2, Rather disagree | 3, Disagree to some extent | 4, Agree to some extent | 5, Rather agree | 6, Totally agree

2.2.3 Recovery

Redcap Form Name: Day 1 Recovery

Questionnaire Name	Title	Variable Name
2.2.3.1 Fatigue	Modified Fatigue Impact Scale (MFIS)	mfis
2.2.3.2 Autonomic Symptoms	Composite Autonomic Symptom Score (COMPASS 31)	bodysymp

Variable / Field Name	Form Name	Section Header	Field Type	Field Label	Choices, Calculations, OR Slider Labels

**day1_recovery_cv**	day_1_recovery		descriptive	Thank you for your participation in the MiSBIE study!<br><br>The following questions are a validated set of questionnaires that will help us understand you better as a person. It is very important that you answer each question as well as you can.<br><br>This package should take about 30 min to complete.<br><br>We want to take this opportunity to remind you that all answers will remain strictly confidential.	
**mfis_1**	day_1_recovery	Fatigue<br>Instructions: The following is a list of statements that describe how fatigue may affect a person. Fatigue is a feeling of physical tiredness and lack of energy that many people experience from time to time. In medical conditions like MS, feelings of fatigue can occur more often and have a greater impact than usual. Please read each statement carefully and then select the one number that best indicates how often fatigue has affected you in this way during the past 4 weeks. (If you need help in marking your responses, tell the	radio	1. Because of my fatigue during the past 4 weeks, I have been less alert.	0, Never | 1, Rarely | 2, Sometimes | 3, Often | 4, Almost always

		interviewer the number of the best response.) Please answer every question. The interviewer can explain any words or phrases that you do not understand.			
**mfis_2**	day_1_recovery		radio	2. Because of my fatigue during the past 4 weeks, I have had difficulty paying attention for long periods of time.	0, Never | 1, Rarely | 2, Sometimes | 3, Often | 4, Almost always
**mfis_3**	day_1_recovery		radio	3. Because of my fatigue during the past 4 weeks, I have been unable to think clearly.	0, Never | 1, Rarely | 2, Sometimes | 3, Often | 4, Almost always
**mfis_4**	day_1_recovery		radio	4. Because of my fatigue during the past 4 weeks, I have been clumsy and uncoordinated.	0, Never | 1, Rarely | 2, Sometimes | 3, Often | 4, Almost always
**mfis_5**	day_1_recovery		radio	5. Because of my fatigue during the past 4 weeks, I have been forgetful.	0, Never | 1, Rarely | 2, Sometimes | 3, Often | 4, Almost always
**mfis_6**	day_1_recovery		radio	6. Because of my fatigue during the past 4 weeks, I have had to pace myself in my physical activities.	0, Never | 1, Rarely | 2, Sometimes | 3, Often | 4, Almost always
**mfis_7**	day_1_recovery		radio	7. Because of my fatigue during the past 4 weeks, I have been less motivated to do anything that requires physical effort.	0, Never | 1, Rarely | 2, Sometimes | 3, Often | 4, Almost always

**mfis_8**	day_1_recovery		radio	8. Because of my fatigue during the past 4 weeks, I have been less motivated to participate in social activities.	0, Never | 1, Rarely | 2, Sometimes | 3, Often | 4, Almost always
**mfis_9**	day_1_recovery		radio	9. Because of my fatigue during the past 4 weeks, I have been less motivated to do things away from home.	0, Never | 1, Rarely | 2, Sometimes | 3, Often | 4, Almost always
**mfis_10**	day_1_recovery		radio	10. Because of my fatigue during the past 4 weeks, I have had trouble maintaining physical effort for long periods.	0, Never | 1, Rarely | 2, Sometimes | 3, Often | 4, Almost always
**mfis_11**	day_1_recovery		radio	11. Because of my fatigue during the past 4 weeks, I have had difficulty making decisions.	0, Never | 1, Rarely | 2, Sometimes | 3, Often | 4, Almost always
**mfis_12**	day_1_recovery		radio	12. Because of my fatigue during the past 4 weeks, I have been less motivated to do anything that requires thinking.	0, Never | 1, Rarely | 2, Sometimes | 3, Often | 4, Almost always
**mfis_13**	day_1_recovery		radio	13. Because of my fatigue during the past 4 weeks, my muscles have felt weak.	0, Never | 1, Rarely | 2, Sometimes | 3, Often | 4, Almost always
**mfis_14**	day_1_recovery		radio	14. Because of my fatigue during the past 4 weeks, I have been physically uncomfortable.	0, Never | 1, Rarely | 2, Sometimes | 3, Often | 4, Almost always

**mfis_15**	day_1_recovery		radio	15. Because of my fatigue during the past 4 weeks, I have had trouble finishing tasks that require thinking.	0, Never | 1, Rarely | 2, Sometimes | 3, Often | 4, Almost always
**mfis_16**	day_1_recovery		radio	16. Because of my fatigue during the past 4 weeks, I have had difficulty organizing my thoughts when doing things at home or at work.	0, Never | 1, Rarely | 2, Sometimes | 3, Often | 4, Almost always
**mfis_17**	day_1_recovery		radio	17. Because of my fatigue during the past 4 weeks, I have been less able to complete tasks that require physical effort.	0, Never | 1, Rarely | 2, Sometimes | 3, Often | 4, Almost always
**mfis_18**	day_1_recovery		radio	18. Because of my fatigue during the past 4 weeks, my thinking has slowed down.	0, Never | 1, Rarely | 2, Sometimes | 3, Often | 4, Almost always
**mfis_19**	day_1_recovery		radio	19. Because of my fatigue during the past 4 weeks, I have had trouble concentrating.	0, Never | 1, Rarely | 2, Sometimes | 3, Often | 4, Almost always
**mfis_20**	day_1_recovery		radio	20. Because of my fatigue during the past 4 weeks, I have limited my physical activities.	0, Never | 1, Rarely | 2, Sometimes | 3, Often | 4, Almost always
**mfis_21**	day_1_recovery		radio	21. Because of my fatigue during the past 4 weeks, I have needed to rest more often or for longer periods.	0, Never | 1, Rarely | 2, Sometimes | 3, Often | 4, Almost always
**compass_1**	day_1_recovery	Body Symptoms	yes/no	In the past year, have you ever felt faint, dizzy,	

		Instructions: The following sets of questions are about some general symptoms you may experience. Please answer each question carefully.		"goofy," or had difficulty thinking soon after standing up from a sitting or lying position?	
**compass_2**	day_1_recovery		radio	When standing up, how frequently do you get these feelings or symptoms?	1, Rarely | 2, Occasionally | 3, Frequently | 4, Almost always
**compass_3**	day_1_recovery		radio	How would you rate the severity of these feelings or symptoms?	1, Mild | 2, Moderate | 3, Severe
**compass_4**	day_1_recovery		radio	In the past year, have these feelings or symptoms that you experienced:	3, Gotten much worse | 2, Gotten somewhat worse | 1, Stayed about the same | 0.1, Gotten somewhat better | 0.01, Gotten much better | 0, Completely gone
**compass_5**	day_1_recovery		yes/no	In the past year, have you ever noticed color changes in your skin, such as red, white, or purple?	
**compass_6**	day_1_recovery		checkbox	What parts of your body are affected by these color changes? Check all that apply.	1, Hands | 2, Feet
**compass_7**	day_1_recovery		radio	Are these changes in your skin color:	3, Gotten much worse | 2, Gotten somewhat worse | 1, Stayed about the same | 0.1, Gotten somewhat better |0.01, Gotten much

					better | 0.001, Completely gone
**compass_8**	day_1_recovery		radio	In the past 5 years, what changes, if any, have occurred in your general body sweating?	1, I sweat much more than I used to | 0.1, I sweat somewhat more than I used to | 0.01, I haven't noticed any changes in my sweating | 1.1, I sweat somewhat less than I used to | 2, I sweat much less than I used to
**compass_9**	day_1_recovery		yes/no	Do your eyes feel excessively dry?	
**compass_10**	day_1_recovery		yes/no	Does you mouth feel excessively dry?	
**compass_11**	day_1_recovery		radio	For the symptom of dry eyes or dry mouth that you have had for the longest period of time, has this symptom:	0, I have not had any of these symptoms | 3, Gotten much worse | 2, Gotten somewhat worse | 1, Stayed about the same | 0.1, Gotten somewhat better | 0.01, Gotten much better | 0.001, Completely gone
**compass_12**	day_1_recovery		radio	In the past year, have you noticed any changes in how quickly you get full when eating a meal?	2, I get full a lot more quickly now than I used to | 1, I get full more quickly now than I used to | 0.1, I haven't noticed any change | 0.01, I get full less quickly now than I used to | 0.001, I get full a lot less quickly now than I used to

**compass_13**	day_1_recovery		radio	In the past year, have you felt excessively full or persistently full (bloated feeling) after a meal?	0, Never | 1, Sometimes | 2, A lot of the time
**compass_14**	day_1_recovery		radio	In the past year, have you vomited after a meal?	0, Never | 1, Sometimes | 2, A lot of the time
**compass_15**	day_1_recovery		radio	In the past year, have you had a cramping or colicky abdominal pain?	0, Never | 1, Sometimes | 2, A lot of the time
**compass_16**	day_1_recovery		yes/no	In the past year, have you had any bouts of diarrhea?	
**compass_17**	day_1_recovery		radio	How frequently does this occur?	1, Rarely | 2, Occasionallys | 3, Frequently | 4, Almost always
**compass_17_freq**	day_1_recovery		radio	How frequently?	1, Less than 2 times per month | 2, 2-5 times per month | 3, 5-10 times per month | 4, 15-20 times per month | 5, 20-25 times per month | 6, 25-30 times per month
**compass_18**	day_1_recovery		radio	How severe are these bouts of diarrhea?	1, Mild | 2, Moderate | 3, Severe
**compass_19**	day_1_recovery		radio	Have your bouts of diarrhea:	3, Gotten much worse | 2, Gotten somewhat worse | 1, Stayed about the same | 0.1, Gotten somewhat better | 0.01, Gotten much better | 0.001, Completely gone

**compass_20**	day_1_recovery		yes/no	In the past year, have you been constipated?	
**compass_21**	day_1_recovery		radio	How frequently are you constipated?	1, Rarely | 2, Occasionally | 3, Frequently | 4, Almost always
**compass_21_freq**	day_1_recovery		radio	How frequently?	1, Less than 2 times per month | 2, 2-5 times per month | 3, 5-10 times per month | 4, 15-20 times per month | 5, 20-25 times per month | 6, 25-30 times per month
**compass_22**	day_1_recovery		radio	How severe are these episodes of constipation?	1, Mild | 2, Moderate | 3, Severe
**compass_23**	day_1_recovery		radio	Has your constipation:	3, Gotten much worse | 2, Gotten somewhat worse | 1, Stayed about the same | 0.1, Gotten somewhat better |0.01, Gotten much better |0.001, Completely gone
**compass_24**	day_1_recovery		radio	In the past year, have you ever lost control of your bladder function?	1, Never | 2, Occasionallys | 3, Frequently | 4, Almost always
**compass_24_freq**	day_1_recovery		radio	How frequently?	1, Less than 2 times per month | 2, 2-5 times per month | 3, 5-10 times per month | 4, 15-20 times per month | 5, 20-25 times per month | 6, 25-30 times per month

**compass_25**	day_1_recovery		radio	In the past year, have you had difficulty passing urine?	1, Never | 2, Occasionally | 3, Frequently | 4, Almost always
**compass_25_freq**	day_1_recovery		radio	How frequently?	1, Less than 2 times per month | 2, 2-5 times per month | 3, 5-10 times per month | 4, 15-20 times per month | 5, 20-25 times per month | 6, 25-30 times per month
**compass_26**	day_1_recovery		radio	In the past year, have you had trouble completely emptying your bladder?	1, Never | 2, Occasionally | 3, Frequently | 4, Almost always
**compass_26_freq**	day_1_recovery		radio	How frequently?	1, Less than 2 times per month | 2, 2-5 times per month | 3, 5-10 times per month | 4, 15-20 times per month | 5, 20-25 times per month | 6, 25-30 times per month
**compass_27**	day_1_recovery		radio	In the past year, without sunglasses or tinted glasses, has bright light bothered your eyes?	1, Never | 2, Occasionally | 3, Frequently | 4, Almost always
**compass_28**	day_1_recovery		radio	How severe is this sensitivity to bright light?	1, Mild | 2, Moderate | 3, Severe
**compass_29**	day_1_recovery		radio	In the past year, have you had trouble focusing your eyes?	1, Never | 2, Occasionally | 3, Frequently | 4, Almost always
**compass_30**	day_1_recovery		radio	How severe is this focusing problem?	1, Mild | 2, Moderate | 3, Severe

**compass_31**	day_1_recovery		radio	Have the most troublesome symptoms with your eyes (i.e.sensitivity to bright light or trouble focusing):	0, I have not had any of these symptoms | 3, Gotten much worse | 2, Gotten somewhat worse | 1, Stayed about the same | 0.1, Gotten somewhat better | 0.01, Gotten much better | 0.001, Completely gone
**bipq_1**	day_1_recovery	Illness Perception<br>Instructions: For the following questions, please select the number that best corresponds to your view:	radio	How much does illness affect your life?	1, 1: No affect at all | 2, 2 | 3, 3 | 4, 4 | 5, 5 | 6, 6 | 7, 7 | 8, 8 | 9, 9 | 10, 10: Severely affects my life
**bipq_2**	day_1_recovery		radio	How long do you think your illness will continue?	1, 1: A very short time | 2, 2 | 3, 3 | 4, 4 | 5, 5 | 6, 6 | 7, 7 | 8, 8 | 9, 9 | 10, 10: Forever
**bipq_3**	day_1_recovery		radio	How much control do you feel you have over your illness?	1, 1: Absolutely no control | 2, 2 | 3, 3 | 4, 4 | 5, 5 | 6, 6 | 7, 7 | 8, 8 | 9, 9 | 10, 10: Extreme amount of control
**bipq_4**	day_1_recovery		radio	How much do you think your treatmnet can help your illness?	1, 1: Not at all | 2, 2 | 3, 3 | 4, 4 | 5, 5 | 6, 6 | 7, 7 | 8, 8 | 9, 9 | 10, 10: Extremely helpful
**bipq_5**	day_1_recovery		radio	How much do you experience symptoms from your illness?	1, 1: No symptoms at all | 2, 2 | 3, 3 | 4, 4 | 5, 5 | 6, 6 | 7, 7 | 8, 8 | 9, 9 | 10, 10: Many severe symptoms
**bipq_6**	day_1_recovery		radio	How concerned are you about your illness?	1, 1: Not at all concerned | 2, 2 | 3, 3 | 4, 4 | 5, 5 | 6, 6

					| 7, 7 | 8, 8 | 9, 9 | 10, 10: Extremely concerned
**bipq_7**	day_1_recovery		radio	How well do you feel you understand your illness?	1, 1: Do not understand at all | 2, 2 | 3, 3 | 4, 4 | 5, 5 | 6, 6 | 7, 7 | 8, 8 | 9, 9 | 10, 10: Understand very clearly
**bipq_8**	day_1_recovery		radio	How much does your illness affect you emotionally? (e.g. Does it make you angry, scared, upset, or depressed?)	1, 1: Not at all affected emotionally | 2, 2 | 3, 3 | 4, 4 | 5, 5 | 6, 6 | 7, 7 | 8, 8 | 9, 9 | 10, 10: Extremely affected emotionally
**bipq_9**	day_1_recovery	Please list in rank order the three most important factors that you believe caused your illness.	text	1	
**bipq_10**	day_1_recovery		text	2	
**bipq_11**	day_1_recovery		text	3	
**spa_1**	day_1_recovery	Getting Older	radio	Things keep getting worse as I get older.	1, 1: Does not apply to me at all | 2, 2 | 3, 3 | 4, 4 | 5, 5: Applies very well to me
**spa_2**	day_1_recovery		radio	I have as much pep as I had last year.	1, 1: Does not apply to me at all | 2, 2 | 3, 3 | 4, 4 | 5, 5: Applies very well to me
**spa_3**	day_1_recovery		radio	As I get older, I am less useful.	1, 1: Does not apply to me at all | 2, 2 | 3, 3 | 4, 4 | 5, 5: Applies very well to me
**spa_4**	day_1_recovery		radio	I am as happy now as I was when I was younger.	1, 1: Does not apply to me at all | 2, 2 | 3, 3 | 4, 4 | 5, 5: Applies very well to me

**spa_5**	day_1_recovery		radio	As I get older, things are ________ than I thought they would be.	1, Better | 2, The same | 3, Worse
**language_spoken**	day_1_recovery	Language History	dropdown	What language(s) did you speak at home when you were growing up?	1, English | 2, Other(List all):
**language_spokenhome_other**	day_1_recovery		dropdown	How many languages other than English did you speak when you was growing up?	1, 1 | 2, 2 | 3, 3 | 4, 4 | 5, 5
**language_otherhome_1**	day_1_recovery		text	Other(List one at a time)	
**language_otherhome_2**	day_1_recovery		text	Other(List one at a time)	
**language_otherhome_3**	day_1_recovery		text	Other(List one at a time)	
**language_otherhome_4**	day_1_recovery		text	Other(List one at a time)	
**language_otherhome_5**	day_1_recovery		text	Other(List one at a time)	
**language_diff_lang**	day_1_recovery		yes/no	Outside English and the language(s) you learn when you was growing up, do you know, or did you ever study, a different language?	
**language_spoken_other**	day_1_recovery		dropdown	How many other languages did you learn?	1, 1 | 2, 2 | 3, 3 | 4, 4 | 5, 5
**language_other_1**	day_1_recovery		text	Other(List one at a time)	
**language_other_2**	day_1_recovery		text	Other(List one at a time)	

**language_other_3**	day_1_recovery		text	Other(List one at a time)	
**language_other_4**	day_1_recovery		text	Other(List one at a time)	
**language_other_5**	day_1_recovery		text	Other(List one at a time)	
**language_english_at_home**	day_1_recovery	How did you learn the following language(s)?<br><br>English	yes/no	AT HOME	
**language_english_other**	day_1_recovery		text	OTHER (please explain) [e.g. School, Live abroad, Work, Non-formal study]	
**language_english_school**	day_1_recovery		text	In SCHOOL (indicate # of years of study)	
**language_english_college**	day_1_recovery		text	In COLLEGE (indicate # of years of study)	
**language_english_frequent**	day_1_recovery		text	Age of Regular usage (frequently, outside of classroom	
**language_otherhome1_at_home**	day_1_recovery	[language_otherhome_1]	yes/no	AT HOME	
**language_otherhome1_otherhome**	day_1_recovery		text	OTHER (please explain) [e.g. School, Live abroad, Work, Non-formal study]	
**language_otherhome1_school**	day_1_recovery		text	In SCHOOL (indicate # of years of study)	
**language_otherhome1_college**	day_1_recovery		text	In COLLEGE (indicate # of years of study)	

**language_otherhome1_frequent**	day_1_recovery		text	Age of Regular usage (frequently, outside of classroom	
**language_otherhome2_at_home**	day_1_recovery	[language_otherhome_2]	yes/no	AT HOME	
**language_otherhome2_otherhome**	day_1_recovery		text	OTHER (please explain) [e.g. School, Live abroad, Work, Non-formal study]	
**language_otherhome2_school**	day_1_recovery		text	In SCHOOL (indicate # of years of study)	
**language_otherhome2_college**	day_1_recovery		text	In COLLEGE (indicate # of years of study)	
**language_otherhome2_frequent**	day_1_recovery		text	Age of Regular usage (frequently, outside of classroom	
**language_otherhome3_at_home**	day_1_recovery	[language_otherhome_3]	yes/no	AT HOME	
**language_otherhome3_otherhome**	day_1_recovery		text	OTHER (please explain) [e.g. School, Live abroad, Work, Non-formal study]	
**language_otherhome3_school**	day_1_recovery		text	In SCHOOL (indicate # of years of study)	
**language_otherhome3_college**	day_1_recovery		text	In COLLEGE (indicate # of years of study)	
**language_otherhome3_frequent**	day_1_recovery		text	Age of Regular usage (frequently, outside of classroom	
**language_otherhome4_at_home**	day_1_recovery	[language_otherhome_4]	yes/no	AT HOME	

**language_otherhome4_otherhome**	day_1_recovery		text	OTHER (please explain) [e.g. School, Live abroad, Work, Non-formal study]	
**language_otherhome4_school**	day_1_recovery		text	In SCHOOL (indicate # of years of study)	
**language_otherhome4_college**	day_1_recovery		text	In COLLEGE (indicate # of years of study)	
**language_otherhome4_frequent**	day_1_recovery		text	Age of Regular usage (frequently, outside of classroom	
**language_otherhome5_at_home**	day_1_recovery	[language_otherhome_5]	yes/no	AT HOME	
**language_otherhome5_otherhome**	day_1_recovery		text	OTHER (please explain) [e.g. School, Live abroad, Work, Non-formal study]	
**language_otherhome5_school**	day_1_recovery		text	In SCHOOL (indicate # of years of study)	
**language_otherhome5_college**	day_1_recovery		text	In COLLEGE (indicate # of years of study)	
**language_other1_at_home**	day_1_recovery	[language_other_1]	yes/no	AT HOME	
**language_other1_other**	day_1_recovery		text	OTHER (please explain) [e.g. School, Live abroad, Work, Non-formal study]	
**language_other1_school**	day_1_recovery		text	In SCHOOL (indicate # of years of study)	
**language_other1_college**	day_1_recovery		text	In COLLEGE (indicate # of years of study)	

**language_other1_frequent**	day_1_recovery		text	Age of Regular usage (frequently, outside of classroom	
**language_other2_at_home**	day_1_recovery	[language_other_2]	yes/no	AT HOME	
**language_other2_other**	day_1_recovery		text	OTHER (please explain) [e.g. School, Live abroad, Work, Non-formal study]	
**language_other2_school**	day_1_recovery		text	In SCHOOL (indicate # of years of study)	
**language_other2_college**	day_1_recovery		text	In COLLEGE (indicate # of years of study)	
**language_other2_frequent**	day_1_recovery		text	Age of Regular usage (frequently, outside of classroom	
**language_other3_at_home**	day_1_recovery	[language_other_3]	yes/no	AT HOME	
**language_other3_other**	day_1_recovery		text	OTHER (please explain) [e.g. School, Live abroad, Work, Non-formal study]	
**language_other3_school**	day_1_recovery		text	In SCHOOL (indicate # of years of study)	
**language_other3_college**	day_1_recovery		text	In COLLEGE (indicate # of years of study)	
**language_other3_frequent**	day_1_recovery		text	Age of Regular usage (frequently, outside of classroom	
**language_other4_at_home**	day_1_recovery	[language_other_4]	yes/no	AT HOME	

**language_other4_other**	day_1_recovery		text	OTHER (please explain) [e.g. School, Live abroad, Work, Non-formal study]	
**language_other4_school**	day_1_recovery		text	In SCHOOL (indicate # of years of study)	
**language_other4_college**	day_1_recovery		text	In COLLEGE (indicate # of years of study)	
**language_other4_frequent**	day_1_recovery		text	Age of Regular usage (frequently, outside of classroom	
**language_other5_at_home**	day_1_recovery	[language_other_5]	yes/no	AT HOME	
**language_other5_other**	day_1_recovery		text	OTHER (please explain) [e.g. School, Live abroad, Work, Non-formal study]	
**language_other5_school**	day_1_recovery		text	In SCHOOL (indicate # of years of study)	
**language_other5_college**	day_1_recovery		text	In COLLEGE (indicate # of years of study)	
**language_easy**	day_1_recovery		text	What language is it easiest for you to speak in (English or Other Language)? Please choose one:	
**language_other5_frequent**	day_1_recovery		text	Age of Regular usage (frequently, outside of classroom	
**language_english_speaking**	day_1_recovery	How proficient are you currently in the following	dropdown	Speaking	1, Almost None | 2, Very Poor | 3, Fair | 4,

		language? Please rate your knowledge of the language using the following scale.<br><br>English			Functional | 5, Good | 6, Very Good | 7, Like Native Speaker
**language_english_reading**	day_1_recovery		dropdown	Reading	1, Almost None | 2, Very Poor | 3, Fair | 4, Functional | 5, Good | 6, Very Good | 7, Like Native Speaker
**language_english_writing**	day_1_recovery		dropdown	Writing	1, Almost None | 2, Very Poor | 3, Fair | 4, Functional | 5, Good | 6, Very Good | 7, Like Native Speaker
**language_english_understanding**	day_1_recovery		dropdown	Understanding	1, Almost None | 2, Very Poor | 3, Fair | 4, Functional | 5, Good | 6, Very Good | 7, Like Native Speaker
**language_english_average**	day_1_recovery		dropdown	Average	1, Almost None | 2, Very Poor | 3, Fair | 4, Functional | 5, Good | 6, Very Good | 7, Like Native Speaker
**language_otherhome1_speaking**	day_1_recovery	[language_otherhome_1]	dropdown	Speaking	1, Almost None | 2, Very Poor | 3, Fair | 4, Functional | 5, Good | 6, Very Good | 7, Like Native Speaker
**language_otherhome1_reading**	day_1_recovery		dropdown	Reading	1, Almost None | 2, Very Poor | 3, Fair | 4, Functional | 5, Good | 6,

					Very Good | 7, Like Native Speaker
**language_otherhome1_writing**	day_1_recovery		dropdown	Writing	1, Almost None | 2, Very Poor | 3, Fair | 4, Functional | 5, Good | 6, Very Good | 7, Like Native Speaker
**language_otherhome1_understanding**	day_1_recovery		dropdown	Understanding	1, Almost None | 2, Very Poor | 3, Fair | 4, Functional | 5, Good | 6, Very Good | 7, Like Native Speaker
**language_otherhome1_average**	day_1_recovery		dropdown	Average	1, Almost None | 2, Very Poor | 3, Fair | 4, Functional | 5, Good | 6, Very Good | 7, Like Native Speaker
**language_otherhome2_speaking**	day_1_recovery	[language_otherhome_2]	dropdown	Speaking	1, Almost None | 2, Very Poor | 3, Fair | 4, Functional | 5, Good | 6, Very Good | 7, Like Native Speaker
**language_otherhome2_reading**	day_1_recovery		dropdown	Reading	1, Almost None | 2, Very Poor | 3, Fair | 4, Functional | 5, Good | 6, Very Good | 7, Like Native Speaker
**language_otherhome2_writing**	day_1_recovery		dropdown	Writing	1, Almost None | 2, Very Poor | 3, Fair | 4, Functional | 5, Good | 6, Very Good | 7, Like Native Speaker

**language_otherhome2_understanding**	day_1_recovery		dropdown	Understanding	1, Almost None | 2, Very Poor | 3, Fair | 4, Functional | 5, Good | 6, Very Good | 7, Like Native Speaker
**language_otherhome2_average**	day_1_recovery		dropdown	Average	1, Almost None | 2, Very Poor | 3, Fair | 4, Functional | 5, Good | 6, Very Good | 7, Like Native Speaker
**language_otherhome3_speaking**	day_1_recovery	[language_otherhome_3]	dropdown	Speaking	1, Almost None | 2, Very Poor | 3, Fair | 4, Functional | 5, Good | 6, Very Good | 7, Like Native Speaker
**language_otherhome3_reading**	day_1_recovery		dropdown	Reading	1, Almost None | 2, Very Poor | 3, Fair | 4, Functional | 5, Good | 6, Very Good | 7, Like Native Speaker
**language_otherhome3_writing**	day_1_recovery		dropdown	Writing	1, Almost None | 2, Very Poor | 3, Fair | 4, Functional | 5, Good | 6, Very Good | 7, Like Native Speaker
**language_otherhome3_understanding**	day_1_recovery		dropdown	Understanding	1, Almost None | 2, Very Poor | 3, Fair | 4, Functional | 5, Good | 6, Very Good | 7, Like Native Speaker
**language_otherhome3_average**	day_1_recovery		dropdown	Average	1, Almost None | 2, Very Poor | 3, Fair | 4, Functional | 5, Good | 6,

					Very Good | 7, Like Native Speaker
**language_otherhome4_speaking**	day_1_recovery	[language_otherhome_4]	dropdown	Speaking	1, Almost None | 2, Very Poor | 3, Fair | 4, Functional | 5, Good | 6, Very Good | 7, Like Native Speaker
**language_otherhome4_reading**	day_1_recovery		dropdown	Reading	1, Almost None | 2, Very Poor | 3, Fair | 4, Functional | 5, Good | 6, Very Good | 7, Like Native Speaker
**language_otherhome4_writing**	day_1_recovery		dropdown	Writing	1, Almost None | 2, Very Poor | 3, Fair | 4, Functional | 5, Good | 6, Very Good | 7, Like Native Speaker
**language_otherhome4_understanding**	day_1_recovery		dropdown	Understanding	1, Almost None | 2, Very Poor | 3, Fair | 4, Functional | 5, Good | 6, Very Good | 7, Like Native Speaker
**language_otherhome4_average**	day_1_recovery		dropdown	Average	1, Almost None | 2, Very Poor | 3, Fair | 4, Functional | 5, Good | 6, Very Good | 7, Like Native Speaker
**language_otherhome5_speaking**	day_1_recovery	[language_otherhome_5]	dropdown	Speaking	1, Almost None | 2, Very Poor | 3, Fair | 4, Functional | 5, Good | 6, Very Good | 7, Like Native Speaker

**language_otherhome5_reading**	day_1_recovery		dropdown	Reading	1, Almost None | 2, Very Poor | 3, Fair | 4, Functional | 5, Good | 6, Very Good | 7, Like Native Speaker
**language_otherhome5_writing**	day_1_recovery		dropdown	Writing	1, Almost None | 2, Very Poor | 3, Fair | 4, Functional | 5, Good | 6, Very Good | 7, Like Native Speaker
**language_otherhome5_understanding**	day_1_recovery		dropdown	Understanding	1, Almost None | 2, Very Poor | 3, Fair | 4, Functional | 5, Good | 6, Very Good | 7, Like Native Speaker
**language_otherhome5_average**	day_1_recovery		dropdown	Average	1, Almost None | 2, Very Poor | 3, Fair | 4, Functional | 5, Good | 6, Very Good | 7, Like Native Speaker
**language_other1_speaking**	day_1_recovery	[language_other_1]	dropdown	Speaking	1, Almost None | 2, Very Poor | 3, Fair | 4, Functional | 5, Good | 6, Very Good | 7, Like Native Speaker
**language_other1_reading**	day_1_recovery		dropdown	Reading	1, Almost None | 2, Very Poor | 3, Fair | 4, Functional | 5, Good | 6, Very Good | 7, Like Native Speaker
**language_other1_writing**	day_1_recovery		dropdown	Writing	1, Almost None | 2, Very Poor | 3, Fair | 4, Functional | 5, Good | 6,

					Very Good | 7, Like Native Speaker
**language_other1_understanding**	day_1_recovery		dropdown	Understanding	1, Almost None | 2, Very Poor | 3, Fair | 4, Functional | 5, Good | 6, Very Good | 7, Like Native Speaker
**language_other1_average**	day_1_recovery		dropdown	Average	1, Almost None | 2, Very Poor | 3, Fair | 4, Functional | 5, Good | 6, Very Good | 7, Like Native Speaker
**language_other2_speaking**	day_1_recovery	[language_other_2]	dropdown	Speaking	1, Almost None | 2, Very Poor | 3, Fair | 4, Functional | 5, Good | 6, Very Good | 7, Like Native Speaker
**language_other2_reading**	day_1_recovery		dropdown	Reading	1, Almost None | 2, Very Poor | 3, Fair | 4, Functional | 5, Good | 6, Very Good | 7, Like Native Speaker
**language_other2_writing**	day_1_recovery		dropdown	Writing	1, Almost None | 2, Very Poor | 3, Fair | 4, Functional | 5, Good | 6, Very Good | 7, Like Native Speaker
**language_other2_understanding**	day_1_recovery		dropdown	Understanding	1, Almost None | 2, Very Poor | 3, Fair | 4, Functional | 5, Good | 6, Very Good | 7, Like Native Speaker

**language_other2_average**	day_1_recovery		dropdown	Average	1, Almost None | 2, Very Poor | 3, Fair | 4, Functional | 5, Good | 6, Very Good | 7, Like Native Speaker
**language_other3_speaking**	day_1_recovery	[language_other_3]	dropdown	Speaking	1, Almost None | 2, Very Poor | 3, Fair | 4, Functional | 5, Good | 6, Very Good | 7, Like Native Speaker
**language_other3_reading**	day_1_recovery		dropdown	Reading	1, Almost None | 2, Very Poor | 3, Fair | 4, Functional | 5, Good | 6, Very Good | 7, Like Native Speaker
**language_other3_writing**	day_1_recovery		dropdown	Writing	1, Almost None | 2, Very Poor | 3, Fair | 4, Functional | 5, Good | 6, Very Good | 7, Like Native Speaker
**language_other3_understanding**	day_1_recovery		dropdown	Understanding	1, Almost None | 2, Very Poor | 3, Fair | 4, Functional | 5, Good | 6, Very Good | 7, Like Native Speaker
**language_other3_average**	day_1_recovery		dropdown	Average	1, Almost None | 2, Very Poor | 3, Fair | 4, Functional | 5, Good | 6, Very Good | 7, Like Native Speaker
**language_other4_speaking**	day_1_recovery	[language_other_4]	dropdown	Speaking	1, Almost None | 2, Very Poor | 3, Fair | 4, Functional | 5, Good | 6,

					Very Good | 7, Like Native Speaker
**language_other4_reading**	day_1_recovery		dropdown	Reading	1, Almost None | 2, Very Poor | 3, Fair | 4, Functional | 5, Good | 6, Very Good | 7, Like Native Speaker
**language_other4_writing**	day_1_recovery		dropdown	Writing	1, Almost None | 2, Very Poor | 3, Fair | 4, Functional | 5, Good | 6, Very Good | 7, Like Native Speaker
**language_other4_understanding**	day_1_recovery		dropdown	Understanding	1, Almost None | 2, Very Poor | 3, Fair | 4, Functional | 5, Good | 6, Very Good | 7, Like Native Speaker
**language_other4_average**	day_1_recovery		dropdown	Average	1, Almost None | 2, Very Poor | 3, Fair | 4, Functional | 5, Good | 6, Very Good | 7, Like Native Speaker
**language_other5_speaking**	day_1_recovery	[language_other_5]	dropdown	Speaking	1, Almost None | 2, Very Poor | 3, Fair | 4, Functional | 5, Good | 6, Very Good | 7, Like Native Speaker
**language_other5_reading**	day_1_recovery		dropdown	Reading	1, Almost None | 2, Very Poor | 3, Fair | 4, Functional | 5, Good | 6, Very Good | 7, Like Native Speaker

**language_other5_writing**	day_1_recovery		dropdown	Writing	1, Almost None | 2, Very Poor | 3, Fair | 4, Functional | 5, Good | 6, Very Good | 7, Like Native Speaker
**language_other5_understanding**	day_1_recovery		dropdown	Understanding	1, Almost None | 2, Very Poor | 3, Fair | 4, Functional | 5, Good | 6, Very Good | 7, Like Native Speaker
**language_other5_average**	day_1_recovery		dropdown	Average	1, Almost None | 2, Very Poor | 3, Fair | 4, Functional | 5, Good | 6, Very Good | 7, Like Native Speaker
**education_age**	day_1_recovery	Education	text	1. At what age did you start school?	
**education_1_dk**	day_1_recovery		checkbox	Don't know	1, Don't know
**education_high_level**	day_1_recovery		dropdown	2. What was the highest level of school that you completed?	0, None | 1, Pre K | 2, Kindergarten | 3, 1st | 4, 2nd | 5, 3rd | 6, 4th | 7, 5th | 8, 6th | 9, 7th | 10, 8th | 11, 9th | 12, 10th | 13, 11th | 14, 12th
**education_school_level**	day_1_recovery		checkbox	3.Please select all school levels you have completed:	1, Primary | 2, Junior High school / Middle School | 3, High School | 4, College/ University | 5, Professional or Technical School
**education_school_level_1**	day_1_recovery		dropdown	How many Primary schools did you attend?	1,1 | 2,2 | 3,3 | 4,4

**school_level_1_city**	day_1_recovery		text	School 1 City:	
**school_level_1_st**	day_1_recovery		text	School 1 State:	
**school_level_1_country**	day_1_recovery		text	School 1 Country:	
**school_level_1_city2**	day_1_recovery		text	School 2 City:	
**school_level_1_st2**	day_1_recovery		text	School 2 State:	
**school_level_1_country2**	day_1_recovery		text	School 2 Country:	
**school_level_1_city3**	day_1_recovery		text	School 3 City:	
**school_level_1_st3**	day_1_recovery		text	School 3 State:	
**school_level_1_country3**	day_1_recovery		text	School 3 Country:	
**school_level_1_city4**	day_1_recovery		text	School 4 City:	
**school_level_1_st4**	day_1_recovery		text	School 4 State:	
**school_level_1_country4**	day_1_recovery		text	School 4 Country:	
**education_school_level_2**	day_1_recovery		dropdown	How many Junior High school/middle schools did you attend?	1,1 | 2,2 | 3,3 | 4,4
**school_level_2_city**	day_1_recovery		text	School 1 City:	
**school_level_2_st**	day_1_recovery		text	School 1 State:	
**school_level_2_country**	day_1_recovery		text	School 1 Country:	

**school_level_2_city2**	day_1_recovery		text	School 2 City:	
**school_level_2_st2**	day_1_recovery		text	School 2 State:	
**school_level_2_country2**	day_1_recovery		text	School 2 Country:	
**school_level_2_city3**	day_1_recovery		text	School 3 City:	
**school_level_2_st3**	day_1_recovery		text	School 3 State:	
**school_level_2_country3**	day_1_recovery		text	School 3 Country:	
**school_level_2_city4**	day_1_recovery		text	School 4 City:	
**school_level_2_st4**	day_1_recovery		text	School 4 State:	
**school_level_2_country4**	day_1_recovery		text	School 4 Country:	
**education_school_level_3**	day_1_recovery		dropdown	How many high schools did you attend?	1,1 | 2,2 | 3,3 | 4,4
**school_level_3_city**	day_1_recovery		text	School 1 City:	
**school_level_3_st**	day_1_recovery		text	School 1 State:	
**school_level_3_country**	day_1_recovery		text	School 1 Country:	
**school_level_3_city2**	day_1_recovery		text	School 2 City:	
**school_level_3_st2**	day_1_recovery		text	School 2 State:	
**school_level_3_country2**	day_1_recovery		text	School 2 Country:	
**school_level_3_city3**	day_1_recovery		text	School 3 City:	

**school_level_3_st3**	day_1_recovery		text	School 3 State:	
**school_level_3_country3**	day_1_recovery		text	School 3 Country:	
**school_level_3_city4**	day_1_recovery		text	School 4 City:	
**school_level_3_st4**	day_1_recovery		text	School 4 State:	
**school_level_3_country4**	day_1_recovery		text	School 4 Country:	
**education_school_level_4**	day_1_recovery		dropdown	How many university/college did you attend?	1,1 | 2,2 | 3,3 | 4,4
**school_level_4_city**	day_1_recovery		text	School 1 City:	
**school_level_4_st**	day_1_recovery		text	School 1 State:	
**school_level_4_country**	day_1_recovery		text	School 1 Country:	
**school_level_4_degree**	day_1_recovery		text	School Degree Attained:	
**school_level_4_city2**	day_1_recovery		text	School 2 City:	
**school_level_4_st2**	day_1_recovery		text	School 2 State:	
**school_level_4_country2**	day_1_recovery		text	School 2 Country:	
**school_level_4_degree2**	day_1_recovery		text	School Degree Attained:	
**school_level_4_city3**	day_1_recovery		text	School 3 City:	
**school_level_4_st3**	day_1_recovery		text	School 3 State:	

**school_level_4_country3**	day_1_recovery		text	School 3 Country:	
**school_level_4_degree3**	day_1_recovery		text	School Degree Attained:	
**school_level_4_city4**	day_1_recovery		text	School 4 City:	
**school_level_4_st4**	day_1_recovery		text	School 4 State:	
**school_level_4_country4**	day_1_recovery		text	School 4 Country:	
**school_level_4_degree4**	day_1_recovery		text	School Degree Attained:	
**education_school_level_5**	day_1_recovery		dropdown	How many Professional or Technical School did you attend?	1,1 | 2,2 | 3,3 | 4,4
**school_level_5_city**	day_1_recovery		text	School 1 City:	
**school_level_5_st**	day_1_recovery		text	School 1 State:	
**school_level_5_country**	day_1_recovery		text	School 1 Country:	
**school_level_5_degree**	day_1_recovery		text	School Degree Attained:	
**school_level_5_city2**	day_1_recovery		text	School 2 City:	
**school_level_5_st2**	day_1_recovery		text	School 2 State:	
**school_level_5_country2**	day_1_recovery		text	School 2 Country:	
**school_level_5_degree2**	day_1_recovery		text	School Degree Attained:	
**school_level_5_city3**	day_1_recovery		text	School 3 City:	

**school_level_5_st3**	day_1_recovery		text	School 3 State:	
**school_level_5_degree3**	day_1_recovery		text	School Degree Attained:	
**school_level_5_country3**	day_1_recovery		text	School 3 Country:	
**school_level_5_city4**	day_1_recovery		text	School 4 City:	
**school_level_5_st4**	day_1_recovery		text	School 4 State:	
**school_level_5_country4**	day_1_recovery		text	School 4 Country:	
**school_level_5_degree5**	day_1_recovery		text	School Degree Attained:	
**education_noschool_reason**	day_1_recovery		checkbox	4. Why didn't you continue school after the [education_high_level] grade? What did you do after you left school?	1, To work (inside or outside the home) | 2, Moved | 3, Financial reasons (e.g. school/books/uniforms too expensive) | 4, School too far away | 5, Difficulty learning in school | 6, Other (e.g. marriage, parents did not allow to continue, illness)
**education_noschool_reason_2**	day_1_recovery		checkbox	4. If 0 (zero) years, Why didn't you attend school?	1, To work (inside or outside the home) | 2, Moved | 3, Financial reasons (e.g. school/books/uniforms too expensive) | 4, School too far away | 5, Difficulty learning in school | 6, Other (e.g. marriage, parents did not allow to continue, illness)

**education_noschool_reason_other**	day_1_recovery		text	Other (e.g. marriage, parents did not allow to continue, illness)	
**education_ged**	day_1_recovery		yes/no	5, Did you obtain a GED?	

2.2.4 Day 1 Hotel

Redcap Form Name: Day 1 Hotel

Questionnaire Name	Title	Variable Name
2.2.4.1 Modified Differential Emotions Scale	Modified Differential Emotions Scale (mDES)	mdes
2.2.4.2 My Daily Stress	My Daily Stres (DISE)	dise
2.2.4.3 Perceived Age	Perceived Age in Years (Perc_age)	perc_age
2.2.4.4 Perceived Social Status	MacArthur Ladder (ML)	ml
2.2.4.5 Morningness	Morningness and Eveningness Questionnaire (MEQ)	meq
2.2.4.6 Sleep	Pittsburgh Sleep Quality Index (PSQI)	psqi
2.2.4.7 Fatigue	Pittsburgh Fatigability Scale (PFS)	pfsq
2.2.4.8 Social Support	Social Support Questionnaire (SSQ)	ssq
2.2.4.9 Lifetime Stressor Exposure	Stress and Adversity Inventory for Adults (STRAIN)	strain

Variable / Field Name	Form Name	Section Header	Field Type	Field Label	Choices, Calculations, OR Slider Labels
**education_ged_year**	day_1_recovery		text	Year:	
**meq_1**	day_1_hotel	Morningness	descriptive	1. Considering only your own "feeling best" rhythm, at what time would you get up if you were entirely free to plan your day?	

**meq_1_h**	day_1_hotel		dropdown	Hour:	5, 5AM | 6, 6AM | 7, 7AM | 8, 8AM | 9, 9AM | 10, 10AM | 11, 11AM | 12, 12PM
**meq_1_m**	day_1_hotel		dropdown	Minutes:	0, 0 | 1, 1 | 2, 2 | 3, 3 | 4, 4 | 5, 5 | 6, 6 | 7, 7 | 8, 8 | 9, 9 | 10, 10 | 11, 11 | 12, 12 | 13, 13 | 14, 14 | 15, 15 | 16, 16 | 17, 17 | 18, 18 | 19, 19 | 20, 20 | 21, 21 | 22, 22 | 23, 23 | 24, 24 | 25, 25 | 26, 26 | 27, 27 | 28, 28 | 29, 29 | 30, 30 | 31, 31 | 32, 32 | 33, 33 | 34, 34 | 35, 35 | 36, 36 | 37, 37 | 38, 38 | 39, 39 | 40, 40 | 41, 41 | 42, 42 | 43, 43 | 44, 44 | 45, 45 | 46, 46 | 47, 47 | 48, 48 | 49, 49 | 50, 50 | 51, 51 | 52, 52 | 53, 53 | 54, 54 | 55, 55 | 56, 56 | 57, 57 | 58, 58 | 59, 59 | 60, 60
**meq_2**	day_1_hotel		descriptive	2. Considering only your own "feeling best" rhythm, at what time would you go to bed if you were entirely free to plan your evening?	
**meq_2_h**	day_1_hotel		dropdown	Hour:	8, 8PM | 9, 9PM | 10, 10PM | 11, 11PM | 12, 12AM | 1, 1AM | 2, 2AM | 3, 3AM
**meq_2_m**	day_1_hotel		dropdown	Minutes:	0, 0 | 1, 1 | 2, 2 | 3, 3 | 4, 4 | 5, 5 | 6, 6 | 7, 7 | 8, 8 | 9, 9 | 10, 10 | 11, 11 | 12, 12 | 13, 13 | 14, 14 | 15, 15 | 16, 16 | 17, 17 | 18, 18 | 19, 19 | 20, 20 | 21, 21 | 22, 22 | 23, 23 | 24, 24 | 25, 25 | 26, 26 | 27, 27 | 28, 28 | 29, 29 | 30, 30 | 31, 31 | 32, 32 | 33, 33 | 34, 34 | 35, 35 | 36, 36 | 37, 37 | 38, 38 | 39, 39

					| 40, 40 | 41, 41 | 42, 42 | 43, 43 | 44, 44 | 45, 45 | 46, 46 | 47, 47 | 48, 48 | 49, 49 | 50, 50 | 51, 51 | 52, 52 | 53, 53 | 54, 54 | 55, 55 | 56, 56 | 57, 57 | 58, 58 | 59, 59 | 60, 60
**meq_3**	day_1_hotel		radio	3. If there is a specific time at which you have to get up in the morning, to what extent are your dependent on being woken up by an alarm clock?	4, Not at all dependent | 3, Slightly dependent | 2, Fairly dependent | 1, Very dependent
**meq_4**	day_1_hotel		radio	4. Assumming adequate environmental conditions, how easy do you find getting up in the morning?	1, Not at all easy | 2, Not very easy | 3, Fairly easy | 4, Very easy
**meq_5**	day_1_hotel		radio	5. How do you feel during the first half hour after having woken in the mornings?	4, Not at all alert | 3, Slightly alert | 2, Fairly alert | 1, Very alert
**meq_6**	day_1_hotel		radio	6. How is your appetite during the first half-hour after having woken in the morning?	1, Very poor | 2, Fairly poor | 3, Fairly good | 4, Very good
**meq_7**	day_1_hotel		radio	7. During the first half-hour after having woken in the morning, how tired do you feel?	1, Very tired | 2, Fairly tired | 3, Fairly refreshed | 4, Very refreshed
**meq_8**	day_1_hotel		radio	8. When you have no commitments the next day, at what time do you go to bed compared to your usual bedtime?	4, Seldom or never later | 3, Less than one hour later | 2, 1-2 hours later | 1, More than two hours later

**meq_9**	day_1_hotel		radio	9. Consider this situation: You have decided to engage in some physical exercise. A friend suggests that you do this one hour twice a week and the best time for him is between 7-8am. Bearing in mind nothing else but your own "feeling best" rhythm, how do you think you would perform?	4, Would be on good form | 3, Would be on reasonable form | 2, Would find it difficult | 1, Would find it very difficult
**meq_10**	day_1_hotel		descriptive	10. At what time in the evening do you feel tired and as a result in need of sleep?	
**meq_10_h**	day_1_hotel		dropdown	Hour:	8, 8PM | 9, 9PM | 10, 10PM | 11, 11PM | 12, 12AM | 1, 1AM | 2, 2AM | 3, 3AM
**meq_10_m**	day_1_hotel		dropdown	Minutes:	0, 0 | 1, 1 | 2, 2 | 3, 3 | 4, 4 | 5, 5 | 6, 6 | 7, 7 | 8, 8 | 9, 9 | 10, 10 | 11, 11 | 12, 12 | 13, 13 | 14, 14 | 15, 15 | 16, 16 | 17, 17 | 18, 18 | 19, 19 | 20, 20 | 21, 21 | 22, 22 | 23, 23 | 24, 24 | 25, 25 | 26, 26 | 27, 27 | 28, 28 | 29, 29 | 30, 30 | 31, 31 | 32, 32 | 33, 33 | 34, 34 | 35, 35 | 36, 36 | 37, 37 | 38, 38 | 39, 39 | 40, 40 | 41, 41 | 42, 42 | 43, 43 | 44, 44 | 45, 45 | 46, 46 | 47, 47 | 48, 48 | 49, 49 | 50, 50 | 51, 51 | 52, 52 | 53, 53 | 54, 54 | 55, 55 | 56, 56 | 57, 57 | 58, 58 | 59, 59 | 60, 60

**meq_11**	day_1_hotel		dropdown	11. You wish to be at your peak performance for a test which you know is going to be mentally exhausting and lasting for two hours. You are entirely free to plan your day and considering only your own "feeling best" rhythm which ONE of the four testing times would you choose?	6, 8:00-10:00 AM | 4, 11:00 - 1:00PM | 2, 3:00-5:00 PM |0,7:00-9:00 PM
**meq_12**	day_1_hotel		dropdown	12. If you went to bed at 11:00PM at what level of tiredness would you be?	0, Not at all tired| 2, A little tired| 3, Fairly Tired | 4, Very tired
**meq_13**	day_1_hotel		dropdown	13.Consider this situation: for some reason you have gone to bed several hours later than usual, but there is no need to get up at any particular time the next morning. Which ONE of the following events are you most likely to experience?	4, Will wake up at usual time and will NOT fall asleep. | 3, Will wake up at the usual time and will dose thereafter | 2, Will wake up at usual time but will fall asleep again | 1, Will NOT wake up until later than usual
**meq_14**	day_1_hotel		dropdown	14. Consider this situation: one night you have to remain awake between 4:00 - 6:00 AM in order to carry out a night watch. You have not commements the next day. Which ONE of the following alternative will suit you the best?	1, Would NOT go to bed until watch was over | 2, Would take a nap before and sleep after | 3, Would take a good sleep before and nap after | 4, Would take all sleep before watch

**meq_15**	day_1_hotel		dropdown	15. Consider this situation: you have to do two hours of hard physical work. You are entirely free to paln your day and considering only your own "feeling best" rhythm. Which ONE of the following times would you choose?	4, 8:00-10:00 AM | 3, 11:00 - 1:00PM | 2, 3:00-5:00 PM | 1,7:00-9:00 PM
**meq_16**	day_1_hotel		dropdown	16. Consider this situation: You have to engage in hard physical exercise. A friend suggests that you do this for one hour twice a week and the best time for him is between 10:00 -11:00 PM. Beasting in mind nothing else but your own "feeling best" rhythm how well do you think you would perform?	1, Would be on good form | 2, Would be on reasonable form | 3, Would find it difficult | 4, Would find it very diffeicult
**meq_17**	day_1_hotel		dropdown	17. Suppose that you can choose your own work hours. Assume that you worked a FIVE hour day (including breaks) and that your job was interssting and paid by results. Which FIVE Consecutive hours would you select? Hours starting from:	1, 12AM | 1.1, 1AM | 1.01, 2AM | 1.02, 3AM | 5, 4AM | 5.01, 5AM | 5.02, 6AM | 5.03, 7AM | 4, 8AM | 3, 9AM | 3.01, 10AM | 3.02, 11AM | 3.03, 12 PM | 3.04, 1PM | 2, 2PM | 2.01, 3PM | 2.03, 4PM | 1.03, 5PM | 1.04, 6PM | 1.05, 7PM | 1.06, 8PM | 1.07, 9PM | 1.08, 10 PM | 1.09, 11PM
**meq_17_end**	day_1_hotel		dropdown	Hours end at:	1, 12AM | 1.1, 1AM | 1.01, 2AM | 1.02, 3AM | 5, 4AM | 5.01, 5AM | 5.02, 6AM | 5.03, 7AM | 4, 8AM | 3, 9AM | 3.01,

					10AM | 3.02, 11AM | 3.03, 12 PM | 3.04, 1PM | 2, 2PM | 2.01, 3PM | 2.03, 4PM | 1.03, 5PM | 1.04, 6PM | 1.05, 7PM | 1.06, 8PM | 1.07, 9PM | 1.08, 10 PM | 1.09, 11PM
**meq_18**	day_1_hotel		dropdown	18. At what time of the day do you think that you reach your "feeling best" peak?	1, 12AM | 1.1, 1AM| 1.01, 2AM | 1.02, 3AM | 5, 4AM| 5.01, 5AM | 5.02, 6AM | 5.03, 7AM | 4, 8AM| 3, 9AM| 3.01,10AM | 3.02, 11AM | 3.03,12 PM | 2, 2PM | 2.01, 3PM | 2.03, 4PM| 1.03, 5PM | 1.04, 6PM | 1.05, 7PM | 1.06, 8PM | 1.07, 9PM | 1.08, 10 PM | 1.09, 11PM
**meq_19**	day_1_hotel		dropdown	19, One hears abour "morning" and "evening" type of people. Which ONE of these types do you consider yourself to be	6, Definitely a " morning" type | 4, Rather more a " morning" type than an "evening" type| 2, Rather more a "evening" type than an "morning" type|0, Definitely a " evening" type
**howold**	day_1_hotel	How old do you feel?	text	Please enter the age you feel:	
**macarthur_ladder**	day_1_hotel		radio	ladder of life	0, 0 | 1, 1 | 2, 2 | 3, 3 | 4, 4 | 5, 5 | 6, 6 | 7, 7 | 8, 8 | 9, 9 | 10, 10
**psqi_bedtime_1**	day_1_hotel	Sleep Quality<br>Instructions:<br>The following questions relate to your usual sleep habits during the past month only. Your answers should indicate the most accurate reply for the majority of days and nights in the past month.	text	1. During the past month, what time have you usually gone to bed at night? Usual bedtime:	

**psqi_fall_asleep_1**	day_1_hotel		text	2. During the past month, how long (in minutes) has it usually taken you to fall asleep each night? Number of minutes:	
**psqi_wake_time_1**	day_1_hotel		text	3. During the past month, what time have you usually gotten up in the morning? Usually getting up time:	
**psqi_actual_sleep_1**	day_1_hotel		text	4. During the past month, how many hours of actual sleep did you get at night? (This may be different than the number of hours you spent in bed.) Hours of sleep per night:	
**psqi_30_minutes_1**	day_1_hotel		radio	5a. During the past month, how often have you had trouble sleeping because you cannot get to sleep within 30 minutes?	0, Not during the past month | 1, Less than once a week | 2, Once or twice a week | 3, Three or more times a week
**psqi_middle_early_1**	day_1_hotel		radio	5b. During the past month, how often have you had trouble sleeping because you wake up in the middle of the night or early morning?	0, Not during the past month | 1, Less than once a week | 2, Once or twice a week | 3, Three or more times a week
**psqi_bathroom_1**	day_1_hotel		radio	5c. During the past month, how often have you had trouble sleeping because you have to get up to use the bathroom?	0, Not during the past month | 1, Less than once a week | 2, Once or twice a week | 3, Three or more times a week

**psqi_breathe_1**	day_1_hotel		radio	5d. During the past month, how often have you had trouble sleeping because you cannot breathe comfortably?	0, Not during the past month | 1, Less than once a week | 2, Once or twice a week | 3, Three or more times a week
**psqi_cough_snore_1**	day_1_hotel		radio	5e. During the past month, how often have you had trouble sleeping because you cough or snore loudly?	0, Not during the past month | 1, Less than once a week | 2, Once or twice a week | 3, Three or more times a week
**psqi_cold_1**	day_1_hotel		radio	5f. During the past month, how often have you had trouble sleeping because you feel too cold?	0, Not during the past month | 1, Less than once a week | 2, Once or twice a week | 3, Three or more times a week
**psqi_hot_1**	day_1_hotel		radio	5g. During the past month, how often have you had trouble sleeping because you feel too hot?	0, Not during the past month | 1, Less than once a week | 2, Once or twice a week | 3, Three or more times a week
**psqi_dreams_1**	day_1_hotel		radio	5h. During the past month, how often have you had trouble sleeping because you had bad dreams?	0, Not during the past month | 1, Less than once a week | 2, Once or twice a week | 3, Three or more times a week
**psqi_pain_1**	day_1_hotel		radio	5i. During the past month, how often have you had trouble sleeping because you have pain?	0, Not during the past month | 1, Less than once a week | 2, Once or twice a week | 3, Three or more times a week
**psqi_other_reason_1**	day_1_hotel		text	5j. Other reason(s) you have had trouble sleeping during the past month (please specify reason(s))	

**psqi_other_reason_freq_1**	day_1_hotel		radio	5ja. How often during the past month have you had trouble sleeping because of this?	0, Not during the past month | 1, Less than once a week | 2, Once or twice a week | 3, Three or more times a week
**psqi_quality_1**	day_1_hotel		radio	6. During the past month, how would you rate your sleep quality overall?	0, Very good | 1, Fairly good | 2, Fairly bad | 3, Very bad
**psqi_medicine_1**	day_1_hotel		radio	7. During the past month, how often have you taken medicine to help you sleep (prescribed or over-the-counter)?	0, Not during the past month | 1, Less than once a week | 2, Once or twice a week | 3, Three or more times a week
**psqi_trouble_awake_1**	day_1_hotel		radio	8. During the past month, how often have you had trouble staying awake while driving, eating meals, or engaging in social activity?	0, Not during the past month | 1, Less than once a week | 2, Once or twice a week | 3, Three or more times a week
**psqi_enthusiasm_1**	day_1_hotel		radio	9. During the past month, how much of a problem has it been for you to keep enough enthusiasm to get things done?	0, No problem at all | 1, Only a very slight problem | 2, Somewhat of a problem | 3, A very big problem
**psqi_10_part**	day_1_hotel		radio	Do you have a bed partner or roommate?	1, No bed partner or roommate | 2, Partner/roommate in other room | 3, Partner/roommate in same room, but not same bed | 4, Partner in same bed
**pqsi_10a**	day_1_hotel	Ask your partner/roommate if you have had the following in the past month:	checkbox	Loud snoring?	0, None during the past month 1, Less than once a week | 2,

					Once or twice a week | 3, Three or more times a week
**pqsi_10b**	day_1_hotel		checkbox	Long pauses between breaths while asleep?	0, None during the past month| 1, Less than once a week | 2, Once or twice a week | 3, Three or more times a week
**pqsi_10c**	day_1_hotel		checkbox	Legs twitching or jerking while you sleep?	0, None during the past month| 1, Less than once a week | 2, Once or twice a week | 3, Three or more times a week
**pqsi_10d**	day_1_hotel		checkbox	Episodes of disorientation or confusion during sleep?	0, None during the past month| 1, Less than once a week | 2, Once or twice a week | 3, Three or more times a week
**pqsi_10e_describe**	day_1_hotel		text	Please describe the other restlessness that you have had while sleeping in the past month.	
**pqsi_10e**	day_1_hotel		checkbox	Other restlessness?	0, None during the past month| 1, Less than once a week | 2, Once or twice a week | 3, Three or more times a week
**pfs_**	day_1_hotel	Fatigue<br>Instructions:<br>The following questions ask you to indicate the level of physical and mental fatigue (i.e., tiredness, exhaustion) you expect or imagine you would feel immediately after completing each of the ten listed activities. For each activity (a-j) please circle responses for both physical and mental fatigue between 0 and 5, where '0'	descriptive	Examples:	

		equals no fatigue at all and '5' equals extreme fatigue.<br>In the last column indicate if you have done the activity in the past month. If you answer 'No,' please make your best guess for the fatigue questions (see Example 2 below). Please fill out all three columns for every activity even for those that you do not do. Also pay careful attention to the duration (e.g., 30 minutes) and intensity (e.g., moderate, brisk) of each activity.			
**pfsq_1**	day_1_hotel	--|------------Physical Fatigue---------|-----------Mental Fatigue----------- |Past month?	checkbox	Leisurely walk for 30 minutes	0, No Fatigue 0 | 1, 1 | 2, 2 | 3, 3 | 4, 4 | 5, Extreme Fatigue 5 | 10, No Fatigue 0 | 11, 1 | 12, 2 | 13, 3 | 14, 4 | 15, Extreme Fatigue 5 | 16, Yes | 17, No
**pfsq_2**	day_1_hotel		checkbox	Brisk or fast walk for 1 hour	0, No Fatigue 0 | 1, 1 | 2, 2 | 3, 3 | 4, 4 | 5, Extreme Fatigue 5 | 10, No Fatigue 0 | 11, 1 | 12, 2 | 13, 3 | 14, 4 | 15, Extreme Fatigue 5 | 16, Yes | 17, No
**pfsq_3**	day_1_hotel		checkbox	Light household activity for 1 hour (cleaning, cooking, dusting, straightening up, baking, making beds, dishwashing,watering plants)	0, No Fatigue 0 | 1, 1 | 2, 2 | 3, 3 | 4, 4 | 5, Extreme Fatigue 5 | 10, No Fatigue 0 | 11, 1 | 12, 2 | 13, 3 | 14, 4 | 15, Extreme Fatigue 5 | 16, Yes | 17, No
**pfsq_4**	day_1_hotel		checkbox	Heavy gardening or outdoor work for 1 hour(mowing (push), raking, weeding, planting, shoveling snow)	0, No Fatigue 0 | 1, 1 | 2, 2 | 3, 3 | 4, 4 | 5, Extreme Fatigue 5 | 10, No Fatigue 0 | 11, 1 | 12, 2 | 13, 3 | 14, 4 | 15, Extreme Fatigue 5 | 16, Yes | 17, No

**pfsq_5**	day_1_hotel		checkbox	Watching TV for 2 hours	0, No Fatigue 0 | 1, 1 | 2, 2 | 3, 3 | 4, 4 | 5, Extreme Fatigue 5 | 10, No Fatigue 0 | 11, 1 | 12, 2 | 13, 3 | 14, 4 | 15, Extreme Fatigue 5 | 16, Yes | 17, No
**pfsq_6**	day_1_hotel		checkbox	Sitting quietly for 1 hour	0, No Fatigue 0 | 1, 1 | 2, 2 | 3, 3 | 4, 4 | 5, Extreme Fatigue 5 | 10, No Fatigue 0 | 11, 1 | 12, 2 | 13, 3 | 14, 4 | 15, Extreme Fatigue 5 | 16, Yes | 17, No
**pfsq_7**	day_1_hotel		checkbox	Moderate- to high-intensity strength training for 30 minutes (hand-held weights or machines greater than 5 lbs, push-ups)	0, No Fatigue 0 | 1, 1 | 2, 2 | 3, 3 | 4, 4 | 5, Extreme Fatigue 5 | 10, No Fatigue 0 | 11, 1 | 12, 2 | 13, 3 | 14, 4 | 15, Extreme Fatigue 5 | 16, Yes | 17, No
**pfsq_8**	day_1_hotel		checkbox	Participating in a social activity for 1 hour (party, dinner, senior center, gathering with family/friends, playing cards, bridge)	0, No Fatigue 0 | 1, 1 | 2, 2 | 3, 3 | 4, 4 | 5, Extreme Fatigue 5 | 10, No Fatigue 0 | 11, 1 | 12, 2 | 13, 3 | 14, 4 | 15, Extreme Fatigue 5 | 16, Yes | 17, No
**pfsq_9**	day_1_hotel		checkbox	Hosting a social event for 1 hour (not including preparation time)	0, No Fatigue 0 | 1, 1 | 2, 2 | 3, 3 | 4, 4 | 5, Extreme Fatigue 5 | 10, No Fatigue 0 | 11, 1 | 12, 2 | 13, 3 | 14, 4 | 15, Extreme Fatigue 5 | 16, Yes | 17, No
**pfsq_10**	day_1_hotel		checkbox	High-intensity activity for 30 minutes (jogging, hiking, biking, swimming, racquet sports, aerobic machines, dancing, Zumba)	0, No Fatigue 0 | 1, 1 | 2, 2 | 3, 3 | 4, 4 | 5, Extreme Fatigue 5 | 10, No Fatigue 0 | 11, 1 | 12, 2 | 13, 3 | 14, 4 | 15, Extreme Fatigue 5 | 16, Yes | 17, No

**ssq_1_help**	day_1_hotel	Social Support Questionnaire 6 (SSQ6)<br>Instructions:<br>The following questions ask about people in your environment who provide you with help or support. Each question has two parts: For the first part, list all the people you know, excluding yourself, whom you can count on for help or support in the manner described. Give the person's initials and their relationship to you. Do not list more than one person in each blank.For the second part, indicate how satisfied you are with the overall support you have.If you have no support for a question, type the words "No one" in blank "a," but still rate your level of satisfaction.	text	1. Whom can you really count on to be dependable when you need help?<br>1)	
**ssq_1b**	day_1_hotel		text	2)	
**ssq_1c**	day_1_hotel		text	3)	
**ssq_1d**	day_1_hotel		text	4)	
**ssq_1e**	day_1_hotel		text	5)	
**ssq_1f**	day_1_hotel		text	6)	
**ssq_1g**	day_1_hotel		text	7)	
**ssq_1h**	day_1_hotel		text	8)	
**ssq_1i**	day_1_hotel		text	9)	
**ssq_1_no**	day_1_hotel		checkbox		1, No One

**ssq_1_help_satis**	day_1_hotel		radio	How satisfied?	6, Very satisfied | 5, Fairly satisfied | 4, A little satisfied | 3, A little dissatisfied | 2, Fairly dissatisfied | 1, Very dissatisfied
**ssq_2_relaxed**	day_1_hotel		text	2. Whom can you really count on to help you feel more relaxed when you are under pressure or tense?<br>1)	
**ssq_2b**	day_1_hotel		text	2)	
**ssq_2c**	day_1_hotel		text	3)	
**ssq_2d**	day_1_hotel		text	4)	
**ssq_2e**	day_1_hotel		text	5)	
**ssq_2f**	day_1_hotel		text	6)	
**ssq_2g**	day_1_hotel		text	7)	
**ssq_2h**	day_1_hotel		text	8)	
**ssq_2i**	day_1_hotel		text	9)	
**ssq_2_no**	day_1_hotel		checkbox		1, No One
**ssq_2_relaxed_satis**	day_1_hotel		radio	How satisfied?	6, Very satisfied | 5, Fairly satisfied | 4, A little satisfied | 3, A little dissatisfied | 2, Fairly dissatisfied | 1, Very dissatisfied

**ssq_3_acceptance**	day_1_hotel		text	3. Who accepts you totally, including both your worst and your best points?<br>1)	
**ssq_3b**	day_1_hotel		text	2)	
**ssq_3c**	day_1_hotel		text	3)	
**ssq_3d**	day_1_hotel		text	4)	
**ssq_3e**	day_1_hotel		text	5)	
**ssq_3f**	day_1_hotel		text	6)	
**ssq_3g**	day_1_hotel		text	7)	
**ssq_3h**	day_1_hotel		text	8)	
**ssq_3i**	day_1_hotel		text	9)	
**ssq_3_no**	day_1_hotel		checkbox		1, No One
**ssq_3_acceptance_satis**	day_1_hotel		radio	How satisfied?	6, Very satisfied | 5, Fairly satisfied | 4, A little satisfied | 3, A little dissatisfied | 2, Fairly dissatisfied | 1, Very dissatisfied
**ssq_4_care**	day_1_hotel		text	4. Whom can you really count on to care about you, regardless of what is happening to you?<br>1)	

**ssq_4b**	day_1_hotel		text	2)	
**ssq_4c**	day_1_hotel		text	3)	
**ssq_4d**	day_1_hotel		text	4)	
**ssq_4e**	day_1_hotel		text	5)	
**ssq_4f**	day_1_hotel		text	6)	
**ssq_4g**	day_1_hotel		text	7)	
**ssq_4h**	day_1_hotel		text	8)	
**ssq_4i**	day_1_hotel		text	9)	
**ssq_4_no**	day_1_hotel		checkbox		1, No One
**ssq_4_care_satis**	day_1_hotel		radio	How satisfied?	6, Very satisfied | 5, Fairly satisfied | 4, A little satisfied | 3, A little dissatisfied | 2, Fairly dissatisfied | 1, Very dissatisfied
**ssq_5_feelbetter**	day_1_hotel		text	5. Whom can you really count on to help you feel better when you are feeling generally down-in-the- dumps? 1)	
**ssq_5b**	day_1_hotel		text	2)	
**ssq_5c**	day_1_hotel		text	3)	

**ssq_5d**	day_1_hotel		text	4)	
**ssq_5e**	day_1_hotel		text	5)	
**ssq_5f**	day_1_hotel		text	6)	
**ssq_5g**	day_1_hotel		text	7)	
**ssq_5h**	day_1_hotel		text	8)	
**ssq_5i**	day_1_hotel		text	9)	
**ssq_5_no**	day_1_hotel		checkbox		1, No One
**ssq_5_feelbetter_satis**	day_1_hotel		radio	How satisfied?	6, Very satisfied | 5, Fairly satisfied | 4, A little satisfied | 3, A little dissatisfied | 2, Fairly dissatisfied | 1, Very dissatisfied
**ssq_6console**	day_1_hotel		text	6. Whom can you count on to console you when you are very upset?<br>1)	
**ssq_6b**	day_1_hotel		text	2)	
**ssq_6c**	day_1_hotel		text	3)	
**ssq_6d**	day_1_hotel		text	4)	
**ssq_6e**	day_1_hotel		text	5)	
**ssq_6f**	day_1_hotel		text	6)	

**ssq_6g**	day_1_hotel		text	7)	
**ssq_6h**	day_1_hotel		text	8)	
**ssq_6i**	day_1_hotel		text	9)	
**ssq_6_no**	day_1_hotel		checkbox		1, No One
**ssq_6_console_satis**	day_1_hotel		radio	How satisfied?	6, Very satisfied | 5, Fairly satisfied | 4, A little satisfied | 3, A little dissatisfied | 2, Fairly dissatisfied | 1, Very dissatisfied
**mdes_amused_d1**	day_1_hotel	Emotions<br>For each of the emotions listed below, please tell us how much you have felt that emotion this evening. Please choose one of the following answers for each question:<br>0, Not at all | 1, A little bit | 2, Moderately | 3, Quite a bit | 4, Extremely	radio	1. I felt amused, fun-loving, or silly.	0, Not at all | 1, A little bit | 2, Moderately | 3, Quite a bit | 4, Extremely
**mdes_angry_d1**	day_1_hotel		radio	2. I felt angry, irritated, or annoyed.	0, Not at all | 1, A little bit | 2, Moderately | 3, Quite a bit | 4, Extremely
**mdes_ashamed_d1**	day_1_hotel		radio	3. I felt ashamed, humiliated, or disgraced.	0, Not at all | 1, A little bit | 2, Moderately | 3, Quite a bit | 4, Extremely
**mdes_awe_d1**	day_1_hotel		radio	4. I felt awe, wonder, or amazement.	0, Not at all | 1, A little bit | 2, Moderately | 3, Quite a bit | 4, Extremely

**mdes_bored_d1**	day_1_hotel		radio	5. I felt bored, disinterested, uninvolved.	0, Not at all | 1, A little bit | 2, Moderately | 3, Quite a bit | 4, Extremely
**mdes_contemptuous_d1**	day_1_hotel		radio	6. I felt contemptuous, scornful, or disdainful.	0, Not at all | 1, A little bit | 2, Moderately | 3, Quite a bit | 4, Extremely
**mdes_control_d1**	day_1_hotel		radio	7. I felt control, coping well, on top of things.	0, Not at all | 1, A little bit | 2, Moderately | 3, Quite a bit | 4, Extremely
**mdes_disgust_d1**	day_1_hotel		radio	8. I felt disgust, distaste, or revulsion.	0, Not at all | 1, A little bit | 2, Moderately | 3, Quite a bit | 4, Extremely
**mdes_embarrassed_d1**	day_1_hotel		radio	9. I felt embarrassed, self-conscious, or blushing.	0, Not at all | 1, A little bit | 2, Moderately | 3, Quite a bit | 4, Extremely
**mdes_excited_d1**	day_1_hotel		radio	10. I felt excited, eager, enthusiastic.	0, Not at all | 1, A little bit | 2, Moderately | 3, Quite a bit | 4, Extremely
**mdes_grateful_d1**	day_1_hotel		radio	11. I felt grateful, appreciative, or thankful.	0, Not at all | 1, A little bit | 2, Moderately | 3, Quite a bit | 4, Extremely
**mdes_guilty_d1**	day_1_hotel		radio	12. I felt guilty, repentant, or blameworthy.	0, Not at all | 1, A little bit | 2, Moderately | 3, Quite a bit | 4, Extremely
**mdes_hate_d1**	day_1_hotel		radio	13. I felt hatred, distrust, or suspicion.	0, Not at all | 1, A little bit | 2, Moderately | 3, Quite a bit | 4, Extremely

**mdes_hopeful_d1**	day_1_hotel		radio	14. I felt hopeful, optimistic, or encouraged.	0, Not at all | 1, A little bit | 2, Moderately | 3, Quite a bit | 4, Extremely
**mdes_inspired_d1**	day_1_hotel		radio	15. I felt inspired, uplifted, or elevated.	0, Not at all | 1, A little bit | 2, Moderately | 3, Quite a bit | 4, Extremely
**mdes_interested_d1**	day_1_hotel		radio	16. I felt interested, alert, or curious.	0, Not at all | 1, A little bit | 2, Moderately | 3, Quite a bit | 4, Extremely
**mdes_joyful_d1**	day_1_hotel		radio	17. I felt joyful, glad, or happy.	0, Not at all | 1, A little bit | 2, Moderately | 3, Quite a bit | 4, Extremely
**mdes_love_d1**	day_1_hotel		radio	18. I felt love, closeness, or trust.	0, Not at all | 1, A little bit | 2, Moderately | 3, Quite a bit | 4, Extremely
**mdes_proud_d1**	day_1_hotel		radio	19. I felt proud, confident, or self-assured.	0, Not at all | 1, A little bit | 2, Moderately | 3, Quite a bit | 4, Extremely
**mdes_rejected_d1**	day_1_hotel		radio	20. I felt rejected, betrayed, left-behind.	0, Not at all | 1, A little bit | 2, Moderately | 3, Quite a bit | 4, Extremely
**mdes_sad_d1**	day_1_hotel		radio	21. I felt sad, downhearted, or unhappy.	0, Not at all | 1, A little bit | 2, Moderately | 3, Quite a bit | 4, Extremely
**mdes_satisfied_d1**	day_1_hotel		radio	22. I felt satisfied, fulfilled, content.	0, Not at all | 1, A little bit | 2, Moderately | 3, Quite a bit | 4, Extremely

**mdes_scared_d1**	day_1_hotel		radio	23. I felt scared, fearful, or afraid.	0, Not at all | 1, A little bit | 2, Moderately | 3, Quite a bit | 4, Extremely
**mdes_stressed_d1**	day_1_hotel		radio	24. I felt stressed, nervous, or overwhelmed.	0, Not at all | 1, A little bit | 2, Moderately | 3, Quite a bit | 4, Extremely
**mdes_tired_d1**	day_1_hotel		radio	25. I felt tired, sleepy, drowsy.	0, Not at all | 1, A little bit | 2, Moderately | 3, Quite a bit | 4, Extremely
**mdes_sextual_d1**	day_1_hotel		radio	26. I felt sexual, desiring, flirtatious.	0, Not at all | 1, A little bit | 2, Moderately | 3, Quite a bit | 4, Extremely
**mdes_sympathy_d1**	day_1_hotel		radio	27. I felt sympathy, concern, compassion.	0, Not at all | 1, A little bit | 2, Moderately | 3, Quite a bit | 4, Extremely
**dise_stresstime_d1**	day_1_hotel	My daily stress<br>We all have events that happen on a daily basis that are considered stressful. We are interested in one actual event that caused you the MOST stress today. This one event could be something major or something minor. It may involve your spouse, your child/children, something at work, finances, or something else.<br>Using the questions below, please describe, with as many details as possible, the event in your life that caused you the most stress today. We are interested in what actually happened, in other words how the event unfolded.	dropdown	1. At approximately what time of the day did this occur?	0, 12 AM | 1, 1 M | 2, 2 AM | 3, 3AM | 4, 4AM | 5, 5AM | 6, 6AM | 7, 7AM | 8, 8AM | 9, 9AM | 10, 10AM | 11, 11AM | 12, 12PM | 13, 1PM | 14, 2PM | 15, 3PM | 16, 4PM | 17, 5PM | 18, 6PM | 19, 7PM | 20, 8PM | 21, 9PM | 22, 10PM | 23, 11PM

**dise_stresslength_d1**	day_1_hotel		radio	2. How long did this event last?	1, For several minutes | 2, For up to an hour | 3, For several hours | 4,For the rest of the day
**dise_howstressful_d1**	day_1_hotel		radio	3. How stressful do you think the average person would find this event?	0, Not at all stressful | 1, A little stressful | 2, Somewhat stressful | 3, Moderately stressful | 4,Very stressful
**dise_stresstype_d1**	day_1_hotel		checkbox	4. Please review the following list and then select the category or categories from the following list that best describe the stressor described above.	1,Housing | 2, Money/Possessions | 3, Crime / Legal | 4,Your own health | 5,The health/death of a loved one | 6,Caregiving for child/children | 7,Marital/Partner relationship| 8,Other family / Household member relationships| 9, Education | 10,Work | 11,Lack of sleep | 12,Other
**dise_instructions_d1**	day_1_hotel		text	Please describe (in a max of 5 words):	
**dise_stressful_d1**	day_1_hotel		slider	5. How stressful was this situation for you, today, at its peak?	Not at all | Somewhat | Extremely
**dise_angry_d1**	day_1_hotel		slider	6. How angry did you feel at the peak of this stressor?	Not at all | Somewhat | Extremely
**dise_anxious_d1**	day_1_hotel		slider	7. How anxious did you feel at the peak of this stressor?	Not at all | Somewhat | Extremely

**dise_sad_d1**	day_1_hotel		slider	8. How sad did you feel, at the peak of this stressor?	Not at all | Somewhat | Extremely
**dise_shame_d1**	day_1_hotel		slider	9. How much shame did you feel, at the peak of this stressor?	Not at all | Somewhat | Extremely
**dise_length_d1**	day_1_hotel		radio	10. For how long afterward did your negative emotions last?	1, Not at all | 2, For several minutes afterwards| 3,For up to an hour afterwards | 4, For more than an hour | 5,For the rest of the day
**dise_stressprevalence_d1**	day_1_hotel		radio	11. To what extent did you find yourself thinking about this stressful situation in the rest of your day afterward?	0, Not at all | 1, A little| 2, Moderately | 3,Quite a bit | 4, A lot
**dise_control_d1**	day_1_hotel		radio	12. Do you feel that you have control over the stressful situation (not your reaction to it, but the actual situation)?	0, Not at all | 1, A little| 2, Moderately | 3,Quite a bit | 4, A lot
**dise_resources_d1**	day_1_hotel		radio	13. To what extent do you have the resources (emotional, interpersonal, or cognitive) to deal with this situation?	0, Not at all | 1, A little| 2, Moderately | 3,Quite a bit | 4, A lot
**dise_demanding_d1**	day_1_hotel		radio	14. How demanding is it to deal with this situation?	0, Not at all | 1, A little| 2, Moderately | 3,Quite a bit | 4, A lot

**dise_handlestress_d1**	day_1_hotel		radio	15. To what extent do you feel you are able to effectively handle this situation?	0, Not at all | 1, A little| 2, Moderately | 3,Quite a bit | 4, A lot
**strain_id**	strain		text	strain ID	
**datecomp**	strain		text	Date Participant Completed the STRAIN	
**minutestocomplete**	strain		text	Time (Minutes) Taken by the Participant to Complete the STRAIN	
**age**	strain		text	Age	
**sex**	strain		text	Sex	
**phq**	strain		text	Physical Health Complaints/Symptoms	
**k6**	strain		text	Mental Health Complaints/Symptoms	
**stressct**	strain		text	Core: Total Count of Stressors	
**stressth**	strain		text	Core: Total Severity of Stressors	
**evntct**	strain		text	Core: Count of Acute Life Events	

**diffct**	strain		text	Core: Count of Chronic Difficulties	
**evntth**	strain		text	Core: Severity of Acute Life Events	
**diffth**	strain		text	Core: Severity of Chronic Difficulties	
**pnct**	strain		text	Time-Limited: Prenatal - Total Count	
**eatotct**	strain		text	Time-Limited: Early Adversity - Total Count	
**eatotth**	strain		text	Time-Limited: Early Adversity - Total Threat	
**eaevntct**	strain		text	Time-Limited: Early Adversity - Count of Acute Life Events	
**eaevntth**	strain		text	Time-Limited: Early Adversity - Severity of Acute Life Events	
**eadiffct**	strain		text	Time-Limited: Early Adversity - Count of Chronic Difficulties	
**eadiffth**	strain		text	Time-Limited: Early Adversity - Severity of Chronic Difficulties	
**adultct**	strain		text	Time-Limited: Adulthood - Total Count	

**adultth**	strain		text	Time-Limited: Adulthood - Total Severity	
**rectotct**	strain		text	Time-Limited: Recent - Total Count of Stressors in the Past 12 Months	
**rectotth**	strain		text	Time-Limited: Recent - Total Severity of Stressors in the Past 12 Months	
**dhevntct**	strain		text	Domain: Housing - Count of Acute Life Events	
**dhdiffct**	strain		text	Domain: Housing - Count of Chronic Difficulties	
**dhallct**	strain		text	Domain: Housing - Total Count	
**dhevntth**	strain		text	Domain: Housing - Severity of Acute Life Events	
**dhdiffth**	strain		text	Domain: Housing - Severity of Chronic Difficulties	
**dhallth**	strain		text	Domain: Housing - Total Severity	
**deevntct**	strain		text	Domain: Education - Count of Acute Life Events	

**dediffct**	strain		text	Domain: Education - Count of Chronic Difficulties	
**deallct**	strain		text	Domain: Education - Total Count	
**deevntth**	strain		text	Domain: Education - Severity of Acute Life Events	
**dediffth**	strain		text	Domain: Education - Severity of Chronic Difficulties	
**deallth**	strain		text	Domain: Education - Total Severity	
**dwevntct**	strain		text	Domain: Work - Count of Acute Life Events	
**dwdiffct**	strain		text	Domain: Work - Count of Chronic Difficulties	
**dwallct**	strain		text	Domain: Work - Total Count	
**dwevntth**	strain		text	Domain: Work - Severity of Acute Life Events	
**dwdiffth**	strain		text	Domain: Work - Severity of Chronic Difficulties	
**dwallth**	strain		text	Domain: Work - Total Severity	

**dtevntct**	strain		text	Domain: Treatment/Health - Count of Acute Life Events	
**dtdiffct**	strain		text	Domain: Treatment/Health - Count of Chronic Difficulties	
**dtallct**	strain		text	Domain: Treatment/Health - Total Count	
**dtevntth**	strain		text	Domain: Treatment/Health - Severity of Acute Life Events	
**dtdiffth**	strain		text	Domain: Treatment/Health - Severity of Chronic Difficulties	
**dtallth**	strain		text	Domain: Treatment/Health - Total Severity	
**dmevntct**	strain		text	Domain: Marital/Partner - Count of Acute Life Events	
**dmdiffct**	strain		text	Domain: Marital/Partner - Count of Chronic Difficulties	
**dmallct**	strain		text	Domain: Marital/Partner - Total Count	

**dmevntth**	strain		text	Domain: Marital/Partner - Severity of Acute Life Events	
**dmdiffth**	strain		text	Domain: Marital/Partner - Severity of Chronic Difficulties	
**dmallth**	strain		text	Domain: Marital/Partner - Total Severity	
**drevntct**	strain		text	Domain: Reproduction - Count of Acute Life Events	
**drdiffct**	strain		text	Domain: Reproduction - Count of Chronic Difficulties	
**drallct**	strain		text	Domain: Reproduction - Total Count	
**drevntth**	strain		text	Domain: Reproduction - Severity of Acute Life Events	
**drdiffth**	strain		text	Domain: Reproduction - Severity of Chronic Difficulties	
**drallth**	strain		text	Domain: Reproduction - Total Severity	
**dfevntct**	strain		text	Domain: Financial - Count of Acute Life Events	

**dfdiffct**	strain		text	Domain: Financial - Count of Chronic Difficulties	
**dfallct**	strain		text	Domain: Financial - Total Count	
**dfevntth**	strain		text	Domain: Financial - Severity of Acute Life Events	
**dfdiffth**	strain		text	Domain: Financial - Severity of Chronic Difficulties	
**dfallth**	strain		text	Domain: Financial - Total Severity	
**dlevntct**	strain		text	Domain: Legal/Crime - Count of Acute Life Events	
**dldiffct**	strain		text	Domain: Legal/Crime - Count of Chronic Difficulties	
**dlallct**	strain		text	Domain: Legal/Crime - Total Count	
**dlevntth**	strain		text	Domain: Legal/Crime - Severity of Acute Life Events	
**dldiffth**	strain		text	Domain: Legal/Crime - Severity of Chronic Difficulties	

**dlallth**	strain		text	Domain: Legal/Crime - Total Severity	
**doevntct**	strain		text	Domain: Other Relationships - Count of Acute Life Events	
**dodiffct**	strain		text	Domain: Other Relationships - Count of Chronic Difficulties	
**doallct**	strain		text	Domain: Other Relationships - Total Count	
**doevntth**	strain		text	Domain: Other Relationships - Severity of Acute Life Events	
**dodiffth**	strain		text	Domain: Other Relationships - Severity of Chronic Difficulties	
**doallth**	strain		text	Domain: Other Relationships - Total Severity	
**ddevntct**	strain		text	Domain: Death - Count of Acute Life Events	
**dddiffct**	strain		text	Domain: Death - Count of Chronic Difficulties	
**ddallct**	strain		text	Domain: Death - Total Count	

**ddevntth**	strain		text	Domain: Death - Severity of Acute Life Events	
**dddiffth**	strain		text	Domain: Death - Severity of Chronic Difficulties	
**ddallth**	strain		text	Domain: Death - Total Severity	
**dxevntct**	strain		text	Domain: Life-Threatening Situations - Count of Acute Life Events	
**dxdiffct**	strain		text	Domain: Life-Threatening Situations - Count of Chronic Difficulties	
**dxallct**	strain		text	Domain: Life-Threatening Situations - Total Count	
**dxevntth**	strain		text	Domain: Life-Threatening Situations - Severity of Acute Life Events	
**dxdiffth**	strain		text	Domain: Life-Threatening Situations - Severity of Chronic Difficulties	
**dxallth**	strain		text	Domain: Life-Threatening Situations - Total Severity	

**dpevntct**	strain		text	Domain: Possessions - Count of Acute Life Events	
**dpdiffct**	strain		text	Domain: Possessions - Count of Chronic Difficulties	
**dpallct**	strain		text	Domain: Possessions - Total Count	
**dpevntth**	strain		text	Domain: Possessions - Severity of Acute Life Events	
**dpdiffth**	strain		text	Domain: Possessions - Severity of Chronic Difficulties	
**dpallth**	strain		text	Domain: Possessions - Total Severity	
**cievntct**	strain		text	Characteristic: Interpersonal Loss - Count of Acute Life Events	
**cidiffct**	strain		text	Characteristic: Interpersonal Loss - Count of Chronic Difficulties	
**ciallct**	strain		text	Characteristic: Interpersonal Loss - Total Count	
**cievntth**	strain		text	Characteristic: Interpersonal Loss -	

				Severity of Acute Life Events	
**cidiffth**	strain		text	Characteristic: Interpersonal Loss - Severity of Chronic Difficulties	
**ciallth**	strain		text	Characteristic: Interpersonal Loss - Total Severity	
**cdevntct**	strain		text	Characteristic: Physical Danger - Count of Acute Life Events	
**cddiffct**	strain		text	Characteristic: Physical Danger - Count of Chronic Difficulties	
**cdallct**	strain		text	Characteristic: Physical Danger - Total Count	
**cdevntth**	strain		text	Characteristic: Physical Danger - Severity of Acute Life Events	
**cddiffth**	strain		text	Characteristic: Physical Danger - Severity of Chronic Difficulties	
**cdallth**	strain		text	Characteristic: Physical Danger - Total Severity	
**chevntct**	strain		text	Characteristic: Humiliation - Count of Acute Life Events	

**chdiffct**	strain		text	Characteristic: Humiliation - Count of Chronic Difficulties	
**challct**	strain		text	Characteristic: Humiliation - Total Count	
**chevntth**	strain		text	Characteristic: Humiliation - Severity of Acute Life Events	
**chdiffth**	strain		text	Characteristic: Humiliation - Severity of Chronic Difficulties	
**challth**	strain		text	Characteristic: Humiliation - Total Severity	
**ceevntct**	strain		text	Characteristic: Entrapment - Count of Acute Life Events	
**cediffct**	strain		text	Characteristic: Entrapment - Count of Chronic Difficulties	
**ceallct**	strain		text	Characteristic: Entrapment - Total Count	
**ceevntth**	strain		text	Characteristic: Entrapment - Severity of Acute Life Events	
**cediffth**	strain		text	Characteristic: Entrapment - Severity of Chronic Difficulties	

**ceallth**	strain		text	Characteristic: Entrapment - Total Severity	
**crevntct**	strain		text	Characteristic: Role Change/Reversal - Count of Acute Life Events	
**crdiffct**	strain		text	Characteristic: Role Change/Reversal - Count of Chronic Difficulties	
**crallct**	strain		text	Characteristic: Role Change/Reversal - Total Count	
**crevntth**	strain		text	Characteristic: Role Change/Reversal - Severity of Acute Life Events	
**crdiffth**	strain		text	Characteristic: Role Change/Reversal - Severity of Chronic Difficulties	
**crallth**	strain		text	Characteristic: Role Change/Reversal - Total Severity	

2.3 Day 2

2.3.1 Part 1 of Questionnaire Package

Redcap Form Name: Day 2 Part 1

Questionnaire Name	Title	Variable Name

2.3.1.1 Personal Well-Being	Ryff's Psychological Well-Being Scale (PWBS)	pwbs
2.3.1.2 Couples Satisfaction	Couples Satisfaction Index (CSI-16)	csi
2.3.1.3 Sense of Coherence	Antonovsky's Sense of Coherence (SOC)	soc
2.3.1.4 Personality	NEO Five factor Model of Personality Short Form (NEO-SF)	neo
2.3.1.5 Gender Role	Bem Sex Role Inventory Long Form (BSRI-L)	bsri
2.3.1.6 Memory Complaints	Memory Complaint Checklist (MCC)	mcc
2.3.1.7 Perceived Social Support	Multidimensional Scale of Perceived Social Support (MPSS)	mpss

Variable / Field Name	Form Name	Section Header	Field Type	Field Label	Choices, Calculations, OR Slider Labels
**day2_cv**	day_2_part_1		descriptive	Thank you for your participation in the MiSBIE study!<br><br>The following questions are a validated set of questionnaires that will help us understand you better as a person. It is very important that you answer each question as well as you can.<br>This package should take about 120 min to complete.<br>We want to take this opportunity to remind you that all answers will remain strictly confidential.	
**pwbs_1**	day_2_part_1	Well-Being<br>Please indicate your degree of agreement (using a score ranging from 1-6) to the following sentences.	radio	I am not afraid to voice my opinions, even when they are in opposition to the opinions of most people.	1, 1: Strongly Disagree | 2, 2 | 3, 3 | 4, 4 | 5, 5 | 6, 6: Strongly agree
**pwbs_2**	day_2_part_1		radio	In general, I feel I am in charge of the situation in which I live.	1, 1: Strongly Disagree | 2, 2 | 3, 3 | 4, 4 | 5, 5 | 6, 6: Strongly agree

**pwbs_3**	day_2_part_1		radio	I am not interested in activities that will expand my horizons.	1, 1: Strongly Disagree | 2, 2 | 3, 3 | 4, 4 | 5, 5 | 6, 6: Strongly agree
**pwbs_4**	day_2_part_1		radio	Most people see me as loving and affectionate.	1, 1: Strongly Disagree | 2, 2 | 3, 3 | 4, 4 | 5, 5 | 6, 6: Strongly agree
**pwbs_5**	day_2_part_1		radio	I live life one day at a time and don't really think about the future	1, 1: Strongly Disagree | 2, 2 | 3, 3 | 4, 4 | 5, 5 | 6, 6: Strongly agree
**pwbs_6**	day_2_part_1		radio	When I look at the story of my life, I am pleased with how things have turned out.	1, 1: Strongly Disagree | 2, 2 | 3, 3 | 4, 4 | 5, 5 | 6, 6: Strongly agree
**pwbs_7**	day_2_part_1		radio	My decisions are not usually influenced by what everyone else is doing	1, 1: Strongly Disagree | 2, 2 | 3, 3 | 4, 4 | 5, 5 | 6, 6: Strongly agree
**pwbs_8**	day_2_part_1		radio	The demands of everyday life often get me down.	1, 1: Strongly Disagree | 2, 2 | 3, 3 | 4, 4 | 5, 5 | 6, 6: Strongly agree
**pwbs_9**	day_2_part_1		radio	I think it is important to have new experiences that challenge how you think about yourself and the world.	1, 1: Strongly Disagree | 2, 2 | 3, 3 | 4, 4 | 5, 5 | 6, 6: Strongly agree
**pwbs_10**	day_2_part_1		radio	Maintaining close relationships has been difficult and frustrating for me.	1, 1: Strongly Disagree | 2, 2 | 3, 3 | 4, 4 | 5, 5 | 6, 6: Strongly agree
**pwbs_11**	day_2_part_1		radio	I have a sense of direction and purpose in life.	1, 1: Strongly Disagree | 2, 2 | 3, 3 | 4, 4 | 5, 5 | 6, 6: Strongly agree
**pwbs_12**	day_2_part_1		radio	In general, I feel confident and positive about myself.	1, 1: Strongly Disagree | 2, 2 | 3, 3 | 4, 4 | 5, 5 | 6, 6: Strongly agree
**pwbs_13**	day_2_part_1		radio	I tend to worry about what other people think of me.	1, 1: Strongly Disagree | 2, 2 | 3, 3 | 4, 4 | 5, 5 | 6, 6: Strongly agree

**pwbs_14**	day_2_part_1		radio	I do not fit in very well with the people and the community around me.	1, 1: Strongly Disagree | 2, 2 | 3, 3 | 4, 4 | 5, 5 | 6, 6: Strongly agree
**pwbs_15**	day_2_part_1		radio	When I think about it, I haven't really improved much as a person over the years.	1, 1: Strongly Disagree | 2, 2 | 3, 3 | 4, 4 | 5, 5 | 6, 6: Strongly agree
**pwbs_16**	day_2_part_1		radio	I often feel lonely because I have few close friends with whom to share my concerns.	1, 1: Strongly Disagree | 2, 2 | 3, 3 | 4, 4 | 5, 5 | 6, 6: Strongly agree
**pwbs_17**	day_2_part_1		radio	My daily activities often seem trivial and unimportant to me.	1, 1: Strongly Disagree | 2, 2 | 3, 3 | 4, 4 | 5, 5 | 6, 6: Strongly agree
**pwbs_18**	day_2_part_1		radio	I feel like many of the people I know have gotten more out of life than I have.	1, 1: Strongly Disagree | 2, 2 | 3, 3 | 4, 4 | 5, 5 | 6, 6: Strongly agree
**pwbs_19**	day_2_part_1		radio	I tend to be influenced by people with strong opinions.	1, 1: Strongly Disagree | 2, 2 | 3, 3 | 4, 4 | 5, 5 | 6, 6: Strongly agree
**pwbs_20**	day_2_part_1		radio	I am quite good at managing the many responsibilities of my daily life	1, 1: Strongly Disagree | 2, 2 | 3, 3 | 4, 4 | 5, 5 | 6, 6: Strongly agree
**pwbs_21**	day_2_part_1		radio	I have the sense that I have developed a lot as a person over time.	1, 1: Strongly Disagree | 2, 2 | 3, 3 | 4, 4 | 5, 5 | 6, 6: Strongly agree
**pwbs_22**	day_2_part_1		radio	I enjoy personal and mutual conversations with family members or friends.	1, 1: Strongly Disagree | 2, 2 | 3, 3 | 4, 4 | 5, 5 | 6, 6: Strongly agree
**pwbs_23**	day_2_part_1		radio	I don't have a good sense of what it is I'm trying to accomplish in life.	1, 1: Strongly Disagree | 2, 2 | 3, 3 | 4, 4 | 5, 5 | 6, 6: Strongly agree
**pwbs_24**	day_2_part_1		radio	I like most aspects of my personality.	1, 1: Strongly Disagree | 2, 2 | 3, 3 | 4, 4 | 5, 5 | 6, 6: Strongly agree

**pwbs_25**	day_2_part_1		radio	I have confidence in my opinions, even if they are contrary to the general consensus.	1, 1: Strongly Disagree | 2, 2 | 3, 3 | 4, 4 | 5, 5 | 6, 6: Strongly agree
**pwbs_26**	day_2_part_1		radio	I often feel overwhelmed by my responsibilities.	1, 1: Strongly Disagree | 2, 2 | 3, 3 | 4, 4 | 5, 5 | 6, 6: Strongly agree
**pwbs_27**	day_2_part_1		radio	I do not enjoy being in new situations that require me to change my old familiar ways of doing things.	1, 1: Strongly Disagree | 2, 2 | 3, 3 | 4, 4 | 5, 5 | 6, 6: Strongly agree
**pwbs_28**	day_2_part_1		radio	People would describe me as a giving person, willing to share my time with others.	1, 1: Strongly Disagree | 2, 2 | 3, 3 | 4, 4 | 5, 5 | 6, 6: Strongly agree
**pwbs_29**	day_2_part_1		radio	I enjoy making plans for the future and working to make them a reality.	1, 1: Strongly Disagree | 2, 2 | 3, 3 | 4, 4 | 5, 5 | 6, 6: Strongly agree
**pwbs_30**	day_2_part_1		radio	In many ways, I feel disappointed about my achievements in life.	1, 1: Strongly Disagree | 2, 2 | 3, 3 | 4, 4 | 5, 5 | 6, 6: Strongly agree
**pwbs_31**	day_2_part_1		radio	It's difficult for me to voice my own opinions on controversial matters.	1, 1: Strongly Disagree | 2, 2 | 3, 3 | 4, 4 | 5, 5 | 6, 6: Strongly agree
**pwbs_32**	day_2_part_1		radio	I have difficulty arranging my life in a way that is satisfying to me.	1, 1: Strongly Disagree | 2, 2 | 3, 3 | 4, 4 | 5, 5 | 6, 6: Strongly agree
**pwbs_33**	day_2_part_1		radio	For me, life has been a continuous process of learning, changing, and growth.	1, 1: Strongly Disagree | 2, 2 | 3, 3 | 4, 4 | 5, 5 | 6, 6: Strongly agree
**pwbs_34**	day_2_part_1		radio	I have not experienced many warm and trusting relationships with others.	1, 1: Strongly Disagree | 2, 2 | 3, 3 | 4, 4 | 5, 5 | 6, 6: Strongly agree
**pwbs_35**	day_2_part_1		radio	Some people wander aimlessly through life, but I am not one of them.	1, 1: Strongly Disagree | 2, 2 | 3, 3 | 4, 4 | 5, 5 | 6, 6: Strongly agree

**pwbs_36**	day_2_part_1		radio	My attitude about myself is probably not as positive as most people feel about themselves.	1, 1: Strongly Disagree | 2, 2 | 3, 3 | 4, 4 | 5, 5 | 6, 6: Strongly agree
**pwbs_37**	day_2_part_1		radio	I judge myself by what I think is important, not by the values of what others think is important.	1, 1: Strongly Disagree | 2, 2 | 3, 3 | 4, 4 | 5, 5 | 6, 6: Strongly agree
**pwbs_38**	day_2_part_1		radio	I have been able to build a home and a lifestyle for myself that is much to my liking.	1, 1: Strongly Disagree | 2, 2 | 3, 3 | 4, 4 | 5, 5 | 6, 6: Strongly agree
**pwbs_39**	day_2_part_1		radio	I gave up trying to make big improvements or changes in my life a long time ago.	1, 1: Strongly Disagree | 2, 2 | 3, 3 | 4, 4 | 5, 5 | 6, 6: Strongly agree
**pwbs_40**	day_2_part_1		radio	I know that I can trust my friends, and they know they can trust me.	1, 1: Strongly Disagree | 2, 2 | 3, 3 | 4, 4 | 5, 5 | 6, 6: Strongly agree
**pwbs_41**	day_2_part_1		radio	I sometimes feel as if I've done all there is to do in life.	1, 1: Strongly Disagree | 2, 2 | 3, 3 | 4, 4 | 5, 5 | 6, 6: Strongly agree
**pwbs_42**	day_2_part_1		radio	When I compare myself to friends and acquaintances, it makes me feel good about who I am.	1, 1: Strongly Disagree | 2, 2 | 3, 3 | 4, 4 | 5, 5 | 6, 6: Strongly agree
**csi_0**	day_2_part_1	Romantic relationship	yes/no	Are you currently involved in a romantic relationship?	
**csi_1**	day_2_part_1		radio	Please indicate the degree of happiness, all things considered, of your relationship.	0, Extremely unhappy | 1,Fairly unhappy | 2,A little unhappy| 3,Happy | 4,Very happy | 5, Extremely happy | 6,Perfect
**csi_2**	day_2_part_1		radio	In general, how often do you think that things between you and your partner are going well?	5, All the time | 4, Most of the time | 3, More often than nor | 2, Occasionally | 1, Rarely | 0, Never

**csi_3**	day_2_part_1		radio	Our relationship is strong	0, Not at all true | 1, A little true | 2, Somewhat true | 3, Mostly true | 4, Almost completely true | 5, Completely true
**csi_4**	day_2_part_1	Please choose one of the following answers for each question:<br>0, Not at all true | 1, A little true | 2, Somewhat true | 3, Mostly true | 4, Almost completely true | 5, Completely true	radio	My relationship with my partner makes me happy	0, Not at all true | 1, A little true | 2, Somewhat true | 3, Mostly true | 4, Almost completely true | 5, Completely true
**csi_5**	day_2_part_1		radio	I have a warm and comfortable relationship with my partner	0, Not at all true | 1, A little true | 2, Somewhat true | 3, Mostly true | 4, Almost completely true | 5, Completely true
**csi_6**	day_2_part_1		radio	I really feel like part of a team with my partner	0, Not at all true | 1, A little true | 2, Somewhat true | 3, Mostly true | 4, Almost completely true | 5, Completely true
**csi_7**	day_2_part_1	Please choose one of the following answers for each question:<br>0, Not at all | 1, A little | 2, Somewhat | 3, Mostly | 4, Almost completely | 5, Completely	radio	How rewarding is your relationship with your partner?	0, Not at all | 1, A little | 2, Somewhat | 3, Mostly | 4, Almost completely | 5, Completely
**csi_8**	day_2_part_1		radio	How well does your partner meet your needs?	0, Not at all | 1, A little | 2, Somewhat | 3, Mostly | 4, Almost completely | 5, Completely
**csi_9**	day_2_part_1		radio	To what extent has your relationship met your original expectations?	0, Not at all | 1, A little | 2, Somewhat | 3, Mostly | 4, Almost completely | 5, Completely

**csi_10**	day_2_part_1		radio	In general, how satisfied are you with your relationship?	0, Not at all | 1, A little | 2, Somewhat | 3, Mostly | 4, Almost completely | 5, Completely
**csi_11**	day_2_part_1	For each of the following items, select the answer that best describes how you feel about your relationship. Base your responses on your first impressions and immediate feelings about the item.	radio		5,5. Interesting | 4, 4 | 3, 3 | 2,2 | 1, 1 |0, 0. Boring
**csi_12**	day_2_part_1		radio		5,5. Bad | 4, 4 | 3, 3 | 2,2 | 1, 1 |0, 0. Good
**csi_13**	day_2_part_1		radio		5,5. Full | 4, 4 | 3, 3 | 2,2 | 1, 1 |0, 0. Empty
**csi_14**	day_2_part_1		radio		5,5. Sturdy | 4, 4 | 3, 3 | 2,2 | 1, 1 |0, 0. Fragile
**csi_15**	day_2_part_1		radio		5,5. Discouraging| 4, 4 | 3, 3 | 2,2 | 1, 1 |0, 0. Hopeful
**csi_16**	day_2_part_1		radio		5,5. Enjoyable | 4, 4 | 3, 3 | 2,2 | 1, 1 |0, 0. Miserable
**soc_1**	day_2_part_1	Life Reflection	radio	When you talk to people, do you have the feeling that they don't understand you?	1, 1: Never | 2, 2 | 3, 3 | 4, 4 | 5, 5 | 6, 6 | 7, 7: Always have this feeling
**soc_2**	day_2_part_1		radio	In the past, when you had to do something which depended upon cooperation with others, did you have the feeling that it:	1, 1: Surely wouldn't get done | 2, 2 | 3, 3 | 4, 4 | 5, 5 | 6, 6 | 7, 7: Surely would get done
**soc_3**	day_2_part_1		radio	Think of the people with whom you come into contact daily, aside from	1, 1: You feel that they are strangers | 2, 2 | 3, 3 | 4, 4 | 5, 5 | 6, 6 | 7, 7: You know them very well

				the ones to whom you feel closest. How well do you know most of them?	
**soc_4**	day_2_part_1		radio	Do you have the feeling that you don't really care about what goes on around you?	1, 1: Very seldom or never | 2, 2 | 3, 3 | 4, 4 | 5, 5 | 6, 6 | 7, 7: Very often
**soc_5**	day_2_part_1		radio	Has it happened in the past that you were surprised by the behavior of people whom you thought you knew well?	1, 1: Never happened | 2, 2 | 3, 3 | 4, 4 | 5, 5 | 6, 6 | 7, 7: Always happened
**soc_6**	day_2_part_1		radio	Has it happened that people whom you counted on disappointed you?	1, 1: Never happened | 2, 2 | 3, 3 | 4, 4 | 5, 5 | 6, 6 | 7, 7: Always happened
**soc_7**	day_2_part_1		radio	Life is:	1, 1: Full of interest | 2, 2 | 3, 3 | 4, 4 | 5, 5 | 6, 6 | 7, 7: Completely routine
**soc_8**	day_2_part_1		radio	Until now, your life has had:	1, 1: No clear goals or purpose at all | 2, 2 | 3, 3 | 4, 4 | 5, 5 | 6, 6 | 7, 7: Very clear goals and purpose
**soc_9**	day_2_part_1		radio	Do you have the feeling that you're being treated unfairly?	1, 1: Very often | 2, 2 | 3, 3 | 4, 4 | 5, 5 | 6, 6 | 7, 7: Very seldom or never
**soc_10**	day_2_part_1		radio	In the past ten years, your life has been:	1, 1: Full of changes without your knowing what will happen next | 2, 2 | 3, 3 | 4, 4 | 5, 5 | 6, 6 | 7, 7: Completely consistent and clear
**soc_11**	day_2_part_1		radio	Most of the things you do in the future will probably be:	1, 1: Completely fascinating | 2, 2 | 3, 3 | 4, 4 | 5, 5 | 6, 6 | 7, 7: Deadly boring
**soc_12**	day_2_part_1		radio	Do you have the feeling that you are in an unfamiliar situation and don't know what to do?	1, 1: Very often | 2, 2 | 3, 3 | 4, 4 | 5, 5 | 6, 6 | 7, 7: Very seldom or never

**soc_13**	day_2_part_1		radio	What best describes how you see life?	1, 1: One can always find a solution to painful things in life | 2, 2 | 3, 3 | 4, 4 | 5, 5 | 6, 6 | 7, 7: There is no solution to painful things in life
**soc_14**	day_2_part_1		radio	When you think about your life, you very often:	1, 1: Feel how good it is to be alive | 2, 2 | 3, 3 | 4, 4 | 5, 5 | 6, 6 | 7, 7: Ask yourself why you exist at all
**soc_15**	day_2_part_1		radio	When you face a difficult problem, the choice of a solution is:	1, 1: Always confusing and hard to find | 2, 2 | 3, 3 | 4, 4 | 5, 5 | 6, 6 | 7, 7: Always completely clear
**soc_16**	day_2_part_1		radio	Doing the things you do every day is:	1, 1: A source of deep pleasure and satisfaction | 2, 2 | 3, 3 | 4, 4 | 5, 5 | 6, 6 | 7, 7: A source of pain and boredom
**soc_17**	day_2_part_1		radio	Your life in the future will probably be:	1, 1: Full of changes without knowing what will happen next | 2, 2 | 3, 3 | 4, 4 | 5, 5 | 6, 6 | 7, 7: Completely consistent and clear
**soc_18**	day_2_part_1		radio	When something unpleasant happened in the past your tendency was:	1, 1: To "eat yourself up" about it | 2, 2 | 3, 3 | 4, 4 | 5, 5 | 6, 6 | 7, 7: To say, "OK, that's that, I have to live with it," and go on.
**soc_19**	day_2_part_1		radio	Do you have very mixed-up feelings and ideas?	1, 1: Very often | 2, 2 | 3, 3 | 4, 4 | 5, 5 | 6, 6 | 7, 7: Very seldom or never
**soc_20**	day_2_part_1		radio	When you do something that gives you a good feeling:	1, 1: It's certain that you'll go on feeling good | 2, 2 | 3, 3 | 4, 4 | 5, 5 | 6, 6 | 7, 7: It's certain that something will happen to spoil the feeling
**soc_21**	day_2_part_1		radio	Does it happen that you have feelings inside you would rather not feel?	1, 1: Very often | 2, 2 | 3, 3 | 4, 4 | 5, 5 | 6, 6 | 7, 7: Very seldom or never

**soc_22**	day_2_part_1		radio	You anticipate that your personal life in the future will be:	1, 1: Totally without meaning or purpose | 2, 2 | 3, 3 | 4, 4 | 5, 5 | 6, 6 | 7, 7: Full of meaning and purpose
**soc_23**	day_2_part_1		radio	Do you think that there will always be people whom you'll be able to count on in the future?	1, 1: You're certain there will be | 2, 2 | 3, 3 | 4, 4 | 5, 5 | 6, 6 | 7, 7: You doubt there will be
**soc_24**	day_2_part_1		radio	Does it happen that you have the feeling that you don't know exactly what's about to happen?	1, 1: Very often | 2, 2 | 3, 3 | 4, 4 | 5, 5 | 6, 6 | 7, 7: Very seldom or never
**soc_25**	day_2_part_1		radio	Many people, even those with a strong character, sometimes feel like sad sacks (losers) in certain situations. How often have you felt this way in the past?	1, 1: Never | 2, 2 | 3, 3 | 4, 4 | 5, 5 | 6, 6 | 7, 7: Very often
**soc_26**	day_2_part_1		radio	When something happened, have you generally found that:	1, 1: You overestimated or underestimated its importance | 2, 2 | 3, 3 | 4, 4 | 5, 5 | 6, 6 | 7, 7: You saw things in the right proportion
**soc_27**	day_2_part_1		radio	When you think of the difficulties you are likely to face in important aspects of your life, do you have the feeling that:	1, 1: You will always succeed in overcoming the difficulties | 2, 2 | 3, 3 | 4, 4 | 5, 5 | 6, 6 | 7, 7: You won't succeed in overcoming the difficulties
**soc_28**	day_2_part_1		radio	How often do you have the feeling that there's little meaning in the things you do in your daily life?	1, 1: Very often | 2, 2 | 3, 3 | 4, 4 | 5, 5 | 6, 6 | 7, 7: Very seldom or never
**soc_29**	day_2_part_1		radio	How often do you have feelings that you're not sure you can keep under control?	1, 1: Very often | 2, 2 | 3, 3 | 4, 4 | 5, 5 | 6, 6 | 7, 7: Very seldom or never
**neo_sf_1**	day_2_part_1	Personality<br>Instructions: Read each statement carefully. For each statement, fill in	radio	I am not a worrier.	1, Strongly Disagree | 2, Disagree | 3, Neutral | 4, Agree | 5, Strongly Agree

		the box with the response that best represents your opinion.			
**neo_sf_2**	day_2_part_1		radio	I like to have a lot of people around me.	1, Strongly Disagree | 2, Disagree | 3, Neutral | 4, Agree | 5, Strongly Agree
**neo_sf_3**	day_2_part_1		radio	I don't like to waste my time daydreaming.	1, Strongly Disagree | 2, Disagree | 3, Neutral | 4, Agree | 5, Strongly Agree
**neo_sf_4**	day_2_part_1		radio	I try to be courteous to everyone I meet.	1, Strongly Disagree | 2, Disagree | 3, Neutral | 4, Agree | 5, Strongly Agree
**neo_sf_5**	day_2_part_1		radio	I keep my belongings neat and clean.	1, Strongly Disagree | 2, Disagree | 3, Neutral | 4, Agree | 5, Strongly Agree
**neo_sf_6**	day_2_part_1		radio	I often feel inferior to others.	1, Strongly Disagree | 2, Disagree | 3, Neutral | 4, Agree | 5, Strongly Agree
**neo_sf_7**	day_2_part_1		radio	I laugh easily.	1, Strongly Disagree | 2, Disagree | 3, Neutral | 4, Agree | 5, Strongly Agree
**neo_sf_8**	day_2_part_1		radio	Once I find the right way to do something, I stick to it.	1, Strongly Disagree | 2, Disagree | 3, Neutral | 4, Agree | 5, Strongly Agree
**neo_sf_9**	day_2_part_1		radio	I often get into arguments with my family and co-workers.	1, Strongly Disagree | 2, Disagree | 3, Neutral | 4, Agree | 5, Strongly Agree
**neo_sf_10**	day_2_part_1		radio	I'm pretty good about pacing myself so as to get things done on time.	1, Strongly Disagree | 2, Disagree | 3, Neutral | 4, Agree | 5, Strongly Agree
**neo_sf_11**	day_2_part_1		radio	When I'm under a great deal of stress, sometimes I feel like I'm going to pieces.	1, Strongly Disagree | 2, Disagree | 3, Neutral | 4, Agree | 5, Strongly Agree
**neo_sf_12**	day_2_part_1		radio	I don't consider myself especially "light-hearted."	1, Strongly Disagree | 2, Disagree | 3, Neutral | 4, Agree | 5, Strongly Agree

**neo_sf_13**	day_2_part_1		radio	I am intrigued by the patterns I find in art and nature.	1, Strongly Disagree | 2, Disagree | 3, Neutral | 4, Agree | 5, Strongly Agree
**neo_sf_14**	day_2_part_1		radio	Some people think I'm selfish and egotistical.	1, Strongly Disagree | 2, Disagree | 3, Neutral | 4, Agree | 5, Strongly Agree
**neo_sf_15**	day_2_part_1		radio	I am not a very methodical person.	1, Strongly Disagree | 2, Disagree | 3, Neutral | 4, Agree | 5, Strongly Agree
**neo_sf_16**	day_2_part_1		radio	I rarely feel lonely or blue.	1, Strongly Disagree | 2, Disagree | 3, Neutral | 4, Agree | 5, Strongly Agree
**neo_sf_17**	day_2_part_1		radio	I really enjoy talking to people.	1, Strongly Disagree | 2, Disagree | 3, Neutral | 4, Agree | 5, Strongly Agree
**neo_sf_18**	day_2_part_1		radio	I believe letting students hear controversial speakers can only confuse or mislead them.	1, Strongly Disagree | 2, Disagree | 3, Neutral | 4, Agree | 5, Strongly Agree
**neo_sf_19**	day_2_part_1		radio	I would rather cooperate with others than compete with them.	1, Strongly Disagree | 2, Disagree | 3, Neutral | 4, Agree | 5, Strongly Agree
**neo_sf_20**	day_2_part_1		radio	I try to perform all the tasks assigned to me conscientiously.	1, Strongly Disagree | 2, Disagree | 3, Neutral | 4, Agree | 5, Strongly Agree
**neo_sf_21**	day_2_part_1		radio	I often feel tense and jittery.	1, Strongly Disagree | 2, Disagree | 3, Neutral | 4, Agree | 5, Strongly Agree
**neo_sf_22**	day_2_part_1		radio	I like to be where the action is.	1, Strongly Disagree | 2, Disagree | 3, Neutral | 4, Agree | 5, Strongly Agree
**neo_sf_23**	day_2_part_1		radio	Poetry has little or no effect on me.	1, Strongly Disagree | 2, Disagree | 3, Neutral | 4, Agree | 5, Strongly Agree
**neo_sf_24**	day_2_part_1		radio	I tend to be cynical and skeptical of other's intentions.	1, Strongly Disagree | 2, Disagree | 3, Neutral | 4, Agree | 5, Strongly Agree

**neo_sf_25**	day_2_part_1		radio	I have a clear set of goals and work toward them in an orderly fashion.	1, Strongly Disagree | 2, Disagree | 3, Neutral | 4, Agree | 5, Strongly Agree
**neo_sf_26**	day_2_part_1		radio	Sometime I feel completely worthless.	1, Strongly Disagree | 2, Disagree | 3, Neutral | 4, Agree | 5, Strongly Agree
**neo_sf_27**	day_2_part_1		radio	I usually prefer to do things alone.	1, Strongly Disagree | 2, Disagree | 3, Neutral | 4, Agree | 5, Strongly Agree
**neo_sf_28**	day_2_part_1		radio	I often try new things and foreign foods.	1, Strongly Disagree | 2, Disagree | 3, Neutral | 4, Agree | 5, Strongly Agree
**neo_sf_29**	day_2_part_1		radio	I believe that most people will take advantage of you if you let them.	1, Strongly Disagree | 2, Disagree | 3, Neutral | 4, Agree | 5, Strongly Agree
**neo_sf_30**	day_2_part_1		radio	I waste a lot of time before settling down to work.	1, Strongly Disagree | 2, Disagree | 3, Neutral | 4, Agree | 5, Strongly Agree
**neo_sf_31**	day_2_part_1		radio	I rarely feel fearful or anxious.	1, Strongly Disagree | 2, Disagree | 3, Neutral | 4, Agree | 5, Strongly Agree
**neo_sf_32**	day_2_part_1		radio	I often feel as if I'm bursting with energy.	1, Strongly Disagree | 2, Disagree | 3, Neutral | 4, Agree | 5, Strongly Agree
**neo_sf_33**	day_2_part_1		radio	I seldom notice the moods or feelings that different environments produce.	1, Strongly Disagree | 2, Disagree | 3, Neutral | 4, Agree | 5, Strongly Agree
**neo_sf_34**	day_2_part_1		radio	Most people I know like me.	1, Strongly Disagree | 2, Disagree | 3, Neutral | 4, Agree | 5, Strongly Agree
**neo_sf_35**	day_2_part_1		radio	I work hard to accomplish my goals.	1, Strongly Disagree | 2, Disagree | 3, Neutral | 4, Agree | 5, Strongly Agree
**neo_sf_36**	day_2_part_1		radio	I often get angry at the way people treat me.	1, Strongly Disagree | 2, Disagree | 3, Neutral | 4, Agree | 5, Strongly Agree

**neo_sf_37**	day_2_part_1		radio	I am a cheerful, high-spirited person.	1, Strongly Disagree | 2, Disagree | 3, Neutral | 4, Agree | 5, Strongly Agree
**neo_sf_38**	day_2_part_1		radio	I believe we should look to our religious authorities for decisions on moral issues.	1, Strongly Disagree | 2, Disagree | 3, Neutral | 4, Agree | 5, Strongly Agree
**neo_sf_39**	day_2_part_1		radio	Some people think of me as cold and calculating.	1, Strongly Disagree | 2, Disagree | 3, Neutral | 4, Agree | 5, Strongly Agree
**neo_sf_40**	day_2_part_1		radio	When I make a commitment, I can always be counted on to follow through.	1, Strongly Disagree | 2, Disagree | 3, Neutral | 4, Agree | 5, Strongly Agree
**neo_sf_41**	day_2_part_1		radio	Too often, when things go wrong, I get discouraged and feel like giving up.	1, Strongly Disagree | 2, Disagree | 3, Neutral | 4, Agree | 5, Strongly Agree
**neo_sf_42**	day_2_part_1		radio	I am not a cheerful optimist.	1, Strongly Disagree | 2, Disagree | 3, Neutral | 4, Agree | 5, Strongly Agree
**neo_sf_43**	day_2_part_1		radio	Sometimes when I am reading poetry or looking at work of art, I feel a chill or wave of excitement.	1, Strongly Disagree | 2, Disagree | 3, Neutral | 4, Agree | 5, Strongly Agree
**neo_sf_44**	day_2_part_1		radio	I'm hard-headed and tough-minded in my attitudes.	1, Strongly Disagree | 2, Disagree | 3, Neutral | 4, Agree | 5, Strongly Agree
**neo_sf_45**	day_2_part_1		radio	Sometimes I'm not as dependable or reliable as I should be.	1, Strongly Disagree | 2, Disagree | 3, Neutral | 4, Agree | 5, Strongly Agree
**neo_sf_46**	day_2_part_1		radio	I am seldom sad or depressed.	1, Strongly Disagree | 2, Disagree | 3, Neutral | 4, Agree | 5, Strongly Agree
**neo_sf_47**	day_2_part_1		radio	My life is fast-paced.	1, Strongly Disagree | 2, Disagree | 3, Neutral | 4, Agree | 5, Strongly Agree

**neo_sf_48**	day_2_part_1		radio	I have little interest in speculating on the nature of the universe or the human condition.	1, Strongly Disagree | 2, Disagree | 3, Neutral | 4, Agree | 5, Strongly Agree
**neo_sf_49**	day_2_part_1		radio	I generally try to be thoughtful and considerate.	1, Strongly Disagree | 2, Disagree | 3, Neutral | 4, Agree | 5, Strongly Agree
**neo_sf_50**	day_2_part_1		radio	I am a productive person who always gets the job done.	1, Strongly Disagree | 2, Disagree | 3, Neutral | 4, Agree | 5, Strongly Agree
**neo_sf_51**	day_2_part_1		radio	I often feel helpless and want someone else to solve my problems.	1, Strongly Disagree | 2, Disagree | 3, Neutral | 4, Agree | 5, Strongly Agree
**neo_sf_52**	day_2_part_1		radio	I am a very active person.	1, Strongly Disagree | 2, Disagree | 3, Neutral | 4, Agree | 5, Strongly Agree
**neo_sf_53**	day_2_part_1		radio	I have a lot of intellectual curiosity.	1, Strongly Disagree | 2, Disagree | 3, Neutral | 4, Agree | 5, Strongly Agree
**neo_sf_54**	day_2_part_1		radio	If I don't like people, I let them know it.	1, Strongly Disagree | 2, Disagree | 3, Neutral | 4, Agree | 5, Strongly Agree
**neo_sf_55**	day_2_part_1		radio	I never seem to be able to get organized.	1, Strongly Disagree | 2, Disagree | 3, Neutral | 4, Agree | 5, Strongly Agree
**neo_sf_56**	day_2_part_1		radio	At times I have been so ashamed I just wanted to hide.	1, Strongly Disagree | 2, Disagree | 3, Neutral | 4, Agree | 5, Strongly Agree
**neo_sf_57**	day_2_part_1		radio	I would rather go my own way than be a leader of others.	1, Strongly Disagree | 2, Disagree | 3, Neutral | 4, Agree | 5, Strongly Agree
**neo_sf_58**	day_2_part_1		radio	I often enjoy playing with theories or abstract ideas.	1, Strongly Disagree | 2, Disagree | 3, Neutral | 4, Agree | 5, Strongly Agree
**neo_sf_59**	day_2_part_1		radio	If necessary, I am willing to manipulate people to get what I want.	1, Strongly Disagree | 2, Disagree | 3, Neutral | 4, Agree | 5, Strongly Agree

**neo_sf_60**	day_2_part_1		radio	I strive for excellence in everything I do.	1, Strongly Disagree | 2, Disagree | 3, Neutral | 4, Agree | 5, Strongly Agree
**bsri_1**	day_2_part_1	Attitude<br>Instructions: The following are a number of personality characteristics. We would like you to use those characteristics to describe yourself; that is, we would like you to indicate, on a scale from 1 to 7, how true of you each of these characteristics is.	radio	Defend my own beliefs	1, Never or almost never true | 2, Usually not true | 3, Sometimes but infrequently true | 4, Occasionally true | 5, Often true | 6, Usually true | 7, Always or almost always true
**bsri_2**	day_2_part_1		radio	Affectionate	1, Never or almost never true | 2, Usually not true | 3, Sometimes but infrequently true | 4, Occasionally true | 5, Often true | 6, Usually true | 7, Always or almost always true
**bsri_3**	day_2_part_1		radio	Conscientious	1, Never or almost never true | 2, Usually not true | 3, Sometimes but infrequently true | 4, Occasionally true | 5, Often true | 6, Usually true | 7, Always or almost always true
**bsri_4**	day_2_part_1		radio	Independent	1, Never or almost never true | 2, Usually not true | 3, Sometimes but infrequently true | 4, Occasionally true | 5, Often true | 6, Usually true | 7, Always or almost always true
**bsri_5**	day_2_part_1		radio	Sympathetic	1, Never or almost never true | 2, Usually not true | 3, Sometimes but infrequently true | 4, Occasionally true | 5, Often true | 6, Usually true | 7, Always or almost always true
**bsri_6**	day_2_part_1		radio	Moody	1, Never or almost never true | 2, Usually not true | 3, Sometimes but infrequently true | 4, Occasionally true | 5, Often true |

					6, Usually true | 7, Always or almost always true
**bsri_7**	day_2_part_1		radio	Assertive	1, Never or almost never true | 2, Usually not true | 3, Sometimes but infrequently true | 4, Occasionally true | 5, Often true | 6, Usually true | 7, Always or almost always true
**bsri_8**	day_2_part_1		radio	Sensitive to needs of others	1, Never or almost never true | 2, Usually not true | 3, Sometimes but infrequently true | 4, Occasionally true | 5, Often true | 6, Usually true | 7, Always or almost always true
**bsri_9**	day_2_part_1		radio	Reliable	1, Never or almost never true | 2, Usually not true | 3, Sometimes but infrequently true | 4, Occasionally true | 5, Often true | 6, Usually true | 7, Always or almost always true
**bsri_10**	day_2_part_1		radio	Strong personality	1, Never or almost never true | 2, Usually not true | 3, Sometimes but infrequently true | 4, Occasionally true | 5, Often true | 6, Usually true | 7, Always or almost always true
**bsri_11**	day_2_part_1		radio	Understanding	1, Never or almost never true | 2, Usually not true | 3, Sometimes but infrequently true | 4, Occasionally true | 5, Often true | 6, Usually true | 7, Always or almost always true
**bsri_12**	day_2_part_1		radio	Jealous	1, Never or almost never true | 2, Usually not true | 3, Sometimes but infrequently true | 4, Occasionally true | 5, Often true | 6, Usually true | 7, Always or almost always true

**bsri_13**	day_2_part_1		radio	Forceful	1, Never or almost never true	2, Usually not true	3, Sometimes but infrequently true	4, Occasionally true	5, Often true	6, Usually true	7, Always or almost always true
**bsri_14**	day_2_part_1		radio	Compassionate	1, Never or almost never true	2, Usually not true	3, Sometimes but infrequently true	4, Occasionally true	5, Often true	6, Usually true	7, Always or almost always true
**bsri_15**	day_2_part_1		radio	Truthful	1, Never or almost never true	2, Usually not true	3, Sometimes but infrequently true	4, Occasionally true	5, Often true	6, Usually true	7, Always or almost always true
**bsri_16**	day_2_part_1		radio	Have leadership abilities	1, Never or almost never true	2, Usually not true	3, Sometimes but infrequently true	4, Occasionally true	5, Often true	6, Usually true	7, Always or almost always true
**bsri_17**	day_2_part_1		radio	Eager to soothe hurt feelings	1, Never or almost never true	2, Usually not true	3, Sometimes but infrequently true	4, Occasionally true	5, Often true	6, Usually true	7, Always or almost always true
**bsri_18**	day_2_part_1		radio	Secretive	1, Never or almost never true	2, Usually not true	3, Sometimes but infrequently true	4, Occasionally true	5, Often true	6, Usually true	7, Always or almost always true
**bsri_19**	day_2_part_1		radio	Willing to take risks	1, Never or almost never true	2, Usually not true	3, Sometimes but infrequently true	4, Occasionally true	5, Often true

					6, Usually true | 7, Always or almost always true
**bsri_20**	day_2_part_1		radio	Warm	1, Never or almost never true | 2, Usually not true | 3, Sometimes but infrequently true | 4, Occasionally true | 5, Often true | 6, Usually true | 7, Always or almost always true
**bsri_21**	day_2_part_1		radio	Adaptable	1, Never or almost never true | 2, Usually not true | 3, Sometimes but infrequently true | 4, Occasionally true | 5, Often true | 6, Usually true | 7, Always or almost always true
**bsri_22**	day_2_part_1		radio	Dominant	1, Never or almost never true | 2, Usually not true | 3, Sometimes but infrequently true | 4, Occasionally true | 5, Often true | 6, Usually true | 7, Always or almost always true
**bsri_23**	day_2_part_1		radio	Tender	1, Never or almost never true | 2, Usually not true | 3, Sometimes but infrequently true | 4, Occasionally true | 5, Often true | 6, Usually true | 7, Always or almost always true
**bsri_24**	day_2_part_1		radio	Conceited	1, Never or almost never true | 2, Usually not true | 3, Sometimes but infrequently true | 4, Occasionally true | 5, Often true | 6, Usually true | 7, Always or almost always true
**bsri_25**	day_2_part_1		radio	Willing to take a stand	1, Never or almost never true | 2, Usually not true | 3, Sometimes but infrequently true | 4, Occasionally true | 5, Often true | 6, Usually true | 7, Always or almost always true

**bsri_26**	day_2_part_1		radio	Love children	1, Never or almost never true | 2, Usually not true | 3, Sometimes but infrequently true | 4, Occasionally true | 5, Often true | 6, Usually true | 7, Always or almost always true
**bsri_27**	day_2_part_1		radio	Tactful	1, Never or almost never true | 2, Usually not true | 3, Sometimes but infrequently true | 4, Occasionally true | 5, Often true | 6, Usually true | 7, Always or almost always true
**bsri_28**	day_2_part_1		radio	Aggressive	1, Never or almost never true | 2, Usually not true | 3, Sometimes but infrequently true | 4, Occasionally true | 5, Often true | 6, Usually true | 7, Always or almost always true
**bsri_29**	day_2_part_1		radio	Gentle	1, Never or almost never true | 2, Usually not true | 3, Sometimes but infrequently true | 4, Occasionally true | 5, Often true | 6, Usually true | 7, Always or almost always true
**bsri_30**	day_2_part_1		radio	Conventional	1, Never or almost never true | 2, Usually not true | 3, Sometimes but infrequently true | 4, Occasionally true | 5, Often true | 6, Usually true | 7, Always or almost always true
**bsri_31**	day_2_part_1		radio	Self-reliant	1, Never or almost never true | 2, Usually not true | 3, Sometimes but infrequently true | 4, Occasionally true | 5, Often true | 6, Usually true | 7, Always or almost always true
**bsri_32**	day_2_part_1		radio	Yielding	1, Never or almost never true | 2, Ususally not true | 3, Sometimes but infrequently true | 4, Occasionally true |

					5, often true | 6, Usually true | 7, Always or almost always true
**bsri_33**	day_2_part_1		radio	Helpful	1, Never or almost never true | 2, Usually not true | 3, Sometimes but infrequently true | 4, Occasionally true | 5, Often true | 6, Usually true | 7, Always or almost always true
**bsri_34**	day_2_part_1		radio	Athletic	1, Never or almost never true | 2, Usually not true | 3, Sometimes but infrequently true | 4, Occasionally true | 5, Often true | 6, Usually true | 7, Always or almost always true
**bsri_35**	day_2_part_1		radio	Cheerful	1, Never or almost never true | 2, Usually not true | 3, Sometimes but infrequently true | 4, Occasionally true | 5, Often true | 6, Usually true | 7, Always or almost always true
**bsri_36**	day_2_part_1		radio	Unsystematic	1, Never or almost never true | 2, Usually not true | 3, Sometimes but infrequently true | 4, Occasionally true | 5, Often true | 6, Usually true | 7, Always or almost always true
**bsri_37**	day_2_part_1		radio	Analytical	1, Never or almost never true | 2, Usually not true | 3, Sometimes but infrequently true | 4, Occasionally true | 5, Often true | 6, Usually true | 7, Always or almost always true
**bsri_38**	day_2_part_1		radio	Shy	1, Never or almost never true | 2, Usually not true | 3, Sometimes but infrequently true | 4, Occasionally true | 5, Often true | 6, Usually true | 7, Always or almost always true

**bsri_39**	day_2_part_1		radio	Inefficient	1, Never or almost never true | 2, Usually not true | 3, Sometimes but infrequently true | 4, Occasionally true | 5, Often true | 6, Usually true | 7, Always or almost always true
**bsri_40**	day_2_part_1		radio	Make decisions easily	1, Never or almost never true | 2, Usually not true | 3, Sometimes but infrequently true | 4, Occasionally true | 5, Often true | 6, Usually true | 7, Always or almost always true
**bsri_41**	day_2_part_1		radio	Flatterable	1, Never or almost never true | 2, Usually not true | 3, Sometimes but infrequently true | 4, Occasionally true | 5, Often true | 6, Usually true | 7, Always or almost always true
**bsri_42**	day_2_part_1		radio	Theatrical	1, Never or almost never true | 2, Usually not true | 3, Sometimes but infrequently true | 4, Occasionally true | 5, Often true | 6, Usually true | 7, Always or almost always true
**bsri_43**	day_2_part_1		radio	Self-sufficient	1, Never or almost never true | 2, Usually not true | 3, Sometimes but infrequently true | 4, Occasionally true | 5, Often true | 6, Usually true | 7, Always or almost always true
**bsri_44**	day_2_part_1		radio	Loyal	1, Never or almost never true | 2, Usually not true | 3, Sometimes but infrequently true | 4, Occasionally true | 5, Often true | 6, Usually true | 7, Always or almost always true
**bsri_45**	day_2_part_1		radio	Happy	1, Never or almost never true | 2, Usually not true | 3, Sometimes but infrequently true | 4, Occasionally true | 5, Often true

					6, Usually true | 7, Always or almost always true
**bsri_46**	day_2_part_1		radio	Individualistic	1, Never or almost never true | 2, Usually not true | 3, Sometimes but infrequently true | 4, Occasionally true | 5, Often true | 6, Usually true | 7, Always or almost always true
**bsri_47**	day_2_part_1		radio	Soft-spoken	1, Never or almost never true | 2, Usually not true | 3, Sometimes but infrequently true | 4, Occasionally true | 5, Often true | 6, Usually true | 7, Always or almost always true
**bsri_48**	day_2_part_1		radio	Unpredictable	1, Never or almost never true | 2, Usually not true | 3, Sometimes but infrequently true | 4, Occasionally true | 5, Often true | 6, Usually true | 7, Always or almost always true
**bsri_49**	day_2_part_1		radio	Masculine	1, Never or almost never true | 2, Usually not true | 3, Sometimes but infrequently true | 4, Occasionally true | 5, Often true | 6, Usually true | 7, Always or almost always true
**bsri_50**	day_2_part_1		radio	Gullible	1, Never or almost never true | 2, Usually not true | 3, Sometimes but infrequently true | 4, Occasionally true | 5, Often true | 6, Usually true | 7, Always or almost always true
**bsri_51**	day_2_part_1		radio	Solemn	1, Never or almost never true | 2, Usually not true | 3, Sometimes but infrequently true | 4, Occasionally true | 5, Often true | 6, Usually true | 7, Always or almost always true

**bsri_52**	day_2_part_1		radio	Competitive	1, Never or almost never true | 2, Usually not true | 3, Sometimes but infrequently true | 4, Occasionally true | 5, Often true | 6, Usually true | 7, Always or almost always true
**bsri_53**	day_2_part_1		radio	Childlike	1, Never or almost never true | 2, Usually not true | 3, Sometimes but infrequently true | 4, Occasionally true | 5, Often true | 6, Usually true | 7, Always or almost always true
**bsri_54**	day_2_part_1		radio	Likable	1, Never or almost never true | 2, Usually not true | 3, Sometimes but infrequently true | 4, Occasionally true | 5, Often true | 6, Usually true | 7, Always or almost always true
**bsri_55**	day_2_part_1		radio	Ambitious	1, Never or almost never true | 2, Usually not true | 3, Sometimes but infrequently true | 4, Occasionally true | 5, Often true | 6, Usually true | 7, Always or almost always true
**bsri_56**	day_2_part_1		radio	Do not use harsh language	1, Never or almost never true | 2, Usually not true | 3, Sometimes but infrequently true | 4, Occasionally true | 5, Often true | 6, Usually true | 7, Always or almost always true
**bsri_57**	day_2_part_1		radio	Sincere	1, Never or almost never true | 2, Usually not true | 3, Sometimes but infrequently true | 4, Occasionally true | 5, Often true | 6, Usually true | 7, Always or almost always true
**bsri_58**	day_2_part_1		radio	Act as a leader	1, Never or almost never true | 2, Usually not true | 3, Sometimes but infrequently true | 4, Occasionally true | 5, Often true

					6, Usually true | 7, Always or almost always true
**bsri_59**	day_2_part_1		radio	Feminine	1, Never or almost never true | 2, Usually not true | 3, Sometimes but infrequently true | 4, Occasionally true | 5, Often true | 6, Usually true | 7, Always or almost always true
**bsri_60**	day_2_part_1		radio	Friendly	1, Never or almost never true | 2, Usually not true | 3, Sometimes but infrequently true | 4, Occasionally true | 5, Often true | 6, Usually true | 7, Always or almost always true
**mcc_1**	day_2_part_1	Memory<br>Instructions: Please read the statements below and chose the answer that best represents your behavior.	radio	Forgetting something you were told yesterday or a few days before. For example: Needing someone to remind you what you were told.	1, Never during the last 6 months | 2, Approximately once during the last 6 months | 3, Several times during the last 6 months BUT less than once per month | 4, Once per month | 5, More than once a month BUT less than once a week | 6, Once a week | 7, More than once a week BUT less than once a day | 8, Once a day | 9, More than once a day
**mcc_2**	day_2_part_1		radio	Forgetting important details regarding something you did or something that was done to you.	1, Never during the last 6 months | 2, Approximately once during the last 6 months | 3, Several times during the last 6 months BUT less than once per month | 4, Once per month | 5, More than once a month BUT less than once a week | 6, Once a week | 7, More than once a week BUT less than once a day | 8, Once a day | 9, More than once a day
**mcc_3**	day_2_part_1		radio	Forgetting things regarding you regular activities. For example: You remember that you go to bridge every Tuesday, but have difficulty remembering who you played with and what you talked about.	1, Never during the last 6 months | 2, Approximately once during the last 6 months | 3, Several times during the last 6 months BUT less than once per month | 4, Once per month | 5, More than once a month BUT less than once a week | 6, Once a week | 7, More than once a week

					BUT less than once a day | 8, Once a day | 9, More than once a day
**mcc_4**	day_2_part_1		radio	Forgetting or mixing up details from a past conversation.	1, Never during the last 6 months | 2, Approximately once during the last 6 months | 3, Several times during the last 6 months BUT less than once per month | 4, Once per month | 5, More than once a month BUT less than once a week | 6, Once a week | 7, More than once a week BUT less than once a day | 8, Once a day | 9, More than once a day
**mcc_5**	day_2_part_1		radio	Having to verify several times if you completed what you set out to do.	1, Never during the last 6 months | 2, Approximately once during the last 6 months | 3, Several times during the last 6 months BUT less than once per month | 4, Once per month | 5, More than once a month BUT less than once a week | 6, Once a week | 7, More than once a week BUT less than once a day | 8, Once a day | 9, More than once a day
**mcc_6**	day_2_part_1		radio	Forgetting where you've left things, losing objects in the house.	1, Never during the last 6 months | 2, Approximately once during the last 6 months | 3, Several times during the last 6 months BUT less than once per month | 4, Once per month | 5, More than once a month BUT less than once a week | 6, Once a week | 7, More than once a week BUT less than once a day | 8, Once a day | 9, More than once a day
**mcc_7**	day_2_part_1		radio	Not recognizing a place you have already visited.	1, Never during the last 6 months | 2, Approximately once during the last 6 months | 3, Several times during the last 6 months BUT less than once per month | 4, Once per month | 5, More than once a month BUT less than once a week | 6, Once a week | 7, More than once a week BUT less than once a day | 8, Once a day | 9, More than once a day

**mcc_8**	day_2_part_1		radio	Forgetting when an event took place. For example: Not certain whether an even took place today, yesterday, or a few days before.	1, Never during the last 6 months | 2, Approximately once during the last 6 months | 3, Several times during the last 6 months BUT less than once per month | 4, Once per month | 5, More than once a month BUT less than once a week | 6, Once a week | 7, More than once a week BUT less than once a day | 8, Once a day | 9, More than once a day
**mcc_9**	day_2_part_1		radio	Getting lost or losing you way in a place you have been to only a few times.	1, Never during the last 6 months | 2, Approximately once during the last 6 months | 3, Several times during the last 6 months BUT less than once per month | 4, Once per month | 5, More than once a month BUT less than once a week | 6, Once a week | 7, More than once a week BUT less than once a day | 8, Once a day | 9, More than once a day
**mcc_10**	day_2_part_1		radio	Doing something twice because you forgot you just did it. For example: Putting sugar in your coffee/tea twice.	1, Never during the last 6 months | 2, Approximately once during the last 6 months | 3, Several times during the last 6 months BUT less than once per month | 4, Once per month | 5, More than once a month BUT less than once a week | 6, Once a week | 7, More than once a week BUT less than once a day | 8, Once a day | 9, More than once a day
**mcc_11**	day_2_part_1		radio	Repeating something you've just said OR asking the same person the same question twice in a row.	1, Never during the last 6 months | 2, Approximately once during the last 6 months | 3, Several times during the last 6 months BUT less than once per month | 4, Once per month | 5, More than once a month BUT less than once a week | 6, Once a week | 7, More than once a week BUT less than once a day | 8, Once a day | 9, More than once a day
**mcc_12**	day_2_part_1		radio	Forgetting a change in your regular activities. For example: For the last 2	1, Never during the last 6 months | 2, Approximately once during the last 6

				years, the garbage had been picked up on Mondays and Fridays, but now, due to budget cuts, it is only picked up on Mondays.	months | 3, Several times during the last 6 months BUT less than once per month | 4, Once per month | 5, More than once a month BUT less than once a week | 6, Once a week | 7, More than once a week BUT less than once a day | 8, Once a day | 9, More than once a day
**mcc_13**	day_2_part_1		radio	Forgetting to bring something and having to go back to get it.	1, Never during the last 6 months | 2, Approximately once during the last 6 months | 3, Several times during the last 6 months BUT less than once per month | 4, Once per month | 5, More than once a month BUT less than once a week | 6, Once a week | 7, More than once a week BUT less than once a day | 8, Once a day | 9, More than once a day
**mcc_14**	day_2_part_1		radio	Forgetting what you were doing.	1, Never during the last 6 months | 2, Approximately once during the last 6 months | 3, Several times during the last 6 months BUT less than once per month | 4, Once per month | 5, More than once a month BUT less than once a week | 6, Once a week | 7, More than once a week BUT less than once a day | 8, Once a day | 9, More than once a day
**mcc_15**	day_2_part_1		radio	Forgetting to give someone an important message.	1, Never during the last 6 months | 2, Approximately once during the last 6 months | 3, Several times during the last 6 months BUT less than once per month | 4, Once per month | 5, More than once a month BUT less than once a week | 6, Once a week | 7, More than once a week BUT less than once a day | 8, Once a day | 9, More than once a day
**mcc_16**	day_2_part_1		radio	During a conversation, forgetting what you have just said. For example: Asking "What was I saying?"	1, Never during the last 6 months | 2, Approximately once during the last 6 months | 3, Several times during the last 6 months BUT less than once per month

					| 4, Once per month | 5, More than once a month BUT less than once a week | 6, Once a week | 7, More than once a week BUT less than once a day | 8, Once a day | 9, More than once a day
**mcc_17**	day_2_part_1		radio	Being unable to follow the flow of a story or article you are reading.	1, Never during the last 6 months | 2, Approximately once during the last 6 months | 3, Several times during the last 6 months BUT less than once per month | 4, Once per month | 5, More than once a month BUT less than once a week | 6, Once a week | 7, More than once a week BUT less than once a day | 8, Once a day | 9, More than once a day
**mcc_18**	day_2_part_1		radio	Have difficulty following a story on television.	1, Never during the last 6 months | 2, Approximately once during the last 6 months | 3, Several times during the last 6 months BUT less than once per month | 4, Once per month | 5, More than once a month BUT less than once a week | 6, Once a week | 7, More than once a week BUT less than once a day | 8, Once a day | 9, More than once a day
**mcc_19**	day_2_part_1		radio	Having to re-read a book or article before realizing that you've read it before.	1, Never during the last 6 months | 2, Approximately once during the last 6 months | 3, Several times during the last 6 months BUT less than once per month | 4, Once per month | 5, More than once a month BUT less than once a week | 6, Once a week | 7, More than once a week BUT less than once a day | 8, Once a day | 9, More than once a day
**mcc_20**	day_2_part_1		radio	During a conversation, you suddenly say things that have nothing to do with the discussion.	1, Never during the last 6 months | 2, Approximately once during the last 6 months | 3, Several times during the last 6 months BUT less than once per month | 4, Once per month | 5, More than once a month BUT less than once a week | 6,

					Once a week | 7, More than once a week BUT less than once a day | 8, Once a day | 9, More than once a day
**mcc_21**	day_2_part_1		radio	Difficulty recognizing family or relatives.	1, Never during the last 6 months | 2, Approximately once during the last 6 months | 3, Several times during the last 6 months BUT less than once per month | 4, Once per month | 5, More than once a month BUT less than once a week | 6, Once a week | 7, More than once a week BUT less than once a day | 8, Once a day | 9, More than once a day
**mcc_22**	day_2_part_1		radio	Forgetting important personal information such as your birth date or address.	1, Never during the last 6 months | 2, Approximately once during the last 6 months | 3, Several times during the last 6 months BUT less than once per month | 4, Once per month | 5, More than once a month BUT less than once a week | 6, Once a week | 7, More than once a week BUT less than once a day | 8, Once a day | 9, More than once a day
**mcc_23**	day_2_part_1		radio	Forgetting where you normally put things and having to search for them.	1, Never during the last 6 months | 2, Approximately once during the last 6 months | 3, Several times during the last 6 months BUT less than once per month | 4, Once per month | 5, More than once a month BUT less than once a week | 6, Once a week | 7, More than once a week BUT less than once a day | 8, Once a day | 9, More than once a day
**mcc_24**	day_2_part_1		radio	Getting lost or losing you way in a new area.	1, Never during the last 6 months | 2, Approximately once during the last 6 months | 3, Several times during the last 6 months BUT less than once per month | 4, Once per month | 5, More than once a month BUT less than once a week | 6, Once a week | 7, More than once a week

					BUT less than once a day | 8, Once a day | 9, More than once a day
**mcc_25**	day_2_part_1		radio	Having difficulty recognizing well known personalities. For example: TV celebrities.	1, Never during the last 6 months | 2, Approximately once during the last 6 months | 3, Several times during the last 6 months BUT less than once per month | 4, Once per month | 5, More than once a month BUT less than once a week | 6, Once a week | 7, More than once a week BUT less than once a day | 8, Once a day | 9, More than once a day
**mcc_26**	day_2_part_1		radio	Difficulty learning new things. For example: Learning a new game or how to use a new machine.	1, Never during the last 6 months | 2, Approximately once during the last 6 months | 3, Several times during the last 6 months BUT less than once per month | 4, Once per month | 5, More than once a month BUT less than once a week | 6, Once a week | 7, More than once a week BUT less than once a day | 8, Once a day | 9, More than once a day
**mcc_27**	day_2_part_1		radio	Having difficulty finding words. For example: Words are on the tip of your tongue but you just can't say them.	1, Never during the last 6 months | 2, Approximately once during the last 6 months | 3, Several times during the last 6 months BUT less than once per month | 4, Once per month | 5, More than once a month BUT less than once a week | 6, Once a week | 7, More than once a week BUT less than once a day | 8, Once a day | 9, More than once a day
**mcc_28**	day_2_part_1		radio	Telling the same person the same story twice.	1, Never during the last 6 months | 2, Approximately once during the last 6 months | 3, Several times during the last 6 months BUT less than once per month | 4, Once per month | 5, More than once a month BUT less than once a week | 6, Once a week | 7, More than once a week BUT less than once a day | 8, Once a day | 9, More than once a day

**mcc_29**	day_2_part_1		radio	Difficulty recognizing voices.	1, Never during the last 6 months | 2, Approximately once during the last 6 months | 3, Several times during the last 6 months BUT less than once per month | 4, Once per month | 5, More than once a month BUT less than once a week | 6, Once a week | 7, More than once a week BUT less than once a day | 8, Once a day | 9, More than once a day
**mcc_30**	day_2_part_1		radio	Difficulty remembering people's names.	1, Never during the last 6 months | 2, Approximately once during the last 6 months | 3, Several times during the last 6 months BUT less than once per month | 4, Once per month | 5, More than once a month BUT less than once a week | 6, Once a week | 7, More than once a week BUT less than once a day | 8, Once a day | 9, More than once a day
**mpss_frd_1**	day_2_part_1	Relationships:<br>Instructions: The following statements refer to feelings and experiences that occur to most people at one time or another in their relationships with friends. For each statement, there are four possible answers: strongly disagree, generally disagree, generally agree, and strongly agree. Please select the answer you choose for each item.	radio	My friends give me the moral support I need.	1, Strongly Disagree | 2, Generally Disagree | 3, Generally Agree | 4, Strongly Agree
**mpss_frd_2**	day_2_part_1		radio	Most other people are closer to their friends than I am.	1, Strongly Disagree | 2, Generally Disagree | 3, Generally Agree | 4, Strongly Agree
**mpss_frd_3**	day_2_part_1		radio	My friends enjoy hearing about what I think.	1, Strongly Disagree | 2, Generally Disagree | 3, Generally Agree | 4, Strongly Agree

**mpss_frd_4**	day_2_part_1		radio	Certain friends come to me when they have problems or need advice.	1, Strongly Disagree | 2, Generally Disagree | 3, Generally Agree | 4, Strongly Agree
**mpss_frd_5**	day_2_part_1		radio	I rely on my friends for emotional support.	1, Strongly Disagree | 2, Generally Disagree | 3, Generally Agree | 4, Strongly Agree
**mpss_frd_6**	day_2_part_1		radio	If I felt that one or more of my friends were upset with me, I'd just keep it to myself.	1, Strongly Disagree | 2, Generally Disagree | 3, Generally Agree | 4, Strongly Agree
**mpss_frd_7**	day_2_part_1		radio	I feel that I'm on the fringe in my circle of friends.	1, Strongly Disagree | 2, Generally Disagree | 3, Generally Agree | 4, Strongly Agree
**mpss_frd_8**	day_2_part_1		radio	There is a friend I could go to if I were just feeling down, without feeling funny about it later.	1, Strongly Disagree | 2, Generally Disagree | 3, Generally Agree | 4, Strongly Agree
**mpss_frd_9**	day_2_part_1		radio	My friends and I are very open about what we think about things.	1, Strongly Disagree | 2, Generally Disagree | 3, Generally Agree | 4, Strongly Agree
**mpss_frd_10**	day_2_part_1		radio	My friends are sensitive to my personal needs.	1, Strongly Disagree | 2, Generally Disagree | 3, Generally Agree | 4, Strongly Agree
**mpss_frd_11**	day_2_part_1		radio	My friends come to me for emotional support.	1, Strongly Disagree | 2, Generally Disagree | 3, Generally Agree | 4, Strongly Agree
**mpss_frd_12**	day_2_part_1		radio	My friends are good at helping me solve problems.	1, Strongly Disagree | 2, Generally Disagree | 3, Generally Agree | 4, Strongly Agree

**mpss_frd_13**	day_2_part_1		radio	I have a deep sharing relationship with a number of friends.	1, Strongly Disagree | 2, Generally Disagree | 3, Generally Agree | 4, Strongly Agree
**mpss_frd_14**	day_2_part_1		radio	My friends get good ideas about how to do things or make things from me.	1, Strongly Disagree | 2, Generally Disagree | 3, Generally Agree | 4, Strongly Agree
**mpss_frd_15**	day_2_part_1		radio	When I confide in friends, it makes me feel uncomfortable.	1, Strongly Disagree | 2, Generally Disagree | 3, Generally Agree | 4, Strongly Agree
**mpss_frd_16**	day_2_part_1		radio	My friends seek me out for companionship.	1, Strongly Disagree | 2, Generally Disagree | 3, Generally Agree | 4, Strongly Agree
**mpss_frd_17**	day_2_part_1		radio	I think that my friends feel that I am good at helping them solve problems.	1, Strongly Disagree | 2, Generally Disagree | 3, Generally Agree | 4, Strongly Agree
**mpss_frd_18**	day_2_part_1		radio	Other people's friend relationships are more intimate than mine.	1, Strongly Disagree | 2, Generally Disagree | 3, Generally Agree | 4, Strongly Agree
**mpss_frd_19**	day_2_part_1		radio	I've recently gotten a good idea about how to do something from a friend.	1, Strongly Disagree | 2, Generally Disagree | 3, Generally Agree | 4, Strongly Agree
**mpss_frd_20**	day_2_part_1		radio	I wish my friends were much different.	1, Strongly Disagree | 2, Generally Disagree | 3, Generally Agree | 4, Strongly Agree
**break**	day_2_part_1		descriptive	You are half way done; you may take a 10 minute break to use the restroom.	

2.3.2 Part 2 of Questionnaire Package

Redcap Form Name: Day 2 Part 2

Questionnaire Name	Title	Variable Name
2.3.2.1 Life Events	Life Events Questionnaire (LEQ)	leq
2.3.2.2 Perceived Stress	Multidimensional Scale of Perceived Social Support (MPSS)	mpss
2.3.2.3 Chronic Stress	Trier Inventory for the Assessment or Chronic Stress (TICS)	tics
2.3.2.4 Daily Hassles	Daily Hassles Scale (DHS)	dhs
2.3.2.5 Anxiety	State and Trait Anxiety Inventory (STAI-Y1)	stai_y1
2.3.2.6 Loneliness	Revised UCLA Loneliness Scale Version III (ULS-8)	ucla
2.3.2.7 Depressive Symptoms	Beck Depression Inventory II (BDI)	bdi
2.3.2.8 Burnout	Maslach Burnout Inventory (MBI)	mbi
2.3.2.9 PTSD	PTSD Check List – Civilian, PTSD Checklist for DSM-5 (PCL-C)	pcl

Variable / Field Name	Form Name	Section Header	Field Type	Field Label	Choices, Calculations, OR Slider Labels
**led**	day_2_part_2		descriptive	LIFE EVENTS QUESTIONNAIRE<br>Listed below are a number of events which may bring about changes in the lives of those who experience them<br>For each of the events listed below, please answer in three parts:<br>Select the events that have occurred in your life<br>If the event did occur in the past year, choose whether these were…<br>Indicate how much the event affected your life by selecting the appropriate statement (no effect, some effect, moderate effect, great effect).	

				If you have not experienced a particular event in the past year, leave it blank.	
**leq_1**	day_2_part_2	Health<br>Please choose whether any of the following events happened to you in the last year. If it did happen, please chose whether these were good or bad and how much the event affected you. If the event did not occur in the last year, leave it blank.	checkbox	Major personal illness or injury	8, Happened | 9, Good | 10, Bad | 0, No effect | 1, Some effect | 2, Moderate effect | 3, Great effect
**leq_2**	day_2_part_2		checkbox	Major change in eating habits	8, Happened | 9, Good | 10, Bad | 0, No effect | 1, Some effect | 2, Moderate effect | 3, Great effect
**leq_3**	day_2_part_2		checkbox	Major change in sleeping habits	8, Happened | 9, Good | 10, Bad | 0, No effect | 1, Some effect | 2, Moderate effect | 3, Great effect
**leq_4**	day_2_part_2		checkbox	Major change in usual type and/or amount of recreation	8, Happened | 9, Good | 10, Bad | 0, No effect | 1, Some effect | 2, Moderate effect | 3, Great effect
**leq_5**	day_2_part_2		checkbox	Major dental work	8, Happened | 9, Good | 10, Bad | 0, No effect | 1, Some effect | 2, Moderate effect | 3, Great effect
**leq_6**	day_2_part_2		checkbox	(Female) pregnancy	8, Happened | 9, Good | 10, Bad | 0, No effect | 1, Some effect | 2, Moderate effect | 3, Great effect
**leq_7**	day_2_part_2		checkbox	(Female) miscarriage or abortion	8, Happened | 9, Good | 10, Bad | 0, No effect | 1, Some effect | 2, Moderate effect | 3, Great effect

**leq_8**	day_2_part_2		checkbox	(Female) started menopause	8, Happened | 9, Good | 10, Bad | 0, No effect | 1, Some effect | 2, Moderate effect | 3, Great effect
**leq_9**	day_2_part_2		checkbox	Major difficulties with birth control pills or devices	8, Happened | 9, Good | 10, Bad | 0, No effect | 1, Some effect | 2, Moderate effect | 3, Great effect
**leq_10**	day_2_part_2	Work<br>Please choose whether any of the following events happened to you in the last year. If it did happen, please chose whether these were good or bad and how much the event affected you. If the event did not occur in the last year, leave it blank.	checkbox	Difficulty finding a job	8, Happened | 9, Good | 10, Bad | 0, No effect | 1, Some effect | 2, Moderate effect | 3, Great effect
**leq_11**	day_2_part_2		checkbox	Beginning work outside the home	8, Happened | 9, Good | 10, Bad | 0, No effect | 1, Some effect | 2, Moderate effect | 3, Great effect
**leq_12**	day_2_part_2		checkbox	Changing to a new type of work	8, Happened | 9, Good | 10, Bad | 0, No effect | 1, Some effect | 2, Moderate effect | 3, Great effect
**leq_13**	day_2_part_2		checkbox	Changing your work hours or conditions	8, Happened | 9, Good | 10, Bad | 0, No effect | 1, Some effect | 2, Moderate effect | 3, Great effect
**leq_14**	day_2_part_2		checkbox	Change in your responsibilities at work	8, Happened | 9, Good | 10, Bad | 0, No effect | 1, Some effect | 2, Moderate effect | 3, Great effect
**leq_15**	day_2_part_2		checkbox	Troubles at work with your employer or co-workers	8, Happened | 9, Good | 10, Bad | 0, No effect | 1, Some effect | 2, Moderate effect | 3, Great effect

**leq_16**	day_2_part_2		checkbox	Major business readjustment	8, Happened | 9, Good | 10, Bad | 0, No effect | 1, Some effect | 2, Moderate effect | 3, Great effect
**leq_17**	day_2_part_2		checkbox	Being fired or laid off from work	8, Happened | 9, Good | 10, Bad | 0, No effect | 1, Some effect | 2, Moderate effect | 3, Great effect
**leq_18**	day_2_part_2		checkbox	Retirement from work	8, Happened | 9, Good | 10, Bad | 0, No effect | 1, Some effect | 2, Moderate effect | 3, Great effect
**leq_19**	day_2_part_2		checkbox	Taking courses by mail or studying at home to help you in your work	8, Happened | 9, Good | 10, Bad | 0, No effect | 1, Some effect | 2, Moderate effect | 3, Great effect
**leq_20**	day_2_part_2	School<br>Please choose whether any of the following events happened to you in the last year. If it did happen, please chose whether these were good or bad and how much the event affected you. If the event did not occur in the last year, leave it blank.	checkbox	Beginning or ceasing school, college, or training program	8, Happened | 9, Good | 10, Bad | 0, No effect | 1, Some effect | 2, Moderate effect | 3, Great effect
**leq_21**	day_2_part_2		checkbox	Change of school, college, or training program	8, Happened | 9, Good | 10, Bad | 0, No effect | 1, Some effect | 2, Moderate effect | 3, Great effect
**leq_22**	day_2_part_2		checkbox	Change in career goal or academic major	8, Happened | 9, Good | 10, Bad | 0, No effect | 1, Some effect | 2, Moderate effect | 3, Great effect
**leq_23**	day_2_part_2		checkbox	Problem in school, college, or training program	8, Happened | 9, Good | 10, Bad | 0, No effect | 1, Some effect | 2, Moderate effect | 3, Great effect

**leq_24**	day_2_part_2	Residence<br>Please choose whether any of the following events happened to you in the last year. If it did happen, please chose whether these were good or bad and how much the event affected you. If the event did not occur in the last year, leave it blank.	checkbox	Difficulty finding housing	8, Happened | 9, Good | 10, Bad | 0, No effect | 1, Some effect | 2, Moderate effect | 3, Great effect
**leq_25**	day_2_part_2		checkbox	Changing residence within the same town or city	8, Happened | 9, Good | 10, Bad | 0, No effect | 1, Some effect | 2, Moderate effect | 3, Great effect
**leq_26**	day_2_part_2		checkbox	Moving to a different town, city, state, or country	8, Happened | 9, Good | 10, Bad | 0, No effect | 1, Some effect | 2, Moderate effect | 3, Great effect
**leq_27**	day_2_part_2		checkbox	Major change in your life conditions (home improvements or a decline in your home or neighborhood)	8, Happened | 9, Good | 10, Bad | 0, No effect | 1, Some effect | 2, Moderate effect | 3, Great effect
**leq_28**	day_2_part_2	Love and Marriage<br>Please choose whether any of the following events happened to you in the last year. If it did happen, please chose whether these were good or bad and how much the event affected you. If the event did not occur in the last year, leave it blank.	checkbox	Began a new, close, personal relationship	8, Happened | 9, Good | 10, Bad | 0, No effect | 1, Some effect | 2, Moderate effect | 3, Great effect
**leq_29**	day_2_part_2		checkbox	Became engaged	8, Happened | 9, Good | 10, Bad | 0, No effect | 1, Some effect | 2, Moderate effect | 3, Great effect
**leq_30**	day_2_part_2		checkbox	Girlfriend or boyfriend problems	8, Happened | 9, Good | 10, Bad | 0, No effect | 1, Some effect | 2, Moderate effect | 3, Great effect

**leq_31**	day_2_part_2		checkbox	Breaking up with a girlfriend or boyfriend or breaking an engagement	8, Happened | 9, Good | 10, Bad | 0, No effect | 1, Some effect | 2, Moderate effect | 3, Great effect
**leq_32**	day_2_part_2		checkbox	(Male) Wife or girlfriend's pregnancy	8, Happened | 9, Good | 10, Bad | 0, No effect | 1, Some effect | 2, Moderate effect | 3, Great effect
**leq_33**	day_2_part_2		checkbox	(Male) Wife or girlfriend having a miscarriage or abortion	8, Happened | 9, Good | 10, Bad | 0, No effect | 1, Some effect | 2, Moderate effect | 3, Great effect
**leq_34**	day_2_part_2		checkbox	Getting married (or beginning to live with someone)	8, Happened | 9, Good | 10, Bad | 0, No effect | 1, Some effect | 2, Moderate effect | 3, Great effect
**leq_35**	day_2_part_2		checkbox	A change in closeness with your partner	8, Happened | 9, Good | 10, Bad | 0, No effect | 1, Some effect | 2, Moderate effect | 3, Great effect
**leq_36**	day_2_part_2		checkbox	Infidelity	8, Happened | 9, Good | 10, Bad | 0, No effect | 1, Some effect | 2, Moderate effect | 3, Great effect
**leq_37**	day_2_part_2		checkbox	Trouble with in-laws	8, Happened | 9, Good | 10, Bad | 0, No effect | 1, Some effect | 2, Moderate effect | 3, Great effect
**leq_38**	day_2_part_2		checkbox	Separation from spouse or partner due to conflict	8, Happened | 9, Good | 10, Bad | 0, No effect | 1, Some effect | 2, Moderate effect | 3, Great effect
**leq_39**	day_2_part_2		checkbox	Separation from spouse or partner due to work, travel, etc.	8, Happened | 9, Good | 10, Bad | 0, No effect | 1, Some effect | 2, Moderate effect | 3, Great effect

**leq_40**	day_2_part_2		checkbox	Reconciliation with spouse or partner	8, Happened | 9, Good | 10, Bad | 0, No effect | 1, Some effect | 2, Moderate effect | 3, Great effect
**leq_41**	day_2_part_2		checkbox	Divorce	8, Happened | 9, Good | 10, Bad | 0, No effect | 1, Some effect | 2, Moderate effect | 3, Great effect
**leq_42**	day_2_part_2		checkbox	Change in your spouse or partner's work outside the home (beginning work, ceasing work, changing jobs, retirement, etc.)	8, Happened | 9, Good | 10, Bad | 0, No effect | 1, Some effect | 2, Moderate effect | 3, Great effect
**leq_43**	day_2_part_2	Family and Close Friends<br>Please choose whether any of the following events happened to you in the last year. If it did happen, please chose whether these were good or bad and how much the event affected you. If the event did not occur in the last year, leave it blank.	checkbox	Gain of a new family member (through birth, adoption, relative moving in, etc)	8, Happened | 9, Good | 10, Bad | 0, No effect | 1, Some effect | 2, Moderate effect | 3, Great effect
**leq_44**	day_2_part_2		checkbox	Child or family member leaving home (due to marriage, to attend college, or for some other reason)	8, Happened | 9, Good | 10, Bad | 0, No effect | 1, Some effect | 2, Moderate effect | 3, Great effect
**leq_45**	day_2_part_2		checkbox	Major change in the health or behavior of a family member or close friend (illness, accidents, drug or disciplinary problems, etc.)	8, Happened | 9, Good | 10, Bad | 0, No effect | 1, Some effect | 2, Moderate effect | 3, Great effect
**leq_46**	day_2_part_2		checkbox	Death of spouse or partner	8, Happened | 9, Good | 10, Bad | 0, No effect | 1, Some effect | 2, Moderate effect | 3, Great effect

**leq_47**	day_2_part_2		checkbox	Death of a child	8, Happened | 9, Good | 10, Bad | 0, No effect | 1, Some effect | 2, Moderate effect | 3, Great effect
**leq_48**	day_2_part_2		checkbox	Death of family member or close friend	8, Happened | 9, Good | 10, Bad | 0, No effect | 1, Some effect | 2, Moderate effect | 3, Great effect
**leq_49**	day_2_part_2		checkbox	Birth of a grandchild	8, Happened | 9, Good | 10, Bad | 0, No effect | 1, Some effect | 2, Moderate effect | 3, Great effect
**leq_50**	day_2_part_2		checkbox	Change in marital status of your parents	8, Happened | 9, Good | 10, Bad | 0, No effect | 1, Some effect | 2, Moderate effect | 3, Great effect
**leq_51**	day_2_part_2	Parenting<br>Please choose whether any of the following events happened to you in the last year. If it did happen, please chose whether these were good or bad and how much the event affected you. If the event did not occur in the last year, leave it blank.	checkbox	Change in child care arrangements	8, Happened | 9, Good | 10, Bad | 0, No effect | 1, Some effect | 2, Moderate effect | 3, Great effect
**leq_52**	day_2_part_2		checkbox	Conflicts with spouse or partner about parenting	8, Happened | 9, Good | 10, Bad | 0, No effect | 1, Some effect | 2, Moderate effect | 3, Great effect
**leq_53**	day_2_part_2		checkbox	Conflicts with child's grandparents (or other important person) about parenting	8, Happened | 9, Good | 10, Bad | 0, No effect | 1, Some effect | 2, Moderate effect | 3, Great effect
**leq_54**	day_2_part_2		checkbox	Taking on full responsibility for parenting as a single parent	8, Happened | 9, Good | 10, Bad | 0, No effect | 1, Some effect | 2, Moderate effect | 3, Great effect

**leq_55**	day_2_part_2		checkbox	Custody battles with former spouse or partner	8, Happened | 9, Good | 10, Bad | 0, No effect | 1, Some effect | 2, Moderate effect | 3, Great effect
**leq_56**	day_2_part_2	Personal or Social<br>Please choose whether any of the following events happened to you in the last year. If it did happen, please chose whether these were good or bad and how much the event affected you. If the event did not occur in the last year, leave it blank.	checkbox	Major personal achievement	8, Happened | 9, Good | 10, Bad | 0, No effect | 1, Some effect | 2, Moderate effect | 3, Great effect
**leq_57**	day_2_part_2		checkbox	Major decision regarding your immediate future	8, Happened | 9, Good | 10, Bad | 0, No effect | 1, Some effect | 2, Moderate effect | 3, Great effect
**leq_58**	day_2_part_2		checkbox	Change in your personal habits (your dress, life-style, hobbies, etc.)	8, Happened | 9, Good | 10, Bad | 0, No effect | 1, Some effect | 2, Moderate effect | 3, Great effect
**leq_59**	day_2_part_2		checkbox	Change in your religious beliefs	8, Happened | 9, Good | 10, Bad | 0, No effect | 1, Some effect | 2, Moderate effect | 3, Great effect
**leq_60**	day_2_part_2		checkbox	Change in your political beliefs	8, Happened | 9, Good | 10, Bad | 0, No effect | 1, Some effect | 2, Moderate effect | 3, Great effect
**leq_61**	day_2_part_2		checkbox	Loss or damage of personal property	8, Happened | 9, Good | 10, Bad | 0, No effect | 1, Some effect | 2, Moderate effect | 3, Great effect
**leq_62**	day_2_part_2		checkbox	Took a vacation	8, Happened | 9, Good | 10, Bad | 0, No effect | 1, Some effect | 2, Moderate effect | 3, Great effect

**leq_63**	day_2_part_2		checkbox	Took a trip other than a vacation	8, Happened | 9, Good | 10, Bad | 0, No effect | 1, Some effect | 2, Moderate effect | 3, Great effect
**leq_64**	day_2_part_2		checkbox	Change in family get-togethers	8, Happened | 9, Good | 10, Bad | 0, No effect | 1, Some effect | 2, Moderate effect | 3, Great effect
**leq_65**	day_2_part_2		checkbox	Change in your social activities (clubs, movies, visiting)	8, Happened | 9, Good | 10, Bad | 0, No effect | 1, Some effect | 2, Moderate effect | 3, Great effect
**leq_66**	day_2_part_2		checkbox	Made new friends	8, Happened | 9, Good | 10, Bad | 0, No effect | 1, Some effect | 2, Moderate effect | 3, Great effect
**leq_67**	day_2_part_2		checkbox	Broke up with a friend	8, Happened | 9, Good | 10, Bad | 0, No effect | 1, Some effect | 2, Moderate effect | 3, Great effect
**leq_68**	day_2_part_2		checkbox	Acquired or lost a pet	8, Happened | 9, Good | 10, Bad | 0, No effect | 1, Some effect | 2, Moderate effect | 3, Great effect
**leq_69**	day_2_part_2	Financial<br>Please choose whether any of the following events happened to you in the last year. If it did happen, please chose whether these were good or bad and how much the event affected you. If the event did not occur in the last year, leave it blank.	checkbox	Major change in finances (increased or decreased income)	8, Happened | 9, Good | 10, Bad | 0, No effect | 1, Some effect | 2, Moderate effect | 3, Great effect
**leq_70**	day_2_part_2		checkbox	Took on a moderate purchase, such as TV, car, freezer, etc.	8, Happened | 9, Good | 10, Bad | 0, No effect | 1, Some effect | 2, Moderate effect | 3, Great effect

**leq_71**	day_2_part_2		checkbox	Took on a major purchase or a mortgage loan, such as a home, business, property, etc.	8, Happened | 9, Good | 10, Bad | 0, No effect | 1, Some effect | 2, Moderate effect | 3, Great effect
**leq_72**	day_2_part_2		checkbox	Experienced a foreclosure on a mortgage or loan	8, Happened | 9, Good | 10, Bad | 0, No effect | 1, Some effect | 2, Moderate effect | 3, Great effect
**leq_73**	day_2_part_2		checkbox	Credit rating difficulties	8, Happened | 9, Good | 10, Bad | 0, No effect | 1, Some effect | 2, Moderate effect | 3, Great effect
**leq_74**	day_2_part_2	Crime and Legal Matters<br>Please choose whether any of the following events happened to you in the last year. If it did happen, please chose whether these were good or bad and how much the event affected you. If the event did not occur in the last year, leave it blank.	checkbox	Being robbed or victim of identity theft	8, Happened | 9, Good | 10, Bad | 0, No effect | 1, Some effect | 2, Moderate effect | 3, Great effect
**leq_75**	day_2_part_2		checkbox	Being a victim of a violent act (rape, assault, etc.)	8, Happened | 9, Good | 10, Bad | 0, No effect | 1, Some effect | 2, Moderate effect | 3, Great effect
**leq_76**	day_2_part_2		checkbox	Involved in an accident	8, Happened | 9, Good | 10, Bad | 0, No effect | 1, Some effect | 2, Moderate effect | 3, Great effect
**leq_77**	day_2_part_2		checkbox	Involved in a law suit	8, Happened | 9, Good | 10, Bad | 0, No effect | 1, Some effect | 2, Moderate effect | 3, Great effect
**leq_78**	day_2_part_2		checkbox	Involved in a minor violation of the law (traffic tickets, disturbing the peace, etc.)	8, Happened | 9, Good | 10, Bad | 0, No effect | 1, Some effect | 2, Moderate effect | 3, Great effect

**leq_79**	day_2_part_2		checkbox	Legal troubles resulting in your being arrested or held in jail	8, Happened | 9, Good | 10, Bad | 0, No effect | 1, Some effect | 2, Moderate effect | 3, Great effect
**leq_80**	day_2_part_2		text	OTHER: Other recent experiences which have had an impact on your life.	
**leq_81**	day_2_part_2		text	OTHER: Other recent experiences which have had an impact on your life.	
**leq_82**	day_2_part_2		text	OTHER: Other recent experiences which have had an impact on your life.	
**pss1**	day_2_part_2	How I am feeling<br>Instructions: The following questions relate to your feelings and thoughts during the last month.<br>Please indicate how often you were thinking or feeling in a certain way. Please answer each question individually and as quickly as possible. Do not try to count the times you had a certain feeling, just make a guess which answer seems to be the best.<br>Please choose one of the following answers for each question:	radio	How often have you been upset because of something that happened unexpectedly?	0, Never | 1, Almost Never | 2, Sometimes | 3, Fairly Often | 4, Very Often
**pss2**	day_2_part_2		radio	How often have you felt unable to control the important things in your life?	0, Never | 1, Almost Never | 2, Sometimes | 3, Fairly Often | 4, Very Often
**pss3**	day_2_part_2		radio	How often have you felt nervous or stressed?	0, Never | 1, Almost Never | 2, Sometimes | 3, Fairly Often | 4, Very Often

**pss4**	day_2_part_2		radio	How often have you felt confident about your ability to handle personal problems?	0, Never | 1, Almost Never | 2, Sometimes | 3, Fairly Often | 4, Very Often
**pss5**	day_2_part_2		radio	How often have you felt that things were going your way?	0, Never | 1, Almost Never | 2, Sometimes | 3, Fairly Often | 4, Very Often
**pss6**	day_2_part_2		radio	How often have you found that you could not cope with all the things you had to do?	0, Never | 1, Almost Never | 2, Sometimes | 3, Fairly Often | 4, Very Often
**pss7**	day_2_part_2		radio	How often have you been able to control irritations in your life?	0, Never | 1, Almost Never | 2, Sometimes | 3, Fairly Often | 4, Very Often
**pss8**	day_2_part_2		radio	How often have you felt that you were on top of things?	0, Never | 1, Almost Never | 2, Sometimes | 3, Fairly Often | 4, Very Often
**pss9**	day_2_part_2		radio	How often have you been angered because of things that happened that were outside of your control?	0, Never | 1, Almost Never | 2, Sometimes | 3, Fairly Often | 4, Very Often
**pss10**	day_2_part_2		radio	How often have you felt that difficulties were piling up so high that you could not overcome them?	0, Never | 1, Almost Never | 2, Sometimes | 3, Fairly Often | 4, Very Often
**tics_1**	day_2_part_2	General Stress<br>In the past month, I experienced this...	radio	There were times when I put off getting some rest, even though I needed it	0, Never | 1, Almost Never | 2, Sometimes | 3, Fairly Often | 4, Very Often
**tics_2**	day_2_part_2		radio	I didn't receive enough appreciation for my accomplishments	0, Never | 1, Almost Never | 2, Sometimes | 3, Fairly Often | 4, Very Often

**tics_3**	day_2_part_2		radio	I didn't have enough time to perform my daily tasks/duties	0, Never | 1, Almost Never | 2, Sometimes | 3, Fairly Often | 4, Very Often
**tics_4**	day_2_part_2		radio	I had a differences of opinion, which led to tension with others	0, Never | 1, Almost Never | 2, Sometimes | 3, Fairly Often | 4, Very Often
**tics_5**	day_2_part_2		radio	My work involved assuming a lot of responsibility for others	0, Never | 1, Almost Never | 2, Sometimes | 3, Fairly Often | 4, Very Often
**tics_6**	day_2_part_2		radio	There were situations in which I had to make an effort to win people's trust	0, Never | 1, Almost Never | 2, Sometimes | 3, Fairly Often | 4, Very Often
**tics_7**	day_2_part_2		radio	I worried that something bad would happen	0, Never | 1, Almost Never | 2, Sometimes | 3, Fairly Often | 4, Very Often
**tics_8**	day_2_part_2		radio	I didn't have interesting tasks to fill my day	0, Never | 1, Almost Never | 2, Sometimes | 3, Fairly Often | 4, Very Often
**tics_9**	day_2_part_2		radio	I had conflicts with others because they had different goals	0, Never | 1, Almost Never | 2, Sometimes | 3, Fairly Often | 4, Very Often
**tics_10**	day_2_part_2		radio	There were times when I couldn't stop thinking about things that worried me	0, Never | 1, Almost Never | 2, Sometimes | 3, Fairly Often | 4, Very Often
**tics_11**	day_2_part_2		radio	I spend a lot of time dealing with other people's problems	0, Never | 1, Almost Never | 2, Sometimes | 3, Fairly Often | 4, Very Often

**tics_12**	day_2_part_2		radio	Despite doing my best I performed my tasks/duties poorly	0, Never | 1, Almost Never | 2, Sometimes | 3, Fairly Often | 4, Very Often
**tics_13**	day_2_part_2		radio	There where times when I had no meaningful tasks/duties	0, Never | 1, Almost Never | 2, Sometimes | 3, Fairly Often | 4, Very Often
**tics_14**	day_2_part_2		radio	I could not disappoint others with the work I had to do	0, Never | 1, Almost Never | 2, Sometimes | 3, Fairly Often | 4, Very Often
**tics_15**	day_2_part_2		radio	I had to make a good impression on the people I had contact with	0, Never | 1, Almost Never | 2, Sometimes | 3, Fairly Often | 4, Very Often
**tics_16**	day_2_part_2		radio	I could no longer cope with the demands of my work	0, Never | 1, Almost Never | 2, Sometimes | 3, Fairly Often | 4, Very Often
**tics_17**	day_2_part_2		radio	There were times when my worries overwhelmed me	0, Never | 1, Almost Never | 2, Sometimes | 3, Fairly Often | 4, Very Often
**tics_18**	day_2_part_2		radio	Dealing with other people's problems took up too much of my time	0, Never | 1, Almost Never | 2, Sometimes | 3, Fairly Often | 4, Very Often
**tics_19**	day_2_part_2		radio	There were times when I didn't have an opportunity to share my thoughts and feelings with others	0, Never | 1, Almost Never | 2, Sometimes | 3, Fairly Often | 4, Very Often
**tics_20**	day_2_part_2		radio	Even though I did my best, my work was not appreciated	0, Never | 1, Almost Never | 2, Sometimes | 3, Fairly Often | 4, Very Often

**tics_21**	day_2_part_2		radio	There were times when I had too many tasks/duties	0, Never | 1, Almost Never | 2, Sometimes | 3, Fairly Often | 4, Very Often
**tics_22**	day_2_part_2		radio	There were situations in which I had to try hard for people to like me	0, Never | 1, Almost Never | 2, Sometimes | 3, Fairly Often | 4, Very Often
**tics_23**	day_2_part_2		radio	I was not adequately rewarded for my efforts	0, Never | 1, Almost Never | 2, Sometimes | 3, Fairly Often | 4, Very Often
**tics_24**	day_2_part_2		radio	I had to do work where my skills were not used properly	0, Never | 1, Almost Never | 2, Sometimes | 3, Fairly Often | 4, Very Often
**tics_25**	day_2_part_2		radio	There were times when I longed to have contact with others	0, Never | 1, Almost Never | 2, Sometimes | 3, Fairly Often | 4, Very Often
**tics_26**	day_2_part_2		radio	I had unnecessary conflicts with others	0, Never | 1, Almost Never | 2, Sometimes | 3, Fairly Often | 4, Very Often
**tics_27**	day_2_part_2		radio	Even though I tried, I did not fulfill my duties as I should have	0, Never | 1, Almost Never | 2, Sometimes | 3, Fairly Often | 4, Very Often
**tics_28**	day_2_part_2		radio	There were times when having responsibilities for others became a burden for me	0, Never | 1, Almost Never | 2, Sometimes | 3, Fairly Often | 4, Very Often
**tics_29**	day_2_part_2		radio	I had tasks to do that allowed no mistakes at all	0, Never | 1, Almost Never | 2, Sometimes | 3, Fairly Often | 4, Very Often

**tics_30**	day_2_part_2		radio	There were times when I had no friends with whom I could do things	0, Never | 1, Almost Never | 2, Sometimes | 3, Fairly Often | 4, Very Often
**dhs_1**	day_2_part_2	Daily Stress<br>Instructions: "Hassles" are events that make you feel bothered or irritated. Read each item below and then choose the answer that best corresponds to how often each hassle occurred in the last month. If a hassle did not occur in the last month or does not apply, select "Did not occur."	radio	Health or well-being of a family member	1, Did not occur | 2, Sometimes | 3, Moderately often | 4, Often | 5, Extremely Often
**dhs_2**	day_2_part_2		radio	Enough money for emergencies	1, Did not occur | 2, Sometimes | 3, Moderately often | 4, Often | 5, Extremely Often
**dhs_3**	day_2_part_2		radio	Difficulty maintaining household	1, Did not occur | 2, Sometimes | 3, Moderately often | 4, Often | 5, Extremely Often
**dhs_4**	day_2_part_2		radio	Enough money for necessities	1, Did not occur | 2, Sometimes | 3, Moderately often | 4, Often | 5, Extremely Often
**dhs_5**	day_2_part_2		radio	Too much free time	1, Did not occur | 2, Sometimes | 3, Moderately often | 4, Often | 5, Extremely Often
**dhs_6**	day_2_part_2		radio	Fear of accidents	1, Did not occur | 2, Sometimes | 3, Moderately often | 4, Often | 5, Extremely Often
**dhs_7**	day_2_part_2		radio	Shopping is a problem	1, Did not occur | 2, Sometimes | 3, Moderately often | 4, Often | 5, Extremely Often

**dhs_8**	day_2_part_2		radio	Solitude	1, Did not occur | 2, Sometimes | 3, Moderately often | 4, Often | 5, Extremely Often
**dhs_9**	day_2_part_2		radio	Concern for your physical appearance	1, Did not occur | 2, Sometimes | 3, Moderately often | 4, Often | 5, Extremely Often
**dhs_10**	day_2_part_2		radio	Fear of being rejected	1, Did not occur | 2, Sometimes | 3, Moderately often | 4, Often | 5, Extremely Often
**dhs_11**	day_2_part_2		radio	Concerns about general health	1, Did not occur | 2, Sometimes | 3, Moderately often | 4, Often | 5, Extremely Often
**dhs_12**	day_2_part_2		radio	Not seeing enough people	1, Did not occur | 2, Sometimes | 3, Moderately often | 4, Often | 5, Extremely Often
**dhs_13**	day_2_part_2		radio	Concerns about friends or extended family	1, Did not occur | 2, Sometimes | 3, Moderately often | 4, Often | 5, Extremely Often
**dhs_14**	day_2_part_2		radio	Disturbed by noise	1, Did not occur | 2, Sometimes | 3, Moderately often | 4, Often | 5, Extremely Often
**dhs_15**	day_2_part_2		radio	Preoccupied by deterioration in neighborhood	1, Did not occur | 2, Sometimes | 3, Moderately often | 4, Often | 5, Extremely Often
**dhs_16**	day_2_part_2		radio	Feel that my physical abilities have decreased	1, Did not occur | 2, Sometimes | 3, Moderately often | 4, Often | 5, Extremely Often

**dhs_17**	day_2_part_2		radio	I have been taken advantage of	1, Did not occur | 2, Sometimes | 3, Moderately often | 4, Often | 5, Extremely Often
**dhs_18**	day_2_part_2		radio	Bothered by the increase in the price of commodities	1, Did not occur | 2, Sometimes | 3, Moderately often | 4, Often | 5, Extremely Often
**dhs_19**	day_2_part_2		radio	Problems with aging parents	1, Did not occur | 2, Sometimes | 3, Moderately often | 4, Often | 5, Extremely Often
**dhs_20**	day_2_part_2		radio	Problems with my children	1, Did not occur | 2, Sometimes | 3, Moderately often | 4, Often | 5, Extremely Often
**dhs_21**	day_2_part_2		radio	Bothered by traffic	1, Did not occur | 2, Sometimes | 3, Moderately often | 4, Often | 5, Extremely Often
**dhs_22**	day_2_part_2		radio	Problems with people younger than me	1, Did not occur | 2, Sometimes | 3, Moderately often | 4, Often | 5, Extremely Often
**dhs_23**	day_2_part_2		radio	Too many things to do	1, Did not occur | 2, Sometimes | 3, Moderately often | 4, Often | 5, Extremely Often
**dhs_24**	day_2_part_2		radio	Preoccupied with my weight	1, Did not occur | 2, Sometimes | 3, Moderately often | 4, Often | 5, Extremely Often
**dhs_25**	day_2_part_2		radio	Lack of energy	1, Did not occur | 2, Sometimes | 3, Moderately often | 4, Often | 5, Extremely Often

**dhs_26**	day_2_part_2		radio	Regrets over decisions made in the past	1, Did not occur | 2, Sometimes | 3, Moderately often | 4, Often | 5, Extremely Often
**dhs_27**	day_2_part_2		radio	Problems with my friends	1, Did not occur | 2, Sometimes | 3, Moderately often | 4, Often | 5, Extremely Often
**dhs_28**	day_2_part_2		radio	Problems with disturbing neighbors	1, Did not occur | 2, Sometimes | 3, Moderately often | 4, Often | 5, Extremely Often
**dhs_29**	day_2_part_2		radio	Transportation problems	1, Did not occur | 2, Sometimes | 3, Moderately often | 4, Often | 5, Extremely Often
**dhs_30**	day_2_part_2		radio	Not enough money for social events or hobbies	1, Did not occur | 2, Sometimes | 3, Moderately often | 4, Often | 5, Extremely Often
**dhs_31**	day_2_part_2		radio	Problems with property, taxes, or investments	1, Did not occur | 2, Sometimes | 3, Moderately often | 4, Often | 5, Extremely Often
**dhs_32**	day_2_part_2		radio	Problems maintaining exterior of house and yard	1, Did not occur | 2, Sometimes | 3, Moderately often | 4, Often | 5, Extremely Often
**dhs_33**	day_2_part_2		radio	Preoccupied with crime	1, Did not occur | 2, Sometimes | 3, Moderately often | 4, Often | 5, Extremely Often
**dhs_34**	day_2_part_2		radio	Objects lost or misplaced	1, Did not occur | 2, Sometimes | 3, Moderately often | 4, Often | 5, Extremely Often

**dhs_35**	day_2_part_2		radio	Concerns about being dependent on others	1, Did not occur | 2, Sometimes | 3, Moderately often | 4, Often | 5, Extremely Often
**staiy1_secure_02**	day_2_part_2	My Life Stress 2<br>Instructions: A number of statements which people have used to describe themselves are given below. Read each statement and then press the appropriate key to indicate how you feel RIGHT NOW, that is, AT THIS MOMENT. There are no right or wrong answers. Do not spend too much time on any one statement but give the answer which seems to describe your present feelings best.	radio	I feel secure.	1, Not at all | 2, Somewhat | 3, Moderately so | 4, Very much so
**staiy1_tense_03**	day_2_part_2		radio	I feel tense.	1, Not at all | 2, Somewhat | 3, Moderately so | 4, Very much so
**staiy1_calm_01**	day_2_part_2		radio	I feel calm.	1, Not at all | 2, Somewhat | 3, Moderately so | 4, Very much so
**staiy1_strain_04**	day_2_part_2		radio	I feel strained.	1, Not at all | 2, Somewhat | 3, Moderately so | 4, Very much so
**staiy1_ease_05**	day_2_part_2		radio	I feel at ease.	1, Not at all | 2, Somewhat | 3, Moderately so | 4, Very much so
**staiy1_upset_06**	day_2_part_2		radio	I feel upset.	1, Not at all | 2, Somewhat | 3, Moderately so | 4, Very much so
**staiy1_worry_07**	day_2_part_2		radio	I am presently worrying over possible misfortunes.	1, Not at all | 2, Somewhat | 3, Moderately so | 4, Very much so
**staiy1_satis_08**	day_2_part_2		radio	I feel satisfied.	1, Not at all | 2, Somewhat | 3, Moderately so | 4, Very much so

**staiy1_fright_09**	day_2_part_2		radio	I feel frightened.	1, Not at all | 2, Somewhat | 3, Moderately so | 4, Very much so
**staiy1_comf_10**	day_2_part_2		radio	I feel comfortable.	1, Not at all | 2, Somewhat | 3, Moderately so | 4, Very much so
**staiy1_self_11**	day_2_part_2		radio	I feel self-confident.	1, Not at all | 2, Somewhat | 3, Moderately so | 4, Very much so
**staiy1_nerv_12**	day_2_part_2		radio	I feel nervous.	1, Not at all | 2, Somewhat | 3, Moderately so | 4, Very much so
**staiy1_jittery_13**	day_2_part_2		radio	I feel jittery.	1, Not at all | 2, Somewhat | 3, Moderately so | 4, Very much so
**staiy1_indec_14**	day_2_part_2		radio	I feel indecisive.	1, Not at all | 2, Somewhat | 3, Moderately so | 4, Very much so
**staiy1_relax_15**	day_2_part_2		radio	I am relaxed.	1, Not at all | 2, Somewhat | 3, Moderately so | 4, Very much so
**staiy1_content_16**	day_2_part_2		radio	I feel content.	1, Not at all | 2, Somewhat | 3, Moderately so | 4, Very much so
**staiy1_worry_17**	day_2_part_2		radio	I am worried.	1, Not at all | 2, Somewhat | 3, Moderately so | 4, Very much so
**staiy1_confuse_18**	day_2_part_2		radio	I feel confused.	1, Not at all | 2, Somewhat | 3, Moderately so | 4, Very much so
**staiy1_steady_19**	day_2_part_2		radio	I feel steady.	1, Not at all | 2, Somewhat | 3, Moderately so | 4, Very much so
**staiy1_pleasant_20**	day_2_part_2		radio	I feel pleasant.	1, Not at all | 2, Somewhat | 3, Moderately so | 4, Very much so

**stai_y1t_pleasant_01**	day_2_part_2	Directions: A number of statements which people have used to describe themselves are given below. Read each statement and then press the appropriate key to indicate how you GENERALLY FEEL.	radio	I feel pleasant.	1, Almost Never | 2, Sometimes | 3, Often | 4, Almost Always
**stai_y1t_restless_02**	day_2_part_2		radio	I feel nervous and restless.	1, Almost Never | 2, Sometimes | 3, Often | 4, Almost Always
**stai_y1t_satis_03**	day_2_part_2		radio	I feel satisfied with myself.	1, Almost Never | 2, Sometimes | 3, Often | 4, Almost Always
**stai_y1t_happy_04**	day_2_part_2		radio	I wish I could be as happy as others seem to be.	1, Almost Never | 2, Sometimes | 3, Often | 4, Almost Always
**stai_y1t_failure_05**	day_2_part_2		radio	I feel like a failure.	1, Almost Never | 2, Sometimes | 3, Often | 4, Almost Always
**stai_y1t_rested_06**	day_2_part_2		radio	I feel rested.	1, Almost Never | 2, Sometimes | 3, Often | 4, Almost Always
**stai_y1_cool_07**	day_2_part_2		radio	I am "calm, cool, and collected".	1, Almost Never | 2, Sometimes | 3, Often | 4, Almost Always
**stai_y1_pile_08**	day_2_part_2		radio	I feel that difficulties are piling up so that I cannot overcome them.	1, Almost Never | 2, Sometimes | 3, Often | 4, Almost Always
**stai_y1_worry_09**	day_2_part_2		radio	I worry too much over something that really doesn't matter.	1, Almost Never | 2, Sometimes | 3, Often | 4, Almost Always
**stai_y1_happy_10**	day_2_part_2		radio	I am happy.	1, Almost Never | 2, Sometimes | 3, Often | 4, Almost Always
**stai_y1_dist_11**	day_2_part_2		radio	I have disturbing thoughts.	1, Almost Never | 2, Sometimes | 3, Often | 4, Almost Always

**stai_y1_self_12**	day_2_part_2		radio	I lack self-confidence.	1, Almost Never | 2, Sometimes | 3, Often | 4, Almost Always
**stai_y1_secure_13**	day_2_part_2		radio	I feel secure.	1, Almost Never | 2, Sometimes | 3, Often | 4, Almost Always
**stai_y1_easy_14**	day_2_part_2		radio	I make decisions easily.	1, Almost Never | 2, Sometimes | 3, Often | 4, Almost Always
**stai_y1_inad_15**	day_2_part_2		radio	I feel inadequate.	1, Almost Never | 2, Sometimes | 3, Often | 4, Almost Always
**stai_y1_content_16**	day_2_part_2		radio	I feel content.	1, Almost Never | 2, Sometimes | 3, Often | 4, Almost Always
**stai_y1_unimp_17**	day_2_part_2		radio	Some unimportant thought runs through my mind and bothers me.	1, Almost Never | 2, Sometimes | 3, Often | 4, Almost Always
**stai_y1_disapp_18**	day_2_part_2		radio	I take disappointments so keenly that I can't put them out of my mind.	1, Almost Never | 2, Sometimes | 3, Often | 4, Almost Always
**stai_y1_steady_19**	day_2_part_2		radio	I am a steady person.	1, Almost Never | 2, Sometimes | 3, Often | 4, Almost Always
**stai_y1_concern_20**	day_2_part_2		radio	I get in a state of tension or turmoil as I think over my recent concerns and interests.	1, Almost Never | 2, Sometimes | 3, Often | 4, Almost Always
**ucla_1**	day_2_part_2	Social Relationships<br>Instructions: Indicate how often each of the statements below pertains to you.	radio	How often do you feel that you are "in tune" with the people around you?	1, Never | 2, Rarely | 3, Sometimes | 4, Often
**ucla_2**	day_2_part_2		radio	How often do you feel that you lack companionship?	1, Never | 2, Rarely | 3, Sometimes | 4, Often

**ucla_3**	day_2_part_2		radio	How often do you feel that there is no one you can turn to?	1, Never | 2, Rarely | 3, Sometimes | 4, Often
**ucla_4**	day_2_part_2		radio	How often do you feel alone?	1, Never | 2, Rarely | 3, Sometimes | 4, Often
**ucla_5**	day_2_part_2		radio	How often do you feel part of a group of friends?	1, Never | 2, Rarely | 3, Sometimes | 4, Often
**ucla_6**	day_2_part_2		radio	How often do you feel that you have a lot in common with the people around you?	1, Never | 2, Rarely | 3, Sometimes | 4, Often
**ucla_7**	day_2_part_2		radio	How often do you feel that you are no longer close to anyone?	1, Never | 2, Rarely | 3, Sometimes | 4, Often
**ucla_8**	day_2_part_2		radio	How often do you feel that your interests and ideas are not shared by those around you?	1, Never | 2, Rarely | 3, Sometimes | 4, Often
**ucla_9**	day_2_part_2		radio	How often do you feel outgoing and friendly?	1, Never | 2, Rarely | 3, Sometimes | 4, Often
**ucla_10**	day_2_part_2		radio	How often do you feel close to people?	1, Never | 2, Rarely | 3, Sometimes | 4, Often
**ucla_11**	day_2_part_2		radio	How often do you feel left out?	1, Never | 2, Rarely | 3, Sometimes | 4, Often
**ucla_12**	day_2_part_2		radio	How often do you feel that your relationships with others are not meaningful?	1, Never | 2, Rarely | 3, Sometimes | 4, Often
**ucla_13**	day_2_part_2		radio	How often do you feel that no one really knows you well?	1, Never | 2, Rarely | 3, Sometimes | 4, Often

**ucla_14**	day_2_part_2		radio	How often do you feel isolated from others?	1, Never | 2, Rarely | 3, Sometimes | 4, Often
**ucla_15**	day_2_part_2		radio	How often do you feel you can find companionship when you want it?	1, Never | 2, Rarely | 3, Sometimes | 4, Often
**ucla_16**	day_2_part_2		radio	How often do you feel that there are people who really understand you?	1, Never | 2, Rarely | 3, Sometimes | 4, Often
**ucla_17**	day_2_part_2		radio	How often do you feel shy?	1, Never | 2, Rarely | 3, Sometimes | 4, Often
**ucla_18**	day_2_part_2		radio	How often do you feel that people are around you but not with you?	1, Never | 2, Rarely | 3, Sometimes | 4, Often
**ucla_19**	day_2_part_2		radio	How often do you feel that there are people you can talk to?	1, Never | 2, Rarely | 3, Sometimes | 4, Often
**ucla_20**	day_2_part_2		radio	How often do you feel that there are people you can turn to?	1, Never | 2, Rarely | 3, Sometimes | 4, Often
**bdi_sad_01**	day_2_part_2	Sad Mood and Symptoms<br>Instructions: This questionnaire consists of 21 groups of statements. Please read each group of statements carefully, and pick out the one statement in each group that best describes the way you have been feeling during the past two weeks, including today. If several statements in the group seem to apply equally well, choose the highest number for that group.	radio		0, I do not feel sad. | 1, I feel sad. | 2, I am sad all the time and I can't snap out of it. | 3, I am so sad or unhappy that I can't stand it.
**bdi_future_02**	day_2_part_2		radio		0, I am not particularly discouraged about the future. | 1, I feel discouraged

					about the future. | 2, I feel I have nothing to look forward to. | 3, I feel that the future is hopeless and that things can't improve.
**bdi_fail_03**	day_2_part_2		radio		0, I do not feel like a failure. | 1, I feel I have failed more than the average person. | 2, As I look back on my life, all I can see is a lot of failures. | 3, I feel I am a complete failure as a person.
**bdi_satis_04**	day_2_part_2		radio		0, I get as much satisfaction out of things as I used to. | 1, I don't enjoy things the way I used to. | 2, I don't get real satisfaction out of anything anymore. | 3, I am dissatisfied or bored with everything.
**bdi_guilt_05**	day_2_part_2		radio		0, I don't feel particularly guilty. | 1, I fee[illegible] guilty a good part of the time. | 2, I feel quite guilty most of the time. | 3, I feel guilty all of the time.
**bdi_pun_06**	day_2_part_2		radio		0, I don't feel I am being punished. | 1, I feel I may be punished. | 2, I expect to be punished. | 3, I feel I am being punished.
**bdi_dis_07**	day_2_part_2		radio		0, I don't feel disappointed in myself. | 1[illegible] I am disappointed in myself. | 2, I am disgusted with myself. | 3, I hate myself[illegible]
**bdi_worse_08**	day_2_part_2		radio		0, I don't feel I am worse than anybody else. | 1, I am critical of myself for my weaknesses or mistakes. | 2, I blame myself all the time for my faults. | 3, I blame myself for everything bad that happens.

**bdi_suic_09**	day_2_part_2		radio		0, I don't have any thoughts of killing myself. | 1, I have thoughts of killing myself, but I would not carry them out. | 2, I would like to kill myself. | 3, I would kill myself if I had the chance.
**bdi_cry_10**	day_2_part_2		radio		0, I don't cry anymore than usual. | 1, I cry more now than I used to. | 2, I cry all the time now. | 3, I used to be able to cry,but now I can't cry even though I want to.
**bdi_irrit_11**	day_2_part_2		radio		0, I am no more irritated now than I ever am. | 1, I get annoyed or irritated more easily than I used to. | 2, I feel irritated all the time now. | 3, I don't get irritated at all by the things that used to irritate me.
**bdi_people_12**	day_2_part_2		radio		0, I have not lost interest in other people. | 1, I am less interested in other people than I used to be. | 2, I have lost most of my interest in other people. | 3, I have lost all of my interest in other people.
**bdi_decis_13**	day_2_part_2		radio		0, I make decisions about as well as I ever could. | 1, I put off making decisions more than I used to. | 2, I have greater difficulty in making decisions than before. | 3, I can't make decisions at all anymore.
**bdi_ugly_14**	day_2_part_2		radio		0, I don't feel I look any worse than I used to. | 1, I am worried that I am looking old or unattractive. | 2, I feel that there are permanent changes in my appearance that make me look unattractive. | 3, I believe that I look ugly.

**bdi_work_15**	day_2_part_2		radio		0, I can work about as well as usual. | 1, It takes an extra effort to get started at doing something. | 2, I have to push myself very hard to do anything. | 3, I can't do any work at all.
**bdi_sleep_16**	day_2_part_2		radio		0, I can sleep as well as before. | 1, I don't sleep as well as I used to. | 2, I wake up 1-2 hours earlier than usual and find it hard to get back to sleep. | 3, I wake up several hours earlier than I used to and cannot get back to sleep.
**bdi_tired_17**	day_2_part_2		radio		0, I don't get more tired than usual. | 1, I get tired more easily than I used to. | 2, I get tired from doing almost anything. | 3, I am too tired to do anything.
**bdi_eat_18**	day_2_part_2		radio		0, My appetite is no worse than usual. | 1, My appetite is not as good as it used to be. | 2, My appetite is much worse now. | 3, I have no appetite at all anymore.
**bdi_weight_19**	day_2_part_2		radio		0, I haven't lost much weight, if any lately. | 1, I have lost more than 5 pounds. | 2, I have lost more than 10 pounds. | 3, I have lost more than 15 pounds.
**bdi_diet_20**	day_2_part_2		radio	I am purposely trying to lose weight by eating less.	0, No | 1, Yes
**bdi_health_21**	day_2_part_2		radio		0, I am no more worried about my health than usual. | 1, I am worried about physical problems such as aches, pains, upset stomach, or constipation. | 2, I am very worried about physical problems and it's hard to think of much else. | 3, I am so worried

					about my physical problems that I cannot think about anything else.
**bdi_sex_22**	day_2_part_2		radio		0, I have not noticed any recent change in my interest in sex. | 1, I am less interested in sex than I used to be. | 2, I am much less interested in sex now. | 3, I have lost interest in sex completely.
**mbi_1**	day_2_part_2	Work Fatigue:<br>Instructions: Please mark your answers to the items on the answer sheet.	radio	I feel emotionally drained from my work.	0, Never | 1, Sporadically: A few times a year or less | 2, Monthly: Once a month or less | 3, Regularly: A few times per month | 4, Often: Once a week | 5, A few times a week | 6, Daily
**mbi_2**	day_2_part_2		radio	I feel used up at the end of the workday.	0, Never | 1, Sporadically: A few times a year or less | 2, Monthly: Once a month or less | 3, Regularly: A few times per month | 4, Often: Once a week | 5, A few times a week | 6, Daily
**mbi_3**	day_2_part_2		radio	I feel tired when I get up in the morning and have to face another day on the job.	0, Never | 1, Sporadically: A few times a year or less | 2, Monthly: Once a month or less | 3, Regularly: A few times per month | 4, Often: Once a week | 5, A few times a week | 6, Daily
**mbi_4**	day_2_part_2		radio	Working all day is really a strain for me.	0, Never | 1, Sporadically: A few times a year or less | 2, Monthly: Once a month or less | 3, Regularly: A few times per month | 4, Often: Once a week | 5, A few times a week | 6, Daily
**mbi_5**	day_2_part_2		radio	I can effectively solve the problems that arise in my work.	0, Never | 1, Sporadically: A few times a year or less | 2, Monthly: Once a month or less | 3, Regularly: A few times per month | 4, Often: Once a week | 5, A few times a week | 6, Daily

**mbi_6**	day_2_part_2		radio	I feel frustrated by my job.	0, Never | 1, Sporadically: A few times a year or less | 2, Monthly: Once a month or less | 3, Regularly: A few times per month | 4, Often: Once a week | 5, A few times a week | 6, Daily
**mbi_7**	day_2_part_2		radio	I feel burned out from my work.	0, Never | 1, Sporadically: A few times a year or less | 2, Monthly: Once a month or less | 3, Regularly: A few times per month | 4, Often: Once a week | 5, A few times a week | 6, Daily
**mbi_8**	day_2_part_2		radio	I feel I'm making an effective contribution to what this organization does.	0, Never | 1, Sporadically: A few times a year or less | 2, Monthly: Once a month or less | 3, Regularly: A few times per month | 4, Often: Once a week | 5, A few times a week | 6, Daily
**mbi_9**	day_2_part_2		radio	I have become less interested in my work since I started this job.	0, Never | 1, Sporadically: A few times a year or less | 2, Monthly: Once a month or less | 3, Regularly: A few times per month | 4, Often: Once a week | 5, A few times a week | 6, Daily
**mbi_10**	day_2_part_2		radio	I have become less enthusiastic about my work.	0, Never | 1, Sporadically: A few times a year or less | 2, Monthly: Once a month or less | 3, Regularly: A few times per month | 4, Often: Once a week | 5, A few times a week | 6, Daily
**mbi_11**	day_2_part_2		radio	In my opinion, I am good at my job.	0, Never | 1, Sporadically: A few times a year or less | 2, Monthly: Once a month or less | 3, Regularly: A few times per month | 4, Often: Once a week | 5, A few times a week | 6, Daily
**mbi_12**	day_2_part_2		radio	I feel exhilarated when I accomplish something at work.	0, Never | 1, Sporadically: A few times a year or less | 2, Monthly: Once a month or less | 3, Regularly: A few times per

					month | 4, Often: Once a week | 5, A few times a week | 6, Daily
**mbi_13**	day_2_part_2		radio	I just want to do my job and not be bothered.	0, Never | 1, Sporadically: A few times a year or less | 2, Monthly: Once a month or less | 3, Regularly: A few times per month | 4, Often: Once a week | 5, A few times a week | 6, Daily
**mbi_14**	day_2_part_2		radio	I doubt the significance of my work.	0, Never | 1, Sporadically: A few times a year or less | 2, Monthly: Once a month or less | 3, Regularly: A few times per month | 4, Often: Once a week | 5, A few times a week | 6, Daily
**mbi_15**	day_2_part_2		radio	I have become more cynical about whether my work contributes anything.	0, Never | 1, Sporadically: A few times a year or less | 2, Monthly: Once a month or less | 3, Regularly: A few times per month | 4, Often: Once a week | 5, A few times a week | 6, Daily
**mbi_16**	day_2_part_2		radio	At my work, I feel confident that I am effective at getting things done.	0, Never | 1, Sporadically: A few times a year or less | 2, Monthly: Once a month or less | 3, Regularly: A few times per month | 4, Often: Once a week | 5, A few times a week | 6, Daily
**pcl_1**	day_2_part_2	Recurring Thoughts<br>Instructions: Below is a list of problems and complaints that people sometimes have in response to stressful experiences. Please read each one carefully and choose the answer that indicates how much you have been bothered by that problem in the last month.	radio	Having repeated, disturbing memories, thoughts, or images of a stressful experience from the past?	1, Not at all | 2, A little bit | 3, Moderately | 4, Quite a bit | 5, Extremely
**pcl_2**	day_2_part_2		radio	Having repeated, disturbing dreams of a stressful experience from the past?	1, Not at all | 2, A little bit | 3, Moderately | 4, Quite a bit | 5, Extremely

**pcl_3**	day_2_part_2		radio	Suddenly acting or feeling as if a stressful experience were happening again (as if you were reliving it)?	1, Not at all | 2, A little bit | 3, Moderately | 4, Quite a bit | 5, Extremely
**pcl_4**	day_2_part_2		radio	Feeling very upset when something reminded you of a stressful experience from the past?	1, Not at all | 2, A little bit | 3, Moderately | 4, Quite a bit | 5, Extremely
**pcl_5**	day_2_part_2		radio	Having physical reactions (e.g., heart pounding, trouble breathing, sweating) when something reminded you of a stressful experience from the past?	1, Not at all | 2, A little bit | 3, Moderately | 4, Quite a bit | 5, Extremely
**pcl_6**	day_2_part_2		radio	Avoiding thinking about or talking about a stressful experience from the past or avoiding having feelings related to it?	1, Not at all | 2, A little bit | 3, Moderately | 4, Quite a bit | 5, Extremely
**pcl_7**	day_2_part_2		radio	Avoiding activities or situations because they reminded you of a stressful experience from the past?	1, Not at all | 2, A little bit | 3, Moderately | 4, Quite a bit | 5, Extremely
**pcl_8**	day_2_part_2		radio	Trouble remembering important parts of a stressful experience from the past?	1, Not at all | 2, A little bit | 3, Moderately | 4, Quite a bit | 5, Extremely
**pcl_9**	day_2_part_2		radio	Loss of interest in activities that you used to enjoy?	1, Not at all | 2, A little bit | 3, Moderately | 4, Quite a bit | 5, Extremely
**pcl_10**	day_2_part_2		radio	Feeling distant or cut off from other people?	1, Not at all | 2, A little bit | 3, Moderately | 4, Quite a bit | 5, Extremely

**pcl_11**	day_2_part_2		radio	Feeling emotionally numb or being unable to have loving feelings for those close to you?	1, Not at all | 2, A little bit | 3, Moderately | 4, Quite a bit | 5, Extremely
**pcl_12**	day_2_part_2		radio	Feeling as if your future somehow will be cut short?	1, Not at all | 2, A little bit | 3, Moderately | 4, Quite a bit | 5, Extremely
**pcl_13**	day_2_part_2		radio	Trouble falling or staying asleep?	1, Not at all | 2, A little bit | 3, Moderately | 4, Quite a bit | 5, Extremely
**pcl_14**	day_2_part_2		radio	Feeling irritable or having angry outbursts?	1, Not at all | 2, A little bit | 3, Moderately | 4, Quite a bit | 5, Extremely
**pcl_15**	day_2_part_2		radio	Having difficulty concentrating?	1, Not at all | 2, A little bit | 3, Moderately | 4, Quite a bit | 5, Extremely
**pcl_16**	day_2_part_2		radio	Being 'super-alert,' watchful, or on guard?	1, Not at all | 2, A little bit | 3, Moderately | 4, Quite a bit | 5, Extremely
**pcl_17**	day_2_part_2		radio	Feeling jumpy or easily startled?	1, Not at all | 2, A little bit | 3, Moderately | 4, Quite a bit | 5, Extremely

2.3.3 Part 3 of Questionnaire Package

Redcap Form Name: Day 2 Part 3

Questionnaire Name	Title	Variable Name
2.3.3.1 Mood Symptoms	DSM-5 Self-Rated Level 1 Cross-Cutting Symptom Measure—Adult	Dsm5
2.3.3.2 Childhood Trauma	Childhood Trauma Questionnaire (CTQ)	ctq

Variable / Field Name	Form Name	Section Header	Field Type	Field Label	Choices, Calculations, OR Slider Labels

**dsm5_lv1_1**	day_2_part_3	Instructions: The questions below ask about things that might have bothered you. For each question, circle the number that best describes how much (or how often) you have been bothered by each problem during the past TWO (2) WEEKS.<br>During the past TWO (2) WEEKS, how much (or how often) have you been bothered by the following problems?	radio	1. Little interest or pleasure in doing things?	0, None / Not at all | 1, Slight / Rare, less than a day or two | 2, Mild / Several days | 3, Moderate / More than half the days | 4, Severe / Nearly every day
**dsm5_lv1_2**	day_2_part_3		radio	2. Feeling down, depressed, or hopeless?	0, None / Not at all | 1, Slight / Rare, less than a day or two | 2, Mild / Several days | 3, Moderate / More than half the days | 4, Severe / Nearly every day
**dsm5_lv1_3**	day_2_part_3		radio	3. Feeling more irritated, grouchy, or angry than usual?	0, None / Not at all | 1, Slight / Rare, less than a day or two | 2, Mild / Several days | 3, Moderate / More than half the days | 4, Severe / Nearly every day
**dsm5_lv1_4**	day_2_part_3		radio	4. Sleeping less than usual, but still have a lot of energy?	0, None / Not at all | 1, Slight / Rare, less than a day or two | 2, Mild / Several days | 3, Moderate / More than half the days | 4, Severe / Nearly every day
**dsm5_lv1_5**	day_2_part_3		radio	5. Starting lots more projects than usual or doing more risky things than usual?	0, None / Not at all | 1, Slight / Rare, less than a day or two | 2, Mild / Several days | 3, Moderate / More than half the days | 4, Severe / Nearly every day

**dsm5_lv1_6**	day_2_part_3		radio	6. Feeling nervous, anxious, frightened, worried, or on edge?	0, None / Not at all | 1, Slight / Rare, less than a day or two | 2, Mild / Several days | 3, Moderate / More than half the days | 4, Severe / Nearly every day
**dsm5_lv1_7**	day_2_part_3		radio	7. Feeling panic or being frightened?	0, None / Not at all | 1, Slight / Rare, less than a day or two | 2, Mild / Several days | 3, Moderate / More than half the days | 4, Severe / Nearly every day
**dsm5_lv1_8**	day_2_part_3		radio	8. Avoiding situations that make you anxious?	0, None / Not at all | 1, Slight / Rare, less than a day or two | 2, Mild / Several days | 3, Moderate / More than half the days | 4, Severe / Nearly every day
**dsm5_lv1_9**	day_2_part_3		radio	9. Unexplained aches and pains (e.g., head, back, joints, abdomen, legs)?	0, None / Not at all | 1, Slight / Rare, less than a day or two | 2, Mild / Several days | 3, Moderate / More than half the days | 4, Severe / Nearly every day
**dsm5_lv1_10**	day_2_part_3		radio	10. Feeling that your illnesses are not being taken seriously enough?	0, None / Not at all | 1, Slight / Rare, less than a day or two | 2, Mild / Several days | 3, Moderate / More than half the days | 4, Severe / Nearly every day

**dsm5_lv1_11**	day_2_part_3		radio	11. Thoughts of actually hurting yourself?	0, None / Not at all | 1, Slight / Rare, less than a day or two | 2, Mild / Several days | 3, Moderate / More than half the days | 4, Severe / Nearly every day
**dsm5_lv1_12**	day_2_part_3		radio	12. Hearing things other people couldn't hear, such as voices even when no one was around?	0, None / Not at all | 1, Slight / Rare, less than a day or two | 2, Mild / Several days | 3, Moderate / More than half the days | 4, Severe / Nearly every day
**dsm5_lv1_13**	day_2_part_3		radio	13. Feeling that someone could hear your thoughts, or that you could hear what another person was thinking?	0, None / Not at all | 1, Slight / Rare, less than a day or two | 2, Mild / Several days | 3, Moderate / More than half the days | 4, Severe / Nearly every day
**dsm5_lv1_14**	day_2_part_3		radio	14. Problems with sleep that affected your sleep quality over all?	0, None / Not at all | 1, Slight / Rare, less than a day or two | 2, Mild / Several days | 3, Moderate / More than half the days | 4, Severe / Nearly every day
**dsm5_lv1_15**	day_2_part_3		radio	15. Problems with memory (e.g., learning new information) or with location (e.g., finding your way home)?	0, None / Not at all | 1, Slight / Rare, less than a day or two | 2, Mild / Several days | 3, Moderate / More than half the days | 4, Severe / Nearly every day

**dsm5_lv1_16**	day_2_part_3		radio	16. Unpleasant thoughts, urges, or images that repeatedly enter your mind?	0, None / Not at all | 1, Slight / Rare, less than a day or two | 2, Mild / Several days | 3, Moderate / More than half the days | 4, Severe / Nearly every day
**dsm5_lv1_17**	day_2_part_3		radio	17. Feeling driven to perform certain behaviors or mental acts over and over again?	0, None / Not at all | 1, Slight / Rare, less than a day or two | 2, Mild / Several days | 3, Moderate / More than half the days | 4, Severe / Nearly every day
**dsm5_lv1_18**	day_2_part_3		radio	18. Feeling detached or distant from yourself, your body, your physical surroundings, or your memories?	0, None / Not at all | 1, Slight / Rare, less than a day or two | 2, Mild / Several days | 3, Moderate / More than half the days | 4, Severe / Nearly every day
**dsm5_lv1_19**	day_2_part_3		radio	19. Not knowing who you really are or what you want out of life?	0, None / Not at all | 1, Slight / Rare, less than a day or two | 2, Mild / Several days | 3, Moderate / More than half the days | 4, Severe / Nearly every day
**dsm5_lv1_20**	day_2_part_3		radio	20. Not feeling close to other people or enjoying your relationships with them?	0, None / Not at all | 1, Slight / Rare, less than a day or two | 2, Mild / Several days | 3, Moderate / More than half the days | 4, Severe / Nearly every day

**dsm5_lv1_21**	day_2_part_3		radio	21. Drinking at least 4 drinks of any kind of alcohol in a single day?	0, None / Not at all | 1, Slight / Rare, less than a day or two | 2, Mild / Several days | 3, Moderate / More than half the days | 4, Severe / Nearly every day
**dsm5_lv1_22**	day_2_part_3		radio	22. Smoking any cigarettes, a cigar, or pipe, or using snuff or chewing tobacco?	0, None / Not at all | 1, Slight / Rare, less than a day or two | 2, Mild / Several days | 3, Moderate / More than half the days | 4, Severe / Nearly every day
**dsm5_lv1_23**	day_2_part_3		radio	23. Using any of the following medicines ON YOUR OWN, that is, without a doctor's prescription, in greater amounts or longer than prescribed [e.g., painkillers (like Vicodin), stimulants (like Ritalin or Adderall), sedatives or tranquilizers (like sleeping pills or Valium), or drugs like marijuana, cocaine or crack, club drugs (like ecstasy), hallucinogens (like LSD), heroin, inhalants or solvents (like glue), or methamphetamine (like speed)]?	0, None / Not at all | 1, Slight / Rare, less than a day or two | 2, Mild / Several days | 3, Moderate / More than half the days | 4, Severe / Nearly every day

**dsm5_pibr_1**	day_2_part_3	Instructions: This is a list of things different people might say about themselves. We are interested in how you would describe yourself. There are no right or wrong answers. So you can describe yourself as honestly as possible, we will keep your responses confidential. We'd like you to take your time and read each statement carefully, selecting the response that best describes you.	radio	1. People would describe me as reckless.	0,Very False or Often False | 1,Sometimes or Somewhat False | 2,Sometimes or Somewhat True | 3,Very True or Often True
**dsm5_pibr_2**	day_2_part_3		radio	2. I feel like I act totally on impulse.	0,Very False or Often False | 1,Sometimes or Somewhat False | 2,Sometimes or Somewhat True | 3,Very True or Often True
**dsm5_pibr_3**	day_2_part_3		radio	3. Even though I know better, I can't stop making rash decisions.	0,Very False or Often False | 1,Sometimes or Somewhat False | 2,Sometimes or Somewhat True | 3,Very True or Often True
**dsm5_pibr_4**	day_2_part_3		radio	4. I often feel like nothing I do really matters.	0,Very False or Often False | 1,Sometimes or Somewhat False | 2,Sometimes or Somewhat True | 3,Very True or Often True
**dsm5_pibr_5**	day_2_part_3		radio	5. Others see me as irresponsible.	0,Very False or Often False | 1,Sometimes or Somewhat False | 2,Sometimes or Somewhat True | 3,Very True or Often True
**dsm5_pibr_6**	day_2_part_3		radio	6. I'm not good at planning ahead.	0,Very False or Often False | 1,Sometimes or Somewhat False |

					2,Sometimes or Somewhat True | 3,Very True or Often True
**dsm5_pibr_7**	day_2_part_3		radio	7. My thoughts often don't make sense to others.	0,Very False or Often False | 1,Sometimes or Somewhat False | 2,Sometimes or Somewhat True | 3,Very True or Often True
**dsm5_pibr_8**	day_2_part_3		radio	8. I worry about almost everything.	0,Very False or Often False | 1,Sometimes or Somewhat False | 2,Sometimes or Somewhat True | 3,Very True or Often True
**dsm5_pibr_9**	day_2_part_3		radio	9. I get emotional easily, often for very little reason.	0,Very False or Often False | 1,Sometimes or Somewhat False | 2,Sometimes or Somewhat True | 3,Very True or Often True
**dsm5_pibr_10**	day_2_part_3		radio	10. I fear being alone in life more than anything else.	0,Very False or Often False | 1,Sometimes or Somewhat False | 2,Sometimes or Somewhat True | 3,Very True or Often True
**dsm5_pibr_11**	day_2_part_3		radio	11. I get stuck on one way of doing things, even when it's clear it won't work.	0,Very False or Often False | 1,Sometimes or Somewhat False | 2,Sometimes or Somewhat True | 3,Very True or Often True

**dsm5_pibr_12**	day_2_part_3		radio	12. I have seen things that weren't really there.	0,Very False or Often False | 1,Sometimes or Somewhat False | 2,Sometimes or Somewhat True | 3,Very True or Often True
**dsm5_pibr_13**	day_2_part_3		radio	13. I steer clear of romantic relationships.	0,Very False or Often False | 1,Sometimes or Somewhat False | 2,Sometimes or Somewhat True | 3,Very True or Often True
**dsm5_pibr_14**	day_2_part_3		radio	14. I'm not interested in making friends.	0,Very False or Often False | 1,Sometimes or Somewhat False | 2,Sometimes or Somewhat True | 3,Very True or Often True
**dsm5_pibr_15**	day_2_part_3		radio	15. I get irritated easily by all sorts of things.	0,Very False or Often False | 1,Sometimes or Somewhat False | 2,Sometimes or Somewhat True | 3,Very True or Often True
**dsm5_pibr_16**	day_2_part_3		radio	16. I don't like to get too close to people.	0,Very False or Often False | 1,Sometimes or Somewhat False | 2,Sometimes or Somewhat True | 3,Very True or Often True
**dsm5_pibr_17**	day_2_part_3		radio	17. It's no big deal if I hurt other peoples' feelings.	0,Very False or Often False | 1,Sometimes or Somewhat False | 2,Sometimes or Somewhat True | 3,Very True or Often True

**dsm5_pibr_18**	day_2_part_3		radio	18. I rarely get enthusiastic about anything.	0,Very False or Often False | 1,Sometimes or Somewhat False | 2,Sometimes or Somewhat True | 3,Very True or Often True
**dsm5_pibr_19**	day_2_part_3		radio	19. I crave attention.	0,Very False or Often False | 1,Sometimes or Somewhat False | 2,Sometimes or Somewhat True | 3,Very True or Often True
**dsm5_pibr_20**	day_2_part_3		radio	20. I often have to deal with people who are less important than me.	0,Very False or Often False | 1,Sometimes or Somewhat False | 2,Sometimes or Somewhat True | 3,Very True or Often True
**dsm5_pibr_21**	day_2_part_3		radio	21. I often have thoughts that make sense to me but that other people say are strange.	0,Very False or Often False | 1,Sometimes or Somewhat False | 2,Sometimes or Somewhat True | 3,Very True or Often True
**dsm5_pibr_22**	day_2_part_3		radio	22. I use people to get what I want.	0,Very False or Often False | 1,Sometimes or Somewhat False | 2,Sometimes or Somewhat True | 3,Very True or Often True
**dsm5_pibr_23**	day_2_part_3		radio	23. I often"zone out" and then suddenly come to and realize that a lot of time has passed.	0,Very False or Often False | 1,Sometimes or Somewhat False | 2,Sometimes or Somewhat True | 3,Very True or Often True

**dsm5_pibr_24**	day_2_part_3		radio	24. Things around me often feel unreal, or more real than usual.	0,Very False or Often False | 1,Sometimes or Somewhat False | 2,Sometimes or Somewhat True | 3,Very True or Often True
**dsm5_pibr_25**	day_2_part_3		radio	25. It is easy for me to take advantage of others.	0,Very False or Often False | 1,Sometimes or Somewhat False | 2,Sometimes or Somewhat True | 3,Very True or Often True
**dsm5_lv2_sa_1**	day_2_part_3	Instructions: On the questionnaire that you just completed, you indicated that during the past 2 weeks you have been bothered by "using medicines on your own without a doctor's prescription, or in greater amounts or longer than prescribed, and/or using drugs like marijuana, cocaine or crack, and/or other drugs" at a slight or greater level of severity. The questions below ask how often you have used these medicines and/or substances during the past 2 weeks.<br><br>During the past TWO (2) WEEKS, about how often did you use any of the following medicines ON YOUR OWN, that is, without a doctor's prescription, in greater amounts or longer than prescribed?	radio	a. Painkillers (like Vicodin)	0, Not at all | 1, One or two days | 2, Several days | 3, More than half the days | 4, Nearly every day
**dsm5_lv2_sa_2**	day_2_part_3		radio	b. Stimulants (like Ritalin, Adderall)	0, Not at all | 1, One or two days | 2, Several days | 3, More than half the days | 4, Nearly every day
**dsm5_lv2_sa_3**	day_2_part_3		radio	c. Sedatives or tranquilizers (like sleeping pills or Valium)	0, Not at all | 1, One or two days | 2, Several days | 3, More than half the days | 4, Nearly every day

**dsm5_lv2_sa_4**	day_2_part_3		radio	d. Marijuana	0, Not at all | 1, One or two days | 2, Several days | 3, More than half the days | 4, Nearly every day
**dsm5_lv2_sa_5**	day_2_part_3		radio	e. Cocaine or crack	0, Not at all | 1, One or two days | 2, Several days | 3, More than half the days | 4, Nearly every day
**dsm5_lv2_sa_6**	day_2_part_3		radio	f. Club drugs (like ecstasy)	0, Not at all | 1, One or two days | 2, Several days | 3, More than half the days | 4, Nearly every day
**dsm5_lv2_sa_7**	day_2_part_3		radio	g. Hallucinogens (like LSD)	0, Not at all | 1, One or two days | 2, Several days | 3, More than half the days | 4, Nearly every day
**dsm5_lv2_sa_8**	day_2_part_3		radio	h. Heroin	0, Not at all | 1, One or two days | 2, Several days | 3, More than half the days | 4, Nearly every day
**dsm5_lv2_sa_9**	day_2_part_3		radio	i. Inhalants or solvents (like glue)	0, Not at all | 1, One or two days | 2, Several days | 3, More than half the days | 4, Nearly every day
**dsm5_lv2_sa_10**	day_2_part_3		radio	j. Methamphetamine (like speed)	0, Not at all | 1, One or two days | 2, Several days | 3, More than half the days | 4, Nearly every day
**dsm5_lv2_ss_1**	day_2_part_3	Instructions: On the questionnaire that you just completed, you indicated that during the past 2 weeks you have been bothered by "unexplained aches and pains", and/or "feeling that your illnesses are not being taken seriously enough"	radio	1. Stomach pain	0, Not bothered at all (0) | 1, Bothered a little (1) | 2, Bothered a lot (2)

		at a mild or greater level of severity. The questions below ask about these feelings in more detail and especially how often you have been bothered by a list of symptoms during the past 7 days.<br>During the past 7 days, how much have you been bothered by any of the following problems?			
**dsm5_lv2_ss_2**	day_2_part_3		radio	2. Back pain	0, Not bothered at all (0) | 1, Bothered a little (1) | 2, Bothered a lot (2)
**dsm5_lv2_ss_3**	day_2_part_3		radio	3. Pain in your arms, legs, or joints (knees, hips, etc.)	0, Not bothered at all (0) | 1, Bothered a little (1) | 2, Bothered a lot (2)
**dsm5_lv2_ss_4**	day_2_part_3		radio	4. Menstrual cramps or other problems with your periods WOMEN ONLY	0, Not bothered at all (0) | 1, Bothered a little (1) | 2, Bothered a lot (2)
**dsm5_lv2_ss_5**	day_2_part_3		radio	5. Headaches	0, Not bothered at all (0) | 1, Bothered a little (1) | 2, Bothered a lot (2)
**dsm5_lv2_ss_6**	day_2_part_3		radio	6. Chest pain	0,Not bothered at all (0) | 1,Bothered a little (1) | 2,Bothered a lot (2)
**dsm5_lv2_ss_7**	day_2_part_3		radio	7. Dizziness	0,Not bothered at all (0) | 1,Bothered a little (1) | 2,Bothered a lot (2)
**dsm5_lv2_ss_8**	day_2_part_3		radio	8. Fainting spells	0,Not bothered at all (0) | 1,Bothered a little (1) | 2,Bothered a lot (2)

**dsm5_lv2_ss_9**	day_2_part_3		radio	9. Feeling your heart pound or race	0,Not bothered at all (0) | 1,Bothered a little (1) | 2,Bothered a lot (2)
**dsm5_lv2_ss_10**	day_2_part_3		radio	10. Shortness of breath	0,Not bothered at all (0) | 1,Bothered a little (1) | 2,Bothered a lot (2)
**dsm5_lv2_ss_11**	day_2_part_3		radio	11. Pain or problems during sexual intercourse	0,Not bothered at all (0) | 1,Bothered a little (1) | 2,Bothered a lot (2)
**dsm5_lv2_ss_12**	day_2_part_3		radio	12. Constipation, loose bowels, or diarrhea	0,Not bothered at all (0) | 1,Bothered a little (1) | 2,Bothered a lot (2)
**dsm5_lv2_ss_13**	day_2_part_3		radio	13. Nausea, gas, or indigestion	0,Not bothered at all (0) | 1,Bothered a little (1) | 2,Bothered a lot (2)
**dsm5_lv2_ss_14**	day_2_part_3		radio	14. Feeling tired or having low energy	0,Not bothered at all (0) | 1,Bothered a little (1) | 2,Bothered a lot (2)
**dsm5_lv2_ss_15**	day_2_part_3		radio	15. Trouble sleeping	0,Not bothered at all (0) | 1,Bothered a little (1) | 2,Bothered a lot (2)
**dsm5_lv2_sd_1**	day_2_part_3	Instructions to patient: On the questionnaire that you just completed, you indicated that during the past 2 weeks you have been bothered by "problems with sleep that affected your sleep quality over all" at a mild or greater level of severity. The questions below ask about these feelings in more detail and especially how often you have been bothered by a list of symptoms during the past 7 days.<br>In the past SEVEN (7) DAYS....	radio	1. My sleep was restless.	1, Not at all | 2, A little bit | 3, Somewhat | 4, Quite a bit | 5, Very much

**dsm5_lv2_sd_2**	day_2_part_3		radio	2. I was satisfied with my sleep.	5,Not at all | 4, A little bit | 3, Somewhat| 2, Quite a bit | 1,Very much
**dsm5_lv2_sd_3**	day_2_part_3		radio	3. My sleep was refreshing.	5,Not at all | 4, A little bit | 3, Somewhat| 2, Quite a bit | 1,Very much
**dsm5_lv2_sd_4**	day_2_part_3		radio	4. I had difficulty falling asleep.	1,Not at all | 2, A little bit | 3, Somewhat| 4, Quite a bit | 5,Very much
**dsm5_lv2_sd_5**	day_2_part_3	In the past SEVEN (7) DAYS....	radio	5. I had trouble staying asleep.	1,Never | 2,Rarely | 3,Sometimes | 4,Often | 5,Always
**dsm5_lv2_sd_6**	day_2_part_3		radio	6. I had trouble sleeping.	1,Never | 2,Rarely | 3,Sometimes | 4,Often | 5,Always
**dsm5_lv2_sd_7**	day_2_part_3		radio	7. I got enough sleep.	5,Never | 4,Rarely | 3,Sometimes | 2,Often | 1,Always
**dsm5_lv2_sd_8**	day_2_part_3	In the past SEVEN (7) DAYS....	radio	8. My sleep quality was...	5,Very Poor | 4,Poor | 3,Fair | 2,Good | 1,Very good
**dsm5_lv2_rtb_1**	day_2_part_3	Instructions: On the questionnaire that you just completed, you indicated that during the past 2 weeks you have been bothered by "unwanted repeated thoughts, images, or urges" and/or "being driven to perform certain behaviors or mental acts over and over" at a mild or greater level of severity. The questions below ask about these feelings in more detail and especially how often you have been bothered by a list of symptoms during the past 7 days.<br>During the past SEVEN (7) DAYS....	radio	1. On average, how much time is occupied by these thoughts or behaviors each day?	0, 0-None | 1, 1-Mild (Less than an hour a day) | 2, 2-Moderate (1 to 3 hours a day) | 3, 3-Severe (3 to 8 hours a day) | 4, 4-Extreme (more than 8 hours a day)

**dsm5_lv2_rtb_2**	day_2_part_3		radio	2. How much distress do these thoughts or behaviors cause you?	0,0-None | 1,1-Mild (slightly disturbing) | 2,2-Moderate (disturbing but still manageable) | 3,3-Severe (very disturbing) | 4,4-Extreme (overwhelming distress)
**dsm5_lv2_rtb_3**	day_2_part_3		radio	3. How hard is it for you to control these thoughts or behaviors?	0,0-Complete control | 1,1-Much control (usually able to control thoughts or behaviors) | 2,2-Moderate control (sometimes able to control thoughts or behaviors) | 3,3-Little control (infrequently able to control thoughts or behaviors) | 4,4-No control (unable to control thoughts or behaviors)
**dsm5_lv2_rtb_4**	day_2_part_3		radio	4. How much do these thoughts or behaviors cause you to avoid doing anything, going anyplace, or being with anyone?	0,0-No avoidance | 1,1-Mild (occasional avoidance) | 2,2-Moderate (regularly avoid doing these things) | 3,3-Severe (frequent and extensive avoidance) | 4,4 - Extreme (nearly complete avoidance; house- bound)
**dsm5_lv2_rtb_5**	day_2_part_3		radio	5. How much do these thoughts or behaviors interfere with school, work, or your social or family life?	0,0-None | 1,1-Mild (slight interference) | 2,2-Moderate; (definite interference with functioning, but still manageable) | 3,3-Severe (substantial interference) | 4,4-Extreme (near-total interference; incapacitated)

**dsm5_lv2_mania_1**	day_2_part_3	Instructions: On the questionnaire you just completed, you indicated that during the past 2 weeks you have been bothered by "sleeping less than usual, but still having a lot of energy" and/or "starting lots more projects than usual or doing more risky things than usual" at a mild or greater level of severity. The five statement groups or questions below ask about these feelings in more detail.<br>1. Please read each group of statements/question carefully.<br>2. Choose the one statement in each group that best describes the way you have been feeling for the past week.<br>3. Check the box next to the number/statement selected.<br>4. Please note: The word "occasionally" when used here means once or twice; "often" means several times or more and "frequently" means most of the time.	radio	Question 1	1,1 I do not feel happier or more cheerful than usual. | 2,2 I occasionally feel happier or more cheerful than usual. | 3,3 I often feel happier or more cheerful than usual. | 4,4 I feel happier or more cheerful than usual most of the time. | 5, 5 I feel happier of more cheerful than usual all of the time.
**dsm5_lv2_mania_2**	day_2_part_3		radio	Question 2	1, 1 I do not feel more self-confident than usual. | 2,2 I occasionally feel more self-confident than usual. | 3, 3 I often feel more self-confident than usual. | 4, 4 I frequently feel more self-confident than usual. | 5, 5 I feel extremely self-confident all of the time.
**dsm5_lv2_mania_3**	day_2_part_3		radio	Question 3	1,1 I do not need less sleep than usual. | 2, 2 I occasionally need less sleep than usual. | 3,3 I often need less sleep than usual. | 4,4 I frequently need less sleep than usual. | 5, 5 I can go all

					day and all night without any sleep and still not feel tired.
**dsm5_lv2_mania_4**	day_2_part_3		radio	Question 4	1,1 I do not talk more than usual. | 2,2 I occasionally talk more than usual. | 3, 3 I often talk more than usual. | 4, 4 I frequently talk more than usual. | 5,5 I talk constantly and cannot be interrupted.
**dsm5_lv2_mania_5**	day_2_part_3		radio	Question 5	1,1 I have not been more active (either socially, sexually, at work, home, or school) than usual. | 2,2 I have occasionally been more active than usual. | 3,3 I have often been more active than usual. | 4, 4 I have frequently been more active than usual. | 5,5 I am constantly more active or on the go all the time.
**dsm5_lv2_depr_1**	day_2_part_3	Instructions: On the questionnaire that you just completed, you indicated that during the past 2 weeks you have been bothered by "no interest or pleasure in doing things" and/or "feeling down, depressed, or hopeless" at a mild or greater level of severity. The questions below ask about these feelings in more detail and especially how often you have been bothered by a list of symptoms during the past 7 days. Please respond to each item by marking one box per row.<br>In the past SEVEN (7) DAYS....	radio	1. I felt worthless.	1, Never | 2, Rarely | 3, Sometimes | 4, Often | 5, Always

**dsm5_lv2_depr_2**	day_2_part_3		radio	2. I felt that I had nothing to look forward to.	1, Never | 2, Rarely | 3, Sometimes | 4, Often | 5, Always
**dsm5_lv2_depr_3**	day_2_part_3		radio	3. I felt helpless.	1, Never | 2, Rarely | 3, Sometimes | 4, Often | 5, Always
**dsm5_lv2_depr_4**	day_2_part_3		radio	4. I felt sad.	1, Never | 2, Rarely | 3, Sometimes | 4, Often | 5, Always
**dsm5_lv2_depr_5**	day_2_part_3		radio	5. I felt like a failure.	1, Never | 2, Rarely | 3, Sometimes | 4, Often | 5, Always
**dsm5_lv2_depr_6**	day_2_part_3		radio	6. I felt depressed.	1, Never | 2, Rarely | 3, Sometimes | 4, Often | 5, Always
**dsm5_lv2_depr_7**	day_2_part_3		radio	7. I felt unhappy.	1, Never | 2, Rarely | 3, Sometimes | 4, Often | 5, Always
**dsm5_lv2_depr_8**	day_2_part_3		radio	8. I felt hopeless.	1, Never | 2, Rarely | 3, Sometimes | 4, Often | 5, Always
**dsm5_lv2_anx_1**	day_2_part_3	Instructions to patient: On the questionnaire that you just completed, you indicated that during the past 2 weeks you have been bothered by "feeling nervous, anxious, frightened, worried, or on edge", "feeling panic or being frightened", and/or "avoiding situations that make you anxious" at a mild or greater level of severity. The questions below ask about these feelings in more detail and especially how often you have been bothered by a list of symptoms during the	radio	1. I felt fearful.	1, Never | 2, Rarely | 3, Sometimes | 4, Often | 5, Always

		past 7 days. Please respond to each item by marking one box<br>per row.<br>In the past SEVEN (7) DAYS....			
**dsm5_lv2_anx_2**	day_2_part_3		radio	2. I felt anxious.	1, Never | 2, Rarely | 3, Sometimes | 4, Often | 5, Always
**dsm5_lv2_anx_3**	day_2_part_3		radio	3. I felt worried.	1, Never | 2, Rarely | 3, Sometimes | 4, Often | 5, Always
**dsm5_lv2_anx_4**	day_2_part_3		radio	4. I found it hard to focus on anything other than my anxiety.	1, Never | 2, Rarely | 3, Sometimes | 4, Often | 5, Always
**dsm5_lv2_anx_5**	day_2_part_3		radio	5. I felt nervous.	1, Never | 2, Rarely | 3, Sometimes | 4, Often | 5, Always
**dsm5_lv2_anx_6**	day_2_part_3		radio	6. I felt uneasy.	1, Never | 2, Rarely | 3, Sometimes | 4, Often | 5, Always
**dsm5_lv2_anx_7**	day_2_part_3		radio	7. I felt tense.	1, Never | 2, Rarely | 3, Sometimes | 4, Often | 5, Always
**dsm5_lv2_angr_1**	day_2_part_3	Instructions: On the questionnaire that you just completed, you indicated that during the past 2 weeks you have been bothered by "feeling irritated, grouchy, or angry" at a mild or greater level of severity. The questions below ask about these feelings in more detail and especially how often you have been bothered by a list of symptoms during the past 7 days. Please	radio	1. I was irritated more than people knew.	1, Never | 2, Rarely | 3, Sometimes | 4, Often | 5, Always

		respond to each item by marking one box per row.<br>In the past SEVEN (7) DAYS....			
**dsm5_lv2_angr_2**	day_2_part_3		radio	2. I felt angry.	1, Never | 2, Rarely | 3, Sometimes | 4, Often | 5, Always
**dsm5_lv2_angr_3**	day_2_part_3		radio	3. I felt like I was ready to explode.	1, Never | 2, Rarely | 3, Sometimes | 4, Often | 5, Always
**dsm5_lv2_angr_4**	day_2_part_3		radio	4. I was grouchy.	1, Never | 2, Rarely | 3, Sometimes | 4, Often | 5, Always
**dsm5_lv2_angr_5**	day_2_part_3		radio	5. I felt annoyed.	1, Never | 2, Rarely | 3, Sometimes | 4, Often | 5, Always
**ctq_eat_1**	day_2_part_3	Early Experiences<br>Instructions: These questions ask about some of your experiences growing up as a child and a teenager. For each question, choose the answer that best describes how you feel. Although some of these questions are of a personal nature, please try to answer as honestly as you can. Your answers will be kept confidential.<br>When I was growing up...	radio	I didn't have enough to eat.	1, Never True | 2, Rarely True | 3, Sometimes True | 4, Often True | 5, Very Often True
**ctq_protect_1**	day_2_part_3		radio	I knew that there was someone to take care of me and protect me.	5, Never True | 4, Rarely True | 3, Sometimes True | 2, Often True | 1, Very Often True
**ctq_stupid_1**	day_2_part_3		radio	3. People in my family called me	1, Never True | 2, Rarely True | 3, Sometimes True

				things like "stupid," "lazy," or "ugly."	| 4, Often True | 5, Very Often True
**ctq_drunk_1**	day_2_part_3		radio	My parents were too drunk or high to take care of the family.	1, Never True | 2, Rarely True | 3, Sometimes True | 4, Often True | 5, Very Often True
**ctq_important_1**	day_2_part_3		radio	There was someone in my family who helped me feel that I was important or special.	5, Never True | 4, Rarely True | 3, Sometimes True | 2, Often True | 1, Very Often True
**ctq_dirty_1**	day_2_part_3		radio	I had to wear dirty clothes.	1, Never True | 2, Rarely True | 3, Sometimes True | 4, Often True | 5, Very Often True
**ctq_loved_1**	day_2_part_3		radio	I felt loved.	5, Never True | 4, Rarely True | 3, Sometimes True | 2, Often True | 1, Very Often True
**ctq_never_born_1**	day_2_part_3		radio	I thought my parents wished I had never been born.	1, Never True | 2, Rarely True | 3, Sometimes True | 4, Often True | 5, Very Often True
**ctq_hit_hospital_1**	day_2_part_3		radio	I got hit so hard by someone in my family that I had to see a doctor or go to the hospital.	1, Never True | 2, Rarely True | 3, Sometimes True | 4, Often True | 5, Very Often True
**ctq_no_change_1**	day_2_part_3		radio	There was nothing I wanted to change about my family.	0.000003, Never True | 0.00001, Rarely True | 0, Sometimes True | 0.00002, Often True | 1, Very Often True

**ctq_hit_bruises_1**	day_2_part_3		radio	People in my family hit me so hard that it left bruises or marks.	1, Never True | 2, Rarely True | 3, Sometimes True | 4, Often True | 5, Very Often True
**ctq_belt_1**	day_2_part_3		radio	I was punished with a belt, a board, a cord, or some other hard object.	1, Never True | 2, Rarely True | 3, Sometimes True | 4, Often True | 5, Very Often True
**ctq_looked_out_1**	day_2_part_3		radio	People in my family looked out for each other.	5, Never True | 4, Rarely True | 3, Sometimes True | 2, Often True | 1, Very Often True
**ctq_insulting_1**	day_2_part_3		radio	People in my family said hurtful or insulting things to me.	1, Never True | 2, Rarely True | 3, Sometimes True | 4, Often True | 5, Very Often True
**ctq_physically_abused_1**	day_2_part_3		radio	I believe I was physically abused.	1, Never True | 2, Rarely True | 3, Sometimes True | 4, Often True | 5, Very Often True
**ctq_perfect_1**	day_2_part_3		radio	I had the perfect childhood.	0.000003, Never True | 0.00001, Rarely True | 0, Sometimes True | 0.00002, Often True | 1, Very Often True
**ctq_hit_teacher_1**	day_2_part_3		radio	I got hit or beaten so badly that it was noticed by someone like a teacher, neighbor, or doctor.	1, Never True | 2, Rarely True | 3, Sometimes True | 4, Often True | 5, Very Often True
**ctq_hated_1**	day_2_part_3		radio	I felt that someone in my family hated me.	1, Never True | 2, Rarely True | 3, Sometimes True

					| 4, Often True | 5, Very Often True
**ctq_close_1**	day_2_part_3		radio	People in my family felt close to each other.	5, Never True | 4, Rarely True | 3, Sometimes True | 2, Often True | 1, Very Often True
**ctq_touch_sexual_1**	day_2_part_3		radio	Some tried to touch me in a sexual way, or tried to make me touch them.	1, Never True | 2, Rarely True | 3, Sometimes True | 4, Often True | 5, Very Often True
**ctq_threatened_sexual_1**	day_2_part_3		radio	Someone threatened to hurt me or tell lies about me unless I did something sexual with them.	1, Never True | 2, Rarely True | 3, Sometimes True | 4, Often True | 5, Very Often True
**ctq_best_1**	day_2_part_3		radio	I had the best family in the world.	0.000003, Never True | 0.00001, Rarely True | 0, Sometimes True | 0.00002, Often True | 1, Very Often True
**ctq_do_watch_sexual_1**	day_2_part_3		radio	Someone tried to make me do sexual things or watch sexual things.	1, Never True | 2, Rarely True | 3, Sometimes True | 4, Often True | 5, Very Often True
**ctq_molested_1**	day_2_part_3		radio	Someone molested me.	1, Never True | 2, Rarely True | 3, Sometimes True | 4, Often True | 5, Very Often True
**ctq_emontionally_abused_1**	day_2_part_3		radio	I believe I was emotionally abused.	1, Never True | 2, Rarely True | 3, Sometimes True | 4, Often True | 5, Very Often True

**ctq_doctor_1**	day_2_part_3		radio	There was someone to take me to the doctor if I needed it.	5, Never True | 4, Rarely True | 3, Sometimes True | 2, Often True | 1, Very Often True
**ctq_sexually_abused_1**	day_2_part_3		radio	I believe that I was sexually abused.	1, Never True | 2, Rarely True | 3, Sometimes True | 4, Often True | 5, Very Often True
**ctq_support_1**	day_2_part_3		radio	My family was a source or strength and support	5, Never True | 4, Rarely True | 3, Sometimes True | 2, Often True | 1, Very Often True

2.4 Questionnaire Scoring

2.4.1 Questionnaire Scoring

RedCAP Form Name: questionnaire_scoring

Description: Global and subscale questionnaire scoring.

Variable / Field Name	Section Header	Field Label	Choices, Calculations, OR Slider Labels
**mdes_emot_pos_d0**	Modified Differential Emotions Scale Day 0	mdes mean of positive valence items:	mean([day0_arm_2][mdes_amused_d0], [day0_arm_2][mdes_awe_d0], [day0_arm_2][mdes_control_d0], [day0_arm_2][mdes_excited_d0], [day0_arm_2][mdes_grateful_d0], [day0_arm_2][mdes_hopeful_d0], [day0_arm_2][mdes_inspired_d0], [day0_arm_2][mdes_interested_d0], [day0_arm_2][mdes_joyful_d0], [day0_arm_2][mdes_love_d0], [day0_arm_2][mdes_proud_d0], [day0_arm_2][mdes_satisfied_d0])

**mdes_emot_neg_d0**		mdes mean of negtive valence items:	mean([day0_arm_2][mdes_angry_d0], [day0_arm_2][mdes_ashamed_d0], [day0_arm_2][mdes_bored_d0], [day0_arm_2][mdes_contemptuous_d0], [day0_arm_2][mdes_disgust_d0], [day0_arm_2][mdes_embarrassed_d0], [day0_arm_2][mdes_guilty_d0], [day0_arm_2][mdes_hate_d0], [day0_arm_2][mdes_rejected_d0], [day0_arm_2][mdes_sad_d0], [day0_arm_2][mdes_scared_d0], [day0_arm_2][mdes_stressed_d0])
**mdes_emot_negbrief_d0**		mdes mean of selected negative valen ce items:	mean([day0_arm_2][mdes_angry_d0], [day0_arm_2][mdes_bored_d0], [day0_arm_2][mdes_embarrassed_d0], [day0_arm_2][mdes_guilty_d0], [day0_arm_2][mdes_sad_d0], [day0_arm_2][mdes_scared_d0], [day0_arm_2][mdes_stressed_d0])
**mdes_emot_tired_d0**		mdes tired:	[day0_arm_2][mdes_tired_d0]
**mdes_emot_pos_d1**	Day1	mdes mean of positive valence items:	mean([mdes_amused_d1], [mdes_awe_d1], [mdes_control_d1], [mdes_excited_d1], [mdes_grateful_d1], [mdes_hopeful_d1], [mdes_inspired_d1], [mdes_interested_d1], [mdes_joyful_d1], [mdes_love_d1], [mdes_proud_d1], [mdes_satisfied_d1])
**mdes_emot_neg_d1**		mdes mean of negtive valence items:	mean([mdes_angry_d1], [mdes_ashamed_d1], [mdes_bored_d1], [mdes_contemptuous_d1], [mdes_disgust_d1], [mdes_embarrassed_d1], [mdes_guilty_d1], [mdes_hate_d1], [mdes_rejected_d1], [mdes_sad_d1], [mdes_scared_d1], [mdes_stressed_d1])
**mdes_emot_negbrief_1**		mdes mean of selected negative valen ce items:	mean([mdes_angry_d1], [mdes_bored_d1], [mdes_embarrassed_d1], [mdes_guilty_d1], [mdes_sad_d1], [mdes_scared_d1], [mdes_stressed_d1])
**mdes_emot_tired_d1**		mdes tired:	[mdes_tired_d1]

**dise_nq_stress_negaffect_d0**	My Daily Stress Day 0	stressor-related negative affect	mean([day0_arm_2][dise_angry_d0], [day0_arm_2][dise_anxious_d0], [day0_arm_2][dise_sad_d0], [day0_arm_2][dise_shame_d0])
**dise_positive_apprs_d0**		positive_appraisa	mean([day0_arm_2][dise_control_d0], [day0_arm_2][dise_resources_d0], [day0_arm_2][dise_handlestress_d0])
**dise_stress_manage_d0**		stressor manageable	mean([day0_arm_2][dise_control_d0], [day0_arm_2][dise_resources_d0], (4-[day0_arm_2][dise_demanding_d0]), [day0_arm_2][dise_handlestress_d0])
**dise_nq_stress_negaffect_d1**	Day1	stressor-related negative affect	mean([dise_angry_d1], [dise_anxious_d1], [dise_sad_d1], [dise_shame_d1])
**dise_positive_apprs_d1**		positive_appraisa	mean([dise_control_d1], [dise_resources_d1], [dise_handlestress_d1])
**dise_stress_manage_d1**		stressor manageable	mean([dise_control_d1], [dise_resources_d1], (4-[dise_demanding_d1]), [dise_handlestress_d1])
**pwbs_total**		PWBS total	[day2_arm_2][pwbs_1]+ [day2_arm_2][pwbs_2]+ (7-[day2_arm_2][pwbs_3])+ [day2_arm_2][pwbs_4]+ (7-[day2_arm_2][pwbs_5])+ [day2_arm_2][pwbs_6]+ [day2_arm_2][pwbs_7]+ [day2_arm_2][pwbs_8]+ [day2_arm_2][pwbs_9]+ (7-[day2_arm_2][pwbs_10])+ [day2_arm_2][pwbs_11]+ [day2_arm_2][pwbs_12]+ (7-[day2_arm_2][pwbs_13])+ (7-[day2_arm_2][pwbs_14])+ (7-[day2_arm_2][pwbs_15])+ (7-[day2_arm_2][pwbs_16])+ (7-[day2_arm_2][pwbs_17])+ (7-[day2_arm_2][pwbs_18])+ (7-[day2_arm_2][pwbs_19])+ [day2_arm_2][pwbs_20]+ [day2_arm_2][pwbs_21]+ [day2_arm_2][pwbs_22]+ (7-[day2_arm_2][pwbs_23])+ [day2_arm_2][pwbs_24]+ [day2_arm_2][pwbs_25]+ (7-[day2_arm_2][pwbs_26])+ (7-[day2_arm_2][pwbs_27])+ [day2_arm_2][pwbs_28]+ [day2_arm_2][pwbs_29]+ (7-[day2_arm_2][pwbs_30])+ (7-[day2_arm_2][pwbs_31])+ (7-[day2_arm_2][pwbs_32])+ [day2_arm_2][pwbs_33]+ (7-[day2_arm_2][pwbs_34])+ [day2_arm_2][pwbs_35]+ (7-[day2_arm_2][pwbs_36])+ [day2_arm_2][pwbs_37]+ [day2_arm_2][pwbs_38]+ (7-[day2_arm_2][pwbs_39])+ [day2_arm_2][pwbs_40]+ (7-[day2_arm_2][pwbs_41])+ [day2_arm_2][pwbs_42]

**pwbs_autonomy**		PWBS Autonomy	[day2_arm_2][pwbs_1]+ [day2_arm_2][pwbs_7]+ (7-[day2_arm_2][pwbs_13])+ (7-[day2_arm_2][pwbs_19])+ [day2_arm_2][pwbs_25]+ (7-[day2_arm_2][pwbs_31])+ [day2_arm_2][pwbs_37]
**pwbs_enviromastery**		PWBS Environmental mastery	[day2_arm_2][pwbs_2]+ [day2_arm_2][pwbs_8]+ (7-[day2_arm_2][pwbs_14])+ [day2_arm_2][pwbs_20]+ (7-[day2_arm_2][pwbs_26])+ (7-[day2_arm_2][pwbs_32])+ [day2_arm_2][pwbs_38]
**pwbs_personal_gro**		PWBS Personal Growth	(7-[day2_arm_2][pwbs_3])+ [day2_arm_2][pwbs_9]+ (7-[day2_arm_2][pwbs_15])+ [day2_arm_2][pwbs_21]+ (7-[day2_arm_2][pwbs_27])+ [day2_arm_2][pwbs_33]+ (7-[day2_arm_2][pwbs_39])
**pwbs_pos_rel**		PWBS Positive Relations:	[day2_arm_2][pwbs_4]+ (7-[day2_arm_2][pwbs_10])+ (7-[day2_arm_2][pwbs_16])+ [day2_arm_2][pwbs_22]+ [day2_arm_2][pwbs_28]+ (7-[day2_arm_2][pwbs_34])+ [day2_arm_2][pwbs_40]
**pwbs_purp_life**		PWBS Purpose in life:	(7-[day2_arm_2][pwbs_5])+ [day2_arm_2][pwbs_11]+ (7-[day2_arm_2][pwbs_17])+ (7-[day2_arm_2][pwbs_23])+ [day2_arm_2][pwbs_29]+ [day2_arm_2][pwbs_35]+ (7-[day2_arm_2][pwbs_41])
**pwbs_self_accept**		PWBS Self-acceptance:	[day2_arm_2][pwbs_6]+ [day2_arm_2][pwbs_12]+ (7-[day2_arm_2][pwbs_18])+ [day2_arm_2][pwbs_24]+ (7-[day2_arm_2][pwbs_30])+

			(7-[day2_arm_2][pwbs_36])+<br>[day2_arm_2][pwbs_42]
**stai_y1_state_total**		STAY State Anxiety	(5-[day2_arm_2][stai_y1_calm_01])+<br>(5-[day2_arm_2][stai_y1_secure_02])+<br>[day2_arm_2][stai_y1_tense_03]+<br>[day2_arm_2][stai_y1_strain_04]+<br>(5-[day2_arm_2][stai_y1_ease_05])+<br>[day2_arm_2][stai_y1_upset_06]+<br>[day2_arm_2][stai_y1_worry_07]+<br>(5-[day2_arm_2][stai_y1_satis_08])+<br>[day2_arm_2][stai_y1_fright_09]+<br>(5-[day2_arm_2][stai_y1_comf_10])+<br>(5-[day2_arm_2][stai_y1_self_11])+<br>[day2_arm_2][stai_y1_nerv_12]+<br>[day2_arm_2][stai_y1_jittery_13]+<br>[day2_arm_2][stai_y1_indec_14]+<br>(5-[day2_arm_2][stai_y1_relax_15])+<br>(5-[day2_arm_2][stai_y1_content_16])+<br>[day2_arm_2][stai_y1_worry_17]+<br>[day2_arm_2][stai_y1_confuse_18]+<br>(5-[day2_arm_2][stai_y1_steady_19])+<br>(5-[day2_arm_2][stai_y1_pleasant_20])
**stai_y1_trait_total**		STAY Trait Anxiety Total	(5-[day2_arm_2][stai_y1_pleasant_01])+<br>[day2_arm_2][stai_y1_restless_02]+<br>(5-[day2_arm_2][stai_y1_satis_03])+<br>[day2_arm_2][stai_y1_happy_04]+<br>[day2_arm_2][stai_y1_failure_05]+<br>(5-[day2_arm_2][stai_y1_rested_06])+<br>(5-[day2_arm_2][stai_y1_cool_07])+<br>[day2_arm_2][stai_y1_pile_08]+<br>[day2_arm_2][stai_y1_worry_09]+<br>(5-[day2_arm_2][stai_y1_happy_10])+<br>[day2_arm_2][stai_y1_dist_11]+<br>[day2_arm_2][stai_y1_self_12]+<br>(5-[day2_arm_2][stai_y1_secure_13])+<br>(5-[day2_arm_2][stai_y1_easy_14])+<br>[day2_arm_2][stai_y1_inad_15]+<br>(5-[day2_arm_2][stai_y1_content_trait_16])+<br>[day2_arm_2][stai_y1_unimp_17]+<br>[day2_arm_2][stai_y1_disapp_18]+<br>(5-[day2_arm_2][stai_y1_steady_trait_19])+

			[day2_arm_2][stai_y1_concern_20]
**scor_abp1_psqi_comp_1**		PSQI - Subjective Sleep Quality	if([psqi_quality_1]='0',0,if([psqi_quality_1]='1',1,if([psqi_quality_1]='2',2,if([psqi_quality_1]='3',3,''))))
**scor_abp1_psqi_comp_2int**		PSQI - Sleep Latency Interim Number (sum of Q2 + Q5)	(if([psqi_fall_asleep_1]< 16,0,if([psqi_fall_asleep_1]< 31,1,if([psqi_fall_asleep_1]< 61,2,if([psqi_fall_asleep_1]>60,3,'')))))+(if([psqi_30_minutes_1]='0',0,if([psqi_30_minutes_1]='1',1,if([psqi_30_minutes_1]='2',2,if([psqi_30_minutes_1]='3',3,'')))))
**scor_abp1_psqi_comp_2**		PSQI - Sleep Latency	if([scor_abp1_psqi_comp_2int]='0',0,if([scor_abp1_psqi_comp_2int]< 3,1,if([scor_abp1_psqi_comp_2int]< 5,2,if([scor_abp1_psqi_comp_2int]< 7,3,''))))
**scor_abp1_psqi_comp_3**		PSQI - Sleep Duration	if([psqi_actual_sleep_1]>7,0,if([psqi_actual_sleep_1]>5,1,if([psqi_actual_sleep_1]=5,2,if([psqi_actual_sleep_1]< 5,3,''))))
**scor_abp1_psqi_comp_4_1**		PSQI - Bedtime time	[psqi_bedtime_1]
**scor_abp1_psqi_comp_4_2**		PSQI - Wake up time	[psqi_wake_time_1]
**scor_abp1_psqi_comp_4_3**		PSQI - Number of hours spent in bed	if(datediff([psqi_wake_time_1],[psqi_bedtime_1],'h')>12,(24-datediff([psqi_wake_time_1],[psqi_bedtime_1],'h')),datediff([psqi_wake_time_1],[psqi_bedtime_1],'h'))
**scor_abp1_psqi_comp_4**		PSQI - Habitual Sleep Efficiency	if((([psqi_actual_sleep_1]/[scor_abp1_psqi_comp_4_3])*100)>85,0,if((([psqi_actual_sleep_1]/[scor_abp1_psqi_comp_4_3])*100)>74,1,if((([psqi_actual_sleep_1]/[scor_abp1_psqi_comp_4_3])*100)>65,2,if((([psqi_actual_sleep_1]/[scor_abp1_psqi_comp_4_3])*100)< 65,3,''))))

**scor_abp1_psqi_comp_5**		PSQI - Sleep Disturbances	if(sum([psqi_middle_early_1],[psqi_bathroom_1],[psqi_breathe_1],[psqi_cough_snore_1],[psqi_cold_1],[psqi_hot_1],[psqi_dreams_1],[psqi_pain_1],[psqi_other_reason_freq_1])=0,0,if(sum ([psqi_middle_early_1],[psqi_bathroom_1],[psqi_breathe_1],[psqi_cough_snore_1],[psqi_cold_1],[psqi_hot_1],[psqi_dreams_1],[psqi_pain_1],[psqi_other_reason_freq_1])< 10,1,if(sum ([psqi_middle_early_1],[psqi_bathroom_1],[psqi_breathe_1],[psqi_cough_snore_1],[psqi_cold_1],[psqi_hot_1],[psqi_dreams_1],[psqi_pain_1],[psqi_other_reason_freq_1])< 19,2,if(sum ([psqi_middle_early_1],[psqi_bathroom_1],[psqi_breathe_1],[psqi_cough_snore_1],[psqi_cold_1],[psqi_hot_1],[psqi_dreams_1],[psqi_pain_1],[psqi_other_reason_freq_1])>18,3,''))))
**scor_abp1_psqi_comp_6**		PSQI - Use of Sleep Medication	[psqi_medicine_1]
**scor_abp1_psqi_comp_7**		PSQI - Daytime Dysfunction	if(([psqi_trouble_awake_1]+[psqi_enthusiasm_1])=0,0,if(([psqi_trouble_awake_1]+[psqi_enthusiasm_1])< 3,1,if(([psqi_trouble_awake_1]+[psqi_enthusiasm_1])< 5,2,if(([psqi_trouble_awake_1]+[psqi_enthusiasm_1])>4,3,''))))
**scor_abp1_psqi_global**		PSQI - Global Score	[scor_abp1_psqi_comp_1]+[scor_abp1_psqi_comp_2]+[scor_abp1_psqi_comp_3]+[scor_abp1_psqi_comp_4]+[scor_abp1_psqi_comp_5]+[scor_abp1_psqi_comp_6]+[scor_abp1_psqi_comp_7]
**pfs_1_phys**		pfs_1_phys	if([pfsq_1(0)]=1,0,if([pfsq_1(1)]=1,1,if([pfsq_1(2)]=1,2,if([pfsq_1(3)]=1,3,if([pfsq_1(4)]=1,4,if([pfsq_1(5)],5,5))))))
**pfs_2_phys**		pfs_2_phys	if([pfsq_2(0)]=1,0,if([pfsq_2(1)]=1,1,if([pfsq_2(2)]=1,2,if([pfsq_2(3)]=1,3,if([pfsq_2(4)]=1,4,if([pfsq_2(5)],5,5))))))
**pfs_3_phys**		pfs_3_phys	if([pfsq_3(0)]=1,0,if([pfsq_3(1)]=1,1,if([pfsq_3(2)]=1,2,if([pfsq_3(3)]=1,3,if([pfsq_3(4)]=1,4,if([pfsq_3(5)],5,5))))))
**pfs_4_phys**		pfs_4_phys	if([pfsq_4(0)]=1,0,if([pfsq_4(1)]=1,1,if([pfsq_4(2)]=1,2,if([pfsq_4(3)]=1,3,if([pfsq_4(4)]=1,4,if([pfsq_4(5)],5,5))))))
**pfs_5_phys**		pfs_5_phys	if([pfsq_5(0)]=1,0,if([pfsq_5(1)]=1,1,if([pfsq_5(2)]=1,2,if([pfsq_5(3)]=1,3,if([pfsq_5(4)]=1,4,if([pfsq_5(5)],5,5))))))

**pfs_6_phys**		pfs_6_phys	if([pfsq_6(0)]=1,0,if([pfsq_6(1)]=1,1,if([pfsq_6(2)]=1,2,if([pfsq_6(3)]=1,3,if([pfsq_6(4)]=1,4,if([pfsq_6(5)],5,5))))))
**pfs_7_phys**		pfs_7_phys	if([pfsq_7(0)]=1,0,if([pfsq_7(1)]=1,1,if([pfsq_7(2)]=1,2,if([pfsq_7(3)]=1,3,if([pfsq_7(4)]=1,4,if([pfsq_7(5)],5,5))))))
**pfs_8_phys**		pfs_8_phys	if([pfsq_8(0)]=1,0,if([pfsq_8(1)]=1,1,if([pfsq_8(2)]=1,2,if([pfsq_8(3)]=1,3,if([pfsq_8(4)]=1,4,if([pfsq_8(5)],5,5))))))
**pfs_9_phys**		pfs_9_phys	if([pfsq_9(0)]=1,0,if([pfsq_9(1)]=1,1,if([pfsq_9(2)]=1,2,if([pfsq_9(3)]=1,3,if([pfsq_9(4)]=1,4,if([pfsq_9(5)],5,5))))))
**pfs_10_phys**		pfs_10_phys	if([pfsq_10(0)]=1,0,if([pfsq_10(1)]=1,1,if([pfsq_10(2)]=1,2,if([pfsq_10(3)]=1,3,if([pfsq_10(4)]=1,4,if([pfsq_10(5)],5,5))))))
**pfs_1_mental**		pfs_1_mental	if([pfsq_1(10)]=1,0,if([pfsq_1(11)]=1,1,if([pfsq_1(12)]=1,2,if([pfsq_1(13)]=1,3,if([pfsq_1(14)]=1,4,if([pfsq_1(15)],5,5))))))
**pfs_2_mental**		pfs_2_mental	if([pfsq_2(10)]=1,0,if([pfsq_2(11)]=1,1,if([pfsq_2(12)]=1,2,if([pfsq_2(13)]=1,3,if([pfsq_2(14)]=1,4,if([pfsq_2(15)],5,5))))))
**pfs_3_mental**		pfs_3_mental	if([pfsq_3(10)]=1,0,if([pfsq_3(11)]=1,1,if([pfsq_3(12)]=1,2,if([pfsq_3(13)]=1,3,if([pfsq_3(14)]=1,4,if([pfsq_3(15)],5,5))))))
**pfs_4_mental**		pfs_4_mental	if([pfsq_4(10)]=1,0,if([pfsq_4(11)]=1,1,if([pfsq_4(12)]=1,2,if([pfsq_4(13)]=1,3,if([pfsq_4(14)]=1,4,if([pfsq_4(15)],5,5))))))
**pfs_5_mental**		pfs_5_mental	if([pfsq_5(10)]=1,0,if([pfsq_5(11)]=1,1,if([pfsq_5(12)]=1,2,if([pfsq_5(13)]=1,3,if([pfsq_5(14)]=1,4,if([pfsq_5(15)],5,5))))))
**pfs_6_mental**		pfs_6_mental	if([pfsq_6(10)]=1,0,if([pfsq_6(11)]=1,1,if([pfsq_6(12)]=1,2,if([pfsq_6(13)]=1,3,if([pfsq_6(14)]=1,4,if([pfsq_6(15)],5,5))))))
**pfs_7_mental**		pfs_7_mental	if([pfsq_7(10)]=1,0,if([pfsq_7(11)]=1,1,if([pfsq_7(12)]=1,2,if([pfsq_7(13)]=1,3,if([pfsq_7(14)]=1,4,if([pfsq_7(15)],5,5))))))
**pfs_8_mental**		pfs_8_mental	if([pfsq_8(10)]=1,0,if([pfsq_8(11)]=1,1,if([pfsq_8(12)]=1,2,if([pfsq_8(13)]=1,3,if([pfsq_8(14)]=1,4,if([pfsq_8(15)],5,5))))))
**pfs_9_mental**		pfs_9_mental	if([pfsq_9(10)]=1,0,if([pfsq_9(11)]=1,1,if([pfsq_9(12)]=1,2,if([pfsq_9(13)]=1,3,if([pfsq_9(14)]=1,4,if([pfsq_9(15)],5,5))))))

**pfs_10_mental**		pfs_10_mental	if([pfsq_10(10)]=1,0,if([pfsq_10(11)]=1,1,if([pfsq_10(12)]=1,2,if([pfsq_10(13)]=1,3,if([pfsq_10(14)]=1,4,if([pfsq_10(15)],5,5))))))
**pfs_phys**		Pittsburgh Fatiguability Scale Physical	[pfs_1_phys]+<br>[pfs_2_phys]+<br>[pfs_3_phys]+<br>[pfs_4_phys]+<br>[pfs_5_phys]+<br>[pfs_6_phys]+<br>[pfs_7_phys]+<br>[pfs_8_phys]+<br>[pfs_9_phys]+<br>[pfs_10_phys]
**pfs_mental**		Pittsburgh Fatiguability Scale Mental	[pfs_1_mental]+<br>[pfs_2_mental]+<br>[pfs_3_mental]+<br>[pfs_4_mental]+<br>[pfs_5_mental]+<br>[pfs_6_mental]+<br>[pfs_7_mental]+<br>[pfs_8_mental]+<br>[pfs_9_mental]+<br>[pfs_10_mental]
**compass_orth_int**	Compass	Orthosataic Intolerance	(round(sum([compass_1],[compass_2]-1,[compass_3],[compass_4])))*4
**compass_vasomotor**		Vasomotor	(if([compass_6(1)] = '1' and [compass_6(2)] = '1', round(sum([compass_5],[compass_7]))+2,if([compass_6(1)] <> '1' and [compass_6(2)] <>'1',round(sum([compass_5],[compass_7])),round(sum([compass_5],[compass_7]))+1)))*.83333
**compass_secretomotor**		Secretomotor	(round(sum([compass_8],[compass_9],[compass_10],[compass_11])))*2.1428571
**compass_gastrointest**		Gastrointestinsl	(round(sum([compass_12],[compass_13],[compass_14],[compass_15],([compass_17]-1),[compass_18],[compass_19],[compass_20],([compass_21]-1),[compass_22],[compass_23])))*.8928571
**compass_bladded**		Bladder	(round(sum(([compass_24]-1),([compass_25]-1),([compass_26]-1))))*1.111
**compass_pupillomotor**		Pupillomotor	(round(sum(([compass_27]-1),[compass_28],([compass_29]-1),[compass_30],[compass_31])))*.333
**compass_total**		Compass Total	sum([compass_orth_int],[compass_vasomotor],[compass_secretomotor],[compass_gastrointest],[compass_bladded],[compass_pupillomotor])

**mfis_phy_sub**	Modified Fatigue Impact Scale	Physical Subscale:	[mfis_4]+<br>[mfis_6]+<br>[mfis_7]+<br>[mfis_10]+<br>[mfis_13]+<br>[mfis_14]+<br>[mfis_17]+<br>[mfis_20]+<br>[mfis_21]
**mfis_cog_sub**		Cognitive Subscale:	[mfis_1]+<br>[mfis_2]+<br>[mfis_3]+<br>[mfis_5]+<br>[mfis_11]+<br>[mfis_12]+<br>[mfis_15]+<br>[mfis_16]+<br>[mfis_18]+<br>[mfis_19]
**mfis_psy_sub**		Psychosocial Subscale:	[mfis_8]+<br>[mfis_9]
**mfis_total**		Total Scale	[mfis_1]+<br>[mfis_2]+<br>[mfis_3]+<br>[mfis_4]+<br>[mfis_5]+<br>[mfis_6]+<br>[mfis_7]+<br>[mfis_8]+<br>[mfis_9]+<br>[mfis_10]+<br>[mfis_11]+<br>[mfis_12]+<br>[mfis_13]+<br>[mfis_14]+<br>[mfis_15]+<br>[mfis_16]+<br>[mfis_17]+<br>[mfis_18]+<br>[mfis_19]+<br>[mfis_20]+<br>[mfis_21]

**bipq_cog_ill_rep**	Brief Illness Perception Questionnaire	cognitive illness representations	[bipq_1]+[bipq_2]+(11-[bipq_3])+(11-[bipq_4])+[bipq_5]
**bipq_emo_rep**		emotiona illness representations	[bipq_6]+[bipq_8]
**bipq_ill_compreh**		illness comprehensibility	(11-[bipq_7])
**spa_score**	Self Perception of Aging	Spa	[spa_1]+<br>(6-[spa_2])+<br>[spa_3]+<br>(6-[spa_4])+<br>[spa_5]
**pss_total**		PSS	[day2_arm_2][pss1]+<br>[day2_arm_2][pss2]+<br>[day2_arm_2][pss3]+<br>(4-[day2_arm_2][pss4])+<br>(4-[day2_arm_2][pss5])+<br>[day2_arm_2][pss6]+<br>(4-[day2_arm_2][pss7])+<br>(4-[day2_arm_2][pss8])+<br>[day2_arm_2][pss9]+<br>[day2_arm_2][pss10]
**bdi_total**		BDI	if([day2_arm_2][bdi_diet_20]="1",[day2_arm_2][bdi_sad_01]+<br>[day2_arm_2][bdi_future_02]+ [day2_arm_2][bdi_fail_03]+<br>[day2_arm_2][bdi_satis_04]+ [day2_arm_2][bdi_guilt_05]+<br>[day2_arm_2][bdi_pun_06]+ [day2_arm_2][bdi_dis_07]+<br>[day2_arm_2][bdi_worse_08]+ [day2_arm_2][bdi_suic_09]+<br>[day2_arm_2][bdi_cry_10]+ [day2_arm_2][bdi_irrit_11]+<br>[day2_arm_2][bdi_people_12]+ [day2_arm_2][bdi_decis_13]+<br>[day2_arm_2][bdi_ugly_14]+ [day2_arm_2][bdi_work_15]+<br>[day2_arm_2][bdi_sleep_16]+ [day2_arm_2][bdi_tired_17]+<br>[day2_arm_2][bdi_eat_18]+ [day2_arm_2][bdi_health_21]+<br>[day2_arm_2][bdi_sex_22],[day2_arm_2][bdi_sad_01]+<br>[day2_arm_2][bdi_future_02]+ [day2_arm_2][bdi_fail_03]+

			[day2_arm_2][bdi_satis_04]+ [day2_arm_2][bdi_guilt_05]+ [day2_arm_2][bdi_pun_06]+ [day2_arm_2][bdi_dis_07]+ [day2_arm_2][bdi_worse_08]+ [day2_arm_2][bdi_suic_09]+ [day2_arm_2][bdi_cry_10]+ [day2_arm_2][bdi_irrit_11]+ [day2_arm_2][bdi_people_12]+ [day2_arm_2][bdi_decis_13]+ [day2_arm_2][bdi_ugly_14]+ [day2_arm_2][bdi_work_15]+ [day2_arm_2][bdi_sleep_16]+ [day2_arm_2][bdi_tired_17]+ [day2_arm_2][bdi_eat_18]+ [day2_arm_2][bdi_weight_19]+ [day2_arm_2][bdi_health_21]+ [day2_arm_2][bdi_sex_22])
**ctq_ea**	CTQ	CTQ 'Emotional Abuse' Subscale	[day2_arm_2][ctq_stupid_1]+[day2_arm_2][ctq_never_born_1]+[day2_arm_2][ctq_insulting_1]+[day2_arm_2][ctq_hated_1]+[day2_arm_2][ctq_emontionally_abused_1]
**ctq_pa**		CTQ 'Physical Abuse' Subscale	[day2_arm_2][ctq_hit_hospital_1]+[day2_arm_2][ctq_hit_bruises_1]+[day2_arm_2][ctq_belt_1]+[day2_arm_2][ctq_physically_abused_1]+[day2_arm_2][ctq_hit_teacher_1]
**ctq_sa**		CTQ 'Sexual Abuse' Subscale	[day2_arm_2][ctq_touch_sexual_1]+[day2_arm_2][ctq_threatened_sexual_1]+[day2_arm_2][ctq_do_watch_sexual_1]+[day2_arm_2][ctq_molested_1]+[day2_arm_2][ctq_sexually_abused_1]
**ctq_en**		CTQ 'Emotional Neglect' Subscale	[day2_arm_2][ctq_important_1]+[day2_arm_2][ctq_loved_1]+[day2_arm_2][ctq_looked_out_1]+[day2_arm_2][ctq_close_1]+[day2_arm_2][ctq_support_1]
**ctq_pn**		CTQ 'Physical Neglect' Subscale	[day2_arm_2][ctq_eat_1]+[day2_arm_2][ctq_protect_1]+[day2_arm_2][ctq_drunk_1]+[day2_arm_2][ctq_dirty_1]+[day2_arm_2][ctq_doctor_1]
**ctq_md**		CTQ 'Minimization/Denial' Subscale	round([day2_arm_2][ctq_no_change_1]+[day2_arm_2][ctq_perfect_1]+[day2_arm_2][ctq_best_1],0)
**srh_score**	SF-36 SRH Question (SRH)	Self rated health	6-[day2_arm_2][srh]
**csi_score**	Couples Satisfaction	Couple Satisfaction Index	[day2_arm_2][csi_1]+[day2_arm_2][csi_2]+[day2_arm_2][csi_3]+[day2_arm_2][csi_4]+[day2_arm_2][csi_5]+[day2_arm_2][csi_6]+[day2_arm_2][csi_7]+[day2_arm_2][csi_8]+[day2_arm_2][csi_9]+[day2_arm_2][csi_10]+[day2_arm_2][csi_11]+[day2_arm_2][csi_12_reversed]+[day2_arm_2][csi_13]+

			[day2_arm_2][csi_14]+[day2_arm_2][csi_15_reversed]+[day2_arm_2][csi_16]
**soc_compre**	Sense of coherence	Soc comprehensibility	(8-[day2_arm_2][soc_1])+[day2_arm_2][soc_3]+(8-[day2_arm_2][soc_5])+[day2_arm_2][soc_10]+[day2_arm_2][soc_12]+[day2_arm_2][soc_15]+[day2_arm_2][soc_17]+[day2_arm_2][soc_19]+[day2_arm_2][soc_21]+[day2_arm_2][soc_24]+[day2_arm_2][soc_26]
**soc_manage**		SOC manageability	[day2_arm_2][soc_2]+(8-[day2_arm_2][soc_6])+[day2_arm_2][soc_9]+(8-[day2_arm_2][soc_13])+[day2_arm_2][soc_18]+(8-[day2_arm_2][soc_20])+(8-[day2_arm_2][soc_23])+(8-[day2_arm_2][soc_25])+(8-[day2_arm_2][soc_27])+[day2_arm_2][soc_29]
**soc_meaning**		SOC meaning	(8-[day2_arm_2][soc_4])+(8-[day2_arm_2][soc_7])+[day2_arm_2][soc_8]+(8-[day2_arm_2][soc_11])+(8-[day2_arm_2][soc_14])+(8-[day2_arm_2][soc_16])+[day2_arm_2][soc_22]+[day2_arm_2][soc_28]
**neo_neuro**	NEO-FFI	NEO Neurotocism	(6-[day2_arm_2][neo_sf_1])+([day2_arm_2][neo_sf_6])+[day2_arm_2][neo_sf_11]+(6-[day2_arm_2][neo_sf_16])+[day2_arm_2][neo_sf_21]+[day2_arm_2][neo_sf_26]+(6-[day2_arm_2][neo_sf_31])+[day2_arm_2][neo_sf_36]+[day2_arm_2][neo_sf_41]+(6-[day2_arm_2][neo_sf_46])+[day2_arm_2][neo_sf_51]+[day2_arm_2][neo_sf_56]
**neo_extra**		NEO extraversion	[day2_arm_2][neo_sf_2]+[day2_arm_2][neo_sf_7]+(6-[day2_arm_2][neo_sf_12])+[day2_arm_2][neo_sf_17]+[day2_arm_2][neo_sf_22]+(6-[day2_arm_2][neo_sf_27])+[day2_arm_2][neo_sf_32]+[day2_arm_2][neo_sf_37]+(6-[day2_arm_2][neo_sf_42])+[day2_arm_2][neo_sf_47]+[day2_arm_2][neo_sf_52]+(6-[day2_arm_2][neo_sf_57])
**neo_open**		NEO Openess	(6-[day2_arm_2][neo_sf_3])+(6-[day2_arm_2][neo_sf_8])+[day2_arm_2][neo_sf_13]+(6-[day2_arm_2][neo_sf_18])+(6-[day2_arm_2][neo_sf_23])+[day2_arm_2][neo_sf_28]+(6-[day2_arm_2][neo_sf_33])+(6-[day2_arm_2][neo_sf_38])+[day2_arm_2][neo_sf_43]+(6-

			[day2_arm_2][neo_sf_48])+[day2_arm_2][neo_sf_53]+[day2_arm_2][neo_sf_58]
**neo_agree**		NEO Agreeableness	[day2_arm_2][neo_sf_4]+(6-[day2_arm_2][neo_sf_9])+(6-[day2_arm_2][neo_sf_14])+[day2_arm_2][neo_sf_19]+(6-[day2_arm_2][neo_sf_24])+(6-[day2_arm_2][neo_sf_29])+[day2_arm_2][neo_sf_34]+(6-[day2_arm_2][neo_sf_39])+(6-[day2_arm_2][neo_sf_44])+[day2_arm_2][neo_sf_49]+(6-[day2_arm_2][neo_sf_54])+[day2_arm_2][neo_sf_59]
**neo_cons**		NEO Conscientiousness	[day2_arm_2][neo_sf_5]+[day2_arm_2][neo_sf_10]+(6-[day2_arm_2][neo_sf_15])+[day2_arm_2][neo_sf_20]+[day2_arm_2][neo_sf_25]+(6-[day2_arm_2][neo_sf_30])+[day2_arm_2][neo_sf_35]+[day2_arm_2][neo_sf_40]+(6-[day2_arm_2][neo_sf_45])+[day2_arm_2][neo_sf_50]+(6-[day2_arm_2][neo_sf_55])+[day2_arm_2][neo_sf_60]
**bsri_mas**	BSRI	Masculinity	[day2_arm_2][bsri_1]+[day2_arm_2][bsri_4]+[day2_arm_2][bsri_7]+[day2_arm_2][bsri_10]+[day2_arm_2][bsri_13]+[day2_arm_2][bsri_16]+[day2_arm_2][bsri_19]+[day2_arm_2][bsri_22]+[day2_arm_2][bsri_25]+[day2_arm_2][bsri_28]+[day2_arm_2][bsri_31]+[day2_arm_2][bsri_34]+[day2_arm_2][bsri_37]+[day2_arm_2][bsri_40]+[day2_arm_2][bsri_43]+[day2_arm_2][bsri_46]+[day2_arm_2][bsri_49]+[day2_arm_2][bsri_52]+[day2_arm_2][bsri_55]+[day2_arm_2][bsri_58]
**bsri_fem**		Femininity	[day2_arm_2][bsri_2]+[day2_arm_2][bsri_5]+[day2_arm_2][bsri_8]+[day2_arm_2][bsri_11]+[day2_arm_2][bsri_14]+[day2_arm_2][bsri_17]+[day2_arm_2][bsri_20]+[day2_arm_2][bsri_23]+[day2_arm_2][bsri_26]+[day2_arm_2][bsri_29]+[day2_arm_2][bsri_32]+[day2_arm_2][bsri_35]+[day2_arm_2][bsri_38]+[day2_arm_2][bsri_41]+[day2_arm_2][bsri_44]+[day2_arm_2][bsri_47]+[day2_arm_2][bsri_50]+[day2_arm_2][bsri_53]+[day2_arm_2][bsri_56]+[day2_arm_2][bsri_59]
**bsri_neu**		Neutral	[day2_arm_2][bsri_3]+[day2_arm_2][bsri_6]+[day2_arm_2][bsri_9]+[day2_arm_2][bsri_12]+[day2_arm_2][bsri_15]+[day2_arm_2][bsri_18]+[day2_arm_2][bsri_21]+[day2_arm_2][bsri_24]+[day2_arm_2][bsri_27]+[day2_arm_2][bsri_30]+[day2_arm_2][bsri_33]+[day2_arm_2][bsri_36]+[day2_arm_2][bsri_39]+[day2_arm_2][bsri_42]+[day2_arm_2][bsri_45]+[day2_arm_2][bsri_48]+[day2_arm_2][bsri_51]+[day2_arm_2][bsri_54]+[day2_arm_2][bsri_57]+[day2_arm_2][bsri_60]

**mcc_score**	Memory Complaints	Memory Complaints Total	[day2_arm_2][mcc_1]+[day2_arm_2][mcc_2]+[day2_arm_2][mcc_3]+[day2_arm_2][mcc_4]+[day2_arm_2][mcc_5]+[day2_arm_2][mcc_6]+[day2_arm_2][mcc_7]+[day2_arm_2][mcc_8]+[day2_arm_2][mcc_9]+[day2_arm_2][mcc_10]+[day2_arm_2][mcc_11]+[day2_arm_2][mcc_12]+[day2_arm_2][mcc_13]+[day2_arm_2][mcc_14]+[day2_arm_2][mcc_15]+[day2_arm_2][mcc_16]+[day2_arm_2][mcc_17]+[day2_arm_2][mcc_18]+[day2_arm_2][mcc_19]+[day2_arm_2][mcc_20]+[day2_arm_2][mcc_21]+[day2_arm_2][mcc_22]+[day2_arm_2][mcc_23]+[day2_arm_2][mcc_24]+[day2_arm_2][mcc_25]+[day2_arm_2][mcc_26]+[day2_arm_2][mcc_27]+[day2_arm_2][mcc_28]+[day2_arm_2][mcc_29]+[day2_arm_2][mcc_30]
**mspss_score**	PSSQ	Perceived Social Support	[day2_arm_2][mspss_frd_1]+(5-[day2_arm_2][mspss_frd_2])+[day2_arm_2][mspss_frd_3]+[day2_arm_2][mspss_frd_4]+[day2_arm_2][mspss_frd_5]+(5-[day2_arm_2][mspss_frd_6])+(5-[day2_arm_2][mspss_frd_7])+[day2_arm_2][mspss_frd_8]+[day2_arm_2][mspss_frd_9]+[day2_arm_2][mspss_frd_10]+[day2_arm_2][mspss_frd_11]+[day2_arm_2][mspss_frd_12]+[day2_arm_2][mspss_frd_13]+[day2_arm_2][mspss_frd_14]+(5-[day2_arm_2][mspss_frd_15])+[day2_arm_2][mspss_frd_16]+[day2_arm_2][mspss_frd_17]+(5-[day2_arm_2][mspss_frd_18])+[day2_arm_2][mspss_frd_19]+(5-[day2_arm_2][mspss_frd_20])
**tics_total_score**	TICS	TICS	[day2_arm_2][tics_1]+[day2_arm_2][tics_2]+[day2_arm_2][tics_3]+[day2_arm_2][tics_4]+[day2_arm_2][tics_5]+[day2_arm_2][tics_6]+[day2_arm_2][tics_7]+[day2_arm_2][tics_8]+[day2_arm_2][tics_9]+[day2_arm_2][tics_10]+[day2_arm_2][tics_11]+[day2_arm_2][tics_12]+[day2_arm_2][tics_13]+[day2_arm_2][tics_14]+[day2_arm_2][tics_15]+[day2_arm_2][tics_16]+[day2_arm_2][tics_17]+[day2_arm_2][tics_18]+[day2_arm_2][tics_19]+[day2_arm_2][tics_20]+[day2_arm_2][tics_21]+[day2_arm_2][tics_22]+[day2_arm_2][tics_23]+[day2_arm_2][tics_24]+[day2_arm_2][tics_25]+[day2_arm_2][tics_26]+[day2_arm_2][tics_27]+[day2_arm_2][tics_28]+[day2_arm_2][tics_29]+[day2_arm_2][tics_30]
**tics_wo**		TICS work overload	[tics_1] + [tics_3] + [tics_21]
**tics_so**		TICS social overoad	[tics_11] + [tics_18] + [tics_28]
**tics_owo**		TICS overextended at work	[tics_12] + [tics_16] + [tics_27]

**tics_lso**		TICS lack of social recognition	[tics_2] + [tics_20] + [tics_23]
**tics_wdi**		TICS work discontent	[tics_8] + [tics_13] + [tics_24]
**tics_pwo**		TICS performance pressure at work	[tics_5] + [tics_14] + [tics_29]
**tics_pso**		TICS performance pressure in social interactions	[tics_6] + [tics_15] + [tics_22]
**tics_soi**		TICS social isolation	[tics_19] + [tics_25] + [tics_30]
**tics_wpr**		TICS worry propensity	[tics_7] + [tics_10] + [tics_17]
**dhs_score**	Daily Hassles Scale	Daily Hassles Scale	[day2_arm_2][dhs_1]+[day2_arm_2][dhs_2]+[day2_arm_2][dhs_3]+[day2_arm_2][dhs_4]+[day2_arm_2][dhs_5]+[day2_arm_2][dhs_6]+[day2_arm_2][dhs_7]+[day2_arm_2][dhs_8]+[day2_arm_2][dhs_9]+[day2_arm_2][dhs_10]+[day2_arm_2][dhs_11]+[day2_arm_2][dhs_12]+[day2_arm_2][dhs_13]+[day2_arm_2][dhs_14]+[day2_arm_2][dhs_15]+[day2_arm_2][dhs_16]+[day2_arm_2][dhs_17]+[day2_arm_2][dhs_18]+[day2_arm_2][dhs_19]+[day2_arm_2][dhs_20]+[day2_arm_2][dhs_21]+[day2_arm_2][dhs_22]+[day2_arm_2][dhs_23]+[day2_arm_2][dhs_24]+[day2_arm_2][dhs_25]+[day2_arm_2][dhs_26]+[day2_arm_2][dhs_27]+[day2_arm_2][dhs_28]+[day2_arm_2][dhs_29]+[day2_arm_2][dhs_30]+[day2_arm_2][dhs_31]+[day2_arm_2][dhs_32]+[day2_arm_2][dhs_33]+[day2_arm_2][dhs_34]+[day2_arm_2][dhs_35]
**ucla_score**	UCLA Loneliness Scale	UCLA Loneliness Scale	(5-[day2_arm_2][ucla_1])+[day2_arm_2][ucla_2]+[day2_arm_2][ucla_3]+[day2_arm_2][ucla_4]+(5-[day2_arm_2][ucla_5])+(5-[day2_arm_2][ucla_6])+[day2_arm_2][ucla_7]+[day2_arm_2][ucla_8]+(5-[day2_arm_2][ucla_9])+(5-[day2_arm_2][ucla_10])+[day2_arm_2][ucla_11]+[day2_arm_2][ucla_12]+[day2_arm_2][ucla_13]+[day2_arm_2][ucla_14]+(5-[day2_arm_2][ucla_15])+(5-[day2_arm_2][ucla_16])+[day2_arm_2][ucla_17]+[day2_arm_2][ucla_18]+(5-[day2_arm_2][ucla_19])+(5-[day2_arm_2][ucla_20])

**mbi_exhaus**	Maslach Burnout Inventory	MBI exhaustion	([day2_arm_2][mbi_1]+[day2_arm_2][mbi_2]+[day2_arm_2][mbi_3]+[day2_arm_2][mbi_4]+[day2_arm_2][mbi_6]+[day2_arm_2][mbi_7])/6
**mbi_cyn**		MBI Cynicism	([day2_arm_2][mbi_9]+[day2_arm_2][mbi_10]+[day2_arm_2][mbi_13]+[day2_arm_2][mbi_14]+[day2_arm_2][mbi_15])/5
**mbi_ineff**		MBI Professional Efficacy	([day2_arm_2][mbi_5]+[day2_arm_2][mbi_8]+[day2_arm_2][mbi_11]+[day2_arm_2][mbi_12]+[day2_arm_2][mbi_16])/5
**pcl_total**		PCL-5 Score	[day2_arm_2][pcl_1]+[day2_arm_2][pcl_2]+[day2_arm_2][pcl_3]+[day2_arm_2][pcl_4]+[day2_arm_2][pcl_5]+[day2_arm_2][pcl_6]+[day2_arm_2][pcl_7]+[day2_arm_2][pcl_8]+[day2_arm_2][pcl_9]+[day2_arm_2][pcl_10]+[day2_arm_2][pcl_11]+[day2_arm_2][pcl_12]+[day2_arm_2][pcl_13]+[day2_arm_2][pcl_14]+[day2_arm_2][pcl_15]+[day2_arm_2][pcl_16]+[day2_arm_2][pcl_17]

**sqffs_sat_fat**	Short Questionnaire for Fat and Free Sugars (SQFFS)	Saturated fat subscale	[day1_arm_2][sqffs_mince_d1]+<br>[day1_arm_2][sqffs_beef_d1]+<br>[day1_arm_2][sqffs_friedchicken_d1]+<br>[day1_arm_2][sqffs_sausages_d1]+<br>[day1_arm_2][sqffs_bacon_d1]+<br>[day1_arm_2][sqffs_saladdressing_d1]+<br>[day1_arm_2][sqffs_margarine_d1]+<br>[day1_arm_2][sqffs_eggs_d1]+<br>[day1_arm_2][sqffs_pizza_d1]+<br>[day1_arm_2][sqffsz_cheese_d1]+<br>[day1_arm_2][sqffs_frenchfries_d1]+<br><br>[day1_arm_2][sqffs_cornchips_d1]
**sqffs_sugar**		Sugar subscale	[day1_arm_2][sqffs_lollies_d1]+<br>[day1_arm_2][sqffs_sportsdrinks_d1]+<br>[day1_arm_2][sqffs_softdrink_d1]+<br>[day1_arm_2][sqffs_sweeteneddrink_d1]+<br>[day1_arm_2][sqffs_whitebread_d1]+<br>[day1_arm_2][sqffs_addedsugar_d1]
**sqffs_sugar_fat**		Fat-sugarsubscale	[day1_arm_2][sqffs_doughnuts_d1]+<br>[day1_arm_2][sqffs_cakes_d1]+<br>[day1_arm_2][sqffs_icecream_d1]+<br>[day1_arm_2][sqffs_chocolate_d1]+<br>[day1_arm_2][sqffs_spreads_d1]+<br>[day1_arm_2][sqffs_pancakes_d1]+<br>[day1_arm_2][sqffs_milk_d1]+<br>[day1_arm_2][sqffs_fastfood_d1]
**pasa**	PASA	PASA	(7-[day1_arm_2][pasa_1])+<br>[day1_arm_2][pasa_5]+<br>(7-[day1_arm_2][pasa_9])+<br>[day1_arm_2][pasa_13]
**meq_total**	Morningness and Eveningness Questionnaire (MEQ)	MEQ total	[day1_arm_2][meq_1_h_rc]+<br>[day1_arm_2][meq_2_h_rc]+<br>[day1_arm_2][meq_3]+<br>[day1_arm_2][meq_4]+<br>[day1_arm_2][meq_5]+<br>[day1_arm_2][meq_6]+<br>[day1_arm_2][meq_7]+<br>[day1_arm_2][meq_8]+<br>[day1_arm_2][meq_9]+

			[day1_arm_2][meq_10_h_rc]+<br>[day1_arm_2][meq_11]+<br>if([day1_arm_2][meq_12]=4,5,[day1_arm_2][meq_12])+<br>[day1_arm_2][meq_13]+<br>[day1_arm_2][meq_14]+<br>[day1_arm_2][meq_15]+<br>[day1_arm_2][meq_16]+<br>[day1_arm_2][meq_17]+<br>[day1_arm_2][meq_18]+<br>[day1_arm_2][meq_19]
**meq_type**		MEQ	
**ssq_s**	SSQ	SSQ S	[day1_arm_2][ssq_1_help_satis]+<br>[day1_arm_2][ssq_2_relaxed_satis]+<br>[day1_arm_2][ssq_3_acceptance_satis]+<br>[day1_arm_2][ssq_4_care_satis]+<br>[day1_arm_2][ssq_5_feelbetter_satis]+<br>[day1_arm_2][ssq_6_console_satis]
**ssq_s_average**		SSQ S Average	([day1_arm_2][ssq_1_help_satis]+<br>[day1_arm_2][ssq_2_relaxed_satis]+<br>[day1_arm_2][ssq_3_acceptance_satis]+<br>[day1_arm_2][ssq_4_care_satis]+<br>[day1_arm_2][ssq_5_feelbetter_satis]+<br>[day1_arm_2][ssq_6_console_satis])/6
**ssq_n**		SSQ N	if([day1_arm_2][ssq_1_help]<>'',1,0)+<br>if([day1_arm_2][ssq_2_relaxed]<>'',1,0)+<br>if([day1_arm_2][ssq_3_acceptance]<>'',1,0)+<br>if([day1_arm_2][ssq_4_care]<>'',1,0)+<br>if([day1_arm_2][ssq_5_feelbetter]<>'',1,0)+<br>if([day1_arm_2][ssq_6console]<>'',1,0)+<br>if([day1_arm_2][ssq_1b]<>'',1,0)+<br>if([day1_arm_2][ssq_1c]<>'',1,0)+<br>if([day1_arm_2][ssq_1d]<>'',1,0)+

			if([day1_arm_2][ssq_1e]<>",1,0)+<br>if([day1_arm_2][ssq_1f]<>",1,0)+<br>if([day1_arm_2][ssq_1g]<>",1,0)+<br>if([day1_arm_2][ssq_1h]<>",1,0)+<br>if([day1_arm_2][ssq_1i]<>",1,0)+<br>if([day1_arm_2][ssq_2b]<>",1,0)+<br>if([day1_arm_2][ssq_2c]<>",1,0)+<br>if([day1_arm_2][ssq_2d]<>",1,0)+<br>if([day1_arm_2][ssq_2e]<>",1,0)+<br>if([day1_arm_2][ssq_2f]<>",1,0)+<br>if([day1_arm_2][ssq_2g]<>",1,0)+<br>if([day1_arm_2][ssq_2h]<>",1,0)+<br>if([day1_arm_2][ssq_2i]<>",1,0)+<br>if([day1_arm_2][ssq_3b]<>",1,0)+<br>if([day1_arm_2][ssq_3c]<>",1,0)+<br>if([day1_arm_2][ssq_3d]<>",1,0)+<br>if([day1_arm_2][ssq_3e]<>",1,0)+<br>if([day1_arm_2][ssq_3f]<>",1,0)+<br>if([day1_arm_2][ssq_3g]<>",1,0)+<br>if([day1_arm_2][ssq_3h]<>",1,0)+<br>if([day1_arm_2][ssq_3i]<>",1,0)+<br>if([day1_arm_2][ssq_4b]<>",1,0)+<br>if([day1_arm_2][ssq_4c]<>",1,0)+<br>if([day1_arm_2][ssq_4d]<>",1,0)+<br>if([day1_arm_2][ssq_4e]<>",1,0)+<br>if([day1_arm_2][ssq_4f]<>",1,0)+<br>if([day1_arm_2][ssq_4g]<>",1,0)+<br>if([day1_arm_2][ssq_4h]<>",1,0)+<br>if([day1_arm_2][ssq_4i]<>",1,0)+<br>if([day1_arm_2][ssq_5b]<>",1,0)+<br>if([day1_arm_2][ssq_5c]<>",1,0)+<br>if([day1_arm_2][ssq_5d]<>",1,0)+<br>if([day1_arm_2][ssq_5e]<>",1,0)+<br>if([day1_arm_2][ssq_5f]<>",1,0)+<br>if([day1_arm_2][ssq_5g]<>",1,0)+<br>if([day1_arm_2][ssq_5h]<>",1,0)+<br>if([day1_arm_2][ssq_5i]<>",1,0)+<br>if([day1_arm_2][ssq_6b]<>",1,0)+<br>if([day1_arm_2][ssq_6c]<>",1,0)+<br>if([day1_arm_2][ssq_6d]<>",1,0)+<br>if([day1_arm_2][ssq_6e]<>",1,0)+<br>if([day1_arm_2][ssq_6f]<>",1,0)+<br>if([day1_arm_2][ssq_6g]<>",1,0)+<br>if([day1_arm_2][ssq_6h]<>",1,0)+

			if([day1_arm_2][ssq_6i]<>",1,0)
**ssq_n_average**		SSQ N Average	(if([day1_arm_2][ssq_1_help]<>",1,0)+<br>if([day1_arm_2][ssq_2_relaxed]<>",1,0)+<br>if([day1_arm_2][ssq_3_acceptance]<>",1,0)+<br>if([day1_arm_2][ssq_4_care]<>",1,0)+<br>if([day1_arm_2][ssq_5_feelbetter]<>",1,0)+<br>if([day1_arm_2][ssq_6console]<>",1,0)+<br>if([day1_arm_2][ssq_1b]<>",1,0)+<br>if([day1_arm_2][ssq_1c]<>",1,0)+<br>if([day1_arm_2][ssq_1d]<>",1,0)+<br>if([day1_arm_2][ssq_1e]<>",1,0)+<br>if([day1_arm_2][ssq_1f]<>",1,0)+<br>if([day1_arm_2][ssq_1g]<>",1,0)+<br>if([day1_arm_2][ssq_1h]<>",1,0)+<br>if([day1_arm_2][ssq_1i]<>",1,0)+<br>if([day1_arm_2][ssq_2b]<>",1,0)+<br>if([day1_arm_2][ssq_2c]<>",1,0)+<br>if([day1_arm_2][ssq_2d]<>",1,0)+<br>if([day1_arm_2][ssq_2e]<>",1,0)+<br>if([day1_arm_2][ssq_2f]<>",1,0)+<br>if([day1_arm_2][ssq_2g]<>",1,0)+<br>if([day1_arm_2][ssq_2h]<>",1,0)+<br>if([day1_arm_2][ssq_2i]<>",1,0)+<br>if([day1_arm_2][ssq_3b]<>",1,0)+<br>if([day1_arm_2][ssq_3c]<>",1,0)+<br>if([day1_arm_2][ssq_3d]<>",1,0)+<br>if([day1_arm_2][ssq_3e]<>",1,0)+<br>if([day1_arm_2][ssq_3f]<>",1,0)+<br>if([day1_arm_2][ssq_3g]<>",1,0)+<br>if([day1_arm_2][ssq_3h]<>",1,0)+<br>if([day1_arm_2][ssq_3i]<>",1,0)+<br>if([day1_arm_2][ssq_4b]<>",1,0)+<br>if([day1_arm_2][ssq_4c]<>",1,0)+<br>if([day1_arm_2][ssq_4d]<>",1,0)+<br>if([day1_arm_2][ssq_4e]<>",1,0)+<br>if([day1_arm_2][ssq_4f]<>",1,0)+<br>if([day1_arm_2][ssq_4g]<>",1,0)+

			if([day1_arm_2][ssq_4h]<>",1,0)+<br>if([day1_arm_2][ssq_4i]<>",1,0)+<br>if([day1_arm_2][ssq_5b]<>",1,0)+<br>if([day1_arm_2][ssq_5c]<>",1,0)+<br>if([day1_arm_2][ssq_5d]<>",1,0)+<br>if([day1_arm_2][ssq_5e]<>",1,0)+<br>if([day1_arm_2][ssq_5f]<>",1,0)+<br>if([day1_arm_2][ssq_5g]<>",1,0)+<br>if([day1_arm_2][ssq_5h]<>",1,0)+<br>if([day1_arm_2][ssq_5i]<>",1,0)+<br>if([day1_arm_2][ssq_6b]<>",1,0)+<br>if([day1_arm_2][ssq_6c]<>",1,0)+<br>if([day1_arm_2][ssq_6d]<>",1,0)+<br>if([day1_arm_2][ssq_6e]<>",1,0)+<br>if([day1_arm_2][ssq_6f]<>",1,0)+<br>if([day1_arm_2][ssq_6g]<>",1,0)+<br>if([day1_arm_2][ssq_6h]<>",1,0)+<br>if([day1_arm_2][ssq_6i]<>",1,0))/6
**leq_positive_events_score**	LEQ	positive events score	if([day2_arm_2][leq_1(9)]=1,[day2_arm_2][leq_value_1],0)+<br>if([day2_arm_2][leq_2(9)]=1,[day2_arm_2][leq_value_2],0)+<br>if([day2_arm_2][leq_3(9)]=1,[day2_arm_2][leq_value_3],0)+<br>if([day2_arm_2][leq_4(9)]=1,[day2_arm_2][leq_value_4],0)+<br>if([day2_arm_2][leq_5(9)]=1,[day2_arm_2][leq_value_5],0)+<br>if([day2_arm_2][leq_6(9)]=1,[day2_arm_2][leq_value_6],0)+<br>if([day2_arm_2][leq_7(9)]=1,[day2_arm_2][leq_value_7],0)+<br>if([day2_arm_2][leq_8(9)]=1,[day2_arm_2][leq_value_8],0)+<br>if([day2_arm_2][leq_9(9)]=1,[day2_arm_2][leq_value_9],0)+<br>if([day2_arm_2][leq_10(9)]=1,[day2_arm_2][leq_value_10],0)+<br>if([day2_arm_2][leq_11(9)]=1,[day2_arm_2][leq_value_11],0)+<br>if([day2_arm_2][leq_12(9)]=1,[day2_arm_2][leq_value_12],0)+<br>if([day2_arm_2][leq_13(9)]=1,[day2_arm_2][leq_value_13],0)+<br>if([day2_arm_2][leq_14(9)]=1,[day2_arm_2][leq_value_14],0)+<br>if([day2_arm_2][leq_15(9)]=1,[day2_arm_2][leq_value_15],0)+<br>if([day2_arm_2][leq_16(9)]=1,[day2_arm_2][leq_value_16],0)+<br>if([day2_arm_2][leq_17(9)]=1,[day2_arm_2][leq_value_17],0)+<br>if([day2_arm_2][leq_18(9)]=1,[day2_arm_2][leq_value_18],0)+<br>if([day2_arm_2][leq_19(9)]=1,[day2_arm_2][leq_value_19],0)+<br>if([day2_arm_2][leq_20(9)]=1,[day2_arm_2][leq_value_20],0)+<br>if([day2_arm_2][leq_21(9)]=1,[day2_arm_2][leq_value_21],0)+<br>if([day2_arm_2][leq_22(9)]=1,[day2_arm_2][leq_value_22],0)+<br>if([day2_arm_2][leq_23(9)]=1,[day2_arm_2][leq_value_23],0)+<br>if([day2_arm_2][leq_24(9)]=1,[day2_arm_2][leq_value_24],0)+<br>if([day2_arm_2][leq_25(9)]=1,[day2_arm_2][leq_value_25],0)+<br>if([day2_arm_2][leq_26(9)]=1,[day2_arm_2][leq_value_26],0)+

			if([day2_arm_2][leq_27(9)]=1,[day2_arm_2][leq_value_27],0)+ if([day2_arm_2][leq_28(9)]=1,[day2_arm_2][leq_value_28],0)+ if([day2_arm_2][leq_29(9)]=1,[day2_arm_2][leq_value_29],0)+ if([day2_arm_2][leq_30(9)]=1,[day2_arm_2][leq_value_30],0)+ if([day2_arm_2][leq_31(9)]=1,[day2_arm_2][leq_value_31],0)+ if([day2_arm_2][leq_32(9)]=1,[day2_arm_2][leq_value_32],0)+ if([day2_arm_2][leq_33(9)]=1,[day2_arm_2][leq_value_33],0)+ if([day2_arm_2][leq_34(9)]=1,[day2_arm_2][leq_value_34],0)+ if([day2_arm_2][leq_35(9)]=1,[day2_arm_2][leq_value_35],0)+ if([day2_arm_2][leq_36(9)]=1,[day2_arm_2][leq_value_36],0)+ if([day2_arm_2][leq_37(9)]=1,[day2_arm_2][leq_value_37],0)+ if([day2_arm_2][leq_38(9)]=1,[day2_arm_2][leq_value_38],0)+ if([day2_arm_2][leq_39(9)]=1,[day2_arm_2][leq_value_39],0)+ if([day2_arm_2][leq_40(9)]=1,[day2_arm_2][leq_value_40],0)+ if([day2_arm_2][leq_41(9)]=1,[day2_arm_2][leq_value_41],0)+ if([day2_arm_2][leq_42(9)]=1,[day2_arm_2][leq_value_42],0)+ if([day2_arm_2][leq_43(9)]=1,[day2_arm_2][leq_value_43],0)+ if([day2_arm_2][leq_44(9)]=1,[day2_arm_2][leq_value_44],0)+ if([day2_arm_2][leq_45(9)]=1,[day2_arm_2][leq_value_45],0)+ if([day2_arm_2][leq_46(9)]=1,[day2_arm_2][leq_value_46],0)+ if([day2_arm_2][leq_47(9)]=1,[day2_arm_2][leq_value_47],0)+ if([day2_arm_2][leq_48(9)]=1,[day2_arm_2][leq_value_48],0)+ if([day2_arm_2][leq_49(9)]=1,[day2_arm_2][leq_value_49],0)+ if([day2_arm_2][leq_50(9)]=1,[day2_arm_2][leq_value_50],0)+ if([day2_arm_2][leq_51(9)]=1,[day2_arm_2][leq_value_51],0)+ if([day2_arm_2][leq_52(9)]=1,[day2_arm_2][leq_value_52],0)+ if([day2_arm_2][leq_53(9)]=1,[day2_arm_2][leq_value_53],0)+ if([day2_arm_2][leq_54(9)]=1,[day2_arm_2][leq_value_54],0)+ if([day2_arm_2][leq_55(9)]=1,[day2_arm_2][leq_value_55],0)+ if([day2_arm_2][leq_56(9)]=1,[day2_arm_2][leq_value_56],0)+ if([day2_arm_2][leq_57(9)]=1,[day2_arm_2][leq_value_57],0)+ if([day2_arm_2][leq_58(9)]=1,[day2_arm_2][leq_value_58],0)+ if([day2_arm_2][leq_59(9)]=1,[day2_arm_2][leq_value_59],0)+ if([day2_arm_2][leq_60(9)]=1,[day2_arm_2][leq_value_60],0)+ if([day2_arm_2][leq_61(9)]=1,[day2_arm_2][leq_value_61],0)+ if([day2_arm_2][leq_62(9)]=1,[day2_arm_2][leq_value_62],0)+ if([day2_arm_2][leq_63(9)]=1,[day2_arm_2][leq_value_63],0)+ if([day2_arm_2][leq_64(9)]=1,[day2_arm_2][leq_value_64],0)+ if([day2_arm_2][leq_65(9)]=1,[day2_arm_2][leq_value_65],0)+ if([day2_arm_2][leq_66(9)]=1,[day2_arm_2][leq_value_66],0)+ if([day2_arm_2][leq_67(9)]=1,[day2_arm_2][leq_value_67],0)+ if([day2_arm_2][leq_68(9)]=1,[day2_arm_2][leq_value_68],0)+ if([day2_arm_2][leq_69(9)]=1,[day2_arm_2][leq_value_69],0)+ if([day2_arm_2][leq_70(9)]=1,[day2_arm_2][leq_value_70],0)+ if([day2_arm_2][leq_71(9)]=1,[day2_arm_2][leq_value_71],0)+

			if([day2_arm_2][leq_72(9)]=1,[day2_arm_2][leq_value_72],0)+<br>if([day2_arm_2][leq_73(9)]=1,[day2_arm_2][leq_value_73],0)+<br>if([day2_arm_2][leq_74(9)]=1,[day2_arm_2][leq_value_74],0)+<br>if([day2_arm_2][leq_75(9)]=1,[day2_arm_2][leq_value_75],0)+<br>if([day2_arm_2][leq_76(9)]=1,[day2_arm_2][leq_value_76],0)+<br>if([day2_arm_2][leq_77(9)]=1,[day2_arm_2][leq_value_77],0)+<br>if([day2_arm_2][leq_78(9)]=1,[day2_arm_2][leq_value_78],0)+<br>if([day2_arm_2][leq_79(9)]=1,[day2_arm_2][leq_value_79],0)
**leq_negative_events_score**		negative events score	if([day2_arm_2][leq_1(10)]=1,[day2_arm_2][leq_value_1],0)+<br>if([day2_arm_2][leq_2(10)]=1,[day2_arm_2][leq_value_2],0)+<br>if([day2_arm_2][leq_3(10)]=1,[day2_arm_2][leq_value_3],0)+<br>if([day2_arm_2][leq_4(10)]=1,[day2_arm_2][leq_value_4],0)+<br>if([day2_arm_2][leq_5(10)]=1,[day2_arm_2][leq_value_5],0)+<br>if([day2_arm_2][leq_6(10)]=1,[day2_arm_2][leq_value_6],0)+<br>if([day2_arm_2][leq_7(10)]=1,[day2_arm_2][leq_value_7],0)+<br>if([day2_arm_2][leq_8(10)]=1,[day2_arm_2][leq_value_8],0)+<br>if([day2_arm_2][leq_9(10)]=1,[day2_arm_2][leq_value_9],0)+<br>if([day2_arm_2][leq_10(10)]=1,[day2_arm_2][leq_value_10],0)+<br>if([day2_arm_2][leq_11(10)]=1,[day2_arm_2][leq_value_11],0)+<br>if([day2_arm_2][leq_12(10)]=1,[day2_arm_2][leq_value_12],0)+<br>if([day2_arm_2][leq_13(10)]=1,[day2_arm_2][leq_value_13],0)+<br>if([day2_arm_2][leq_14(10)]=1,[day2_arm_2][leq_value_14],0)+<br>if([day2_arm_2][leq_15(10)]=1,[day2_arm_2][leq_value_15],0)+<br>if([day2_arm_2][leq_16(10)]=1,[day2_arm_2][leq_value_16],0)+<br>if([day2_arm_2][leq_17(10)]=1,[day2_arm_2][leq_value_17],0)+<br>if([day2_arm_2][leq_18(10)]=1,[day2_arm_2][leq_value_18],0)+<br>if([day2_arm_2][leq_19(10)]=1,[day2_arm_2][leq_value_19],0)+<br>if([day2_arm_2][leq_20(10)]=1,[day2_arm_2][leq_value_20],0)+<br>if([day2_arm_2][leq_21(10)]=1,[day2_arm_2][leq_value_21],0)+<br>if([day2_arm_2][leq_22(10)]=1,[day2_arm_2][leq_value_22],0)+<br>if([day2_arm_2][leq_23(10)]=1,[day2_arm_2][leq_value_23],0)+<br>if([day2_arm_2][leq_24(10)]=1,[day2_arm_2][leq_value_24],0)+<br>if([day2_arm_2][leq_25(10)]=1,[day2_arm_2][leq_value_25],0)+<br>if([day2_arm_2][leq_26(10)]=1,[day2_arm_2][leq_value_26],0)+<br>if([day2_arm_2][leq_27(10)]=1,[day2_arm_2][leq_value_27],0)+<br>if([day2_arm_2][leq_28(10)]=1,[day2_arm_2][leq_value_28],0)+<br>if([day2_arm_2][leq_29(10)]=1,[day2_arm_2][leq_value_29],0)+<br>if([day2_arm_2][leq_30(10)]=1,[day2_arm_2][leq_value_30],0)+<br>if([day2_arm_2][leq_31(10)]=1,[day2_arm_2][leq_value_31],0)+<br>if([day2_arm_2][leq_32(10)]=1,[day2_arm_2][leq_value_32],0)+<br>if([day2_arm_2][leq_33(10)]=1,[day2_arm_2][leq_value_33],0)+<br>if([day2_arm_2][leq_34(10)]=1,[day2_arm_2][leq_value_34],0)+<br>if([day2_arm_2][leq_35(10)]=1,[day2_arm_2][leq_value_35],0)+<br>if([day2_arm_2][leq_36(10)]=1,[day2_arm_2][leq_value_36],0)+

			if([day2_arm_2][leq_37(10)]=1,[day2_arm_2][leq_value_37],0)+ if([day2_arm_2][leq_38(10)]=1,[day2_arm_2][leq_value_38],0)+ if([day2_arm_2][leq_39(10)]=1,[day2_arm_2][leq_value_39],0)+ if([day2_arm_2][leq_40(10)]=1,[day2_arm_2][leq_value_40],0)+ if([day2_arm_2][leq_41(10)]=1,[day2_arm_2][leq_value_41],0)+ if([day2_arm_2][leq_42(10)]=1,[day2_arm_2][leq_value_42],0)+ if([day2_arm_2][leq_43(10)]=1,[day2_arm_2][leq_value_43],0)+ if([day2_arm_2][leq_44(10)]=1,[day2_arm_2][leq_value_44],0)+ if([day2_arm_2][leq_45(10)]=1,[day2_arm_2][leq_value_45],0)+ if([day2_arm_2][leq_46(10)]=1,[day2_arm_2][leq_value_46],0)+ if([day2_arm_2][leq_47(10)]=1,[day2_arm_2][leq_value_47],0)+ if([day2_arm_2][leq_48(10)]=1,[day2_arm_2][leq_value_48],0)+ if([day2_arm_2][leq_49(10)]=1,[day2_arm_2][leq_value_49],0)+ if([day2_arm_2][leq_50(10)]=1,[day2_arm_2][leq_value_50],0)+ if([day2_arm_2][leq_51(10)]=1,[day2_arm_2][leq_value_51],0)+ if([day2_arm_2][leq_52(10)]=1,[day2_arm_2][leq_value_52],0)+ if([day2_arm_2][leq_53(10)]=1,[day2_arm_2][leq_value_53],0)+ if([day2_arm_2][leq_54(10)]=1,[day2_arm_2][leq_value_54],0)+ if([day2_arm_2][leq_55(10)]=1,[day2_arm_2][leq_value_55],0)+ if([day2_arm_2][leq_56(10)]=1,[day2_arm_2][leq_value_56],0)+ if([day2_arm_2][leq_57(10)]=1,[day2_arm_2][leq_value_57],0)+ if([day2_arm_2][leq_58(10)]=1,[day2_arm_2][leq_value_58],0)+ if([day2_arm_2][leq_59(10)]=1,[day2_arm_2][leq_value_59],0)+ if([day2_arm_2][leq_60(10)]=1,[day2_arm_2][leq_value_60],0)+ if([day2_arm_2][leq_61(10)]=1,[day2_arm_2][leq_value_61],0)+ if([day2_arm_2][leq_62(10)]=1,[day2_arm_2][leq_value_62],0)+ if([day2_arm_2][leq_63(10)]=1,[day2_arm_2][leq_value_63],0)+ if([day2_arm_2][leq_64(10)]=1,[day2_arm_2][leq_value_64],0)+ if([day2_arm_2][leq_65(10)]=1,[day2_arm_2][leq_value_65],0)+ if([day2_arm_2][leq_66(10)]=1,[day2_arm_2][leq_value_66],0)+ if([day2_arm_2][leq_67(10)]=1,[day2_arm_2][leq_value_67],0)+ if([day2_arm_2][leq_68(10)]=1,[day2_arm_2][leq_value_68],0)+ if([day2_arm_2][leq_69(10)]=1,[day2_arm_2][leq_value_69],0)+ if([day2_arm_2][leq_70(10)]=1,[day2_arm_2][leq_value_70],0)+ if([day2_arm_2][leq_71(10)]=1,[day2_arm_2][leq_value_71],0)+ if([day2_arm_2][leq_72(10)]=1,[day2_arm_2][leq_value_72],0)+ if([day2_arm_2][leq_73(10)]=1,[day2_arm_2][leq_value_73],0)+ if([day2_arm_2][leq_74(10)]=1,[day2_arm_2][leq_value_74],0)+ if([day2_arm_2][leq_75(10)]=1,[day2_arm_2][leq_value_75],0)+ if([day2_arm_2][leq_76(10)]=1,[day2_arm_2][leq_value_76],0)+ if([day2_arm_2][leq_77(10)]=1,[day2_arm_2][leq_value_77],0)+ if([day2_arm_2][leq_78(10)]=1,[day2_arm_2][leq_value_78],0)+ if([day2_arm_2][leq_79(10)]=1,[day2_arm_2][leq_value_79],0)

**leq_total_events_score**		Total events score	sum([day2_arm_2][leq_value_1],<br>[day2_arm_2][leq_value_2],<br>[day2_arm_2][leq_value_3],<br>[day2_arm_2][leq_value_4],<br>[day2_arm_2][leq_value_5],<br>[day2_arm_2][leq_value_6],<br>[day2_arm_2][leq_value_7],<br>[day2_arm_2][leq_value_8],<br>[day2_arm_2][leq_value_9],<br>[day2_arm_2][leq_value_10],<br>[day2_arm_2][leq_value_11],<br>[day2_arm_2][leq_value_12],<br>[day2_arm_2][leq_value_13],<br>[day2_arm_2][leq_value_14],<br>[day2_arm_2][leq_value_15],<br>[day2_arm_2][leq_value_16],<br>[day2_arm_2][leq_value_17],<br>[day2_arm_2][leq_value_18],<br>[day2_arm_2][leq_value_19],<br>[day2_arm_2][leq_value_20],<br>[day2_arm_2][leq_value_21],<br>[day2_arm_2][leq_value_22],<br>[day2_arm_2][leq_value_23],<br>[day2_arm_2][leq_value_24],<br>[day2_arm_2][leq_value_25],<br>[day2_arm_2][leq_value_26],<br>[day2_arm_2][leq_value_27],<br>[day2_arm_2][leq_value_28],<br>[day2_arm_2][leq_value_29],<br>[day2_arm_2][leq_value_30],<br>[day2_arm_2][leq_value_31],<br>[day2_arm_2][leq_value_32],<br>[day2_arm_2][leq_value_33],<br>[day2_arm_2][leq_value_34],<br>[day2_arm_2][leq_value_35],<br>[day2_arm_2][leq_value_36],<br>[day2_arm_2][leq_value_37],<br>[day2_arm_2][leq_value_38],<br>[day2_arm_2][leq_value_39],<br>[day2_arm_2][leq_value_40],<br>[day2_arm_2][leq_value_41],<br>[day2_arm_2][leq_value_42],<br>[day2_arm_2][leq_value_43],<br>[day2_arm_2][leq_value_44],<br>[day2_arm_2][leq_value_45],

			[day2_arm_2][leq_value_46],<br>[day2_arm_2][leq_value_47],<br>[day2_arm_2][leq_value_48],<br>[day2_arm_2][leq_value_49],<br>[day2_arm_2][leq_value_50],<br>[day2_arm_2][leq_value_51],<br>[day2_arm_2][leq_value_52],<br>[day2_arm_2][leq_value_53],<br>[day2_arm_2][leq_value_54],<br>[day2_arm_2][leq_value_55],<br>[day2_arm_2][leq_value_56],<br>[day2_arm_2][leq_value_57],<br>[day2_arm_2][leq_value_58],<br>[day2_arm_2][leq_value_59],<br>[day2_arm_2][leq_value_60],<br>[day2_arm_2][leq_value_61],<br>[day2_arm_2][leq_value_62],<br>[day2_arm_2][leq_value_63],<br>[day2_arm_2][leq_value_64],<br>[day2_arm_2][leq_value_65],<br>[day2_arm_2][leq_value_66],<br>[day2_arm_2][leq_value_67],<br>[day2_arm_2][leq_value_68],<br>[day2_arm_2][leq_value_69],<br>[day2_arm_2][leq_value_70],<br>[day2_arm_2][leq_value_71],<br>[day2_arm_2][leq_value_72],<br>[day2_arm_2][leq_value_73],<br>[day2_arm_2][leq_value_74],<br>[day2_arm_2][leq_value_75],<br>[day2_arm_2][leq_value_76],<br>[day2_arm_2][leq_value_77],<br>[day2_arm_2][leq_value_78],<br>[day2_arm_2][leq_value_79])
**dsm_5_cross_cutting**	DSM-5	Cross-Cutting Symptom Measure	[day2_arm_2][dsm5_lv1_1]+<br>[day2_arm_2][dsm5_lv1_2]+<br>[day2_arm_2][dsm5_lv1_3]+<br>[day2_arm_2][dsm5_lv1_4]+<br>[day2_arm_2][dsm5_lv1_5]+<br>[day2_arm_2][dsm5_lv1_6]+<br>[day2_arm_2][dsm5_lv1_7]+<br>[day2_arm_2][dsm5_lv1_8]+<br>[day2_arm_2][dsm5_lv1_9]+<br>[day2_arm_2][dsm5_lv1_10]+

			[day2_arm_2][dsm5_lv1_11]+<br>[day2_arm_2][dsm5_lv1_12]+<br>[day2_arm_2][dsm5_lv1_13]+<br>[day2_arm_2][dsm5_lv1_14]+<br>[day2_arm_2][dsm5_lv1_15]+<br>[day2_arm_2][dsm5_lv1_16]+<br>[day2_arm_2][dsm5_lv1_17]+<br>[day2_arm_2][dsm5_lv1_18]+<br>[day2_arm_2][dsm5_lv1_19]+<br>[day2_arm_2][dsm5_lv1_20]+<br>[day2_arm_2][dsm5_lv1_21]+<br>[day2_arm_2][dsm5_lv1_22]+<br>[day2_arm_2][dsm5_lv1_23]
**dsm_5_neg_aff**		Negative Affect	[day2_arm_2][dsm5_pibr_8]+<br>[day2_arm_2][dsm5_pibr_9]+<br>[day2_arm_2][dsm5_pibr_10]+<br>[day2_arm_2][dsm5_pibr_11]+<br>[day2_arm_2][dsm5_pibr_15]
**dsm_5_detachment**		Detachment	[day2_arm_2][dsm5_pibr_4]+<br>[day2_arm_2][dsm5_pibr_13]+<br>[day2_arm_2][dsm5_pibr_14]+<br>[day2_arm_2][dsm5_pibr_16]+<br>[day2_arm_2][dsm5_pibr_18]
**dsm_5_antagonism**		Antagonism	[day2_arm_2][dsm5_pibr_17]+<br>[day2_arm_2][dsm5_pibr_19]+<br>[day2_arm_2][dsm5_pibr_20]+<br>[day2_arm_2][dsm5_pibr_22]+<br>[day2_arm_2][dsm5_pibr_25]
**dsm_5_disinhibition**		Disinhibition	[day2_arm_2][dsm5_pibr_1]+<br>[day2_arm_2][dsm5_pibr_2]+<br>[day2_arm_2][dsm5_pibr_3]+<br>[day2_arm_2][dsm5_pibr_5]+<br>[day2_arm_2][dsm5_pibr_6]

**dsm_5_psychoticism**		psychoticism	[day2_arm_2][dsm5_pibr_7]+<br>[day2_arm_2][dsm5_pibr_12]+<br>[day2_arm_2][dsm5_pibr_21]+<br>[day2_arm_2][dsm5_pibr_23]+<br>[day2_arm_2][dsm5_pibr_24]
**dsm_5_substance**		Substance Use	[day2_arm_2][dsm5_lv2_sa_1]+<br>[day2_arm_2][dsm5_lv2_sa_2]+<br>[day2_arm_2][dsm5_lv2_sa_3]+<br>[day2_arm_2][dsm5_lv2_sa_4]+<br>[day2_arm_2][dsm5_lv2_sa_5]+<br>[day2_arm_2][dsm5_lv2_sa_6]+<br>[day2_arm_2][dsm5_lv2_sa_7]+<br>[day2_arm_2][dsm5_lv2_sa_8]+<br>[day2_arm_2][dsm5_lv2_sa_9]+<br>[day2_arm_2][dsm5_lv2_sa_10]
**dsm_5_somatic**		Somatic Symptom	[day2_arm_2][dsm5_lv2_ss_1]+<br>[day2_arm_2][dsm5_lv2_ss_2]+<br>[day2_arm_2][dsm5_lv2_ss_3]+<br>[day2_arm_2][dsm5_lv2_ss_4]+<br>[day2_arm_2][dsm5_lv2_ss_5]+<br>[day2_arm_2][dsm5_lv2_ss_6]+<br>[day2_arm_2][dsm5_lv2_ss_7]+<br>[day2_arm_2][dsm5_lv2_ss_8]+<br>[day2_arm_2][dsm5_lv2_ss_9]+<br>[day2_arm_2][dsm5_lv2_ss_10]+<br>[day2_arm_2][dsm5_lv2_ss_11]+<br>[day2_arm_2][dsm5_lv2_ss_12]+<br>[day2_arm_2][dsm5_lv2_ss_13]+<br>[day2_arm_2][dsm5_lv2_ss_14]+<br>[day2_arm_2][dsm5_lv2_ss_15]
**dsm_5_sleep_dist**		Sleep Disturbance	[day2_arm_2][dsm5_lv2_sd_1]+<br>[day2_arm_2][dsm5_lv2_sd_2]+<br>[day2_arm_2][dsm5_lv2_sd_3]+<br>[day2_arm_2][dsm5_lv2_sd_4]+<br>[day2_arm_2][dsm5_lv2_sd_5]+<br>[day2_arm_2][dsm5_lv2_sd_6]+<br>[day2_arm_2][dsm5_lv2_sd_7]+<br>[day2_arm_2][dsm5_lv2_sd_8]

**dsm_5_repetitiv**		Repetitive Thoughts and Behaviors	[day2_arm_2][dsm5_lv2_rtb_1]+<br>[day2_arm_2][dsm5_lv2_rtb_2]+<br>[day2_arm_2][dsm5_lv2_rtb_3]+<br>[day2_arm_2][dsm5_lv2_rtb_4]+<br>[day2_arm_2][dsm5_lv2_rtb_5]
**dsm_5_mania**		Mania	[day2_arm_2][dsm5_lv2_mania_1]+<br>[day2_arm_2][dsm5_lv2_mania_2]+<br>[day2_arm_2][dsm5_lv2_mania_3]+<br>[day2_arm_2][dsm5_lv2_mania_4]+<br>[day2_arm_2][dsm5_lv2_mania_5]
**dsm_5_depre**		Depression	[day2_arm_2][dsm5_lv2_depr_1]+<br>[day2_arm_2][dsm5_lv2_depr_2]+<br>[day2_arm_2][dsm5_lv2_depr_3]+<br>[day2_arm_2][dsm5_lv2_depr_4]+<br>[day2_arm_2][dsm5_lv2_depr_5]+<br>[day2_arm_2][dsm5_lv2_depr_6]+<br>[day2_arm_2][dsm5_lv2_depr_7]+<br>[day2_arm_2][dsm5_lv2_depr_8]
**dsm_5_anx**		Anxiety	[day2_arm_2][dsm5_lv2_anx_1]+<br>[day2_arm_2][dsm5_lv2_anx_2]+<br>[day2_arm_2][dsm5_lv2_anx_3]+<br>[day2_arm_2][dsm5_lv2_anx_4]+<br>[day2_arm_2][dsm5_lv2_anx_5]+<br>[day2_arm_2][dsm5_lv2_anx_6]+<br>[day2_arm_2][dsm5_lv2_anx_7]
**dsm_5_anger**		Anger	[day2_arm_2][dsm5_lv2_angr_1]+<br>[day2_arm_2][dsm5_lv2_angr_2]+<br>[day2_arm_2][dsm5_lv2_angr_3]+<br>[day2_arm_2][dsm5_lv2_angr_4]+<br>[day2_arm_2][dsm5_lv2_angr_5]
**vigorous_work_total_min**		Total minutes for ipaq_3_total_min	if(sum([ipaq_3_h]*60, [ipaq_3_m])>180,180,sum([ipaq_3_h]*60, [ipaq_3_m]))
**moderate_work_total_min**		Total minutes for ipaq_5_total_min	if(sum([ipaq_5_h]*60, [ipaq_5_m])>180,180,sum([ipaq_5_h]*60, [ipaq_5_m]))

**walking_work_total_min**		Total minutes for ipaq_7_total_min	if(sum([ipaq_7_h]*60, [ipaq_7_m])>180,180,sum([ipaq_7_h]*60, [ipaq_7_m]))
**total_work_min**		Total minutes for ipaq_7_total_min	sum([vigorous_work_total_min],[moderate_work_total_min],[walking_work_total_min])
**motor_travel_total_min**		Total minutes for ipaq_9_m_total_min	if(sum([ipaq_9]*60, [ipaq_9_m]) > 180, 180, sum([ipaq_9]*60, [ipaq_9_m]))
**cycle__travel_total_min**		Total minutes for ipaq_11_total_min	if(sum([ipaq_11]*60, [ipaq_11_m]) > 180, 180, sum([ipaq_11]*60, [ipaq_11_m]))
**walking_travel_total_min**		Total minutes for ipaq_13_total_min	if(sum([ipaq_13]*60, [ipaq_13_m]) > 180, 180, sum([ipaq_13]*60, [ipaq_13_m]))
**vigorous_yard_total_min**		Total minutes for ipaq_15_total_min	if(sum([ipaq_15]*60, [ipaq_15_m]) > 180, 180, sum([ipaq_15]*60, [ipaq_15_m]))
**moderate_yard_total_min**		Total minutes for ipaq_17_total_min	if(sum([ipaq_17]*60, [ipaq_17_m]) > 180, 180, sum([ipaq_17]*60, [ipaq_17_m]))
**moderate_home_total_min**		Total minutes for ipaq_19_total_min	if(sum([ipaq_19]*60, [ipaq_19_m]) > 180, 180, sum([ipaq_19]*60, [ipaq_19_m]))
**walking_leisure_total_min**		Total minutes for ipaq_21_total_min	if(sum([ipaq_21]*60, [ipaq_21_m]) > 180, 180, sum([ipaq_21]*60, [ipaq_21_m]))
**vigorous_leisure_total_min**		Total minutes for ipaq_23_total_min	if(sum([ipaq_23]*60, [ipaq_23_m]) > 180, 180, sum([ipaq_23]*60, [ipaq_23_m]))
**moderate_leisure_total_min**		Total minutes for ipaq_25_total_min	if(sum([ipaq_25]*60, [ipaq_25_m]) > 180, 180, sum([ipaq_25]*60, [ipaq_25_m]))
**sitting_leisure_total_min**		Total minutes for ipaq_27_total_min	sum([ipaq_27]*60, [ipaq_27_m], [ipaq_26]*60, [ipaq_26_m]*7)

**vigorous_work_total_min_week**		Total MET for vigorous physical activities at work	[vigorous_work_total_min]*[ipaq_2]*8
**moderate_work_total_min_week**		Total MET for moderate physical activities at work	[moderate_work_total_min]*[ipaq_4]*4
**walking_work_total_min_week**		Total MET for walking at work	[walking_work_total_min]*[ipaq_6]*3.3
**total_work_min_week**		Total MET total work	sum([vigorous_work_total_min_week],[moderate_work_total_min_week],[walking_work_total_min_week])
**motor_travel_total_min_week**		Total minutes for traveling in a train, bus, car, tram, or other kind of motor vehicle	[motor_travel_total_min]*[a]*2
**cycle__travel_total_min_week**		Total MET for bicycle	[cycle__travel_total_min]*[ipaq_10]*6
**walking_travel_total_min_week**		Total MET for walk	[walking_leisure_total_min]*[ipaq_12]*3.3
**vigorous_yard_total_min_week**		Total MET for vigorous physical activities in the yard	[vigorous_leisure_total_min]*[ipaq_14]*5.5
**moderate_yard_total_min_week**		Total MET for moderate physical activities in the yard	[moderate_yard_total_min]*[ipaq_16]*4
**moderate_home_total_min_week**		Total MET for moderate physical activities at home	[moderate_home_total_min]*[ipaq_18]*3
**walking_leisure_total_min_week**		Total MET for walking leisure time	[walking_leisure_total_min]*[ipaq_20]*3.3

**vigorous_leisure_total_min_week**		Total MET for vigorous physical activities at leisure	[vigorous_leisure_total_min]*[ipaq_22]*8
**moderate_leisure_total_min_week**		Total MET for moderate physical activities at leisure	[moderate_leisure_total_min]*[ipaq_24]*4
**sitting_leisure_total_min_week**		Total Minutes for sitting	[sitting_leisure_total_min]
**total_met_transporation**		Total MET-minutes/week for transportation	sum([cycle__travel_total_min_week],[walking_travel_total_min_week])
**total_met_demostic_garden**		Total MET-minutes/week from domestic and garden	sum([vigorous_yard_total_min_week],[moderate_yard_total_min_week],[moderate_home_total_min_week])
**total_met_leisure**		Total MET-minutes/week in leisure-time	sum([walking_leisure_total_min_week],[vigorous_leisure_total_min_week],[moderate_leisure_total_min_week],[sitting_leisure_total_min_week])
**total_met_walking**		Total walking	sum([walking_travel_total_min_week],[walking_leisure_total_min_week])
**total_met_mod**		Total moderate	sum([moderate_yard_total_min_week],[moderate_home_total_min_week],[moderate_leisure_total_min_week])
**total_met_vigorous**		Total Vigorous	sum([vigorous_yard_total_min_week],[vigorous_leisure_total_min_week])
**ipaq_total_met**		Total Physical Activity Score	sum([total_work_min_week],[motor_travel_total_min_week],[cycle__travel_total_min_week],[walking_travel_total_min_week],[vigorous_yard_total_min_week],[moderate_yard_total_min_week],[moderate_home_total_min_week],[walking_leisure_total_min_week],[vigorous_leisure_total_min_week],[moderate_leisure_total_min_week],[sitting_leisure_total_min_week])

2.5 Home

2.5.1 Home Logbook

Redcap Form Name: Home Logbook

Questionnaire Name	Title	Variable Name
2.4.1.1 Modified Differential Emotions Scale	Modified Differential Emotions Scale (mDES)	mdes
2.4.1.2 My Daily Stress	Daily Inventory of Stressful Events (DISE)	dise

Variable / Field Name	Form Name	Section Header	Field Type	Field Label	Choices, Calculations, OR Slider Labels
**saliva_s2_colltime_h1**	home_logbook		text	Time for sample # 2	
**saliva_s3_colltime_h1**	home_logbook		text	Time for sample # 3	
**saliva_s4_colltime_h1**	home_logbook		text	Time for sample # 4	
**saliva_s2_eatdrink_h1**	home_logbook		text	Time Eat or Drink before Sample # 2	
**saliva_s3_eatdrink_h1**	home_logbook		text	Time Eat or Drink before Sample # 3	
**hl_bedtime_h1**	home_logbook		text	What time did you try to go to sleep last night (lights out)?	
**hl_waketime_h1**	home_logbook		text	What time did you wake up today?	
**hl_sleepquality_h1**	home_logbook		radio	How would you rate the quality of your sleep last night?	1, Very bad | 2, Fairly bad | 3, Fairly good | 4, Very good
**hl_dayprospects_h1**	home_logbook		slider	Please indicate by marking an X on the line below to what extent you are looking forward to versus dreading today's events.	Really dreading today's events | Neutral | Really looking forward to today's events
**hl_dayworried_h1**	home_logbook		slider	Please indicate by marking an X on the line below to what extent you are	At ease | Neutral | Worrying a lot

				worrying about how things are going to go today.	
**hl_daypredictable_h1**	home_logbook		slider	Please indicate by marking an X on the line below to what extent you feel the events of your upcoming day re predictable (you know exactly what's going to appen today).	Completely Predictable | Completely Unpredictable
**hl_stressed_h1**	home_logbook		radio	I feel stressed, anxious, overwhelmed	0, Not at all | 1, A little bit | 2, Somewhat | 3, Moderately | 4, Extremely
**hl_control_h1**	home_logbook		radio	I feel in control, coping well, on top of things	0, Not at all | 1, A little bit | 2, Somewhat | 3, Moderately | 4, Extremely
**hl_joyful_h1**	home_logbook		radio	I feel joyful, glad, happy	0, Not at all | 1, A little bit | 2, Somewhat | 3, Moderately | 4, Extremely
**hl_sleepdisruption_h1**	home_logbook		text	Did anything disturb your sleep last night?	
**mdes_amused_h1**	home_logbook		radio	1. I felt amused, fun-loving, or silly.	0, Not at all | 1, A little bit | 2, Moderately | 3 Quite a bit | 4, Extremely
**mdes_angry_h1**	home_logbook		radio	2. I felt angry, irritated, or annoyed.	0, Not at all | 1, A little bit | 2, Moderately | 3 Quite a bit | 4, Extremely
**mdes_ashamed_h1**	home_logbook		radio	3. I felt ashamed, humiliated, or disgraced.	0, Not at all | 1, A little bit | 2, Moderately | 3 Quite a bit | 4, Extremely
**mdes_awe_h1**	home_logbook		radio	4. I felt awe, wonder, or amazement.	0, Not at all | 1, A little bit | 2, Moderately | 3 Quite a bit | 4, Extremely
**mdes_bored_h1**	home_logbook		radio	5. I felt bored, disinterested, uninvolved.	0, Not at all | 1, A little bit | 2, Moderately | 3 Quite a bit | 4, Extremely

**mdes_contemptuous_h1**	home_logbook		radio	6. I felt contemptuous, scornful, or disdainful.	0, Not at all | 1, A little bit | 2, Moderately | 3, Quite a bit | 4, Extremely
**mdes_control_h1**	home_logbook		radio	7. I felt control, coping well, on top of things.	0, Not at all | 1, A little bit | 2, Moderately | 3, Quite a bit | 4, Extremely
**mdes_disgust_h1**	home_logbook		radio	8. I felt disgust, distaste, or revulsion.	0, Not at all | 1, A little bit | 2, Moderately | 3, Quite a bit | 4, Extremely
**mdes_embarrassed_h1**	home_logbook		radio	9. I felt embarrassed, self-conscious, or blushing.	0, Not at all | 1, A little bit | 2, Moderately | 3, Quite a bit | 4, Extremely
**mdes_excited_h1**	home_logbook		radio	10. I felt excited, eager, enthusiastic.	0, Not at all | 1, A little bit | 2, Moderately | 3, Quite a bit | 4, Extremely
**mdes_grateful_h1**	home_logbook		radio	11. I felt grateful, appreciative, or thankful.	0, Not at all | 1, A little bit | 2, Moderately | 3, Quite a bit | 4, Extremely
**mdes_guilty_h1**	home_logbook		radio	12. I felt guilty, repentant, or blameworthy.	0, Not at all | 1, A little bit | 2, Moderately | 3, Quite a bit | 4, Extremely
**mdes_hatred_h1**	home_logbook		radio	13. I felt hatred, distrust, or suspicion.	0, Not at all | 1, A little bit | 2, Moderately | 3, Quite a bit | 4, Extremely
**mdes_hopeful_h1**	home_logbook		radio	14. I felt hopeful, optimistic, or encouraged.	0, Not at all | 1, A little bit | 2, Moderately | 3, Quite a bit | 4, Extremely
**mdes_inspired_h1**	home_logbook		radio	15. I felt inspired, uplifted, or elevated.	0, Not at all | 1, A little bit | 2, Moderately | 3, Quite a bit | 4, Extremely
**mdes_interested_h1**	home_logbook		radio	16. I felt interested, alert, or curious.	0, Not at all | 1, A little bit | 2, Moderately | 3, Quite a bit | 4, Extremely
**mdes_joyful_h1**	home_logbook		radio	17. I felt joyful, glad, or happy.	0, Not at all | 1, A little bit | 2, Moderately | 3, Quite a bit | 4, Extremely

<table>
<tr><th></th><th></th><th></th><th></th><th></th><th></th></tr>
<tr><td>mdes_love_h1</td><td>home_logbook</td><td></td><td>radio</td><td>18. I felt love, closeness, or trust.</td><td>0, Not at all | 1, A little bit | 2, Moderately | 3, Quite a bit | 4, Extremely</td></tr>
<tr><td>mdes_proud_h1</td><td>home_logbook</td><td></td><td>radio</td><td>19. I felt proud, confident, or self-assured.</td><td>0, Not at all | 1, A little bit | 2, Moderately | 3, Quite a bit | 4, Extremely</td></tr>
<tr><td>mdes_rejected_h1</td><td>home_logbook</td><td></td><td>radio</td><td>20. I felt rejected, betrayed, left-behind.</td><td>0, Not at all | 1, A little bit | 2, Moderately | 3, Quite a bit | 4, Extremely</td></tr>
<tr><td>mdes_sad_h1</td><td>home_logbook</td><td></td><td>radio</td><td>21. I felt sad, downhearted, or unhappy.</td><td>0, Not at all | 1, A little bit | 2, Moderately | 3, Quite a bit | 4, Extremely</td></tr>
<tr><td>mdes_satisfied_h1</td><td>home_logbook</td><td></td><td>radio</td><td>22. I felt satisfied, fulfilled, content.</td><td>0, Not at all | 1, A little bit | 2, Moderately | 3, Quite a bit | 4, Extremely</td></tr>
<tr><td>mdes_scared_h1</td><td>home_logbook</td><td></td><td>radio</td><td>23. I felt scared, fearful, or afraid.</td><td>0, Not at all | 1, A little bit | 2, Moderately | 3, Quite a bit | 4, Extremely</td></tr>
<tr><td>mdes_stressed_h1</td><td>home_logbook</td><td></td><td>radio</td><td>24. I felt stressed, nervous, or overwhelmed.</td><td>0, Not at all | 1, A little bit | 2, Moderately | 3, Quite a bit | 4, Extremely</td></tr>
<tr><td>mdes_tired_h1</td><td>home_logbook</td><td></td><td>radio</td><td>25. I felt tired, sleepy, drowsy.</td><td>0, Not at all | 1, A little bit | 2, Moderately | 3, Quite a bit | 4, Extremely</td></tr>
<tr><td>mdes_sexual_h1</td><td>home_logbook</td><td></td><td>radio</td><td>26. I felt sexual, desiring, flirtatious.</td><td>0, Not at all | 1, A little bit | 2, Moderately | 3, Quite a bit | 4, Extremely</td></tr>
<tr><td>mdes_sympathy_h1</td><td>home_logbook</td><td></td><td>radio</td><td>27. I felt sympathy, concern, compassion.</td><td>0, Not at all | 1, A little bit | 2, Moderately | 3, Quite a bit | 4, Extremely</td></tr>
<tr><td>hl_bedtime_h2</td><td>home_logbook</td><td>day 2</td><td>text</td><td>What time did you try to go to sleep last night (lights out)?</td><td></td></tr>
<tr><td>hl_waketime_h2</td><td>home_logbook</td><td></td><td>text</td><td>What time did you wake up today?</td><td></td></tr>
</table>

**hl_sleepquality_h2**	home_logbook		radio	How would you rate the quality of your sleep last night?	1, Very bad | 2, Fairly bad | 3, Fairly good | 4, Very good
**hl_dayprospects_h2**	home_logbook		slider	Please indicate by marking an X on the line below to what extent you are looking forward to versus dreading today's events.	Really dreading Today's Event | Neutral | Really looking forward to today's events
**hl_dayworried_h2**	home_logbook		slider	Please indicate by marking an X on the line below to hat extent you are worrying about how things are going to go today.	At ease | Neutral | Worrying a lot
**hl_daypredictable_h2**	home_logbook		slider	Please indicate by marking an X on the line below to what extent you feel the events of your upcoming day re predictable (you know exactly what's going to appen today).	Completely Predictable | Completely Unpredictable
**hl_stressed_h2**	home_logbook		radio	I feel stressed, anxious, overwhelmed	0, Not at all | 1, A little bit | 2, Somewhat | 3, Moderately | 4, Extremely
**hl_control_h2**	home_logbook		radio	I feel in control, coping well, on top of things	0, Not at all | 1, A little bit | 2, Somewhat | 3, Moderately | 4, Extremely
**hl_joyful_h2**	home_logbook		radio	I feel joyful, glad, happy	0, Not at all | 1, A little bit | 2, Somewhat | 3, Moderately | 4, Extremely
**hl_sleepdisruption_h2**	home_logbook		text	Did anything disturb your sleep last night?	
**mdes_amused_h2**	home_logbook		radio	1. I felt amused, fun-loving, or silly.	0, Not at all | 1, A little bit | 2, Moderately | 3, Quite a bit | 4, Extremely
**mdes_angry_h2**	home_logbook		radio	2. I felt angry, irritated, or annoyed.	0, Not at all | 1, A little bit | 2, Moderately | 3, Quite a bit | 4, Extremely

**mdes_ashamed_h2**	home_logbook		radio	3. I felt ashamed, humiliated, or disgraced.	0, Not at all | 1, A little bit | 2, Moderately | 3 Quite a bit | 4, Extremely
**mdes_awe_h2**	home_logbook		radio	4. I felt awe, wonder, or amazement.	0, Not at all | 1, A little bit | 2, Moderately | 3 Quite a bit | 4, Extremely
**mdes_bored_h2**	home_logbook		radio	5. I felt bored, disinterested, uninvolved.	0, Not at all | 1, A little bit | 2, Moderately | 3 Quite a bit | 4, Extremely
**mdes_contemptuous_h2**	home_logbook		radio	6. I felt contemptuous, scornful, or disdainful.	0, Not at all | 1, A little bit | 2, Moderately | 3 Quite a bit | 4, Extremely
**mdes_control_h2**	home_logbook		radio	7. I felt control, coping well, on top of things.	0, Not at all | 1, A little bit | 2, Moderately | 3 Quite a bit | 4, Extremely
**mdes_disgust_h2**	home_logbook		radio	8. I felt disgust, distaste, or revulsion.	0, Not at all | 1, A little bit | 2, Moderately | 3 Quite a bit | 4, Extremely
**mdes_embarrassed_h2**	home_logbook		radio	9. I felt embarrassed, self-conscious, or blushing.	0, Not at all | 1, A little bit | 2, Moderately | 3 Quite a bit | 4, Extremely
**mdes_excited_h2**	home_logbook		radio	10. I felt excited, eager, enthusiastic.	0, Not at all | 1, A little bit | 2, Moderately | 3 Quite a bit | 4, Extremely
**mdes_grateful_h2**	home_logbook		radio	11. I felt grateful, appreciative, or thankful.	0, Not at all | 1, A little bit | 2, Moderately | 3 Quite a bit | 4, Extremely
**mdes_guilty_h2**	home_logbook		radio	12. I felt guilty, repentant, or blameworthy.	0, Not at all | 1, A little bit | 2, Moderately | 3 Quite a bit | 4, Extremely
**mdes_hatred_h2**	home_logbook		radio	13. I felt hatred, distrust, or suspicion.	0, Not at all | 1, A little bit | 2, Moderately | 3 Quite a bit | 4, Extremely
**mdes_hopeful_h2**	home_logbook		radio	14. I felt hopeful, optimistic, or encouraged.	0, Not at all | 1, A little bit | 2, Moderately | 3 Quite a bit | 4, Extremely

**mdes_inspired_h2**	home_logbook		radio	15. I felt inspired, uplifted, or elevated.	0, Not at all | 1, A little bit | 2, Moderately | 3, Quite a bit | 4, Extremely
**mdes_interested_h2**	home_logbook		radio	16. I felt interested, alert, or curious.	0, Not at all | 1, A little bit | 2, Moderately | 3, Quite a bit | 4, Extremely
**mdes_joyful_h2**	home_logbook		radio	17. I felt joyful, glad, or happy.	0, Not at all | 1, A little bit | 2, Moderately | 3, Quite a bit | 4, Extremely
**mdes_love_h2**	home_logbook		radio	18. I felt love, closeness, or trust.	0, Not at all | 1, A little bit | 2, Moderately | 3, Quite a bit | 4, Extremely
**mdes_proud_h2**	home_logbook		radio	19. I felt proud, confident, or self-assured.	0, Not at all | 1, A little bit | 2, Moderately | 3, Quite a bit | 4, Extremely
**mdes_rejected_h2**	home_logbook		radio	20. I felt rejected, betrayed, left-behind.	0, Not at all | 1, A little bit | 2, Moderately | 3, Quite a bit | 4, Extremely
**mdes_sad_h2**	home_logbook		radio	21. I felt sad, downhearted, or unhappy.	0, Not at all | 1, A little bit | 2, Moderately | 3, Quite a bit | 4, Extremely
**mdes_satisfied_h2**	home_logbook		radio	22. I felt satisfied, fulfilled, content.	0, Not at all | 1, A little bit | 2, Moderately | 3, Quite a bit | 4, Extremely
**mdes_scared_h2**	home_logbook		radio	23. I felt scared, fearful, or afraid.	0, Not at all | 1, A little bit | 2, Moderately | 3, Quite a bit | 4, Extremely
**mdes_stressed_h2**	home_logbook		radio	24. I felt stressed, nervous, or overwhelmed.	0, Not at all | 1, A little bit | 2, Moderately | 3, Quite a bit | 4, Extremely
**mdes_tired_h2**	home_logbook		radio	25. I felt tired, sleepy, drowsy.	0, Not at all | 1, A little bit | 2, Moderately | 3, Quite a bit | 4, Extremely
**mdes_sexual_h2**	home_logbook		radio	26. I felt sexual, desiring, flirtatious.	0, Not at all | 1, A little bit | 2, Moderately | 3, Quite a bit | 4, Extremely

**mdes_sympathy_h2**	home_logbook		radio	27. I felt sympathy, concern, compassion.	0, Not at all | 1, A little bit | 2, Moderately | 3, Quite a bit | 4, Extremely
**saliva_s1colltime_h3**	home_logbook	day 3	text	Time for sample # 1	
**saliva_s2_colltime_h3**	home_logbook		text	Time for sample # 2	
**saliva_s3_colltime_h3**	home_logbook		text	Time for sample # 3	
**saliva_s4_colltime_h3**	home_logbook		text	Time for sample # 4	
**saliva_s2_eatdrink_h3**	home_logbook		text	Time Eat or Drink before Sample # 2	
**saliva_s3_eatdrink_h3**	home_logbook		text	Time Eat or Drink before Sample # 3	
**hl_bedtime_h3**	home_logbook		text	What time did you try to go to sleep last night (lights out)?	
**hl_waketime_h3**	home_logbook		text	What time did you wake up today?	
**hl_sleepquality_h3**	home_logbook		radio	How would you rate the quality of your sleep last night?	1, Very bad | 2, Fairly bad | 3, Fairly good | 4, Very good
**hl_dayprospects_h3**	home_logbook		slider	Please indicate by marking an X on the line below to what extent you are looking forward to versus dreading today's events.	Really dreading today's events | Neutral | Really looking forward to today's events
**hl_dayworried_h3**	home_logbook		slider	Please indicate by marking an X on the line below to hat extent you are worrying about how things are going to go today.	At ease | Neutral | Worrying a lot
**hl_daypredictable_h3**	home_logbook		slider	Please indicate by marking an X on the line below to what extent you feel the events of your upcoming day re	Completely Predictable | Completely Unpredictable

<table>
<tr><td></td><td></td><td></td><td></td><td>predictable (you know exactly what's going to appen today).</td><td></td></tr>
<tr><td>hl_stressed_h3</td><td>home_logbook</td><td></td><td>radio</td><td>I feel stressed, anxious, overwhelmed</td><td>0, Not at all | 1, A little bit | 2, Somewhat | 3, Moderately | 4, Extremely</td></tr>
<tr><td>hl_control_h3</td><td>home_logbook</td><td></td><td>radio</td><td>I feel in control, coping well, on top of things</td><td>0, Not at all | 1, A little bit | 2, Somewhat | 3, Moderately | 4, Extremely</td></tr>
<tr><td>hl_joyful_h3</td><td>home_logbook</td><td></td><td>radio</td><td>I feel joyful, glad, happy</td><td>0,, Not at all | 1, A little bit | 2, Somewhat | 3, Moderately | 4, Extremely</td></tr>
<tr><td>hl_sleepdisruption_h3</td><td>home_logbook</td><td></td><td>text</td><td>Did anything disturb your sleep last night?</td><td></td></tr>
<tr><td>mdes_amused_h3</td><td>home_logbook</td><td></td><td>radio</td><td>1. I felt amused, fun-loving, or silly.</td><td>0, Not at all | 1, A little bit | 2, Moderately | 3 Quite a bit | 4, Extremely</td></tr>
<tr><td>mdes_angry_h3</td><td>home_logbook</td><td></td><td>radio</td><td>2. I felt angry, irritated, or annoyed.</td><td>0, Not at all | 1, A little bit | 2, Moderately | 3 Quite a bit | 4, Extremely</td></tr>
<tr><td>mdes_ashamed_h3</td><td>home_logbook</td><td></td><td>radio</td><td>3. I felt ashamed, humiliated, or disgraced.</td><td>0, Not at all | 1, A little bit | 2, Moderately | 3 Quite a bit | 4, Extremely</td></tr>
<tr><td>mdes_awe_h3</td><td>home_logbook</td><td></td><td>radio</td><td>4. I felt awe, wonder, or amazement.</td><td>0, Not at all | 1, A little bit | 2, Moderately | 3 Quite a bit | 4, Extremely</td></tr>
<tr><td>mdes_bored_h3</td><td>home_logbook</td><td></td><td>radio</td><td>5. I felt bored, disinterested, uninvolved.</td><td>0, Not at all | 1, A little bit | 2, Moderately | 3 Quite a bit | 4, Extremely</td></tr>
<tr><td>mdes_contemptuous_h3</td><td>home_logbook</td><td></td><td>radio</td><td>6. I felt contemptuous, scornful, or disdainful.</td><td>0, Not at all | 1, A little bit | 2, Moderately | 3 Quite a bit | 4, Extremely</td></tr>
<tr><td>mdes_control_h3</td><td>home_logbook</td><td></td><td>radio</td><td>7. I felt control, coping well, on top of things.</td><td>0, Not at all | 1, A little bit | 2, Moderately | 3 Quite a bit | 4, Extremely</td></tr>
</table>

**mdes_disgust_h3**	home_logbook		radio	8. I felt disgust, distaste, or revulsion.	0, Not at all | 1, A little bit | 2, Moderately | 3, Quite a bit | 4, Extremely
**mdes_embarrassed_h3**	home_logbook		radio	9. I felt embarrassed, self-conscious, or blushing.	0, Not at all | 1, A little bit | 2, Moderately | 3, Quite a bit | 4, Extremely
**mdes_excited_h3**	home_logbook		radio	10. I felt excited, eager, enthusiastic.	0, Not at all | 1, A little bit | 2, Moderately | 3, Quite a bit | 4, Extremely
**mdes_grateful_h3**	home_logbook		radio	11. I felt grateful, appreciative, or thankful.	0, Not at all | 1, A little bit | 2, Moderately | 3, Quite a bit | 4, Extremely
**mdes_guilty_h3**	home_logbook		radio	12. I felt guilty, repentant, or blameworthy.	0, Not at all | 1, A little bit | 2, Moderately | 3, Quite a bit | 4, Extremely
**mdes_hatred_h3**	home_logbook		radio	13. I felt hatred, distrust, or suspicion.	0, Not at all | 1, A little bit | 2, Moderately | 3, Quite a bit | 4, Extremely
**mdes_hopeful_h3**	home_logbook		radio	14. I felt hopeful, optimistic, or encouraged.	0, Not at all | 1, A little bit | 2, Moderately | 3, Quite a bit | 4, Extremely
**mdes_inspired_h3**	home_logbook		radio	15. I felt inspired, uplifted, or elevated.	0, Not at all | 1, A little bit | 2, Moderately | 3, Quite a bit | 4, Extremely
**mdes_interested_h3**	home_logbook		radio	16. I felt interested, alert, or curious.	0, Not at all | 1, A little bit | 2, Moderately | 3, Quite a bit | 4, Extremely
**mdes_joyful_h3**	home_logbook		radio	17. I felt joyful, glad, or happy.	0, Not at all | 1, A little bit | 2, Moderately | 3, Quite a bit | 4, Extremely
**mdes_love_h3**	home_logbook		radio	18. I felt love, closeness, or trust.	0, Not at all | 1, A little bit | 2, Moderately | 3, Quite a bit | 4, Extremely
**mdes_proud_h3**	home_logbook		radio	19. I felt proud, confident, or self-assured.	0, Not at all | 1, A little bit | 2, Moderately | 3, Quite a bit | 4, Extremely

**mdes_rejected_h3**	home_logbook		radio	20. I felt rejected, betrayed, left-behind.	0, Not at all | 1, A little bit | 2, Moderately | 3, Quite a bit | 4, Extremely
**mdes_sad_h3**	home_logbook		radio	21. I felt sad, downhearted, or unhappy.	0, Not at all | 1, A little bit | 2, Moderately | 3, Quite a bit | 4, Extremely
**mdes_satisfied_h3**	home_logbook		radio	22. I felt satisfied, fulfilled, content.	0, Not at all | 1, A little bit | 2, Moderately | 3, Quite a bit | 4, Extremely
**mdes_scared_h3**	home_logbook		radio	23. I felt scared, fearful, or afraid.	0, Not at all | 1, A little bit | 2, Moderately | 3, Quite a bit | 4, Extremely
**mdes_stressed_h3**	home_logbook		radio	24. I felt stressed, nervous, or overwhelmed.	0, Not at all | 1, A little bit | 2, Moderately | 3, Quite a bit | 4, Extremely
**mdes_tired_h3**	home_logbook		radio	25. I felt tired, sleepy, drowsy.	0, Not at all | 1, A little bit | 2, Moderately | 3, Quite a bit | 4, Extremely
**mdes_sexual_h3**	home_logbook		radio	26. I felt sexual, desiring, flirtatious.	0, Not at all | 1, A little bit | 2, Moderately | 3, Quite a bit | 4, Extremely
**mdes_sympathy_h3**	home_logbook		radio	27. I felt sympathy, concern, compassion.	0, Not at all | 1, A little bit | 2, Moderately | 3, Quite a bit | 4, Extremely
**hl_bedtime_h4**	home_logbook	day 4	text	What time did you try to go to sleep last night (lights out)?	
**hl_waketime_h4**	home_logbook		text	What time did you wake up today?	
**hl_sleepquality_h4**	home_logbook		radio	How would you rate the quality of your sleep last night?	1, Very bad | 2, Fairly bad | 3, Fairly good | 4, Very good
**hl_dayprospects_h4**	home_logbook		slider	Please indicate by marking an X on the line below to what extent you are looking forward to versus dreading today's events.	Really dreading Today's Event | Neutral | Really looking forward to today's events

**hl_dayworried_h4**	home_logbook		slider	Please indicate by marking an X on the line below to hat extent you are worrying about how things are going to go today.	At ease | Neutral | Worrying a lot
**hl_daypredictable_4**	home_logbook		slider	Please indicate by marking an X on the line below to what extent you feel the events of your upcoming day re predictable (you know exactly what's going to appen today).	Completely Predictable | Completely Unpredictable
**hl_stressed_h4**	home_logbook		radio	I feel stressed, anxious, overwhelmed	0, Not at all | 1, A little bit | 2, Somewhat | 3, Moderately | 4, Extremely
**hl_control_h4**	home_logbook		radio	I feel in control, coping well, on top of things	0, Not at all | 1, A little bit | 2, Somewhat | 3, Moderately | 4, Extremely
**hl_joyful_h4**	home_logbook		radio	I feel joyful, glad, happy	0, Not at all | 1, A little bit | 2, Somewhat | 3, Moderately | 4, Extremely
**hl_sleepdisruption_h4**	home_logbook		text	Did anything disturb your sleep last night?	
**mdes_amused_h4**	home_logbook		radio	1. I felt amused, fun-loving, or silly.	0, Not at all | 1, A little bit | 2, Moderately | 3, Quite a bit | 4, Extremely
**mdes_angry_h4**	home_logbook		radio	2. I felt angry, irritated, or annoyed.	0, Not at all | 1, A little bit | 2, Moderately | 3, Quite a bit | 4, Extremely
**mdes_ashamed_h4**	home_logbook		radio	3. I felt ashamed, humiliated, or disgraced.	0, Not at all | 1, A little bit | 2, Moderately | 3, Quite a bit | 4, Extremely
**mdes_awe_h4**	home_logbook		radio	4. I felt awe, wonder, or amazement.	0, Not at all | 1, A little bit | 2, Moderately | 3, Quite a bit | 4, Extremely
**mdes_bored_h4**	home_logbook		radio	5. I felt bored, disinterested, uninvolved.	0, Not at all | 1, A little bit | 2, Moderately | 3, Quite a bit | 4, Extremely

**mdes_contemptuous_h4**	home_logbook		radio	6. I felt contemptuous, scornful, or disdainful.	0, Not at all | 1, A little bit | 2, Moderately | 3 Quite a bit | 4, Extremely
**mdes_control_h4**	home_logbook		radio	7. I felt control, coping well, on top of things.	0, Not at all | 1, A little bit | 2, Moderately | 3 Quite a bit | 4, Extremely
**mdes_disgust_h4**	home_logbook		radio	8. I felt disgust, distaste, or revulsion.	0, Not at all | 1, A little bit | 2, Moderately | 3 Quite a bit | 4, Extremely
**mdes_embarrassed_h4**	home_logbook		radio	9. I felt embarrassed, self-conscious, or blushing.	0, Not at all | 1, A little bit | 2, Moderately | 3 Quite a bit | 4, Extremely
**mdes_excited_h4**	home_logbook		radio	10. I felt excited, eager, enthusiastic.	0, Not at all | 1, A little bit | 2, Moderately | 3 Quite a bit | 4, Extremely
**mdes_grateful_h4**	home_logbook		radio	11. I felt grateful, appreciative, or thankful.	0, Not at all | 1, A little bit | 2, Moderately | 3 Quite a bit | 4, Extremely
**mdes_guilty_h4**	home_logbook		radio	12. I felt guilty, repentant, or blameworthy.	0, Not at all | 1, A little bit | 2, Moderately | 3 Quite a bit | 4, Extremely
**mdes_hatred_h4**	home_logbook		radio	13. I felt hatred, distrust, or suspicion.	0, Not at all | 1, A little bit | 2, Moderately | 3 Quite a bit | 4, Extremely
**mdes_hopeful_h4**	home_logbook		radio	14. I felt hopeful, optimistic, or encouraged.	0, Not at all | 1, A little bit | 2, Moderately | 3 Quite a bit | 4, Extremely
**mdes_inspired_h4**	home_logbook		radio	15. I felt inspired, uplifted, or elevated.	0, Not at all | 1, A little bit | 2, Moderately | 3 Quite a bit | 4, Extremely
**mdes_interested_h4**	home_logbook		radio	16. I felt interested, alert, or curious.	0, Not at all | 1, A little bit | 2, Moderately | 3 Quite a bit | 4, Extremely
**mdes_joyful_h4**	home_logbook		radio	17. I felt joyful, glad, or happy.	0, Not at all | 1, A little bit | 2, Moderately | 3 Quite a bit | 4, Extremely

**mdes_love_h4**	home_logbook		radio	18. I felt love, closeness, or trust.	0, Not at all | 1, A little bit | 2, Moderately | 3, Quite a bit | 4, Extremely
**mdes_proud_h4**	home_logbook		radio	19. I felt proud, confident, or self-assured.	0, Not at all | 1, A little bit | 2, Moderately | 3, Quite a bit | 4, Extremely
**mdes_rejected_h4**	home_logbook		radio	20. I felt rejected, betrayed, left-behind.	0, Not at all | 1, A little bit | 2, Moderately | 3, Quite a bit | 4, Extremely
**mdes_sad_h4**	home_logbook		radio	21. I felt sad, downhearted, or unhappy.	0, Not at all | 1, A little bit | 2, Moderately | 3, Quite a bit | 4, Extremely
**mdes_satisfied_h4**	home_logbook		radio	22. I felt satisfied, fulfilled, content.	0, Not at all | 1, A little bit | 2, Moderately | 3, Quite a bit | 4, Extremely
**mdes_scared_h4**	home_logbook		radio	23. I felt scared, fearful, or afraid.	0, Not at all | 1, A little bit | 2, Moderately | 3, Quite a bit | 4, Extremely
**mdes_stressed_h4**	home_logbook		radio	24. I felt stressed, nervous, or overwhelmed.	0, Not at all | 1, A little bit | 2, Moderately | 3, Quite a bit | 4, Extremely
**mdes_tired_h4**	home_logbook		radio	25. I felt tired, sleepy, drowsy.	0, Not at all | 1, A little bit | 2, Moderately | 3, Quite a bit | 4, Extremely
**mdes_sexual_h4**	home_logbook		radio	26. I felt sexual, desiring, flirtatious.	0, Not at all | 1, A little bit | 2, Moderately | 3, Quite a bit | 4, Extremely
**mdes_sympathy_h4**	home_logbook		radio	27. I felt sympathy, concern, compassion.	0, Not at all | 1, A little bit | 2, Moderately | 3, Quite a bit | 4, Extremely
**saliva_s1colltime_h5**	home_logbook	day 5	text	Time for sample # 1	
**saliva_s2_colltime_h5**	home_logbook		text	Time for sample # 2	
**saliva_s3_colltime_h5**	home_logbook		text	Time for sample # 3	

**saliva_s4_colltime_h5**	home_logbook		text	Time for sample # 4	
**saliva_s2_eatdrink_h5**	home_logbook		text	Time Eat or Drink before Sample # 2	
**saliva_s3_eatdrink_h5**	home_logbook		text	Time Eat or Drink before Sample # 3	
**hl_bedtime_h5**	home_logbook		text	What time did you try to go to sleep last night (lights out)?	
**hl_waketime_h5**	home_logbook		text	What time did you wake up today?	
**hl_sleepquality_h5**	home_logbook		radio	How would you rate the quality of your sleep last night?	1, Very bad | 2, Fairly bad | 3, Fairly good | 4, Very good
**hl_dayprospects_h5**	home_logbook		slider	Please indicate by marking an X on the line below to what extent you are looking forward to versus dreading today's events.	Really dreading today's events | Neutral | Really looking forward to today's events
**hl_dayworried_h5**	home_logbook		slider	Please indicate by marking an X on the line below to hat extent you are worrying about how things are going to go today.	At ease | Neutral | Worrying a lot
**hl_daypredictable_h5**	home_logbook		slider	Please indicate by marking an X on the line below to what extent you feel the events of your upcoming day re predictable (you know exactly what's going to appen today).	Completely Predictable | Completely Unpredictable
**hl_stressed_h5**	home_logbook		radio	I feel stressed, anxious, overwhelmed	0, Not at all | 1, A little bit | 2, Moderately | 3, Quite a bit | 4, Extremely
**hl_control_h5**	home_logbook		radio	I feel in control, coping well, on top of things	0, Not at all | 1, A little bit | 2, Moderately | 3, Quite a bit | 4, Extremely

**hl_joyful_h5**	home_logbook		radio	I feel joyful, glad, happy	0, Not at all | 1, A little bit | 2, Moderately | 3, Quite a bit | 4, Extremely
**hl_sleepdisruption_h5**	home_logbook		text	Did anything disturb your sleep last night?	
**mdes_amused_h5**	home_logbook		radio	1. I felt amused, fun-loving, or silly.	0, Not at all | 1, A little bit | 2, Moderately | 3, Quite a bit | 4, Extremely
**mdes_angry_h5**	home_logbook		radio	2. I felt angry, irritated, or annoyed.	0, Not at all | 1, A little bit | 2, Moderately | 3, Quite a bit | 4, Extremely
**mdes_ashamed_h5**	home_logbook		radio	3. I felt ashamed, humiliated, or disgraced.	0, Not at all | 1, A little bit | 2, Moderately | 3, Quite a bit | 4, Extremely
**mdes_awe_h5**	home_logbook		radio	4. I felt awe, wonder, or amazement.	0, Not at all | 1, A little bit | 2, Moderately | 3, Quite a bit | 4, Extremely
**mdes_bored_h5**	home_logbook		radio	5. I felt bored, disinterested, uninvolved.	0, Not at all | 1, A little bit | 2, Moderately | 3, Quite a bit | 4, Extremely
**mdes_contemptuous_h5**	home_logbook		radio	6. I felt contemptuous, scornful, or disdainful.	0, Not at all | 1, A little bit | 2, Moderately | 3, Quite a bit | 4, Extremely
**mdes_control_h5**	home_logbook		radio	7. I felt control, coping well, on top of things.	0, Not at all | 1, A little bit | 2, Moderately | 3, Quite a bit | 4, Extremely
**mdes_disgust_h5**	home_logbook		radio	8. I felt disgust, distaste, or revulsion.	0, Not at all | 1, A little bit | 2, Moderately | 3, Quite a bit | 4, Extremely
**mdes_embarrassed_h5**	home_logbook		radio	9. I felt embarrassed, self-conscious, or blushing.	0, Not at all | 1, A little bit | 2, Moderately | 3, Quite a bit | 4, Extremely
**mdes_excited_h5**	home_logbook		radio	10. I felt excited, eager, enthusiastic.	0, Not at all | 1, A little bit | 2, Moderately | 3, Quite a bit | 4, Extremely

**mdes_grateful_h5**	home_logbook		radio	11. I felt grateful, appreciative, or thankful.	0, Not at all | 1, A little bit | 2, Moderately | 3, Quite a bit | 4, Extremely
**mdes_guilty_h5**	home_logbook		radio	12. I felt guilty, repentant, or blameworthy.	0, Not at all | 1, A little bit | 2, Moderately | 3, Quite a bit | 4, Extremely
**mdes_hatred_h5**	home_logbook		radio	13. I felt hatred, distrust, or suspicion.	0, Not at all | 1, A little bit | 2, Moderately | 3, Quite a bit | 4, Extremely
**mdes_hopeful_h5**	home_logbook		radio	14. I felt hopeful, optimistic, or encouraged.	0, Not at all | 1, A little bit | 2, Moderately | 3, Quite a bit | 4, Extremely
**mdes_inspired_h5**	home_logbook		radio	15. I felt inspired, uplifted, or elevated.	0, Not at all | 1, A little bit | 2, Moderately | 3, Quite a bit | 4, Extremely
**mdes_interested_h5**	home_logbook		radio	16. I felt interested, alert, or curious.	0, Not at all | 1, A little bit | 2, Moderately | 3, Quite a bit | 4, Extremely
**mdes_joyful_h5**	home_logbook		radio	17. I felt joyful, glad, or happy.	0, Not at all | 1, A little bit | 2, Moderately | 3, Quite a bit | 4, Extremely
**mdes_love_h5**	home_logbook		radio	18. I felt love, closeness, or trust.	0, Not at all | 1, A little bit | 2, Moderately | 3, Quite a bit | 4, Extremely
**mdes_proud_h5**	home_logbook		radio	19. I felt proud, confident, or self-assured.	0, Not at all | 1, A little bit | 2, Moderately | 3, Quite a bit | 4, Extremely
**mdes_rejected_h5**	home_logbook		radio	20. I felt rejected, betrayed, left-behind.	0, Not at all | 1, A little bit | 2, Moderately | 3, Quite a bit | 4, Extremely
**mdes_sad_h5**	home_logbook		radio	21. I felt sad, downhearted, or unhappy.	0, Not at all | 1, A little bit | 2, Moderately | 3, Quite a bit | 4, Extremely
**mdes_satisfied_h5**	home_logbook		radio	22. I felt satisfied, fulfilled, content.	0, Not at all | 1, A little bit | 2, Moderately | 3, Quite a bit | 4, Extremely

**mdes_scared_h5**	home_logbook		radio	23. I felt scared, fearful, or afraid.	0, Not at all | 1, A little bit | 2, Moderately | 3, Quite a bit | 4, Extremely
**mdes_stressed_h5**	home_logbook		radio	24. I felt stressed, nervous, or overwhelmed.	0, Not at all | 1, A little bit | 2, Moderately | 3, Quite a bit | 4, Extremely
**mdes_tired_h5**	home_logbook		radio	25. I felt tired, sleepy, drowsy.	0, Not at all | 1, A little bit | 2, Moderately | 3, Quite a bit | 4, Extremely
**mdes_sexual_h5**	home_logbook		radio	26. I felt sexual, desiring, flirtatious.	0, Not at all | 1, A little bit | 2, Moderately | 3, Quite a bit | 4, Extremely
**mdes_sympathy_h5**	home_logbook		radio	27. I felt sympathy, concern, compassion.	0, Not at all | 1, A little bit | 2, Moderately | 3, Quite a bit | 4, Extremely
