# Supplementary material for: A platform to map the mind–mitochondria connection and the hallmarks of psychobiology: the MiSBIE study
Source: Trends Endocrinol Metab. Author manuscript; Available in PMC 2024 Nov 12. (PMC11555495; doi:10.1016/j.tem.2024.08.006)
Supplement: MMC1 — File S1. Summary MiSBIE protocol and outcome measures. [file NIHMS2028739-supplement-MMC1.pdf]

Summary MiSBIE Protocol and Outcome Measures

The narrative below summarizes the major elements of the MiSBIE Study protocol and the associated outcome measures. These sections are organized chronologically in accordance with the Day 1 and Day 2 procedures, as outlined in Supplemental Figure 1. For further details on the MiSBIE protocol, please refer to Supplemental File 4. For a detailed list of all data available in the MiSBIE database as of 2024, please refer to Supplemental File 6.

The figure below broadly outlines the combination of mitochondrial phenotyping (*left*), stress-reactivity and mitochondrial disease (MitoD)-related outcome measures (*right*), and the potential mediating role of the brain and associated neural, cognitive, and emotional processes.

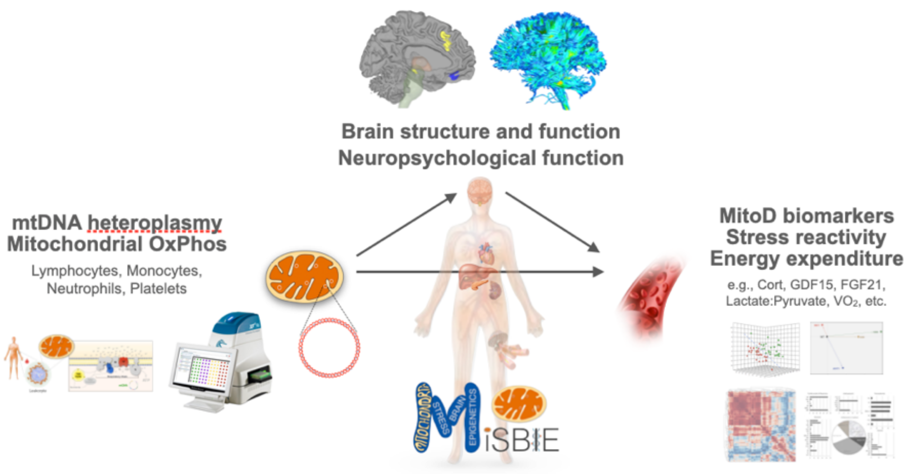

Fasting blood biomarkers, immune cell isolation, and biospecimen collection

Fasting blood was drawn at ~9:50 AM with an intravenous catheter on the left arm. A total of 100ml was collected for standard blood chemistry, complete blood count with differential (CBC w diff.), and metabolic panel. In addition, serum (red top), plasma (EDTA, citrate), PBMCs frozen both as dry pellets and cryopreserved for downstream analyses, and isolated monocytes, lymphocytes, and neutrophils were stored (cells in RNAlater for sequencing, and dry pellets for biochemistry and genetic analyses) were stored. A total of 26 saliva samples were also collected for each participant (14 during the 2-day protocol, 12 at home). Hair for steroid hormones (two 3cm samples), a buccal swab for DNA, fecal matter for microbiome, and overnight urine (8 PM – 8 AM hotel night between days 1 and 2) for catecholamines were collected and stored as multiple aliquots. All collected samples and MiSBIE biobank aliquots (n=164 per participant) are detailed in Supplemental File 5. An overview of biospecimens collected and processing steps is presented in Supplemental Figure 4.

Mitochondrial phenotyping

Mitochondrial phenotyping is achieved in three complementary ways. 1) stored pellets of 1-5M peripheral blood mononuclear cells (PBMCs) were used to quantify OxPhos enzyme activities (complexes I, II, IV) plus markers of mitochondrial content (citrate synthase, mtDNA copy number) [1]. However, different types of immune cells contain strikingly different mitochondrial phenotypes (i.e., mitotypes), which confounds measures in mixed cell populations [2]. We therefore performed these measures also in four major cell types (monocytes, lymphocytes, neutrophils, and platelets; isolated with magnetic beads). These measures are then integrated into a mitochondrial health index (MHI; or mitochondrial respiratory capacity, MRC) [3], which reflects energy transformation capacity on a per-mitochondrion basis. 2) Basal and uncoupled (i.e., maximal) respiratory and glycolytic capacity, as well as other parameters, were measured using extracellular flux analysis in the same four, freshly isolated cell types [4]. In addition, all measurements were repeated while inhibiting the mitochondrial uptake of the major source of

carbon/energy, the pyruvate carrier (UK50099), yielding estimates of mitochondrial "metabolic flexibility" for each cell type. 3) Cryopreserved PBMCs are used to perform molecular profiling of immune cell mitochondria using single-cell RNA sequencing (scRNAseq). This mitotyping [5,6] approach yields a high-dimensional picture of cell subtype-specific mitotypes (149 MitoPathways x ~30 cell types). The resulting functional and molecular immune mitochondrial phenotypes can be integrated to produce lower dimensionality metrics of OxPhos capacity and more refined mitochondrial phenotypes for each MiSBIE participant. All methodological details and protocols for these measurements are available in Supplemental File 4.

Clinical Assessment

We assess clinical symptoms of mitochondrial disease through a general medical assessment and with five clinical impairment scales including: 1) the Newcastle Mitochondrial Disease Assessment Scale (NMDAS) [7], 2) Columbia Neurological Score (CNS) [8], 3) the composite autonomic symptoms score (COMPASS-31) [9], 4) functional capacity based on a 30-second sit-stand test [10], 5) two self-reported instruments for fatigue including the fatigue impact scale (FIS) [11] and the Pittsburgh Fatiguability Scale (PFS) [12], and 6) the Karnofsky Performance Scale [13]. Other indicators of disease severity include 7) the North American Mitochondrial Disease Consortium (NAMDC) Case Report Form [14], 8) the illness perception [15], and 9)the memory complaint checklist (MCC), which reflects the participant's perception of their memory impairment in daily life, serving as a complement to the more objective assessment by neuropsychological testing [16]. All instruments are listed in Supplemental File 4 and self-reported questionnaires with their administration order are detailed in Supplemental Table 2.

Body composition and energetics

Body composition (% fat mass and fat-free mass, FFM) was estimated using a 4-point bioelectrical impedance device. Resting energy expenditure was estimated in the sitting position by measuring oxygen consumption on two occasions across the two-day protocol (Day 1 afternoon, Day 2 morning; Supplemental Figure 1) as described in Supplemental File 4.

Neuropsychological testing

A total of 18 neurocognitive tests were administered to quantify 13 main variables reflecting different cognitive domains including working memory, language production, visual learning, and cognitive flexibility. The test battery includes subtests from the Delis-Kaplan Test of Executive Functions (DKEFS) [17], Neuropsychological Assessment Battery (NAB) [18], Repeatable Battery for the Assessment of Neuropsychological Status (RBANS) [19], Wechsler Abbreviated Scale of Intelligence (WASI II) [20], and Test of Premorbid Functioning (TOPF) [21]. The assessment lasted ~90 minutes on the morning of Day 2. Details are listed in Supplemental File 4

Questionnaires and psychosocial measures

We assess psychosocial and behavioral constructs including demographics (e.g. education, socio-economic status), social support, acute and chronic stress, mood and affect, personality, life events, health-related behaviors (diet, sleep, exercise, etc.), mental health and clinical symptoms as well as perception of aging. The questionnaire battery (39 questionnaires) was administered as five coherent packages strategically distributed along the 2-day study visit. The complete list of instruments and subscales, order of administration, and approximate durations are detailed in Supplemental Table 2 The coding scheme for each questionnaire is detailed in the Data Dictionary (Supplemental File 6).

Stress reactivity

To elicit the stress response, the MiSBIE study used a modified speech task adapted from the Trier Social Stress Test (TSST) [22-25], which lasted 5 minutes: 2 minutes of speech preparation and 3 minutes of delivery. Participants were asked to prepare and deliver a simulated public speech task, consisting of defending themselves against an alleged transgression (shoplifting on Day 1, running a stop sign on Day 2 in the scanner) while directly facing an evaluator wearing a white lab coat, a mirror, and video camera. Further details on the stress challenges administered in the MiSBIE study can be found in Supplemental File 4. To collect blood along the stress reactivity and recovery periods, an intravenous catheter was inserted into the right arm >45 minutes before the onset of the TSST. Blood was collected at the following timepoints relative to the beginning of the speech task: -5, +5, 10, 20, 30, 60, 90, and 120 minutes (n= 8

timepoints). Simultaneously with each blood draw, we collected saliva using salivettes [26], surface body temperature (4 corporeal locations), and affect (3 positive, 4 negative affect items).

To assess psychophysiological reactivity/recovery [27-30] to non-psychological stressors, participants performed four other tasks including: 1) a paced deep breathing (PDB) task (5-second inspiration, 7-second exhalation) that elicits parasympathetic outflow and increases heart rate variability (HRV) [31,32]; 2) an orthostatic challenge induced with the transition from sitting to standing (ST) for a 5-min period [33]; 3) a 30-seconds period of sit-stand transition (SST) as fast as possible, which is highly energy demanding, reflects functional capacity and is used as a measure of frailty [34]; and 4) the cold pressor test (CP) [35-37] where participants submerge their right hand in cold water (3.1-3.4°C) for 90 seconds, a painful (average 6.72 on a 0-10 scale) but not energy expensive stimulus [38,39]. The instruction scripts used by the study coordinator are available in **Supplemental File 4**.

For the duration of the speech task reactivity and recovery protocol, we continuously measured the following signals: i) cardiac activity using 3-lead electrocardiography (ECG), ii) beat-to-beat blood pressure using finger plethysmography, iii) sympathetic nervous system (SNS) outflow using skin conductance (electrodermal activity, EDA), and iv) ventilatory dynamics (rate and volume) with chest and abdominal sensors. These data are sampled at high frequency (1000 Hz) and available as aggregate pre-processed averages over variable intervals (10 seconds, 1 minute, 5 minutes) along each period of the protocol (see **Supplemental Figure 2**).

Neuroimaging

In the afternoon of Day 2, we measured brain anatomy using T1- and T2-weighted magnetic resonance on a 3 T Siemens Prisma scanner (Siemens Medical Solutions). We then collected functional MRI images using the same scanner to measure BOLD activity during psychological tasks. The tasks included:

1) a multisensory task (5 min), which included alternating 30-sec periods of rest and a full-field, 3 Hz contrast-reversing checkerboard combined with auditory stimulation (15 tones from 233-1319 Hz) [40]

2) resting state with eyes open (10 min);

3) an N-back task (0-back and 2-back, 2 scans each 4:28 mins), which included alternating working memory tasks where participants viewed a series of stimuli (body part, face, place, tool) and quickly decided if the current stimulus was the same as the one presented N (1 or 2) back;

4) MRI-compatible social evaluative threat task (6 mins), adapted from the Trier Social Stress Test (TSST) as in [41], in which participants first rested (2 min), then prepared to give a speech as on Day 1 (2 min, see specifics in Stress Reactivity task above) with the additional provision of negative feedback about the participant's previous day performance to prevent habituation, then were told that they had been randomly selected to not give the speech and to rest during the last portion of the scan (2 min);

5) a modified cold pressor task (6 min), which elicited mild pain (average 4.01 on a 0-10 scale); a room temperature wrap was wrapped around the participant's right hand and wrist (1.5 min), this was replaced by a frozen ice pack by an experimenter waiting in the scan room (2 min) and finally replaced with the room temperature wrap again (1.5 min).

We then imaged white matter anatomy with a multishell (b = 0; 300, 1000, and 2000) diffusion-weighted imaging sequence distributed over two scans (21 mins) [42]. The detailed MRI protocol and detailed participant instructions are described and illustrated in **Supplemental File 4**.

Home-based saliva sampling of the awakening response and actigraphy

At home during the week following their on-site visit, participants collected saliva at four timepoints (0, 30, 45 min after waking up, and at bedtime) on three non-consecutive days (Monday, Wednesday, Friday) for a total of 12 samples (4 timepoints x 3 days). Collection times and instructions were based on guidelines to quantify the cortisol awakening response [43-45]. Samples were collected in parallel with mood, stress, and coping measures in the morning and evening over the course of the week using a home logbook and standardized daily stress assessments from the NIH Stress Measurement Network [46] (**Supplemental File 3**). These were implemented in a custom-designed MiSBIE App accessed with an iPad. For a period of ~10 days starting on Day 2 of their visit and including this at-home sample collection week, participants

wore a wrist actigraph (Actiwatch) to quantify physical activity and estimate sleep duration and quality as well as circadian functioning, complemented with daily subjective reports of sleep satisfaction, and the Pittsburgh Sleep Quality Index (PSQI) [47].

Database and analysis plan

Analytically, the MiSBIE study will be amenable to five main types of analyses. 1) Simple group means (i.e., case-control) comparisons between control (Group 1) and mitochondrial diseases (MitoD) (Groups 2-4). 2) Contrast analyses between Mutation and Deletion (Group 2/3 vs 4), to get at the specificity of mtDNA defects. 3) Regression analyses among the mtDNA defect groups will identify factors associated with disease severity or specific symptom constellations. 4) Regression analyses among the control group, or among the entire study cohort, to identify general mitochondrial psychobiological associations among individuals with a broad range of mitochondrial OxPhos capacity. And 5) Multivariate and other types of analyses can be used to discover subsets of individuals with specific multisystem, psychobiological profiles linked to specific outcomes of interest.

The deep phenotyping MiSBIE study database includes >5,000 variables distributed across 16 major categories. The dimensionality of these major outcome variables is summarized in Table 1. All variables and their description are described in the Data Dictionary (**Supplemental File 6**).

References

1. Karan, K.R. et al. (2020) Mitochondrial respiratory capacity modulates LPS-induced inflammatory signatures in human blood. Brain Behav Immun Health 5, 100080. 10.1016/j.bbih.2020.100080

2. Rausser, S. et al. (2021) Mitochondrial phenotypes in purified human immune cell subtypes and cell mixtures. Elife 10. 10.7554/eLife.70899. 10.7554/eLife.70899

3. Picard, M. et al. (2018) A Mitochondrial Health Index Sensitive to Mood and Caregiving Stress. Biol Psychiatry 84, 9-17. 10.1016/j.biopsych.2018.01.012

4. Kramer, P.A. et al. (2014) Bioenergetics and the oxidative burst: protocols for the isolation and evaluation of human leukocytes and platelets. Journal of visualized experiments: JoVE, (85), 51301. 10.3791/51301

5. Rosenberg, A.M., et al. (2023) Brain mitochondrial diversity and network organization predict anxiety-like behavior in male mice. Nature Communications. 14(1), 4726. 10.1038/s41467-023-39941-0

6. Monzel, A.S. et al. (2023) Multifaceted mitochondria: moving mitochondrial science beyond function and dysfunction. Nat Metab 5, 546-562. 10.1038/s42255-023-00783-1

7. Schaefer, A.M. et al. (2006) Mitochondrial disease in adults: a scale to monitor progression and treatment. Neurology 66, 1932-1934. 10.1212/01.wnl.0000219759.72195.41

8. Kaufmann, P. et al. (2004) Cerebral lactic acidosis correlates with neurological impairment in MELAS. Neurology 62, 1297-1302. 10.1212/01.wnl.0000120557.83907.a8

9. Sletten, D.M. et al. (2012) COMPASS 31: a refined and abbreviated Composite Autonomic Symptom Score. Mayo Clin Proc 87, 1196-1201. 10.1016/j.mayocp.2012.10.013

10. Beaudart, C. et al. (2019) Assessment of Muscle Function and Physical Performance in Daily Clinical Practice : A position paper endorsed by the European Society for Clinical and Economic Aspects of Osteoporosis, Osteoarthritis and Musculoskeletal Diseases (ESCEO). Calcif Tissue Int 105, 1-14. 10.1007/s00223-019-00545-w

11. Fisk, J.D. et al. (1994) Measuring the functional impact of fatigue: initial validation of the fatigue impact scale. Clin Infect Dis 18 Suppl 1, S79-83. 10.1093/clinids/18.supplement_1.s79

12. Glynn, N.W. et al. (2015) The Pittsburgh Fatigability scale for older adults: development and validation. J Am Geriatr Soc 63, 130-135. 10.1111/jgs.13191

13. Karnofsky, D.A., Burchenal, J.H. (1949) The Clinical Evaluation of Chemotherapeutic Agents in Cancer. In Evaluation of Chemotherapeutic Agents (MacLeod, C.M., ed), Columbia University Press

14. Emmanuele, V. et al. (2022) Time to harmonize mitochondrial syndrome nomenclature and classification: A consensus from the North American Mitochondrial Disease Consortium (NAMDC). Mol Genet Metab 136, 125-131. 10.1016/j.ymgme.2022.05.001

15. Broadbent, E. et al. (2006) The brief illness perception questionnaire. J Psychosom Res 60, 631-637. 10.1016/j.jpsychores.2005.10.020

16. Sunderland, A., Harris, J.E., Baddeley, A.D. (1983) Do Laboratory Tests Predict Everyday Memory? A Neuropsychological Study. Journal of Verbal Learning and Verbal Behavior, 22(3), 341-357. 10.1016/S0022-5371(83)90229-3

17. Delis, D.C., Kaplan, E., Kramer, J.H.. (2001) Delis-Kaplan Executive Function System (D-KEFS): Technical Manual Pearson

18. Stern, R., White, T. (2001) Neuropsychological Assessment Battery (NAB): Technical Manual Psychological Assessment Battery

19. Randolph, C. (2012) Repeatable Battery for the Assessment of Neuropsychological Status Update (RBANS): Technical Manual Pearson

20. Wechsler, D. (2011) Wechsler Abbreviated Scale of Intelligence | Second Edition: Technical Manual Pearson

21. Wechsler, D. (2009) Test of Premorbid Functioning (TOPF): Techinical Manual Pearson

22. Strahler, J. et al. (2015) Acute psychosocial stress induces differential short-term changes in catecholamine sensitivity of stimulated inflammatory cytokine production. Brain Behav Immun 43, 139-148. 10.1016/j.bbi.2014.07.014

23. Giese-Davis, J. et al. (2006) Depression and stress reactivity in metastatic breast cancer. Psychosom Med 68, 675-683. 10.1097/01.psy.0000238216.88515.e5

24. Dedovic, K. et al. (2005) The Montreal Imaging Stress Task: using functional imaging to investigate the effects of perceiving and processing psychosocial stress in the human brain. Journal of Psychiatry & Neuroscience 30(5), 319-325

25. Kirschbaum, C. et al. (1995) Persistent high cortisol responses to repeated psychological stress in a subpopulation of healthy men. Psychosom Med 57, 468-474. 10.1097/00006842-199509000-00009

26. Trumpff, C. et al. (2021) Stress and circulating cell-free mitochondrial DNA: A systematic review of human studies, physiological considerations, and technical recommendations. Mitochondrion 59, 225-245. 10.1016/j.mito.2021.04.002

27. Brosschot, J.F. et al. (2005) Expanding stress theory: prolonged activation and perseverative cognition. Psychoneuroendocrinology 30, 1043-1049. 10.1016/j.psyneuen.2005.04.008

28. Brosschot, J.F. (2010) Markers of chronic stress: prolonged physiological activation and (un)conscious perseverative cognition. Neurosci Biobehav Rev 35, 46-50. 10.1016/j.neubiorev.2010.01.004

29. Lovallo, W.R. and Gerin, W. (2003) Psychophysiological reactivity: mechanisms and pathways to cardiovascular disease. Psychosom Med 65, 36-45. 10.1097/01.psy.0000033128.44101.c1

30. Lovallo, W.R. (2010) Cardiovascular responses to stress and disease outcomes: a test of the reactivity hypothesis. Hypertension 55 (4), 842-843. 10.1161/HYPERTENSIONAHA.110.149773

31. Magnon, V. et al. (2021) Benefits from one session of deep and slow breathing on vagal tone and anxiety in young and older adults. Sci Rep 11, 19267. 10.1038/s41598-021-98736-9

32. Russo, M.A. et al. (2017) The physiological effects of slow breathing in the healthy human. Breathe (Sheff) 13, 298-309. 10.1183/20734735.009817

33. Dienberg Love, G. et al. (2010) Bioindicators in the MIDUS national study: protocol, measures, sample, and comparative context. J Aging Health 22, 1059-1080. 10.1177/0898264310374355

34. McCarthy, E.K., et al. (2004) Repeated chair stands as a measure of lower limb strength in sexagenarian women. The journals of gerontology Series A, Biological sciences and medical sciences (59(11)), 1207–1212. 10.1093/gerona/59.11.1207

35. Bullinger, M. et al. (1984) Endocrine effects of the cold pressor test: relationships to subjective pain appraisal and coping. Psychiatry Res 12, 227-233. 10.1016/0165-1781(84)90028-3

36. Errico, A.L. et al. (1993) Attenuated cortisol response to biobehavioral stressors in sober alcoholics. J Stud Alcohol 54, 393-398. 10.15288/jsa.1993.54.393

37. Pascualy, M. et al. (2000) Hypothalamic pituitary adrenocortical and sympathetic nervous system responses to the cold pressor test in Alzheimer's disease. Biol Psychiatry 48, 247-254. 10.1016/s0006-3223(00)00879-9

38. Hines, E.A., Brown, G. E. (1936) The cold pressor test for measuring the reactibility of the blood pressure: Data concerning 571 normal and hypertensive subjects. The American Heart Journal 11, 1-9. 10.1016/S0002-8703(36)90370-8.

39. al'Absi, M. et al. (2002) Adrenocortical and hemodynamic predictors of pain perception in men and women. Pain 96, 197-204. 10.1016/s0304-3959(01)00447-x

40. Lopez-Sola, M. et al. (2017) Towards a neurophysiological signature for fibromyalgia. Pain 158, 34-47. 10.1097/j.pain.0000000000000707

41. Wager, T.D. et al. (2009) Brain mediators of cardiovascular responses to social threat: part I: Reciprocal dorsal and ventral sub-regions of the medial prefrontal cortex and heart-rate reactivity. Neuroimage 47, 821-835. 10.1016/j.neuroimage.2009.05.043

42. Zhang, H. et al. (2012) NODDI: practical in vivo neurite orientation dispersion and density imaging of the human brain. Neuroimage 61, 1000-1016. 10.1016/j.neuroimage.2012.03.072

43. Stalder, T. et al. (2016) Assessment of the cortisol awakening response: Expert consensus guidelines. Psychoneuroendocrinology 63, 414-432. 10.1016/j.psyneuen.2015.10.010

44. Trumpff, C. et al. (2022) Dynamic behavior of cell-free mitochondrial DNA in human saliva. Psychoneuroendocrinology 143, 105852. 10.1016/j.psyneuen.2022.105852

45. Chida, Y. and Steptoe, A. (2009) Cortisol awakening response and psychosocial factors: a systematic review and meta-analysis. Biol Psychol 80, 265-278. 10.1016/j.biopsycho.2008.10.004

46. Almeida, D.M. et al. (2002) The daily inventory of stressful events: an interview-based approach for measuring daily stressors. Assessment 9, 41-55. 10.1177/1073191102091006

47. Buysse, D.J. et al. (1989) The Pittsburgh Sleep Quality Index: a new instrument for psychiatric practice and research. Psychiatry Res 28, 193-213. 10.1016/0165-1781(89)90047-4
