# Supplementary material for: A platform to map the mind–mitochondria connection and the hallmarks of psychobiology: the MiSBIE study
Source: Trends Endocrinol Metab. Author manuscript; Available in PMC 2024 Nov 12. (PMC11555495; doi:10.1016/j.tem.2024.08.006)
Supplement: MMC4 — File S4. Detailed protocol, step-by-step experimental procedures, illustrations, materials, and scripts v1.0. [file NIHMS2028739-supplement-MMC4.pdf]

Supplemental File 4

CLINICAL PROCEDURES AND LABORATORY METHODS

This document includes all details necessary to interpret and replicate the NIH-funded Mitochondrial Stress, Brain Imaging, and Epigenetics (MiSBIE) research study performed at Columbia University Irving Medical Center (CUIMC).

This document is organized in two main sections. We begin with a general narrative overview of the procedures across the 2-day protocol (*Section 1*). We then describe the procedures and methods to collect and generate data (*Section 2*). The 12 subsections include methodological details on physiological measurements, collection and processing of biospecimens, study-specific tasks, neuropsychological assessment, magnetic resonance imaging (MRI) procedures, self-report questionnaires, and home-based data collection.

For each procedure, we provide the item description (with catalog #), a brief background about the measurement or assay, a detailed description of the procedures, and related outcome measures that can be found in the MiSBIE database and Data Dictionary (Supplemental File 4).

Table of Contents

General Procedural Overview: **4**

Day 1: **4**

Day 2: **5**

Controlled study conditions and design elements: **7**

Detailed Methods ***8***

1 Screening Procedures **8**
1.1 Recruitment 8
1.2 Phone Screening 8

2 Physiological Measures **10**
2.1 Resting Blood Pressure and Heart Rate 10
2.2 Height and Weight 10
2.3 Hip and Waist Circumference 11
2.4 Body Composition 12
2.5 Temperature 13
2.6 Spirometry 14
2.7 Heart Rate 15
2.8 Electrodermal Activity 16
2.9 Respiratory Rate 17
2.10 Blood Pressure 18
2.11 Resting Metabolic Rate 19

3 Biospecimen Collection **21**
3.1 Morning Blood Draw 21
3.2 Afternoon Blood Draw 22
3.3 Saliva Sample 23
3.4 Buccal Swab 24
3.5 Hair Collection 24
3.6 Urine Collection 26

4 Biospecimen Processing **28**
4.1 Fasting Serum Processing 28
4.2 Fasting Plasma Processing 28
4.3 Whole Blood Processing 29
4.3.1 Platelet Isolation 29

4.3.2 Purifying PBMCs ... 29
4.3.3 PBMC Cryopreservation ... 31
4.3.4 Purifying Leukocyte Subtypes ... 32
4.3.5 Lymphocyte Isolation ... 34
4.3.6 Cell counting and platelet quantification ... 35
4.3.7 Seahorse Bioenergetics Measurements ... 36
4.3.8 Leukocyte Storage ... 38
4.3.9 HEK Passaging ... 38
4.4 Blood Processing: Stress Reactivity ... 39
4.4.1 Serum and Plasma Processing ... 39
4.4.2 PBMC Cryopreservation ... 40
4.4.3 Whole Blood Processing: ... 41
4.4.4 Purifying PBMCs ... 41
4.5 Saliva Processing ... 42
4.5.3 Diurnal Saliva processing ... 43
4.6 Urine Processing ... 43
4.7 Fecal Sample Processing ... 43

5 Biospecimen Measurements ... 44
5.1 Cell-free DNA ... 44
5.2 Biogenic Amines ... 45
5.3 Steroid Hormones ... 45
5.4 GDF-15 ... 46

6 Study Specific Tasks ... 47
6.1 Psychophysiological Session Flow ... 47
6.2 Speech Task ... 47
6.3 Deep Breathing Task ... 49
6.4 Stand Task ... 50
6.5 Sit-to-Stand Task ... 50
6.6 Cold Pressor Task ... 52
6.7 Resting Metabolic Rate (RMR) ... 53
6.8 Verbal Time Estimation Task ... 54

7 Medical assessment ... 57
7.1 Columbia Neurological Score (CNS) ... 57
7.2 Newcastle Mitochondrial Disease Assessment Scale (NMDAS) ... 57
7.3 NAMDC Case Report Form (CRF) ... 57
7.4 Karnofsky Performance Scale Index ... 57

8 Neuropsychological assessment ... 58
8.1 Premorbid Functioning ... 58
8.2 Intellectual Functioning ... 58
8.3 Visuospatial ... 58
8.4 Language ... 58
8.5 Memory ... 59
8.6 Executive Functioning and Attention ... 59

9 Magnetic Resonance Imaging (MRI) ... 60
9.1 MR-safe Electrocardiography ... 63
9.2 Multisensory Scan ... 64
9.3 N-back Task ... 65
9.4 Modified Speech Task ... 65
9.5 Modified Cold Pressor ... 66

10 Self-report questionnaires ... 68
10.1 Psychosocial and Personality ... 68
10.2 Mental Health and Wellbeing ... 68
10.3 Life Events ... 68
10.4 Health Related Behaviors ... 69
10.5 Demographic ... 69

10.6 Physical and Cognitive Symptoms 69
10.7 Affect Ratings 69

11 Home-Based Sample Collection 71
11.1 MiSBIE Home Logbook 71
11.2 Saliva Samples 72
11.3 Fecal Sample Collection 73
11.4 Actigraphy 74

12 Supplemental Resources 76
12.1 Purifying Leukocyte Subtypes Numbered Protocol 76
12.2 Trier Social Stress Task Day 1 90
12.3 Verbal Time Estimation Task 91
12.4 Speech Task Day 2 93
12.5 Columbia Suicide Severity Rating Scale (C-SSRS) 93
12.6 Debriefing Script 94
12.7 Breakfast and Lunch Menu 95

General Procedural Overview:

Day 1:

Prior to the study visit, all inclusion and exclusion criteria are verified. MiSBIE study visits always take place on Tuesdays (Day 1) and Wednesdays (Day 2). All participants stay at a nearby hotel for at least two nights, Monday and Tuesday nights (some are allowed to stay an extra night to accommodate out-of-state travels the following day). Upon check-in to the hotel, participants are given a package containing informational documents (consent form, general itinerary, and study instructions) as well as a short questionnaire to complete before Tuesday morning. On Tuesday morning at 9 am, participants arrive at CUIMC via a scheduled car service (Lyft Concierge). They are greeted in the adult emergency room lobby by the study coordinator. The study coordinator completes COVID-19 screening questions and takes participants' forehead temperature before escorting them to a private room to administer informed consent.

Participants are led to a private conference room to complete informed consent as well as a HIPAA authorization form. Participants are asked compliance questions to ensure protocol consistency across visits and are asked to complete the Columbia Suicide Severity Rating Scale (C-SSRS) to ensure participant safety during the duration of their visit. After consent is completed and all participant questions are addressed, anthropomorphic measurements are completed including resting blood pressure, height, weight, hip and waist circumference. Body composition is estimated using the TANITA body composition scale. Participants are then relocated to the neighboring Psychophysiology Laboratory. Momentary general affect (emotional state), body temperature, and a saliva sample are collected prior to the fasting blood draw. The study nurse inserts an intravenous catheter in the left antecubital vein to complete a fasting blood draw including the following collection tubes: 2.7ml buffered sodium citrate tubes (5), 2ml EDTA coated tube, 5ml serum separation tubes (2), 8.5ml acid citrate dextrose tubes (5), 8ml non-coated serum tubes (2), and 8ml plasma tubes (2).

Immediately following the morning blood draw, participants' affect and discomfort ratings are recorded. The study coordinator collects epithelial buccal cells from the inside of the mouth (cheek) using a buccal swab. Participants are then given breakfast; meal choices and food intake are documented to account for nutritional variance between subjects. After breakfast, two small hair samples are collected from the back of the head for hormone and molecular analyses. Lung function is measured by spirometry and a medical assessment is completed by a trained physician. Participants are then given lunch, where again, food choices are documented.

The stress reactivity-recovery paradigm is completed in the afternoon of Day 1. The nurse places an intravenous line in participants' right arm, typically the forearm, and the physiological technician instruments participants with the following devices: a 3-lead ECG to monitor heart rate, a finger cuff blood pressure monitor, skin conductance surface device to monitor sympathetic nervous system activity (sweating), and two respiration bands placed on the abdomen and lower chest to measure ventilation. After instrumentation, participants rest quietly for 30 minutes alone in a room without access to their personal phones or other media. The study coordinator, technician, and nurse sit in an adjacent room where participants are visible through a one-sided window. Participants are exposed to a standard socio-evaluative laboratory challenge and are asked to perform a simulated public speaking task consisting of two minutes of preparation, followed by three minutes of mock-videotaped speech delivery where participants defend themselves against an alleged transgression. Data and biospecimens are collected at a total of eight (8) time points: before and throughout the two-hour task period, at the following times relative to the onset of the stressor: -5, +5, +10, +20, +30, +60, +90 and +120 minutes. At each time point, the nurse and study coordinator collect repeated samples of blood, saliva, physiological monitoring (blood pressure, heart rate variability, galvanic skin response, and respiration), body temperature, and affect self-reports. Each blood collection consists of 10ml of plasma (5ml) and serum (5ml). At the final time point (+120), an additional 8.5 ml of blood is collected to isolate total PBMCs for enzymatic and molecular analyses, and one tube (2ml) for CBC with differential cell count, for a total of 10.5ml in addition to other samples. For a subset of participants (starting at 45), total PBMCs are cryopreserved from the blood samples at all eight-time stress time points and fasting.

Five minutes after the final sample collection period (+120) participants complete a deep breathing task with a visual guide, enabling assessment of autonomic regulation through breathing. Participants are then asked to transition from a sitting to a standing position, which they maintain for five minutes, allowing monitoring of the orthostatic response. Participants then complete the sit-stand test, a 30-second test of functional capacity and frailty. Participants complete the cold pressor task where they immerse their right hand and wrist into a bucket filled with cold water for (up to) 90 seconds. This is followed by a 10-minute recovery period. Finally, a portion of participants (starting at Mi043) perform a 10-minute resting metabolic rate measurement before being de-instrumented. After returning to the 15th floor, participants complete the time estimation task, which measures participants' perception of time or their "internal clock".

The study coordinator provides reminders and instructions for sample and questionnaire collection for the evening of Day 1, Tuesday. Participants are asked to complete two questionnaires and to collect urine overnight between 8 pm-8 am.

Day 2:

On Wednesday morning (Day 2), participants arrive at CUIMC via a scheduled car service (Lyft Concierge). They are greeted at 9 am in the adult emergency entrance by the study coordinator. The study coordinator, again, completes COVID-19 screening questions and takes participants' forehead temperature before escorting them to the 15th floor. The study coordinator completes compliance questions to ensure general study instructions and sample collection protocols are followed and collects completed hotel questionnaires and urine sample.

Participants complete an affect self-report questionnaire and a saliva sample. During saliva collection (2-5 minutes), participants complete a magnetic resonance imaging screening form provided by the imaging institute to ensure participant safety. Any participant with a female-assigned reproductive system completes a pregnancy test and shares the completed test with the study coordinator; a positive test disqualifies participants from completing neuroimaging procedures.

Prior to breakfast, participants (starting at Mi043) perform a 10-minute resting metabolic rate measurement. Participants eat breakfast, meal choices and leftovers are documented.

Immediately following breakfast, the study coordinator administers a neuropsychological assessment. Participants and study coordinator sit in adjacent rooms, most of the assessment is delivered virtually, through Zoom; neuropsychological tests that require direct interaction with participants are administered in face-to-face.

At approximately 11:30 pm, participants eat lunch. After lunch, participants begin a set of three questionnaire packages completed independently. One of the instruments assessing domains of mental health address suicidal thoughts and ideation. To comply with institutional regulations and ensure participant safety, the study coordinator monitors responses to these questions. If participants indicate thoughts of suicide and has a moderate Beck Depression Inventory (BDI)(>=17) or indicates that they want to kill themselves on the BDI, the study coordinator initiates a conversation to determine whether participants require, or request, an evaluation with the study psychiatrist using the Columbia Suicide Severity Rating Scale (C-SSRS).

Participants and study coordinator then travel to the Mortimer B. Zuckerman Mind Brain Behavior Institute (ZMBBI) for Magnetic Resonance Imaging procedures. Participants and study coordinator meet in a private room (Zone II) to prepare for neuroimaging. To ensure participants feel comfortable and to minimize novelty of the scanning environment, the study coordinator completes an overview of the schedule and detailed procedures of the scan, including playing scanner noises (T1/T2, BOLD, DTI pulse sequences) and showing pictures of the magnet bore. Participants also complete a matching task on a laptop which simulates a task they will complete in the scanner. Participants change into a set of medical scrubs and removes all jewelry and metal in or on their body. The study coordinator instruments

the predicant with MRI-safe electrode leads on the (1) right thoracic outlet and (3) in a line below the left pectoral muscles.

Upon entering the control room (Zone III) the MRI technician screens participants again for any metals in or on their body, using a metal detector. The study coordinator attaches the MRI-safe 3-lead electrocardiogram to the preplaced leads before entering the scan room (Zone VI). In the scanner, participants hold a button box, for an active visual matching task, and an alarm squeeze ball to be used in case of emergency. Participants are set up with blankets and a knee bolster for lower back support. Finally, the MRI technician completes the 64-channel head coil setup. The study coordinator asks participants to rate their nervousness and scanning begins.

Once in the scanner, participants rate the volume of a series of tones played through the headphones to ensure they can hear the audio when the scanner is in operation. The study coordinator remains in communication with participants during the entirety of the scan through headphones. After most scanning blocks, participants rate their discomfort level. The study coordinator collects two saliva samples, after the resting state 1 scan and DTI scans sequence 1-2, and immediately places them on ice. After saliva samples are collected, participants complete a series of affect self-report questions verbally. Functional scans which require participant engagement include the multisensory scan, N-back task 1 and 2, speech prep, and modified cold pressor.

When neuroimaging procedures are complete, the study coordinator and MRI technician deinstruments participants. Participants change back into their clothes and offered a break.

The study coordinator and participant meet in a closed room for debriefing and take-home sample instructions. Debriefing includes an explanation of the speech task on Day 1 and Day 2, all additional participant questions regarding the study are answered in this time. Finally, the study coordinator provides detailed instructions for take-home sample collection.

Starting the Monday after their in-person visit (i.e., 5 days after the on site visit), participants complete take-home sample collections. Participants take an iPad with custom-built MiSBIE Study app home to ensure a smooth and timely sample collection week and daily questionnaire completion. On all five days (Monday – Friday), participants complete a morning and evening questionnaire addressing daily mood and a sleep diary. On three days (Monday, Wednesday, and Friday) participants collect four daily saliva samples (awakening, +30 min, +45 min, bedtime) for a total of 12 samples. Saliva samples are stored in the freezer immediately after collection. A fecal sample is completed at any point over the sample collection week. An Actiwatch activity tracker is worn throughout the collection week to estimate physical activity levels and sleep patterns.

To finalize their participation in the study, participants mail all samples and devices (at room temperature) to the CUIMC lab in a pre-stamped envelope. Once samples and devices are received, their compensation is transferred, and the MiSBIE visit is complete.

Controlled study conditions and design elements:

Six main elements of the MISBIE study design contributed to enhancing data reliability: 1) Participants resided at a local hotel for the two nights preceding the hospital visits. This standardizes the novelty and transport time on visit days across all local and out-of-town participants. 2) Participants were instructed to avoid any form of exertion and were sent a car service to minimize exertion on the morning of visit days. 3) The protocol was highly standardized, a measure enforced using a detailed script for the study coordinator, a procedural checklist, and consistent environmental conditions ensured consistency across visits. 4) At least 30 minutes of rest was imposed before collecting blood, saliva, and physiological measurements. The main blood draw on Tuesday morning was performed in the fasting state (overnight fast since Monday 8:00 PM), standardizing physiological conditions across all participants. 5) Participants were fed at specified times during the protocol (~10:00 AM breakfast and ~12:00 PM lunch) from a predefined menu, preventing large deviations in meal macronutrient composition that could confound other measures. 6) All biospecimens were processed immediately after collection (e.g., blood was centrifuged within <1min after draw and stored on ice until freezing) to maximally preserve the metabolite, protein composition, or immune cell state of the participant's blood at the time of draw. 7) Samples for sensitive biomarkers (enzyme activities, metabolites) were stored in liquid nitrogen to achieve maximal biomarker stability over the duration of the study.

Detailed Methods

1 Screening Procedures

1.1 Recruitment

Background:

Recruitment took place over a 5-year period, from June 2018 to June 2023 from the following sources: Columbia University Irving Medical Center Neuromuscular Clinic (Dr. Hirano) and an ongoing natural history study (Kaufmann, 2011), the North American Mitochondrial Disease Consortium (NAMDC), the United Mitochondrial Disease Foundation (UMDF), the Champ Foundation, previous participant registries within Columbia, CUIMC community, and Columbia RecruitMe.

Procedure:

Potential participants are contacted by either the study coordinator or genetic counselor or contact the study coordinator directly by phone or email. If they are interested in learning more about the MiSBIE study, the study coordinator shares useful forms and information and schedules a phone screening call.

1.2 Phone Screening

Materials:

- **Telephone Interview for Cognitive Status (TICS).** (Psychological Assessment Resources, 5180-KT, Lutz, FL)

Background:

Screening calls ensure the potential participant meets all inclusion and exclusion criteria for study participation.

Procedure:

Participants are screened via phone using a phone screening script, this ensures consistency between screening calls. Prior to the screening call, the study coordinator receives potential participant contact information through Recruit Me, from the clinical team, or directly from participants. The study coordinator begins by obtaining verbal consent for the screening process. After approximately 10 minutes of screening questions, the study coordinator provides an overview of all study procedures and logistics.

CRITERION	METHOD OF ASCERTAINMENT
**Inclusion:**	
1. Individuals between the age of 18- 60	Patient clinical records<br>Phone screen or clinical evaluation
2. Willing to provide saliva samples and have intravenous catheter installed for blood collection during the hospital visit	Phone screen or clinical evaluation
3. Willing to provide informed consent and capacity to consent	Self-report
4. Use of effective method of birth control for women of childbearing capacity	Phone screen or clinical evaluation

5. English Speaking	Self-report, Phone screen or clinical evaluation
**Exclusion:**	
1. Individuals with cognitive deficit incapable of providing informed consent will not be included	TICS scores $\geq$ 30, administered via phone screen or clinical evaluation
2. Symptoms of flu or other seasonal infection four weeks preceding hospital visit, as this would influence immune system parameters	Phone screen or clinical evaluation
3. Raynaud's syndrome (Rayneau phenomenon)	Phone screen or clinical evaluation
4. Involvement in any therapeutic trials listed on clinicaltrials.gov, including exercise	Phone screen or clinical evaluation
5. Metal inside or outside the body or claustrophobia prohibitive to MRI testing	Phone screen or clinical evaluation
6. Diagnosed with mitochondrial disease m.3243A>G, or large scale mtDNA deletion	Phone screen or clinical evaluation

2 Physiological Measures

2.1 Resting Blood Pressure and Heart Rate

Materials:

- **Blood Pressure Monitor**. (BpTRU, SKU:BPM-300-45: BpTRU, Coquitlam, BC)

Background:

Blood pressure and heart rate are important vital signs and indicators of cardiovascular function frequently used in clinical and psychobiology studies. There is growing evidence that chronic psychosocial stress may lead to hypertension and that even the thought of stressful events can contribute to a delay in blood pressure recovery (Spruill, 2010).

Procedure:

To account for intra-individual variation in both heart rate and blood pressure, the study coordinator collects three measurements in succession and then averages these readings for a reliable, average reading. The appropriate cuff band (small, medium or large) is placed one inch above the elbow on the right upper arm. Participants sit in a chair with a back, to provide core support, and sits upright with legs uncrossed and arms placed on the armrest. The study coordinator instructs participants to rest for 5 minutes with no phone or other entertainment. Participants are instructed to avoid speaking during the data collection period and to expect the study coordinator to speak very little, if at all. After the 5-minute rest period, the study coordinator collects three consecutive blood pressure and heart rate measurements.

Outcome measures:

- Resting systolic blood pressure (SBP)
- Resting diastolic blood pressure (DBP)
- Resting heart rate (HR)

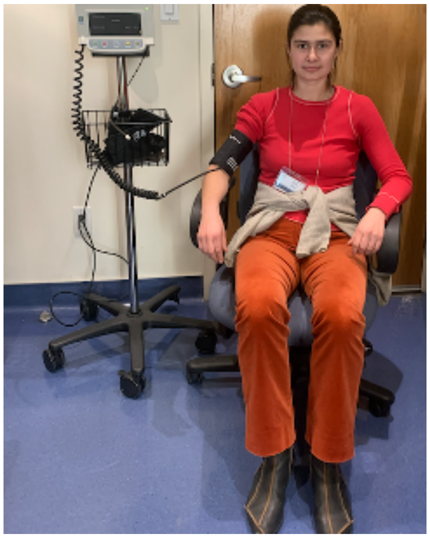

2.2 Height and Weight

Materials:

- **Mechanical Column Scale with Eye-Level Beam.** (seca, SKU:CE 0123, Chino, CA)

Background:

Height and weight are essential measurements to determine an individual's body mass index (BMI). Body mass index is used to imperfectly but objectively classify individuals into groups such as underweight, overweight, or obese (Khanna et al., 2022) These classifications can serve as a tool to predict disease risk.

Procedure:

Participants remove their shoes before height and weight are assessed. The study coordinator collects height and weight using a physician beam scale.

Outcome measures:

- Height (cm)
- Weight (pounds)

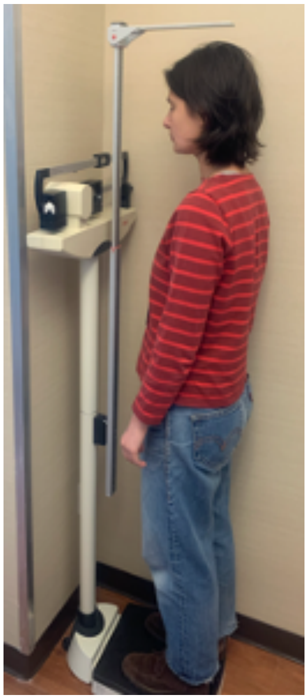

2.3 Hip and Waist Circumference

Materials:

- **MyoTapeBody Measure Tape.** (AccuFitness, SKU:BC-1500, Denver, CO)

Background:

Hip-waist circumference ratio can be a useful predictor of health outcomes including Type 2 diabetes and cardiovascular disease, and is often considered a more reliable indicator of metabolic health than BMI (WHO, 2008).

Procedure:

Participants remove any excess, baggy clothing and points to their belly button. To obtain waist circumference, the study coordinator wraps the tape around the smallest point of the torso, usually at the belly button. To obtain hip circumference, the study coordinator wraps the tape around the hips at the widest point (Hou et al., 2019).

Outcome Measures:

- Waist circumference (inch)
- Hip circumference (inch)
-

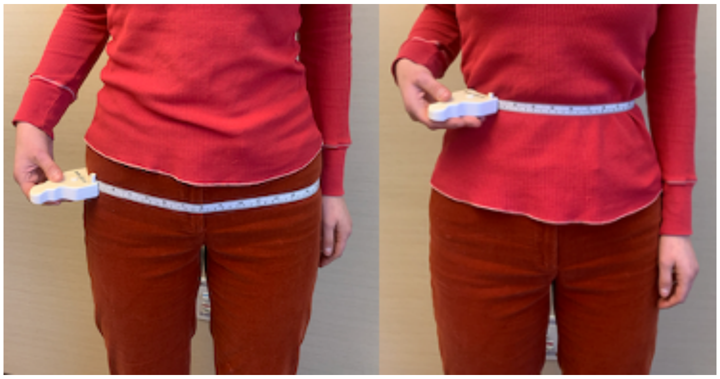

2.4 Body Composition

Materials:

- **Body Composition Scale.** InnerScan PRO, Multi-Frequency, Segmental, Wireless, Body Composition Monitor (Tanita, SKU: MT05, Arlington Heights, IL)

Background:

Body composition provides meaningful information about the relative contribution of fat mass or lean mass that makes up an individual's total weight. Total fat mass and visceral fat area have been associated with higher mortality and are, therefore, clinically relevant (Kuriyan, 2018). It is particularly relevant to determine body composition in a sample of individuals with mitochondrial disease due to the high energy demands and low body weight characteristic of this population (Hou et al., 2019).

Procedure:

Participants remove their shoes and stand on the body composition scale while holding the hand grip electrodes. The study coordinator records body fat percentage twice for a reliable, average measurement.

Outcome Measures:

- Body fat percentage

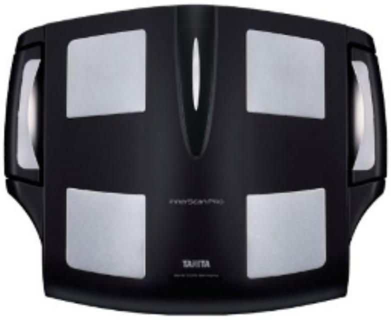

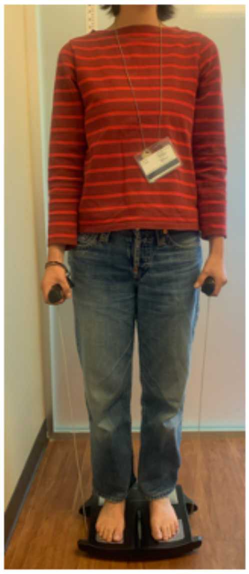

2.5 Temperature

Materials:

- **Non-Contact Infrared Thermometer**. (Norwood, SKU: NOR-SCH1005, Gilbert, AZ)

Background:

Thermoregulation is an essential tool in maintaining homeostasis, failure to maintain an optimal core body temperature can lead to serious illness and death(EV Osilla 2022). Thermoregulation is energy demanding so it is relevant to consistently monitor core body temperature in a sample of individuals with diverse mitochondrial energy production capacities.

Although core body temperature measurements can be difficult to gather with non-invasive methods, such as an infrared thermometer, taking multiple measurements, rather than relying solely on one temperature reading, allows for more precision (Patel et al., 1996). There is some evidence for altered thermoregulation in people with depression, further evidence is needed to investigate the relationship between temperature regulation and mental state (von Salis et al., 2021).

Procedure:

The study coordinator gathers temperature readings using a no-touch infrared thermometer in surface mode at three points (left hand, lower esophageal, and the tip of the tongue) maintaining a distance of 1-2 inches from the skin. Tongue measurements are taken immediately after participants open his or her mouth to avoid ambient cooling. Right-hand temperature is also taken before, immediately after, and ten minutes following the cold pressor task. The study team regularly calibrates the thermometer using an ice bath.

Outcome Measures:

- Left-hand temperature
- Lower esophageal temperature
- Tip of the tongue temperature
- Right-hand temperature (cold pressor task only)

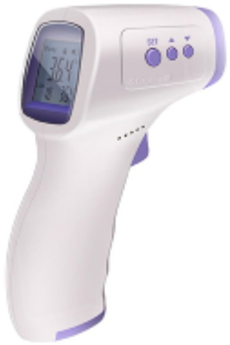

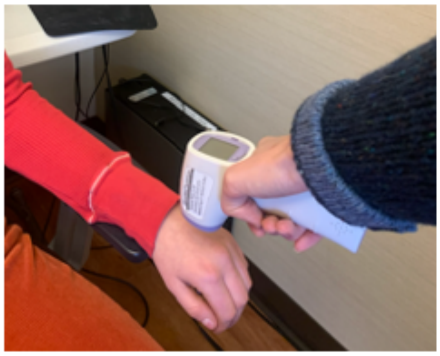

2.6 Spirometry

Materials:

- **Micro I Spirometer.** (Cafefusion SKU#MICRO I, San Diego, CA).
- **Spirometer Calibration Syringe 3L. (**Medical International Research (MIR), Catalog Number #HM45381, Roma, RM, Italy)
- **Disposable Mouthpiece of Digital Spirometer.** Vitalograph SafeTwat One-way Mouthpieces (Vitograph, Catalog Number #20242, Lenexa, KS, USA).
- **Disposable Nose Clips.** Midmark Disposable Nose Clips (Midmark, #2-244-0001, Dayton, OH, USA)

Background:

Spirometry is a standard test of pulmonary function. This non-invasive test includes measurements of volume, time, and flow (Moore, 2012). Spirometry measurements include the following: FEV (forced expiratory volume), FVC (forced vital capacity), FEV1/FVC (ratio). Obstructive or restrictive lung function has been associated with greater mental health, well-being, and general health disturbances (Renee D Goodwin 1, 2007).

Procedure:

Prior to spirometry measurement, the study staff notes participants' age, height, and sex in the spirometry device; the spirometry device provides estimated values for FEV, FVC, and FEV/FVC based on this information. The study coordinator calibrates the spirometer prior to each use; using a 3L calibration syringe, a standard volume of air is pushed into the spirometer. The spirometer should record within 3% of the known 3L volume (Moore, 2012).

Proper participant instruction is vital for accurate measurements. Repetition of instructions is likely necessary between measurements, a total of three measurements are recorded. Participants must be instructed by the operator (study coordinator) to sit upright with feet flat on the floor, uncrossed (Graham et al., 2019). The operator should instruct participants to breathe normally as instructions are given. Participants must inspire completely and rapidly, they are told to take as deep of a breath as possible and specifically given the prompt of, “more, more, more” as they inhale (Graham et al., 2019). The operator must ensure participants form a tight seal with their lips around the mouthpiece and expels their air with maximal effort. Participants are told to blast, not blow, their air out as hard and as fast as possible with active and enthusiastic coaching by the operator (Graham et al., 2019). After this first effort to expel air, the operator must actively coach participants to keep breathing until all air is expelled.

After the first attempt, the operator logs the results as participants return to a normal breathing pattern. The operator repeats instructions with vigorous coaching (Graham et al., 2019). If the first attempt results in a "low effort" reading, the measurement can be repeated. A total of three readings are recorded, and participants can complete a maximum of four attempts.

Outcome Variables:

- Forced expiratory volume (FEV)
- Forced vital capacity (FVC)
- Ratio (FEV1/FVC)

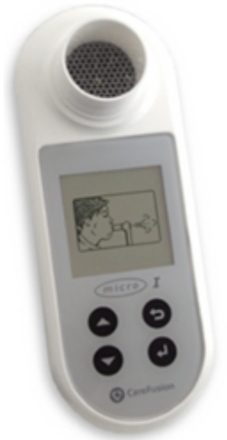

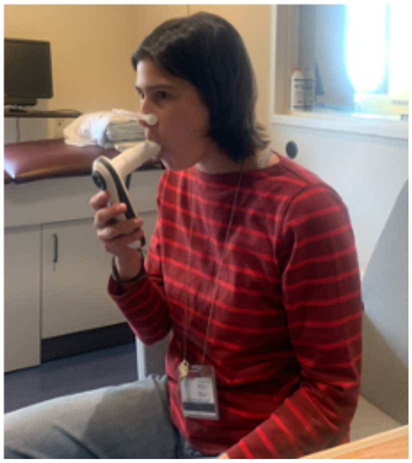

2.7 Heart Rate

Materials:

- **BlueSensor VL** (Ambu, VL-00-A/25, Ballerup, Denmark)
- **BioNex 8-Slot System** (BioNex, 50-3711-08, North Brunswick, NJ)

Background:

Heart rate (HR) and heart rate variability (HRV) are known physiological symptoms of an acute stress response (Schubert et al., 2009). Both heart rate and heart rate variability are sensitive to mental stressors; these measures have the potential to measure a stress response (Joachim Taelman, 2009).

Procedure:

The BioNex system records ECG output via three BlueSensor ECG electrodes placed at the midline sub clavicular space on the right and left shoulders, and the lower left abdominal quadrant, sampled at 500 Hz. The ECG waveform is submitted to an R-wave detection software resulting in an RR interval series. Trained research assistants correct errors in marking R waves by visual inspection and ectopic beats by interpolation. To derive a continuous sampling of HR, investigators apply the following equation: HR=60/(RR interval). A Fourier transformation algorithm, similar to that used by DeBoer, Karemaker, and Strackee is applied to the RR time-series to derive frequency-domain heart rate variability (DeBoer et al., 1984; Schubert et al., 2009) . The investigators set frequency ranges following the guidelines recommended by the HRV Task Force of the European Society of Cardiology and the North American Society of Pacing and Electrophysiology ; Very low-frequency HRV <.04 Hz, Low-frequency HRV = .04-.15 Hz, High-frequency HRV = .015-.4 Hz (Anon, 1996).

Outcome Variables:

- Heart rate
- Heart rate variability
-

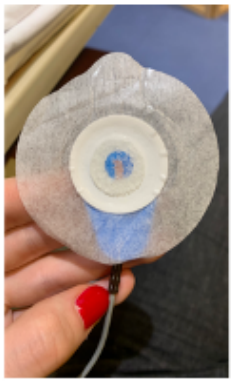

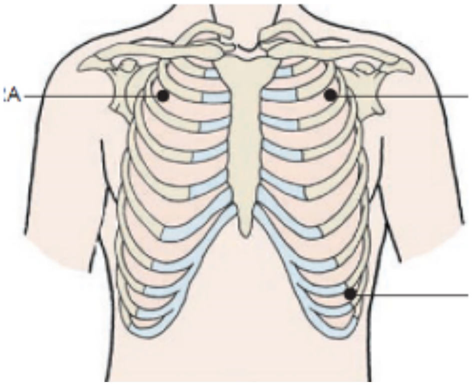

2.8 Electrodermal Activity

Materials:

- **GSR/GSC Electrodes** (MindWare, 93-0102-00, Westerville, OH)
- **BioNex 8-Slot System** (BioNex, 50-3711-08, North Brunswick, NJ)

Background:

Electrodermal activity (EDA) is a reliable biomarker of arousal and part of the sympathetic nervous system response (George I. Christopoulos, 2016). Therefore, it is relevant to track changes in skin conductance during an induced stress task. Electrodermal activity has been shown to be a reliable tool for the classification of mental stress levels (Rahma et al., 2022).

Procedure:

The BioNex system recorded EDA output via two GSC electrodes on the left palm. Skin conductance signal is measured in microsiemens (uS). The variables listed below were measured using Electrodermal Activity Analysis Software from Mindware Technologies.

Data Quality Assurance Procedure:

Of the 110 participants included in the study, *X* participants had no missing data, *X* participants had some missing data, and *X* participants had no data. Reasons for missing data varied, but most common were 1) period not run due to technical issues, 2) loss of signal due to faulty electrodes, or 3) excessive amounts of noise due to faulty electrodes. For periods of otherwise clean data in which there were brief, easily identifiable sections of noise, manual removal of misidentified peak-trough pairs was performed.

Subsequent to initial pre-processing, internal validity of the dataset was assessed by modeling two key outcome measures: SCR Response (the total change in uS throughout a given period) and Total SCRs (the number of peak-trough pairs throughout a given period). First, for each outcome measure, correlation matrixes between all periods for all participants were generated. For both measures, we expected to see moderate-to-high R-values across periods, indicating that a given participant tended to show relatively consistent patterns of physiology throughout the study visit. For cases in which the R-

value was low (>0.15), the correlation was then visualized as a scatterplot in order to identify individual outliers. Identified outliers were then manually re-inspected in the Mindware program, and either manually cleaned or excluded if they were determined to contain non-physiological data.

Outcome Variables:

- Total Skin Conductance Responses (SCRs)
- Tonic Skin Conductance Level (SCL)
- Mean Skin Conductance (SC)
- Tonic Period
- Maximum
- Minimum
- SCR Response
- Trough SCL (single peak)
- Peak SCL (single peak)
- SCR (single peak)
- Recovery Time (single peak)

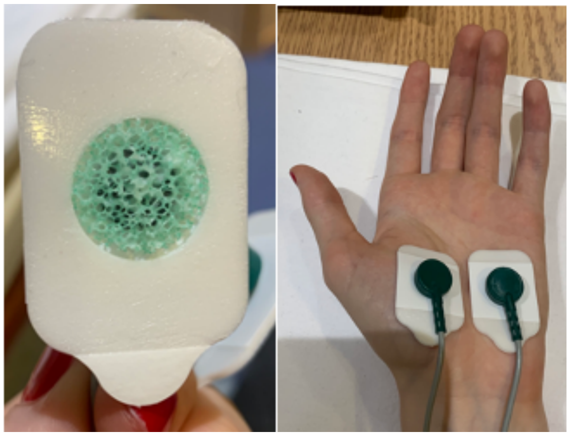

2.9 Respiratory Rate

Materials:

- **Transducer Bands** (Ambulatory Monitoring, Inc.,10.5032, Ardsley, NY, USA)
- **Inductotrace Portable Respiratory Monitor** (Ambulatory Monitoring, Inc., PRM-131L, Ardsley, NY, USA)
- **Inflatable Spiro Bags** (Ambulatory Monitoring, Inc., 10.4028, Ardsley, NY, USA)
- **BioNex 8-Slot System** (BioNex, 50-3711-08, North Brunswick, NJ)

Background:

Respiratory rate is a sensitive vital sign highly responsive to stress (Nicolo et al., 2020). To best understand an acute stress response, respiratory rate analysis is necessary. Mental states, including anxiety, have been associated with increased respiratory rate (Zamoscik et al., 2020).

Procedure:

The BioNex system records respiration from the Inductotrace via the transducer bands placed at thoracic and abdominal positions, sampled at 20 Hz. The RSP waveform is submitted to a signal peak-and-trough detection software, resulting in a time series of inspiration and expiration events. Breath

volume is calibrated in a seated and standing position using five breaths into and out of the Spiro Bags (X L) to convert analog signal into breathing volume.

Outcome Variables:

- Respiratory rate
- Respiratory waveform

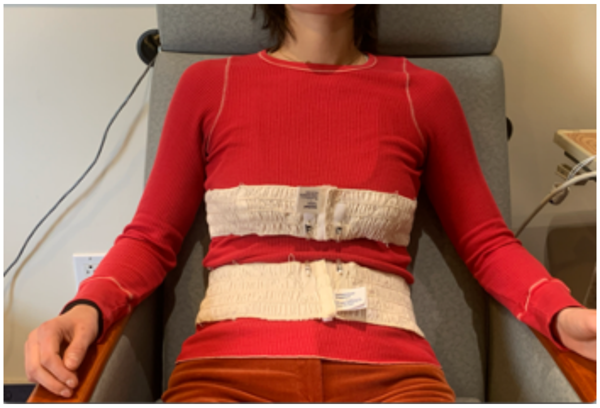

2.10 Blood Pressure

Materials:

- **BMEYE Nexfin Continuous Blood Pressure Monitor.** (Edwards Lifesciences Corp., Irvine, CA, USA)

Background:

The Trier Social Stress Task, a version of which is completed in this study, reliably activates the hypothalamic-pituitary-adrenal (HPA) axis in most participants (Frisch et al., 2015), leading to a change in blood pressure. Heightened or blunted blood pressure reactivity to acute stress can provide meaningful insights into an individual's health (Wright et al., 2014). Chronic stress can contribute to negative health outcomes, including hypertension, and alter long-term stress reactivity patterns, including delayed recovery (Spruill, 2010).

Procedures:

The BioNex system records BP from the BMEYE Nexfin system, via a finger cuff applied to the midphalanx of the left middle finger, sampled at 500 Hz. The waveform is submitted to a signal peak-and-trough detection software resulting in a time series of systolic and diastolic arterial pressure events.

Outcome Variables:

- Systolic blood pressure
- Diastolic blood pressure

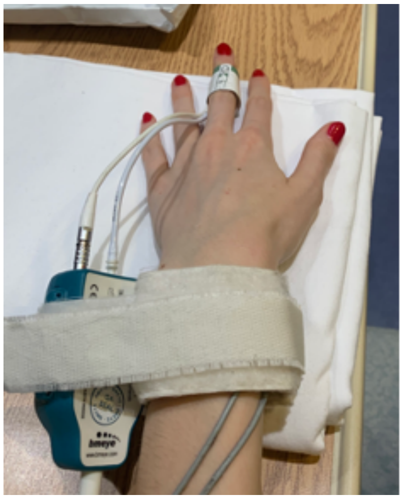

2.11 Resting Metabolic Rate

Materials:

- **Reevue Metabolic Rate Analysis System**. (Korr Medical Technologies, CPT#94690, Salt Lake City, Utah)
- **Bacterial Viral Filter**. (Korr Medical Technologies, 9FG0079, Salt Lake City, UT)

Background:

Mitochondrial diseases are associated with impairments in oxidative phosphorylation (OxPhos) which can trigger hypermetabolism(Sturm et al., 2023). The Reevue device measures resting metabolic rate by analyzing oxygen consumption and using standard algorithms to convert this to metabolic rate.

Procedure:

The study coordinator instructs participants to breathe through a disposable tube with a nose clip for the duration of the analysis. The study coordinator provides instructions to breathe normally through the one-way valve for ten minutes while they remain relaxed and still in a comfortable chair. After the ten-minute analysis is complete, participants note if they felt air escaping from the mouthpiece and if they ever took the mouthpiece out of their mouth. In addition, the study coordinator notes whether participants move during the analysis.

Outcome Variables:

- Resting Energy Expenditure (REE) (kcal/day)
- VO_2 (mlO_2/min)
- FeO_2 (%)
- Tidal volume (L)
- Minute ventilation VE (L/min)
- Respiratory rate (breaths/min)
- *Calculated*: Weight-adjusted VO_2 (mLO_2/min/kg)
- *Calculated*: Fat free mass-adjusted VO_2 (mLO_2/min/kg_ffm)

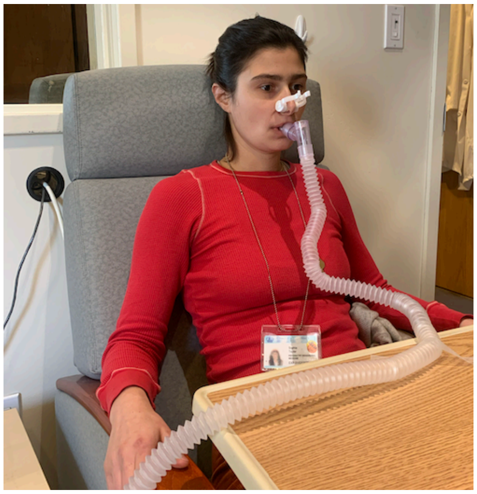

3 Biospecimen Collection

3.1 Morning Blood Draw

Materials:

- **ACD Solution A Vacutainer**. (Becton, Dickinson and Company, BD-364606, Franklin Lakes, NJ)
- **Buffered Na Citrate Vacutainer**. (Becton, Dickinson and Company, BD-366560, Franklin Lakes, NJ)
- **K2 EDTA Vacutainer**. (Becton, Dickinson and Company, BD-366643, Franklin Lakes, NJ)
- **Serum Separating Tube**. (Becton, Dickinson and Company, BD-36786, Franklin Lakes, NJ)
- **Serum Vacutainer.** (Becton, Dickinson and Company, BD-36815, Franklin Lakes, NJ)
- **Z Serum Clot Activator**. (Greiner Bio-One North America, 455092, Monroe, NC)

Background:

Blood is sampled for the following analyses: standard blood chemistry, mitochondrial phenotyping, mtDNA mutation/deletion heteroplasmy, allostatic load, DNA methylation, metabolomics, proteomics, pro- and anti-inflammatory cytokines, and other circulating biomarkers of interest.

Morning Procedure:

Participants fast starting at midnight the night before sample collection ($\geq$ 10 hours fasted). Blood is extracted by intravenous catheter in the antecubital vein and collected at ~9:50am. Vacutainers are filled in the following standardized order.

Tube Type

# Tubes	Vacutainer
5	2.7ml Sodium Citrate
1	3ml Lavender K_2EDTA
2	5ml Serum Separation
5	8.5ml Yellow ACD-A
2	8 ml Red (no additive)
2	8ml Purple

Draw Order

Vacutainer
2.7ml Sodium Citrate
3ml Lavender K_2EDTA
5ml Serum Separation
5ml Serum Separation
8.5ml Yellow ACD-A
8.5ml Yellow ACD-A
8.5ml Yellow ACD-A
8.5ml Yellow ACD-A
8.5ml Yellow ACD-A
2.7ml Sodium Citrate
2.7ml Sodium Citrate
8 ml Red (no additive)
8ml Purple EDTA
2.7ml Sodium Citrate
2.7ml Sodium Citrate
8 ml Red (no additive)
8ml Purple EDTA

Outcome Variables:

- Fasting timepoint specimen collection
- Serum and Plasma collection

- Platelet collection
- Purified PBMC collection
- Purified Leukocyte Subtypes collection (see below for Laboratory Procedures)

3.2 Afternoon Blood Draw

Materials:

- **K2 EDTA Vacutainer**. (Becton, Dickinson and Company, BD-367899, Franklin Lakes, NJ)
- **Serum Vacutainer.** (Becton, Dickinson and Company, BD-367820, Franklin Lakes, NJ)
- **K2 EDTA Vacutainer**. (Becton, Dickinson and Company, BD-366643, Franklin Lakes, NJ)
- **ACD Solution A Vacutainer**. (Becton, Dickinson and Company, BD-364606, Franklin Lakes, NJ)

Background:

Blood is sampled for the following analyses: standard blood chemistry, mitochondrial phenotyping, mtDNA mutation/deletion heteroplasmy, allostatic load, stress reactivity, DNA methylation, metabolomics, proteomics, pro- and anti-inflammatory cytokines, and other circulating biomarkers of interest.

Procedure:

Blood is collected via an intravenous catheter and collected in vacutainer tubes throughout the afternoon in the right forearm. Blood samples are collected prior to and after a stress-inducing task. Blood is collected in the following order throughout the afternoon.

Timepoint	Vacutainer	Sample Collected
**-5**	5 ml Red	Ser-S-1
	5 ml Purple EDTA	Pl-S-1
**+5**	5 ml Red	Ser-S-2
	5 ml Purple EDTA	Pl-S-2
**+10**	5 ml Red	Ser-S-3
	5 ml Purple EDTA	Pl-S-3
**+20**	5 ml Red	Ser-S-4
	5 ml Purple EDTA	Pl-S-4
**+30**	5 ml Red	Ser-S-5
	5 ml Purple EDTA	Pl-S-5
**+60**	5 ml Red	Ser-S-6
	5 ml Purple EDTA	Pl-S-6
**+90**	5 ml Red	Ser-S-7
	5 ml Purple EDTA	Pl-S-7
**+120**	5 ml Red	Ser-S-8

	5 ml Purple EDTA	Pl-S-8
	8.5ml Yellow ACD-A	PBMC isolation
	3ml Lavender K_2EDTA	CBC

Outcome Variables:

- Stress timepoint specimen collection
- Serum and Plasma collection
- Purified PBMC collection and cryopreservation

3.3 Saliva Sample

Materials:

- **Salivette** (Starstedt, Cat# 51.1534.500, Numbrecht, Germany)

Background:

Free cortisol is measured in saliva (Kirschbaum & Hellhammer, 1994). Mitochondria also release their genetic material as cell-free mitochondrial DNA (cf-mtDNA) in biofluids including blood and saliva under acute stress (Trumpff et al., 2022). Saliva samples collected non-invasively may be used to probe stress physiology and to develop biomarkers of health.

Procedure:

Participants place the cotton salivette in their mouth, in the middle of their tongue, for 2-5 minutes without biting on the swab or moving it. After two minutes, the study coordinator determines if the salivette is saturated or requires further time to collect a sufficient sample. After sample collection, the study coordinator immediately places the salivette on ice.

Outcome Variables:

- Salivary steroid hormones (cortisol, cortisone, DHEA, testosterone, progesterone, corticosterone)
- Salivary cf-mtDNA
- GDF15
- Proteomics
- Other biomarkers

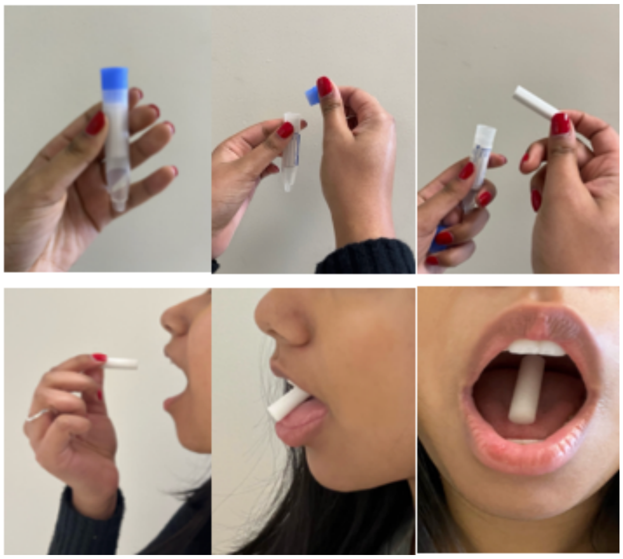

3.4 Buccal Swab

Materials:

- **Histobrush.** Puritan Histobrush Cytology Collection Device (Puritan, SKU: 252188, Guilford, ME)

Background:

Collected material will enable to measurement of mtDNA copy number, DNA methylation, and mtDNA mutation heteroplasmy in a somatic tissue. Buccal swab sampling is an effective, non-invasive alternative to traditional tissue sampling methods (Acin-Perez et al., 2021; Yuri Masaoka, 2001)

Procedure:

A cheek swab of the internal mucosa from both cheeks is performed to collect mtDNA. The study coordinator performs the cheek swab (3 down-and-up swipes on each side, total 6 swipes). This procedure takes approximately ten seconds and is painless.

Outcome Variables:

- mtDNA mutation heteroplasmy
- mtDNA copy number
- DNA methylation

3.5 Hair Collection

Materials:

- **Nylon Weaving Thread, Catalog Number #10557501.** Strong Upholstery Thread (Coats and Clark, Greer, SC, USA)is prepared with a slip knot prior to hair sample.

- **8.8 Inch Tail Comb with Pick**. (Fromm International, Catalog Number #D7150) Pick end is used to isolate hair samples.
- **Stainless Steel Hair Scissors. (SurgiDental, Catalog Number #B01JF6AWSY)**
- **Aluminum Foil. (Reynolds. Catalog Number #B0014D5OA8)**

Background:

Hair steroid hormones analysis has been determined to be a reliable measurement of steroid hormone exposure over an extended period of time (Russell et al., 2012). Hair growth rate is approximately 1 cm /month; analysis of hair can therefore approximate steroid hormone production over months and even years (Russell et al., 2012). The study coordinator is trained to take two samples of hair from the posterior vertex region of the scalp. In total, a minimum of 20 mg of hair or a 3cm segment is collected (Gao, et al). The hair sampling method is based on the guidance of MiSBIE collaborator Dr. Clemens Kirshbaum (Kirschbaum, 2017).

Procedure:

The study coordinator clips back excess hair to get a clean sample from the posterior vertex region. Two small lines of hair are isolated by placing a loop of thread with a slipknot around the chosen samples. Once the thread is tightened and the hair samples are secure, the study coordinator cuts the hair as close to the scalp as possible. The hair samples are placed on a piece of aluminum foil and, if necessary, they are secured using a paperclip. The scalp side of each sample is noted. The aluminum foil is folded to keep the sample secure and completely covered. The aluminum package is then placed into a Ziploc bag with participants' unique identification number. All hair samples are kept in a safe with no access to light and kept at room temperature prior to shipment for analysis (Kirschbaum, 2017).

Outcome Variables:

- Hair steroid hormones (cortisol, cortisone, DHEA, testosterone, progesterone, corticosterone)

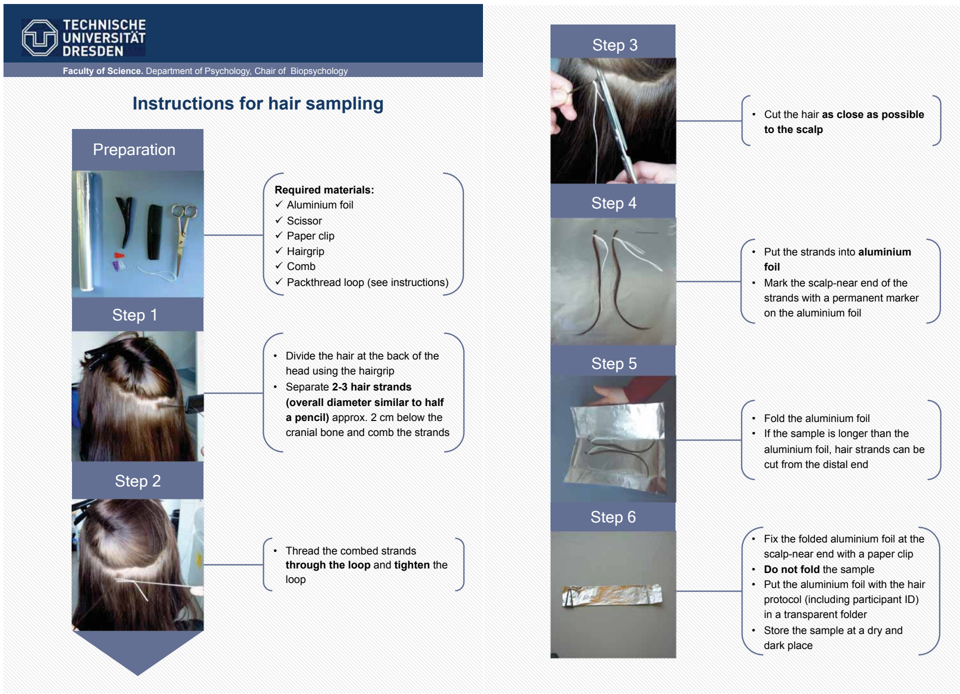

3.6 Urine Collection

Materials:

- **Urine Collection Container.** 24 HR Collection Container with 30 mL Acetic Acid (Therapak, #38533G, Claremont, CA)

Background:

Urinalysis serves as a diagnostic tool that can provide clinically relevant information regarding health status.

Procedure:

At the end of Day 1, participants are given a urine container to take urine samples at the hotel between 8pm-8am. Participants are provided with either a disposable cup(male) or a urine hat(female) and are instructed to urinate in the cup/hat and then pour their contents into a larger container to avoid any potential skin contact with the preservative. The collection container is sealed in a plastic bag to prevent an undesirable odor and transported in an opaque tote bag to minimize any discomfort that could be associated with this task. An instruction sheet is provided to all participants for reference.

Outcome Variables:

- Cell free urine collection
- Urine epithelial cell collection

INSTRUCTIONS FOR URINE COLLECTION

With the container provided, collect urine overnight over a period of 12 hours.
This will start tonight at **8:00pm**, until to **8:00am** tomorrow morning.

Starting at 8:00pm, please **use cups to collect the urine every time you go to the bathroom tonight**.

Please write down below the time of your **first** and **last** urine collection.
First: __ : __ __ pm *(should be around 8:00pm)*
Last: __ : __ __ am *(should be around 8:00am)*

IMPORTANT: Please do not urinate directly into the provided urine container because it includes preservatives. **Use the cups to urinate, and then pour the content of the cups into the container.**

Always make sure that the lid is securely screwed to the container to avoid spillage.

REMINDERS:
Keep the container in the bathroom between the sink and toilet.
*Place **cups** ON top of the toilet seat after each collection.*

After collecting your last sample around 8:00am, place the container into the **Ziplock bag** and use the fabric bag provided to transport the container. Hand in the container to the study coordinator.

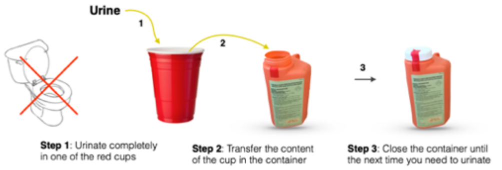

INSTRUCTIONS FOR URINE COLLECTION

With the container provided, collect urine overnight over a period of 12 hours.
This will start tonight at **8:00pm**, until **8:00am** tomorrow morning.

Starting at 8:00pm, please **use the urine hats to collect your urine every time you go to the bathroom tonight**.

Please write down below the time of your **first** and **last** urine collection.
First: __ : __ __ pm *(should be around 8:00pm)*
Last: __ : __ __ am *(should be around 8:00am)*

IMPORTANT: Please do not urinate directly into the provided urine container because it includes preservatives. **Use the urine hat to urinate, and then pour the content of the hat into the container.**

Always make sure that the lid is securely screwed to the container to avoid spillage.

REMINDERS:
Keep the container in the bathroom between the sink and toilet.
*Place **urine hats** UNDER the seat, or ON the toilet cover after each collection.*

After collecting your last sample around 8:00am, place the container into the **Ziplock bag** and use the fabric bag provided to transport the container. Hand in the container to the study coordinator.

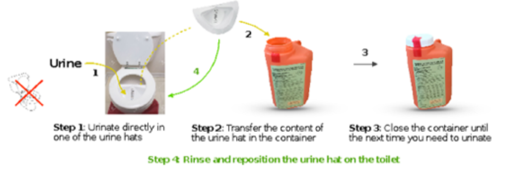

4 Biospecimen Processing

Below are the detailed methods used to process and store all biospecimens in the MiSBIE Biobank. The step-by-step, illustrated laboratory protocol is available in **Supplemental Material 12.1**.

Background:

Rapidly processing biological fluids by centrifugation is used to isolate specific components from whole blood including serum, plasma, PBMCs, leukocyte subtypes, and saliva. These components are used to measure biological parameters of interest.

4.1 Fasting Serum Processing

Materials:

- **Sorvall ST 16R refrigerated centrifuge** (ThermoFisher Scientific, catalog 75004381)
- **Centrifuge 5702**, low-speed room temperature (Eppendorf, catalog 5702000010)
- **15ml conical tubes** (Eppendorf, catalog 0030122151)
- **50ml conical tubes** (Eppendorf, catalog 0030122178)
- **Cryogenic storage tubes** (Millipore Sigma, catalog BR114841).

Procedure:

Blood is collected by intravenous catheter in the antecubital vein and collected in 2 X 8.0ml Serum Clot activator tubes (BD-367820) at ~10am. Upon collection, a 30-minute-timer is started, and blood is transported in a Styrofoam box to the laboratory (5 minutes' walk) for further processing. After 30min, the serum tubes are centrifuged immediately at 2,000 X G for 10 min at 4 °C and 80% of the serum from each tube is collected and pooled to new 15ml conical tube. The pooled serum tube is then centrifuged again at 2,000xg for 10 min at 4°C. 90% of the serum is then transferred to new 15ml tube, mixed by inversion and then aliquoted.

4.2 Fasting Plasma Processing

Materials:

- **Sorvall ST 16R refrigerated centrifuge** (ThermoFisher Scientific, catalog 75004381)
- **Centrifuge 5702**, low-speed room temperature (Eppendorf, catalog 5702000010)
- **15ml conical tubes** (Eppendorf, catalog 0030122151)
- **50ml conical tubes** (Eppendorf, catalog 0030122178)
- **Cryogenic storage tubes** (Millipore Sigma, catalog BR114841).

Background:

Processing of biological fluids provides isolation and collection of individual whole blood components including serum, plasma, PBMCs, leukocyte subtypes and saliva. These components are used to measure various biological parameters.

Procedure:

Blood is extracted by intravenous catheter in the antecubital vein and collected in 2 X 8.0ml (BD-366643) tubes at 10am from the left arm. Immediately after collection, samples are inverted 10-12 times and centrifuged at 1,000xg for 5 minutes at room temperature with breaks off and then placed on ice(4°C) in a Styrofoam box for transport to the laboratory (5 minutes walk). To avoid platelets and other cellular debris, approximately 80% of the plasma is collected from each tube and pooled into a fresh 15 mL conical tube. To further eliminate cellular components, pooled plasma tube is centrifuged at 2000xg for 10 minutes at 4°C. Around 90% of the resulting plasma supernatant is transferred to fresh 15 mL conical tubes and mixed through inversion and then aliquoted.

4.3 Whole Blood Processing

Immediately following blood collection:

Blood is extracted by intravenous catheter in the antecubital vein and collected in 5 X 8.5ml (BD-364606) tubes at 10 am from the left arm. Immediately after collection, samples are inverted 10-12 times. Blood tubes are placed in a Styrofoam box at room temperature for transport to the laboratory (5-minute walk). At the laboratory, blood is centrifuged 500xg for 15 minutes at room temperature with centrifuge brake off.

4.3.1 Platelet Isolation

Background:

- **15ml conical tubes** (Eppendorf, catalog 0030122151)
- **Prostaglandin I2** (Cayman Chemical, catalog 18220)
- **Centrifuge** 5702, low-speed room temperature (Eppendorf, catalog 5702000010)

Procedure:

After completion of the spin, 3ml of plasma is aspirated from each of the 5 ACD-A tubes and pooled in a 15ml conical centrifuge tube. To prevent platelet aggregation, 15 □l of Prostaglandin I2 (PGI2) (Cayman Chemical, #18220) at 1mM concentration is added to the tube (PGI2 final concentration at 1□M) The tube is centrifuged at 1000xg for 5 minutes at room temperature to pellet red blood cells (RBCs). After this spin, 90% of the supernatant is transferred to a new 15 ml tube and centrifuge at 1500xg for 10 minutes at room temperature. After this spin, the supernatant is discarded and the pellet is resuspended in the remaining liquid by gently tapping the tube against a closed fist. 10mL of PBS-PGI2 (1□M) buffer is drawn up in a serological pipette, 5mL is dispensed into the 15mL tube and then mixed via repeated aspirating/dispensing until the cell suspension is homogenous. Next the total volume is dispensed into the tube and centrifuged at 1500xg for 10 minutes at room temperature. After the spin, the supernatant is discarded, and the remaining platelets are resuspended in 3ml PBS-PGI2 buffer (1□M). Platelets are set aside at room temperature until counting.

4.3.2 Purifying PBMCs

Materials:

- **15ml conical tubes** (Eppendorf, catalog 0030122151)
- **1.5ml microcentrifuge tubes (**Eppendorf, 022364116)

- **Hank's Balanced Salt Solution** 1X (HBSS) (Gibco, catalog 14175103)
- **Histopaque 1077** (Sigma, catalog 10771)
- **Histopaque 1119 (Sigma, catalog 11191)**
- **10 ml serological pipette**
- **Centrifuge** 5702, low-speed room temperature (Eppendorf, catalog 5702000010)
- **Countess II FL Automated Cell Counter** (ThermoFisher Scientific, AMQAF1000)
- **Countess cell counting chamber slides** (ThermoFisher Scientific, catalog C12208)
- **Trypan blue vial (ThermoFisher Scientific,** T10282)
- **Centrifuge 5427 R, high-speed refrigerated (Eppenndorf, catalog 5429000133)**

Procedure:

After spinning the ACD-A tubes and aspirating the plasma (see *Platelet Isolation*), the leftover of the first collected ACD-A tube is poured into a 15ml conical tube. 5mL HBSS (Gibco, #14175103) is added to wash the ACD-A tube and to collect any remaining cells, and finally dispensed into the same 15ml tube. The diluted blood is aspirated and slowly layered over 4mL of Histopaque 1077 (Sigma, # 10771) preloaded in a 15ml conical tube. To achieve a gentle overlay, prior to dispensing blood, the blood wet serological pipette tip is used to draw an inverted "Y" shape on the tube wall – this branching slows the flow of blood and prevents the blood from mixing with the Histopaque 1077. The pipette tip is placed at the top of the "Y" shape, near the top of the 15mL Ficoll tube, before slowly dispensing the blood. It is crucial to maintain a constant flow of liquid while dispensing to prevent turbulence. Once the overlay is complete, the tube is moved gently to a centrifuge and spun at 400xg for 30 minutes at room temperature. To keep the gradient intact during certification the centrifuge must be set to **"break off".** After centrifugation, mononuclear cells and platelets can be seen as a thin whitish layer between the pale yellow plasma and the clear Histopaque. Around ¾ of the diluted plasma is discarded. A wide bore 1 ml pipette is used to collect a total of 3 ml of the cell layer and transferred to a 15 ml conical tube pre-filled with 2 ml HBSS. After cell layer collection, the tube is filled to 15ml with HBSS and centrifuged at 500xg for 10 minutes at room temperature to pellet cells. After this spin, the supernatant is discarded by pouring and the tube is taped against a closed fist to resuspend the cell pellet with any remaining liquid. The tube is filled again to 15mL with HBSS, carefully inverted, and centrifuged at 200xg for 10 minutes at room temperature. The supernatant is discarded again, cells are resuspended by tapping the tube, filled to 15 ml with HBSS and centrifuged for a third and final time at 200xg for 10 minutes at room temperature.

After 5 minutes of the third spin, Trypan blue vial is placed in a bead bath to warm. After spin, the supernatant is discarded and the tube is tapped to resuspend the pellet in any remaining liquid. The cell suspension is resuspended in 1 ml of HBSS. For cell counting, 10□l of trypan blue is mixed with 10□l of cell suspension and 10□l of the suspension is loaded into ports A and B of the Countess cell counting chamber slide. Cell count is performed on Countess II (Invitrogen, #AMQAF1000) automated cell counter in bright field. Live cell number, cell size, and percent cell death are recorded for both ports. After averaging total live cell count for both ports and averaging the count, the percent difference is calculated. A criteria of a percent difference of 10 or less was established to ensure accurate live cell count. Once the percent difference is confirmed to be within the established threshold, cell aliquots of 5 million cells is calculated based on the average live cell count and stored as fasting PBMC aliquot labeled F.1. The remaining cell suspension volume is measured, cell count calculated based on average count and cryopreserved as stress reactivity PBMC aliquot labeled F.2. Aliquots are centrifuged at 2000xg for 2 minutes in a pre-chilled micro-centrifuge at 4°C. The supernatant is aspirated as much as possible without

disrupting the pellet and stored in a -80°C freezer before transferring it to long-term storage in liquid nitrogen. Any leftover cells are cryopreserved as described below (4.3.3 *PBMC cryopreservation).*

4.3.3 PBMC Cryopreservation

Materials:

- **Centrifuge** 5702, low-speed room temperature (Eppendorf, catalog 5702000010)
- **Centrifuge 5427 R, high-speed refrigerated (Eppenndorf, catalog 5429000133)**
- **Plasma prostaglandin E1** (Sigma, catalog P7527)
- **1.5ml microcentrifuge tubes** (Eppendorf, 022363204)
- **Hank's Balanced Salt Solution** 1X (HBSS) (Gibco, catalog 14175103)
- **Histopaque 1077** (Sigma, catalog 10771)
- **Histopaque 1119** (Sigma, catalog 11191)
- **Countess cell counting chamber slides** (ThermoFisher Scientific, catalog C12208)
- **Fetal Bovine Serum** (FBS) (Gibco, catalog 10437028)
- **DMSO Hybri-Max** (Sigma, catalog D2650)
- **Externally threaded cryogenic vials** (Corning, catalog 430659)

Procedure:

For a portion of the study (Mi045-Mi110), 4 citrate tubes are centrifuged at 1000xg for 5 min (at room temperature) without break. From each tube, 990 □l of plasma supernatant is collected in a 1.5 ml microcentrifuge tube (Eppendorf) and 10 □l of 1mM Plasma prostaglandin E1 (PGE1, Sigma, #P7527) is added for a final concentration of 10 □M. The tubes are centrifuged at 5000xg for 10 min at room temperature. From all 4 tubes, the supernatant is pooled and distributed into 4 fresh 1.5ml microcentrifuge tubes (Eppendorf). The plasma is snap-frozen in 100% ethanol with dry ice at approximately -78ºC and stored at -80 ºC

After plasma collection, the 4 citrate tubes containing buffy coat and red blood cells are carefully inverted 2-3 times and the blood is collected in a 50ml conical tube containing 30ml HBSS (Gibco, #14175103). A new 50ml tube is filled with 10ml Histopaque 1077 (Sigma, #107711) and the blood/HBSS is carefully layered onto the Histopaque using a 10 ml serological pipette. The layered blood/Histopaque mix is centrifuged at 400xg for 30 min without a break at room temperature. After centrifugation, the cells were collected from the layer on top of the Histopaque , transferred to a fresh 15 ml conical tube filled with 10ml HBSS, and centrifuged at 500xg for 10 min (with breaks on) at room temperature. The supernatant is removed by pouring, and the pellet is resuspended in the remaining liquid by tapping the tube against a closed fist. Next, 15 ml fresh HBSS are added to wash out platelets, followed by centrifugation at 200xg for 10 min at room temperature. This washing step is repeated one more time, and the pellet is finally resuspended in 1 ml HBSS. Cells are counted with Trypan Blue using a Countess II cell counter (Invitrogen) as described above. 2x1M, 1x2M and leftover (live) cells, as well as any leftover cells from the ACD-A PBMC collection (split by two if >1.5M cells), are collected in 1.5 ml microcentrifuge tubes and centrifuged at 300xg for 5 min at room temperature. After removing the supernatant, the cell pellet is re-suspended in 500 □l FBS (Gibco #10437028) and transferred to externally threaded cryogenic vials (Corning, #430659). As cryopreservation media, 500 □l FBS containing 20% DMSO Hybri-Max (Sigma, # D2650) is added dropwise to the cryogenic vial, and the cell suspension is carefully mixed (resulting in 10% DMSO in FBS). The cells are placed in a Mr. Frosty Freezing Container (Nalgene, #5100-000) filled with isopropanol (Sigma, #I9516) and

placed at -80 ºC for 2 days. Afterwards, cells are stored long-term in the vapor phase of liquid nitrogen. The ACD-A PBMC cryovials are labelled Leuk-PBMC-F.2-1 and Leuk-PBMC-F.2-2, and the citrate PBMC cryovials are labelled Leuk-PBMC-F.3-1 (1M), Leuk-PBMC-F.3-2 (1M), Leuk-PBMC-F.3-3 (2M), and Leuk-PBMC-F.3-4 (leftover cells). An overview of the PBMC fating sample collection is outlined below:

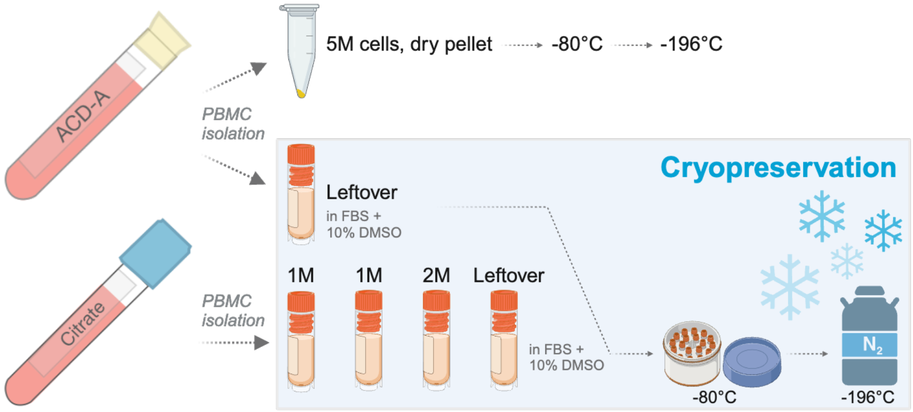

4.3.4 Purifying Leukocyte Subtypes

Materials:

- ☐ **50ml conical tubes** (Eppendorf, catalog 0030122178)
- ☐ **Hank's Balanced Salt Solution** 1X (HBSS) (Gibco, catalog 14175103)
- ☐ **Histopaque 1077** (Sigma, catalog 10771)
- ☐ **Histopaque 1119** (Sigma, catalog 11191)
- ☐ **Centrifuge** 5702, low-speed room temperature (Eppendorf, catalog 5702000010, Enfield, CT)
- ☐ **HBSS/BSA** (0.5% BSA in HBSS) (Sigma, #A3733)
- ☐ **1.5ml microcentrifuge tubes** (Eppendorf, 022363204)
- ☐ **CD14 antibody** (Miltenyi Biotec, #130050201)
- ☐ **MACs separator columns** (Miltenyi Biotech, #130042401)
- ☐ **CD15 antibody** (Miltenyi Biotec, #130046601)
- ☐ **Centrifuge 5427 R,** high-speed refrigerated (Eppenndorf, catalog 5429000133)

Procedure:

Two Ficoll double gradients are prepared by pipetting 10 ml of Histopaque 1077 to the bottom of two 50 ml conical tubes, then carefully dispensing 10 ml of Histopaque 1119 underneath the 1077 layer. After spinning the ACD-A tubes and aspirating the plasma (see *Platelet Isolation*), 3 ml of buffy coat is aspirated from each of the four remaining ACD-A tubes (5 drawn, 1 used for PBMCs) and pooled in a 50 ml conical tube. The tube is filled to 50 ml with HBSS and inverted ten times to ensure adequate mixing. 25ml of the leukocyte cell suspension is added to each of the two 50 ml Histopaque double gradient tubes (10 ml each of Histopaque 1077 and 1119). As described above, the cell suspension is carefully layered on the Histopaque using the blood wet pipette tip

to draw an upside down “Y” shape on the wall of the tube, then dispensing from the top of the “Y.” To properly balance the tubes in the centrifuge, each 50 ml tube should have equal amount of blood. The two tubes are carefully moved to the centrifuge and spun at 700xg for 30 minutes at room temperature with the centrifuge set **“break off.”** After centrifugation, 3 distinct layers are observed. The mononuclear cell (MCN) layer is a white layer between plasma and Histopaque 1077. The polymorphonuclear cell (PMN) layer is a red band (of varying definition; see Figure below) between Histopaque 1119 and 1077. Red blood cell (RBC) layer is located at the bottom of the tube.

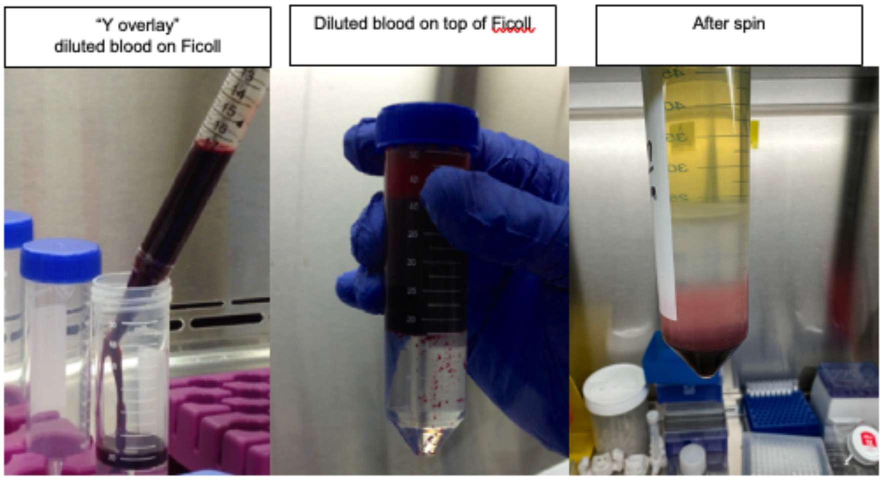

3/4 of the top diluted plasma layer is removed using a 5ml pipette. The MCN layer is collected from both 50 ml tubes with a wide bore 1 ml pipette, and pooled in a new 50 ml tube pre-filled with 5 ml HBSS. Similarly, the PMN band is collected from both 50 ml tubes and pooled in a new 50 ml tube pre-filled with 5 ml HBSS. HBSS is added to the new, pooled MCN and PMN tubes up to 50 ml followed by a centrifugation at 700xg for 10 minutes at room temperature with the centrifuge “break off.” After centrifugation, the supernatant is discarded via pouring for the MCN tube (attached pellet), and via pipette for the PMN tube (loose pellet). The cell pellets are resuspended by gently tapping the tube against a closed fist. Following this, 1 ml of HBSS/BSA (0.5% BSA in HBSS) (Sigma, #A3733) is added to each tube, cells are collected and the cell suspension is mixed, and dispensed in two separate 1.5 ml tubes labelled M14 for MCN and P15 for PMN. These 1.5 ml tubes are centrifuged at 700xg for 40 seconds at room temperature to pellet cells. The supernatant is discarded from each tube using a 1ml pipette, the pellets are resuspended by flicking with the finger, and 240 □l of HBSS/BSA is added to dissolve cell aggregates and form a uniform suspension of cells.

The monocyte, neutrophil and lymphocyte cell populations are isolated using magnetic bead labeled antibodies and MACs separator columns (Miltenyi Biotech, #130042401). The previously resuspended 1.5 ml tubes (M14 and P15) are mixed to ensure uniform cell suspension. For positive selection of monocytes, 66.4 □l of magnetic bead labeled CD14 antibody (Miltenyi Biotec, #130050201) are added to the M14 tube. For positive selection of neutrophils, 66.4 □l of magnetic bead labeled CD15 antibody (Miltenyi Biotec, #130046601) are added to the P15 tube. Tubes are inverted by hand to ensure mixing and the tubes are placed on wet ice to incubate for 15 minutes. Two MACs separator LS columns are labeled and attached to the magnetic station and two 15 ml tubes are placed below the columns to collect flow through for each cell type. Each column is equilibrated with 3 ml HBSS/BSA. After 15 minutes of antibody incubation,1 ml of HBSS/BSA is added to each 1.5 ml tube and mixed to properly wash the cell-antibody suspension. To pellet the cells, the tubes are centrifuged at 700xg for 40 seconds at room temperature. The supernatant is

discarded using a 1 ml pipette and the tubes are flicked with a finger to resuspend the pellet. 1 ml of HBSS/BSA is added to each tube and properly mixed to form a uniform cell suspension. Once suspended, the MCN (M14) and PMN (P15) cell suspensions is immediately applied to their respectively marked LS columns (MCN+ and PMN+). The sample is dispensed at the bottom of the column, close to and directly above – but without touching – the filter. Each column is washed three times with 3 ml of HBSS/BSA (total 9ml/column) and the flow through is collected for each cell suspension in their respective tubes. After completing 3 washes, the MCN+ and PMN+ columns are removed from the magnetic station while keeping them over their respective 15 ml tubes to collect any drops that gather during movement. The PMN flow through is discarded; the MCN flow through is kept for lymphocyte isolation (see below). To collect the cells attached to the columns, the columns are placed over new 15 ml tubes, 5 ml of HBSS is added to each column and the supplied piston is used to plunge it immediately. These positively selected monocytes and neutrophils cells are kept at room temperature till the lymphocyte isolation process is completed.

4.3.5 Lymphocyte Isolation

Materials:

- **HBSS/BSA** (0.5% BSA in HBSS) (Sigma, #A3733, Gibco, catalog 14175103)
- **1.5ml microcentrifuge tubes** (Eppendorf, 022363204)
- **CD61 antibody** (Miltenyi Biotec, catalog 130051101)
- **CD235 antibody** (Miltenyi Biotec, catalog 130050501)
- **Seahorse XF Assay Medium** (Agilent, catalog 102365-100)
- **Glucose** (Gibco, catalog 15023021)
- **Sodium pyruvate** (Gibco, catalog 11360070)
- **L-glutamine** (Gibco, catalog 25030081)
- **Uridine** (Sigma, catalog U6381-5G)
- **Palmitate** (Sigma, catalog P9767-5G)
- **BSA without fatty acids** (Sigma, catalog A3733-50G)
- **BCA assay kit** (Bioworld #20831001-1)
- **CelLytic M reagent** (Sigma, C2978-50ML)
- **Countess II FL Automated Cell Counter** (ThermoFisher, AMQAF1000)
- **Centrifuge 5427 R, high-speed refrigerated** (Eppenndorf, catalog 5429000133)

Procedure:

The MCN flow through tube is centrifuged at 300xg for 10 minutes at room temperature to pellet the cells. The supernatant is discarded, the cell pellet is resuspended in 1 ml of HBSS/BSA (0.5% BSA in HBSS), and the suspension is collected in a 1.5 ml tube. To pellet the cells, the tube is centrifuged at 700xg for 40 seconds at room temperature. The supernatant is discarded with a pipette, the tube is flicked to break up the cell pellet and the cells are resuspended in 240 □l of HBSS/BSA. Negative selection of lymphocytes is performed using magnetic beads labeled CD61(Miltenyi Biotec, #130051101) and CD235 (Miltenyi Biotec, #130050501) antibodies. 66.4 □l of both CD61 and CD235 antibody are added to the cell suspension. The suspension is mixed by inverting the tube, and incubated on wet ice for 15 minutes. Attach another MACs separator LS columns to the magnetic station, place one 15 ml tube below the column to collect flow through, and equilibrate the column with 3 ml HBSS/BSA. After the 15 minute antibody incubation, add 1 ml of HBSS/BSA to the 1.5 ml tube and mix to wash the cell-antibody suspension. Centrifuge the

tube at 700xg for 40 seconds at room temperature to pellet cells. Use a 1 ml pipette to discard the supernatant and flick the tube with a finger to resuspend the pellet. Add 1 ml of HBSS/BSA to the tube and mix properly to form a uniform cell suspension. Apply this cell suspension to the LS column at the bottom of the column, close to and directly above the filter. Wash the column three times with 3 ml of HBSS/BSA (total 9ml). After completing 3 washes, the negatively collected lymphocytes are in the 15 ml tube flow through; remove and discard (do not plunge) the LYM+ column.

Centrifuge all three 15 ml tubes – MCN for monocytes, PMN for neutrophils, and LYM for lymphocytes – at 700xg for 10 minutes at room temperature to pellet cells. After the spin, discard the supernatant and resuspend the cell pellet. Monocytes, neutrophils, and lymphocytes are resuspended in XF Media containing no pH buffers and supplemented with 5.5 mM glucose (Gibco, #15023021), 1 mM sodium pyruvate (Gibco, #11360070), 1 mM L-glutamine (Gibco, #25030081), 50 ug/ml uridine (Sigma, #U6381-5G), and 10 mM palmitate (Sigma, #P9767-5G) conjugated to 1.7 mM BSA without fatty acids (Sigma, #A3733-50G).

4.3.6 Cell counting and platelet quantification

Monocytes, neutrophils, and lymphocytes are counted using an automated cell counter (Countess II, Invitrogen). Platelets from Mi001 to Mi027 were quantified using a turbidity assay: 1 ml of platelets are transferred to a 1cm path length cuvette. A separate cuvette is filled with 1 ml of PBS-PGI2 buffer with no cells serving as control. The absorbance of the blank and platelet cuvette is measured at 750 nm 3 times. Between the measurements, the platelet suspension is carefully mixed by pipetting up and down. Platelet count is estimated using the formula described in (Walkowiak et al., 1997).

$$\frac{number\ of\ platelets}{ml} * 10^8 = 6.23 \Big/ \left(2.016 - 1.33 * 750 * \frac{\varepsilon}{750}\right) - 3.09 \quad * dil.factor$$

The ε (absorbance) value for control well should be around 0.5842666 (OD) at 750 nm. This value is subtracted to the sample & value for adequate count.

From Mi028 on, platelets were quantified based on whole protein content using a BCA assay kit (Bioworld #20831001-1) in a flat bottom 96 well plate (Eppendorf): 20 □l of platelets are lysed in 40 □l CelLytic M reagent (Sigma, C2978-50ML), and pipetted up and down several times for homogeneous resuspension, followed by an incubation for 10 minutes at RT. Reagent A and B are mixed with a dilution of 1:50[1]. Protein standards are prepared in advance using bovine serum albumin (BSA). 1500 mg BSA (Sigma, #A3733) is dissolved in distilled water to 50 mg/ml w/v, serving as a stock. Five serial dilutions and a blank are prepared in distilled water: 1 mg/ml, 500 □g/ml, 250 □g/ml, 125 □g/ml, and 62.5 □g/ml. Standards are aliquoted in PCR tubes strips (60 □l per tube) and stored at -30□C. 60 □l of each BSA standard and of the platelet lysate is transferred to a 96 well flat bottom plate (Eppendorf). 200 □l of reagent A + B mix is added to platelets and protein standard wells, mixed by pipetting and incubated at 37°C in a non-CO2 incubator for 30min. End-point absorbance at 562 nm is measured in a plate reader (Spectramax) without plate cover. Platelet absorbance is converted to □g protein using the BSA protein standards.

[1] This is technically wrong, as the BSA assay is one part A, 50 parts B. The mistake was just continued to keep it comparable. Shouldn't change the result too much, it'll probably change the overall protein quantification.

4.3.7 Seahorse Bioenergetics Measurements

Materials:

- **XFe96 Seahorse extracellular flux analyzer** (Agilent Technologies
- **XFe96 well plate** (Agilent Technologies, #102416-100)
- **30 □l poly-d-lysine** (PDL) hydrobromide (Sigma, #P6407)
- **Seahorse cartridge** (Agilent Technologies #102416-100)
- **XF Calibrant solution** (Agilent Technologies, #100840-000)
- **5.5 mM glucose** (Gibco, #15023021)
- **Sodium pyruvate** (Gibco,#11360070)
- **Glutamine** (Gibco, #25030081)
- **Uridine** (Sigma, #U6381)
- **Palmitate** (Sigma, #P9767)
- **BSA without fatty acids** (Sigma, #A3733)
- **ATP synthase inhibitor oligomycin** (Sigma, #75351, final concentration: 1 mM)
- **Protonophoric uncoupler FCCP** (Sigma, #C2920, final concentration: 2 mM)
- **Rotenone** (Sigma, #R8875, final concentration: 1 mM)
- **Antimycin A** (Sigma #A8674, final concentration: 1 mM)
- **Pyruvate carrier inhibitor UK-5099** (Sigma-Aldrich # PZ0160, final concentration: 5 mM)
- **Mammalian protein extraction reagent** (M-PER™, Thermo Fisher #78503
- **Bicinchoninic Acid method** (BCA, Bioworld #20831001-1)
- **Nuclear fluorescent stain** (Thermofisher #62249)
- **Cytation1 Cell Imager** (BioTek, now Agilent)

Procedure:

Oxygen consumption rate (OCR) and extracellular acidification rate (pH change) are measured over a confluent cell monolayer of isolated leukocytes and platelets using the XFe96 Seahorse extracellular flux analyzer (Agilent Technologies). 6 Hours prior to the Seahorse run, an XFe96 well plate (Agilent Technologies, #102416-100) is coated with 30 □l poly-d-lysine (PDL) hydrobromide (Sigma, #P6407) per well for 1 hour at RT. Afterwards, excess PDL is removed, and non-bound PDL is washed off with water (2 x 100 □l). The plate is kept slightly open in a sterile environment to allow drying. A seahorse cartridge (Agilent Technologies #102416-100) is hydrated in XF Calibrant solution (Agilent Technologies, #100840-000) for 6 hours at 37°C.

After leukocyte separation, monocytes, neutrophils, and lymphocytes are resuspended in XF Media containing no pH buffers and supplemented with 5.5 mM glucose (Gibco, #15023021), 1 mM sodium pyruvate (Gibco, #11360070), 1 mM L-glutamine (Gibco, #25030081), 50 ug/ml uridine (Sigma, #U6381), and 10 mM palmitate (Sigma, #P9767) conjugated to 1.7 mM BSA without fatty acids (Sigma, #A3733). A total of 0.75 µg platelet protein (~25M platelets) and 0.25M monocytes, neutrophils, and lymphocytes respectively are seeded per well (n=6 technical replicates) in the PDL-coated XF 96 well plate (see figure below). From the same cell suspension in XF media, two aliquots of 25M platelets and 0.25M monocytes, neutrophils and lymphocytes each are collected in a PCR tube strip for mitochondrial DNA copy number measurements, and stored long-term at -80°C. To control for plate-to-plate variability and as an injection control of the seahorse run, a HEK293 cell line is maintained and run on each seahorse plate at 40,000 cells per well. Once seeded, the plate is pulse spun at up to 400xg, the plate is reversed and spun again to allow uniform cell adhesion. Finally, it is incubated in a non-CO_2 incubator for 1 hour to equilibrate temperature and atmospheric gases. If not otherwise specified, the same plate setup is maintained (see figure below).

The Seahorse instrument is programmed to assess various respiratory states using the manufacturer's protocol of sequential substrate addition and measurements (4x, 3 measurements over 18 minutes). Basal respiration, ATP turnover, proton leak, coupling efficiency, maximum respiration rate, respiratory control ratio, spare respiratory capacity and non-mitochondrial respiration are all determined by the sequential additions of the ATP synthase inhibitor oligomycin (Sigma, #75351, final concentration: 1 mM), the protonophoric uncoupler FCCP (Sigma, #C2920, final concentration: 2 mM), and the electron transport chain Complex I and III inhibitors, rotenone (Sigma, #R8875, final concentration: 1 mM) and antimycin A (Sigma #A8674, final concentration: 1 mM). Additionally, prior to these injections, half of the plate is injected with a pyruvate carrier inhibitor UK-5099 (final concentration: 5 mM, Sigma-Aldrich # PZ0160) and a baseline is measured to quantify metabolic flexibility between sugar and fatty acid oxidation (3 measurements over 18 minutes). The optimal concentration for the uncoupler FCCP yielding maximal uncoupled respiration is determined based on a titration performed on the leukocytes.

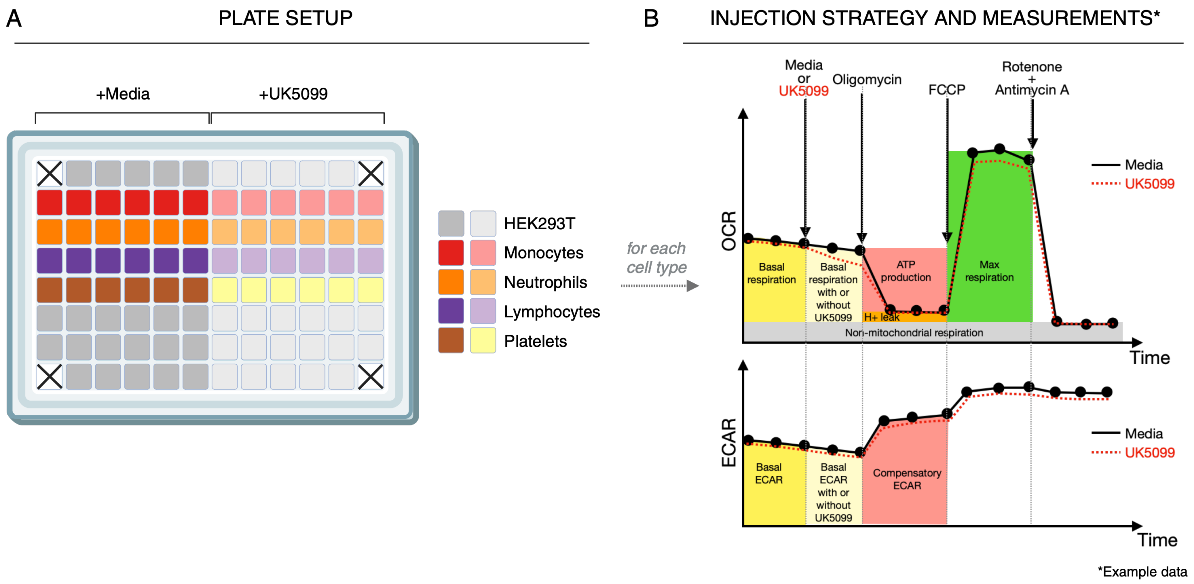

A XFe96 plate setup. Cells seeded in the left side of the plate (columns 1 – 6) receive supplemented XF media in the first injection (i.e. following a normal MitoStressTest) and cells in the right side of the plate (columns 7-12) receive the pyruvate carrier inhibitor UK5099 in the first injection. **B** Injection strategy and example OCR and ECAR traces.

After each run, normalization is performed with either a protein concentration (Mi001-014) or using image-based cell counts (Mi016-110). For protein normalization, cells from each well are lysed using a mammalian protein extraction reagent (M-PER™, Thermo Fisher #78503). Relative protein concentration is determined spectrophotometrically by the Bicinchoninic Acid method (BCA, Bioworld #20831001-1), and OCR measurements are normalized to the protein content on a per-well basis. For the cell count-based normalization, the final injection of rotenone+antimycin A includes 2.5 μM Hoechst nuclear fluorescent stain (Thermofisher #62249) to allow for automatic cell counting. After each run, cell nuclei are counted automatically using the Cytation1 Cell Imager (BioTek, now Agilent) and raw bioenergetic measurements are normalized to relative cell counts on a per-well basis. For platelets (that are lacking nuclei), brightfield images are taken in the same run.

ATP production rates from OxPhos (ATP_{OX}) and glycolysis (ATP_{Glyc}), as well as total cellular ATP production and consumption (ATP_{TOTAL}), are estimated using the method described by Mookerjee and Desousa (Desousa et al., 2023; Mookerjee et al., 2017). Briefly, the method relies on the phosphate-to-oxygen (P/O) ratios of OxPhos and glycolysis, using oxygen consumption and proton production rates (PPR) as input variables. The same constants are used for all estimations, assuming glucose as the predominant carbon source. The buffer factor (i.e. amount of protons added to the assay medium to change the pH level by 1 pH unit in the seahorse instrument) was determined experimentally by titrating HCl to the assay medium according to the instructions provided by agilent (Agilent Seahorse XF Buffer Factor Protocol). The buffer factor of the seahorse medium used in this study (DMEM without HEPES) was 0.9 mM/pH.

4.3.8 Leukocyte Storage

Materials:

- **Sorvall ST 16R refrigerated centrifuge** (ThermoFisher Scientific, catalog 75004381)
- **RNAlater** (Sigma #R0901)

Procedure:

The remaining leukocytes are stored in two aliquots (where applicable). The first aliquot containing 1M cells (monocytes, Leuk-Mon-F.1), 3M cells (neutrophils, Leuk-Neu-F.1), 5M cells (lymphocytes, Leuk-Lym-F.1), and 5.84ug (~200M platelets, Leuk-Plt-F.1) is centrifuged at 300xg for 5min at 4°C. The supernatant is removed, and the pellet is resuspended in 500 µl RNAlater (Sigma #R0901). The sample is stored in a microcentrifuge tube short-term at -80°C and long-term in the vapor phase of liquid nitrogen. The remaining cells (Leuk-Mon-F.2, Leuk-Neu-F.2, Leuk-Lym-F.2, Leuk-Plt-F.2) are centrifuged at 300xg for 5min at RT, the supernatant is removed, and the pellet is washed in PBS, followed by a centrifugation step at 2000xg for 2 min at 4°C. The PBS is aspirated, and the dry pellet is stored in a microcentrifuge tube short-term at -80°C and long-term in the vapor phase of liquid nitrogen.

4.3.9 HEK Passaging

Materials:

- **Dulbecco's Phosphate Buffered Saline** 1X (DPBS) (Gibco, catalog 14190-250)
- **HyClone Trypsin** (Cytiva, #SH30042.02)
- **DMEM** (Gibco #10569044)
- **10% FBS** (Gibco, #10437028)
- **1% MEM-non-essential amino acid solution** (Sigma, #M7145)
- **Countess II Automated Cell Counter**, Introvergen (Thermo Fisher, AMQAX1000)

Procedure:

HEK293 cells are passaged once per week on the day of the seahorse experiment. Cells cultured in a T25 or T75 flask are washed with 1x DPBS (Gibco, #14190250), and incubated in HyClone Trypsin (Cytiva, #SH30042.02) for 3 min at 37 ºC. Trypsin is neutralized with HEK media, consisting of high glucose DMEM (Gibco #10569044), 10% FBS (Gibco, #10437028, same LOT across the entire study) and 1% MEM-non-essential amino acid solution (Sigma, # M7145). Cells are collected in a 15 ml conical tube, and centrifuged at 300xg for 5 min. Next, the supernatant is

removed, and cells are resuspended in HEK media. Cells are counted using an automated cell counter (Countess II, Invitrogen), and 83k (T25) or 250k (T75) cells are seeded and placed in a CO_2 incubator at 37ºC.

4.4 Blood Processing: Stress Reactivity

4.4.1 Serum and Plasma Processing

Materials:

- **Sorvall ST 16R refrigerated centrifuge** (ThermoFisher Scientific, catalog 75004381)
- **15ml conical tubes (**Eppendorf, catalog 0030122151)
- **2ml crtogenic storage tubes**

Procedure:

Serum:

Blood is extracted by intravenous catheter in the antecubital vein and collected in 8 X 6.0ml Serum Clot activator tubes (BD, REF367815) throughout the afternoon. Afternoon serum samples are immediately inverted 10-12 times. Blood rested for 30 minutes at room temperature. After 30min, samples are centrifuged at 2000xg (3.6rpm) for 3.5 minutes at room temperature. Samples are immediately placed on ice (4°C) in a Styrofoam box. The first spin is recorded in the centrifugation log (below).

Plasma:

Blood was extracted by intravenous catheter in the antecubital vein and collected in 8 X 6.0ml (K2 EDTA, BD, REF367899) tubes throughout the afternoon. Whole blood tubes for afternoon stress plasma samples were immediately inverted 10-12 times and centrifuged at 2000xg (3.6rpm) for 3.5 minutes at room temperature after collection. Samples are immediately placed on ice (4°C) in a Styrofoam box. The first spin is recorded in the centrifugation log (below).

	Pink Plasma		Red Serum	
**-5**		Time drawn:<br>Time centrifuged:		Time drawn:<br>Time centrifuged:
**+5**		Time drawn:<br>Time centrifuged:		Time drawn:<br>Time centrifuged:
**+10**		Time drawn:<br>Time centrifuged:		Time drawn:<br>Time centrifuged:
**+20**		Time drawn:<br>Time centrifuged:		Time drawn:<br>Time centrifuged:
**+30**		Time drawn:<br>Time centrifuged:		Time drawn:<br>Time centrifuged:
**+60**		Time drawn:<br>Time centrifuged:		Time drawn:<br>Time centrifuged:
**+90**		Time drawn:<br>Time centrifuged:		Time drawn:<br>Time centrifuged:
**+120**		Time drawn:<br>Time centrifuged:		Time drawn:<br>Time centrifuged:

Serum and Plasma

After blood collection and first centrifugation(above), plasma and serum blood tubes are placed on ice(4°C) in a Styrofoam box for transport to the laboratory (5 minutes' walk). Stress reactivity time points include 8 tubes for each timepoint, starting at -5 minutes to 120 minutes. A total of 16 blood tubes are collected, 8 each for plasma and serum. At the laboratory, blood tubes are centrifuged at 2000g for 10 minutes at 4 degrees Celsius. After centrifugation, tubes are placed back on wet ice, and around 80 percent of plasma and serum is aspirated without disturbing the sediment into a new 15 ml conical tubes. Plasma tubes for each time point are retained for PBMC isolation and serum tubes are discarded. After collection into new 15 ml tubes, all tubes are centrifuged again at 2000xg for 10 minutes at 4 degrees Celsius. After centrifugation, tubes are placed back on wet ice, and around 90 percent of plasma and serum are aspirated without disturbing the sediment into a new 15 ml conical tubes. These samples are now considered clean of red blood cells and are aliquoted into 2ml cryogenic storage tubes. Each time point for plasma and serum is aliquoted thrice (e.g. Stress plasma 1.1, 1.2, 1,3 and Stress serum 1.1, 1.2, 1.3) in separate 2ml cryogenic storage tubes. Aliquots are immediately stored in a -80 freezer and storage time for each sample type is recorded.

4.4.2 PBMC Cryopreservation

Materials:

- **Histopaque 1077** (Sigma, catalog 10771)

- **Centrifuge** 5702, low-speed room temperature (Eppendorf, catalog 5702000010)
- **Hank's Balanced Salt Solution** 1X (HBSS) (Gibco, catalog 14175103)
- **Fetal Bovine Serum** (FBS) (Gibco, catalog 10437028)
- **Cryogenic vials** (Corning, cryogenic vials, CLS430289)
- **FBS + 20%DMSO Hybri-Max** (Sigma, D2650)
- **Mr. Frosty freezing container** (Thermo Scientific, 51000001)
- **Isopropanol** (Sigma)

Procedure:

After plasma collection, the leftover buffy coat and red blood cells are carefully inverted 2-3 times and for each stress reactivity sample, the blood is collected in a 15ml conical tube containing 8ml HBSS (Gibco). A new 15ml tube is filled with 4ml Histopaque 10771 and the diluted blood is carefully layered onto the Histopaque using a 10ml pipette. The layered blood/Histopaque mix is centrifuged at 400xg for 30 min without a break at RT. After centrifugation, the cells are collected from the layer on top of the Histopaque in a 15 ml conical tube filled with 10ml HBSS, and centrifuged at 500xg for 10 min (with breaks on). The supernatant is removed by pouring, and 15ml fresh HBSS is added to wash out platelets, followed by centrifugation at 200xg for 10 min. This washing step is repeated one more time, and the pellet is resuspended in 1ml FBS (Gibco). 10 µl of stress reactivity tube 1, 4, and 8 are collected each and pooled for cell counting (as described above). For each stress reactivity timepoint, the cell suspension is divided into two cryogenic vials (Corning) and 500ul of FBS + 20%DMSO Hybri-Max (Sigma) are added dropwise to the vial, and the cell suspension is carefully mixed. The same procedure is applied to the leftover whole blood PBMC pellet (S.2-1 and S.2-2, see below 4.4.3). The cells are placed in a Mr. Frosty freezing container filled with isopropanol (Sigma) and placed at -80 ºC for 2 days. Afterwards, cells are stored long-term in the vapor phase of liquid nitrogen. The stress reactivity PBMCs cryovials are labelled SRx-1 and SRx-2 (with x being the stress reactivity time point between -5 (1) and +120min (8), and 1 or 2 indicating the replicate).

4.4.3 Whole Blood Processing:

Materials:

- **Centrifuge** 5702, low-speed room temperature (Eppendorf, catalog 5702000010)

Procedure:

Blood is extracted by intravenous catheter in the antecubital vein and collected in 5 X 8.5ml (BD-364606) tubes at 4pm from the left arm. Plasma and serum blood tubes are placed over wet ice contained in a Styrofoam box for transport to the laboratory (5-minute walk). At the laboratory, plasma and serum tubes are centrifuged at 2000xg for 10 minutes at 4 degree Celsius. ACD-A blood tube used for PBMC isolation is transported to laboratory at room temperature along with remaining blood tubes. At the laboratory, ACD-A blood tube is centrifuged 500xg for 15 minutes at room temperature with centrifuge brake off.

4.4.4 Purifying PBMCs

Please refer to section 4.3.2 for materials and PBMC isolation procedure.

Procedure:

Once PBMCs are purified and cell count has been performed, cell aliquots of 5 million cells are calculated based on the average live cell count and stored as stress reactivity PBMC aliquot labeled S.1. The remaining cell suspension volume is measured, cell count calculated based on average count and cryopreserved as stress reactivity PBMC aliquot labeled S.2-1 and S.2-2. Aliquots are centrifuged at 2000xg for 2 minutes in a pre-chilled micro-centrifuge at 4-degree Celsius. The supernatant is aspirated as much as possible without disrupting the pellet and stored in a -80 freezer.

4.5 Saliva Processing

Materials:

- **Sorvall ST 16R refrigerated centrifuge** (ThermoFisher Scientific, catalog 75004381)
- **Cryogenic vials** (Corning, cryogenic vials, CLS430289)

Procedure:

Saliva was collected at 26 timepoints: 1) day 1 morning fasting sample, 2) 8 afternoon samples collected during the stress reactivity protocol, 3) 1 sample after a cold pressor test, 4) day 2 morning fasting sample, 5) 3 afternoon stress samples before and during the MRI, 6) 12 at home samples collected across 3 days: (i) immediately upon awakening, (ii) 30 min after waking up, (iii) 45 min after waking up, and (iv) at bedtime.

4.5.1 Fasting Saliva:

Saliva time point Sal 1 (Day 1) was collected ~10am and immediately placed in a styrofoam box on ice (4°C) for transport to the lab. At the lab, salivette tubes are centrifuged at 1000g for 5 minutes at 4 degrees Celsius. Supernatant is carefully aliquoted into 2ml cryogenic storage tubes with two aliquots for each timepoint (e.g., Fasting Sal 1.1, 1.2) and stored immediately in -80 freezer. Time of sample storage is recorded.

On Day 2, Sal 11 is collected at ~9:15am and immediately placed in a Styrofoam box on ice (4°C). Saliva timepoints Sal 11 to Sal 14 (Day 2) are transported to the lab late afternoon and are stored on wet ice, inside a 4°C refrigerator, and processed the morning of the following day before 11am following the same procedures as Sal 1(above).

4.5.2 Afternoon Saliva:

On Day 1, saliva time points Sal 2 to Sal 10 (Day 1) are collected after lunch and during stress reactivity and immediately placed in a Styrofoam box on ice (4°C). After all samples have been collected (~4:15pm), samples are transported to the lab for processing following the same procedures as fasting samples.

On Day 2, saliva time points Sal 12 to Sal 14 (Day 2) are collected after and during brain imaging procedures. After collection, all samples are immediately placed in a styrofoam box on ice (4°C). Samples are transported to the lab at ~5:15pm and are stored on wet ice in a 4°C refrigerator. Samples 11-14 are processed the morning of the following day (Wednesday) before 11am.

4.5.3 Diurnal Saliva processing

Materials:

- **Centrifuge** 5702, low-speed room temperature (Eppendorf, catalog 5702000010)
- **Cryogenic vials** (Corning, cryogenic vials, CLS430289)

Procedure:

Within 2 weeks of collection, participants either transported (10%) or shipped Diurnal saliva (dsal) samples (90%) to the laboratory in a non-temperature controlled, pre-stamped USPS priority shipping. Salivette tubes are stored at 4°C immediately after reception and processed either the day of reception or the following morning. Tubes are centrifuged at 1000xg for 5 minutes at room temperature to extract saliva from the salivette. The supernatant, without touching the sedimented cells, was carefully aliquoted into 2ml cryogenic storage tubes as two aliquots for all 12 timepoint (e.g., Fasting Dsal 1.1, 1.2) and stored immediately at -80°C.

4.6 Urine Processing

Materials:

- **50ml conical tubes** (Eppendorf, catalog 0030122178)
- **Centrifuge** 5702, low-speed room temperature (Eppendorf, catalog 5702000010)

Procedure:

Urine is collected overnight at the hotel, from 8am to 8pm, in a container containing 30% acetic acid (Avantor, Cat#38533G). Whole urine is then transferred to 50ml conical tubes and centrifuged at 1,000g for 10 minutes to pellet cellular components. Cell-free urine aliquots (Ur.1, Ur.2) (2 x 1ml) are collected and stored.

Urinary epithelial cells are pooled from all tubes, resuspended with 200ul of PBS, separated in two equal aliquots (UEp.1, UEp.2), and stored at -80°C.

4.7 Fecal Sample Processing

Procedure:

Fecal sample is delivered along with Diurnal saliva samples by the USPS mailing service. Upon receiving, the specimen tube is stored at -80°C.

5 Biospecimen Measurements

5.1 Cell-free DNA

Materials:

- **96-well semi-skirted PCR plates** (BrandTech #781375)
- **Tris-HCl** (Sigma #T3253)
- **6% Tween-20** (Sigma #P1379)
- **Proteinase K** (Thermofisher #AM2548)
- **Domed tube caps** (BrandTech #781340)
- **T100 thermocycler** (Bio-Rad #1861096)
- **2xTaqMan Universal Mastermix Fast** (Life Technologies #4444965)
- **B2M Probes-FAM** (idtDNA.com).
- **qPCR reaction plate** (ThermoFisher # 4309849)
- **MicroAmp optical adhesive film** (ThermoFisher #4311971).
- **PCR system** (ThermoFisher #4485701).

Procedure:

The MitoQuicLy method as previously described is used to quantify cell-free mitochondrial DNA (cf-mtDNA) and cell-free nuclear DNA (cf-nDNA) (Michelson et al., 2023). Briefly, frozen plasma, serum, and saliva are thawed on ice, and a 50 µL aliquot is transferred from each sample into 96-well semi-skirted PCR plates (BrandTech #781375). We isolate DNA from each sample in duplicate by combining 10 µL of sample with 190 µL of MitoQuicLy lysis buffer in new 96-well semi-skirted PCR plates. The lysis buffer is composed of 114 mM Tris-HCl (Sigma #T3253), 6% Tween-20 (Sigma #P1379), and 200 µg/mL proteinase K (Thermofisher #AM2548) which are dissolved in nuclease-free water (Thermofisher #AM9939). Lysis plates are firmly sealed using domed tube caps (BrandTech #781340) and placed in a T100 thermocycler (Bio-Rad #1861096). Samples are incubated in the thermocycler at 55ºC for 16 hours, followed by a proteinase K inactivation step at 95ºC for 10 minutes, and then held at 4ºC until quantitative polymerase chain reaction (qPCR).

We performed qPCR using a duplex TaqMan assay targeting amplicons in the MT-ND1 (mtDNA) and B2M (nDNA) genes. The TaqMan assay mastermix is composed of 2xTaqMan Universal Mastermix Fast (Life Technologies #4444965) with 300 nM MT-ND1 Primers F+R, 300 nM B2M Primers F+R, 100 nM MT-ND1 Probes-VIC, and 100 nM B2M Probes-FAM (idtDNA.com). The MT-ND1 gene (ENSG00000198888.2) is targeted using a 69bp amplicon from position 3,485-3,553. The forward primer is GAGCGATGGTGAGAGCTAAGGT and the reverse primer is CCCTAAAACCCGCCACATCT. The ND1 probe is HEX-CCATCACCCTCTACATCACCGCCC-3IABkFQ. The B2M gene (ENSG00000166710.22) in chromosome 15 was targeted using a 96bp amplicon from position 44,715,455-44,715,550. The B2M forward primer is TCTCTCTCCATTCTTCAGTAAGTCAACT and the reverse primer is CCAGCAGAGAATGGAAAGTCAA. The B2M probe is FAM-ATGTGTCTGGGTTTCATCCATCCGACA-3IABkFQ.

12 µL of TaqMan mastermix is combined with 8 µL lysed sample in a 384-well qPCR reaction plate (ThermoFisher # 4309849). All lysates are run in triplicates. The qPCR reation plates are sealed with MicroAmp optical adhesive film (ThermoFisher #4311971). We use the QuantStudio 7 Flex real-time PCR system (ThermoFisher #4485701). The cycling conditions are as follows: 50ºC for 2 minutes, 95ºC for 20 seconds, followed by 40 cycles of 95ºC for 1 second, and 60ºC

for 20 minutes (total runtime: 40 minutes). A ∆Rn (normalized reaction) threshold of 0.08 was used to obtain cycle threshold (Ct) values. We compute the average Ct and coefficient of variation (CV) for each set of qPCR triplicates and discarded outlier wells that contributed to CVs ≥2%. For each lysate, we compute the number of mtDNA copies by correlating cycle thresholds of a 8-point standard curve run on each plate with known copy numbers from this standard (for details, see (Michelson et al., 2023)). We obtained cf-mtDNA and cf-nDNA levels for each sample by computing the average values between both lysates.

5.2 Biogenic Amines

Procedure:

Norepinephrine, Epinephrine, Dopamine and Serotonin were measured in human serum and urine samples using Liquid Chromatography-Tandem Mass Spectrometry (UPLC-MSMS). The metabolites were extracted from the samples spiked with a deuterated internal standards by protein precipitation followed by derivatization with dansyl chloride. The supernatant was further subjected to liquid-liquid extraction using ethyl acetate, and the extracted metabolites were suspended in acetonitrile for LCMS analysis. Chromatographic separation was carried out on a Waters ACQUITY UPLC HHS C18 column (2.1× 100 mm, 1.8 µm) maintained at 40°C, by gradient elution with water and acetonitrile with 0.1% formic acid as mobile phases at a flow rate of 300 µL/min. LC-MS/MS analysis was performed using positive ESI with multiple reaction monitoring (MRM) mode (transitions: norepinephrine: 869.2>170.3; epinephrine: 883.3> 170.2; dopamine: 853.30>170.37; serotonin: 643.29>146.05) on a Waters Xevo TQS MS integrated with ACQUITY UPLC system (Waters, Milford, MA, USA). These assays were performed by the Biomarkers Core Laboratory at the Irving Institute for Clinical and Translational Research, home to Columbia University's Clinical and Translational Science Award.

5.3 Steroid Hormones

Materials:

- **API 5000 QTrap mass spectrometer** (AB Sciex #6669)

Procedure:

Steroid hormone levels from hair and saliva samples were evaluated using high performance liquid chromatography–tandem mass spectrometry (LC–MS/MS), as previously described (Gao et al., 2013; Gao et al., 2015). Briefly, two hair strands of 3 cm were carefully cut with fine scissors as close as possible to the scalp from a posterior vertex position of each participant and stored at room temperature until analysis. The samples were then washed with isopropanol and 10 mg whole, non-pulverized hair was used for steroid hormone extraction by methanol incubation. A column switching strategy for on-line solid phase extraction (SPE) was applied, followed by analyte detection on an AB Sciex API 5000 QTrap mass spectrometer. Cortisol, cortisone, corticosterone, testosterone, and progesterone were detected within a range of 0.09-90 pg/mg, and of dehydroepiandrosterone (DHEA) within a range of 0.9-900 pg/mg, with an intra- and inter-assay coefficients of variation between 3.7 and 9.1%.

On the other hand, saliva samples were collected using Salivette devices and stored at -20°C until analysis. After thawing, samples were centrifuged, and 100 µL of saliva was added to a tube with 50 µL of internal standard and 100 µL of methanol/water solution containing zinc sulfate. The mixture was then vortexed, centrifuged, and the supernatant was injected into the LC-MS/MS

system for analysis. Cortisol, cortisone, corticosterone, testosterone, and progesterone were detected within a range of 0.001-10 ng/mL and DHEA within a range of 0.01-20 ng/mL for, with an intra- and inter-assay coefficients of variance between 4.3% and 10.8%.

Out of the total 18480 potential values obtained from analyzing six steroid hormones in 2 hair samples and 26 saliva samples per participant across 110 participants, we successfully covered 70.41% of them. Of the remaining values, 20.61% fell below the detection range, with more than half (10.83%) attributed to Corticosterone. The other 8.98% of values were missed, with 5.33% due to lack of sample and 3.66% due to technical issues.

5.4 GDF-15

Materials:

- **96-well semi-skirted PCR plates** (BrandTech #781375)
- **Human GDF-15 Quantikine ELISA Kit** (R&D #DGD150, # SGD150)

Procedure:

Plasma and saliva GDF15 levels were quantified using a high-sensitivity ELISA kit (R&D, DGD150, SGD150) following the manufacturer's instructions. Different lot numbers were used and the average coefficient of variation (CV) between lot number was determined by reference samples for quality control. Plasma and serum samples were diluted with assay diluent provide in the kit (1:4 ratio), saliva samples were not diluted. Absorbance was gauged at 450nm, and concentrations were computed utilizing the Four Parameter Logistic Curve (4PL) model. Samples were run in duplicate plates when sufficient volume was available, and the concentration for each sample was computed from the average of the duplicates.

Standard curve (5 samples per plate) and plasma reference samples (2-3 samples per plate, same sample per batch) were run with each individual assay and the inter-assay CV was monitored. All standard curves and references were overlaid on top of each other to monitor failed runs. Samples were run in duplicate plates when possible and those with CV larger than 15% were rerun. When it was not possible to rerun (eg. no sample left), sample sets with a CV >15% between the duplicates were excluded for sensitivity analysis and did not change the results. For the MiSBIE study, samples were run in 3 batches over 2 years, multiple quality control measures were applied to monitor batch-to-batch and within batch variability. Values below the mean minimum detectable dose (2.0pg/ml) were considered as non-detectable (reported as NA) and excluded in the graphs or statistical analyses. For full sample size, table below. Data-preprocessing and quality control measures was done using R Software (version 4.2.2) and are available on Github at https://github.com/mitopsychobio/2024_GDF15_Dynamics_Huang

Name	Population	Age range	Number of Participants	Sample Type	Total Number of Samples	Actual number of sample	Sampling success rate	Reason for missing sample	Number of sample included in analyses	Assay success rate
MiSBIE	Healthy adults	18-60	70	Plasma	630	571	91%	Failed blood draw	571	100%
				Saliva	1820	1505	83%	Low saliva volumn	1476	98%
	Deletion		15	Plasma	135	102	76%	Failed blood draw	102	100%
				Saliva	390	306	78%	Low saliva volumn	302	99%
	3243A>G mutation		20	Plasma	180	160	89%	Failed blood draw	160	100%
				Saliva	520	459	88%	Low saliva volumn	453	99%
	MELAS		5	Plasma	45	28	62%	Failed blood draw	28	100%
				Saliva	130	95	73%	Low saliva volumn	95	100%

6 Study Specific Tasks

6.1 Psychophysiological Session Flow

Background:

The 202-minute psychophysiological protocol was designed to monitor participants' autonomic nervous system activity while resting and in response to four discrete stress tasks, as well as a mindfulness task.

Procedures:

Measurements occur in a quiet and temperature-controlled room. A trained technician places electrodes, transducer bands, and the Nexfin finger cuff. Participants begins the psychophysiological monitoring session in a seated position to minimize hydrostatic errors and limit spontaneous variations in BP or HR. After calibration procedures described above, a 360-second baseline period assessment occurs. A 1440-second non-monitoring period progressed before a 300-second "5-minute pre-stress" sampling period. The investigators then deploy the Trier social stress test consisting of 120 seconds of speech preparation, followed by 180 seconds of speech delivery under evaluation. Immediately following the TSST, two 300-second sampling period continues, delineating "5-minutes post-TSST," and "10-minutes post-TSST" periods. The investigators also sample identical periods at "20-minutes post-TSST," "30-minutes post-TSST," "60-minutes post-TSST," "90-minutes post-TSST," and "120-minutes post-TSST."

Participants then complete a 300-second deep-breathing task (DBT), wherein participants guide their breathing to a metronomic visualization of 5-second-long inspirations and 7-second-long expirations. After the DBT, the technician situates participants in a standing position for 300 seconds to evoke an orthostatic stress response. Breath volume is calibrated in the standing position using the method described above. Following the orthostatic stress, participants perform a 30-second Chair Stand task (Centers for Disease Control and Prevention, 2017), wherein participants rapidly alternate from a chair-seated to a standing position for 30 seconds, using only their lower body strength, ending in a seated position, with a 300-second recovery period to follow.

The investigators deploy the Cold Pressor Test, wherein participants submerge their right hand into a 10-liter container of water chilled to 3.1-3.4°C for as long as they can bear, up to 120 seconds, followed by a 300-second recovery period.

Following cold pressor test recovery, participants complete a ten-minute metabolic rate analysis. At the conclusion of the metabolic rate analysis, participants are de-instrumented.

For analysis, the investigators segregate the measurements described above into 300-second, 60-second, and 10-second intervals for each period, to the extent that period length, sampling rate, signal clarity, resolution, and presence of noise or artifacts permitted.

6.2 Speech Task

Materials:

- ☐ **Full Length Mirror**
- ☐ **Video Camera with tripod**
- ☐ **Clipboard**
- ☐ **White Coat**

Background:

To access the stress response to a psychosocial stress, the Trier Social Stress Test was utilized. This psychosocial stressor has been used as a reliable situation to induce and measure the stress response (Jezova et al., 2004).

Procedure:

Five minutes before the introduction of the speech task, participant's blood, saliva, temperature, and affect are collected to determine a baseline before the introduction of stress. To induce stress, the study coordinator enters the room with a clipboard and a video camera with a tripod attached. The coordinator silently sets up the video camera and makes it appear as though it is recording. A full-length mirror was then set up in front of participants so they could clearly see themselves during the duration of the task. The coordinator instructs participants to prepare a speech in reaction to an uncomfortable situation, being accused of stealing store merchandise by a security guard. After the instructions and situation are read, participants complete the Primary Appraisal Secondary Appraisal (PASA) threat questionnaire. Participants are given 2 minutes to prepare their speech and instructed to then deliver their speech to an evaluator for 3 minutes. The evaluator, wearing a white coat and carrying a clipboard, walks into the room silently and takes notes as participants complete their speech. After the 3-minute period, the camera and mirror are immediately removed from sight; blood, saliva, temperate and affect ratings are immediately taken. Further details regarding this task) can be found later in this document (study coordinator script, evaluator instructions, participant handout).

Outcome Variables:

- Primary Appraisal Secondary Appraisal (threat)
- Continuous physiological measurements (body temperature, blood pressure, respiratory rate, pulse rate, skin conductance)
- Biospecimens (blood, saliva)
- Affect (positive and negative)

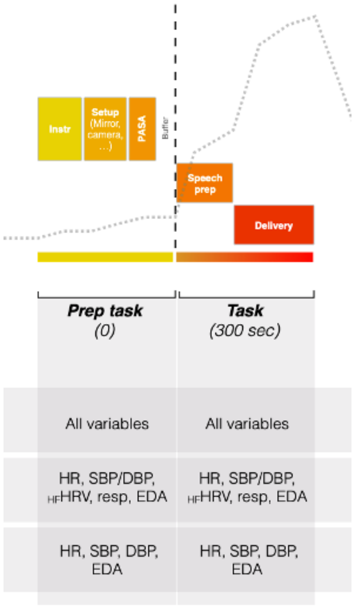

6.3 Deep Breathing Task

Materials:

- **Custom-designed paced breathing visual task on laptop**

Background:

Deep breathing has been associated with a shift towards parasympathetic nervous system dominance (Russo et al., 2017). A paced, deep breathing task enables assessment of automatic regulation through breathing.

Procedure:

Participants complete a 300-second deep-breathing task (DBT), wherein participants guide their breathing to a metronomic visualization of 5-second-long inspirations and 7-second-long expirations.

Outcome Measures:

- Continuous physiological measurements (body temperature, blood pressure, respiratory rate, pulse rate, skin conductance)

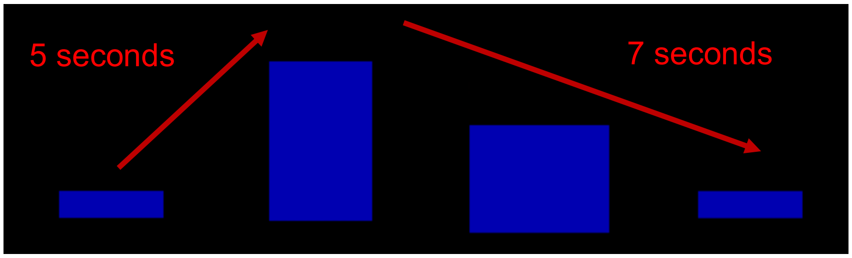

6.4 Stand Task

Materials:

- **Inflatable Spiro Bags** (Ambulatory Monitoring, Inc., 10.4028, Ardsley, NY, USA)

Background:

Orthostasis describes the physiological phenomenon of blood volume shift from the upper body to the lower body when standing from a sitting position. This is a normal response in the transition from sitting to standing but in some individuals this transition is accompanied by hypotension (dizziness, lightheadedness). Orthostasic hypotension can increase the likeliness of falls and decrease quality of life (Campos Munoz A, 2022).

Procedures:

Participants stand and remain upright for 300-seconds (i.e., 5 minutes) to evoke an orthostatic challenge. After 300 seconds, the breathing bands are calibrated in the standing position using a known volume of air with a spiro bag.

Outcome Measures:

- Continuous physiological measurements (body temperature, blood pressure, respiratory rate, pulse rate, skin conductance)

6.5 Sit-to-Stand Task

Materials:

- **Digital Timer.** Traceable Four-Channel Alarm Timer with Calibration (Traceable, #UX-90225-35, Webster, TX)
- **Reclining Phlebotomy Chair** (Treatment Hight Recliner, Alo XL, 7349M1)(similar)

Background:

The 30-second chair stand test, a part of the larger short physical performance battery (SPPB), is completed during the psychophysiology session. This widely used test accesses lower body power, balance, and endurance (Beaudart et al., 2019).

Procedures:

Participants are instructed to sit and stand as many times as they can in 30 seconds. Feet are kept flat on the floor and arms are crossed in front of the chest to avoid use of the upper body. Participants are given the following standard encouragements:

After 10 seconds: "*10 seconds down. As fast as you can!*"

After 20 seconds: "*10 seconds to go. As fast as you can!*"

After 25 seconds: "*5 seconds left*"

After 30 seconds: "*You did it*"

The total number of sit-stands is recorded as well as the time to complete 5 total sit-stands.

Outcome Measures:

- Number of sit-stands repetitions (e.g., 18)
- Time to complete 5 sit-stands (e.g., 12 seconds)
- Energy expended during sit-stand task (round(([weight]*0.8125*(1/2.2046)*([height]/100-0.45)* [number of sit-stands]*10)/1000,2))
- Continuous physiological measurements (body temperature, blood pressure, respiratory rate, pulse rate, skin conductance)

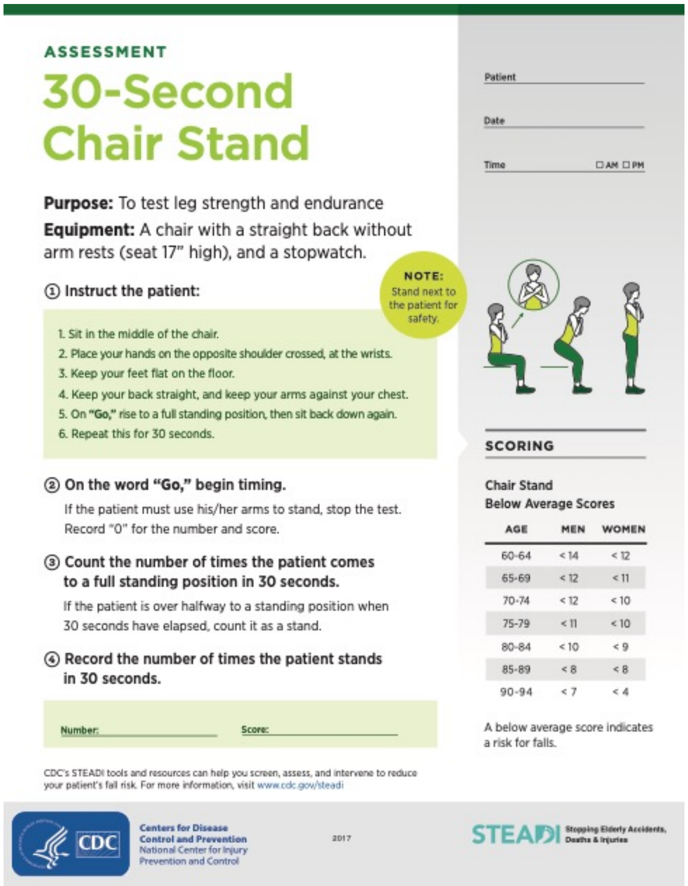

ASSESSMENT

30-Second Chair Stand

Patient

Date

Time ☐ AM ☐ PM

Purpose: To test leg strength and endurance

Equipment: A chair with a straight back without arm rests (seat 17" high), and a stopwatch.

NOTE: Stand next to the patient for safety.

① **Instruct the patient:**

1. Sit in the middle of the chair.
2. Place your hands on the opposite shoulder crossed, at the wrists.
3. Keep your feet flat on the floor.
4. Keep your back straight, and keep your arms against your chest.
5. On **"Go,"** rise to a full standing position, then sit back down again.
6. Repeat this for 30 seconds.

② **On the word "Go," begin timing.**

If the patient must use his/her arms to stand, stop the test. Record "0" for the number and score.

③ **Count the number of times the patient comes to a full standing position in 30 seconds.**

If the patient is over halfway to a standing position when 30 seconds have elapsed, count it as a stand.

④ **Record the number of times the patient stands in 30 seconds.**

Number: ______ Score: ______

SCORING

Chair Stand
Below Average Scores

AGE	MEN	WOMEN
60-64	< 14	< 12
65-69	< 12	< 11
70-74	< 12	< 10
75-79	< 11	< 10
80-84	< 10	< 9
85-89	< 8	< 8
90-94	< 7	< 4

A below average score indicates a risk for falls.

CDC's STEADI tools and resources can help you screen, assess, and intervene to reduce your patient's fall risk. For more information, visit www.cdc.gov/steadi

CDC Centers for Disease Control and Prevention National Center for Injury Prevention and Control 2017 STEADI Stopping Elderly Accidents, Deaths & Injuries

6.6 Cold Pressor Task

Materials:

Crushed Ice

- **10-liter bucket**
- **Digital Thermometer.** Traceable Digital Pocket Thermometer with Calibration, 572℉; 11.5 Long-Stem (Traceable Products, ISO 17025:2017, Galveston, TX, USA).
- **Digital Timer.** Traceable Four-Channel Alarm Timer with Calibration (Traceable, #UX-90225-35, Webster, TX)

Background:

The Cold Pressor Test is a reliable test to monitor pain-elicited physiological changes (von Baeyer et al., 2005).

Procedure:

Participants submerge their right hand, up to and including the wrist into a half-filled 10-liter container of water chilled to 3.1-3.4°C. Participants leave their hand in the water for 90 seconds, or for as long as they can manage. This period is followed by a 300-second recovery period. To achieve consistent temperature for the cold pressor test, tap water is cooled with -20°C crushed ice, stirred, and the temperature is monitored with a digital thermometer. Once the water temperature reaches 3°C, excess ice was removed with a strainer. The temperature reaches 3.1-3.4°C by titrating in warm water while stirring.

Before the Cold Pressor Test, the study coordinator delivers the following instructions:

> *"As it was explained in the consent form, we want to see how the body responds to a stimulus or stress. So, we are going to do a simple test to look at this. You'll stay sitting there and put your right hand all the way in with the wrist into a bucket of cold water that we'll bring here in a minute. Keep your hand in the water for as long as you can. I'll let you know when 90 seconds is over. I know that you may find it uncomfortable but that's normal because it's a standard test to stimulate the body. You are of course allowed to stop whenever you want, but it's important for the study that you keep your hand in there for as long as you can."*

Once participants place their hand in the water, the study coordinator starts the timer. The following standard feedback is given, "60 seconds left, 30 seconds left, 10 seconds left".

After the Cold Pressor Task, the following question is asked regarding pain perception, "Please rate how painful the task was from 0 (not painful at all) to 10 (extremely painful).

Ten minutes following the onset of the CP, participant temperature, affect rating, and saliva are collected. In addition to the three locations temperature is routinely taken, the temperature of the immersed, right, hand was also monitored.

Outcome Measures:

- Time hand submerged (seconds)

- Temperature before, immediately after, and 10 minutes after task
- Self-reported pain

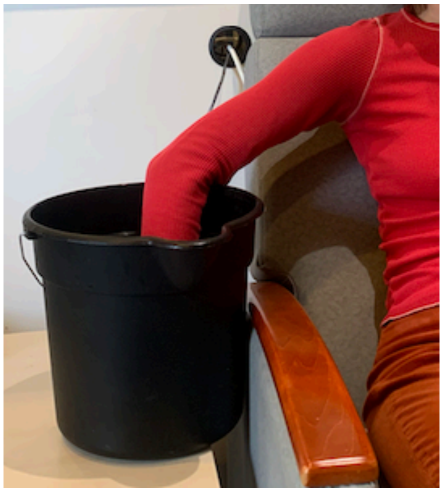

6.7 Resting Metabolic Rate (RMR)

Materials:

- **Bacterial Viral Filter**. (Korr Medical Technologies, 9FG0079, Salt Lake City, UT)
- **Reevue Metabolic Rate Analysis System**. (Korr Medical Technologies, CPT#94690, Salt Lake City, Utah)
- **Reevue Metabreather** (Korr Medical Techonologies, 9FG0185, Salt Lake City, Utah)
- **Reclining Phlebotomy Chair** (Treatment Hight Recliner, Alo XL, 7349M1)(similar)

Background:

Mitochondrial diseases are associated with impairments in oxidative phosphorylation (OxPhos) which can trigger hypermetabolism, or excess energy expenditure (Sturm et al., 2023). The *Reevue* device measures metabolic rate by analyzing end tidal oxygen tension and tidal volume, estimating oxygen consumption, which is converted to metabolic rate using a standard algorithm (https://korr.com/products/medical-metabolic-rate-analysis-system/).

Procedure:

The procedures are the same as Day 1 resting metabolic rate. Participants are given a disposable breathing tube and nose clip for the analysis. Participants are instructed to breathe normally through a one-way valve for ten minutes while they remain relaxed and still in a comfortable chair for the duration of the analysis. After the ten-minute analysis is complete, participants are asked if they felt air escaping from the mouthpiece and if they ever took the mouthpiece out of their mouth. In addition, the study coordinator notes whether participants moved during the analysis, which could confound (i.e., increase) metabolic rate due to the energetic cost of muscle contraction.

Outcome Variables:

- Resting Energy Expenditure (REE) (kcal/day)
- VO_2 (mlO_2/min)
- FeO_2 (%)
- Tidal volume (L)
- Minute ventilation VE (L/min)
- Respiratory rate (breaths/min)
- *Calculated*: Weight-adjusted VO_2 (mLO_2/min/kg)
- *Calculated*: Fat free mass-adjusted VO_2 (mLO_2/min/kg_ffm)

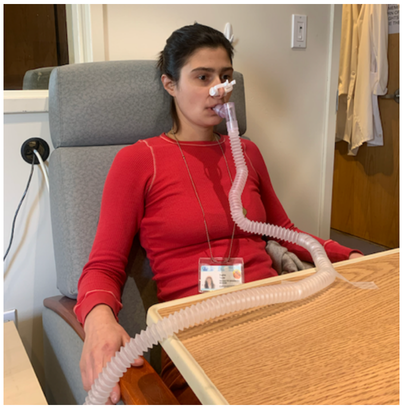

6.8 Verbal Time Estimation Task

Materials:

- **Digital Timer.** Traceable Four-Channel Alarm Timer with Calibration (Traceable, #UX-90225-35, Webster, TX)

Background:

A series of studies have demonstrated differences in durational judgement between older and younger participants (Block et al., 1998; Carrasco et al., 2001; Espinosa-Fernández et al., 2003). A time estimation task, modeled from previous durational estimate studies, was implemented in the MiSBIE study to better understand how mitochondrial biology and other bioenergetic parameters may influence time perception.

Procedure:

Time Estimation Task

Participants sit in a comfortable chair in a quiet room, across the table from the study coordinator. Participants complete two tasks: estimation and production. During each task, the study coordinator avoids eye contact with the participant or the timer, thus minimizing response bias. The study coordinator reads scripted feedback throughout the trials. Each of the two tasks (estimation and production) lasts approximately 10 minutes. For more details about task instructions and flow, please refer to verbal time estimation supplemental resources.

Outcome Variables:

- Duration judgement ratio (subjective to objective duration) for time interval estimation
- Duration judgement ratio (subjective to objective duration) for time interval production
- Intraindividual variation

Verbal Estimation:

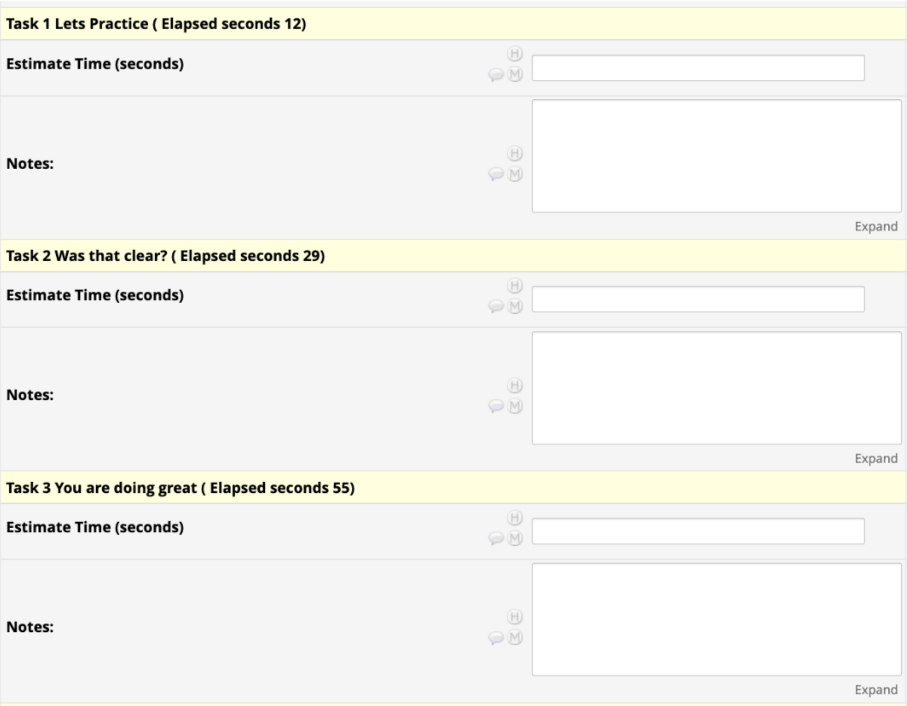

Task 1 Lets Practice (Elapsed seconds 12)

Estimate Time (seconds)

Notes:

Expand

Task 2 Was that clear? (Elapsed seconds 29)

Estimate Time (seconds)

Notes:

Expand

Task 3 You are doing great (Elapsed seconds 55)

Estimate Time (seconds)

Notes:

Expand

Production:

Task 1 Lets Practice (Elapsed seconds 10)

Estimate Time (seconds)

Notes:

Expand

Task 2 Was that clear? (Elapsed seconds 10)

Estimate Time (seconds)

Notes:

Expand

Task 3 You are doing great (Elapsed seconds 10)

Estimate Time (seconds)

Notes:

Expand

Task 4 (Elapsed seconds 10)

7 Medical assessment

Materials:

- **Karnofsky Performance Scale** (D. A. Karnofsky, 1949)
- **Columbia Neurological Scale** (CNS) (Kaufmann et al., 2009; Kaufmann, 2011; Kaufmann et al., 2004)
- **North American Mitochondrial Disease Consortium** (NAMDC) Case Report Form
- **Newcastle Mitochondrial Disease Assessment Scale** (NMDAS) (Schaefer et al., 2006)

Background:

Due to the heterogeneity of clinical presentations among individual with the same mutations, and to capture the nature and severity of disease in each participant (Moggio et al., 2014), a thorough medical assessment is completed, serving as a basis to later track mitochondrial disease progression.

Procedure:

A physician completes the listed scales in the following order: Karnofsky Performance Scale (KPS), Newcastle Mitochondrial Disease Assessment Scale (NMDAS), Columbia Neurological Scale (CNS), and North American Mitochondrial Disease Consortium (NAMDC). The eye exam, necessary for CNS and NMDAS, is completed at the end of the assessment to avoid having the short-term vision disturbance influencing other assessments.

We do not collect head circumference as part of the CNS assessment. Therefore, our scoring range is 0-75, rather than 0-76.

Measure	Description	Time (minutes)
**7.1 Columbia Neurological Score (CNS)**	Assessment of central nervous system symptoms	**30**
**7.2 Newcastle Mitochondrial Disease Assessment Scale (NMDAS)**	Semi-quantitative assessment of different domains of functioning	**40**
**7.3 NAMDC Case Report Form (CRF)**	Detailed assessment of clinical history, comorbidities, symptoms checklist	**30**
**7.4 Karnofsky Performance Scale Index**	Classifies functional impairment	**1**

8 Neuropsychological assessment

Materials

- **DKEFS Test**. Delis-Kaplan Executive Function System (Pearson Assessments, 0158091108, Coushatta, LA)
- **WASI-II.** Wechsler Abbreviated Scale of Intelligence Second Edition (Pearson Assessments, 0158981561, Coushatta, LA)
- **RBANS**. Repeatable Battery for the Assessment of Neuropsychological Status (Pearson Assessments, 0158006976, Coushatta, LA)
- **NAB**. Neuropsychological Assessment Battery (Psychological Assessment Resources, 11918-KD, Lutz, FL)
- **TOPF**. Test of Premorbid Functioning (Pearson Assessments, 015800972X, Coushatta, LA)

Background:

Cognitive impairments and mental disorders are common in mitochondrial diseases (Klein et al., 2021),possibly due to the high energy demands of the brain and the reduced energy production (OxPhos) capacity of this clinical population. To understand how the brain is impacted, we must understand both functional and structural impacts through neuropsychological testing and brain imaging.

Procedure:

All neuropsychological tests are administered according to specific test requirements and specifications. The study coordinator is trained and tested by a neuropsychologist prior to test administration. Due to the Covid-19 pandemic, this battery of tests has been delivered virtually starting in 2021, with the exception of interactive tests which require in-person administration. The tests included in this battery evaluate cognitive domains including: Premorbid functioning, Intellectual functioning, Visuospatial, Language, Memory, and Executive functioning and attention.

Cognitive Domains	Measure	Description
8.1 Premorbid Functioning	Test of Premorbid Functioning (TOPF)	Estimated verbal premorbid functioning
8.2 Intellectual Functioning	WASI-II - Vocabulary and Matrix Reasoning	General intellectual ability
8.3 Visuospatial	RBANS - Line Orientation	Visuospatial judgement
8.4 Language	D-KEFS - Verbal Fluency	Letter and category fluency, verbal set-shifting

	RBANS - Picture Naming	Confrontation naming
8.5 Memory	NAB - Shape Learning	Visual learning and memory
	RBANS - List Learning, List Recall, List Recognition	Verbal learning and memory
8.6 Executive Functioning and Attention	NAB - Numbers and Letters	Attention and inhibition
	D-KEFS - Trail Making	Working memory, cognitive flexibility and speed
	NAB - Digits Forward and Backward	Attention and working memory
	D-KEFS - Color-Word	Response inhibition
	D-KEFS - Sorting	Mental flexibility and conceptualization
	RBANS - Coding	Processing speed
		**Total duration: ~90 minutes**

9 Magnetic Resonance Imaging (MRI)

Materials:

- **Magnetic Resonance Imaging Scanner**. Magnetom Aera 3T (Siemens, SKU#14456150, Munich, Germany)
- **Magnetic Resonance Imaging Coil**. Head/Neck 64 channel (Siemens, SKU# 14456220, Munich, Germany)
- **Handball/squeezeball**. (Siemens SKU#04757246, Munich, Germany)
- **Button Box**. 4 Button Bimanual. (Current Design, SKU# HHSC-2x2, Philadelphia, PA)
- **Headphone System.** (Optoacoustics, EOU 7000, Mazor, Israel)

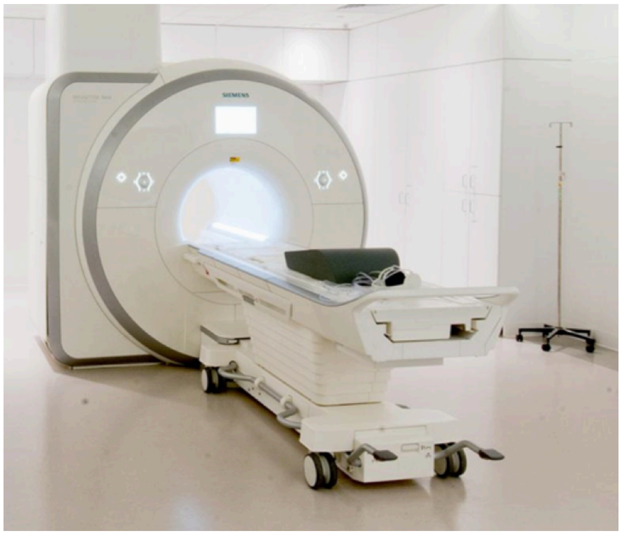

Background:

The high energetic demand of the brain makes it vulnerable to energy production deficits, characteristic of mitochondrial disease. Neurodegeneration is a common symptom of mitochondrial disease that leads to distinct patters of tissue damage in mitochondrial disease subtypes (Distelmaier & Klopstock, 2023). Using functional MRI, we can monitor how this tissue damage impacts brain activity.

Neuroimaging includes a family of techniques non-invasively measuring activity in the brain. Varieties include magnetic resonance imaging (MRI)-based and Positron Emission Tomography (PET), among others (Geuter, 2016; Hansen et al., 2022). MiSBIE includes three MRI-based types, including i) T1 and T2-weighted images that map gross anatomical structures and gray-matter variations; ii) diffusion-weighted images that can map white-matter tracts and their variation (Forkel & Catani, 2017), iii) functional Bold Oxygen-Level Dependent (fMRI-BOLD) images (Yang et al., 2021) that reflect regional brain signals related to blood flow and oxygen consumption on a second-by-second basis (Sterling, 1988).

Procedure:

On the second outpatient hospital visit (Day 2) that takes place at the Mortimer B. Zuckerman Mind Brain Behavior Institute (ZMBBI), we collect fMRI-blood oxygen level-dependent (BOLD) data during psychological tasks and rest. Specifically, we administer four tasks to assess brain function across basic sensory, threat/salience (Seeley, 2019), and working memory networks

(Hampson et al., 2006), which include the somatosensory cortex (Vierck et al., 2013), sensory cortex, insula, prefrontal cortex, and amygdala.,

Magnetic resonance imaging is used with a protocol consisting of one structural scan of high–resolution T1–weighted images, and fMRI-BOLD imaging is employed over task runs consisting of T2*weighted gradient–echo images. Scanning takes place on a Siements Magnetom Aera at ZMBBI.

Participants are escorted to ZMBBI on the second day of the study, on the first floor. Upon arrival, participants are ushered by the study coordinator to a private waiting room in the MRI suite on the basement floor of the ZMBBI building. Prior to the MRI scan, participants are given a brief presentation on what they can expect during their scan including the sights, sounds, and sensations they will experience. Participants are shown the MRI scan schedule and each scan is explained by the study coordinator.

After this brief presentation is complete, 1 mL of saliva is collected with a salivette, as per the previous day. Affect ratings (9 questions) are also collected, as per the previous day.

On the morning of Day 2, participants complete an MRI screening form to ensure participant safety. Participants are reminded to remove all jewelry and are given a pair of scrubs to wear during the scan. Upon entering the control room, participants are scanned with a metal detector.

The scanning protocol is 1.5 hours. Participants are positioned on the MRI table, using a plastic head cradle and coil to decrease head movement during the scan. Participants are given foam earplugs to reduce the noise of the MRI as well as MRI-compatible headphones for audio delivery and communication with MRI technician. Participants hold an MRI-compatible hand-held response pad is their left hand and an emergency squeeze ball in their right. A three-lead ECG is connected to participants to monitor heart rate responses in conjunction with brain activation.

The task instructions are projected from a liquid crystal display projector zoomed to a screen inside the MR suite or viewed through dual-channel binocular goggles. Functional fMRI-BOLD images are acquired.

To account for the accessibility needs of patients with visual processing issues or ophthalmoplegia, instructions and text during scanning are read aloud by the coordinator.

During each pause between acquisitions, participants are asked to rate their physical discomfort on a scale of 0-10. If the score rises above 5, the coordinator pauses the session and asks if participants wish to continue or if any adjustments can be made (i.e. change in position, an additional blanket for warmth, a sitting break).

As on Day 1, at two time points during the MRI imaging session saliva samples are collected, along with affect ratings (5 questions).

These fMRI tasks are administered in the following order:

Saliva sample and affect rating #12

(A) Localizer (1 run, 1 min)
Localizing of brain and defining region of interest.

(B) Field map (1 run, 1 min)
Acquisition of field map for multiband correction.

(C) T2 scan (1 run, 11:15 min)
T2-weighted images for detection of potential lesions and ventricles. Participants are instructed not to fall asleep.

(D) T1 structural scan (1 run, 5:21 min)
T1-weighted images for anatomical co-registration.

(E) Multisensory (1 run, 5 min)
Participants are trained and later exposed to the multisensory task. This task consists of visual (checkerboard), auditory stimulation (constant pitch sound), button push on the button box.

(F) Resting State Run #1 (1 run, 10:51 min)
Participants are reminded not to fall asleep.

Saliva sample and affect rating #13

(G) N-back task Run #1 (1 run, 4:28 min)
This working memory task (64) consists of 9 blocks presented in ABBA or BAAB order, interspersed by 25 s of resting fixation. On each of 360 trials, an image is presented for 2 s followed by an inter-stimulus-interval fixation cross for 1 s. Participants are instructed to respond as quickly and accurately as possible to each word with a button press indicating, “yes, this word matches the word presented n-trials ago” (a target response) or “no, this word does not match the one presented n-trials ago” (a nontarget response). The first 3 trials of each block are not included in the analysis; these trials are always nontargets in the 3-back block and therefore did not carry the same task demand as the remaining trials.

1.	**Scout**	**(0:14)**
2.	**T2 scan**	**(11:15)**
3.	**T1 scan**	**(5:21)**
4.	**Multisensory**	**(5:00)**
5.	**Resting state 1**	**(10:51)**
	**Affect 13**	**Saliva 13**
6.	**N-back task 1**	**(4:28)**
7.	**N-back task 2**	**(4:28)**
8.	**Socio-evaluative task**	**(6:00)**
9.	**Modified cold pressor**	**(6:00)**
10.	**DTI** (seq 1-2)	**(6:27)**
	**Affect 14**	**Saliva 14**
12.	**DTI** (seq 3-6)	**(14:37)**
	**De-instrumentation**	

(H) N-back task Run #2 (1 run, 4:28 min)
This is the second run of the N-back task. Instructions are the same as in “G”.

(I) Socio-evaluative task(1run,6min)
The procedure is the same as in day 1 except subjects are told that their “performance on the first speech task was slightly below average when compared with other participants’ speeches” and participants are asked to “try to be more persuasive when delivering this speech”. They are told that the speech will be delivered after the scan session to the expert who evaluated you yesterday. At the end of the task, participants are told that they were randomly selected not to give their speech, as in previous work. A pulse oximeter is hooked up to participants, and heart rate responses in conjunction with brain activation will be assessed.

(J)Modified Cold Pressor Task (1 run, 6 minutes)
This is a modified task from the cold pressor administered on Day 1. Participants are instrumented with a thermal arm wrap designed specifically for theMRI environment and are instructed as follows: “We will place this arm wrap around your hand and arm. It is quite cold and may be uncomfortable, but it will not injure you.” The arm wrap is a fabric-based foldable unit fitted with Velcro attachments for detachable gel packs, which are kept in a temperature-controlled freezer prior

(K) Diffusion Weighted Imaging – DWI (Seq 1-2, 6:27 min)

Saliva sample and affect rating #14

(L) Diffusion Weighted Imaging – DWI (Seq 3-6, 14:37 min)

Participants are then de-instrumented (chest electrodes removed) and recover their personal belongings that could not be introduced in the MRI room.

total scan time is (1 + 1 + 12 + 5 + 5 + 11 + 5 + 5 + 6 + 6 + 6 + 15 min) 90 minutes. The scanner is booked for 120 minutes for each participant to accommodate saliva samples, instrumentation/de-instrumentation with EKG, time between tasks, and additional setup time.

In this study, MRI data were collected using a 3 Tesla Magnetom Aera whole-body scanner (Siemens, SKU#14456150, Munich, Germany), equipped with a Head/Neck 64 head coil. Various acquisition parameters were set for each type of scan. For T2-weighted scans, the parameters included a repetition time (TR) of 3200 ms, an echo time (TE) of 562 ms, a flip angle (FA) of 90, a field of view (FOV) of 224x224 mm, and a matrix size of 320x320 mm. In the case of T1-weighted scans, the settings were TR: 2300 ms, TE: 3049 ms, FA: 8, FOV: 256x256 mm, and a matrix size of 256x256 mm. For tasks and resting state scans, the parameters were TR: 460 ms, TE: 29 ms, FA: 46, FOV: 248x248 mm, and a matrix size of 82x69.7 mm. Diffusion Weighted Imaging (DWI, or Diffusion Tensor Imaging) scans were conducted with a TR of 3500 ms, TE of 75 ms, FA of 90, FOV of 216x216 mm, and a matrix size of 120x120 mm. Finally, for field map scans, the parameters included TR: 546 ms, TE1: 3.98 ms, TE2: 6.44 ms, FA: 60, FOV: 248x248 mm, and a matrix size of 82x82 mm. These diverse and specific scanning parameters were meticulously chosen to optimize the quality and relevance of the MRI data for our study objectives.

In the afternoon of Day 2, we measure brain anatomy using T1- and T2-weighted magnetic resonance on a 3 T Siemens Prisma scanner (Siemens Medical Solutions). The sequence parameters for T1: echo time, 0.00349 s; repetition time, 2.3 s; flip angle, 8°; voxel size, 1 × 1 × 1 mm; slice thickness, 1 mm. Parallel imaging with a reduction factor of 2 was used. The parameter for T2: echo time, 0.562 s; repetition time, 3.2 s; flip angle, 120°; voxel size, 0.7 × 0.7 × 0.7 mm; slice thickness, 0.7 mm. Parallel imaging with a reduction factor of 2 was used.
We then collect functional MRI images using the same scanner to measure BOLD activity during a series of psychological tasks. The sequence parameters were as follows: echo time, 0.029 s; repetition time, 0.46 s; flip angle, 44°; voxel size, 3.02 × 3.02 × 3 mm; slice thickness, 3 mm. Slices were acquired with a multiband acceleration factor of 8.

9.1 MR-safe Electrocardiography

Materials:

Radiolucent Electrode. Con Med Cleartrace (ConMed, #2700-003, Largo, FL)

Electrocardiograph (ECG). (Siemens, PPU098, Munich, Germany)

Background:

Heart rate(HR) and heart rate variability(HRV) are known physiological symptoms of an acute stress response (Schubert et al., 2009).

Procedure:

Before entering Zone III (control room), participants are instrumented with four MRI-safe electrode leads. Leads are placed on the (1) right thoracic outlet and (3) in a line below the left pectoral muscles. Upon entering Zone III, the electrocardiogram is attached to the preplaced leads. The signal is checked prior to scanning procedures.

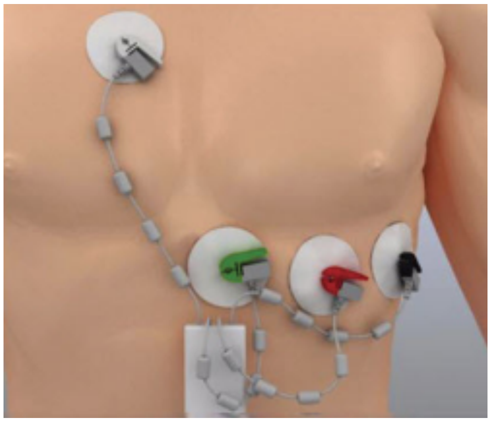

9.2 Multisensory Scan

Background

This fMRI task is based on the multisensory task used by Lopez-Solà et al. The multisensory stimuli consist of the simultaneous visual, flashing checkerboard grid of black and white alternating squares, and auditory stimulus, a series of 15 tones in frequency between 233.1 to 1318.5 Hz (Lopez-Sola et al., 2017).

Procedure

This block design fMRI task consists of alternating periods of multisensory stimulus and rest. Ten blocks are presented at 30-second intervals, the total scan time is 5 minutes. Prior to scanning, participants are asked to rate the volume of the tones to ensure a similar auditory experience between participants. Volume levels were adjusted until the participant reported the volume of tones was a 6 from 0 (not loud at all) – 10 (extremely loud).This scan includes alternating 30-sec periods of rest and a full-field, 3 Hz contrast-reversing checkerboard combined with auditory stimulation (15 tones from 233-1319 Hz).

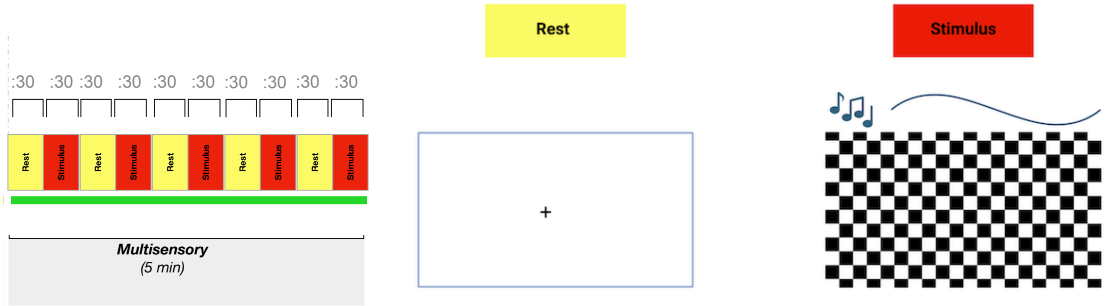

9.3 N-back Task

Background

The N-back task is an established tool to assess working memory and cognitive control in an fMRI environment (Barch et al., 2013; Drobyshevsky et al., 2006). This task was introduced by Kirchner to target participants' memory; participants are asked to recall items visually presented n items back (Kirchner, 1958). The version of the N-back task that we run, a block design fMRI paradigm, is based on that used in the Human Connectome fMRI task (Barch et al., 2013).

Procedure

This task includes alternating blocks or 0-back and 2-back working memory tasks. The images include faces, man-made objects, body parts, and places. Participants complete two blocks of these tasks; each block is 4 minutes and 35 seconds with a short break in between. Prior to scan start, participants are given instructions for the 0-back and 2-back tasks. They play a short practice game on a computer to ensure an appropriate understanding of the task.

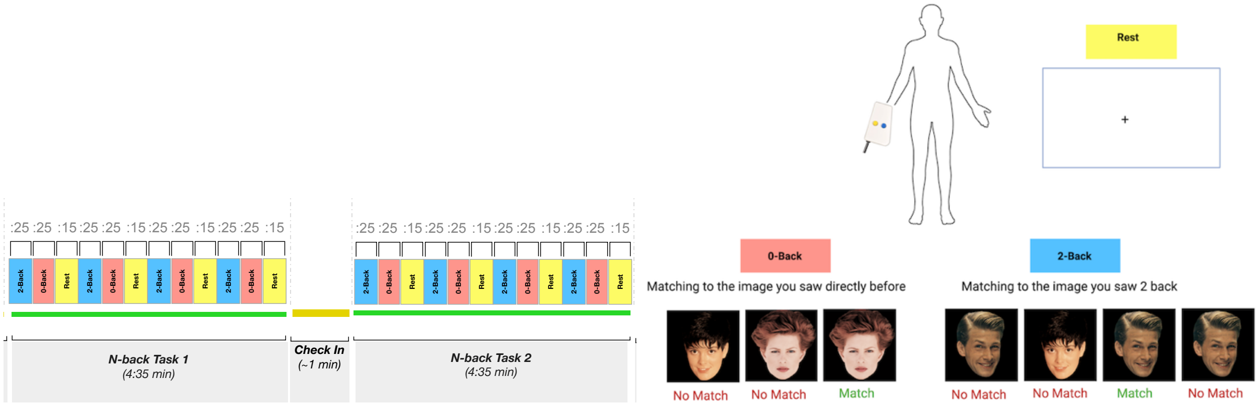

9.4 Modified Speech Task

Background

To access the stress response to a psychosocial stress, a modified version of the Trier Social Stress Test is utilized. This psychosocial stressor has been used as a reliable situation to induce and measure the stress response (Jezova et al., 2004). Brain patterns, captured by fMRI, have been shown to predict heart rate and skin conductance levels, two established markers of stress (Eisenbarth et al., 2016). Wager et al. identified two cortical-subcortical pathways implicated in the translation of social threat into physiological responses (Wager, van Ast, et al., 2009; Wager, Waugh, et al., 2009).

Procedure

Prior to entering the scanner, participants are told that, similar to Day 1, they would be asked to prepare and deliver a speech. They were told they would be given the speech prompt and time to prepare in the scanner and directly after the scan their speech would be audiotaped.

The task is 6 minutes in total. Prior to the scan, simple instructions are read aloud to participants and projected. Participants are told their performance on the first speech task, Day 1, was below average when compared with other participants and to try to be more persuasive when delivering

this speech. The scan begins with two minutes of rest. The speech prompt is then projected and read aloud, participants are accused of running a stop sign and are. asked to defend themselves to a court judge. After instructions are read, participants are given two minutes to prepare their speech. After two minutes, all participants are told they have been randomly selected to not deliver their speech and to relax for the remainder of the scan. Participants rest for approximately one minute. For more details on speech task instructions, please refer to speech task day 2 supplemental resources.

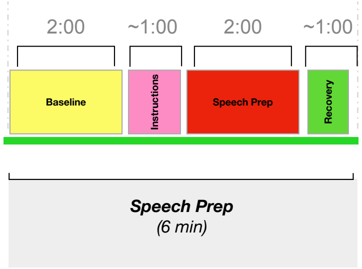

9.5 Modified Cold Pressor

Materials:

Cold Wrap (Similar Model) FlexiKold Gel Cold Pak (Natracure, A6300-COLD, Whippany, NJ)

Background:

The Cold Pressor Arm Wrap (CPAW) is a practical alternative to the Cold Pressor Test (CPT), which typically involves a participant holding their hand in very cold water for several minutes. The traditional Cold Pressor Test would be impossible to complete during the magnetic resonance imaging (MRI) procedure due to safety considerations, the Cold Pressor Arm Wrap is used instead. CPAW results in the activation of the sympatho-adrenomedullary (SAM) axis and hypothalamic-pituitary-adrenal (HPA) axis activation, similar to the CPT (Porcelli, 2014). The Cold Pressor Arm Wrap test takes place during the MRI scan, specifically between diffusion tensor imaging (DTI) sequences 1-2 and diffusion tensor imaging (DTI) sequences 3-6.

Procedure:

The CPAW is guided by an e-prime script which ensures standard timing of the following procedures. The study coordinator must first place the room temperature wrap around participants' right hand for 90 seconds. The study coordinator holds the pack to ensure it covers participants' entire right hand and wrist. After 90 seconds, the room temperature pack is removed. There is a buffer period of 30 seconds before the cold pack is applied. The cold pack is then applied to cover participants' entire hand and wrist for two minutes. After two minutes, the cold pack is removed. There is a second buffer period of 30 seconds before the room temperature wrap is applied again. The room temperature wrap is then applied for 90 seconds. After 90 seconds, the room-temperature wrap is removed. This marks the end of the Cold Pressor Arm Wrap (CPAW) and the diffusion tensor imaging scan resumes.

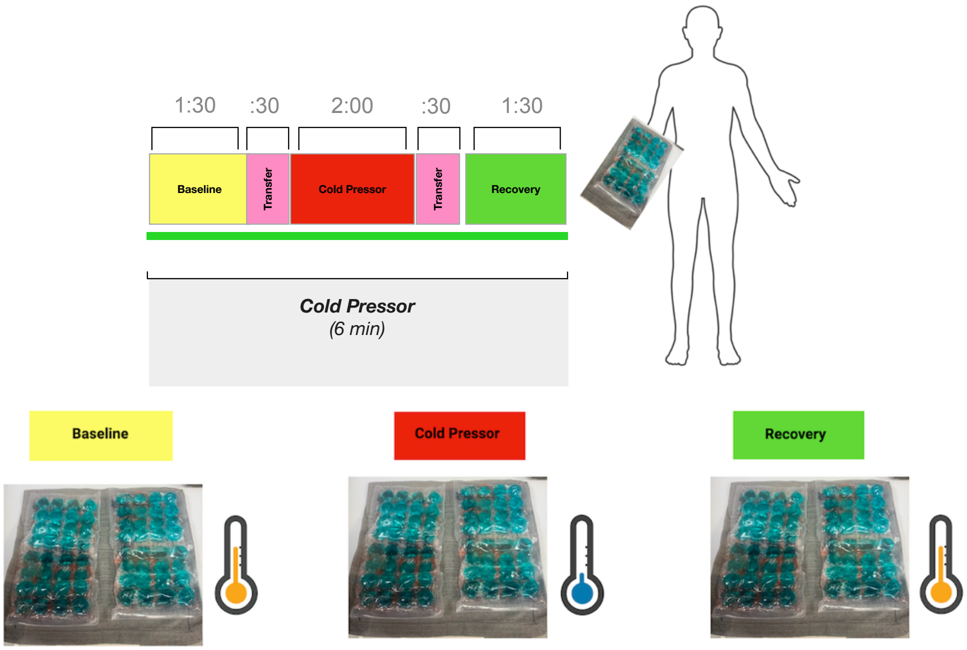
1:30
:30
2:00
:30
1:30
Baseline
Transfer
Cold Pressor
Transfer
Recovery
Cold Pressor
(6 min)
Baseline
Cold Pressor
Recovery

10 Self-report questionnaires

Background:

Self-reported questionnaires provide meaningful insights into the life habits, personality, and life experiences of research study participants. The following questionnaires were carefully selected based on their reliability and importance. The full list of questionnaires, average administration times, and references are available in Supplemental Table 2.

Procedures:

Most self-report questionnaires are administered in person via iPad at the study site. Day 0 and Day 1 Hotel questionnaires are administered and completed on paper. The Stress and Adversity Inventory (STRAIN) is completed via iPad at the hotel on Tuesday evening (Day 1).

10.1 Psychosocial and Personality

- Antonovsky's Sense of Coherence (SOC)
- Bem Sex Role Inventory Long Form (BSRI-L)
- Couples Satisfaction Index (CSI-16)
- MacArthur ladder (ML)
- NEO Five factor Model of Personality Short Form (NEO-SF)
- Perceived age in years (Perc_age)
- Perceived Social Support (pssq_frd)
- Primary Appraisal Secondary Appraisal (PASA)
- Revised UCLA Loneliness Scale Version III (ULS-8)
- Self-Perception of Aging (SPA)
- Social Support Questionnaire (SSQ)
- State and Trait Anxiety Inventory (STAI-Y1)

10.2 Mental Health and Wellbeing

- Beck Depression Inventory II (BDI)
- Daily Hassles Scale (DHS)
- DSM-5 Self-Rated Level 1 Cross-Cutting Symptom Measure—Adult
- Maslach Burnout Inventory (MBI)
- Modified Differential Emotions Scale (mDES)
- Daily Inventory of Stressful Events (DISE)
- Perceived Stress Scale (PSS)
- PTSD Check List – Civilian, PTSD Checklist for DSM-5 (PCL-C)
- Ryff's Psychological Well-Being Scale (PWBS)
- Trier Inventory for the Assessment or Chronic Stress (TICS)

10.3 Life Events

- Childhood Trauma Questionnaire (CTQ)
- Life Events Questionnaire (LEQ)
- Stress and Adversity Inventory for Adults (STRAIN)

10.4 Health Related Behaviors

- ☐ Food Frequency Inventory (FFI)
- ☐ International Physical Activity Questionnaire (IPAQ)
- ☐ MESA Vitamin Use subscale (MESA)
- ☐ Morningness and Eveningness Questionnaire (MEQ)
- ☐ Pittsburg Sleep Quality Index (PSQI)
- ☐ Pittsburgh Fatigability Scale (PFS)
- ☐ Short Questionnaire for Fat and Free Sugars (SQFFS)
- ☐ Stanford Leisure-Time Activity Categorical Item (L-Cat)

10.5 Demographic

- ☐ Education history
- ☐ Language history
- ☐ Participant Information (PI)

10.6 Physical and Cognitive Symptoms

- ☐ Composite Autonomic Symptom Score (COMPASS 31)
- ☐ Memory Complaint Checklist (MCC)
- ☐ Modified Fatigue Impact Scale (MFIS)
- ☐ SF-36 SRH Question (SRH)
- ☐ The Brief Illness Perception Questionnaire (BIPQ)

10.7 Affect Ratings

Throughout study participation, participants are asked to reflect on their current emotional state a total of 14 times. This questionnaire was derived and adapted from the Positive and Negative Affect Scale (PANAS).

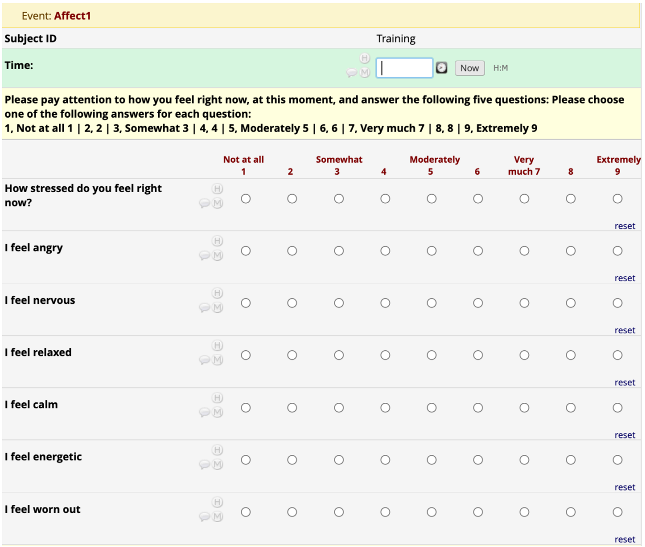

Event: **Affect1**

Subject ID Training

Time: Now H:M

Please pay attention to how you feel right now, at this moment, and answer the following five questions: Please choose one of the following answers for each question:
1, Not at all 1 | 2, 2 | 3, Somewhat 3 | 4, 4 | 5, Moderately 5 | 6, 6 | 7, Very much 7 | 8, 8 | 9, Extremely 9

	Not at all 1	2	Somewhat 3	4	Moderately 5	6	Very much 7	8	Extremely 9
How stressed do you feel right now?	○	○	○	○	○	○	○	○	○ reset
I feel angry	○	○	○	○	○	○	○	○	○ reset
I feel nervous	○	○	○	○	○	○	○	○	○ reset
I feel relaxed	○	○	○	○	○	○	○	○	○ reset
I feel calm	○	○	○	○	○	○	○	○	○ reset
I feel energetic	○	○	○	○	○	○	○	○	○ reset
I feel worn out	○	○	○	○	○	○	○	○	○ reset

Day 0
SF-36 SRH Question (SRH)
Modified Differential Emotions Scale (mDES)
The Daily Inventory of Stressful Events (DISE)
**Day 1 Post -Breakfast**
Participant Information (pi)
Stanford Leisure-Time Activity Categorical Item (L-Cat)
International Physical Activity Questionnaire (IPAQ)
Short Questionnaire for Fat and Free Sugars (SQFFS)
Food Frequency Inventory (FFI)
MESA Vitamin Use subscale
**Day 1 and 2**
Primary Appraisal Secondary Appraisal (PASA)
**Day 1 Recovery**
Modified Fatigue Impact Scale (MFIS)
Composite Autonomic Symptom Score (COMPASS 31)
The Brief Illness Perception Questionnaire (BIPQ)
Self Perception of Aging (SPA)
Language history
Education history
**Day 1 Hotel**
Perceived age in years (Perc_age)
MacArthur ladder (ML)
Morningness and Eveningness Questionnaire (MEQ)
Pittsburg Sleep Quality Index (PSQI)
Pittsburgh Fatigability Scale (PFS)
Social Support Questionnaire (SSQ)
Modified Differential Emotions Scale (mDES)
The Daily Inventory of Stressful Events (DISE)
Stress and Adversity Inventory for Adults (STRAIN)
**Day 2 Part 1**
Ryff's Psychological Well-Being Scale (PWBS)
Couples Satisfaction Index (CSI-16)
Antonovsky's Sense of Coherence (SOC)
NEO Five factor Model of Personality Short Form (NEO-SF)
Bem Sex Role Inventory Long Form (BSRI-L)
Memory Complaint Checklist (MCC)
Perceived Social Support (pssq_frd)
**Day 2 Part 2**
Life Events Questionnaire (LEQ)
Perceived Stress Scale (PSS)
Trier Inventory for the Assessment or Chronic Stress (TICS)
Daily Hassles Scale (DHS)
State and Trait Anxiety Inventory (STAI-Y1)
Revised UCLA Loneliness Scale Version III (ULS-8)
Beck Depression Inventory II (BDI)
Maslach Burnout Inventory (MBI)
PTSD Check List – Civilian, PTSD Checklist for DSM-5 (PCL-C)
Stress and Adversity Inventory for Adults (STRAIN)
**Day 2 Part 3**
DSM-5 Self-Rated Level 1 Cross-Cutting Symptom Measure—Adult
Childhood Trauma Questionnaire (CTQ)
**Home**
Modified Differential Emotions Scale (mDES)
The Daily Inventory of Stressful Events (DISE)

11 Home-Based Sample Collection

Day 1 Monday	Day 2 Tuesday	Day 3 Wednesday	Day 4 Thursday	Day 5 Friday
Awakening Saliva Sampling<br>Morning Questionnaire<br>+30 min Saliva Sampling<br>+45 min Saliva Sampling<br>Evening Questionnaire<br>Bedtime Saliva Sampling	Morning Questionnaire<br>Evening Questionnaire	Awakening Saliva Sampling<br>Morning Questionnaire<br>+30 min Saliva Sampling<br>+45 min Saliva Sampling<br>Evening Questionnaire<br>Bedtime Saliva Sampling	Morning Questionnaire<br>Evening Questionnaire	Awakening Saliva Sampling<br>Morning Questionnaire<br>+30 min Saliva Sampling<br>+45 min Saliva Sampling<br>Evening Questionnaire<br>Bedtime Saliva Sampling

11.1 MiSBIE Home Logbook

Background:

Home based biospecimen sampling and questionnaire completion provide meaningful information about daily mood, steroid hormonel awakening patterns, and sleep schedules/quality that would not have been possible in our laboratory setting alone. This information is gathered through an iPad customized and simplified for our study purposes. Home-based sample (saliva) collection is accompanied by daily stress and mood ratings (Trumpff et al., 2022).

Procedure:

We provide all participants with an iPad, set in single-app mode to the MiSBIE app. This app provides an easy-to-follow schedule, has sample collection reminders and timers, and keeps detailed records of sample collection times. Participants complete a short morning and evening questionnaire on the MiSBIE app on all five days of their sample collection week, Monday – Friday. Participants collect saliva samples and note this collection in the app four times on Monday, Wednesday, and Friday (12 samples).

Participants are asked to complete a daily morning and evening questionnaire on the provided iPad. The questionnaires include the Daily Stress questionnaire (morning) and the Modified Differential Emotions Scale (evening).

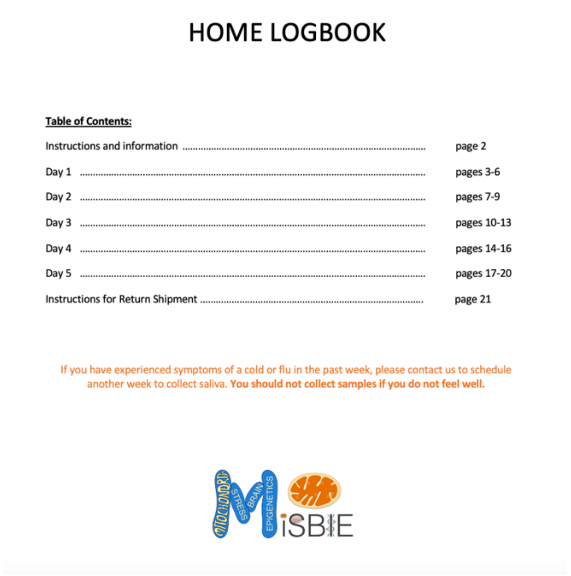

HOME LOGBOOK

Table of Contents:

Instructions and information .... page 2
Day 1 .... pages 3-6
Day 2 .... pages 7-9
Day 3 .... pages 10-13
Day 4 .... pages 14-16
Day 5 .... pages 17-20
Instructions for Return Shipment .... page 21

If you have experienced symptoms of a cold or flu in the past week, please contact us to schedule another week to collect saliva. **You should not collect samples if you do not feel well.**

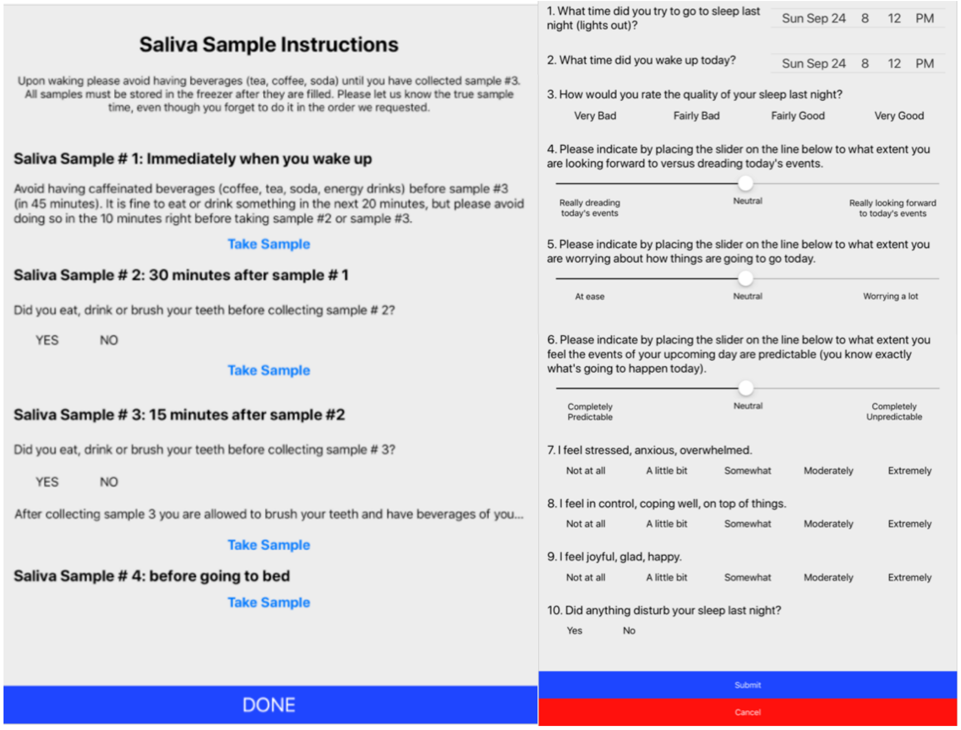

11.2 Saliva Samples

Materials:

Salivette (Starstedt, Cat# 51.1534.500, Numbrecht, Germany)

Background:

Free cortisol is measured in saliva (Kirschbaum & Hellhammer, 1994). Mitochondria also release their genetic material as cell-free mitochondrial DNA (cf-mtDNA) in biofluids including blood and saliva under acute stress (Trumpff et al., 2022). Saliva samples collected non-invasively may be used to probe stress physiology and to develop biomarkers of health.

The cortisol awakening response (CAR) is the sharp increase in cortisol secretion over the first 30-45 minutes following awakening (Stalder et al., 2022). The CAR is a standard measure of hypothalamic- pituitary-adrenal (HPA) axis activity.

Procedure:

At home, participants collect 4 daily saliva samples on 3 non-consecutive days, for a total of 12 samples. Participants take samples on Monday, Wednesday, and Friday at the following points: upon awakening, +30 minutes after awakening, +45 minutes after awakening, at bedtime. Each participant is given an iPad that is set in single app mode to the MiSBIE app. Like our in-person procedures, participants place the cotton salivette in their mouth, specifically in the middle of their tongue, for 2-5 minutes without biting on the swab or moving it. Afterward, participants reinserted the cotton swab into the salivette recipient tube. salivettes were frozen immediately after collection in a home freezer (typically -20°C). During at-home morning collections, individuals were advised to delay tooth brushing and eating until after the third sample (+45 minutes), while the nighttime sample was to be taken prior to bedtime tooth brushing. Participants were instructed to avoid consuming water or any other liquids within 10 minutes of each saliva sample collection. This was monitored and controlled for the laboratory samples. Within 2 weeks of collection, participants either transported (10%) or shipped them (90%) to the laboratory in a non-temperature controlled, pre-stamped USPS priority shipping.

Outcome Variables:

- Salivary steroid hormones (cortisol, cortisone, DHEA, testosterone, progesterone, corticosterone)
- Salivary cf-mtDNA
- GDF15

11.3 Fecal Sample Collection

Materials:

Fecal Collection Kit. Omnigene Gut for microbiome (DNA Genotek, #OMR-200, Ottawa, ON, Canada)

Background:

There is growing evidence for the connection between health and gut microbiota. Disruptions in the gut microbiota are evident in diseases such as diabetes, obesity, and inflammation (Rieder et al., 2017). There is also evidence linking gut microbial health to mental health conditions, including depression (Jarbrink-Sehgal & Andreasson, 2020).

Procedure:

Participants collect a small fecal sample for microbial analysis. The collection kit includes a collector container with stabilizing liquid, spatula, and toilet accessory. The study coordinator completes a demonstration to ensure proper specimen collection. Participants use the toilet accessory to avoid sample contamination in toilet water. With the provided spatula, a small sample is collected and store. Participants shake the sample for 30 seconds to ensure homogenization and stabilization and freeze the sample in a standard freezer until shipment to the lab for processing. Participants are reminded via text to complete this sample on Wednesday of their at-home sample collection week, but it is possible that they complete the sample at any time over this week.

Outcome Variables:

Microbiome analysis (pending)

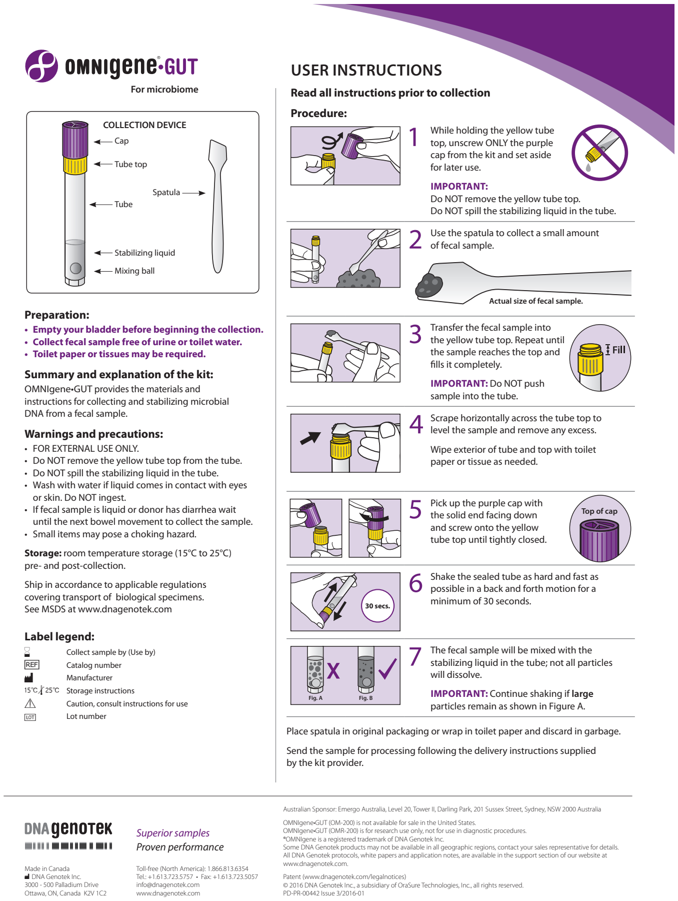

OMNIgene•GUT
For microbiome

COLLECTION DEVICE
Cap
Tube top
Tube
Spatula
Stabilizing liquid
Mixing ball

Preparation:
- **Empty your bladder before beginning the collection.**
- **Collect fecal sample free of urine or toilet water.**
- **Toilet paper or tissues may be required.**

Summary and explanation of the kit:
OMNIgene•GUT provides the materials and instructions for collecting and stabilizing microbial DNA from a fecal sample.

Warnings and precautions:
- FOR EXTERNAL USE ONLY.
- Do NOT remove the yellow tube top from the tube.
- Do NOT spill the stabilizing liquid in the tube.
- Wash with water if liquid comes in contact with eyes or skin. Do NOT ingest.
- If fecal sample is liquid or donor has diarrhea wait until the next bowel movement to collect the sample.
- Small items may pose a choking hazard.

Storage: room temperature storage (15°C to 25°C) pre- and post-collection.

Ship in accordance to applicable regulations covering transport of biological specimens. See MSDS at www.dnagenotek.com

Label legend:
Collect sample by (Use by)
REF Catalog number
Manufacturer
15°C 25°C Storage instructions
Caution, consult instructions for use
LOT Lot number

USER INSTRUCTIONS

Read all instructions prior to collection

Procedure:

1 While holding the yellow tube top, unscrew ONLY the purple cap from the kit and set aside for later use.
IMPORTANT:
Do NOT remove the yellow tube top.
Do NOT spill the stabilizing liquid in the tube.

2 Use the spatula to collect a small amount of fecal sample.
Actual size of fecal sample.

3 Transfer the fecal sample into the yellow tube top. Repeat until the sample reaches the top and fills it completely.
IMPORTANT: Do NOT push sample into the tube.
Fill

4 Scrape horizontally across the tube top to level the sample and remove any excess.
Wipe exterior of tube and top with toilet paper or tissue as needed.

5 Pick up the purple cap with the solid end facing down and screw onto the yellow tube top until tightly closed.
Top of cap

6 Shake the sealed tube as hard and fast as possible in a back and forth motion for a minimum of 30 seconds.
30 secs.

7 The fecal sample will be mixed with the stabilizing liquid in the tube; not all particles will dissolve.
IMPORTANT: Continue shaking if **large** particles remain as shown in Figure A.
Fig. A
Fig. B

Place spatula in original packaging or wrap in toilet paper and discard in garbage.

Send the sample for processing following the delivery instructions supplied by the kit provider.

DNA genotek
Superior samples
Proven performance

Made in Canada
DNA Genotek Inc.
3000 - 500 Palladium Drive
Ottawa, ON, Canada K2V 1C2

Toll-free (North America): 1.866.813.6354
Tel.: +1.613.723.5757 • Fax: +1.613.723.5057
info@dnagenotek.com
www.dnagenotek.com

Australian Sponsor: Emergo Australia, Level 20, Tower II, Darling Park, 201 Sussex Street, Sydney, NSW 2000 Australia

OMNIgene•GUT (OM-200) is not available for sale in the United States.
OMNIgene•GUT (OMR-200) is for research use only, not for use in diagnostic procedures.
®OMNIgene is a registered trademark of DNA Genotek Inc.
Some DNA Genotek products may not be available in all geographic regions, contact your sales representative for details.
All DNA Genotek protocols, white papers and application notes, are available in the support section of our website at www.dnagenotek.com.

Patent (www.dnagenotek.com/legalnotices)
© 2016 DNA Genotek Inc., a subsidiary of OraSure Technologies, Inc., all rights reserved.
PD-PR-00442 Issue 3/2016-01

11.4 Actigraphy

Materials:

Actiwatch 2 Device (Phillips, 1048090, Bend, OR)

Background:

Actigraphy devices, such as the Actiwatch 2, have been validated as successful tools to estimate sleep onset latency (SOL), total sleep time (TST), and sleep efficiency (SE) (Shin et al., 2015).

Procedure:

The study coordinator configures the Actiwatch device with participant demographic information. Devices are set to collect data for up to 15 days with an epoch length of 30 seconds. Participants wear the device for 10 days, other than bathing or swimming to avoid physical damage to the device. At the end of Day 2, prior to leaving, participants begin wearing the Actiwatch, they remove the Actiwatch at the end of their sample collection week. Participants are given paper instructions(below) in addition to this demonstration.

Outcome Variables:

- -Sleep onset latency (SOL)
- -Total sleep time (TST)
- -Sleep efficiency (SE)

<u>Actiwatch</u>

- The Actiwatch will be activated when you receive it – No need to turn it on.
- Press the silver button to let us know when you're going to sleep & when you wake up.
- The Actiwatch is water-resistant, but shouldn't be submerged in water for too long (i.e., while swimming or bathing).
- Please wear the watch for 10 days. You may remove the watch next Saturday morning.

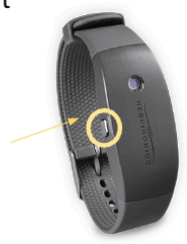

12 <u>Supplemental Resources</u>

12.1 Purifying Leukocyte Subtypes Numbered Protocol

PBMC and sub-type purification on Ficoll (1077 and 1119) gradient

For MHI, Seahorse, and molecular analyses.

This protocol describes procedures to purify PBMC and to isolate monocytes, neutrophils, lymphocytes and platelets using a double Ficoll gradient and magnetic bead separation, adapted from Kramer et al. (*JOVE* 2014). Cell pellets will be stored at -170°C for various measurements.

The night before:

In TC1/room 425:

Fridge, then set in fume hood:

- Take out Ficoll (1077 and 1119) to room temperature
- Take out DPBS (1X) to room temperature
- Take out 500ml of HBSS buffer to room temperature
- Take out 100ml of HBSS/0.5% BSA buffer to room temperature

BSC-2 Hood (white-top, tissue culture tubes):

- Label 3 X 15ml tubes (1x “Ficoll (1077),” 1x “Diluted blood,” 1x “Cells”)
- Label 3 X 15ml tubes (1x Platelet Rich Plasma “**PRP**,” 1x “**PRPlatelets**,” 1 x inhibitor “**PI2**”)
- Label 2 X 50ml tubes (“1077/1119 Ficoll”)
- Label 1 X 50ml tube (“Buffy coat”)
- Label 2 X 50ml tubes (1 x MCN diluted gradient “**MCN**,” 1 x PMN diluted gradient “**PMN**”)
- Label 5 X 15ml tubes (2 x Monocyte as “**Mono” (+/-)**, 2 x Neutrophils as **“Neut” (+/-)**, 1 x Lymphocytes as **“Lym (-)”**
- Label 3 X 1.5ml tube – (1 x “**M14**,” 1 x “**P15**,” 1 x “**LYM**”)
- Label 3 X 15ml tubes (1x “Ficoll (1077),” 1x “Diluted blood,” 1x “Cells”) [afternoon]

On personal bench in main lab area (green or purple top general-use tubes):

- Label 4 X 15ml tubes for **plasma** and **serum** (2 x “Pooled”, 2 x “Clean”)
- Label 32 X 15ml Tubes (16 x **P.**Stress -5 through +120 and **S.**Stress -5 through +120) → keep 4°C

  Note: if labeling the day-of, put in 4°C a few hours before use

Morning of Procedure:

In TC1/425:

- Set Necropsy Room centrifuge to 4°C (Program 1)
- Get “MiSBIE: Freezer Transfer” bag from Necropsy Room; fill styrofoam with wet ice and place Sample Blocks inside (including blue wire 15ml tube rack)
- Place 9x9 and 10x10 MiSBIE storage boxes in shelf in -80
- Can prep 15ml PBS in “PGI2,” 2ml HBSS in “Cells,” 5ml HBSS in “**MCN**” & “**PMN**”

Seven types of tubes are used for different blood products:

- For Whole blood assessment: 3ml Lavender top, EDTA coated tubes x1 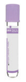
- For: 2.7ml Blue top, Sodium Citrate coated tubes x1 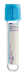
- For **Serum Separation**: 8ml Gold top, non-coated tubes x2 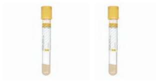
- For **Single cell** analysis: 2.7ml Blue top, Sodium Citrate coated tubes x4 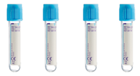
- For **Serum**: 8ml Red top, non-coated tubes x2 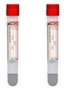
- For **Plasma**: 8ml Purple top, EDTA coated tubes x2 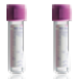
- For **Cells**: 8.5ml Yellow top, Acid Citrate Dextrose (ACD-A) x5 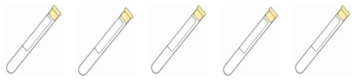

Start Time:

1. BLOOD DRAW → FIRST SPIN

1.1. Stand in room with participant holding blue bag
- When handed tube, gently invert x 8-12 (quickly enough to keep up with flow)

1.2. In blue bag: 1 lavender top EDTA, 1 blue sodium citrate, 2 gold top non coated

1.3. For PURPLE **plasma** tubes, immediately after blood draw is completed centrifuge **BREAK OFF** at 1,000g x 5 min RT and then place on wet ice.
- Plasma, buccal swab, + salivette are transported on wet ice; everything else is transported at RT in rack

1.4. When handed **RED** (serum) tubes, set timer for 30 minutes, and leave at room temperature for transfer
1.4.1. At 30 minutes, spin **RED** (serum) tubes **plasma** tubes 2,000 g x 10 min at 4 degree Necropsy

1.5. Buccal swab is collected; when given, label top of tube, and place in wet ice, same as salivate

--- TRANSPORT TUBES TO LAB ---

1.6. ***Start **PBMC** Isolation: Spin ACD-A tubes **BREAK OFF** at 500g x 15min RT

1.7. Take out PG12 inhibitor (1mM) from F-30 Freezer Shelf 1 Rack 3.

1.8. Add 15ul PGI2 inhibitor to 15mL PBS in "PGI2" tube; set aside for later.

Checkpoint #1:
- ☐ **PGI2 retrieved**
- ☐ **Ficoll gradients ready**

2. PURIFYING PBMCS AND LEUKOCYTE SUBTYPES

(All steps are done at room temperature in the Tissue Culture Room)

Ficoll Preparation:

2.1 **PBMC:** Place 4 ml of Ficoll (Ficoll-Paque® PLUS VWR Cat# 95021-205) in the 15ml “Ficoll 1077” conical tube (for PBMC isolation).

2.2 **Leukocytes:** Prepare the two 50ml “1077/1119” conical tubes with double ficoll gradient by using 1077 as the top layer and 1199 as the bottom layer. Using serological pipette, add 10ml of 1077 to the bottom of the 50ml tube, then add 10ml of 1119 to the bottom of the tube SLOWLY.

2.2.1 Total 20ml of density gradient added to each. Can SLOWLY hold up to light to see shadow indicating gradient is intact.

Plasma Replacement (PBMC) & Diluting Buffy Coat (Leukocytes):

2.3 **PRP:** Use 1ml pipette to aspirate 3ml of plasma from each of the 5 ACD-A tubes; pool in the 15ml “**PRP**” collection tube

2.4 Add 15µl PGI2 inhibitor to “**PRP**” tube; set aside for later.

- Final [PGI2] of 1µM

2.5 **PBMCs:** Pour the first collected ACD-A tube into the 15ml “Diluted blood” tube

2.6 Use 10ml sero pipette to add 5mL of HBSS to that ACD-A tube; pipette around to collect cells; dispense into “Diluted blood” tube.

2.7 From the 4 remaining ACD-A tubes, extract 3ml of buffy coat; pool in the 50ml “Buffy coat” tube.

Note: The buffy coat is at the interface of the plasma and blood. Hold pipette above the interface so that when you pipette, the interface gets sucked UP into the pipette (you’ll see RBCs trying to jump up too - pause suction to avoid this). In the final pipette, you will probably get some RBCs, but should be minimal). Total volume = 12ml

2.8 Recap each ACD-A tube and dispose in the sharps container

2.9 Add HBSS to “Buffy coat” until total volume = 50ml (this is ~4x the starting buffy coat volume)

2.9.1 Invert ~10 times

Ficoll Overlay:

2.10 Set serological pipette to lowest dispense speed.

2.11 **PBMCs:** Use 10ml sero pipette to aspirate contents of “Diluted blood” tube (~9-10ml)

2.12 SLOWLY use this to overlay “Ficoll 1077” tube

Tip: even before dispensing, the pipette tip is wet; use this to draw a 2-way “Y” shape on the tube to reduce disruption of the Ficoll interface. (***Figure 1**, #1 & 2*). Then raise the pipette to near the top of the 50ml tube, ‘attached’ to the blood-residue “Y” shape, and begin slowly dispensing. If one branch of the “Y” is weaker, can pause dispensing to try to grow it.

Gently manipulate tubes VERY slowly to avoid disrupting the interface

2.13 Centrifuge immediately **BREAK OFF** at 400g x 30 min RT (total ~35min), in a horizontal rotor (swing-out head) in tabletop centrifuge. **Spin along with Anna’s 50 mL conical tube labeled .3**

Note: If only processing two 15ml tubes, add two 50ml falcon tubes to the other two positions in the centrifuge to create inertia and make the centrifugation smoother for the Ficoll spin.

2.14 **Leukocytes:** Use serological pipette to overlay Ficoll with diluted Buffy Coat (25ml/50ml Tube) VERY SLOWLY, Tip: draw a 2-way "Y" shape on tube to reduce disruption of Ficoll interface. (***Figure 1****, #1 & 2*)

Gently manipulate tubes to avoid disrupting the interface; move to cent one at a time, with both hands

2.15 Centrifuge immediately **BREAK OFF** at 700g x 30 min RT (total ~35min).

Note: Post-spin there should be 3 distinct cell layers: The **MCN** (white layer between plasma and 1077), **PMN** (red band between 1119 and 1077) and the RBCs located at the bottom (Fig. 1.3)

WHILE GRADIENTS SPIN, RETRIEVE PLASMA AND SERUM SAMPLES AND ALIQUOT; keep necropsy room centrifuge lid closed to retain cold temperature for later use

Figure 1

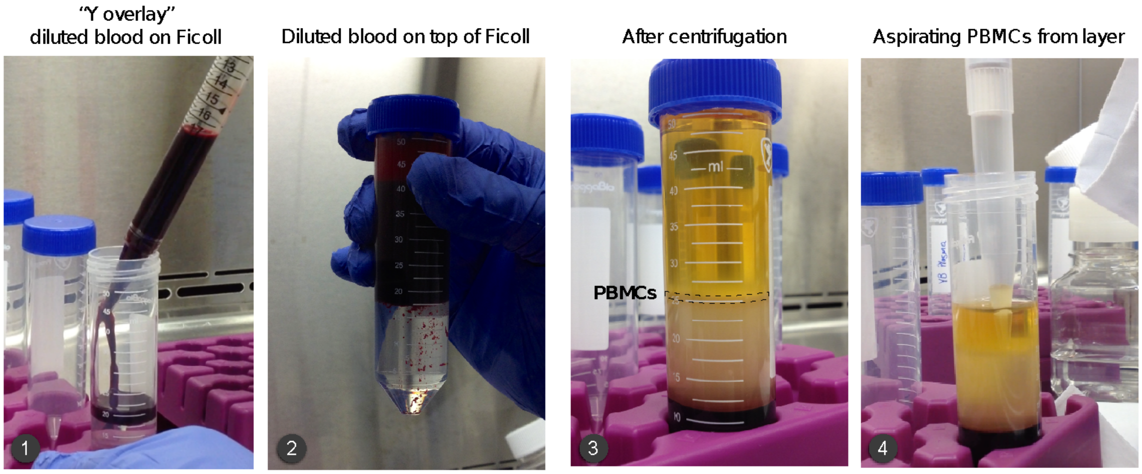

3. **PURIFYING PBMCs AND LEUKOCYTE SUB-TYPES**

3.1 **PBMCs:** After 15mL Ficoll tube centrifugation, mononuclear cells and platelets will be in a whitish layer just above the Ficoll layer. (***Figure 1****, #3*)

3.2 When centrifugation ends, immediately aspirate and discard about ¾ of the diluted plasma above the interface using a 5ml pipette

3.3 Collect cell layer with a 1ml pipette three times (***Figure 1****, #4*) and transfer to the 15ml "Cells" tube pre-filled with 2 ml of HBSS. QSP to 15ml with HBSS.

Note: Collection of cells immediately following centrifugation will yield best results. Ideally, cells are collected with as little Ficoll or plasma, however it is better to aspirate plasma than Ficoll.

3.4 **1* PBMC spin:** Pellet cells by centrifuging at 500g x 10 min RT
Spin along with Anna's 50 mL conical tube labeled .3

3.5 **2* PBMC spin:** After centrifugation, pour supernatant into waste beaker and tap tube against fist to resuspend cells

3.6 Add 15ml of HBSS.

3.7 Centrifuge at 200g x 10 min RT.

3.8 **3* PBMCs:** After centrifugation, pour out supernatant and tap tube against fist to resuspend cells.

3.9 Add 15ml of HBSS.

3.10 Centrifuge at 200g x 10 min (the third and final centrifugation)
Note *(morning):* Anna takes over here - leave in centrifuge

3.11 **Leukocytes:** After spin (700g x 30min), there should be 3 distinct cell layers: The **MCN** (top layer between 1077 and Plasma), **PMN** (middle layer between 1119 and 1077) and the RBCs located at the bottom.

3.12 Use a 5ml pipette to discard about ¾ of the diluted plasma above the interface.

3.13 Use a 1ml pipette to collect **MCN** (higher cell layer between plasma and ficoll) from both tubes and pool in the 50mL "**MCN**" tube pre-filled with 5ml HBSS.

3.14 Use a 1ml pipette to collect PMN (lower cell layer) from both tubes and pool in the 50mL "**PMN**" tube pre-filled with 5ml HBSS.
3.14.1 If **PMN** layer is bloody/poorly separated, be hesitant; too much blood clogs columns

3.15 Add HBSS to 50ml "**MCN**" and "**PMN**" tubes until = 50ml

3.16 Centrifuge **BREAK OFF** at 700g x 10 min RT.

3.17 **Leukocytes:** After centrifugation, pour supernatant into waste beaker, and tap tubes against fist to resuspend pellets. Note: For "**PMN**" supernatant use a serological pipette to aspirate. **DO NOT POUR**

3.18 Add 1ml of HBSS/BSA to 50ml "**MCN**" tube; pipette around to collect cells; transfer to 1.5ml "**M14**" tube.

3.19 Add 1ml of HBSS/BSA to 50ml "**PMN**" tube; pipette around to collect cells; transfer to 1.5ml "**P15**" tube.

3.20 Centrifuge both 1.5ml tubes at 700g x 40 sec RT to pellet cells.

3.21 For each tube, use 1ml pipette to discard supernatant and flick tube with finger to resuspend pellet

3.22 Add 240ul of HBSS/BSA; pipette up and down a few times.

3.23 **Monocyte Isolation:** Positive selection of monocytes, add 66.4 uL of magnetic bead labeled CD14 to 1.5ml "**M14**" tube. Invert by hand a few times.

3.24 **Neutrophil Isolation:** Positive selection of neutrophils, add 66.4 uL of magnetic bead labeled CD15 to 1.5ml "**P15**" tube. Invert by hand a few times.

3.25 Place in 4°C (fridge or on wet ice) and incubate for 15 min (set a timer).

MAC's Separation

3.26 **Leukocytes:** Place a MAC's Separator LS column over both the "**MCN-**" and "**PMN-**" 15mL tubes; label columns correspondingly (1 x "**MCN+**," 1 x "**PMN+**")

3.27 Equilibrate columns with 3ml of HBSS/BSA.

3.28 After **MCN**/**PMN** 15 min antibody incubation, add 1ml of HBSS/BSA and pipette up and down to wash

3.29 Centrifuge at 700g x 40 sec RT to pellet cells; should see pellet at the bottom of eppendorf

3.30 Use 1ml pipette to discard supernatant

3.31 Flick tube to resuspend cells

3.32 Add 1ml of HBSS/BSA to 1.5ml tube, pipette up and down, and then

3.33 Apply **MCN**/**PMN** cell suspension to the bottom of the coordinating **+**/**+** LS column, close to and directly above the filter

3.34 Wash column 3 X 3ml of HBSS/BSA. Collect cell suspension flow through and washes in the "**MCN-**" and "**PMN-**" 15mL tubes.

3.35 **Lymphocyte** isolation: Centrifuge the "**M14-**" tube 300g x 10 min RT

3.36 Discard the "**PMN-**" 15mL tube in sharps container.

3.37 Place the **MCN+**/**PMN+** LS columns over the corresponding 15ml **Mono+**/**Neut+** collection tubes

3.38 Pipette 5ml of HBSS buffer in column and immediately plunge the volume through with the piston. **Note: Mono+** and **Neut+** tubes can sit at room temperature until lymphocytes are purified.

3.39 **Lymphocyte** isolation: After spin, pour to discard supernatant

3.40 Tap tube against a closed fist to resuspend pellet

3.41 Add 1ml of HBSS/BSA; pipette around to collect cells; transfer to 1.5ml "**LYM**" tube

3.42 Centrifuge at 700g x 40 sec RT to pellet cells

3.43 Use 1ml pipette to discard supernatant

3.44 Flick tube with finger to resuspend pellet

3.45 Add 240ul of HBSS/BSA; pipette up and down a few times

3.46 Add 66.4ul of magnetic bead labeled CD61 (+) and 66.4ul of magnetic bead labeled CD235a (+). Invert a few times.

3.47 Incubate at 4°C for 15 min.

3.48 Place a MAC's Separator LS column over the 15mL "**LYM-**" tube, label "**LYM+**"

3.49 Equilibrate **LYM+** column with 3 ml HBSS/BSA.

Note: this is a good time to begin platelet isolation.

3.50 After **LYM** 15 min incubation with antibodies, add 1ml of HBSS/BSA and pipette up and down to wash

3.51 Centrifuge at 700g x 40 sec RT

3.52 Set centrifuge to 4°C, close lid, and press "fast temp" so it's ready for later

3.53 Use 1ml pipette to discard supernatant

Checkpoint #2:
- ☐ **Microcentrifuge at 4C**

3.54 Flick tube to resuspend cells

3.55 Add 1mL of HBSS/BSA, pipette up and down, then

3.56 Apply **LYM** cell suspension to the bottom of the **LYM+** LS column, close to and directly above the filter

3.57 Wash column 3 X 3ml of HBSS/BSA. Collect cell suspension flow through and washes in 15ml **LYM-** tube labeled lymphocyte (-). The lymphocyte (-) 9ml flow-through will be used for lymphocyte processing and the lymphocyte (+) column can be discarded.
Note: *The (-) flow-through should be labeled CD61(-) and CD235a (-) and discard (+) cells. This yields Lymphocytes (M14-, CD61-, CD235a-).*

3.58 Pellet **MCN**, PMN, and **LYM**: Centrifuge tubes at 700g for 10 min RT

3.59 Discard supernatant and tap tube to resuspend cells

3.60 Add 3ml of extracellular flux (XF) media from bead bath

3.61 Ensure tubes are labeled appropriately; place in fume hood for Anna

4. PLATELET ISOLATION

4.1 Centrifuge 15ml of PRP at 1,000g for 5 min RT to pellet RBCs. Transfer 90% to Clean PRP" 15ml tube.

4.2 Pellet "Clean PRP" at 1,500g for 10 min RT.

4.3 Pour out supernatant (plasma) and tap tube to resuspend cells.

4.4 Use 10mL sero tip to draw 10mL of PGI2 buffer; add 5mL to "Clean PRP," then pipette up and down to suspend platelets and mix; once mixed, dispense remaining 5mL of buffer (in total you add 10mL PGI2 for final [PGI2] of 1µM.

4.4.1 (Platelets can be left at room temperature at this point and processed later in procedure)

4.5 Centrifuge 1,500g for 10 min at room temperature.

4.6 Discard supernatant by pouring and tap tube to resuspend cells.

4.7 Add 3mL of PBS-PGI2 buffer
Note: Holding phase or platelets are ready for counting.

F.1 Cell storage

4.8 Store cells in pre-labeled box at -80°C until use.

4.9 Retrieve 1.5ml tube from tissue culture fridge and spin 2000g x 2 min in the pre-chilled micro-centrifuge at 4ºC (Bench Top, B2). Discard as much supernatant as possible without disturbing cell pellet. Store in -80.

4.10 Clean benches/hoods, discard & clean waste beaker, store supplies, turn off centrifuges.

Buccal swab processing:

4.11 Above bench, get new set of sterile blue tweezers

4.12 Use to move swab from 1.5ml ep from Catherine into labeled blue 2mL cryotube.

Checkpoint #3:
□ PBMC F.1 from fridge

End Time:

5. **SERUM & PLASMA COLLECTION**

5.1 If there is white film (fat) on top of the tubes, remove by aspirating with a 1ml pipette and discard.

5.2 With a 1 ml pipette, aspirate approximately 80% of the *Plasma* from each tube, and pool in a new 15ml conical tube labeled "Pooled Plasma." Discard the rest.

5.3 Repeat the procedure for *Serum*, transferring 80% of serum to a tube "Pooled Serum." Discard the rest.

5.4 Centrifuge the Pooled Plasma and Serum tubes at 2,000g x 10 min at 4ºC.

5.5 Aspirate 90% of the volume without disrupting the pellet, transfer to a new 15ml conical tube "Clean Plasma."

5.6 Repeat with Serum. Transfer to "Clean Serum."

5.7 Invert a few times to homogenize the content of the tubes. Keep on ice.

**Sample** ___________	**Date:** ___________	
Sample	Volume (ml)	Hemolysis (H), (M), (L)
Plasma Fasting .7		
Plasma Fasting .8		
Serum Fasting .5		

Serum Fasting .6		

Time Plasma Samples Put in Freezer:______________

Time Serum Samples Put in Freezer:______________

Note: Fasting Sal1.2 collected at blood draw, stored in lab at 4 degrees. Fasting Sal2.2 - 14.2 are delivered to lab by bag later in the day.

5.8 Spin Fasting Sal1.2-14.2 at 1000g x 5 min 4ºC

Fasting Sal 1.2		

Time Saliva Samples Put in Freezer:______________

Start Time:

STRESS Reactivity

6.

***For Total PBMC procedure follow from **Step 1.4**

6.1 Centrifuge ACD-A tube **BREAK OFF** at 500g x 15 min RT

6.2 Add 4mL Ficoll 1077 into a 15mL tube labeled “Ficoll 1077”.

6.3 After centrifugation, discard 3mL of plasma in waste beaker (draw from middle)

6.4 Pour tube into “diluted blood” tube

6.5 Use 5ml sero pipette to add 5mL of HBSS buffer to wash tube and collect cells, then transfer to “diluted blood” tube.

6.6 Use 10ml sero tip to draw the “diluted blood” tube and slowly overlay the Ficoll

6.7 Centrifuge immediately **BREAK OFF** at 400g x 30 min RT
Note: pre-load “PBMC” tube with HBSS.

6.8 Discard ¾ plasma.

6.9 Circle 4 times with 1mL pipette in white layer, transfer to “PBMC” tube pre-loaded with 2mL HBSS

6.10 Centrifuge 500g x 10 min RT (Break ON).

6.11 Pour out supernatant, tap tube to resuspend, add 15 mL of HBSS

Checkpoint #4:
- ☐ **Microcentrifuge at 4C**
- ☐ **Trypan blue in bath**

6.12 Centrifuge for 200g x 10 min RT

6.13 Pour out supernatant, tap tube to resuspend, add 15 mL of HBSS

6.14 Centrifuge for 200g x 10 min RT (the third and final centrifugation)

Note *(afternoon):* When 5 minutes left on spin, put Trypan Blue in the bead bath & retrieve cell ports

6.15 **(Afternoon) PBMCs:** Pour out supernatant, tap tube to resuspend.

6.16 Add 1ml of HBSS.

6.17 Add 10ul of 0.1% Trypan Blue to pre-labeled 1.5ml Eppendorf tube for each cell type.

6.18 Add 10ul of cell mixture directly into the 10ul Trypan Blue (1:1 dilution); pipette to mix

6.19 Pipette 10ul of the cell+Blue dilution into both ports A and B (Figure 2), and immediately perform cell counts on *Countess II* automated cell counter in bright field.

Note: Select BF as collect channel; uncheck DAPI and GFP. Wait until photo focuses, then click capture and record # of live cells.

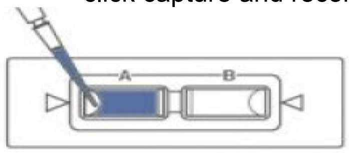

Note the cell concentration, and average cell size. Take the average of both values as the cell count

	**Stress Sample __________**			**Date:__________**		
	**Count #1**			**Recount (if required)**		
	Count (M cells/ml)	Size (µm)	Cell Death %	Count (M cells/ml)	Size (µm)	Cell Death %
**A**						
**B**						
**Average**						
% difference						
Proceed? (Must be 10% or less to proceed)	Yes / No			Yes / No		
Cell aliquots (5M)						

Cryostorage cell count						

Cell aliquots = 5M / [average] = take this from re-suspended cells to store in stress PBMC 1.5ml. Cryostorage cell count = the estimated amount of cells left in a 15mL PBMC tube after 5M are stored. Use 1mL pipette to measure what's leftover in the 15mL tube, then multiply by average (row 3 of table). On the tube, write ul of liquid left, and average (row 3), record here, and then put tube in urine FH.

6.20 Pipette cell aliquot volume (row 7) of cell suspension into prelabeled 1.5ml

6.21 Immediately centrifuge at 2000g for 2 min in the pre-chilled micro-centrifuge at 4°C (Bench Top, B2).

- Aspirate or discard as much supernatant as possible without disturbing cell pellet. Store in -80

Checkpoint #5:
- [ ] **PBMC S.1 in -80**

7. SERUM & PLASMA COLLECTION

***For Serum/Plasma isolation: spin 2,000g x 10min at 4°C (Necropsy Room), transfer ~80% of each tube into corresponding new 15ml conical tube labeled with timepoint -5 → 120; spin again 2,000g x 10 min at 4°C, transfer 90% of the volume - don't disrupt pellet - to new corresponding 15ml conical tube

- Invert a few times to homogenize the content of the tubes
- Keep on ice
- Starting from the <u>middle</u> of tube, pipette up and down 3 times, then aliquot 0.5ml to corresponding pre-labeled cryovial.

Note: **DO NOT DISCARD INITIAL PLASMA TUBES.** Keep them and place them in Anna's fume hood.

Label and set up plasma and serum tubes in this order:

-5	5	10	20	30	60	90	120
-5	5	10	20	30	60	90	120
-5	5	10	20	30	60	90	120
-5	5	10	20	30	60	90	120

SERUM & PLASMA OBSERVATION (+) ALIQUOTS

**Sample ___________**	**Date: ___________**	
Sample	Volume (ml)	Hemolysis (H), (M), (L)
**Plasma Fasting .7**		
**Plasma Fasting .8**		
**Serum Fasting .5**		
**Serum Fasting .6**		
Stress Plasma 1.3		
Stress Plasma 2.3		
Stress Plasma 3.3		
Stress Plasma 4.3		
Stress Plasma 5.3		
Stress Plasma 6.3		
Stress Plasma 7.3		
Stress Plasma 8.3		
Stress Serum 1.3		
Stress Serum 2.3		
Stress Serum 3.3		
Stress Serum 4.3		
Stress Serum 5.3		
Stress Serum 6.3		
Stress Serum 7.3		
Stress Serum 8.3		
**Fasting Sal 1.2**		
Fasting Sal 2.2		
Fasting Sal 3.2		
Fasting Sal 4.2		
Fasting Sal 5.2		
Fasting Sal 6.2		
Fasting Sal 7.2		
Fasting Sal 8.2		
Fasting Sal 9.2		

Fasting Sal 10.2		
**Fasting Sal 11.2**		
**Fasting Sal 12.2**		
**Fasting Sal 13.2**		
**Fasting Sal 14.2**		

Fasting saliva processing:

End of day checkpoint:
- ◻ PBMC F.1 in -80
- ◻ PBMC S.1 in -80
- ◻ Sal 1-14
- ◻ Buccal swab

Note: Fasting Sal1.2 collected at blood draw, stored in lab at 4 degrees. Fasting Sal2.2 - 14.2 are delivered to lab by bag later in the day.

7.1 Spin Fasting Sal1.2-14.2 at 1000g x 5 min 4°C

7.2 Aliquot samples into appropriate cryotube and store in MiSBIE sample box

Time Saliva Samples Put in Freezer:______________

Time Plasma Samples Put in Freezer:______________

Time Serum Samples Put in Freezer:______________

8. Wednesday morning: Urine processing

- *Room temperature PBS, ethanol, paper towels, 1000mL beaker, 50mL tubes and holder; all in main room fume hood*

8.1 Judge amount of urine; prepare coordinating number of 50mL tubes.
8.3 Pour whole urine into a 1000mL beaker; then aliquot appropriately into 50mL tubes.
8.4 Centrifuge tubes 1000g x 10 min RT (break ON). Note if two spins needed for the quantity.
8.5 Collect and store two cell-free 1.5mL urine samples.
8.6 Discard most of the remaining urine and check if the pellet is mobile or not. Resuspend cells from pellet and pool all cells into one **15 mL** tube. If sample getting caught in tip, use cut tips for bigger diameter
8.7 Fill with 15ml PBS, centrifuge 2000g x 10 min RT
8.9 Discard the supernatant and tap to resuspend the pellet
8.10 Add 200 uL of PBS by adding another tip and cutting ½ cm at the end. Resuspend cells a few times

8.11 Add 200ul of urine epithelial cells in each aliquot and store at -80C until further use.

**Sample ___________**			**Date:___________**		
Volume (mL)	Color	Precipitate	Pellet size	Pellet mobility	Turbidity

Urine was collected at hotel 8pm-8am in container containing 30ml acetic acid (Fisher Scientific, Cat# 22-130-554)

9. Thursday morning: Putting samples away

9.1 Get dry ice and put in large styrofoam MiSBIE box on a rolling cart (both from the Necropsy room)

9.2 From the -80 Freezer take out the 9x9 and 10x10 MiSBIE temp storage plastic boxes.

9.3 Put away cells in Liquid Nitrogen in order:

- PBMC F.1, PBMC F.2, Plt.1, Plt.2, Mono.1, Mono.2, Neut.1, Neut.2, Lym.1, Lym.2

9.4 Put away **Saliva.1** (Shelf 2 Rack 26), **.2** (Shelf 2 Rack __) and **.3** (Shelf 2 Rack 30)

9.5 Put away **Plasma Fasting.1** (Shelf 2 Rack 38) and **.2** (Shelf 2 Rack 39)

9.6 Put away **Serum Fasting.1** (Shelf 2 Rack 40) and**.2** (Shelf 2 Rack 41)

9.7 Put away **Plasma Stress.1** (Shelf 2 Rack 42), **.2** (Shelf 2 Rack 44), and **.3** (Shelf 2 Rack 46)

9.8 Put away **Serum Stress.1** (Shelf 2 Rack 48), **.2** (Shelf 3 Rack 57), and **.3** (Shelf 3 Rack 60)

9.9 Put away **Buccal swab** and **Urine Epithelial Cells** (Shelf 3 Rack 62) and **Urine** (shelf 3 Rack 61)

- Update MiSBIE Biobank
- Photograph each page of the protocol with notes/comments
- Upload pictures to corresponding participant folder in annual MiSBIE folder
- Send Catherine screenshot of saliva volume collection update

**Sample ___________**	**Date: ___________**
Sample	Volume (ml)
Dsal 1.2	
Dsal 2.2	
Dsal 3.2	
Dsal 4.2	
Dsal 5.2	
Dsal 6.2	
Dsal 7.2	
Dsal 8.2	
Dsal 9.2	
Dsal 10.2	
Dsal 11.2	
Dsal 12.2	

10. Diurnal saliva processing (when delivered by USPS):

10.1 Keep at RT; ideally process immediately, but definitely by end of day; note if not possible.

10.2 Centrifuge 1000g x 5 mins RT

10.3 Aliquot samples into corresponding cryovial, and store in -80

Fecal sample (should be delivered by USPS in same package as diurnal saliva):

10.4 Put on label (kept on bench)

10.5 Store in box in -80 (Shelf 3 Rack 62)

12.2 Trier Social Stress Task Day 1

Study Coordinator Instructions:

Speech task (PSO) – Do during end of set up period (2 min.)

Bring in camera, participant sheet, and pen, position mirror

Open camera and turn on

“The task we are going to have you do now is a speech task. I will give you a story and it is your task to make up a response to the situation. This is the situation I would like you to make up a story about:

Situation: *You are in a shopping mall and you pick up a wallet to look at it. All of sudden you feel a hand grab you on the shoulder, turn you around, and a voice say "Okay lady/sir, I saw you steal that wallet." You then realize that you are being accused by a department store security guard of stealing store merchandise, which of course, you didn't do. Describe what you will say to the judge in your own defense and include the following:*

1. *What you think should happen to the security guard for his error*
2. *What you think should happen to the department store for hiring the security guard*
3. *How you should be compensated.*

Here is a sheet of paper with those points on it. You have 2 minutes after I leave the room to prepare your speech. Dr. Morrison, the evaluator who will monitor your performance, will then come in and tell you when to begin. You have 3 minutes to give your speech while looking directly into the TV camera. We will be videotaping your speech so that we can rate your performance at a later time. You will need to speak for the whole 3 minutes because we want to measure the effects of speaking on heart rate and blood pressure. The evaluator will tell you when to stop. Do you have any questions? When you are giving your speech you should look directly into the camera as though you were being filmed for television. Remember to keep speaking for the entire 3 minutes. Please start your preparation period.

Give PASA (give directly after you explain instructions)

Participant Instructions:

You are in a shopping mall and you pick up a wallet to look at it. All of sudden you feel a hand grab you on the shoulder, turn you around, and a voice say "Okay sir/lady, I saw you steal that wallet." You then realize that you are being accused by a department store security guard of stealing store merchandise, which of course, you didn't do. Describe what you will say to the judge in your own defense and include the following:

1. What you think should happen to the security guard for his error
2. What you think should happen to the department store for hiring the security guard
3. How you should be compensated.

Evaluator Instructions:

1. General instructions
   - Do NOT smile, maintain a neutral facial expression
   - Maintain visual contact with participant
2. When entering the room:
   - Say "Hello" when you enter, no physical contact
   - Take the clipboard with the preparation sheet from participants.
3. Position yourself between the mirror and the camera, immediately in front of participants.
4. Once you are in position, participants should begin their speech:
   - Start timer
   - Say: "Please begin"
5. During the speech:
   - <u>Take notes for 10 seconds at each minute, throughout the speech task.</u>
   - <u>Make eye contact for the entire duration.</u>
   - If participants stops speaking for 30 seconds, prompt:

     "You still have time, please continue."

     "Keep speaking."
6. When 3 minutes are done, say "Your time is up.
7. Take the **camera**, **walk out** of the room without saying anything.

12.3 Verbal Time Estimation Task

Time Interval Estimation Instructions:

1. Participants sit in a comfortable chair in a quiet room with the study coordinator.

2. Participants are told, "We will run an exercise that looks at how you perceive time. This exercise is meant to help us understand your internal clock. There will be two different exercises, about 10 minutes each.

3. Participants must remove wristwatch and put away all devices that have a clock on it.

4. Participants are told, "For the first exercise, I will start a timer of a random period of time. When the timer ends and a sound plays, you will tell me how much time you think has passed. Please refrain from speaking during the task. This will take approximately 10 minutes altogether. Do you have any questions?"

5. When participants are ready, participants are told, "When I say start, tell me how much time has passed after the buzzer sounds... starting now." and initiates a timer with an amount of time indicated in the below table.

    a. Note: Study coordinator should not look at participant during the task.
    b. Note: Study coordinator should not look at the timer during the task.
    c. Note: Study coordinator should ensure that participants do not see data sheet. Use a clipboard oriented towards study coordinator.

6. Once the timer is up a sound plays and the study coordinator records the amount of time participants believe has passed.

7. This task is repeated to fill the below table, with a 5-second pause between each task where the study coordinator sets the next tasks timer and asks, "Are you ready for the next task?".

Time Interval Production Instructions:

1. Participants are told, "For the second exercise, I will tell you an amount of time, for example 30 seconds, and you will tell me when 30 seconds has passed. I will tell you when to start counting and simply tell me 'now' when you believe 30 seconds has passed. Please refrain from speaking during the task. These tasks will take approximately 10 minutes all together. Do you have any questions?"

2. When participants are ready, the study coordinator says "Tell me when X seconds have passed starting now." and initiates a timer starting at 0

3. When participants believe the allotted time has passed he/she says stops and the study coordinator stops the timer and records the elapsed time.

    a. Note: Study coordinator should not look at participant during the task.
    b. Note: Study coordinator should not look at the timer during the task.
    c. Note: Study coordinator should ensure that participants do not see data sheet. Use a clipboard oriented towards study coordinator.

d. Note: if there is a delay between participants saying stop and the study coordinator stopping of the timer, mark that down in notes.

4. This task is repeated to fill the below table, with a 5-second pause between each task, and asks, "Are you ready for the next task?"

12.4 Speech Task Day 2

Before scan start:

"In this task you will be preparing a speech which will be audiotaped immediately after the finish the scan. It will then be judged by a panel of peers on its persuasiveness, organization and intellectual quality. Your performance on the first speech task was slightly below average when compared with other participants' speeches. Try to be more persuasive when delivering this speech. There is a small chance you will be randomly selected to not give your speech."

Speech Instructions:

"The topic for your speech is:

You are driving down a neighborhood street when you suddenly realize that there is a police officer behind you with his red light flashing. When you pull over, the officer gives you a ticket for failing to stop at a stop sign the block before. You are sure that no stop sign existed. When you finally talk him into going back, so you can show him there is no sign there, you realize that there is a sign but it is almost totally hidden by the trees. The officer gives you a $50 ticket. Tell a story about the incident as if you were arguing your case in traffic court and include the following:

1. The events that led up to the officer giving you a ticker
2. Whether you should or should not have been given a ticker
3. The extent of the city's responsibility in keeping road signs in good view

After 2 minutes:

"You were randomly selected to not give your speech. Please relax for the rest of the scan. Remain still and focused on the cross."

12.5 Columbia Suicide Severity Rating Scale (C-SSRS)

The Columbia Suicide Severity Rating Scale uses simple, clear language and questions to determine the prevalence and severity of suicidal thoughts and behaviors. As mandated by the New York State Psychiatric Institute (NYSPI) we complete this scale at the start of each visit. For the purposes of our study, any rating > 4 requires contact with the study psychologist. The psychologist completes an assessment to determine immediate risk for participant, if necessary, the study visit is discontinued.

Always ask questions 1 and 2.	Past Month	
**1) Have you wished you were dead or wished you could go to sleep and not wake up?**		
**2) Have you actually had any thoughts about killing yourself?**		
If **YES** to 2, ask questions 3, 4, 5 and 6. If **NO** to 2, skip to question 6.		
**3) Have you been thinking about how you might do this?**		
**4) Have you had these thoughts and had some intention of acting on them?**	**High Risk**	
**5) Have you started to work out or worked out the details of how to kill yourself? Did you intend to carry out this plan?**	**High Risk**	
**Always Ask Question 6**	**Life-time**	**Past 3 Months**
**6) Have you done anything, started to do anything, or prepared to do anything to end your life?** *Examples*: Took pills, tried to shoot yourself, cut yourself, tried to hang yourself; or collected pills, obtained a gun, gave away valuables, wrote a will or suicide note, took out pills but didn't swallow any, held a gun but changed your mind or it was grabbed from your hand, went to the roof but didn't jump, etc. **If yes, was this within the past 3 months?**		**High Risk**

988 SUICIDE & CRISIS LIFELINE

If YES to 2 or 3, seek behavioral healthcare for further evaluation.
If the answer to 4, 5 or 6 is YES, get immediate help: Call or text 988, call 911 or go to the emergency room.
STAY WITH THEM until they can be evaluated.

Download Columbia Protocol app

12.6 Debriefing Script

Debriefing Form

First of all, thank you for participating in the MiSBIE study. We very much appreciate your time.

This study is designed to understand the interaction between the mind and the body. Everybody is different, and there are no right or wrong answers, or “better” or “worse” ways to respond to the different tasks. You did very well over the past two days [smile].

Yesterday we asked you to keep your hand in the bucket of ice water because this is “stressful” and makes the body react. To understand how you as a person react to this, we measured how your heart, skin, and different organs reacted in your body, sometimes by looking at hormones in blood and saliva. There was also a camera and someone wearing a white lab coat looking at you while you did this. We told you this person is Dr. Morrison and an expert in this procedure because most people feel evaluated or judged in this situation, and this also makes the body react in different ways. The camera was not recording, it was there just to increase the stress, called [say slowly] “socio-evaluative” stress. In fact, this person is a very friendly colleague of ours who was asked to look very serious, but he played the role of the evaluator so we can study how the body responds when we feel under pressure, or stress.

Likewise, today when you were in the MRI scanner, we asked you to recognize words, to prepare a speech, and we put the cold wrap on your arm to elicit the same stress response. This was important to understand how the body processes stress. When you were told that you were randomly selected not to give a speech, this was a structured component of the study to help us better understand what was happening [emphasize] while you were preparing the speech.

If people are told from the beginning about what is going to happen in a study like this, they tend to get stressed right away just by thinking about it, this is called anticipation. This prevents us from studying the body when you are relaxed, and then during and after a stressor. It was therefore necessary not to tell you everything that was going to happen, and make you believe that you were being evaluated so we can understand the cross-talk between the mind and the body.

We really appreciate your participation in this part of the study.

We'll give you a little package to go home with to collect saliva samples. This part of the study is just as important as the one you just completed.

Do you have any question or is there anything you are not clear about?
[Time for questions, discussion with study coordinator]

If anything comes to mind in the coming days you can always contact the study coordinator by phone or email. You have the contact information on the consent form and on the MiSBIE brochure.

12.7 Breakfast and Lunch Menu

Both breakfast and lunch meals were selected from a restricted study menu to avoid large differences in meal types between participants. Breakfast was typically eaten around 10 am, lunch was typically eaten around noon.

Breakfast Menu

Please select from the menu below what you would like for breakfast in the morning on the two days you will be participating in this study. Please choose one entree and up to two other items.

**Do you typically drink caffeine in the morning? (coffee, tea)**	◯ Yes<br>◯ No
**Entree**	◯ Cereal<br>◯ Bagel
**Yogurt**	☐ Strawberry<br>☐ Raspberry<br>☐ Peach<br>☐ Blueberry
**Fruit**	☐ Banana<br>☐ Cheese and Grapes<br>☐ Pineapple

Lunch Menu

Please select from the menu below what you would like for lunch on the two days you will be participating in this study. Choose one entree and up to 3 other items.

**Entree**	◯ Caesar Salad (croutons, parmesan, roma lettuce blend, caesar dressing)<br>◯ Greek Salad (feta, kalamata olives, grape tomatoes, red onions, cucumbers, roma lettuce blend, champagne vinaigrette)<br>◯ Southwestern Chicken Salad (southwestern seasoned chicken breast, pepperjack, black bean-corn salsa, pico de gallo, mesclun greens, ranch dressing)<br>◯ Tuna and Cucumber Sandwich (tuna, mayo, mustard, celery, onion, salt, pepper)<br>◯ Grilled Chicken and Cheddar Sandwich (grilled chicken breast, cheddar, lettuce, tomato, honey mustard, plain hero)<br>◯ Roasted Turkey and Swiss Sandwich (roasted turkey, swiss, lettuce, honey mustard, multigrain hero)<br>reset
**Yogurt**	☐ Strawberry<br>☐ Raspberry<br>☐ Peach<br>☐ Blueberry
**Fruit**	☐ Banana<br>☐ Cheese and Grapes<br>☐ Pineapple
**Other Snacks**	☐ Assorted Nuts and Dried Fruit<br>☐ Hummus with Pretzels<br>☐ Regular Salted Potato Chips

References

Acin-Perez, R., Beninca, C., Shabane, B., Shirihai, O. S., & Stiles, L. (2021). Utilization of Human Samples for Assessment of Mitochondrial Bioenergetics: Gold Standards, Limitations, and Future Perspectives. *Life (Basel)*, *11*(9). https://doi.org/10.3390/life11090949

Anon. (1996). Heart rate variability. Standards of measurement, physiological interpretation, and clinical use. Task Force of the European Society of Cardiology and the North American Society of Pacing and Electrophysiology. *Eur Heart J*, *17*(3), 354-381. https://www.ncbi.nlm.nih.gov/pubmed/8737210

Barch, D. M., Burgess, G. C., Harms, M. P., Petersen, S. E., Schlaggar, B. L., Corbetta, M., Glasser, M. F., Curtiss, S., Dixit, S., Feldt, C., Nolan, D., Bryant, E., Hartley, T., Footer, O., Bjork, J. M., Poldrack, R., Smith, S., Johansen-Berg, H., Snyder, A. Z., . . . Consortium, W. U.-M. H. (2013). Function in the human connectome: task-fMRI and individual differences in behavior. *Neuroimage*, *80*, 169-189. https://doi.org/10.1016/j.neuroimage.2013.05.033

Beaudart, C., Rolland, Y., Cruz-Jentoft, A. J., Bauer, J. M., Sieber, C., Cooper, C., Al-Daghri, N., Araujo de Carvalho, I., Bautmans, I., Bernabei, R., Bruyère, O., Cesari, M., Cherubini, A., Dawson-Hughes, B., Kanis, J. A., Kaufman, J. M., Landi, F., Maggi, S., McCloskey, E., . . . Fielding, R. A. (2019). Assessment of Muscle Function and Physical Performance in Daily Clinical Practice : A position paper endorsed by the European Society for Clinical and Economic Aspects of Osteoporosis, Osteoarthritis and Musculoskeletal Diseases (ESCEO). *Calcif Tissue Int*, *105*(1), 1-14. https://doi.org/10.1007/s00223-019-00545-w

Block, R. A., Zakay, D., & Hancock, P. A. (1998). Human aging and duration judgments: a meta-analytic review. *Psychol Aging*, *13*(4), 584-596. https://doi.org/10.1037//0882-7974.13.4.584

Campos Munoz A, V. S., Gupta M. (2022). *Orthostasis*.

Carrasco, M. C., Bernal, M. C., & Redolat, R. (2001). Time estimation and aging: a comparison between young and elderly adults. *Int J Aging Hum Dev*, *52*(2), 91-101. https://doi.org/10.2190/7nfl-cgcp-g9e1-p0h1

Centers for Disease Control and Prevention, N. C. f. I. P. a. C. (2017). *Assessment 30-Second Chair Stand*. https://www.cdc.gov/steadi/pdf/STEADI-Assessment-30Sec-508.pdf

D. A. Karnofsky, B., J.H.,. (1949). The Clinical Evaluation of Chemotherapeutic Agents in Cancer. In E. C. M. MacLeod (Ed.), *Evaluation of Chemotherapeutic Agents* (pp. 196). Columbia University Press.

DeBoer, R. W., Karemaker, J. M., & Strackee, J. (1984). Comparing spectra of a series of point events particularly for heart rate variability data. *IEEE Trans Biomed Eng*, *31*(4), 384-387. https://doi.org/10.1109/tbme.1984.325351

Desousa, B. R., Kim, K. K., Jones, A. E., Ball, A. B., Hsieh, W. Y., Swain, P., Morrow, D. H., Brownstein, A. J., Ferrick, D. A., Shirihai, O. S., Neilson, A., Nathanson, D. A., Rogers, G. W., Dranka, B. P., Murphy, A. N., Affourtit, C., Bensinger, S. J., Stiles, L., Romero, N., & Divakaruni, A. S. (2023). Calculation of ATP production rates using the Seahorse XF Analyzer. *EMBO Rep*, *24*(10), e56380. https://doi.org/10.15252/embr.202256380

Distelmaier, F., & Klopstock, T. (2023). Neuroimaging in mitochondrial disease. *Handb Clin Neurol*, *194*, 173-185. https://doi.org/10.1016/B978-0-12-821751-1.00016-6

Drobyshevsky, A., Baumann, S. B., & Schneider, W. (2006). A rapid fMRI task battery for mapping of visual, motor, cognitive, and emotional function. *Neuroimage*, *31*(2), 732-744. https://doi.org/10.1016/j.neuroimage.2005.12.016

Eisenbarth, H., Chang, L. J., & Wager, T. D. (2016). Multivariate Brain Prediction of Heart Rate and Skin Conductance Responses to Social Threat. *J Neurosci*, *36*(47), 11987-11998. https://doi.org/10.1523/JNEUROSCI.3672-15.2016

Espinosa-Fernández, L., Miró, E., Cano, M., & Buela-Casal, G. (2003). Age-related changes and gender differences in time estimation. *Acta Psychol (Amst)*, *112*(3), 221-232. https://doi.org/10.1016/s0001-6918(02)00093-8

EV Osilla , J. M., S. Sharma. (2022). *Temperature Regulation*. StatPearls. https://www.ncbi.nlm.nih.gov/books/NBK507838/

Forkel, S. J., & Catani, M. (2017). Structural Neuroimaging. In *Research Methods in Psycholinguistics and the Neurobiology of Language* (pp. 288-309). https://doi.org/https://doi.org/10.1002/9781394259762.ch15

Frisch, J. U., Hausser, J. A., & Mojzisch, A. (2015). The Trier Social Stress Test as a paradigm to study how people respond to threat in social interactions. *Front Psychol*, *6*, 14. https://doi.org/10.3389/fpsyg.2015.00014

Gao, W., Stalder, T., Foley, P., Rauh, M., Deng, H., & Kirschbaum, C. (2013). Quantitative analysis of steroid hormones in human hair using a column-switching LC-APCI-MS/MS assay. *J Chromatogr B Analyt Technol Biomed Life Sci*, *928*, 1-8. https://doi.org/10.1016/j.jchromb.2013.03.008

Gao, W., Stalder, T., & Kirschbaum, C. (2015). Quantitative analysis of estradiol and six other steroid hormones in human saliva using a high throughput liquid chromatography-tandem mass spectrometry assay. *Talanta*, *143*, 353-358. https://doi.org/10.1016/j.talanta.2015.05.004

George I. Christopoulos, M. A. U., and Wei Jie Yap. (2016). The Body and the Brain: Measuring Skin Conductance Responses to Understand the Emotional Experience. *Organizational Reserach Methods*(22(1)), 394-420. https://doi.org/https://doi.org/10.1177/1094428116681073

Geuter, S., Lindquist, M. A., & Wager, T. D. . (2016). Fundamentals of Functional Neuroimaging. In L. G. T. J. T. Cacioppo, & G. G. Berntson (Ed.), *Handbook of Psychophysiology* (pp. pp. 41–73). Cambridge University Press.

Graham, B. L., Steenbruggen, I., Miller, M. R., Barjaktarevic, I. Z., Cooper, B. G., Hall, G. L., Hallstrand, T. S., Kaminsky, D. A., McCarthy, K., McCormack, M. C., Oropez, C. E., Rosenfeld, M., Stanojevic, S., Swanney, M. P., & Thompson, B. R. (2019). Standardization of Spirometry 2019 Update. An Official American Thoracic Society and European Respiratory Society Technical Statement. *Am J Respir Crit Care Med*, *200*(8), e70-e88. https://doi.org/10.1164/rccm.201908-1590ST

Hampson, M., Driesen, N. R., Skudlarski, P., Gore, J. C., & Constable, R. T. (2006). Brain connectivity related to working memory performance. *J Neurosci*, *26*(51), 13338-13343. https://doi.org/10.1523/JNEUROSCI.3408-06.2006

Hansen, J. Y., Shafiei, G., Markello, R. D., Smart, K., Cox, S. M. L., Norgaard, M., Beliveau, V., Wu, Y., Gallezot, J. D., Aumont, E., Servaes, S., Scala, S. G., DuBois, J. M., Wainstein, G., Bezgin, G., Funck, T., Schmitz, T. W., Spreng, R. N., Galovic, M., . . . Misic, B. (2022). Mapping neurotransmitter systems to the structural and functional organization of the human neocortex. *Nat Neurosci*, *25*(11), 1569-1581. https://doi.org/10.1038/s41593-022-01186-3

Hou, Y., Xie, Z. Y., Zhao, X. T., Yuan, Y., Dou, P., & Wang, Z. X. (2019). Appendicular skeletal muscle mass: A more sensitive biomarker of disease severity than BMI in adults with mitochondrial diseases. *Plos One*, *14*(7). https://doi.org/ARTN e0219628
10.1371/journal.pone.0219628

Jarbrink-Sehgal, E., & Andreasson, A. (2020). The gut microbiota and mental health in adults. *Curr Opin Neurobiol*, *62*, 102-114. https://doi.org/10.1016/j.conb.2020.01.016

Jezova, D., Makatsori, A., Duncko, R., Moncek, F., & Jakubek, M. (2004). High trait anxiety in healthy subjects is associated with low neuroendocrine activity during psychosocial stress. *Prog Neuropsychopharmacol Biol Psychiatry*, *28*(8), 1331-1336. https://doi.org/10.1016/j.pnpbp.2004.08.005

Joachim Taelman, S. V., A. Spaepen & S. Van Huffel (2009). Influence of Mental Stress on Heart Rate and Heart Rate Variability. *International Federation for Medical and Biological Engineering*, *22*(4), 1366–1369.

Kaufmann, P., Engelstad, K., Wei, Y., Kulikova, R., Oskoui, M., Battista, V., Koenigsberger, D. Y., Pascual, J. M., Sano, M., Hirano, M., DiMauro, S., Shungu, D. C., Mao, X., & De Vivo, D. C. (2009). Protean phenotypic features of the A3243G mitochondrial DNA mutation. *Arch Neurol*, *66*(1), 85-91. https://doi.org/10.1001/archneurol.2008.526

Kaufmann, P., Engelstad, K., Wei, Y., Kulikova, R., Oskoui, M., Sproule, D. M., Battista, V., Koenigsberger, D. Y., Pascual, J. M., Shanske, S., Sano, M., Mao, X., Hirano, M., Shungu, D. C., Dimauro, S., & De Vivo, D. C. . (2011). Natural history of MELAS associated with mitochondrial DNA m.3243A>G genotype. *Neurology*, *77*(22), 1965–1971. https://doi.org/https://doi.org/10.1212/WNL.0b013e31823a0c7f

Kaufmann, P., Shungu, D. C., Sano, M. C., Jhung, S., Engelstad, K., Mitsis, E., Mao, X., Shanske, S., Hirano, M., DiMauro, S., & De Vivo, D. C. (2004). Cerebral lactic acidosis correlates with neurological impairment in MELAS. *Neurology*, *62*(8), 1297-1302. https://doi.org/10.1212/01.wnl.0000120557.83907.a8

Khanna, D., Peltzer, C., Kahar, P., & Parmar, M. S. (2022). Body Mass Index (BMI): A Screening Tool Analysis. *Cureus*, *14*(2), e22119. https://doi.org/10.7759/cureus.22119

Kirchner, W. K. (1958). Age differences in short-term retention of rapidly changing information. *J Exp Psychol*, *55*(4), 352-358. https://doi.org/10.1037/h0043688

Kirschbaum, C. (2017). *TU Dresden Biopsychology - How to Collect a Hair Sample* https://www.youtube.com/watch?v=8Jf_aIDtz4o

Kirschbaum, C., & Hellhammer, D. H. (1994). Salivary cortisol in psychoneuroendocrine research: recent developments and applications. *Psychoneuroendocrinology*, *19*(4), 313-333. https://doi.org/10.1016/0306-4530(94)90013-2

Klein, I. L., van de Loo, K. F. E., Smeitink, J. A. M., Janssen, M. C. H., Kessels, R. P. C., van Karnebeek, C. D., van der Veer, E., Custers, J. A. E., & Verhaak, C. M. (2021). Cognitive functioning and mental health in mitochondrial disease: A systematic scoping review. *Neurosci Biobehav Rev*, *125*, 57-77. https://doi.org/10.1016/j.neubiorev.2021.02.004

Kuriyan, R. (2018). Body composition techniques. *Indian J Med Res*, *148*(5), 648-658. https://doi.org/10.4103/ijmr.IJMR_1777_18

Lopez-Sola, M., Woo, C. W., Pujol, J., Deus, J., Harrison, B. J., Monfort, J., & Wager, T. D. (2017). Towards a neurophysiological signature for fibromyalgia. *Pain*, *158*(1), 34-47. https://doi.org/10.1097/j.pain.0000000000000707

Michelson, J., Rausser, S., Peng, A., Yu, T., Sturm, G., Trumpff, C., Kaufman, B. A., Rai, A. J., & Picard, M. (2023). MitoQuicLy: A high-throughput method for quantifying cell-free DNA from human plasma, serum, and saliva. *Mitochondrion*, *71*, 26-39. https://doi.org/10.1016/j.mito.2023.05.001

Moggio, M., Colombo, I., Peverelli, L., Villa, L., Xhani, R., Testolin, S., Di Mauro, S., & Sciacco, M. (2014). Mitochondrial disease heterogeneity: a prognostic challenge. *Acta Myol*, *33*(2), 86-93. https://www.ncbi.nlm.nih.gov/pubmed/25709378

Mookerjee, S. A., Gerencser, A. A., Nicholls, D. G., & Brand, M. D. (2017). Quantifying intracellular rates of glycolytic and oxidative ATP production and consumption using extracellular flux measurements. *J Biol Chem*, *292*(17), 7189-7207. https://doi.org/10.1074/jbc.M116.774471

Moore, V. C. (2012). Spirometry: step by step. *Breathe*, *8*(3), 232-240. https://doi.org/10.1183/20734735.0021711

Nicolo, A., Massaroni, C., Schena, E., & Sacchetti, M. (2020). The Importance of Respiratory Rate Monitoring: From Healthcare to Sport and Exercise. *Sensors (Basel)*, *20*(21). https://doi.org/10.3390/s20216396

Patel, N., Smith, C. E., Pinchak, A. C., & Hagen, J. F. (1996). Comparison of esophageal, tympanic, and forehead skin temperatures in adult patients. *J Clin Anesth*, *8*(6), 462-468. https://doi.org/10.1016/0952-8180(96)00103-1

Porcelli, A. J. (2014). An alternative to the traditional cold pressor test: the cold pressor arm wrap. *J Vis Exp*(83), e50849. https://doi.org/10.3791/50849

Rahma, O. N., Putra, A. P., Rahmatillah, A., Putri, Y., Fajriaty, N. D., Ain, K., & Chai, R. (2022). Electrodermal Activity for Measuring Cognitive and Emotional Stress Level. *J Med Signals Sens*, *12*(2), 155-162. https://doi.org/10.4103/jmss.JMSS_78_20

Renee D Goodwin 1, S. C., Nicole Simuro, Mark Davies, Daniel S Pine. (2007). Association between lung function and mental health problems among adults in the United States: findings from the First National Health and Nutrition Examination Survey. *American Journal of Epidemiology*(165(4)), 383-388. https://doi.org/10.1093/aje/kwk026

Rieder, R., Wisniewski, P. J., Alderman, B. L., & Campbell, S. C. (2017). Microbes and mental health: A review. *Brain Behav Immun*, *66*, 9-17. https://doi.org/10.1016/j.bbi.2017.01.016

Russell, E., Koren, G., Rieder, M., & Van Uum, S. (2012). Hair cortisol as a biological marker of chronic stress: current status, future directions and unanswered questions. *Psychoneuroendocrinology*, *37*(5), 589-601. https://doi.org/10.1016/j.psyneuen.2011.09.009

Russo, M. A., Santarelli, D. M., & O'Rourke, D. (2017). The physiological effects of slow breathing in the healthy human. *Breathe (Sheff)*, *13*(4), 298-309. https://doi.org/10.1183/20734735.009817

Schaefer, A. M., Phoenix, C., Elson, J. L., McFarland, R., Chinnery, P. F., & Turnbull, D. M. (2006). Mitochondrial disease in adults: a scale to monitor progression and treatment. *Neurology*, *66*(12), 1932-1934. https://doi.org/10.1212/01.wnl.0000219759.72195.41

Schubert, C., Lambertz, M., Nelesen, R. A., Bardwell, W., Choi, J. B., & Dimsdale, J. E. (2009). Effects of stress on heart rate complexity--a comparison between short-term and chronic stress. *Biol Psychol*, *80*(3), 325-332. https://doi.org/10.1016/j.biopsycho.2008.11.005

Seeley, W. W. (2019). The Salience Network: A Neural System for Perceiving and Responding to Homeostatic Demands. *J Neurosci*, *39*(50), 9878-9882. https://doi.org/10.1523/JNEUROSCI.1138-17.2019

Shin, M., Swan, P., & Chow, C. M. (2015). The validity of Actiwatch2 and SenseWear armband compared against polysomnography at different ambient temperature conditions. *Sleep Sci*, *8*(1), 9-15. https://doi.org/10.1016/j.slsci.2015.02.003

Spruill, T. M. (2010). Chronic psychosocial stress and hypertension. *Curr Hypertens Rep*, *12*(1), 10-16. https://doi.org/10.1007/s11906-009-0084-8

Stalder, T., Lupien, S. J., Kudielka, B. M., Adam, E. K., Pruessner, J. C., Wust, S., Dockray, S., Smyth, N., Evans, P., Kirschbaum, C., Miller, R., Wetherell, M. A., Finke, J. B., Klucken, T., & Clow, A. (2022). Evaluation and update of the expert consensus guidelines for the assessment of the cortisol awakening response (CAR). *Psychoneuroendocrinology*, *146*, 105946. https://doi.org/10.1016/j.psyneuen.2022.105946

Sterling, P. E., J. (1988). Allostasis: A New Paradigm to Explain Arousal Pathology. In *Handbook of of Life Stress, Cognition and Health* (pp. 629-649). John Wiley & Sons.

Sturm, G., Karan, K. R., Monzel, A. S., Santhanam, B., Taivassalo, T., Bris, C., Ware, S. A., Cross, M., Towheed, A., Higgins-Chen, A., McManus, M. J., Cardenas, A., Lin, J., Epel, E. S., Rahman, S., Vissing, J., Grassi, B., Levine, M., Horvath, S., . . . Picard, M. (2023). OxPhos defects cause hypermetabolism and reduce lifespan in cells and in patients with

mitochondrial diseases. *Commun Biol*, *6*(1), 22. https://doi.org/10.1038/s42003-022-04303-x

Trumpff, C., Rausser, S., Haahr, R., Karan, K. R., Gouspillou, G., Puterman, E., Kirschbaum, C., & Picard, M. (2022). Dynamic behavior of cell-free mitochondrial DNA in human saliva. *Psychoneuroendocrinology*, *143*, 105852. https://doi.org/10.1016/j.psyneuen.2022.105852

Vierck, C. J., Whitsel, B. L., Favorov, O. V., Brown, A. W., & Tommerdahl, M. (2013). Role of primary somatosensory cortex in the coding of pain. *Pain*, *154*(3), 334-344. https://doi.org/10.1016/j.pain.2012.10.021

von Baeyer, C. L., Piira, T., Chambers, C. T., Trapanotto, M., & Zeltzer, L. K. (2005). Guidelines for the cold pressor task as an experimental pain stimulus for use with children. *J Pain*, *6*(4), 218-227. https://doi.org/10.1016/j.jpain.2005.01.349

von Salis, S., Ehlert, U., & Fischer, S. (2021). Altered Experienced Thermoregulation in Depression-No Evidence for an Effect of Early Life Stress. *Front Psychiatry*, *12*, 620656. https://doi.org/10.3389/fpsyt.2021.620656

Wager, T. D., van Ast, V. A., Hughes, B. L., Davidson, M. L., Lindquist, M. A., & Ochsner, K. N. (2009). Brain mediators of cardiovascular responses to social threat, part II: Prefrontal-subcortical pathways and relationship with anxiety. *Neuroimage*, *47*(3), 836-851. https://doi.org/10.1016/j.neuroimage.2009.05.044

Wager, T. D., Waugh, C. E., Lindquist, M., Noll, D. C., Fredrickson, B. L., & Taylor, S. F. (2009). Brain mediators of cardiovascular responses to social threat: part I: Reciprocal dorsal and ventral sub-regions of the medial prefrontal cortex and heart-rate reactivity. *Neuroimage*, *47*(3), 821-835. https://doi.org/10.1016/j.neuroimage.2009.05.043

Walkowiak, B., Kesy, A., & Michalec, L. (1997). Microplate reader--a convenient tool in studies of blood coagulation. *Thromb Res*, *87*(1), 95-103. https://doi.org/10.1016/s0049-3848(97)00108-4

WHO. (2008). *Waist Circumference and Waist-Hip Ratio Report of a WHO Expert Consultation*. World Health Organization 2011. https://www.who.int/publications/i/item/9789241501491

Wright, B. J., O'Brien, S., Hazi, A., & Kent, S. (2014). Increased systolic blood pressure reactivity to acute stress is related with better self-reported health. *Sci Rep*, *4*, 6882. https://doi.org/10.1038/srep06882

Yang, X., Heinemann, M., Howard, J., Huber, G., Iyer-Biswas, S., Le Treut, G., Lynch, M., Montooth, K. L., Needleman, D. J., Pigolotti, S., Rodenfels, J., Ronceray, P., Shankar, S., Tavassoly, I., Thutupalli, S., Titov, D. V., Wang, J., & Foster, P. J. (2021). Physical

bioenergetics: Energy fluxes, budgets, and constraints in cells. *Proc Natl Acad Sci U S A*, *118*(26). https://doi.org/10.1073/pnas.2026786118

Yuri Masaoka, A. K. I. H. (2001). *Anxiety and Respiration* Respiration and Emotion, Springer, Tokyo.

Zamoscik, V., Schmidt, S. N. L., Timm, C., Kuehner, C., & Kirsch, P. (2020). Modulation of respiration pattern variability and its relation to anxiety symptoms in remitted recurrent depression. *Heliyon*, *6*(7), e04261. https://doi.org/10.1016/j.heliyon.2020.e04261
